# Comparative Assessment of Intrinsic Disorder Predictions with a Focus on Protein and Nucleic Acid-Binding Proteins

**DOI:** 10.3390/biom10121636

**Published:** 2020-12-04

**Authors:** Akila Katuwawala, Lukasz Kurgan

**Affiliations:** Department of Computer Science, Virginia Commonwealth University, Richmond, VA 23284, USA; KatuwawalaAI@vcu.edu

**Keywords:** intrinsic disorder, intrinsically disordered proteins, prediction, protein-protein interactions, protein-nucleic acids interactions, predictive performance

## Abstract

With over 60 disorder predictors, users need help navigating the predictor selection task. We review 28 surveys of disorder predictors, showing that only 11 include assessment of predictive performance. We identify and address a few drawbacks of these past surveys. To this end, we release a novel benchmark dataset with reduced similarity to the training sets of the considered predictors. We use this dataset to perform a first-of-its-kind comparative analysis that targets two large functional families of disordered proteins that interact with proteins and with nucleic acids. We show that limiting sequence similarity between the benchmark and the training datasets has a substantial impact on predictive performance. We also demonstrate that predictive quality is sensitive to the use of the well-annotated order and inclusion of the fully structured proteins in the benchmark datasets, both of which should be considered in future assessments. We identify three predictors that provide favorable results using the new benchmark set. While we find that VSL2B offers the most accurate and robust results overall, ESpritz-DisProt and SPOT-Disorder perform particularly well for disordered proteins. Moreover, we find that predictions for the disordered protein-binding proteins suffer low predictive quality compared to generic disordered proteins and the disordered nucleic acids-binding proteins. This can be explained by the high disorder content of the disordered protein-binding proteins, which makes it difficult for the current methods to accurately identify ordered regions in these proteins. This finding motivates the development of a new generation of methods that would target these difficult-to-predict disordered proteins. We also discuss resources that support users in collecting and identifying high-quality disorder predictions.

## 1. Introduction

Intrinsically disordered regions (IDRs) are functional regions in a protein sequence that lack a stable structure in isolation and instead exist as dynamic ensembles of conformations [1,2,3]. The “intrinsically disordered” term originated from the fact that these regions cannot be resolved due to variable or fluctuating positions with the crystal lattice, while “intrinsic” denotes the fact that disordered state is encoded in the protein sequence. Proteins that include of one or more IDRs are referred to as the intrinsically disordered proteins. Intrinsically disordered proteins carry out a variety of cellular and biochemical functions [4,5,6] but they can be also associated with a wide range of pathogenic conditions [7,8]. Recent computational studies reveal that disorder is particularly abundant in eukaryotic proteins, where as many as 19% of residues are estimated to be disordered [9] and where up to 50% of proteins, depending on the specific organism, include one or more long disordered region (30 or more amino acids in length) [9,10,11,12,13]. The presence of the intrinsic disorder is considered to be the driving factor that defines dark proteomes [14,15,16]. IDRs are associated with a wide range of cellular functions including signal transduction, molecular assembly, molecular recognition, cell cycle regulation, transcription, translation, and viral cycle regulation, to name just a few [4,5,6,17,18,19,20,21,22,23,24,25,26,27,28,29]. One of the key hallmarks of the disordered regions is their ability to fold upon binding to their physiological partner(s) [6,30,31,32,33,34,35]. A major portion of these interactions involves binding to DNA, RNA and protein partners [19,21,22,34,36,37,38,39,40].

Several databases, such as DisProt [41], MobiDB [42], Protein Data Bank (PDB) [43] and Intrinsically Disordered proteins with Extensive Annotations and Literature (IDEAL) [44] provide access to the experimentally annotated IDRs. However, they are relatively small. DisProt, IDEAL and PDB include annotations for about 1600 [41], 1000 [44], and 26,000 [45] proteins, respectively. These proteins correspond to only about 0.016% of the current protein universe that is represented by the 188 million proteins in the 2020_04 release of the UniProt database [46]. Closing this large and continuously growing annotation gap can be facilitated by computational predictors of disorder. Well over 60 disorder predictors have been developed to date [47,48,49,50]. Some of the arguably more popular and accurate predictors include (in alphabetical order): CSpritz [51], disCoP [52], DisEMBL [53], DisMeta [54], DISOPRED [55,56,57], DISpro [58,59], ESpritz [60], GlobPlot [61], IUPred [62,63,64], MD [65], MetaDisorder [66], MFDp [67,68,69], MobiDB-lite [70], PONDR-FIT [71], PrDOS [72], PreDisorder [73], SPOT-Disorder [74,75,76], and VSL2B [77,78]. Moreover, pre-computed disorder predictions can be obtained from two large databases: MobiDB [42] and Database of Disorder Protein Predictions (D^2^P^2^) [13]. Users also benefit from the availability of a comprehensive disorder prediction webserver, DisorderEd PredictIon CenTER (DEPICTER) [79], which generates predictions for a wide range of tools including IUPred, the fast version of SPOT-Disorder (SPOT-Disorder-Single [76]), and several specialized disorder function predictors, such as DFLpred [80], DMRpred [81], DisoRDPbind [82,83], fMoRFpred [30], and ANCHOR2 [64].

Predictive performance of the disorder predictors was assessed in several comparative studies [45,84,85,86,87,88,89,90,91,92,93]. These studies, which were comprehensively surveyed in a recent article [50], guide users in the selection of accurate predictors and inform users and developers about current levels of predictive quality offered by the best available tools. The latter fuels progress in the development of gradually more accurate tools. To this end, the predictive quality measured with Area Under the ROC Curve (AUC) have risen from the 0.73–0.79 range in the mid-2000s, to the 0.85–0.90 range that was secured by the methods that were published in the last four years [3]. While these past comparative studies provide invaluable insights, they also share a few drawbacks. **First**, they perform assessment using a generic set of proteins while they rarely (i.e., only once [92]) evaluate performance for specific functional protein families. **Second**, they overlook an important aspect of the similarity between the benchmark dataset and the training datasets that were used to develop the tested predictors. High levels of similarity may distort the results by favoring certain methods for which the similarity is higher, and may result in an overestimation of the predictive performance; we demonstrate that in this article. **Third**, some of the past comparative studies that source their data from the DisProt database [84,91] perform assessment by assuming that the sequence regions that lack disorder annotation are ordered, while in fact some of these residues could be disordered.

To this end, we overview the past surveys and perform novel comparative assessment that accommodates for the above drawbacks. We analyze predictive quality for two key functional types of disordered proteins: protein-binding and nucleic acid-binding proteins [21,22,38,40], and compare these results to the typically performed test on a generic set of proteins. Moreover, we develop and utilize a new benchmark dataset that shares low level of similarity with the training datasets of the evaluated predictors and that relies solely on the experimentally verified annotations (i.e., it does not assume order annotations).

## 2. Surveys of the Intrinsic Disorder Predictors

We found a total of 28 surveys of the intrinsic disorder predictors that were published over the last 17 years [1,45,47,48,49,50,84,85,86,87,88,89,90,91,92,93,94,95,96,97,98,99,100,101,102,103,104,105]. These surveys typically provide a brief historical overview of the disorder prediction field and summarize and contrast a selected set of predictors. The latter often involves summarizing their designs, a discussion of their availability and features, a comparison of their predictive architectures, and, in some cases, a comparative assessment of their predictive performance.

**Figure 1:**
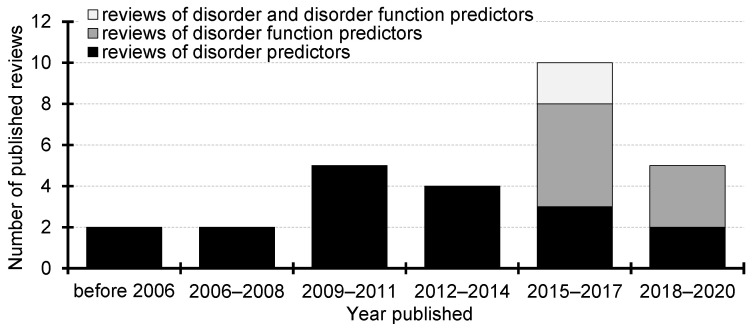
Chronological summary of the past surveys of the intrinsic disorder and intrinsic disorder function predictors.

To the best of our knowledge, the first survey was published in 2003 [90]. It summarizes results of the Critical Assessment of Structure Prediction (CASP5) experiment, the first CASP event that included disorder prediction. This article describes six disorder predictors and performs empirical comparative assessment of their predictive quality. Figure 1 provides a chronological summary of the 28 surveys. We divide them into three categories: those that discuss the predictors of intrinsic disorder, the predictors of disorder functions (specific functional types of disordered regions), and those that review both types of predictors. We reveal a recent shift from surveys of disorder predictors towards covering predictors of disorder functions. The functions of IDRs are classified based on the underlying cellular functions and the associated molecular partners [28,106,107]. This classification is utilized in DisProt, the largest database of the functionally annotated IDRs [41,108,109]. The main types of cellular functions include entropic chains, display sites, chaperons, effectors, assemblers and scavengers [106]. The six types of partners that interact with the IDRs are proteins, nucleic acids (DNA and RNAs), lipids, metals, inorganic salt and small molecules [41,108]. According to a recent analysis [94], the two most commonly annotated partners are proteins and nucleic acids. They collectively cover 84% of the partner-annotated IDRs in the DisProt resource. More precisely, 66% partner-annotated regions have protein partners, 17% nucleic acids partners, 6% metals, 5% lipids, 5% small molecules, and 1% has inorganic salt partners. This motivates the focus of this survey on the disordered proteins that interact with proteins and nucleic acids.

Further analysis shows that only 11 of the 28 surveys include an original empirical comparative assessment. We summarize them in Table 1. We quantify the impact of these studies by listing the number of citations in Google Scholar that the corresponding 11 articles have received. The majority of the articles (7 out of 11) were cited over 100 times each. The median and total numbers of citations are 114 and 1016, respectively. This shows substantial interest in this topic. Six of the 11 surveys concern the CASP experiments between 2002 (CASP5) and 2012 (CASP10) [85,86,87,88,89,90]. After CASP10, the assessment of disorder prediction was discontinued in CASP in favor of an effort led by the disorder prediction community, the Critical Assessment of Intrinsic protein Disorder (CAID) experiment (http://disprotcentral.org/caid). While the first round of the CAID experiment was completed in 2018, the official results are still pending. A preliminary CAID manuscript reveals that that this assessment is similar to the CASP experiment, with the main difference being the underlying benchmark dataset that is sourced from DisProt (disorder database) [110]. This is arguably a more appropriate type of dataset compared to the CASP datasets that were sourced from PDB (structure database).

Interestingly, different comparative surveys conclude that different predictors offer the best predictive accuracy. This is in part due to the fact that these assessments consider different collections of predictors [50]. Moreover, the best-performing methods are often published recently and were simply unavailable during the prior comparative studies. This is apparent when reading the ‘suggested best disorder predictor’ and ‘year the most recent assessed predictor published’ columns in Table 1. A case in point is the most recent comparative survey [93] that concludes that SPOT-Disorder [74] and DISOPRED3 [57] outperform the other current tools, while SPOT-Disorder was released after all but two of the 11 comparative surveys were published. Considering the three most-recent empirical assessments [45,91,93], the best performing predictors include SPOT-Disorder [74], DISOPRED3 [57], ESpritz [60], DisEMBL [53] and IUPred [62,63,64]. Our comparative analysis includes these five methods.

Table 1 analyzes the target of the past assessments, i.e., whether the evaluation assesses the prediction of disorder for proteins that interact with specific partner types. Overall, 10 out of the 11 comparative surveys focus on the assessment of a generic set of the disordered proteins, with the exception of one study that focuses on the integral membrane proteins [92]. The latter article compares 13 disorder predictors on a dataset of about 350 membrane proteins [92]. The absence of the targeted studies can be explained by the lack of the experimentally annotated data that could be used to perform the tests on the functionally annotated IDRs. More specifically, the annotations of the binding partners were added only in 2016 in version 7 of the DisProt database. The original set of 1108 partner-annotated IDRs was further extended to 1476 in the recently released version 8 of DisProt.

The last column in Table 1 considers another desirable aspect of the comparative evaluation, namely the similarity of the proteins in the benchmark dataset to the proteins in the training datasets that were used to develop the considered disorder predictors. While the benchmark datasets used in the past comparative surveys use new protein datasets (compared to the training datasets), the degree of similarity between the benchmark and the training proteins was never explicitly limited. For instance, the CASP assessments rely on unreleased depositions into the PDB that were not screened against the training proteins [85,86,87,88,89,90]. Similarly, the datasets used in the recent comparative studies [45,91,93] were collected from the MobiDB, DisProt and UniProt resources without screening them against the training datasets. Similarly, the forthcoming CAID assessment relies on the benchmark proteins collected from DisProt that were not screened for similarity to the training proteins [110]. At the same time, the practice of screening the test datasets against similarity to the training proteins is common and expected when individual predictors are published and evaluated against (usually limited) set of competing methods. A typical approach that was used in the recent articles that introduce disorder predictors is to limit sequence similarity between the test proteins and the training proteins to below 30% [69,74,111,112,113]. This ensures a fair assessment across all considered tools, i.e., none of the methods has the ‘advantage’ of benefitting from using more similar training proteins. Moreover, this approach measures the predictive quality for a challenging set of proteins for which alignment-based predictions cannot provide accurate results.

In sum, the analysis of the past surveys reveals two major limitations. They are exclusively focused on the analysis of performance for a generic set of disordered proteins and they do not follow the common practice of limiting similarity between the benchmark and the training proteins. We address these issues in the comparative survey that we introduce next.

## 3. Setup for Comparative Analysis

### 3.1. Selection of Disorder Predictors

We cover 10 carefully selected, popular, publicly available and diverse disorder predictors. They include three versions of ESpritz that are tuned to predict intrinsic disorder annotated from X-ray structures (ESpritz-Xray), NMR structures (ESpritz-NMR) and using DisProt database (ESpritz-DisProt) [60]; two versions of IUPred that target prediction of short (IUPred-short) and long (IUPred-long) IDRs [62,64]; DisEMBL [53]; GlobPlot [61]; VSL2B [77], DISOPRED3 [57], and SPOT-Disorder [74]. These predictors are highly cited and, by extension, often used. Their citation numbers are 2109 (IUPred), 1263 (DisEMBL), 1035 (GlobPlot), 731 (VSL2B), 409 (DISOPRED3), 288 (ESpritz), and 127 (SPOT-Disorder); source: Google Scholar on September 29, 2020. This selection overlaps with the predictors that were covered in the recent comparative assessments [45,91,93] and includes five tools that have been highlighted as the best-performing in the last three comparative surveys (methods shown in bold font in Table 1). Moreover, the 10 selected tools uniformly represent the three major categories of disorder predictors [48,49,50,93,114]: the ab initio tools (IUPred-short, IUPred-long and GlobPlot); the machine-learning-based predictors (DisEMBL, VSL2B, and SPOT-Disorder); the meta-predictors (DISOPRED3, ESpritz-Xray, ESpritz-NMR and ESpritz-DisProt). Finally, in the spirit of CASP experiments, methods developed by the assessors (i.e., authors of this comparative survey) are excluded from this comparison.

### 3.2. Benchmark Dataset

Recent comprehensive survey concludes that an ‘updated and more comprehensive benchmark datasets should be established’ [50]. Correspondingly, we devised a new benchmark dataset that accommodates the three drawbacks of the comparative studies that we list in the introduction [45,84,85,86,87,88,89,90,91,92,93]. To overcome the first drawback, we quantify and comparatively analyze performance for the two key functional families of disordered proteins, the protein-binding and the nucleic-acid binding proteins. To address the second drawback, we explicitly reduce the similarity of the new benchmark dataset to the training sets of the selected 10 disorder predictors. Finally, we tackle the third drawback by using validated experimental annotations for both disordered and ordered regions.

We collected the new dataset in four steps. First, we obtained the complete set of 1418 proteins from that have experimental annotations of disorder and binding partners from the new version 8 of DisProt. We excluded the disorder annotations marked as “ambiguous”. Second, we collected the training datasets of the ten disorder predictors. We clustered the combined set of the DisProt and training proteins using CD-Hit [115] at 30% sequence similarity and we removed clusters that included at least one training protein. The remaining set of 319 DisProt proteins was dissimilar to the training proteins (at 30%) and included functional annotations that allowed us to identify the protein-binding proteins (that have at least one disordered protein-binding region) and the nucleic-acid-binding proteins (that include at least one disordered nucleic-acid-binding region). Third, we ensured that only experimentally validated annotations were used to test the predictive performance. We mapped the unannotated regions of the DisProt proteins into PDB to annotate ordered/structured residues. To do that, we first created a dataset of the PDB sequences that were structured by masking residues in these sequences that lacked structure. We aligned segments of the DisProt sequences that lack annotations into these masked PDB sequences with Basic Local Alignment Search Tool (BLAST) [116,117]. We annotated the DisProt segments that shared ≥90% similarity and had an e-value ≤0.1 with at least one masked PDB sequence as structured. Fourth, we balanced this dataset of proteins with IDRs, which included 38 fully disordered proteins (i.e., all amino acids are disordered), by adding 38 fully structured proteins. We collected structured proteins from PDB using criteria that minimize the chances that they include disorder, i.e., we selected monomers with high-quality crystal structures (resolution <2 Å) that covered complete UniProt sequences based on mapping with Structure Integration with Function, Taxonomy and Sequence (SIFTS) [118]. We clustered these sequences with the training proteins using CD-Hit at 30% similarity and we selected, at random, 38 sequences from the clusters that exclude the training proteins. Consequently, the resulting dataset has 357 proteins that shared <30% similarity to the training proteins and included disordered residues (using experimental annotation from DisProt that included protein- and nucleic-acid-binding regions), structured residues (using experimental annotations from DisProt and PDB) and residues that lack annotation. The latter residues were excluded from the assessment. We provide this novel benchmark dataset in the Supplement and we summarize it in Table 2.

### 3.3. Assessment of Predictive Performance

Disorder predictors generate two types of output for every residue in the input protein chain: a real-valued putative propensity for intrinsic disorder and a binary disorder prediction (disordered vs. ordered). The binary predictions are usually produced from the propensities such that the residues with high propensities that are above a predictor-specific threshold are classified as disordered, while the remaining residues are assumed to be ordered. We assessed the predictive quality for both types of output. We utilized AUC to measure the predictive quality of the putative propensities. This measure was used across all recent dataset-level assessments [45,50,84,85,91,92,93,102]. We supplemented the AUC values that could be insensitive to false positives and false negatives by several other widely used measures that quantified the predictive quality of the binary predictions [45,50,84,85,91,92,93,102]: precision = TP/(TP + FP), sensitivity = TP/(TP + FN), false positive rate (FPR) = FP/(FP + TN), and Matthews’s correlation coefficient (MCC) = (TP*TN + FP*FN)/square root((TP + FP)*(TP + FN)*(TN + FP)*(TN + FN)), where True Positives (TP) and True Negatives (TN) denote the number of the correctly predicted disordered and ordered residues, respectively, False Positives (FP) denotes the number of the ordered residues predicted as disordered and False Negatives (FN) denotes the number of the disordered residues predicted as ordered. The precision and sensitivity quantify the rates of correct predictions among the predicted disordered residues and among the native disordered residues, respectively. FPR measures the rate of disorder predictions among structured residues. MCC is a correlation between the native and the predicted binary annotations of disorder.

## 4. Comparative Assessments

### 4.1. Impact of Sequence Similarity and Experimental Validation of Annotations

**Figure 2:**
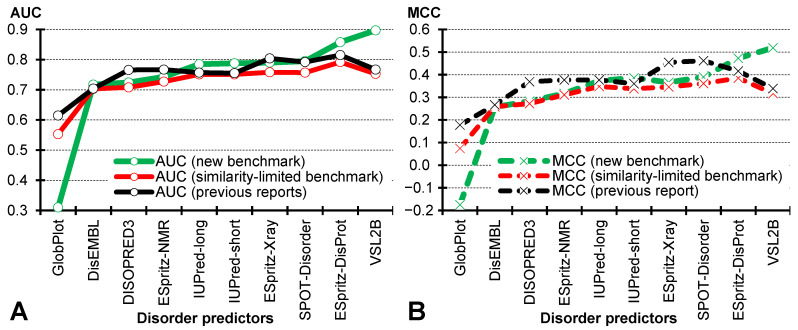
Comparison of the predictive quality measured with AUC (panel **A**; solid lines) and MCC (panel **B**; dashed lines). We report results on the *new benchmark* (in green; dataset with <30% sequence similarity to the training proteins + with experimental validation of structured regions + with fully structured proteins), based on recent *previous reports* (in black; datasets with no limits on sequence similarity to the training proteins + with no experimental validation of structured regions + with only disordered proteins), and based on a *similarity-limited benchmark* (in red; a version of the new benchmark dataset with <30% sequence similarity to the training proteins + no experimental validation of structured regions + only disordered proteins). The latter dataset is a proxy for the datasets used in prior studies, with the only difference being the reduced similarity to the training proteins. Disorder predictors are sorted by their AUC values on the new benchmark dataset.

Figure 2 compares results from recent comparative surveys against the results generated on the new benchmark dataset for the same set of 10 predictors. We consider three sets of results. The first is based on recently published comparative surveys (dubbed ‘previous results’ in Figure 2). These results were taken from [91], except for SPOT-Disorder’s results that were collected from [75]; this predictor was excluded from the other assessment. These evaluations rely on the benchmark proteins collected from the same main source as our new benchmark set, the DisProt database. The defining features of these datasets are that they do not impose limits on sequence similarity with the training proteins, do not experimentally validate the annotations of the structured regions, and include only proteins with IDRs. The second set of results is based on a test dataset that is equivalent to the past benchmarks, with the only difference being limiting the sequence similarity of the test proteins to <30% compared to the training proteins (dubbed “limited-similarity benchmark” in Figure 2). This dataset also does not validate the annotations of the structured regions and covers only proteins with IDRs. The third set of results relies on the new benchmark dataset (dubbed “new benchmark” in Figure 2). This dataset limits the similarity to the training proteins to <30%, uses experimentally validated structured regions and includes fully structured proteins.

Comparison of the ‘previous results’ (black markers in Figure 2) with the results for the ‘limited-similarity benchmark’ (red markers in Figure 2) allows us to directly quantify the impact of limiting the sequence similarity. These two sets of results are highly correlated, with the Pearson’s correlation coefficient of 0.94 for AUC and 0.89 for MCC. Moreover, we observe a consistent drop in performance when the similarity is reduced. On average, the results based on the benchmark that limits similarity are lower by 0.03 in AUC (0.72 vs. 0.75; Figure 2A), and by 0.06 in MCC (0.30 vs. 0.36; Figure 2B). The difference in AUC for the predictor that secures the highest AUC in the prior surveys, ESpritz-DisProt, is 0.05 (0.758 vs. 0.804). Similarly, the difference in MCC for the method that obtains the highest MCC in the prior surveys, SPOT-Disorder, is 0.10 (0.361 vs. 0.462). These results demonstrate that use of the benchmark datasets that allow for high sequence similarity with the training proteins leads to a substantial increase/over-estimation of the predictive quality.

Comparison of the ‘limited-similarity benchmark’ (red markers in Figure 2) with the results for the new benchmark (green markers in Figure 2) reveals that the use of higher quality annotations and fully structured proteins impacts the predictive performance. In most cases, we find that the predictive quality improves when the new benchmark dataset is used. This is expected, as we eliminate the residues with ambiguous/low-quality annotations from the assessment. We show the biggest improvement is seen for VSL2B, where AUC goes up by 0.14 and MCC by 0.20; we explain this improvement in Section 4.2. We also find that GlobPlot’s performance drops by a substantial margin, 0.24 in AUC and 0.25 in MCC. This tool was originally developed to detect disorder by differentiating between globular and non-globular domains/regions, and apparently it detects the validated structured regions are non-globular (GlobPlot’s proxy for disorder) while they are not disordered.

### 4.2. Comparative Assessment on the Benchmark Dataset

Table 3 compares predictive quality for the 10 representative predictors. The left side of the table summarizes the assessment of the complete set of 357 benchmark proteins. We find that VSL2B provides AUC = 0.90, which is significantly better than the results of the other nine predictors (*p*-value < 0.01). However, several other tools provide high-quality predictions, with AUC > 0.75 and MCC > 0.35, including ESpritz-DisProt, SPOT-Disorder, ESpritz-Xray and both versions of IUPred. Precision values reveal that over half of the disordered residues output by VSL2B and ESpritz-DisProt are predicted correctly. VSL2B and SPOT-Disorder secure the two best sensitivity values, which show that they correctly predict over 75% of the native disordered residues. Altogether, these results demonstrate that several current disorder predictors provide very accurate predictions.

The right side of Table 3 gives results on the 319 proteins from the benchmark dataset that exclude the fully ordered/structured proteins. We observe a few substantial differences between these results and the results on the complete benchmark set. First, the top performer is ESpritz-DisProt. It scores AUC = 0.85, which is significantly higher than AUCs of the other nine methods (*p*-value < 0.01). While sensitivity is the same because the set of native disordered residues is the same for both versions of the dataset, the precision goes substantially up for all methods but VSL2B. This is because they predict a substantial number of disordered residues in the fully structured proteins. The FPR scores in the ‘Fully disordered proteins’ column in Table 3 reveal that anywhere between 5% (for ESpritz-DisProt) and 100% (for GlobPlot) of residues in the fully structured proteins are predicted as disordered, thus lowering their precision on the full benchmark dataset. We note that the FPRs on the fully structured proteins are comparable to the FPRs on the remainder of the dataset that includes disordered proteins (i.e., proteins that have IDRs), see the second last column in Table 3. This means that these nine methods equally over-predict disorder in the fully structured proteins and in the structured regions in the disordered proteins. The only method that does not produce a substantial number of false positives on the fully structured proteins is VSL2B. Moreover, VSL2B also generates the best/highest sensitivity on the fully disordered proteins (‘Fully disordered proteins’ column in Table 3), which means that this predictor is the closest to recognizing that these are fully disordered proteins. These two results explain why VSL2B secures the top scores on the benchmark dataset. These findings also explain why VSL2B improves so much between the similarity-limited benchmark and the complete benchmark results in Figure 2.

We conclude that VSL2B is the most versatile method. The results show that this is the top choice to predict the fully structured and the fully disordered proteins, while also performing very well on the disordered proteins. Moreover, ESpritz-DisProt and SPOT-Disorder are the two best choices to make predictions on the disordered proteins, as their AUC and MCC scores outperform VSL2B and other predictors on these proteins.

### 4.3. Comparative Assessment for the Disordered Protein-Binding and Nucleic Acid-Binding Proteins

We provide the first assessment of the predictive performance for two major functional families of disordered proteins: protein-binding and nucleic acids-binding proteins. We compare these results with each other and against the results on all disordered proteins in the benchmark dataset. To do that, we first accommodate for the fact that predictive quality is sensitive to the native amount of the disorder content [45,74,76,93]. These studies show that the performance on proteins with a substantial amount of disorder is typically lower compared to the proteins with smaller amounts of disorder. Correspondingly, we sub-sample the benchmark dataset and the dataset of the disordered protein-binding proteins to match the distribution of the disorder content in the smallest dataset of the disordered nucleic-acid binding proteins (Table 2). More specifically, we quantify the significance of the differences in the protein-level disorder content distributions with the Kolmogorov–Smirnov test, and we progressively remove proteins that improve the *p*-value by the largest margin (to minimize the amount of sub-sampling) until we reach a *p*-value of 0.001. Supplementary Figure S1 compares the distributions of the AUC and MCC scores in the original datasets (grey plots) and after the disorder content distributions are equalized (white plots). These plots show that while the absolute levels of performance have shifted after sampling, the relative differences in the performance on the benchmark proteins and the protein-binding and nucleic acid-binding proteins are consistent before and after the sampling.

**Figure 3:**
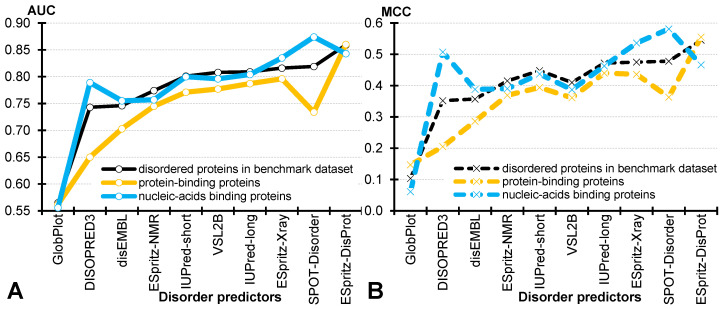
Comparison of the predictive quality measured with AUC (panel **A**; solid lines) and MCC (panel **B**; dashed lines). We report results on the generic set of disordered proteins (i.e., proteins that have disordered residues) from benchmark dataset (in black), the disordered protein-binding proteins (in orange), and the disordered nucleic-acid-binding proteins (in blue). Disorder predictors are sorted by their AUC values on the disordered proteins.

Supplementary Table S1 gives a complete set of results for the 10 representative predictors on the three disorder-content equalized datasets. Figure 3 summarizes values of the two typically reported metrics, AUC and MCC. We find that on average, across the 10 predictors, the predictive quality for the nucleic acids-binding proteins and the generic set of disordered proteins is similar, i.e., average AUC = 0.781 (nucleic acids-binding) vs. 0.774 (all disordered proteins in the benchmark dataset); average MCC = 0.422 vs. 0.406. However, the results for the disordered protein-binding proteins are substantially worse, with the average AUC = 0.739 and average MCC = 0.356. Figure 3 shows that the drop in the performance for these proteins is consistent across eight methods, with two exceptions of the ESpritz-DisProt, that maintains similarly strong levels of performance across the three protein sets, and GlobPlot, that secures consistently poor results. Moreover, while the best predictor for the disordered proteins and the disordered protein-binding proteins is ESpritz-DisProt (AUC = 0.86), the best AUC = 0.87 for the disordered nucleic-acid-binding proteins is secured by SPOT-Disorder (vs. AUC = 0.84 for ESpritz-DisProt). These results agree with a recent study that focuses on predictions for the structured proteins [119]. It similarly shows that the predictions of the protein-binding residues suffer much worse predictive performance when compared to the predictions of the DNA- and RNA-binding residues in the structured proteins.

We further analyze the underlying data to investigate potential reasons for this drop in predictive performance for the disordered protein-binding proteins. We compare the predictor-generated disorder propensities for the experimental disordered regions between the protein-binding proteins and the remainder of the benchmark dataset. We observe that the results are mixed, i.e., some methods predict higher scores for the protein-binding proteins (the three version of ESpritz, IUPred-long, VSL2B, DISOPRED3, and SPOT-Disorder) while others generate higher scores for the non-protein-binding proteins (DisEMBL, GlobPlot and IUPred-short). However, similar comparison of the disorder propensities that were generated for the experimental ordered regions reveals a consistent pattern, Namely, we find that the median disorder propensities of the 10 predictors are always significantly higher for the ordered regions in the disordered protein-binding proteins compared to the other disordered proteins; *p*-value ≤ 0.01 using the statistical test defined in Table 3. This suggests that disorder predictors struggle to identify structured regions in these proteins. This can be explained by the fact that the disordered protein-binding proteins are much more disordered (median protein-level disorder content = 0.62) compared to the non-protein-binding disordered proteins in our benchmark set (median protein-level disorder content = 0.32). This observation agrees with several recent studies which observe that predictions for the proteins with longer disordered regions and for proteins with higher disorder content are characterized by lower predictive quality [45,74,85,93].

## 5. Summary

Accurate predictors of intrinsic disorder are necessary to annotate the millions of the currently unannotated protein sequences. Over 60 disorder predictors have been published to date. Users who navigate the challenging task of predictor selection undoubtedly benefit from the availability of the 28 surveys that we discussed. However, we show that only 11 of these surveys include a comparative component that allows for a direct side-by-side analysis of predictive performance [45,84,85,86,87,88,89,90,91,92,93]. We present a novel comparative study that tests a representative set of 10 disorder predictors on a well-designed benchmark set to address the three shortcomings of the past comparative studies that we identify in the Introduction. The specific improvements over the past surveys that we cover include the use of the benchmark dataset with reduced similarity to the training datasets of the considered predictors, use of the experimentally validated ordered region and analysis that focuses on the two large functional families of disordered proteins that contain protein-binding and nucleic-acid-binding regions.

**Figure 4:**
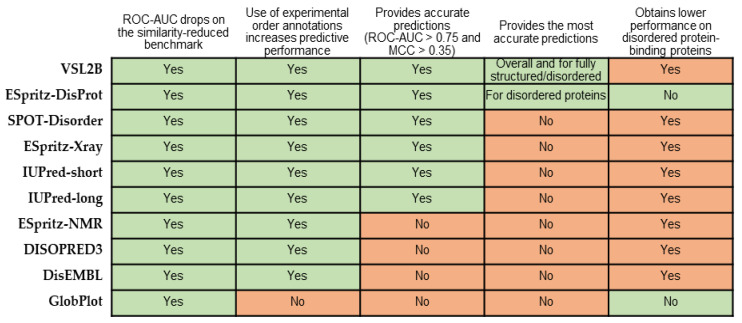
Summary of the empirical comparative results.

The results of our comparative survey are summarized in Figure 4. Our analysis reveals that limiting the sequence similarity between the benchmark dataset and the training datasets has a substantial impact on the predictive performance across the 10 considered predictors. The AUC and MCC values are lower on average by 0.03 and 0.06, respectively, when using the similarity-reduced benchmark set. This type of correction should be made when considering results from the recent studies [45,85,91,93,110]. Moreover, we analyze the impact of using experimental annotations of order and related inclusion of the fully structured proteins on the predictive performance. We demonstrate that predictive quality is sensitive to these issues as it consistently (across all predictors, except only for GlobPlot) improves with the use of the higher-quality order annotations. We postulate that future assessments should rely on such improved annotation protocols and should include fully structured proteins. Our results suggest that the majority of the predictors including VSL2B, SPOT-Disorder, ESpritz-DisProt, ESpritz-Xray and both versions of IUPred provide accurate predictions. VSL2B secures the overall best result and it also outperforms the other methods on the fully structured and the fully disordered proteins. However, the most accurate predictions for the disordered proteins (i.e., proteins that have disordered residues) are produced by ESpritz-DisProt. A particularly novel finding is that predictions for the disordered protein-binding proteins suffer low performance. The AUC and MCC values for these proteins are, on average, lower by 0.04 and 0.05 when compared to the set of generic disordered proteins, and by 0.04 and 0.07 when compared to the disordered nucleic-acid-binding proteins, respectively. We find that the underlying reason for this is the relatively poor ability of the current disorder predictors to identify ordered regions in the disordered protein-binding proteins, which, overall, have high amounts of disorder. The importance of this finding is motivated by the fact that disordered protein-binding proteins constitute a significant majority (66%) of the partner-annotated IDRs [94].

Lastly, we offer a few suggestions for users and developers. The issues with the lower quality of predictions can be improved on by designing a new class of predictors and by using tools that support the collection of high-quality disorder predictions. The result that highlights the difficulty of the current disorder predictors with the disordered protein-binding proteins motivates the development of a new generation of methods that specifically target this difficult-to-predict class of the disordered proteins. This aligns with the active research in the prediction of the protein-binding residues, which could offer useful design clues [120,121]. Furthermore, given the importance of AUC as the predictive performance metric, developers may consider explicitly optimizing their machine learning algorithms to maximize the AUC scores [122]. There are also several tools that can be used to collect and identify high-quality disorder predictions. A recently developed QUality Assessment for pRotein inTrinsic disordEr pRedictions (QUARTER) tool generates quality assessment scores for the residue-level predictions generated by several popular disorder predictors [123,124], including the well-performing (according to this analysis) ESpritz, VSL2B and IUPred. These scores inform users of whether a given residue is accurately predicted by a given method, and can be used to identify poorly predicted residues and proteins. Another viable option is to utilize consensus predictors, such as MobiDB-lite [70], that combine predictions from multiple disorder predictors. The consensus predictors, including MobiDB-lite, were shown to improve on the results offered by their inputs predictors [70,125]. One more alternative is to utilize the DISOselect platform that suggests the most accurate disorder predictor for a given input protein chain and estimates the predictive quality for this method [126]. The use of the latter tool is supported by the two databases of the disorder predictions, MobiDB [42] and D^2^P^2^ [13], which provide easy access to pre-computed predictions for close to a dozen disorder predictors. More specifically, users would first identify a well-performing predictor using DISOselect from among the methods covered by these databases, and then collect its predictions from a given database.

## Figures and Tables

**Table 1:** Summary of the past comparative assessments of disorder predictors. The articles are sorted chronologically (from the most recent). The citation numbers were collected from Google Scholar on 29 September 2020. Predictors shown in the bold font in the “suggested best disorder predictors” column are included in the comparative assessment in this article.

Article	Target of Assessment	Suggested Best Disorder Predictors (Year Published)	Year Assessment Published	Year Most Recent Assessed Predictor Published	Number of Citations	Benchmark Dataset has Reduced Similarity with Training Sets of the Assessed Predictors
This article	disordered proteins; disordered protein-binding protein; disordered nucleic acids-binding proteins	N/A	N/A	2018	N/A	yes
[93]	disordered proteins	**SPOT-Disorder** (2017), **DISOPRED3** (2015)	2019	2017	4	no
[91]	disordered proteins	**Espritz** (2012)	2018	2017	33	no
[45]	disordered proteins	**DisEMBL** (2003), **IUPred** (2005)	2015	2012	121	no
[85]	disordered proteins	**DISOPRED3** (2015), PrDOS (2007), MFDp (2010)	2014	2015	128	no
[92]	disordered integral membrane proteins	PreDisorder (2009)	2014	2012	12	no
[84]	disordered proteins	MFDp (2010), MD (2009), PONDR-FIT (2010)	2012	2010	149	no
[86]	disordered proteins	PrDOS (2007), DISOPRED (2004)	2011	2010	118	no
[87]	disordered proteins	GS-MetaServer (2012), PreDisorder (2009)	2009	2008	131	no
[88]	disordered proteins	DISOPRED (2004), DISpro (2005)	2007	2006	109	no
[89]	disordered proteins	predictor by Obradovic et al.	2005	2004	114	no
[90]	disordered proteins	N/A	2003	2002	97	no

**Table 2:** Summary of the Benchmark Dataset.

Dataset Characteristic	Complete Dataset	Protein-Binding Proteins	Nucleic Acids-Binding Proteins
Number of proteins	357	108	15
Number of residues	186,337	38,221	5934
Number of disordered residues	31,608	14,125	1567
Disorder content (% of disordered residues)	0.17	0.37	0.26

**Table 3:** Predictive performance on the new benchmark dataset. The table lists results on the complete benchmark dataset with 357 proteins, the set of 38 fully disordered proteins, the set of 38 fully structured proteins, and the benchmark dataset of 319 proteins that exclude the fully structured proteins. We quantify statistical significance of differences in AUC between the best predictor (identified in bold font) and each the other nine predictors on a given dataset. We bootstrap 50% of the proteins 100 times. For normal measurements (tested with the Anderson–Darling test at 0.05 significance) we use the paired *t*-test; otherwise we use the Wilcoxon rank sum test; = and + mean that the differences are not significant (*p*-value > 0.01) and significant (*p*-value ≤ 0.01), respectively.

Predictor	Benchmark Dataset					Fully Disordered Proteins	Fully Ordered Proteins	Benchmark Dataset without Fully Ordered Proteins				
	AUC	Precision	Sensitivity	FPR	MCC	Sensitivity	FPR	AUC	Precision	Sensitivity	FPR	MCC
VSL2B	**0.897**	**0.609**	**0.845**	**0.204**	**0.519**	**0.925**	**0.000**	0.805+	0.611	**0.845**	0.399	0.404
ESpritz-DisProt	0.858+	0.593	0.487	0.060	0.473	0.811	0.052	**0.842**	**0.685**	0.487	**0.067**	**0.486**
SPOT-Disorder	0.795+	0.334	0.756	0.261	0.390	0.662	0.290	0.826+	0.578	0.756	0.234	0.485
ESpritz-Xray	0.790+	0.375	0.623	0.193	0.366	0.702	0.226	0.812+	0.586	0.623	0.160	0.459
IUPred-short	0.788+	0.431	0.613	0.170	0.386	0.692	0.176	0.801+	0.607	0.613	0.165	0.444
IUPred-long	0.785+	0.422	0.693	0.233	0.373	0.834	0.262	0.806+	0.625	0.693	0.206	0.463
ESpritz-NMR	0.743+	0.336	0.721	0.310	0.317	0.774	0.351	0.776+	0.563	0.721	0.272	0.414
DISOPRED3	0.724+	0.294	0.653	0.293	0.283	0.662	0.340	0.767+	0.513	0.653	0.248	0.380
DisEMBL	0.717+	0.308	0.439	0.162	0.257	0.559	0.193	0.741+	0.520	0.439	0.132	0.336
GlobPlot	0.310+	0.122	0.428	0.655	−0.175	0.388	1.000	0.563+	0.332	0.428	0.326	0.096

## Supplementary Materials

The following are available online at https://www.mdpi.com/2218-273X/10/12/1636/s1. Figure S1: Distribution of the AUCs (panel A) and MCCs (panel B) over nine disorder predictors, Table S1: Predictive performance on the disorder-content equalized subset of the benchmark dataset, the disordered nucleic-acids binding proteins, and the disordered protein-binding proteins, Benchmark Dataset: Each protein is described using five lines that represent amino acid sequence, disorder annotation, disordered protein binding annotation, and disordered nucleic acid binding annotation.
