# Supplementary material for: Comparative Assessment of Intrinsic Disorder Predictions with a Focus on Protein and Nucleic Acid-Binding Proteins
Source: Biomolecules. 2020 Dec 4;10(12):1636. doi: 10.3390/biom10121636 (PMC7762010; doi:10.3390/biom10121636)
Supplement: Supplementary file 1 [file biomolecules-10-01636-s001.pdf]

Supplement for “Comparative assessment of intrinsic disorder predictions with a focus on protein and nucleic acid-binding proteins”

Akila Katuwawala[1] and Lukasz Kurgan[1]*
[1]Department of Computer Science, Virginia Commonwealth University, Richmond, Virginia, USA
*corresponding author: lkurgan@vcu.edu

Supplementary Tables

Supplementary Table S1. Predictive performance on the disorder-content equalized subset of the benchmark dataset, the disordered nucleic-acids binding proteins, and the disordered protein-binding proteins. We quantify statistical significance of differences in AUC between the best predictor (identified in bold font) and each the other nine predictors on a given dataset. We bootstrap 50% of the proteins 100 times. For normal measurements (tested with the Anderson-Darling test at 0.05 significance) we use paired t-test; otherwise we use the Wilcoxon rank sum test; = and + mean that the differences are not significant (*p*-value > 0.01) and significant (*p*-value ≤ 0.01), respectively.

Dataset	Predictor	AUC	Precision	Sensitivity	MCC
Disorder-content equalized benchmark dataset	**ESpritz-DisProt**	**0.859**	0.751	0.537	0.545
	SPOT-Disorder	0.819+	0.599	0.751	0.478
	ESpritz-Xray	0.816+	0.634	0.626	0.475
	IUPred-long	0.809+	0.669	0.695	0.472
	VSL2B	0.808+	0.671	0.848	0.410
	IUPred-short	0.801+	0.645	0.606	0.448
	ESpritz-NMR	0.774+	0.599	0.713	0.415
	disEMBL	0.746+	0.570	0.455	0.357
	DISOPRED3	0.743+	0.518	0.635	0.352
	GlobPlot	0.565+	0.364	0.427	0.103
Disorder-content equalized disordered protein-binding proteins	**ESpritz-DisProt**	**0.860**	0.816	0.552	0.555
	ESpritz-Xray	0.796+	0.684	0.648	0.436
	IUPred-long	0.787+	0.701	0.741	0.440
	VSL2B	0.777+	0.710	0.875	0.361
	IUPred-short	0.771+	0.672	0.617	0.394
	ESpritz-NMR	0.745+	0.639	0.738	0.369
	SPOT-Disorder	0.734+	0.587	0.807	0.363
	disEMBL	0.703+	0.610	0.423	0.286
	DISOPRED3	0.65+	0.522	0.668	0.205
	GlobPlot	0.562+	0.479	0.450	0.147
Disorder-content equalized disordered nucleic acids-binding proteins	**SPOT-Disorder**	**0.874**	0.752	0.814	0.581
	ESpritz-DisProt	0.843=	0.687	0.446	0.466
	ESpritz-Xray	0.835=	0.583	0.692	0.536
	IUPred-long	0.804=	0.571	0.685	0.463
	IUPred-short	0.800=	0.575	0.580	0.437
	VSL2B	0.796+	0.522	0.834	0.387
	DISOPRED3	0.789+	0.500	0.720	0.507
	ESpritz-NMR	0.757+	0.493	0.765	0.390
	disEMBL	0.755+	0.548	0.486	0.388
	GlobPlot	0.556+	0.298	0.407	0.061

Supplementary Figures

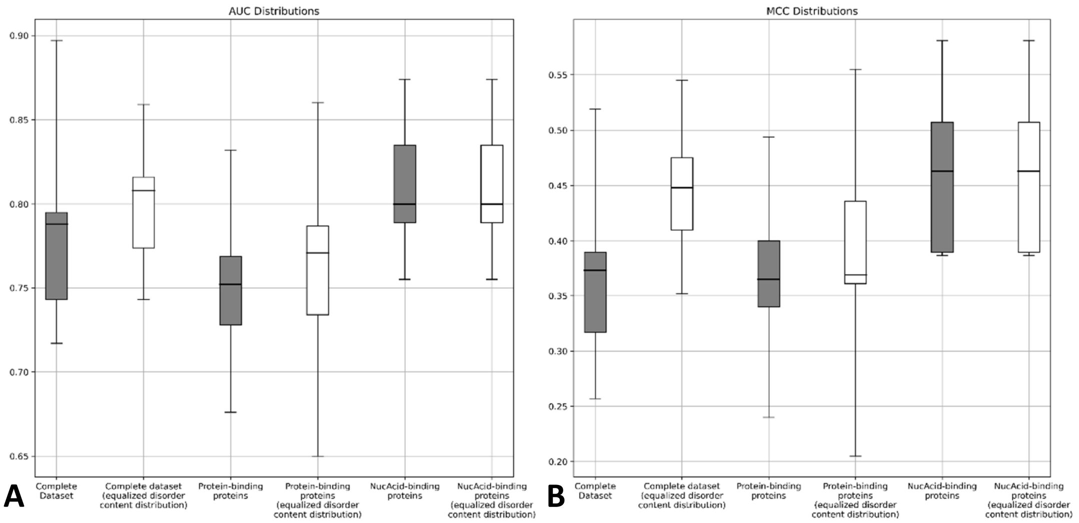

Supplementary Figure S1. Distribution of the AUCs (panel A) and MCCs (panel B) over nine disorder predictors. We exclude the poorly-performing GlobPlot from this analysis. The box plots show the lowest AUC (bottom error bar), first quartile (bottom of box), median (horizontal line inside box), third quartile (top of box) and highest AUC (top error bar). The grey plots are for the original datasets while the white plots are for the sampled/disorder content-equalized datasets that have similar distribution of the per-protein disorder content. The content distribution similarity was measured using Kolmogorov–Smirnov test at *p*-value of 0.001.

Benchmark Dataset

Each protein is described using five lines:

1. >Uniprot ID
2. AA sequence using 1-letter AA encoding
3. Disorder annotation: 1 for intrinsic disorder; 0 for structure/order; and X for missing annotation (excluded from assessment)
4. Disordered protein binding annotation: 1 for disordered protein-binding region; 0 for other disordered and ordered regions; and X for missing annotation
5. Disordered nucleic acids binding annotation: 1 for disordered nucleic acids-binding region; 0 for other disordered and ordered regions; and X for missing annotation

>P10163
MLLILLSVALLALSSAESSSEDVSQEESLFLISGKPEGRRPQGGNQPQRPPPPPGKPQGPPPQGGNQSQGPPPPPGKPEGRPPQGGNQSQGPPPHPGKPERPPPQGGNQSQGPPPHPGKPESRPPQGGHQSQGPPPTPGKPEGPPPQGGNQSQGTPPPPGKP
EGRPPQGGNQSQGPPPHPGKPERPPPQGGNQSHRPPPPPGKPERPPPQGGNQSQGPPPHPGKPEGPPPQEGNKSRSARSPPGKPQGPPQQEGNKPQGPPPPGKPQGPPPAGGNPQQPQAPPAGKPQGPPPPPQGGRPPRPAQGQQPPQ
XXXXXXXXXXXXXXXX1111111111111111111111111111111111111111111111111111111111111111111111111111111111111111111111111111111111111111111111111111111111111111111111111111
11111111111111111111111111111111111111111111111111111111111111111111111111111111111111111111111111111111111111111111111111111111111111
XXXXXXXXXXXXXXXX0000000000000000000000000000000000000000000000000000000000000000000000000000000000000000000000000000000000000000000000000000000000000000000000000000
00000000000000000000000000000000000000000000000000000000000000000000000000000000000000000000000000000000000000000000000000000000000000
XXXXXXXXXXXXXXXX0000000000000000000000000000000000000000000000000000000000000000000000000000000000000000000000000000000000000000000000000000000000000000000000000000
00000000000000000000000000000000000000000000000000000000000000000000000000000000000000000000000000000000000000000000000000000000000000
>P22531
MSYQQQQCKQPCQPPPVCPTPKCPEPCPPPKCPEPCPPPKCPQPCPPQQCQQKCPPVTPSPPCQPKCPPKSK
11111111111111111111XXXXXXXXXXXXXXXXXXXXXXXXXXX1111111111111111111111111
11111111111111111111XXXXXXXXXXXXXXXXXXXXXXXXXXX1111111111111111111111111
00000000000000000000XXXXXXXXXXXXXXXXXXXXXXXXXXX0000000000000000000000000
>O60927
MAEAGAGLSETVTETTVTVTTEPENRSLTIKLRKRKPEKKVEWTSDTVDNEHMGRRSSKCCCIYEKPRAFGESSTESDEEEEEGCGHTHCVRGHRKGRRRATLGPTPTTPPQPPDPSQPPPGPMQH
111111111111111111111111111111111111111111111111111111111111111111111111111111111111111111111111111111111111111111111111111111
111111111111111111111111111111111111111111111111111111111111111111111111111111111111111111111111111111111111111111111111111111
000000000000000000000000000000000000000000000000000000000000000000000000000000000000000000000000000000000000000000000000000000
>P10388
MAKRLVLFVAVVVALVALTVAEGEASEQLQCERELQELQERELKACQQVMDQQLRDISPECHPVVVSPVAGQYEQQIVVPPKGGSFYPGETTPPQQLQQRIFWGIPALLKRYYPSVTCPQQVSYYPGQASPQRPGQGQQPGQGQQGYYPTSPQQPGQWQQP
EQGQPRYYPTSPQQSGQLQQPAQGQQPGQGQQGQQPGQGQPGYYPTSSQLQPGQLQQPAQGQQGQQPGQAQQGQQPGQGQQPGQGQQGQQPGQGQQPGQGQQGQQLGQGQQGYYPTSLQQSGQGQPGYYPTSLQQLGQGQSGYYPTSPQQ
PGQGQQPGQLQQPAQGQQPGQGQQGQQPGQGQQGQQPGQGQQPGQGQPGYYPTSPQQSGQGQPGYYPTSSQQPTQSQQPGQGQQGQQVGQGQQAQQPGQGQQPGQGQPGYYPTSPQQSGQGQPGYYLTSPQQSGQGQQPGQLQQSAQG
QKGQQPGQGQQPGQGQQGQQPGQGQQGQQPGQGQPGYYPTSPQQSGQGQQPGQWQQPGQGQPGYYPTSPLQPGQGQPGYDPTSPQQPGQGQQPGQLQQPAQGQGQQLAQGQGQQPAQVQQGQRPAQGQGQQPGQGQQGQQLGQ
GQQGQQPGQGQQGQQPAQGQGQQPGQGQQGQQPGQGQQGQQPGQGQQPGQGQPWYYPTSPQESGQGQQPGQWQQPGQGQPGYYLTSPLQLGQGQQGYYPTSLQQPGQGQQPGQWQQSGQGQHWYYPTSPQLSGQGQRPGQWLQPG
QGQQGYYPTSPQQPGQGQQLGQWLQPGQGQQGYYPTSLQQTGQGQQSGQGQQGYYSSYHVSVEHQAASLKVAKAQQLAAQLPAMCRLEGGDALSASQ
XXXXXXXXXXXXXXXXXXXXXXXXXXXXXXXXXXXXXXXXXXXXXXXXXXXXXXXXXXXXXXXXXXXXXXXXXXXXXXXXXXXXXXXXXXXXXXXXXXXX1111111111111111111111111111111111111111111111111111111111111111111111111
11111111111111111111111111111111111111111111111111111111111111111111111111111111111111111111111111111111111111111111111111111111111111111111111111111111111111111111111111111111
1111111111111111111111111111111111111111111111111111111111111111111111111111111111111111111XXXXXXXXXXXXXXXXXXXXXXXXXXXXXXXXXXXXXXXXXXXXXXXXXXXXXXXXXXXXXXXXXXXXXXXXXXXXXXXXXXXXX
XXXXXXXXXXXXXXXXXXXXXXXXXXXXXXXXXXXXXXXXXXXXXXXXXXXXXXXXXXXXXXXXXXXXXXXXXXXXXXXXXXXXXXXXXXXXXXXXXXXXXXXXXXXXXXXXXXXXXXXXXXXXXXXXXXXXXXXXXXXXXXXXXXXXXXXXXXXXXXXXXXXXXXX
XXXXXXXXXXXXXXXXXXXXXXXXXXXXXXXXXXXXXXXXXXXXXXXXXXXXXXXXXXXXXXXXXXXXXXXXXXXXXXXXXXXXXXXXXXXXXXXXXXXXXXXXXXXXXXXXXXXXXXXXXXXXXXXXXXXXXXXXXXXXXXXXXXXXXXXX
XXXXXXXXXXXXXXXXXXXXXXXXXXXXXXXXXXXXXXXXXXXXXXXXXXXXXXXXXXXXXXXXXXXXXXXXXXXXXXXXXXXXXXXXXXXXXXXXXXXX0000000000000000000000000000000000000000000000000000000000000000000000000
00000000000000000000000000000000000000000000000000000000000000000000000000000000000000000000000000000000000000000000000000000000000000000000000000000000000000000000000000000000
0000000000000000000000000000000000000000000000000000000000000000000000000000000000000000000XXXXXXXXXXXXXXXXXXXXXXXXXXXXXXXXXXXXXXXXXXXXXXXXXXXXXXXXXXXXXXXXXXXXXXXXXXXXXXXXXXXXX
XXXXXXXXXXXXXXXXXXXXXXXXXXXXXXXXXXXXXXXXXXXXXXXXXXXXXXXXXXXXXXXXXXXXXXXXXXXXXXXXXXXXXXXXXXXXXXXXXXXXXXXXXXXXXXXXXXXXXXXXXXXXXXXXXXXXXXXXXXXXXXXXXXXXXXXXXXXXXXXXXXXXXXX
XXXXXXXXXXXXXXXXXXXXXXXXXXXXXXXXXXXXXXXXXXXXXXXXXXXXXXXXXXXXXXXXXXXXXXXXXXXXXXXXXXXXXXXXXXXXXXXXXXXXXXXXXXXXXXXXXXXXXXXXXXXXXXXXXXXXXXXXXXXXXXXXXXXXXXXX
XXXXXXXXXXXXXXXXXXXXXXXXXXXXXXXXXXXXXXXXXXXXXXXXXXXXXXXXXXXXXXXXXXXXXXXXXXXXXXXXXXXXXXXXXXXXXXXXXXXX0000000000000000000000000000000000000000000000000000000000000000000000000
00000000000000000000000000000000000000000000000000000000000000000000000000000000000000000000000000000000000000000000000000000000000000000000000000000000000000000000000000000000
0000000000000000000000000000000000000000000000000000000000000000000000000000000000000000000XXXXXXXXXXXXXXXXXXXXXXXXXXXXXXXXXXXXXXXXXXXXXXXXXXXXXXXXXXXXXXXXXXXXXXXXXXXXXXXXXXXXX
XXXXXXXXXXXXXXXXXXXXXXXXXXXXXXXXXXXXXXXXXXXXXXXXXXXXXXXXXXXXXXXXXXXXXXXXXXXXXXXXXXXXXXXXXXXXXXXXXXXXXXXXXXXXXXXXXXXXXXXXXXXXXXXXXXXXXXXXXXXXXXXXXXXXXXXXXXXXXXXXXXXXXXX
XXXXXXXXXXXXXXXXXXXXXXXXXXXXXXXXXXXXXXXXXXXXXXXXXXXXXXXXXXXXXXXXXXXXXXXXXXXXXXXXXXXXXXXXXXXXXXXXXXXXXXXXXXXXXXXXXXXXXXXXXXXXXXXXXXXXXXXXXXXXXXXXXXXXXXXX
>Q9ES89
MMRGCHICKLPGRVMGIRVLRFSLVVILVLLLVAGALTNLLPNIKEDKMLTLRREIKSPSKSALDSFTLIMQTYNRTDLLLRLLNHYQAVPSLHKVIVVWNNVGEKGPEELWNSLGPHPIPVIFKPQTANKMRNRLQVFPEVETNAVLMVDDDTLISAQDLVFAFSIW
QQFPDQIIGFVPRKHVSTSSGIYSYGGFELQTPGPGNGDQYSMVLIGASFFNSKYLELFQKQPAAVHALIDETQNCDDIAMNFLVTRHTGKPSGIFVKPINMVNLEKETNGYSGMWHRAEHFLQRSYCINKLVNIYDGMPLKYSNIMISQFGFPYANHKSKM
00000000000000000000000000000000000000000000000000000000000000000000000000000000000000000000000000000000000000000000000000000000000000000000000000000000000000000000000000000000
0000000000000000000000000000000000000000000000000000000000000000000000111111111111000000000000000000000000000000000000000000000000000000000000000000000000
00000000000000000000000000000000000000000000000000000000000000000000000000000000000000000000000000000000000000000000000000000000000000000000000000000000000000000000000000000000
0000000000000000000000000000000000000000000000000000000000000000000000111111111111000000000000000000000000000000000000000000000000000000000000000000000000
00000000000000000000000000000000000000000000000000000000000000000000000000000000000000000000000000000000000000000000000000000000000000000000000000000000000000000000000000000000
0000000000000000000000000000000000000000000000000000000000000000000000000000000000000000000000000000000000000000000000000000000000000000000000000000000000
>Q57696
MIEKLAEIRKKIDEIDNKILKLIAERNSLAKDVAEIKNQLGIPINDPEREKYIYDRIRKLCKEHNVDENIGIKIFQILIEHNKALQKQYLEETQNKNKK
XXXXXXXXXXXXXXXXXXXXX111111110000000000000000000000000000000000000000000000000000000000000000000000
XXXXXXXXXXXXXXXXXXXXXX00000000000000000000000000000000000000000000000000000000000000000000000000000
XXXXXXXXXXXXXXXXXXXXXX00000000000000000000000000000000000000000000000000000000000000000000000000000
>Q9UBT2
MALSRGLPRELAEAVAGGRVLVVGAGGIGCELLKNLVLTGFSHIDLIDLDTIDVSNLNRQFLFQKKHVGRSKAQVAKESVLQFYPKANIVAYHDSIMNPDYNVEFFRQFILVMNALDNRAARNHVNRMCLAADVPLIESGTAGYLGQVTTIKKGVTECYECHPKPTQ
RTFPGCTIRNTPSEPIHCIVWAKYLFNQLFGEEDADQEVSPDRADPEAAWEPTEAEARARASNEDGDIKRISTKEWAKSTGYDPVKLFTKLFKDDIRYLLTMDKLWRKRKPPVPLDWAEVQSQGEETNASDQQNEPQLGLKDQQVLDVKSYARLFSKSIETLRVHLA
EKGDGAELIWDKDDPSAMDFVTSAANLRMHIFSMNMKSRFDIKSMAGNIIPAIATTNAVIAGLIVLEGLKILSGKIDQCRTIFLNKQPNPRKKLLVPCALDPPNPNCYVCASKPEVTVRLNVHKVTVLTLQDKIVKEKFAMVAPDVQIEDGKGTILISSEEGETEANNH
KKLSEFGIRNGSRLQADDFLQDYTLLINILHSEDLGKDVEFEVVGDAPEKVGPKQAEDAAKSITNGSDDGAQPSTSTAQEQDDVLIVDSDEEDSSNNADVSEEERSRKRKLDEKENLSAKRSRIEQKEELDDVIALD
00000000000000000000000000000000000000000000000000000000000000000000000000000000000000000000000000000000000000000000000000000000000000000000000000000000000000000000000000000000
00000000000000000000000000000000000000000011111111111111111100000000000000000000000000000000000000000000000000000000000000111111111111110000000000000000000000000000000000000000
00000000000000000000000000000000000000000000000000000000000000000000000000000000000000000000000000000000000000000000000000000000000000000000000000000000000000000000000000000000
0000000000000000000001111111111111111111111111111111111111111111111111111111111111111111111111111111111111111111
00000000000000000000000000000000000000000000000000000000000000000000000000000000000000000000000000000000000000000000000000000000000000000000000000000000000000000000000000000000
00000000000000000000000000000000000000000000000000000000000000000000000000000000000000000000000000000000000000000000000000000000000000000000000000000000000000000000000000000000
00000000000000000000000000000000000000000000000000000000000000000000000000000000000000000000000000000000000000000000000000000000000000000000000000000000000000000000000000000000
0000000000000000000000000000000000000000000000000000000000000000000000000000000000000000000000000000000000000000
00000000000000000000000000000000000000000000000000000000000000000000000000000000000000000000000000000000000000000000000000000000000000000000000000000000000000000000000000000000
00000000000000000000000000000000000000000000000000000000000000000000000000000000000000000000000000000000000000000000000000000000000000000000000000000000000000000000000000000000
00000000000000000000000000000000000000000000000000000000000000000000000000000000000000000000000000000000000000000000000000000000000000000000000000000000000000000000000000000000
0000000000000000000000000000000000000000000000000000000000000000000000000000000000000000000000000000000000000000
>Q01417
MQGGKKAGESIKETATNIGASAKAGMEKTKATVQEKAERMTARDPVQKELATQKKEAKMNQAELDKQAARQHNTAAKQSATTAGHMGHGHHTTGTGTGTATYSTTGEYGQPMGAHQTSAMPGHGTGQPTGHVTEGVVGSHPIGTNRGPGGTATAHNT
RAGGKPNDYGYGTGGT
11111111111111111111111111111111111111111111111111111111111111111111111111111111111111111111111111111111111111111111111111111111111111111111111111111111111111111111111111111
00000000000000000000000000000000000000000000000000000000000000000000000000000000000000000000000000000000000000000000000000000000000000000000000000000000000000000000000000000
00000000000000000000000000000000000000000000000000000000000000000000000000000000000000000000000000000000000000000000000000000000000000000000000000000000000000000000000000000
>Q38151
MIEKDVVQILKAVSEFYPGRFQPDDLKGTVKAWHRVLAEYELEEIMNNLTDYAKVNKFPPTVSDLLKAQSEQRDRFIPSYEETQRILKEQAEAEEAARNDPDLQAAQEENMRKIREMLGINRGGAR
000000000000000000000000000000000000000000000000000000000000000000001111111111111111111111111111111111111111111111111111111111
000000000000000000000000000000000000000000000000000000000000000000000000000000000000000000000000000000000000000000000000000000
000000000000000000000000000000000000000000000000000000000000000000000000000000000000000000000000000000000000000000000000000000
>E6PBU9
MQVQVKTLEGETKIYTLEQGTSVLDLKSQISQDMGFEIDMMTLVNNGFIAPNTELVTDDVTYYLSLKLLGGKKKKKKKSYTTKKKTKHRHVHTKLGALAFYKLENNGKVSLQQKGCPKCGPGIFMAKHYDRHYCGKCHLTLKIDAETAKKNLEELKKKQDAKKSGAA
ATDAGAGGAGKKDAKKGKKGKK
11111111111111111111111111111111111111111111111111111111111111111111111XXXXXXXXXXXXXXXXXXXXXXXXXXXXXXXXXXXXXXXXXXXXXXXXXXXXXXXXXXXXXXXXXXXXXXXXXXXXXXXXXXXXXXXXXXXXXXXXXXXXXXX
XXXXXXXXXXXXXXX
00000000000000000000000000000000000000000000000000000000000000000000000XXXXXXXXXXXXXXXXXXXXXXXXXXXXXXXXXXXXXXXXXXXXXXXXXXXXXXXXXXXXXXXXXXXXXXXXXXXXXXXXXXXXXXXXXXXXXXXXXXXXXXXX
XXXXXXXXXXXXXXX
11111111111111111111111111111111111111111111111111111111111111111111111XXXXXXXXXXXXXXXXXXXXXXXXXXXXXXXXXXXXXXXXXXXXXXXXXXXXXXXXXXXXXXXXXXXXXXXXXXXXXXXXXXXXXXXXXXXXXXXXXXXXXXXX
XXXXXXXXXXXXXXX
>Q9BSY4
MQAALEVTARYCGRELEQYGQCVAAKPESWQRDCHYLKMSIAQCTSSHPIIRQIRQACAQPFEAFEECLRQNEAAVGNCAEHMRRFLQCAEQVQPPRSPATVEAQPLPAS
XXXXXXXXXXXXXXXXXXXXXXXXXXXXXXXXXXXXXXXXXXXXXXXXXXXXXXXXXXXXXXXXXXXXXXXXXXXXXXXXXXXXXXXXXXXXX1111111111111111X
XXXXXXXXXXXXXXXXXXXXXXXXXXXXXXXXXXXXXXXXXXXXXXXXXXXXXXXXXXXXXXXXXXXXXXXXXXXXXXXXXXXXXXXXXXXXX0000000000000000X
XXXXXXXXXXXXXXXXXXXXXXXXXXXXXXXXXXXXXXXXXXXXXXXXXXXXXXXXXXXXXXXXXXXXXXXXXXXXXXXXXXXXXXXXXXXXX0000000000000000X
>E6PBU3
MGKQPAAGQKKTKEAIAKAAQAKKGGKKKWTKGKAKDKVNHAVFIEKKNVESIINNPSKVGKVLTVSTVVEKLKVNGSLARQLMRTMADRKLVEKVAKNGNQWVYSVIGGVKEDKTAAPAAGGKKQQQSKKGAAADKEEVQA
111111111111111111111XXXXXXXXXXXXXXXXXXXXXXXXXXXXXXXXXXXXXXXXXXXXXXXXXXXXXXXXXXXXXXXXXXXXXXXXXXXXXXXXXXXXXXXXX1111111111111111111111111111
000000000000000000000XXXXXXXXXXXXXXXXXXXXXXXXXXXXXXXXXXXXXXXXXXXXXXXXXXXXXXXXXXXXXXXXXXXXXXXXXXXXXXXXXXXXXXXXXX0000000000000000000000000000
000000000000000000000XXXXXXXXXXXXXXXXXXXXXXXXXXXXXXXXXXXXXXXXXXXXXXXXXXXXXXXXXXXXXXXXXXXXXXXXXXXXXXXXXXXXXXXXXX0000000000000000000000000000
>O00585
MAQSLALSLLILVLAFGIPRTQGSDGGAQDCCLKYSQRKIPAKVVRSYRKQEPSLGCSIPAILFLPRKRSQAELCADPKELWVQQLMQHLDKTPSPQKPAQGCRKDRGASKTGKKGKGSKGCKRTERSQTPKGP

1111110000000000000000000000000000000000000000000000000000000000000011111111111111111111111111111111111111111XXXXXXXXXXXXXXXXXXXXXXX
000000000000000000000000000000000000000000000000000000000000000000000000000000000000000000000000000000000000000XXXXXXXXXXXXXXXXXXXXXXX
000000000000000000000000000000000000000000000000000000000000000000000000000000000000000000000000000000000000000XXXXXXXXXXXXXXXXXXXXXXX
>Q24F70
MVHVLKATKIRIYKQLLQDGVFVLKKDFEGHHEETGVPNLHCYILVRSLKDRGFLEEIFNWGFTYYYLNKEGCEYLKTKLGISADNVIPKTFKASNVNFISKEEDEEERPRRQFNKGGRTGERDGRNKRGVGRGTRREGEEAAKEEGAAETAQGNQETPAQE
0000000000000000000000000000000000000000000000000000000000000000000000000000000000000000000000000000000011111111111111111111111111111111111111111111111111111111111111111
00000000000000000000000000000000000000000000000000000000000000000000000000000000000000000000000000000000000000000000000000000000000000000000000000000000000000000
00000000000000000000000000000000000000000000000000000000000000000000000000000000000000000000000000000000000000000000000000000000000000000000000000000000000000000
>P46670
MDFTAKIKELERELSETSDYKTLQKKTISLRSELNTLSHSLTSYEKEHFSNDIENVLKSINAKLSESKGKKRLFSFKQKNSSSAVHKNVERTELANAPAYTTTLKKHYVLEKGDSAFENLEFCTVTSTTDYSGNSALSGSLCFRNITKCVINLQRIFFQTGSIFITDCTDSIIFLRSP
SDKDFQIRLRDLKNCKILIEKLSPSIDCKQVVIIENCHKCIFNASTRDHLIIQDFSNPFQSEETEDNSAFAFEDFDICNKDTMQLFRAYL
11111111111111111111111111111111111111000000000000000000000000000000000000000000000000000000000000000000000000000000000000000000000000000000000000000000000000000000000000000000
0000000000000000000000000000000000000000000111111111111111111111111100000000000000000000000
11111111111111111111111111111111111111000000000000000000000000000000000000000000000000000000000000000000000000000000000000000000000000000000000000000000000000000000000000000000
0000000000000000000000000000000000000000000111111111111111111111111100000000000000000000000
00000000000000000000000000000000000000000000000000000000000000000000000000000000000000000000000000000000000000000000000000000000000000000000000000000000000000000000000000000000
00000000000000000000000000000000000000000000000000000000000000000000000000000000000000000000
>P38801
MEDIEKIKPYVRSFSKALDELKPEIEKLTSKSLDEQLLLLSDERAKLELINRYAYVLSSLMFANMKVLGVKDMSPILGELKRVKSYMDKAKQYDNRITKSNEKSQAEQEKAKNIISNVLDGNKNQFEPSISRSNFQGKHTKFENDELAESTTTKIIDSTDHIRKASSKKSKRL
DKVGKKKGGKK
00000000000000000000000000000000000000000000000000000000000000000000000000000000000000000000000000000000000000000000011111111111111111111111111111111111111111111111111111111111
11111111
00000000000000000000000000000000000000000000000000000000000000000000000000000000000000000000000000000000000000000000000000000000000000000000000000000000000000000000000000000000
00000000
00000000000000000000000000000000000000000000000000000000000000000000000000000000000000000000000000000000000000000000000000000000000000000000000000000000000000000000000000000000
00000000
>P08245
MSDSNSRLVYSTETGRIDEPKAAPVRPKGDGVVRIQRQTSGRKGKGVCLITGVDLDDAELTKLAAELKKKCGCGGAVKDGVIEIQGDKRDLLKSLLEAKGMKVKLAGG
111111111111111111111111111111100000000000000000000000000000000000000000000000000000000000000000000000000000
000000000000000000000000000000000000000000000000000000000000000000000000000000000000000000000000000000000000
000000000000000000000000000000000000000000000000000000000000000000000000000000000000000000000000000000000000
>P9WHJ5
MADRVLRGSRLGAVSYETDRNHDLAPRQIARYRTDNGEEFEVPFADDAEIPGTWLCRNGMEGTLIEGDLPEPKKVKPPRTHWDMLLERRSIEELEELLKERLELIRSRRRG
11111111111111111111111111XXXXXXXXXXXXXXXXXXXXXXXXXXXXXXXXXXXXXXXXXXX111111111110000000000000000000000000000000
11111111111111111111111110XXXXXXXXXXXXXXXXXXXXXXXXXXXXXXXXXXXXXXXXXXX000000000000000000000000000000000000000000
11111111111111111111111111XXXXXXXXXXXXXXXXXXXXXXXXXXXXXXXXXXXXXXXXXXX000000000000000000000000000000000000000000
>P36886
MASQLSAMTSVPQFHGLRTYSSPRSMATLPSLRRRRSQGIRCDYIGSSTNLIMVTTTTLMLFAGRFGLAPSANRKATAGLKLEARESGLQTGDPAGFTLADTLACGAVGHIMGVGIVLGLKNTGVLDQIIG
11111111111111111111111111111111111111111110000000000000000000000000000000000000000000000000000000000000000000000000000000000000011111
00000000000000000000000000000000000000000000000000000000000000000000000000000000000000000000000000000000000000000000000000000000000
00000000000000000000000000000000000000000000000000000000000000000000000000000000000000000000000000000000000000000000000000000000000
>O15273
MATSELSCEVSEENCERREAFWAEWKDLTLSTRPEEGCSLHEEDTQRHETYHQQGQCQVLVQRSPWLMMRMGILGRGLQEYQLPYQRVLPLPIFTPAKMGATKEEREDTPIQLQELLALETALGGQCVDRQEVAEITKQLPPVVPVSKPGALRRSLSRSMSQEA
QRG
00000000000000000000000000000000000000000000000000000000000000000000000000000000000000000011111111111111111111111111111111111111111111111111111111111111111111111111111
00000000000000000000000000000000000000000000000000000000000000000000000000000000000000000011111111111111111111111111111111111111111111111111111111111111111111111111111
00000000000000000000000000000000000000000000000000000000000000000000000000000000000000000000000000000000000000000000000000000000000000000000000000000000000000000000000
>P9WNK3
MSQIMYNYPAMLGHAGDMAGYAGTLQSLGAEIAVEQAALQSAWQGDTGIYQAWQAQWNQAMEDLVRAYHAMSSTHEANTMAMMARDTAEAAKWGG
11111111111111110000000000000000000000000000000000000000000000000000000000011111111111111111111
00000000000000000000000000000000000000000000000000000000000000000000000000000000000000000000000
00000000000000000000000000000000000000000000000000000000000000000000000000000000000000000000000
>P22179
MASLATLAAVQPTTLKGLAGSSIAGTKLHIKPARQSFKLNNVRSGAIVAKYGDKSVYFDLEDIANTTGQWDVYGSDAPSPYNSLQSKFFETFAAPFTKRGLLLKFLILGGGSLLTYVSANAPQDVLPITRGPQQPPKLGPRGKI
111111111111111111111111111111111111111111111111111111111000000000000000000000000000000000000000000000000000000000000000000000000000000000000000
111111111111111111111111111111111111111111111111111111111000000000000000000000000000000000000000000000000000000000000000000000000000000000000000
000000000000000000000000000000000000000000000000000000000000000000000000000000000000000000000000000000000000000000000000000000000000000000000000
>A1B8N7
MTVANSASISADIAGRYAQALFDLVRDSGGIDALSSQIDDLASAYDASQDLRDLTLSPLYDRQQQEAAVGALSERMGLSAELANTLRLLARNRRLFTLPQFVAKLRNLIADAKGEVTADVVSAQALTDEQKARLADTLAAKSGKTVKLNARVDESLIGGMIVKLGSQ
MIDSSIRSKLASLQNAMKEVG
00000000000000000000000000000000000000000000000000000000000000000000000000000000000000000000000000000000000000000001111111111111111111111111111111111111111111111111111111111111
111111111111
00000000000000000000000000000000000000000000000000000000000000000000000000000000000000000000000000000000000000000000000000000000000000000000000000000000000000000000000000000000
000000000000
00000000000000000000000000000000000000000000000000000000000000000000000000000000000000000000000000000000000000000000000000000000000000000000000000000000000000000000000000000000
000000000000
>P24937
MEDINFASLAPRHGSRPFMGNWQDIGTSNMSGGAFSWGSLWSGIKNFGSTVKNYGSKAWNSSTGQMLRDKLKEQNFQQKVVDGLASGISGVVDLANQAVQNKINSKLDPRPPVEEPPPAVETVSPEGRGEKRPRPDREETLVTQIDEPPSYEEALKQGLPTTR
PIAPMATGVLGQHTPVTLDLPPPADTQQKPVLPGPTAVVVTRPSRASLRRAASGPRSLRPVASGNWQSTLNSIVGLGVQSLKRRRCF
111111111111111111111111111111111111111111111111111XXXXXXXXXXXXXXXXXXXXXXXXXXXXXXXXXXXXXXXXXXXXXXXXXXXXXXXXXXXXXXXXXXXXXXXXXXXXXXXXXXXXXXXXXXXXXXXXXXXXXXXX1111111111111111
11111111111111111111111111111111111111111111111111111111111111111111111111111
111111111111111111111111111111111111111111111111111XXXXXXXXXXXXXXXXXXXXXXXXXXXXXXXXXXXXXXXXXXXXXXXXXXXXXXXXXXXXXXXXXXXXXXXXXXXXXXXXXXXXXXXXXXXXXXXXXXXXXXXX1111111111111111
11111111111111111111111111111111111111111111111111111111111111111111111111111
000000000000000000000000000000000000000000000000000XXXXXXXXXXXXXXXXXXXXXXXXXXXXXXXXXXXXXXXXXXXXXXXXXXXXXXXXXXXXXXXXXXXXXXXXXXXXXXXXXXXXXXXXXXXXXXXXXXXXXXXX0000000000000000
00000000000000000000000000000000000000000000000000000000000000000000000000000
>P80870
MAAKFEVGSVYTGKVTGLQAYGAFVALDEETQGLVHISEVTHGFVKDINEHLSVGDEVQVKVLAVDEEKGKISLSIRATQAAPEKKESKPRKPKAAQVSEEASTPQGFNTLKDKLEEWIEMSNRKDLIKK
0000000000000000000000000000000000000000000000000000000000000000000000000000000011111111111111111111111111111111111111111111111111
0000000000000000000000000000000000000000000000000000000000000000000000000000000000000000000000000000000000000000000000000000000000
0000000000000000000000000000000000000000000000000000000000000000000000000000000000000000000000000000000000000000000000000000000000
>Q9V452
MGEPRSQPPVERPPTAETFLPLSRVRTIMKSSMDTGLITNEVLFLMTKCTELFVRHLAGAAYTEEFGQRPGEALKYEHLSQVVNKNKNLEFLLQIVPQKIRVHQFQEMLRLNRSAGSDDDDDDDDDDDEEESESESESDE
11111111111111111111111111100000000000000000000000000000000000000000000000000000000000000000000000000000111111111111111111111111111111111111111
11111111111111111111111111100000000000000000000000000000000000000000000000000000000000000000000000000000111111111111111111111111111111111111111
11111111111111111111111111100000000000000000000000000000000000000000000000000000000000000000000000000000111111111111111111111111111111111111111
>Q97QZ2
MIGKNIKSLRKTHDLTQLEFARIVGISRNSLSRYENGTSSVSTELIDIICQKFNVSYVDIVGEDKMLNPVEDYELTLKIEIVKERGANLLSRLYRYQDSQGISIDDESNPWILMSDDLSDLIHTNIYLVETFDEIERYSGYLDGIERMLEISEKRMVA
11111111111111111111111111111111111111111111111111111111111111110000000000000000000000000000000000000000000000000000000000000000000000000000000000000000000000
11111111111111111111111111111111111111111111111111111111111111110000000000000000000000000000000000000000000000000000000000000000000000000000000000000000000000
11111111111111111111111111111111111111111111111111111111111111110000000000000000000000000000000000000000000000000000000000000000000000000000000000000000000000
>O73557
MGNKQAKAPESKDSPRASLIPDATHLGPQFCKSCWFENKGLVECNNHYLCLNCLTLLLSVSNRCPICKMPLPTKLRPSAAPTAPPTGAADSIRPPPYSP
111111111111111111111111111110000000000000000000000000000000000000000000111111111111111111111111111
111111111111111111111111000000000000000000000000000000000000000000000000000001111111111111111111111
000000000000000000000000000000000000000000000000000000000000000000000000000000000000000000000000000
>Q9NJS1
MANKLVLISLLCVVLVAKITQAAPQYAPGDEPSYDEDTDDSDKLVENDTSITDEDYAAIEASLSETFNTAADPGRRLGEGSKP
XXXXXXXXXXXXXXXXXXXXXX1111111111111111111111111111111111111111111111111111111111111
XXXXXXXXXXXXXXXXXXXXXX0000000000000000000000000000001111111111111111111111111111111
XXXXXXXXXXXXXXXXXXXXXX0000000000000000000000000000000000000000000000000000000000000
>O31467
MFSNIGIPGLILIFVIALIIFGPSKLPEIGRAAGRTLLEFKSATKSLVSGDEKEEKSAELTAVKQDKNAG
XXXXXXXXXXXXXXXXXXXXXXXXXXXXXXXXXXXXXXXXX11111111111111111111111111111
XXXXXXXXXXXXXXXXXXXXXXXXXXXXXXXXXXXXXXXXX00000000000000000000000000000
XXXXXXXXXXXXXXXXXXXXXXXXXXXXXXXXXXXXXXXXX00000000000000000000000000000
>P13183
MFPLAKNALSRLRVQSIQQAVARQIHQKRAPDFHDKYGNAVLASGATFCVAVWVYMATQIGIEWNPSPVGRVTPKEWREQ
XXXXXXXXXXXXXXXXXXXXXXXXXXXXXXXXXXX111111111111111111111000000000000000000000000
XXXXXXXXXXXXXXXXXXXXXXXXXXXXXXXXXXX000000000000000000000000000000000000000000000
XXXXXXXXXXXXXXXXXXXXXXXXXXXXXXXXXXX000000000000000000000000000000000000000000000
>P22995
MSRLTIDMTDQQHQSLKALAALQGKTIKQYALERLFPGDADADQAWQELKTMLGNRINDGLAGKVSTKSVGEILDEELSGDRA
XXXXXXXXXXXXXXXXXXXXXXXXXXXXXXXXXXXXXXXXXXXXXXXXXXXXXXX1111111111111111111111111111
XXXXXXXXXXXXXXXXXXXXXXXXXXXXXXXXXXXXXXXXXXXXXXXXXXXXXXX1111111111111111111111111111
XXXXXXXXXXXXXXXXXXXXXXXXXXXXXXXXXXXXXXXXXXXXXXXXXXXXXXX0000000000000000000000000000
>P69428
MGGISIWQLLIIAVIVVLLFGTKKLGSIGSDLGASIKGFKKAMSDDEPKQDKTSQDADFTAKTIADKQADTNQEQAKTEDAKRHDKEQV
XXXXXXXXXXXXXXXXXXXXX11111111111111111111111111111111111111111111111111111111111111111111
XXXXXXXXXXXXXXXXXXXXX00000000000000000000000000000000000000000000000000000000000000000000
XXXXXXXXXXXXXXXXXXXXX00000000000000000000000000000000000000000000000000000000000000000000
>Q03337
MSQRIIQPSASDQQFPGKSDGYEYTVGPKQAITSEASTTYIPSRIYSESLLFKRQEASLSAMAFLFQEMISQLHRTCKTAGDFETKLSDYGHNIGIRLLELLNFRASVSPSSLPRASAFLSQNESSSKLSNASNSPGMLANSSTATSASANERLQEKQTESLSNYITKMRRR
DLKILDILQFIHGTLWSYLFNHVSDDLVKSSERDNEYMIVDNFPTLTQFIPGENVSCEYFVCGIIKGFLFNAGFPCGVTAHRMPQGGHSQRTVYLIQFDRQVLDREGLRFG
11111111111111111111111111111111111111111111111111111111110000000000000000000000000000000000000000000000111111111111111111111111111111111111111111111111111111111111110000000000
00000000000000000000000000000000000000000000000000000000000000000000000000000000000000000000000000000000000
00000000000000000000000000000000000000000000000000000000000000000000000000000000000000000000000000000000000000000000000000000000000000000000000000000000000000000000000000000000
00000000000000000000000000000000000000000000000000000000000000000000000000000000000000000000000000000000000
00000000000000000000000000000000000000000000000000000000000000000000000000000000000000000000000000000000000000000000000000000000000000000000000000000000000000000000000000000000
00000000000000000000000000000000000000000000000000000000000000000000000000000000000000000000000000000000000

00000000000000000000000000000000000000000000000000000000000000000000000000000000000000000000000000000000000000000000000000000000000000000000000000000000000000000000000000000000
00000000000000000000000000000000000000000000000000000000000000000000000000000000000000000000000000000000000
>O50955
MKIKPLIQLKLLGLFLFSCTIDANLNEDYKNKVKGILNKAADDQETTSADTNSNAAKNIPIADNDKVAAELKKQSQAAKTVAAAPNKGSQNQPQTTPNKGSQNQQAAPSPQLQSLSFSADLSNLPKTTAARAASLTKQRIPIQAVTTVPGNTRTFNSRNSGLPTFALN
YSFSQPTRQQTNSSSAVQTTTSSGSKLQTLKNELIRAISEEKNKTQNNFGFRETYDQFKMKDSAFELLDVISSAKVYDRSYAPQLNSNTPEAENERNKFYALMDFDQYKIEQFGSIMEALYNENQNHSLIRELMISGLGTQISFELALEEINKKIEIFNQDYLNAKINSFDF
TMKLKELKSKLNQILDKRKEWSRQADGLIANASSNSSLSDSKSLAEYIKKRYLDNMQNARQSVLEAYISIM
00000000000000000000000000000000000000000000000000000000000000000000000000000000000000000000000000000000000000000000000000000000000000000000000000000000000000000000000000000000
00000000000000000000000000000000000000000000000000000000000000001111100000000000000000000000000000000000000000000000000000000000000000000000000000000000000000000000000000000000
00000000000000000000000000000000000000000000000000000000000
00000000000000000000000000000000000000000000000000000000000000000000000000000000000000000000000000000000000000000000000000000000000000000000000000000000000000000000000000000000
00000000000000000000000000000000000000000000000000000000000000000000000000000000000000000000000000000000000000000000000000000000000000000000000000000000000000000000000000000000
00000000000000000000000000000000000000000000000000000000000
00000000000000000000000000000000000000000000000000000000000000000000000000000000000000000000000000000000000000000000000000000000000000000000000000000000000000000000000000000000
00000000000000000000000000000000000000000000000000000000000000000000000000000000000000000000000000000000000000000000000000000000000000000000000000000000000000000000000000000000
00000000000000000000000000000000000000000000000000000000000
>P20220
MAQTLNSYKMAEIMYKILEKKGELTLEDILAQFEISVPSAYNIQRALKAICERHPDECEVQYKNRKTTFKWIKQEQKEEQKQEQTQDNIAKIFDAQPANFEQTDQGFIKAKQ
0000000000000000000000000000000000000000000000000000000000000000000000001111111111111111111111111111111111111111
0000000000000000000000000000000000000000000000000000000000000000000000000000000000000000000000000000000000000000
0000000000000000000000000000000000000000000000000000000000000000000000000000000000000000000000000000000000000000
>Q49ZM2
MKKLVTATTLTAGIGAAIVGLDHGNEADAAEQTQPTNQSTTQSTSGSSANLYTAGQCTWYVYDKVGGNIGSTWGNANNWASAASSAGYTVNNSPEAGSILQSTAGGYGHVAYVENVNSDGSVEVSEMNYNGGPFSVSERTISAGEASSYNYIHLN
1111111111111111111111111111111111111111111111111000000000000000000000000000000000000000000000000000000000000000000000000000000000000000000000000000000000
00000000000000000000000000000000000000000000000000000000000000000000000000000000000000000000000000000000000000000000000000000000000000000000000000000000000
00000000000000000000000000000000000000000000000000000000000000000000000000000000000000000000000000000000000000000000000000000000000000000000000000000000000
>Q9Q8E9
MTVIKPSSRPRPRKNKNIKVNTYRTSAMDLSPGSVHEGIVYFKDGIFKVRLLGYEGHECILLDYLNYRQDTLDRLKERLVGRVIKTRVVRADGLYVDLRRFF
11111111111111111111111111111111XXXXXXXXXXXXXXXXXXXXXXXXXXXXX111111111111XXXXXXXXXXXXXXXXXXXXXXXXXXXXX
00000000000000000000000000000000XXXXXXXXXXXXXXXXXXXXXXXXXXXXX000000000000XXXXXXXXXXXXXXXXXXXXXXXXXXXXX
00000000000000000000000000000000XXXXXXXXXXXXXXXXXXXXXXXXXXXXX000000000000XXXXXXXXXXXXXXXXXXXXXXXXXXXXX
>Q9KX51
MSVTTETTAGAAAGSDAIVDLRGMWVGVAGLNIFYLIVRIYEQIYGWRAGLDSFAPEFQTYWLSILWTEIPLELVSGLALAGWLWKTRDRNVDAVAPREELRRHVVLVEWLVVYAVAIYWGASFFTEQDGTWHMTVIRDTDFTPSHIIEFYMSYPIYSIMAVGAFF
YAKTRIPYFAHGFSLAFLIVAIGPFMIIPNVGLNEWGHTFWFMEELFVAPLHWGFVFFGWMALGVFGVVLQILMGVKRLIGKDCVAALVG
11111111111111110000000000000000000000000000000000000000000000000000000000000000000000000000000000000000000000000000000000000000000000000000000000000000000000000000000000000000
11111111111111111111111111111111111111111111111111111111111111111111111111111111
00000000000000000000000000000000000000000000000000000000000000000000000000000000000000000000000000000000000000000000000000000000000000000000000000000000000000000000000000000000
00000000000000000000000000000000000000000000000000000000000000000000000000000000
00000000000000000000000000000000000000000000000000000000000000000000000000000000000000000000000000000000000000000000000000000000000000000000000000000000000000000000000000000000
00000000000000000000000000000000000000000000000000000000000000000000000000000000
>P00127
MGMLELVGEYWEQLKITVVPVVAAAEDDNEQHEEKAAEGEEKEEENGDEDEDEDEDEDDDDDDDEDEEEEEEVTDQLEDLREHFKNTEEGKALVHHYEECAERVKIQQQPGYADLEHKEDCVEEFFHLQHYLDTATAPRLFDKLK
11111111111111111111111111111111111111111111111111111111111111111111111000000000000000000000000000000000000000000000000000000000000000000000000000
11111111111111111111111111111111111111111111111111111111111111111111111000000000000000000000000000000000000000000000000000000000000000000000000000
0000000000000000000000000000000000000000000000000000000000000000000000000000000000000000000000000000000000000000000000000000000000000000000000000
>Q980M2
MQANSEEYCELCGSQIRGKGITVSYEGSIITVCNSCYDRIRKHAVIVKADSKKSETRKKATLKPPKMENAELEIVTDYYKIIKTAREQLGISQQQLAQKLKVSENIVKRFESGKLKPTISQARQLEKILGIKLVTPLENNEESEKEFDDTGLTLGDVVNIKEGKK
XXXXXXXXXXXXXXXXXXXXXXXXXXXXXXXXXXXXXXXXXXXXXXXXXXXXXXXXXXXXXXXXXXXXX11111111111100000000000000000000000000000000000000000000000000000001111111111111111111111111111111
XXXXXXXXXXXXXXXXXXXXXXXXXXXXXXXXXXXXXXXXXXXXXXXXXXXXXXXXXXXXXXXXXXXXX000000000000000000000000000000000000000000000000000000000000000000000000000000000000000000000000000000
XXXXXXXXXXXXXXXXXXXXXXXXXXXXXXXXXXXXXXXXXXXXXXXXXXXXXXXXXXXXXXXXXXXXX000000000000000000000000000000000000000000000000000000000000000000000000000000000000000000000000000000
>A8AZZ3
MKKVLLSSVVALTLFAAAAPVFSADEATDAARNNDGAYYLQTQFTNADKVNEYLAQHDGEIRAEAAADPAVVAAKAALDAVEGGSHNYGEVKAAYEAAFNNAFNAVRNKYVQQFQATYNNATEQEGKTYIQGETPEQANARYLKRVGAANNQNPAAEDKGA
TTPASKEEAKKSEAAAKNAGKAAGKALPKTSAVK
XXXXXXXXXXXXXXXXXXXXXXX111111111111111111111XXXXXXXXXXXXXXXXXXXXXXXXXXXXXXXXXXXXXXXXXXXXXXXXXXXXXXXXXXXXXXXXXXXXXXX1111111111111111111XXXXXXXXXXX111111111111111111111111111111
111111111111111111111
XXXXXXXXXXXXXXXXXXXXXXX000000000000000000000XXXXXXXXXXXXXXXXXXXXXXXXXXXXXXXXXXXXXXXXXXXXXXXXXXXXXXXXXXXXXXXXXXXXXXXX0000000000000000000XXXXXXXXXXX000000000000000000000000000000
000000000000000000000
XXXXXXXXXXXXXXXXXXXXXXX111111111111111111111XXXXXXXXXXXXXXXXXXXXXXXXXXXXXXXXXXXXXXXXXXXXXXXXXXXXXXXXXXXXXXXXXXXXXXXX0000000000000000000XXXXXXXXXXX000000000000000000000000000000
000000000000000000000
>Q9L9I0
MKRNNKSAIALIALSLLALSSGAAFAGHHWGNNDGMWQQGGSPLTTEQQATAQKIYDDYYTQTSALRQQLISKRYEYNALLTASSPDTAKINAVAKEMESLGQKLDEQRVKRDVAMAQAGIPRGAGMGYGGCGGYGGGYHRGGGHMGMGNW
0000000000000000000000000000000000000000000000000000000000000000000000000000000000000000000000000000000000000000000000000111111111111111111111111111111
0000000000000000000000000000000000000000000000000000000000000000000000000000000000000000000000000000000000000000000000000000000000000000000000000000000
0000000000000000000000000000000000000000000000000000000000000000000000000000000000000000000000000000000000000000000000000000000000000000000000000000000
>P49785
MLKKQTVWLLTMLSLVVVLSVYYIMSPESKNAVQMQSEKSASDSGEVATEKAPAKQDTKEKSGTETEKGKEDGTKGTKDSSADKETSAEASEKGTVVTETADDDLFTTYRLDLEDARSKEREELNAIVSSDDATAKEKSEAYDKMTALSEVEGTEKQLETLIKTQGYE
DALVNAEGDKINITVKSDKHSKSKATAIIDLVAKEIKTMKDVAVTFEPSK
XXXXXXXXXXXXXXXXXXXXXXXXXXXXXXXX1111111111111111111111111111111111111111111111111111111100000000000000000000000000000000000000000000000000000000000000000000000000000000000000000
000000000000000000000000000000000000000000
XXXXXXXXXXXXXXXXXXXXXXXXXXXXXXXX00000000000000000000000000000000000000000000000000000000000000000000000000000000000000000000000000000000000000000000000000000000000000000000
000000000000000000000000000000000000000000
XXXXXXXXXXXXXXXXXXXXXXXXXXXXXXXX00000000000000000000000000000000000000000000000000000000000000000000000000000000000000000000000000000000000000000000000000000000000000000000
000000000000000000000000000000000000000000
>Q9X0Q3
MDLKKLLPCGKVIVFRKGEIVKHQDDPIEDVLILLEGTLKTEHVSENGKTLEIDEIKPVQIIASGFIFSSEPRFPVNVVAGENSKILSIPKEVFLDLLMKDRELLLFFLKDVSEHFRVVSEKLFFLTTKTLREKLMNFLVRHMNEKRELTLPVTLEELSRLFGCARPALSRVFQELE
REGYIEKHGRRIKVLKNPFEHDRI
00000000000000000000000000000000000000000000000000000000000000000000000000000000000000000000000000000000000000000000000000000000001111111111111111111111111111111111111111111111
1111111111111111111111111
00000000000000000000000000000000000000000000000000000000000000000000000000000000000000000000000000000000000000000000000000000000000000000000000000000000000000000000000000000000
0000000000000000000000000
00000000000000000000000000000000000000000000000000000000000000000000000000000000000000000000000000000000000000000000000000000000000000000000000000000000000000000000000000000000
0000000000000000000000000
>Q9BQ15
MTTETFVKDIKPGLKNLNLIFIVLETGRVTKTKDGHEVRTCKVADKTGSINISVWDDVGNLIQPGDIIRLTKGYASVFKGCLTLYTGRGGDLQKIGEFCMVYSEVPNFSEPNPEYSTQQAPNKAVQNDSNPSASQPTTGPSAASPASENQNGNGLSAPPGPGGGPHP
PHTPSHPPSTRITRSQPNHTPAGPPGPSSNPVSNGKETRRSSKR
XXXXXXXXXX11111111XXXXXXXXX1111111111000000000000000000000000000000000111111111110000000000000000000000000000011111111111111111111111111111111111111111111111111111111111111111
11111111111111111111111111111111111
XXXXXXXXXX00000000XXXXXXXXX00000000000000000000000000000000000000000000000000000000000000000000000000000000000000000000000000000000000000000000000000000000000000000000000000
00000000000000000000000000000000000
XXXXXXXXXX00000000XXXXXXXXX00000000000000000000000000000000000000000000000000000000000000000000000000000000000000000000000000000000000000000000000000000000000000000000000000
00000000000000000000000000000000000
>Q6P3W2
MAVEQMPKKDWYSILGADPSANISDLKQKYQKLILMYHPDKQSTDVPAGTVEECVQKFIEIDQAWKILGNEETKREYDLQRCEDDLRNVGPVDAQVYLEEMSWNEGDHSFYLSCRCGGKYSVSKDEAEEVSLISCDTCSLIIELLHYN
1111111111000000000000000000000000000000000000000000000000000000000000011111111111111100000000000000000000000000000000000000000000000000000000000000
0000000000000000000000000000000000000000000000000000000000000000000000000000000000000000000000000000000000000000000000000000000000000000000000000000
0000000000000000000000000000000000000000000000000000000000000000000000000000000000000000000000000000000000000000000000000000000000000000000000000000
>F2Z293
AQPKSGFSTDRPLWYPGAKAPEYLDGSLVGDYGFDPFGLKPAEYLQYDYDGLDQNLAKNLAGDIIGTRTESADVKSTSLQPYSEVFGLQRFRECELIHGRWAMLATLGALTVEGLTGITWQDAGKVELIEGSSYLGQPLPFSMTTLIWIEVLVIGYIEFQRNAELDTE
KRLYPGGTFDPLGLASDPEKKPILQLAEIKHARLAMVGFLGFAVQAAVTGKGPLNNWVTHLSDPLHTTILDRFL
11111111111111111111111111111111111111111111111111111111111111111111111111111111111111111111111111111111111111111111111111111110000000000000000000000000000000000000000000000000
000000000000000000000000000000000000000000000000000000000000000000
00000000000000000000000000000000000000000000000000000000000000000000000000000000000000000000000000000000000000000000000000000000000000000000000000000000000000000000000000000000
000000000000000000000000000000000000000000000000000000000000000000
00000000000000000000000000000000000000000000000000000000000000000000000000000000000000000000000000000000000000000000000000000000000000000000000000000000000000000000000000000000
000000000000000000000000000000000000000000000000000000000000000000
>A4ZNR2
MDPNTVSSFQDILLRMSKMQLESSSEDLNGMITQFESLKLYRDSLGEAVMRMGDLHSLQNRNEKWREQLGQKFEEIRWLIEEVRHKLKITENSFEQITFMQALHLLLEVEQEIRTFSFQLI
1111111111111111111111111111111111111111111111111111111111111111111111111111111111111111111111111111111111111111111111111
0000000000000000000000000000000000000000000000000000000000000000000000000000000000000000000000000000000000000000000000000
0000000000000000000000000000000000000000000000000000000000000000000000000000000000000000000000000000000000000000000000000
>Q9H981
MTQAEKGDTENGKEKGGEKEKEQRGVKRPIVPALVPESLQEQIQSNFIIVIHPGSTTLRIGRATDTLPASIPHVIARRHKQQGQPLYKDSWLLREGLNKPESNEQRQNGLKMVDQAIWSKKMSNGTRRIPVSPEQARSYNKQMRPAILDHCSGNKWTNTSHHPEY
LVGEEALYVNPLDCYNIHWPIRRGQLNIHPGPGGSLTAVLADIEVIWSHAIQKYLEIPLKDLKYYRCILLIPDIYNKQHVKELVNMILMKMGFSGIVVHQESVCATYGSGLSSTCIVDVGDQKTSVCCVEDGVSHRNTRLCLAYGGSDVSRCFYWLMQRAGFPYRECQL
TNKMDCLLLQHLKETFCHLDQDISGLQDHEFQIRHPDSPALLYQFRLGDEKLQAPMALFYPATFGIVGQKMTTLQHRSQGDPEDPHDEHYLLATQSKQEQSAKATADRKSASKPIGFEGDLRGQSSDLPERLHSQEVDLGSAQGDGLMAGNDSEEALTALMSRKT
AISLFEGKALGLDKAILHSIDCCSSDDTKKKMYSSILVVGGGLMFHKAQEFLQHRILNKMPPSFRRIIENVDVITRPKDMDPRLIAWKGGAVLACLDTTQELWIYQREWQRFGVRMLRERAAFVW
00000000000000000000000000000000000000000000000000000000000000000000000000000000000000000000000000000000000000000000000000000000000000000000000000000000000000000000000000000000
00000000000000000000000000000000000000000000000000000000000000000000000000000000000000000000000000000000000000000000000000000000000000000000000000000000000000000000000000000000
00000000000000000000000000000000000000000000000011111111111111111111111111111111111111111111111111111111111111111111111111111111111111111111111111111111111111111000000000000000
000000000000000000000000000000000000000000000000000000000000000000000000000000000000000000000000
00000000000000000000000000000000000000000000000000000000000000000000000000000000000000000000000000000000000000000000000000000000000000000000000000000000000000000000000000000000
00000000000000000000000000000000000000000000000000000000000000000000000000000000000000000000000000000000000000000000000000000000000000000000000000000000000000000000000000000000
00000000000000000000000000000000000000000000000011111111111111111111111111111111111111111111111111111111111111111111111111111111111111111111111111111111111111111000000000000000
000000000000000000000000000000000000000000000000000000000000000000000000000000000000000000000000
00000000000000000000000000000000000000000000000000000000000000000000000000000000000000000000000000000000000000000000000000000000000000000000000000000000000000000000000000000000
00000000000000000000000000000000000000000000000000000000000000000000000000000000000000000000000000000000000000000000000000000000000000000000000000000000000000000000000000000000
00000000000000000000000000000000000000000000000000000000000000000000000000000000000000000000000000000000000000000000000000000000000000000000000000000000000000000000000000000000
000000000000000000000000000000000000000000000000000000000000000000000000000000000000000000000000
>Q12406

MTLNRKCVVIHNGSHRTVAGFSNVELPQCIIPSSYIKRTDEGGEAEFIFGTYNMIDAAAEKRNGDEVYTLVDSQGLPYNWDALEMQWRYLYDTQLKVSPEELPLVITMPATNGKPDMAILERYYELAFDKLNVPVFQIVIEPLAIALSMGKSSAFVIDIGASGCNVTPII
DGIVVKNAVVRSKFGGDFLDFQVHERLAPLIKEENDMENMADEQKRSTDVWYEASTWIQQFKSTMLQVSEKDLFELERYYKEQADIYAKQQEQLKQMDQQLQYTALTGSPNNPLVQKKNFLFKPLNKTLTLDLKECYQFAEYLFKPQLISDKFSPEDGLGPLMAK
SVKKAGASINSMKANTSTNPNGLGTSHINTNVGDNNSTASSSNISPEQVYSLLLTNVIITGSTSLIEGMEQRIIKELSIRFPQYKLTTFANQVMMDRKIQGWLGALTMANLPSWSLGKWYSKEDYETLKRDRKQSQATNATN
00000000000000000000000000000000000000000000000000000000000000000000000000000000000000000000000000000000000000000000000000000000000000000000000000000000000000000000000000000000
00000000000000000000000000111111111100000000000000000000000000000000000000000000000011111111111110000000000000000000000000000000000000000000000000000000000000000000000000000000
1111111111111111111111111000000000000000000000000000000000000000000000000000000000000000000000000000000000000000000000000
00000000000000000000000000000000000000000000000000000000000000000000000000000000000000000000000000000000000000000000000000000000000000000000000000000000000000000000000000000000
00000000000000000000000000000000000000000000000000000000000000000000000000000000000000000000000000000000000000000000000000000000000000000000000000000000000000000000000000000000
00000000000000000000000000000000000000000000000000000000000000000000000000000000000000000000000000000000000000000000000000000
00000000000000000000000000000000000000000000000000000000000000000000000000000000000000000000000000000000000000000000000000000000000000000000000000000000000000000000000000000000
00000000000000000000000000000000000000000000000000000000000000000000000000000000000000000000000000000000000000000000000000000000000000000000000000000000000000000000000000000000
00000000000000000000000000000000000000000000000000000000000000000000000000000000000000000000000000000000000000000000000000000
>P69723
MENRWQVMIVWQVDRMRIRTWKSLVKHHMYVSGKARGWFYRHHYESPHPRISSEVHIPLGDARLVITTYWGLHTGERDWHLGQGVSIEWRKKRYSTQVDPELADQLIHLYYFDCFSDSAIRKALLGHIVSPRCEYQAGHNKVGSLQYLALAALITPKKIKPPLPS
VTKLTEDRWNKPQKTKGHRGSHTMNGH
00000000000000000000000000000000000000000000000000000000000000000000000000000001111111111111111111111111111111111111111111111111111111111111111111111111111111111111111111111111
1111111111111111
00000000000000000000000000000000000000000000000000000000000000000000000000000000000000000000000000000000000000000000000000000000000000000000000000000000000011111111111111111111
1111111111111111
00000000000000000000000000000000000000000000000000000000000000000000000000000000000000000000000000000000000000000000000000000000000000000000000000000000000000000000000000000000
0000000000000000
>P9WHN5
MAQEQTKRGGGGGDDDDIAGSTAAGQERREKLTEETDDLLDEIDDVLEENAEDFVRAYVQKGGQ
1111111111111111111111111111111111111111111111111111111111111111
0000000000000000000111111111111111111111111111111111111111111000
0000000000000000000000000000000000000000000000000000000000000000
>G8HXD9
MLDLPGNKDKKASSKKSPAKVQSKDRDMGAALRSAYQKTIEEQVPDEMLDLLNKLA
11111111111111111111111111111111111111111111111111111111
00000000000000000000000000011111111111111111111111111111
00000000000000000000000000000000000000000000000000000000
>Q83ZN8
MMNATENQIFVETVSDQELEMLIGGADRGWIKTLTKDCPNVISSICAGTIITACKNCA
XXXXXXXXXXXXXXXXXXXXXXXXX1111111XXX1111111111111111111111X
XXXXXXXXXXXXXXXXXXXXXXXXX0000000XXX0000000000000000000000X
XXXXXXXXXXXXXXXXXXXXXXXXX0000000XXX0000000000000000000000X
>P13338
MTQFSLNDIRPVDETGLSEKELSIKKEKDEIAKLLDRQENGFIIEKMVEEFGMSYLEATTAFLEENSIPETQFAKFIPSGIIEKIQSEAIDENLLRPSVVRCEKTNTLDFLL
1111111111111111111111111111110000000000000000000000000000000000000000000000000000000000000000000000001111111111
0000000000000000000000000000000000000000000000000000000000000000000000000000000000000000000000000000000000000000
0000000000000000000000000000000000000000000000000000000000000000000000000000000000000000000000000000000000000000
>Q04660
MMAKNNKTTEAKMSKKRAASEESDVEEDEDKLLSVDGLIDAEASESDEDDDEYESAVEEKESSSDKEAQDDSDDDSDAELNKLLAEEEGDGEEDYDSSEFSDDTTSLTDRLSGVKLQTIVDPNIYSKYADGSDRIIKPEINPVYDSDDSDAETQNTIGNIPLSAYDEMP
HIGYDINGKRIMRPAKGSALDQLLDSIELPEGWTGLLDKNSGSSLNLTKEELELISKIQRNEQTDDSINPYEPLIDWFTRHEEVMPLTAVPEPKRRFVPSKNEAKRVMKIVRAIREGRIIPPKKLKEMKEKEKIENYQYDLWGDSTETNDHVMHLRAPKLPPPTNEESYN
PPEEYLLSPEEKEAWENTEYSERERNFIPQKYSALRKVPGYGESIRERFERSLDLYLAPRVRKNKLNIDPNSLIPELPSPKDLRPFPIRCSTIYAGHKGKVRTLSIDPSGLWLATGSDDGTVRVWEILTGREVYRTTLIDDEENPDYHIECIEWNPDANNGILAVAVGENIHLI
VPPIFGYDIENNGKTKIEDGFGYDTFGTVKKSNLEVNENGDGDEDGENESAKNAVKKQVAQWNKPSQKQLEKDICITISCKKTVKKLSWHRKGDYFVTVQPDSGNTSVLIHQVSKHLTQSPFKKSKGIIMDAKFHPFKPQLFVCSQRYVRIYDLSQQILVKKLLPGAR
WLSKIDIHPRGDNLIASSFDKRVLWHDLDLASTPYKTLRYHEKAVRSVNFHKKLPLFSSAADDGTIHVFHATVYDDMMKNPMIVPLKKLTGHKVINSLGVLDAIWHPREAWLFSAGADNTARLWTT
00000000000000000000000000000000000000000000000000000000000000000000000000000000000000000000000000000000000000000000000000000000000000000000000000000000000000000000000000000000
00000000000000000000000000000000000000000000000000000000000000000000000000000000000000000000000000000000000000000000000000000000000000000000000000000000000000000000000000000000
00000000000000000000000000000000000000000000000000000000000000000000000000000000000000000000000000000000000000000000000000000000000000000000000000000000000000000000000000000000
00000011111111111111111111111111111111111110000000000000000000000000000000000000000000000000000000000000000000000000000000000000000000000000000000000000000000000000000000000000
0000000000000000000000000000000000000000000000000000000000000000000000000000000000000000000000000000000
00000000000000000000000000000000000000000000000000000000000000000000000000000000000000000000000000000000000000000000000000000000000000000000000000000000000000000000000000000000
00000000000000000000000000000000000000000000000000000000000000000000000000000000000000000000000000000000000000000000000000000000000000000000000000000000000000000000000000000000
00000000000000000000000000000000000000000000000000000000000000000000000000000000000000000000000000000000000000000000000000000000000000000000000000000000000000000000000000000000
00000000000000000000000000000000000000000000000000000000000000000000000000000000000000000000000000000000000000000000000000000000000000000000000000000000000000000000000000000000
0000000000000000000000000000000000000000000000000000000000000000000000000000000000000000000000000000000
00000000000000000000000000000000000000000000000000000000000000000000000000000000000000000000000000000000000000000000000000000000000000000000000000000000000000000000000000000000
00000000000000000000000000000000000000000000000000000000000000000000000000000000000000000000000000000000000000000000000000000000000000000000000000000000000000000000000000000000
00000000000000000000000000000000000000000000000000000000000000000000000000000000000000000000000000000000000000000000000000000000000000000000000000000000000000000000000000000000
00000011111111111111111111111111111111111110000000000000000000000000000000000000000000000000000000000000000000000000000000000000000000000000000000000000000000000000000000000000
0000000000000000000000000000000000000000000000000000000000000000000000000000000000000000000000000000000
>Q58876
MRVEYSKDLIRKGISTISQLKKAKIRVEKDDKKISYKDAKPGKIDVNEFKKAIYLLIEADDFLYKKAPKHELNEEEAKEFCKLIIKCQEHLNKILANFGFEFEEKEIDEGALYIVSNKKLFKKLKNKNPNLKVVCTEGMLDIEDMRAIGVPEKALEGLKKKVEIARKNVERFIEKY
KPEKIFVVVEDDKDELLYLRAKNLYNAEKLDADEILD
00000000000000000000000000000000000000000000000000000000000000000000000000000000000000000000000000000001111111111111111111111111111111111111111111111111111111111111111111111111
1111111111111111111111111111111111111
00000000000000000000000000000000000000000000000000000000000000000000000000000000000000000000000000000000000000000000000000000000000000000000000000000000000000000000000000000000
0000000000000000000000000000000000000
00000000000000000000000000000000000000000000000000000000000000000000000000000000000000000000000000000000000000000000000000000000000000000000000000000000000000000000000000000000
0000000000000000000000000000000000000
>P9WJ35
MALSIKHPEADRLARALAARTGETLTEAVVTALRERLARETGRARVVPLRDELAAIRHRCAALPVVDNRSAEAILGYDERGLPA
111111111111111111111111111111111111111111111000000000000000000000000000000000000000
000000000000000000000000000000000000000000000000000000000000000000000000000000000000
000000000000000000000000000000000000000000000000000000000000000000000000000000000000
>Q5JH72
MDRKLDEFIGDATPKKVSKEKPVRRKKRLKPTSLDSFLPEEHINYFRDLRIGSKKIRNAKIEEL
1111111111111111111111110000000000000000000000000000000000000000
0000000000000000000000000000000000000000000000000000000000000000
0000000000000000000000000000000000000000000000000000000000000000
>Q8GY31
MGRSIFSFFTKKKKMAMARSISYITSTQLLPLHRRPNIAIIDVRDEERNYDGHIAGSLHYASGSFDDKISHLVQNVKDKDTLVFHCALSQVRGPTCARRLVNYLDEKKEDTGIKNIMILERGFNGWEASGKPVCRCAEVPCKGDCA
00000000000000000000000000000000000000000000000000000000000000000000000000000000000000000000000000000000000000000000000000000000011111111111111111
00000000000000000000000000000000000000000000000000000000000000000000000000000000000000000000000000000000000000000000000000000000000000000000000000
00000000000000000000000000000000000000000000000000000000000000000000000000000000000000000000000000000000000000000000000000000000000000000000000000
>A0MHA3
MNKKNILPQQGQPVIRLTAGQLSSQLAELSEEALGDAGLEASVTACITFCAYDGVEPSCTLCYTLCAYDGE
0000000000000000000000000000000001111111111111111111111111111111XXXXXXX
0000000000000000000000000000000000000000000000000000000000000000XXXXXXX
0000000000000000000000000000000000000000000000000000000000000000XXXXXXX
>Q9BQB4
MQLPLALCLVCLLVHTAFRVVEGQGWQAFKNDATEIIPELGEYPEPPPELENNKTMNRAENGGRPPHHPFETKDVSEYSCRELHFTRYVTDGPCRSAKPVTELVCSGQCGPARLLPNAIGRGKWWRPSGPDFRCIPDRYRAQRVQLLCPGGEAPRARKVRLVASC
KCKRLTRFHNQSELKDFGTEAARPQKGRKPRPRARSAKANQAELENAY
XXXXXXXXXXXXXXXXXXXXXXXX1111111111111111111111111111111111111111111111111111111XXXXXXXXXXXXXXXXXXXXXXXXXXXXX111111111111111111111111XXXXXXXXXXXXXXXXXXXXXXXXXXXXXXXXXX111111
111111111111111111111111111111111111111
XXXXXXXXXXXXXXXXXXXXXXXX0000000000000000000000000000000000000000000000000000000XXXXXXXXXXXXXXXXXXXXXXXXXXXXX111111111111111111111111XXXXXXXXXXXXXXXXXXXXXXXXXXXXXXXXXX000000
000000000000000000000000000000000000000
XXXXXXXXXXXXXXXXXXXXXXXX0000000000000000000000000000000000000000000000000000000XXXXXXXXXXXXXXXXXXXXXXXXXXXXX000000000000000000000000XXXXXXXXXXXXXXXXXXXXXXXXXXXXXXXXXX000000
000000000000000000000000000000000000000
>Q5ZYC9
MGNNTDDSARNPFGFYTPPRVKEIGEPDVTDATLGSVYSEIISPVKDCILTVAKAVSFNPGGKDNTDAVEVLTELNTKVERAALNQPILTTKTERMFGAAESEKSSEPPSHDERGFKLSS
111111111111111111111111100000000000000000000000000000000000000000000011111111111111111111111111111111111111111111111111
000000000000000000000000000000000000000000000000000000000000000000000000000000000000000000000000000000000000000000000000
000000000000000000000000000000000000000000000000000000000000000000000000000000000000000000000000000000000000000000000000
>O88998
MSVPLLKIGVVLSTMAMITNWMSQTLPSLVGLNTTRLSAASGGTLDRSTGVLPTNPEESWQVYSSAQDSEGRCICTVVAPQQTMCSRDARTKQLRQLLEKVQNMSQSIEVLDRRTQRDLQYVEKMENQMKGLETKFKQVEESHKQHLARQFKAIKAKMDELRP
LIPVLEEYKADAKLVLQFKEEVQNLTSVLNELQEEIGAYDYDELQSRVSNLEERLRACMQKLACGKLTGISDPVTVKTSGSRFGSWMTDPLAPEGDNRVWYMDGYHNNRFVREYKSMVDFMNTDNFTSHRLPHPWSGTGQVVYNGSIYFNKFQSHIIIRFDLKTEA
ILKTRSLDYAGYNNMYHYAWGGHSDIDLMVDENGLWAVYATNQNAGNIVISKLDPVSLQILQTWNTSYPKRSAGEAFIICGTLYVTNGYSGGTKVHYAYQTNASTYEYIDIPFQNKYSHISMLDYNPKDRALYAWNNGHQTLYNVTLFHVIRSDEL
00000000000000000000000000000000000000000000000000000000000000000000000000000000000000000000000000000000000000000000000000000000000000000000000000000000000000000000000000000000
00000000000000000000000000000000000000000000000000000000000000000000000000000000000000000000000000000000000000000000000000000000000000000000000000000000000000000001111111111111
0000000000000000000000000000000000000000000000000000000000000000000000000000000000000000000000000000000000000000000000000000000000000
00000000000000000000000000000000000000000000000000000000000000000000000000000000000000000000000000000000000000000000000000000000000000000000000000000000000000000000000000000000
00000000000000000000000000000000000000000000000000000000000000000000000000000000000000000000000000000000000000000000000000000000000000000000000000000000000000000000000000000000
0000000000000000000000000000000000000000000000000000000000000000000000000000000000000000000000000000000000000000000000000000000000000
00000000000000000000000000000000000000000000000000000000000000000000000000000000000000000000000000000000000000000000000000000000000000000000000000000000000000000000000000000000
00000000000000000000000000000000000000000000000000000000000000000000000000000000000000000000000000000000000000000000000000000000000000000000000000000000000000000000000000000000
0000000000000000000000000000000000000000000000000000000000000000000000000000000000000000000000000000000000000000000000000000000000000
>Q647G9
MAKSTILVALLALVLVAHASAMRRERGRQGDSSSCERQVDRVNLKPCEQHIMQRIMGEQEQYDSYDIRSTRSSDQQQRCCDELNEMENTQRCMCEALQQIMENQCDRLQDRQMVQQFKRELMNLPQQCNFRAPQRCDLDVSGGRC
XXXXXXXXXXXXXXXXXXXXX11111111111110000000000000000000000111111111111111XXXXXXXXXXXXX1111110000000000000000111111100000000000000000111111111111111
XXXXXXXXXXXXXXXXXXXXX00000000000000000000000000000000000000000000000000XXXXXXXXXXXXX0000000000000000000000000000000000000000000000000000000000000
XXXXXXXXXXXXXXXXXXXXX00000000000000000000000000000000000000000000000000XXXXXXXXXXXXX0000000000000000000000000000000000000000000000000000000000000
>P17677
MLCCMRRTKQVEKNDDDQKIEQDGIKPEDKAHKAATKIQASFRGHITRKKLKGEKKDDVQAAEAEANKKDEAPVADGVEKKGEGTTTAEAAPATGSKPDEPGKAGETPSEEKKGEGDAATEQAAPQAPASSEEKAGSAETESATKASTDNSPSSKAEDAPAKEEP
KQADVPAAVTAAAATTPAAEDAAAKATAQPPTETGESSQAEENIEAVDETKPKESARQDEGKEEEPEADQEHA
11111111111111111111111111111111111111111111111111111111111111111111111111111111111111111111111111111111111111111111111111111111111111111111111111111111111111111111111111111111
11111111111111111111111111111111111111111111111111111111111111

00000000000000000000000000000000000000000000000000000000000000000000000000000000000000000000000000000000000000000000000000000000000000000000000000000000000000000000000000000000
00000000000000000000000000000000000000000000000000000000000000
00000000000000000000000000000000000000000000000000000000000000000000000000000000000000000000000000000000000000000000000000000000000000000000000000000000000000000000000000000000
00000000000000000000000000000000000000000000000000000000000000
>Q9H832
MAESPTEEAATAGAGAAGPGASSVAGVVGVSGSGGGFGPPFLPDVWAAAAAAGGAGGPGSGLAPLPGLPPSAAAHGAALLSHWDPTLSSDWDGERTAPQCLLRIKRDIMSIYKEPPPGMFVVPDTVDMTKIHALITGPFDTPYEGGFFLFVFRCPPDYPIHPPR
VKLMTTGNNTVRFNPNFYRNGKVCLSILGTWTGPAWSPAQSISSVLISIQSLMTENPYHNEPGFEQERHPGDSKNYNECIRHETIRVAVCDMMEGKCPCPEPLRGVMEKSFLEYYDFYEVACKDRLHLQGQTMQDPFGEKRGHFDYQSLLMRLGLIRQKVLERLH
NENAEMDSDSSSSGTETDLHGSLRV
11111111111111111111111111111111111111111111111111111111111111111111111111111111111111111111111111000000000000000000000000000000000000000000000000000000000000000000000000000000
00000000000000000000000000000000000000000000000000000000000000000000000000000000000000000000000000000000000000000000000000000000011111111111111111111111111111111111111111111111111
11
00000000000000000000000000000000000000000000000000000000000000000000000000000000000000000000000000000000000000000000000000000000000000000000000000000000000000000000000000000000
00000000000000000000000000000000000000000000000000000000000000000000000000000000000000000000000000000000000000000000000000000000000000000000000000000000000000000000000000000000
00
00000000000000000000000000000000000000000000000000000000000000000000000000000000000000000000000000000000000000000000000000000000000000000000000000000000000000000000000000000000
00000000000000000000000000000000000000000000000000000000000000000000000000000000000000000000000000000000000000000000000000000000000000000000000000000000000000000000000000000000
00
>P06836
MLCCMRRTKQVEKNDEDQKIEQDGIKPEDKAHKAATKIQASFRGHITRKKLKGEKKGDAPAAEAEANEKDEAAVAEGTEKKEGEGSTPAEAAPGAGPKPEEKTGKAGETPSEEKKGEGAPDAATEQAAPQAPAPSEEKAGSAETESATKASTDNSPSSKAEDAPA
KEEPKQADVPAAVTAAAATAPAAEDAAAMATAQPPTETAESSQAEEKIEAVDETKPKDSARQDEGKGEEREADQEHA
11111111111111111111111111111111111111111111111111111111111111111111111111111111111111111111111111111111111111111111111111111111111111111111111111111111111111111111111111111111
111111111111111111111111111111111111111111111111111111111111111111
00000000000000000000000000000000000000000000000000000000000000000000000000000000000000000000000000000000000000000000000000000000000000000000000000000000000000000000000000000000
000000000000000000000000000000000000000000000000000000000000000000
00000000000000000000000000000000000000000000000000000000000000000000000000000000000000000000000000000000000000000000000000000000000000000000000000000000000000000000000000000000
000000000000000000000000000000000000000000000000000000000000000000
>Q63K18
MSNPPTPLLADYEWSGYLTGIGRAFDDGVKDLNKQLQDAQANLTKNPSDPTALANYQMIMSEYNLYRNAQSSAVKSMKDIDSSIVSNFR
1111111XX111111111111111111000000000000000000000000000000000000000000000000000000000000000
0000000XX000000000000000000000000000000000000000000000000000000000000000000000000000000000
0000000XX000000000000000000000000000000000000000000000000000000000000000000000000000000000
>C0J347
MSARIQVPLSQRRHTNVAVVRYTKNGVRLEIACYKNKVISYRGGIETRMDEVLQVERVFTNVSRGLYSSEKEIEAVFGKGTSEKEALQYILDHGELQVAQQERAAEIDQMFIDIAVIISQKCVNEVTQRPFPSQVIEQALHSIGAAVKLDQPVKKQALAFIHQLIDAKTIP
IARARMKLRCVVPDEPSLEKLVEWCETNGTSILQKVVEAGGSDAALQIHSLLILLQPHLFRDIERFVKTEMPPGGSVHVIENAAMDVGEGDVMDAELIARANAHMTGASGVGNADKSHCTGSSSNPHAESNRGRRKGKGGSRKPQGVQRVLGKDDGTDQQTVT
TAELSSTPAVVGENDDELKVALSKLVLDETNGSRDEDYDGKGRRGKKKAKRRQGEPHQKQQQQPATPQKTVEVDEQQGSDEEVLVNRKQRKQAAARVRVKDDKRNEYWNDADDDDYDYGYEDEDAGEVRD
00000000000000000000000000000000000000000000000000000000000000000000000000000000000000000000000000000000000000000000000000000000000000000000000000000000000000000000000000000000
00000000000000000000000000000000000000000000000000000000000000000000000000000000000000001111111111111111111111111111111111111111111111111111111111111111111111111111111111111111
1111111111111111111111111111111111111111111111111111111111111111111111111111111111111111111111111111111111111111
00000000000000000000000000000000000000000000000000000000000000000000000000000000000000000000000000000000000000000000000000000000000000000000000000000000000000000000000000000000
00000000000000000000000000000000000000000000000000000000000000000000000000000000000000000000000000000000000000000000000000000000000000000000000000000000000000000000000000000000
0000000000000000000000000000000000000000000000000000000000000000000000000000000000000000000000000000000000000000
00000000000000000000000000000000000000000000000000000000000000000000000000000000000000000000000000000000000000000000000000000000000000000000000000000000000000000000000000000000
00000000000000000000000000000000000000000000000000000000000000000000000000000000000000001111111111111111111111111111111111111111111111111111111111111111111111111111111111111111
1111111111111111111111111111111111111111111111111111111111111111111111111111111111111111111111111111111111111111
>P46984
MKLPVAQYSAPDGVEKSFAPIRDDPRYMTTEGRTTGPSDHVLNAGQIDRDKPSEPERTKDGSQLTYLGQLRTQLTGLQDDINEFLTGRMELAKNKKKAGADEKRIQEEINQLLDGGDGDEDAV
111111111111111111111111111111111111111111111111111111111111111111111111111111111111111111111111111111111111111111111111111
011111111111111111111000000000000000000000000001111111111111111111111111111111111111111111111111111111111111111111000000000
000000000000000000000000000000000000000000000000000000000000000000000000000000000000000000000000000000000000000000000000000
>P0DJ89
MINNVSSLFPTVNRNITAVYKKSSFSVSPQKITLNPVKISSPFSPSSSSISATTLFRAPNAHSASFHRQSTAESSLHQQLPNVRQRLIQHLAEHGIKPARSMAEHIPPAPNWPAPPPPVQNEQSRPLPDVAQRLVQHLAEHGIQPARNMAEHIPPAPNWPAPPLPVQN
EQSRPLPDVAQRLVQHLAEHGIQPARSMAEHIPPAPNWPAPPPPVQNEQSRPLPDVAQRLMQHLAEHGIQPARNMAEHIPPAPNWPAPTPPVQNEQSRPLPDVAQRLMQHLAEHGIQPARNMAEHIPPAPNWPAPTPPVQNEQSRPLPDVAQRLMQHLA
EHGINTSKRS
XXXXXXXXXXXXXXXXXXXXXXXXXXXXXXXXXXXXXXXXXXXXXXXXXXXXXXXXXXXXXXXXXXXXXXXXXXXXXXXXXXXXXXXXXXXXXXXXXXXXXXXXXXXXXXXXXXXXXXXXXXXXXXXXXXXXXXXXXXXXXXXXXXXXXXXXXXXXXXXXXXXXXXXXXXX
XXXXXXXXXXXXXXXXXXXXXXXXXXXXXXXXXXXXXXXXXXXXXXXXXXXXXXXXXXXXXXXXXXXXXXXXXXXXXXXXXXXXXXXXXXXXXXXXXXXXXXXXXXXXX111111111XXXX111111111111111111111XXXXXXXXXXXXXXXXXXXXXXXXX
XXXXXXXXXXXXXXXXXXXXXXXXXXXXXXXXXXXXXXXXXXXXXXXXXXXXXXXXXXXXXXXXXXXXXXXXXXXXXXXXXXXXXXXXXXXXXXXXXXXXXXXXXXXXXXXXXXXXXXXXXXXXXXXXXXXXXXXXXXXXXXXXXXXXXXXXXXXXXXXXXXXXXXXXXXXXXX
XXXXXXXXXXXXXXXXXXXXXXXXXXXXXXXXXXXXXXXXXXXXXXXXXXXXXXXXXXXXXXXXXXXXXXXXXXXXXXXXXXXXXXXXXXXXXXXXXXXXXXXXXXXXX111111111XXXX111111111111110000000XXXXXXXXXXXXXXXXXXXXXXXXX
XXXXXXXXXXXXXXXXXXXXXXXXXXXXXXXXXXXXXXXXXXXXXXXXXXXXXXXXXXXXXXXXXXXXXXXXXXXXXXXXXXXXXXXXXXXXXXXXXXXXXXXXXXXXXXXXXXXXXXXXXXXXXXXXXXXXXXXXXXXXXXXXXXXXXXXXXXXXXXXXXXXXXXXXXXXXXX
XXXXXXXXXXXXXXXXXXXXXXXXXXXXXXXXXXXXXXXXXXXXXXXXXXXXXXXXXXXXXXXXXXXXXXXXXXXXXXXXXXXXXXXXXXXXXXXXXXXXXXXXXXXXX000000000XXXX000000000000000000000XXXXXXXXXXXXXXXXXXXXXXXXX
>Q86FP9
MKHFAILILAVVASAVVMAYPERDSAKEGNQEQERALHVKVQKRTDGDADYDEYEEDGTTPTPDPTAPTAKPRLRGNKP
XXXXXXXXXXXXXXXXXX1111111111111111111111111111111111111111111111111111111111111
XXXXXXXXXXXXXXXXXXX000000000000000000000000000000111111111111111111111111100000
XXXXXXXXXXXXXXXXXXX000000000000000000000000000000000000000000000000000000000000
>Q86FP8
MKHFVILILAVVASAVVMAYPERDSAKDGNQEKERALLVKVQERYQGNQGDYDEYDQDETTPPPDPTAQTARPRLRQNQD
XXXXXXXXXXXXXXXXXX11111111111111111111111111111111111111111111111111111111111111
XXXXXXXXXXXXXXXXXX11111111111111111111111111111111111111111111111111111111111111
XXXXXXXXXXXXXXXXXX00000000000000000000000000000000000000000000000000000000000000
>P12357
MAAATASLSSTLLAPCSSKQPQPQQQHQHQQLKCKSFSGLRPLKLNISSNNSSSSLSMSSARRSMTCRAELSPSLVISLSTGLSLFLGRFVFFNFQRENMAKQVPEQNGMSHFEAGDTRAKEYVSLLKSNDPVGFNIVDVLAWGSIGHIVAYYILATASNGYDPSFF
XXXXXXXXXXXXXXXXXXXXXXXXXXXXXXXXXXXXXXXXXXXXXXXXXXXXXXXXXXXXXXXXXXX11111111111111111111111111111111111111111111111111111111111111111111111111111111111111111111111111111111
XXXXXXXXXXXXXXXXXXXXXXXXXXXXXXXXXXXXXXXXXXXXXXXXXXXXXXXXXXXXXXXXXXX00000000000000000000000000000000000000000000000000000000000000000000000000000000000000000000000000000000
XXXXXXXXXXXXXXXXXXXXXXXXXXXXXXXXXXXXXXXXXXXXXXXXXXXXXXXXXXXXXXXXXXX00000000000000000000000000000000000000000000000000000000000000000000000000000000000000000000000000000000
>Q87WW0
MVDKLTHLKQLEAESIHIIREVAAEFDNPVMLYSIGKDSAVMLHLARKAFFPGKLPFPVMHVDTRWKFQEMYRFRDQMVEEMGLDLITHINPDGVAQGINPFTHGSAKHTDIMKTEGLKQALDKHGFDAAFGGARRDEEKSRAKERVYSFRDSKHRWDPKNQR
PELWNVYNGNVNKGESIRVFPLSNWTELDIWQYIYLEGIPIVPLYFAAERDVIEKNGTLIMIDDERILEHLTDEEKSRIVKKKVRFRTLGCYPLTGAVESEATSLTDIIQEMLLTRTSERQGRVIDHDGAGSMEEKKRQGYF
00000000000000000000000000000000000000000000000000000000000000000000000000000000000000000000000000000000000000000000000000000000000000000000000000000000000000000000000000000000
000000000000000000000000000000000000000000000000000000000000000000000000000000000000111111111111111111111111111111111111111111111
00000000000000000000000000000000000000000000000000000000000000000000000000000000000000000000000000000000000000000000000000000000000000000000000000000000000000000000000000000000
000000000000000000000000000000000000000000000000000000000000000000000000000000000000000000000000000000000000000000000000000000000
00000000000000000000000000000000000000000000000000000000000000000000000000000000000000000000000000000000000000000000000000000000000000000000000000000000000000000000000000000000
000000000000000000000000000000000000000000000000000000000000000000000000000000000000000000000000000000000000000000000000000000000
>A0A0H3CFC9
MIEHVPMEDKRKGAAALDEARLRQQAIGVKLRQMFDEVVNEPVPDEFLAILRKAERPAGGE
1111111111111111111111000000000000000000000000000000000111111
0000000000000000000000000000000000000000000000000000000000000
0000000000000000000000000000000000000000000000000000000000000
>P0ACX9
MRYTDSRKLTPETDANHKTASPQPIRRISSQTLLGPDGKLIIDHDGQEYLLRKTQAGKLLLTK
111111111111111111100000000000000000000000000000000000000000000
000000000000000000000000000000000000000000000000000000000000000
000000000000000000000000000000000000000000000000000000000000000
>P12355
MSFTIPTNLYKPLATKPKHLSSSSFAPRSKIVCQQENDQQQPKKLELAKVGANAAAALALSSVLLSSWSVAPDAAMADIAGLTPCKESKQFAKREKQALKKLQASLKLYADDSAPALAIKATMEKTKKRFDNYGKYGLLCGSDGLPHLIVSGDQRHWGEFITPGILFLYI
AGWIGWVGRSYLIAIRDEKKPTQKEIIDVPLASSLLFRGFSWPVAAYRELLNGELVDNNF
11111111111111111111111111111111111111111111111111111111111111111111111111111111111111111111111100000000000000000000000000000000000000000000000000000000000000000000000000000000
0000000000000000000000000000000000000000000000000000000
00000000000000000000000000000000000000000000000000000000000000000000000000000000000000000000000000000000000000000000000000000000000000000000000000000000000000000000000000000000
0000000000000000000000000000000000000000000000000000000
00000000000000000000000000000000000000000000000000000000000000000000000000000000000000000000000000000000000000000000000000000000000000000000000000000000000000000000000000000000
0000000000000000000000000000000000000000000000000000000
>Q9I332
MLKLNAVDTAPLVSSDTLAPLPPLRAQQIAFEQALPAHRPPAPRPPFDKGDETTEAAATADAPTSTPLADQPAAPAADRPPTIRQPPMPVAADATPTPTPTPTPTPTPTPTPTPTVSPSGSVARQAPAVSARVAASTQAREPASVSAPPVDEPPLVPVSSHPQIA
GRTHERPQPGPGFPAKTAAEVAPTAQASVQASPPAPTAGGEGRGEERRQPGETDPSALPPDDQAPVPLPAMQTPGDRLLARLLASSGSRPLPLADLARLLDAVQGRIQVASAAESHAARLQVRLPQLGAVEVQVLHGHGQLQIEISASPGSLALLQQARGELLER
LQRLHPEQPVQLTFNQQQDSGQRSRQRRYLHEEWQAE
11111111111111111111111111111111111111111111111111111111111111111111111111111111111111111111111111111111111111111111111111111111111111111111111111111111111111111111111111111111
11111111111111111111111111111111111111111111111111111111111111111111111111111111111111111000000000000000000000000000000000000000000000000000000000000000000000000000000000000000
00000000000000000
00000000000000000001111111111111111111100000000000000000000000000000000000000000000000000000000000000000000000000000000000000000000000000000000000000000000000000000000000000000
00000000000000000000000000000000000000000000000000000000000000000000000000000000000000000000000000000000000000000000000000000000000000000000000000000000000000000000000000000000
00000000000000000
00000000000000000000000000000000000000000000000000000000000000000000000000000000000000000000000000000000000000000000000000000000000000000000000000000000000000000000000000000000
00000000000000000000000000000000000000000000000000000000000000000000000000000000000000000000000000000000000000000000000000000000000000000000000000000000000000000000000000000000
00000000000000000
>P39015
MSNPFDLLGNDVEDADVVVLPPKEIVKSNTSSKKADVPPPSADPSKARKNRPRPSGNEGAIRDKTAGRRNNRSKDVTDSATTKKSNTRRATDRHSRTGKTDTKKKVNQGWGDDKKELSAEKEAQADAAAEIAEDAAEAEDAGKPKTAQLSLQDYLNQQANNQF
NKVPEAKKVELDAERIETAEKEAYVPATKVKNVKSKQLKTKEYLEFDATFVESNTRKNFGDRNNNSRNNFNNRRGGRGARKGNNTANATNSANTVQKNRNIDVSNLPSLA
11111111XXXXXXXXXXXXXXXXXXXXXXXXXXXXXXXXXXXXXXXXXXXXXXXXXXXXXXXXXXXXXXXXXXXXXXXXXXX111111111111111111111111111111111111XXXXXXXXXXXXXXXXXXXX111111111111111111111111111XXXXXX
XX1111111111111111111111111111111111111111111111111111111111111111111111111111111111111111111111
00000000XXXXXXXXXXXXXXXXXXXXXXXXXXXXXXXXXXXXXXXXXXXXXXXXXXXXXXXXXXXXXXXXXXXXXXXXXXX000000000000000000000000000000000000XXXXXXXXXXXXXXXXXXXX000000000000000000000000000XXXXXX
XX0000000000000000000000000000000000000000000000000000000000000000000000000000000000000000000000
00000000XXXXXXXXXXXXXXXXXXXXXXXXXXXXXXXXXXXXXXXXXXXXXXXXXXXXXXXXXXXXXXXXXXXXXXXXXXX000000000000000000000000000000000000XXXXXXXXXXXXXXXXXXXX000000000000000000000000000XXXXXX
XX0000000000000000000000000000000000000000000000000000000000000000000000000000000000000000000000
>P01097
MLPRSALARSLQLQRGVAARFYSEGSTGTPRGSGSEDSFVKRERATEDFFVRQREKEQLRHLKEQLEKQRKKIDSLENKIDSMTK
XXXXXXXXXXXXXXXXXXXXXXXXXXX1111111111111111111111XXXXXXXXXXXXXXXXXXXXXXXXXXXXXXXXXXXX
XXXXXXXXXXXXXXXXXXXXXXXXXXX1111111111111111111111XXXXXXXXXXXXXXXXXXXXXXXXXXXXXXXXXXXX

XXXXXXXXXXXXXXXXXXXXXXXXXXX00000000000000000000000XXXXXXXXXXXXXXXXXXXXXXXXXXXXXXXXXXXX
>P97303
MSVDEKPGSPMYVYESTVHCANILLGLNDQRKKDILCDVTLIVERKEFRAHRAVLAACSEYFWQALVGQTKDDLVVSLPEEVTARGFGPLLQFAYTAKLLLSRENIREVIRCAEFLRMHNLEDSCFSFLQTQLLNREDGLFVCRKDSACQRPQEDHGNSAGEEEEEEE
TMDSETARMACATDQMLPDPISFEATAIPVAEKEEALLPESEVPTDTKENSEKGALTQYPRYKKYQLACTKNVYSAPSHGTSGFASTFSEDSPGNSLKPGLPMGQIKSEPPSEETEEESITLCLSGDETDIKDRPGDVEMDRKQPSPAPTPSTPTGAACLDRSRSVSSPS
CLRSLFGITKGVESTGLPSTSQQPLVRSSACPFNKGISQGDLKTDYTPLAGNYGQPHVGQKDVSNFAMGSPLRGPGPETLCKQEGELDRRSVIFSASACDQPNTPVHSYSAVSNLDKDLSEPVPKSLWVGAGQSLPSSQAYSHSGLMADHLPGRIRPNTSCPVPIKV
CPRSPPLETRTRTSSSCSSYSYAEDGSGGSPCSLPLCEFSSSPCSQGARFLATEHQEPGLMGDGMYNQVRPQIKCEQSYGTNSSDESGSFSEADSESCPVQDRGQEVKLPFPVDQITDLPRNDFQMMIKMHKLTSEQLEFIHDIRRRSKNRIAAQRCRKRKLDCIQNL
ECEIRKLVCEKEKLLSERNHLKACMGELLDNFSCLSQEVCRDIQSPEQIQALHRYCPVLIPMDLPGASVNPPPVGVEQSLAPSPCAVGGSVPCCLEPGAAPPGLPWVPSNTSENCTSGRRLEGSDPGTFSERGPPLEARSQSVTVDFCQEMTEKCTTDEQPRKDYA
00000000000000000000000000000000000000000000000000000000000000000000000000000000000000000000000000000000000000000000000000000000000000000000000000000000000000000000000000
00000000000000000000000000000000000000000000000000000000000000000000000000000000000000000000000000000000000000000000000000000000000000000000000000000000111111111111111111111111
11111111111111111111111111111111111111111111111111111111111111111111111111111111111111111111111111111111111111111111111111111111111111111111111111111111111111111110000000000
00000000000000000000000000000000000000000000000000000000000000000000000000000000000000000000000000000000000000000000000000000000000000000000000000000000000000000000000000
00000000000000000000000000000000000000000000000000000000000000000000000000000000000000000000000000000000000000000000000000000000000000000000000000000000000000000000000000
000000000000000000000000000000000000000000000000000000000000000000000000000000000000000000000000000000000000000000000000000000000
00000000000000000000000000000000000000000000000000000000000000000000000000000000000000000000000000000000000000000000000000000000000000000000000000000000000000000000000000
00000000000000000000000000000000000000000000000000000000000000000000000000000000000000000000000000000000000000000000000000000000000000000000000000000000000000000000000000
00000000000000000000000000000000000000000000000000000000000000000000000000000000000000000000000000000000000000000000000000000000000000000000000000000000000000000000000000
00000000000000000000000000000000000000000000000000000000000000000000000000000000000000000000000000000000000000000000000000000000000000000000000000000000000000000000000000
00000000000000000000000000000000000000000000000000000000000000000000000000000000000000000000000000000000000000000000000000000000000000000000000000000000000000000000000000
000000000000000000000000000000000000000000000000000000000000000000000000000000000000000000000000000000000000000000000000000000000
00000000000000000000000000000000000000000000000000000000000000000000000000000000000000000000000000000000000000000000000000000000000000000000000000000000000000000000000000
00000000000000000000000000000000000000000000000000000000000000000000000000000000000000000000000000000000000000000000000000000000000000000000000000000000000000000000000000
00000000000000000000000000000000000000000000000000000000000000000000000000000000000000000000000000000000000000000000000000000000000000000000000000000000000000000000000000
00000000000000000000000000000000000000000000000000000000000000000000000000000000000000000000000000000000000000000000000000000000000000000000000000000000000000000000000000
00000000000000000000000000000000000000000000000000000000000000000000000000000000000000000000000000000000000000000000000000000000000000000000000000000000000000000000000000
000000000000000000000000000000000000000000000000000000000000000000000000000000000000000000000000000000000000000000000000000000000
>P27392
MDKKKLLYWVGGGLVLILIWLWFRNRPAAQVASNWEGPPYMTYNQPQAGSVTLPVAGYTSPSPTLPNRNRSCGCNPAVSAAMAQGADLASKLTDSITSQLNDYASSLNDYLASQAGV
000000000000000000000000000000000000000000000000000000000000000011111111111111111111111111111111111110000000000000000
000000000000000000000000000000000000000000000000000000000000000000000000000000000000000000000000000000000000000000000
000000000000000000000000000000000000000000000000000000000000000000000000000000000000000000000000000000000000000000000
>O94742
MSTDVAAAQAQSKIDLTKKKNEEINKKSLEEDDEFEDFPIDTWANGETIKSNAVTQTNIWEENWDDVEVDDDFTNELKAELDRYKRENQ
11111111111111111111111111111111111111111111111111111111111111111111111111111111111111111
11111111111111111111111111111111111111111111111111111111111111111111111111111111111111111
00000000000000000000000000000000000000000000000000000000000000000000000000000000000000000
>Q9FUW7
MKTFLIFVLLAMAMKIATAARELNPSNKELQSPQQSFSYQQQPFPQQPYPQQPYPSQQPYPSQQPFPTPQQQFPEQSQQPFTQPQQPTPIQPQQPFPQQPQQPQQPFPQPQQPFPWQPQQPFPQTQQSFPLQPQQPFPQQPQQPFPQPQLPFPQQSEQII
PQQLQQPFPLQPQQPFPQQPQQPFPQPQQPIPVQPQQSFPQQSQSQQPFAQPQQLFPELQQPIPQQPQQPFPLQPQQPFPQQPQQPFPQQPQQSFPQQPQQPYPQQQPYGSSLTSIGGQ
11111111111111111111111111111111111111111111111111111111111111111111111111111111111111111111111111111111111111111111111111111111111111111111111111111111111111111111111111
11111111111111111111111111111111111111111111111111111111111111111111111111111111111111111111111111111111
00000000000000000000000000000000000000000000000000000000000000000000000000000000000000000000000000000000000000000000000000000000000000000000000000000000000000000000000000
00000000000000000000000000000000000000000000000000000000000000000000000000000000000000000000000000000000
00000000000000000000000000000000000000000000000000000000000000000000000000000000000000000000000000000000000000000000000000000000000000000000000000000000000000000000000000
00000000000000000000000000000000000000000000000000000000000000000000000000000000000000000000000000000000
>Q42512
MAMSFSGAVLTGMASSFHSGAKQSSFGAVRVGQKTQFVVVSQRKKSLIYAAKGDGNILDDLNEATKKASDFVTDKTKEALADGEKAKDYVVEKNSETADTLGKEAEKAAAYVEEKGKEAANKAAEFAEGKAGEAKDATK
XXXXXXXXXXXXXXXXXXXXXXXXXXXXXXXXXXXXXXXXXX1111111111111111111111111111111111111111111111111111111111111111111111111111111111111111111111111
XXXXXXXXXXXXXXXXXXXXXXXXXXXXXXXXXXXXXXXXXX1111111111111111111111111111111111111111111111111111111111111111111111111111111111111111111111111
XXXXXXXXXXXXXXXXXXXXXXXXXXXXXXXXXXXXXXXXXX0000000000000000000000000000000000000000000000000000000000000000000000000000000000000000000000000
>Q9SIN5
MAMSLSGAVLSGMGSSFHNVGAKQSGVGTVRVGRKSELVVVAQRKKSLIYAVKSDGNILDDLNEATKKASDFVTDKTKEALADGEKTKDYIVEKTIEANETATEEAKKALDYVTEKGKEAGNKAAEFVEGKAEEAKNATKS
XXXXXXXXXXXXXXXXXXXXXXXXXXXXXXXXXXXXXXXXX11111111111111111111111111111111111111111111111111111111111111111111111111111111111111111111111111XX
XXXXXXXXXXXXXXXXXXXXXXXXXXXXXXXXXXXXXXXXX11111111111111111111111111111111111111111111111111111111111111111111111111111111111111111111111111XX
XXXXXXXXXXXXXXXXXXXXXXXXXXXXXXXXXXXXXXXXX00000000000000000000000000000000000000000000000000000000000000000000000000000000000000000000000000XX
>Q71FK2
MPDTTPVAATSSAPPTAKDAGAKAPSDFSNPNTAPSLSDLKKVKYVSTVTSVATPAEIEALGKIFTAMGLAANETGPAMWDLARAYADVQSSKSAQLIGATPSNPALSRRALAAQFDRINITPRQFCMYFAKVVWNILLDSNIPPANWAKLGYQEDTKFAAFDFFD
GVTNPASLQPADGLIRQPNEKELAAHSVAKYGALARQKISTGNYITTLGEVTRGHMGGANTMYAIDPAPPEL
11111111111111111110000000000000000000000000000000000000000000000000000000000000000000000000000000000000000000000000000000000000000000000000000000000000000000000000000000
0000000000000000000000000000000000000000000000000000000000000
00000000000000000000000000000000000000000000000000000000000000000000000000000000000000000000000000000000000000000000000000000000000000000000000000000000000000000000000000
0000000000000000000000000000000000000000000000000000000000000
00000000000000000000000000000000000000000000000000000000000000000000000000000000000000000000000000000000000000000000000000000000000000000000000000000000000000000000000000
0000000000000000000000000000000000000000000000000000000000000
>Q9Y6C2
MAPRTLWSCYLCCLLTAAAGAASYPPRGFSLYTGSSGALSPGGPQAQIAPRPASRHRNWCAYVVTRTVSCVLEDGVETYVKYQPCAWGQPQCPQSIMYRRFLRPRYRVAYKTVTDMEWRCCQGYGGDDCAESPAPALGPASSTPRPLARPARPNLSGSSAGSPL
SGLGGEGPGESEKVQQLEEQVQSLTKELQGLRGVLQGLSGRLAEDVQRAVETAFNGRQQPADAAARPGVHETLNEIQHQLQLLDTRVSTHDQELGHLNNHHGGSSSSGGSRAPAPASAPPGPSEELLRQLEQRLQESCSVCLAGLDGFRRQQQEDRERLRAME
KLLASVEERQRHLAGLAVGRRPPQECCSPELGRRLAELERRLDVVAGSVTVLSGRRGTELGGAAGQGGHPPGYTSLASRLSRLEDRFNSTLGPSEEQEESWPGAPGGLSHWLPAARGRLEQLGGLLANVSGELGGRLDLLEEQVAGAMQACGQLCSGAPGEQDS
QVSEILSALERRVLDSEGQLRLVGSGLHTVEAAGEARQATLEGLQEVVGRLQDRVDAQDETAAEFTLRLNLTAARLGQLEGLLQAHGDEGCGACGGVQEELGRLRDGVERCSCPLLPPRGPGAGPGVGGPSRGPLDGFSVFGGSSGSALQALQGELSEVILSFSSLN
DSLNELQTTVEGQGADLADLGATKDRIISEINRLQQEATEHATESEERFRGLEEGQAQAGQCPSLEGRLGRLEGVCERLDTVAGGLQGLREGLSRHVAGLWAGLRETNTTSQMQAALLEKLVGGQAGLGRRLGALNSSLQLLEDRLHQLSLKDLTGPAGEAGPPGP
PGLQGPPGPAGPPGSPGKDGQEGPIGPPGPQGEQGVEGAPAAPVPQVAFSAALSLPRSEPGTVPFDRVLLNDGGYYDPETGVFTAPLAGRYLLSAVLTGHRHEKVEAVLSRSNQGVARVDSGGYEPEGLENKPVAESQPSPGTLGVFSLILPLQAGDTVCVDLVM
GQLAHSEEPLTIFSGALLYGDPELEHA
00000000000000000000000000000000000000000000000000000000000000000000000000000000000000000000000000000000000000000000000000000000000000000000000000000000000000000000000000
00000000000000000000000000000000000000000000000000000000000000000000000000000000000000000000000000000000000000000000000000000000000000000000000000000000000000000000000000
00000000000000000000000000000000000000000000000000000000000000000000000000000000000000000000000000000000000000000000000000000000000000000000000000000000000000000000000000
00000000000000000000000000000000000000000000000000000000000000000000000000000000000000000000000000000000000000000000000000000000000000000000000000000000000000000000000000
00000000000000000000000000000000000000000000000000000000000000000000000000000000000000000000000000000000000000000000000000000000000000000000000000000000000000000000000000
00000000000000000000000000000000000000000000000000000000000000000000000000000000000000000000000000000000000000000000000000000000000000000000000000000000000000000000000000
000000000000000000000000000000000000000000000000111111111111111111111111XXXXXXXXXXXXXXXXXXXXXXXXXXXXXXXXXXXXXXXXXXXXXXXXXXXXXXXXXXXXXXX
00000000000000000000000000000000000000000000000000000000000000000000000000000000000000000000000000000000000000000000000000000000000000000000000000000000000000000000000000
00000000000000000000000000000000000000000000000000000000000000000000000000000000000000000000000000000000000000000000000000000000000000000000000000000000000000000000000000
00000000000000000000000000000000000000000000000000000000000000000000000000000000000000000000000000000000000000000000000000000000000000000000000000000000000000000000000000
00000000000000000000000000000000000000000000000000000000000000000000000000000000000000000000000000000000000000000000000000000000000000000000000000000000000000000000000000
00000000000000000000000000000000000000000000000000000000000000000000000000000000000000000000000000000000000000000000000000000000000000000000000000000000000000000000000000
00000000000000000000000000000000000000000000000000000000000000000000000000000000000000000000000000000000000000000000000000000000000000000000000000000000000000000000000000
000000000000000000000000000000000000000000000000111111111111111111111111XXXXXXXXXXXXXXXXXXXXXXXXXXXXXXXXXXXXXXXXXXXXXXXXXXXXXXXXXXXXXXX
00000000000000000000000000000000000000000000000000000000000000000000000000000000000000000000000000000000000000000000000000000000000000000000000000000000000000000000000000
00000000000000000000000000000000000000000000000000000000000000000000000000000000000000000000000000000000000000000000000000000000000000000000000000000000000000000000000000
00000000000000000000000000000000000000000000000000000000000000000000000000000000000000000000000000000000000000000000000000000000000000000000000000000000000000000000000000
00000000000000000000000000000000000000000000000000000000000000000000000000000000000000000000000000000000000000000000000000000000000000000000000000000000000000000000000000
00000000000000000000000000000000000000000000000000000000000000000000000000000000000000000000000000000000000000000000000000000000000000000000000000000000000000000000000000
00000000000000000000000000000000000000000000000000000000000000000000000000000000000000000000000000000000000000000000000000000000000000000000000000000000000000000000000000
000000000000000000000000000000000000000000000000000000000000000000000000XXXXXXXXXXXXXXXXXXXXXXXXXXXXXXXXXXXXXXXXXXXXXXXXXXXXXXXXXXXXXXX
>P19599
MKVIKTLSIINFFIFVTFNIKNESKYSNTFINNAYNMSIRRSMANEGSNTNSVGANAPNADTIASGSQRSTNSASTSTTNNGESQTTTPTAADTIASGSQRSTNSASTSTTNNGESQTTTPTAADTPTATESISPSPPITTTESSKFWQCTNKTDGKGEESEKQNELNEST
EEGPKAPQEPQTAENENPAAPENKGTGQHGHMHGSRNNHPQNTSDSQKECTDGNKENCGAATSLLSNSSNIASINKFVVLISATLVLSFAIFI
XXXXXXXXXXXXXXXXXXX1111111111111111111111111111111111111111111111111111111111111111111111111111111111111111111111111111111111111111111111111111111111111111111111111111111
11111111111111111111111111111111111111111111111111111111111111XXXXXXXXXXXXXXXXXXXXXXXXX
XXXXXXXXXXXXXXXXXXX0000000000000000000000000000000000000000000000000000000000000000000000000000000000000000000000000000000000000000000000000000000000000000000000000000000
00000000000000000000000000000000000000000000000000000000000000XXXXXXXXXXXXXXXXXXXXXXXXX
XXXXXXXXXXXXXXXXXXX0000000000000000000000000000000000000000000000000000000000000000000000000000000000000000000000000000000000000000000000000000000000000000000000000000000
00000000000000000000000000000000000000000000000000000000000000XXXXXXXXXXXXXXXXXXXXXXXXX
>Q9UBB5
MRAHPGGGRCCPEQEEGESAAGGSGAGGDSAIEQGGQGSALAPSPVSGVRREGARGGGRGRGRWKQAGRGGGVCGRGRGRGRGRGRGRGRGRGRPPSGGSGLGGDGGGCGGGGSGGGGAPRREPVPFPSGSAGPGPRGPRATESGKRMDCPALP
PGWKKEEVIRKSGLSAGKSDVYYFSPSGKKFRSKPQLARYLGNTVDLSSFDFRTGKMMPSKLQKNKQRLRNDPLNQNKGKPDLNTTLPIRQTASIFKQPVTKVTNHPSNKVKSDPQRMNEQPRQLFWEKRLQGLSASDVTEQIIKTMELPKGLQGVGPGSNDETL
LSAVASALHTSSAPITGQVSAAVEKNPAVWLNTSQPLCKAFIVTDEDIRKQEERVQQVRKKLEEALMADILSRAADTEEMDIEMDSGDEA
XXXXXXXXXXXXXXXXXXXXXXXXXXXXXXXXXXXXXXXXXXXXXXXXXXXXXXXXXXXXXXXXXXXXXXXXXXXXXXXXXXXXXXXXXXXXXXXXXXXXXXXXXXXXXXXXXXXXXXXXXXXXXXXXXXXXXXXXXXXXXXXXXXXXXXXXXXXXXXXXXXXX
XXXXXXXXXXXXXXXXXXXXXXXXXXXXXXXXXXXXXXXXXXXXXXXXXXXXXXXXXXXXXXXXX11111111111111111111111111111111111111111111111111111111111111111111111111111111111111111111111111111111111111
111111111XXXXXXXXXXXXXXXXXXXXXXXXXXXXXXXXXXXXXXXXXXXXXXXXXXXXXXX
XXXXXXXXXXXXXXXXXXXXXXXXXXXXXXXXXXXXXXXXXXXXXXXXXXXXXXXXXXXXXXXXXXXXXXXXXXXXXXXXXXXXXXXXXXXXXXXXXXXXXXXXXXXXXXXXXXXXXXXXXXXXXXXXXXXXXXXXXXXXXXXXXXXXXXXXXXXXXXXXXXXXXXXX
XXXXXXXXXXXXXXXXXXXXXXXXXXXXXXXXXXXXXXXXXXXXXXXXXXXXXXXXXXXXXXXXX11111111111111111111111111111111111111111111111111111111111111111111111111111111111111111111111111111111111111
111111111XXXXXXXXXXXXXXXXXXXXXXXXXXXXXXXXXXXXXXXXXXXXXXXXXXXXXXX
XXXXXXXXXXXXXXXXXXXXXXXXXXXXXXXXXXXXXXXXXXXXXXXXXXXXXXXXXXXXXXXXXXXXXXXXXXXXXXXXXXXXXXXXXXXXXXXXXXXXXXXXXXXXXXXXXXXXXXXXXXXXXXXXXXXXXXXXXXXXXXXXXXXXXXXXXXXXXXXXXXXXXXXXX
XXXXXXXXXXXXXXXXXXXXXXXXXXXXXXXXXXXXXXXXXXXXXXXXXXXXXXXXXXXXXXXXX00000000000000000000000000000000000000000000000000000000000000000000000000000000000000000000000000000000000000
000000000XXXXXXXXXXXXXXXXXXXXXXXXXXXXXXXXXXXXXXXXXXXXXXXXXXXXXXX
>Q6MRH6
MKRLLVLSILLTLGFSFAGTASADENANRPVNPGEDPNEAFRSTPFEATTSALGDCRECIAYRTGATTGKGSRRHDDTVSREIKGSSATPGGSEKAGTGRQ
11111111111111111111111111111111111111111111111111111111111111111111111111111111111111111111111111111
11111111111111111111111111111111111111111111111111111111111111111111111111111111111111111111111111111
00000000000000000000000000000000000000000000000000000000000000000000000000000000000000000000000000000
>P42568
MASSCAVQVKLELGHRAQVRKKPTVEGFTHDWMVFVRGPEHSNIQHFVEKVVFHLHESFPRPKRVCKDPPYKVEESGYAGFILPIEVYFKNKEEPRKVRFDYDLFLHLEGHPPVNHLRCEKLTFNNPTEDFRRKLLKAGGDPNRSIHTSSSSSSSSSSSSSSSSSSSSSS
SSSSSSSSSSSSSSSSSSSTSFSKPHKLMKEHKEKPSKDSREHKSAFKEPSRDHNKSSKESSKKPKENKPLKEEKIVPKMAFKEPKPMSKEPKPDSNLLTITSGQDKKAPSKRPPISDSEELSAKKRKKSSSEALFKSFSSAPPLILTCSADKKQIKDKSHVKMGKVKIESETSEK
KKSTLPPFDDIVDPNDSDVEENISSKSDSEQPSPASSSSSSSSSFTPSQTRQQGPLRSIMKDLHSDDNEEESDEVEDNDNDSEMERPVNRGGSRSRRVSLSDGSDSESSSASSPLHHEPPPPLLKTNNNQILEVKSPIKQSKSDKQIKNGECDKAYLDELVELHRRLMTL
RERHILQQIVNLIEETGHFHITNTTFDFDLCSLDKTTVRKLQSYLETSGTS
00000000000000000000000000000000000000000000000000000000000000000000000000000000000000000000000000000000000000000000000000000000000000000000000000000000000000000000000000
00000000000000000000000000000000000000000000000000000000000000000000000000000000000000000000000000000000000000000000000000000000000000000000000000000000000000000000000000
00000000000000000000000000000000000000000000000000000000000000000000000000000000000000000000000000000000000000000000000000000000000000000111111111111111111111111111111111111111
1111111111111111111111111111111111111111
00000000000000000000000000000000000000000000000000000000000000000000000000000000000000000000000000000000000000000000000000000000000000000000000000000000000000000000000000
00000000000000000000000000000000000000000000000000000000000000000000000000000000000000000000000000000000000000000000000000000000000000000000000000000000000000000000000000
00000000000000000000000000000000000000000000000000000000000000000000000000000000000000000000000000000000000000000000000000000000000000000111111111111111111111111111111111111111
1111111111111111111111111111111111111111
00000000000000000000000000000000000000000000000000000000000000000000000000000000000000000000000000000000000000000000000000000000000000000000000000000000000000000000000000
00000000000000000000000000000000000000000000000000000000000000000000000000000000000000000000000000000000000000000000000000000000000000000000000000000000000000000000000000

00000000000000000000000000000000000000000000000000000000000000000000000000000000000000000000000000000000000000000000000000000000000000000000000000000000000000000000000000000000
0000000000000000000000000000000000000000
>O23764
MESQLHRPTEQEVMEGQTADHGEKKSMLAKVKEKAKKLKGSINKKHGSSQDDDADNDEEINTSPAVHGAPGTSPPPPTQGGEYGGLSERDVNIPHPLASTQANLDKPADVTDASRELQVPPPVPETTPEVSDKGLTEDLGSNAGQGVKESDVDSLTQGLKGVN
YGGDDSNPLSGQEHQTISDEPKSLPGQGNDLPQSHPSSEDEPKKFDANDQPQSMPQDTITGKLSSVPAVIIDRAAAAKNVVASKLGYGGSQAQESAADAGAAQQKKPLTETAAEYKNLVAEKLTPVYEKVAGAGSTVTSKVWGSGGTTAGEQTQGGEGVVDGG
GAASNKGVFTKDYLSEKLKPGDEDKALSQAIMEKLQLSKKPAVEGGAGDETKASESSPGVVGTIKGAVGSLIGGGNKSSGAESAAAADEQTQALGE
11111111111111111111111111111111111111111111111111111111111111111111111111111111111111111111111111111111111111111111111111111111111111111111111111111111111111111111111111111111
11111111111111111111111111111111111111111111111111111111111111111111111111111111111111111111111111111111111111111111111111111111111111111111111111111111111111111111111111111111
1111111111111111111111111111111111111111111111111111111111111111111111
11111111111111111111111111111111111111111111111111111111111111111111111111111111111111111111111111111111111111111111111111111111111111111111111111111111111111111111111111111111
11111111111111111111111111111111111111111111111111111111111111111111111111111111111111111111111111111111111111111111111111111111111111111111111111111111111111111111111111111111
1111111111111111111111111111111111111111111111111111111111111111111111
00000000000000000000000000000000000000000000000000000000000000000000000000000000000000000000000000000000000000000000000000000000000000000000000000000000000000000000000000000000
00000000000000000000000000000000000000000000000000000000000000000000000000000000000000000000000000000000000000000000000000000000000000000000000000000000000000000000000000000000
0000000000000000000000000000000000000000000000000000000000000000000000
>Q7YUB9
MPPIATRRGQYEPKVQQAKLSPDTIPLNPADKTKDPLARADSLHHHVESDSQEDDKAAEEPPLSRKRWQNRTFRRKGRRQAPYKHK
11111111111111111111111111111111111111111111111111111111111111111111111111111111111111
11111111111111111111111111111111111111111111111111111111111111111111111111111111111111
11111111111111111111111111111111111111111111111111111111111111111111111111111111111111
>Q02629
MFGNNRPMFGGSNLSFGSNTSSFGGQQSQQPNSLFGNSNNNNNSTSNNAQSGFGGFTSAAGSNSNSLFGNNNTQNNGAFGQSMGATQNSPFGSLNSSNASNGNTFGGSSSMGSFGGNTNNAFNNNSNSTNSPFGFNKPNTGGTLFGSQNNNSAGTSSL
FGGQSTSTTGTFGNTGSSFGTGLNGNGSNIFGAGNNSQSNTTGSLFGNQQSSAFGTNNQQGSLFGQQSQNTNNAFGNQNQLGGSSFGSKPVGSGSLFGQSNNTLGNTTNNRNGLFGQMNSSNQGSSNSGLFGQNSMNSSTQGVFGQNNNQMQINGNN
NNSLFGKANTFSNSASGGLFGQNNQQQGSGLFGQNSQTSGSSGLFGQNNQKQPNTFTQSNTGIGLFGQNNNQQQQSTGLFGAKPAGTTGSLFGGNSSTQPNSLFGTTNVPTSNTQSQQGNSLFGATKLTNMPFGGNPTANQSGSGNSLFGTKPASTTGSLF
GNNTASTTVPSTNGLFGNNANNSTSTTNTGLFGAKPDSQSKPALGGGLFGNSNSNSSTIGQNKPVFGGTTQNTGLFGATGTNSSAVGSTGKLFGQNNNTLNVGTQNVPPVNNTTQNALLGTTAVPSLQQAPVTNEQLFSKISIPNSITNPVKATTSKVNADMKR
NSSLTSAYRLAPKPLFAPSSNGDAKFQKWGKTLERSDRGSSTSNSITDPESSYLNSNDLLFDPDRRYLKHLVIKNNKNLNVINHNDDEASKVKLVTFTTESASKDDQASSSIAASKLTEKAHSPQTDLKDDHDESTPDPQSKSPNGSTSIPMIENEKISSKVPGLLSNDVT
FFKNNYYISPSIETLGNKSLIELRKINNLVIGHRNYGKVEFLEPVDLLNTPLDTLCGDLVTFGPKSCSIYENCSIKPEKGEGINVRCRVTLYSCFPIDKETRKPIKNITHPLLKRSIAKLKENPVYKFESYDPVTGTYSYTIDHPVLT
11111111111111111111111111111111111111111111111111111111111111111111111111111111111111111111111111111111111111111111111111111111111111111111111111111111111111111111111111111111
11111111111111111111111111111111111111111111111111111111111111111111111111111111111111111111111111111111111111111111111111111111111111111111111111111111111111111111111111111111
11111111111111111111111111111111111111111111111111111111111111111111111111111111111111111111111111111111111111111111111111111111111111111111111111111111111111111111111111111111
11111111111111111111111111111111111111111111111111111111111111111111111111111111111111111111111111111111111111111111111000000000000000000000000000000000000000000000000000000000
00000000000000000000000000000000000000000000000000000000000000000000000000000000000000000000000000000000000000000000000000000000000000000000000000000000000000000000000000000000
0000000000000000000000000000000000000000000000000000000000000000000000000000000
11111111111111111111111111111111111111111111111111111111111111111111111111111111111111111111111111111111111111111111111111111111111111111111111111111111111111111111111111111111
11111111111111111111111111111111111111111111111111111111111111111111111111111111111111111111111111111111111111111111111111111111111111111111111111111111111111111111111111111111
11111111111111111111111111111111111111111111111111111111111111111111111111111111111111111111111111111111111111111111111111111111111111111111111111111111111111111111111111111111
11111111111111111111111111111111111111111111111111111111111111111111111111111111111111111111111111111111111111111111111000000000000000000000000000000000000000000000000000000000
00000000000000000000000000000000000000000000000000000000000000000000000000000000000000000000000000000000000000000000000000000000000000000000000000000000000000000000000000000000
0000000000000000000000000000000000000000000000000000000000000000000000000000000
00000000000000000000000000000000000000000000000000000000000000000000000000000000000000000000000000000000000000000000000000000000000000000000000000000000000000000000000000000000
00000000000000000000000000000000000000000000000000000000000000000000000000000000000000000000000000000000000000000000000000000000000000000000000000000000000000000000000000000000
00000000000000000000000000000000000000000000000000000000000000000000000000000000000000000000000000000000000000000000000000000000000000000000000000000000000000000000000000000000
00000000000000000000000000000000000000000000000000000000000000000000000000000000000000000000000000000000000000000000000000000000000000000000000000000000000000000000000000000000
00000000000000000000000000000000000000000000000000000000000000000000000000000000000000000000000000000000000000000000000000000000000000000000000000000000000000000000000000000000
0000000000000000000000000000000000000000000000000000000000000000000000000000000
>P14907
MNFNTPQQNKTPFSFGTANNNSNTTNQNSSTGAGAFGTGQSTFGFNNSAPNNTNNANSSITPAFGSNNTGNTAFGNSNPTSNVFGSNNSTTNTFGSNSAGTSLFGSSSAQQTKSNGTAGGNTFGSSSLFNNSTNSNTTKPAFGGLNFGGGNNTTPSSTGNAN
TSNNLFGATANANKPAFSFGATTNDDKKTEPDKPAFSFNSSVGNKTDAQAPTTGFSFGSQLGGNKTVNEAAKPSLSFGSGSAGANPAGASQPEPTTNEPAKPALSFGTATSDNKTTNTTPSFSFGAKSDENKAGATSKPAFSFGAKPEEKKDDNSSKPAFSFGAKS
NEDKQDGTAKPAFSFGAKPAEKNNNETSKPAFSFGAKSDEKKDGDASKPAFSFGAKPDENKASATSKPAFSFGAKPEEKKDDNSSKPAFSFGAKSNEDKQDGTAKPAFSFGAKPAEKNNNETSKPAFSFGAKSDEKKDGDASKPAFSFGAKSDEKKDSDSSKPAFSF
GTKSNEKKDSGSSKPAFSFGAKPDEKKNDEVSKPAFSFGAKANEKKESDESKSAFSFGSKPTGKEEGDGAKAAISFGAKPEEQKSSDTSKPAFTFGAQKDNEKKTEESSTGKSTADVKSSDSLKLNSKPVELKPVSLDNKTLDDLVTKWTNQLTESASHFEQYTKKINS
WDQVLVKGGEQISQLYSDAVMAEHSQNKIDQSLQYIERQQDELENFLDNFETKTEALLSDVVSTSSGAAANNNDQKRQQAYKTAQTLDENLNSLSSNLSSLIVEINNVSNTFNKTTNIDINNEDENIQLIKILNSHFDALRSLDDNSTSLEKQINSIKK
11111111111111111111111111111111111111111111111111111111111111111111111111111111111111111111111111111111111111111111111111111111111111111111111111111111111111111111111111111111
11111111111111111111111111111111111111111111111111111111111111111111111111111111111111111111111111111111111111111111111111111111111111111111111111111111111111111111111111111111
11111111111111111111111111111111111111111111111111111111111111111111111111111111111111111111111111111111111111111111111111111111111111111111111111111111111111111111111111111111
11111111111111111111111111111111111111111111111111111111111111111111111111111111111111111110000000000000000000000000000000000000000000000000000000000000000000000000000000000000
00000000000000000000000000000000000000000000000000000000000000000000000000000000000000000000000000000000000000000000000
11111111111111111111111111111111111111111111111111111111111111111111111111111111111111111111111111111111111111111111111111111111111111111111111111111111111111111111111111111111
11111111111111111111111111111111111111111111111111111111111111111111111111111111111111111111111111111111111111111111111111111111111111111111111111111111111111111111111111111111
11111111111111111111111111111111111111111111111111111111111111111111111111111111111111111111111111111111111111111111111111111111111111111111111111111111111111111111111111111111
11111111111111111111111111111111111111111111111111111111111111111111111111111111111111111110000000000000000000000000000000000000000000000000000000000000000000000000000000000000
00000000000000000000000000000000000000000000000000000000000000000000000000000000000000000000000000000000000000000000000
00000000000000000000000000000000000000000000000000000000000000000000000000000000000000000000000000000000000000000000000000000000000000000000000000000000000000000000000000000000
00000000000000000000000000000000000000000000000000000000000000000000000000000000000000000000000000000000000000000000000000000000000000000000000000000000000000000000000000000000
00000000000000000000000000000000000000000000000000000000000000000000000000000000000000000000000000000000000000000000000000000000000000000000000000000000000000000000000000000000
00000000000000000000000000000000000000000000000000000000000000000000000000000000000000000000000000000000000000000000000000000000000000000000000000000000000000000000000000000000
00000000000000000000000000000000000000000000000000000000000000000000000000000000000000000000000000000000000000000000000
>P40477
MSSLKDEVPTETSEDFGFKFLGQKQILPSFNEKLPFASLQNLDISNSKSLFVAASGSKAVVGELQLLRDHITSDSTPLTFKWEKEIPDVIFVCFHGDQVLVSTRNALYSLDLEELSEFRTVTSFEKPVFQLKNVNNTLVILNSVNDLSALDLRTKSTKQLAQNVTSFDVTNS
QLAVLLKDRSFQSFAWRNGEMEKQFEFSLPSELEELPVEEYSPLSVTILSPQDFLAVFGNVISETDDEVSYDQKMYIIKHIDGSASFQETFDITPPFGQIVRFPYMYKVTLSGLIEPDANVNVLASSCSSEVSIWDSKQVIEPSQDSERAVLPISEETDKDTNPIGVAVDVV
TSGTILEPCSGVDTIERLPLVYILNNEGSLQIVGLFHVAAIKSGHYSINLESLEHEKSLSPTSEKIPIAGQEQEEKKKNNESSKALSENPFTSANTSGFTFLKTQPAAANSLQSQSSSTFGAPSFGSSAFKIDLPSVSSTSTGVASSEQDATDPASAKPVFGKPAFGAIAKEPSTS
EYAFGKPSFGAPSFGSGKSSVESPASGSAFGKPSFGTPSFGSGNSSVEPPASGSAFGKPSFGTPSFGSGNSSAEPPASGSAFGKPSFGTSAFGTASSNETNSGSIFGKAAFGSSSFAPANNELFGSNFTISKPTVDSPKEVDSTSPFPSSGDQSEDESKSDVDSSSTPFGT
KPNTSTKPKTNAFDFGSSSFGSGFSKALESVGSDTTFKFGTQASPFSSQLGNKSPFSSFTKDDTENGSLSKGSTSEINDDNEEHESNGPNVSGNDLTDSTVEQTSSTRLPETPSDEDGEVVEEEAQKSPIGKLTETIKKSANIDMAGLKNPVFGNHVKAKSESPFSAFAT
NITKPSSTTPAFSFGNSTMNKSNTSTVSPMEEADTKETSEKGPITLKSVENPFLPAKEERTGESSKKDHNDDPKDGYVSGSEISVRTSESAFDTTANEEIPKSQDVNNHEKSETDPKYSQHAVVDHDNKSKEMNETSKNNERSGQPNHGVQGDGIALKKDNEKENFD
SNMAIKQFEDHQSSEEDASEKDSRQSSEVKESDDNMSLNSDRDESISESYDKLEDINTDELPHGGEAFKAREVSASADFDVQTSLEDNYAESGIQTDLSESSKENEVQTDAIPVKHNSTQTVKKEAVDNGLQTEPVETCNFSVQTFEGDENYLAEQCKPKQLKEYYTS
AKVSNIPFVSQNSTLRLIESTFQTVEAEFTVLMENIRNMDTFFTDQSSIPLVKRTVRSINNLYTWRIPEAEILLNIQNNIKCEQMQITNANIQDLKEKVTDYVRKDIAQITEDVANAKEEYLFLMHFDDASSGYVKDLSTHQFRMQKTLRQKLFDVSAKINHTEELLNILKL
FTVKNKRLDDNPLVAKLAKESLARDGLLKEIKLLREQVSRLQLEEKGKKASSFDASSSITKDMKGFKVVEVGLAMNTKKQIGDFFKNLNMAK
00000000000000000000000000000000000000000000000000000000000000000000000000000000000000000000000000000000000000000000000000000000000000000000000000000000000000000000000000000000
00000000000000000000000000000000000000000000000000000000000000000000000000000000000000000000000000000000000000000000000000000000000000000000000000000000000000000000000000000000
00000000000000000000000000000000000000000000000000000000000000000000000000000000000011111111111111111111111111111111111111111111111111111111111111111111111111111111111111111111
11111111111111111111111111111111111111111111111111111111111111111111111111111111111111111111111111111111111111111111111111111111111111111111111111111111111111111111111111111111
11111111111111111111111111111111111111111111111111111111111111111111111111111111111111111111111111111111111111111111111111111111111111111111111111111111111111111111111111111111
10000000000000000000000000000000000000000000000000000000000000000000000000000000000000000000000000000000000000000000000000000000000000000000000000000000000000000000000000000000
00000000000000000000000000000000000000000000000000000000000000000000000000000000000000000000000000000000000000000000000000000000000000000000000000000000000000000000000000000000
00000000000000000000000000000000000000000000000000000000000000000000000000000000000000000000000000000000000000000000000000000000000000000000000000000000000000000000000000000000
0000000000000000000000000000000000000000000000000000
00000000000000000000000000000000000000000000000000000000000000000000000000000000000000000000000000000000000000000000000000000000000000000000000000000000000000000000000000000000
00000000000000000000000000000000000000000000000000000000000000000000000000000000000000000000000000000000000000000000000000000000000000000000000000000000000000000000000000000000
00000000000000000000000000000000000000000000000000000000000000000000000000000000000011111111111111111111111111111111111111111111111111111111111111111111111111111111111111111111
11111111111111111111111111111111111111111111111111111111111111111111111111111111111111111111111111111111111111111111111111111111111111111111111111111111111111111111111111111111
11111111111111111111111111111111111111111111111111111111111111111111111111111111111111111111111111111111111111111111111111111111111111111111111111111111111111111111111111111111
10000000000000000000000000000000000000000000000000000000000000000000000000000000000000000000000000000000000000000000000000000000000000000000000000000000000000000000000000000000
00000000000000000000000000000000000000000000000000000000000000000000000000000000000000000000000000000000000000000000000000000000000000000000000000000000000000000000000000000000
00000000000000000000000000000000000000000000000000000000000000000000000000000000000000000000000000000000000000000000000000000000000000000000000000000000000000000000000000000000
0000000000000000000000000000000000000000000000000000
00000000000000000000000000000000000000000000000000000000000000000000000000000000000000000000000000000000000000000000000000000000000000000000000000000000000000000000000000000000
00000000000000000000000000000000000000000000000000000000000000000000000000000000000000000000000000000000000000000000000000000000000000000000000000000000000000000000000000000000
00000000000000000000000000000000000000000000000000000000000000000000000000000000000000000000000000000000000000000000000000000000000000000000000000000000000000000000000000000000
00000000000000000000000000000000000000000000000000000000000000000000000000000000000000000000000000000000000000000000000000000000000000000000000000000000000000000000000000000000
00000000000000000000000000000000000000000000000000000000000000000000000000000000000000000000000000000000000000000000000000000000000000000000000000000000000000000000000000000000
00000000000000000000000000000000000000000000000000000000000000000000000000000000000000000000000000000000000000000000000000000000000000000000000000000000000000000000000000000000
00000000000000000000000000000000000000000000000000000000000000000000000000000000000000000000000000000000000000000000000000000000000000000000000000000000000000000000000000000000
00000000000000000000000000000000000000000000000000000000000000000000000000000000000000000000000000000000000000000000000000000000000000000000000000000000000000000000000000000000
0000000000000000000000000000000000000000000000000000
>P40517
MSETNGGNAARENSEVKQTAVENPIDKLDGTPKRPREKDQDEQAEETSDKSEAPNKNDEEKKEEGKKDQEPSHKKIKVDDGKTVESGIVEDDKKEDKFVFGAASKFGTGFGVAKKDTKDGDATTSTESLPASDSKTKKPFAFGSGLSFGSGFNILKNKTENNSESEK
KATDVDKDKVHSGSEQLANASEDTKDKPKPLKLQKQEVKSGEESEECIYQVNAKLYQLSNIKEGWKERGVGIIKINKSKDDVEKTRIVMRSRGILKVILNIQLVKGFTVQKGFTGSLQSEKFIRLLAVDDNGDPAQYAIKTGKKETTDELYNIIVKSVPK
11111111111111111111111111111111111111111111111111111111111111111111111111111111111111111111111111111111111111111111111111111111111111111111111111111111111111111111111111111111
1111111111111111111111111110000000000000000000000000000000000000000000000000000000000000000000000000000000000000000000000000000000000000000000000000
11111111111111111111111111111111111111111111111111111111111111111111111111111111111111111111111111111111111111111111111111111111111111111111111111111111111111111111111111111111
1111111111111111111111111110000000000000000000000000000000000000000000000000000000000000000000000000000000000000000000000000000000000000000000000000
00000000000000000000000000000000000000000000000000000000000000000000000000000000000000000000000000000000000000000000000000000000000000000000000000000000000000000000000000000000
0000000000000000000000000000000000000000000000000000000000000000000000000000000000000000000000000000000000000000000000000000000000000000000000000000000
>Q9XES8
MAKSKEDITYATSQARLSEDEAVRVAYEHGSPLEGGKIADSQPVDLFSSAHNMPKSGQTTMDSNTSDQSQMQRDTQEGGSKEFTTGAPG
11111111111111111111111111111111111111111111111111111111111111111111111111111111111111111
11111111111111111111111111111111111111111111111111111111111111111111111111111111111111111
00000000000000000000000000000000000000000000000000000000000000000000000000000000000000000
>D0VWX1
GCEGKQCGLFRSCGGGCRCWPTVTPGVGICSSS
XXXXXX1111111XXXXXXXXXXXXXXXXXXXX
XXXXXX0000000XXXXXXXXXXXXXXXXXXXX
XXXXXX0000000XXXXXXXXXXXXXXXXXXXX
>B7T1D7
MNGIRWIASYPKAGNTWVRCMLAAYITGKAPQVWNDIDAESLTLEAMLRFGDLPPAEPMEPVLVKTHLKADVPVLGLYGEATAKVLYLVRNPRDMLLSSMRMASISRDDVEKSRDFARKFIANEGLGWNALGAGGGVGLGSWPENVRSWTESSSDRFPNADV
LTMRYEDLKGDPVARFSEIVEFLDLGGPVDIEDIRRAVAASTLERMRELEKRSEQQGGGSPIRHGDARMMKGGPGGARPQFVGEGRYDQSLSFLGEDIESDYQELLHGDSGFALYAKQYGYAG
00000000000000000000000000000000000000000000000000000000000000000000000000000000000000000000000000000000000000000000000000111111111110000000000000000000000000000000000000000000
0000000000000000000000000000000000000000111111111111111111111111111111111110000000000000000000000000000000000

00000000000000000000000000000000000000000000000000000000000000000000000000000000000000000000000000000000000000000000000000000000000000000000000000000000000000000000000000000000
0000000000000000000000000000000000000000000000000000000000000000000000000000000000000000000000000000000000000
00000000000000000000000000000000000000000000000000000000000000000000000000000000000000000000000000000000000000000000000000000000000000000000000000000000000000000000000000000000
0000000000000000000000000000000000000000000000000000000000000000000000000000000000000000000000000000000000000
>Q9BP37
MTYMCSILICLVLILCARGAEADDNGNYGNGMASVRTQGNTYDDLASLISYLTRHSFRRPFHECALCYSITDPGERQRCIDMYCSYTN
XXXXXXXXXXXXXXXXXXXXXX111111111111111111111111111111XXX111111111111111111111111111111111
XXXXXXXXXXXXXXXXXXXXXX111111111111111111111111111111XXX000000000000000000000000000000000
XXXXXXXXXXXXXXXXXXXXXX000000000000000000000000000000XXX000000000000000000000000000000000
>Q9BP38
MTWMKMFYLTLAVTAAVLLVTVAAADDDEDASSGLCNQYNQNVTTRPNNKPKMFLRKNINFEIISVHNIWRDPNTVYWCDFSLEEEDGIKHWRHYDFNATHWWVEKGCSGTFVVEECNTKDITNPGPRSTAGKSPMQGTLAAPKPVANWMSIMSRSRFDM
GTWDKEGFNML
XXXXXXXXXXXXXXXXXXX11111111111111111111111111111111111XXXXXXXXXXXXXXXXXXXXXXXXXXXXXXXXXXXXXXXXXXXXXXXXXXXXXXXXXXXXXXXXXXXXXXXXXXXXXXXXXXXXXXXXXXXXXXXXXXXXXXXXXXXXX
XXXXXXXXXXXXXXXXXXX11111111111111111111111111111111111XXXXXXXXXXXXXXXXXXXXXXXXXXXXXXXXXXXXXXXXXXXXXXXXXXXXXXXXXXXXXXXXXXXXXXXXXXXXXXXXXXXXXXXXXXXXXXXXXXXXXXXXXXXXXX
XXXXXXXXXXXXXXXXXXX00000000000000000000000000000000000XXXXXXXXXXXXXXXXXXXXXXXXXXXXXXXXXXXXXXXXXXXXXXXXXXXXXXXXXXXXXXXXXXXXXXXXXXXXXXXXXXXXXXXXXXXXXXXXXXXXXXXXXXXXXX
>Q27934
MSTNQQQQANPSAAVAAPAASSEYLKRTCLICGCHTNQTINIYEPRSGPNIVQLIQAKFKFQPLNEDKFLCFSCNNWLINWHSLQAVNSNEAESQSQSPSHMGNSVLQQERTKLRPVAMVRPTVRVQPQSQPQLQPQVPINPTPAPIVYSKRRASRRSASVSRM
SRVLRQCCVESLRRSPKKRNQQSVFVCLRPQGQKRSNAICKVECVAPRRKPVERLVKDVAATATPTPVLNTQSTPTYQRFPQPSVDGKVVAMFRRLGTTLSREEPAAYSAESNPACSKLPQIMSPLKEAPRWTRDLDDDEILLEFDTAISEVLPTARYQVTHEENKEN
QQAQEMELELEEEEEVDGRAELEVVQEAEAPLEPQSHHKQGNSHQNSHQASIQLAGLRLPMGLSISLV
XXXXXXXXXXXXXXXXXXXXXXXXXXXXXXXXXXXXXXXXXXXXXXXXXXXXXXXXXXXXXXXXXXXXXXXXXXXXXXXXXXXXXXXXXXXXXXXXXXXXXXXXXX111111111111111111111111XXXXXXXXXXXXXXXXXXXXXXXXXXXXXXXXXXXXXXXXXXX
XXXXXXXXXXXXXXXXXXXXXXXXXXXXXXXXXXXXXXXXXXXXXXXXXXXXXXXXXXXXXXXXXXXXXXXXXXXXXXXXXXXXXXXXXXXXXXXXXXXXXXXXXXXXXXXXXXXXXXXXXXXXXXXXXXXXXXXXXXXXXXXXXXXXXXXXXXXXXXXXXXXXXXXX
XXXXXXXXXXXXXXXXXXXXXXXXXXXXXXXXXXXXXXXXXXXXXXXXXXXXXXX
XXXXXXXXXXXXXXXXXXXXXXXXXXXXXXXXXXXXXXXXXXXXXXXXXXXXXXXXXXXXXXXXXXXXXXXXXXXXXXXXXXXXXXXXXXXXXXXXXXXXXXXXXX111111111111111111111111XXXXXXXXXXXXXXXXXXXXXXXXXXXXXXXXXXXXXXXXXXX
XXXXXXXXXXXXXXXXXXXXXXXXXXXXXXXXXXXXXXXXXXXXXXXXXXXXXXXXXXXXXXXXXXXXXXXXXXXXXXXXXXXXXXXXXXXXXXXXXXXXXXXXXXXXXXXXXXXXXXXXXXXXXXXXXXXXXXXXXXXXXXXXXXXXXXXXXXXXXXXXXXXXXXXX
XXXXXXXXXXXXXXXXXXXXXXXXXXXXXXXXXXXXXXXXXXXXXXXXXXXXXXX
XXXXXXXXXXXXXXXXXXXXXXXXXXXXXXXXXXXXXXXXXXXXXXXXXXXXXXXXXXXXXXXXXXXXXXXXXXXXXXXXXXXXXXXXXXXXXXXXXXXXXXXXXX000000000000000000000000XXXXXXXXXXXXXXXXXXXXXXXXXXXXXXXXXXXXXXXXXXX
XXXXXXXXXXXXXXXXXXXXXXXXXXXXXXXXXXXXXXXXXXXXXXXXXXXXXXXXXXXXXXXXXXXXXXXXXXXXXXXXXXXXXXXXXXXXXXXXXXXXXXXXXXXXXXXXXXXXXXXXXXXXXXXXXXXXXXXXXXXXXXXXXXXXXXXXXXXXXXXXXXXXXXXX
XXXXXXXXXXXXXXXXXXXXXXXXXXXXXXXXXXXXXXXXXXXXXXXXXXXXXXX
>Q9Y261
MLGAVKMEGHEPSDWSSYYAEPEGYSSVSNMNAGLGMNGMNTYMSMSAAAMGSGSGNMSAGSMNMSSYVGAGMSPSLAGMSPGAGAMAGMGGSAGAAGVAGMGPHLSPSLSPLGGQAAGAMGGLAPYANMNSMSPMYGQAGLSRARDPKTY
RRSYTHAKPPYSYISLITMAIQQSPNKMLTLSEIYQWIMDLFPFYRQNQQRWQNSIRHSLSFNDCFLKVPRSPDKPGKGSFWTLHPDSGNMFENGCYLRRQKRFKCEKQLALKEAAGAAGSGKKAAAGAQASQAQLGEAAGPASETPAGTESPHSSASPCQEHKR
GGLGELKGTPAAALSPPEPAPSPGQQQQAAAHLLGPPHHPGLPPEAHLKPEHHYAFNHPFSINNLMSSEQQHHHSHHHHQPHKMDLKAYEQVMHYPGYGSPMPGSLAMGPVTNKTGLDASPLAADTSYYQGVYSRPIMNSS
00000000000000000000000000000000000000000000000000000000000000000000000000000000000000000000000000000000000000000000000000000000000000000000000000000000000000000000000000000000
000000000000000000000000000000000000000000000000000000000000000000000000000000000001111111111111111111XXXXXXXXXXXXXXXXXXXXXXXXXXXXXXXXXXXXXXXXXXXXXXXXXXXXXXXXXXXXXXXXXXXXXXXX
XXXXXXXXXXXXXXXXXXXXXXXXXXXXXXXXXXXXXXXXXXXXXXXXXXXXXXXXXXXXXXXXXXXXXXXXXXXXXXXXXXXXXXXXXXXXXXXXXXXXXXXXXXX
00000000000000000000000000000000000000000000000000000000000000000000000000000000000000000000000000000000000000000000000000000000000000000000000000000000000000000000000000000000
00000000000000000000000000000000000000000000000000000000000000000000000000000000000XXXXXXXXXXXXXXXXXXXXXXXXXXXXXXXXXXXXXXXXXXXXXXXXXXXXXXXXXXXXXXXXXXXXXXXXXXXXXXXXXXXXXXXXXXXXXXXXX
XXXXXXXXXXXXXXXXXXXXXXXXXXXXXXXXXXXXXXXXXXXXXXXXXXXXXXXXXXXXXXXXXXXXXXXXXXXXXXXXXXXXXXXXXXXXXXXXXXXXXXXXXXX
00000000000000000000000000000000000000000000000000000000000000000000000000000000000000000000000000000000000000000000000000000000000000000000000000000000000000000000000000000000
000000000000000000000000000000000000000000000000000000000000000000000000000000000001111111111111111111XXXXXXXXXXXXXXXXXXXXXXXXXXXXXXXXXXXXXXXXXXXXXXXXXXXXXXXXXXXXXXXXXXXXXXXX
XXXXXXXXXXXXXXXXXXXXXXXXXXXXXXXXXXXXXXXXXXXXXXXXXXXXXXXXXXXXXXXXXXXXXXXXXXXXXXXXXXXXXXXXXXXXXXXXXXXXXXXXXXX
>O43561
MEEAILVPCVLGLLLLPILAMLMALCVHCHRLPGSYDSTSSDSLYPRGIQFKRPHTVAPWPPAYPPVTSYPPLSQPDLLPIPRSPQPLGGSHRTPSSRRDSDGANSVASYENEGASGIRGAQAGWGVWGPSWTRLTPVSLPPEPACEDADEDEDDYHNPGYLVVLP
DSTPATSTAAPSAPALSTPGIRDSAFSMESIDDYVNVPESGESAEASLDGSREYVNVSQELHPGAAKTEPAALSSQEAEEVEEEGAPDYENLQELN
XXXXXXXXXXXXXXXXXXXXXXXXXXXX1111111111111111111111111111111111111111111111111111111111111111111111111111111111111111111111111111111111111111111111111111111111111111111111111111
111111111111111111111111111111111111111111111111111111111XXXXXXXXXXXXXXXXXXXXXXXXXXXXX
XXXXXXXXXXXXXXXXXXXXXXXXXXXX1111111111111111111111111111111111111111111111111111111111111111111111111111111111111111111111111111111111111111111111111111111111111111111111111111
111111111111111111111111111111111111111111111111111111111XXXXXXXXXXXXXXXXXXXXXXXXXXXXX
XXXXXXXXXXXXXXXXXXXXXXXXXXXX0000000000000000000000000000000000000000000000000000000000000000000000000000000000000000000000000000000000000000000000000000000000000000000000000000
000000000000000000000000000000000000000000000000000000000XXXXXXXXXXXXXXXXXXXXXXXXXXXXX
>Q8VHK2
MGKEQELVQAVKAEDVGTAQRLLQRPRPGKAKLLGSTKKINVNFQDPDGFSALHHAALNGNTELISLLLEAQAAVDIKDNKGMRPLHYAAWQGRKEPMKLVLKAGSAVNVPSDEGHIPLHLAAQHGHYDVSEMLLQHQSNPCIVDNSGKTPLDLACEFGRVGV
VQLLLSSNMCAALLEPRPGDTTDPNGTSPLHLAAKNGHIDIIRLLLQAGIDINRQTKSGTALHEAALCGKTEVVRLLLDSGINAQVRNTYSQTALDIVHQFTTSQASKEIKQLLREASAALQVRATKDYCNNYDLTSLNVKAGDIITVLEQHPDGRWKGCIHDNRTGND
RVGYFPSSLGEAIVKRAGSRTGSEPSPPQGGGSLGPSAPPEEIWVLRKPFAGGDRSGSLSNVAGGRSTGGHALHAGAEGVKLLATVLSQKSVSESSPGDSPVKPPEGSSGAARSQPPAAHAGQVYGEQPPKKLESSSASEGKSAEAVSQWLATFQLQLYAPNFTSA
GYDLPTISRMTPEDLTAIGVTKPGHRKKITAEISGLNIPDCLPEHKPANLAVWLSMIGLAQYYKVLVDNGYENIDFITDITWEDLQEIGITKLGHQKKLMLAVRKLAELQKAEYSKYEGGPLRRKAPQSLEMMAIESPPPSEPAAAECQSPKMTTFQDSELSGELQAALS
GPAEAGAAAAEKSSNHLPATPRTTSRQESSLSGRARHMSSSQELLGDGPQGPGSPMSRSQEYLLDEGPAPGTPPKEVRSSRHGHSVKRASVPPVPGKPRQVLPSGVSHFTPPQTPTKAQPGSPQALGGPHGPATAKVKPTPQLLPPTDRPMSPRSLPQSPTHRGF
AYVLPQPVEGEAGPPAPGPVPPPVPAAVPTLCLPPEADVEPRRPKKRAHSLNRYAASDSEPERDELLVPAAAGPYATVQRRVGRSHSVRAPAGTDKNVNRSQSFAVRPRKKGPPPPPPKRSSSAMASANLADEPSPDVETEDGRLGVRAQRRRASDLAGSVDTGS
AGSVKSIAAMLELSSIGGGGRAIRRPPEGHPTPRPASPDPGRVATVLASVKHKEAIGPDGEVVNRRRTLSGPVTGLLATARRGPGEPAEQSHFMEDGTARQRLRGPAKGEAGVEGPPLARVEASATLKRRIRAKQSQQENVKFILTESDTVKRRPKAKEPDIGPEPPP
PLSVYQNGTATIRRRPASEQAGPPELPPPPPPAEPPPTDLMPLPPLPLPDGSARKPVKPPVSPKPILAQPVSKIQGSPTPASKKVPLPGPGSPEVKRAHGTPPPVSPKPPPPPTAPKPAKALAGLQSSSATPSPVPSPARQPPAALIKPASSPPSQSASPAKPPSPGAPAL
QVPTKPPRAAASVVSGPPVASDCASPGDSARQKLEETSACLAAALQAVEEKIRQEDGQGPRPSSIEEKSTGSILEDIGSMFDDLADQLDAMLE
00000000000000000000000000000000000000000000000000000000000000000000000000000000000000000000000000000000000000000000000000000000000000000000000000000000000000000000000000000000
00000000000000000000000000000000000000000000000000000000000000000000000000000000000000000000000000000000000000000000000000000000000000000000000000000000000000000000000000000000
00000000000000000000000000000000000000000000000000000000000000000000000000000000000000000000000000000000000000000000000000000000000000000000000000000000000000000000000000000000
00000000000000000000000000000000000000000000000000000000000000000000000000000000011111111111111111111111111111111111111111111111111111111111111111111111111111111111111111111111111
11111111111111111111111111111111111111111111111111111111111111111111111111111111111111111111111111111111111111111111111111111111111111111111111111111111111111111111111111111111
11111111111111111111111111111111111111111111111111111111111111111111111111111111111111111111111111111111111111111111111111111111111111111111111111111111111111111111111111111111
11111111111111111111111111111111111111111111111111111111111111111111111111111111111111111111111111111111111111111111111111111111111111111111111111111111111111111111111111111111
11111111111111111111111111111111111111111111111111111111111111111111111111111111111111111111111111111111111111111111111111111111111111111111111111111111111111111111111111111111
11111111111111111111111111111111111111111111111111111111111111111111111111111111111111111111111111111111111111111111111111111111111111111111111111111111111111111111111111111111
1111111111111111111111
00000000000000000000000000000000000000000000000000000000000000000000000000000000000000000000000000000000000000000000000000000000000000000000000000000000000000000000000000000000
00000000000000000000000000000000000000000000000000000000000000000000000000000000000000000000000000000000000000000000000000000000000000000000000000000000000000000000000000000000
00000000000000000000000000000000000000000000000000000000000000000000000000000000000000000000000000000000000000000000000000000000000000000000000000000000000000000000000000000000
00000000000000000000000000000000000000000000000000000000000000000000000000000000011111111111111111111111111111111111111111111111111111111111111111111111111111111111111111111111111
11111111111111111111111111111111111111111111111111111111111111111111111111111111111111111111111111111111111111111111111111111111111111111111111111111111111111111111111111111111
11111111111111111111111111111111111111111111111111111111111111111111111111111111111111111111111111111111111111111111111111111111111111111111111111111111111111111111111111111111
11111111111111111111111111111111111111111111111111111111111111111111111111111111111111111111111111111111111111111111111111111111111111111111111111111111111111111111111111111111
11111111111111111111111111111111111111111111111111111111111111111111111111111111111111111111111111111111111111111111111111111111111111111111111111111111111111111111111111111111
11111111111111111111111111111111111111111111111111111111111111111111111111111111111111111111111111111111111111111111111111111111111111111111111111111111111111111111111111111111
1111111111111111111111
00000000000000000000000000000000000000000000000000000000000000000000000000000000000000000000000000000000000000000000000000000000000000000000000000000000000000000000000000000000
00000000000000000000000000000000000000000000000000000000000000000000000000000000000000000000000000000000000000000000000000000000000000000000000000000000000000000000000000000000
00000000000000000000000000000000000000000000000000000000000000000000000000000000000000000000000000000000000000000000000000000000000000000000000000000000000000000000000000000000
00000000000000000000000000000000000000000000000000000000000000000000000000000000000000000000000000000000000000000000000000000000000000000000000000000000000000000000000000000000
00000000000000000000000000000000000000000000000000000000000000000000000000000000000000000000000000000000000000000000000000000000000000000000000000000000000000000000000000000000
00000000000000000000000000000000000000000000000000000000000000000000000000000000000000000000000000000000000000000000000000000000000000000000000000000000000000000000000000000000
00000000000000000000000000000000000000000000000000000000000000000000000000000000000000000000000000000000000000000000000000000000000000000000000000000000000000000000000000000000
00000000000000000000000000000000000000000000000000000000000000000000000000000000000000000000000000000000000000000000000000000000000000000000000000000000000000000000000000000000
00000000000000000000000000000000000000000000000000000000000000000000000000000000000000000000000000000000000000000000000000000000000000000000000000000000000000000000000000000000
0000000000000000000000
>P40019
MSDEAKEKRELESQKESSHNKSEKSVEPKPKRRRRRNYDDYDAEVAKEETKAKNGLTKSENNGTVEDSESDMDDAKLDALMGNEGEEEEDDLAEIDTSNIITSGRRTRGKVIDYKKTAEELDKKEPSTGSKDDVGYGEKEEDDEDEEDDDFKE
111111111111111111111111111111111111111111111111111111111111111111111111111111111111111111111111111111111111111111111111111111111111111111111111111111111
000000000000000000000000000000000000000000000000000000000000000000000000000000000000000000000000000000000000000000000000000000000000000000000000000000000
000000000000000000000000000000000000000000000000000000000000000000000000000000000000000000000000000000000000000000000000000000000000000000000000000000000
>P38293
MNIVPQDTFKSQVSTDQDKSVLSSAVPSLPDTLRQQEGGAVPLSTQLNDRHPLESTLKNWETTQRQRQMEQYRQIFGIAEPMKRTMEMEIVNRTDFNPLSTNGSIHRDILLNKECSIDWEDVYPGTGLQASTMVGDDVHSKIEKQLGI
1111111111111111111111111111111111111111111111111111111111111111111111111111111111111111111111111111111111111111111111111111111111111111111111111111
0000000000000000000000000000000000000000000000000000000000000000000000000000000000000000000000000000000000000000000000000000000000000000000000000000
0000000000000000000000000000000000000000000000000000000000000000000000000000000000000000000000000000000000000000000000000000000000000000000000000000
>P08008
MKISSFISTSLPLPTSVSGSSSVGEMSGRSVSQQTSDQYANNLAGRTESPQGSSLASRIIERLSSVAHSVIGFIQRMFSEGSHKPVVTPAPTPAQMPSPTSFSDSIKQLAAETLPKYMQQLNSLDAEMLQKNHDQFATGSGPLRGSITQCQGLMQFCGGELQAEASAI
LNTPVCGIPFSQWGTIGGAASAYVASGVDLTQAANEIKGLAQQMQKLLSLM
11111111111111111111111111111111111111111111111111111111111111111111111111111111111111111111111111111110000000000000000000000000000000000000000000000000000000000000000000000000
0000000000000000000000000000000000000000000
11111111111111111111111111111111111111111111111111111111111111111111111111111111111111111111111111111110000000000000000000000000000000000000000000000000000000000000000000000000
0000000000000000000000000000000000000000000
00000000000000000000000000000000000000000000000000000000000000000000000000000000000000000000000000000000000000000000000000000000000000000000000000000000000000000000000000000000
0000000000000000000000000000000000000000000
>A0A0H3JEE8
MPPRLSYKTGGNMNRALSPMVSEFETIEQENSYNEWLRAKVATSLADPRPAIPHDEVERRMAERFAKMRKERSKQ
XXXXXXXXXXXX111111111111111111111111111111111111111111111111111111111111111
XXXXXXXXXXXX111111111111111111111111111111111111111111111111111111111111111
XXXXXXXXXXXX000000000000000000000000000000000000000000000000000000000000000
>O34800
MERAFQNRCEPRAAKPFKILKKRSTTSVASYQVSPHTARIFKENERLIDEYKRKKA
11111111111111111111111111111111111111111111111111111111
11111111111111111111111111111111111111111111111111111111
00000000000000000000000000000000000000000000000000000000
>Q9I322
MKIESISPVQPSQDAGAEAVGHFEGRSVTRAAVRGEDRSSVAGLARWLARNVAGDPRSEQALQRLADGDGTPLEARTVRRR
11111111111111111111111111111111111111111111111111111111111111111111111111111111
11111111111111111111111111111111111111111111111111111111111111111111111111111111
00000000000000000000000000000000000000000000000000000000000000000000000000000000
>P9WI73
MAKASETERSGPGTQPADAQTATSATVRPLSTQAVFRPDFGDEDNFPHPTLGPDTEPQDRMATTSRVRPPVRRLGGGLVEIPRAPDIDPLEALMTNPVVPESKRFCWNCGRPVGRSDSETKGASEGWCPYCGSPYSFLPQLNPGDIVAGQYEVKGCIAHGGLG
WIYLALDRNVNGRPVVLKGLVHSGDAEAQAMAMAERQFLAEVVHPSIVQIFNFVEHTDRHGDPVGYIVMEYVGGQSLKRSKGQKLPVAEAIAYLLEILPALSYLHSIGLVYNDLKPENIMLTEEQLKLIDLGAVSRINSFGYLYGTPGFQAPEIVRTGPTVATDIYTVG
RTLAALTLDLPTRNGRYVDGLPEDDPVLKTYDSYGRLLRRAIDPDPRQRFTTAEEMSAQLTGVLREVVAQDTGVPRPGLSTIFSPSRSTFGVDLLVAHTDVYLDGQVHAEKLTANEIVTALSVPLVDPTDVAASVLQATVLSQPVQTLDSLRAARHGALDADGVDFSE
SVELPLMEVRALLDLGDVAKATRKLDDLAERVGWRWRLVWYRAVAELLTGDYDSATKHFTEVLDTFPGELAPKLALAATAELAGNTDEHKFYQTVWSTNDGVISAAFGLARARSAEGDRVGAVRTLDEVPPTSRHFTTARLTSAVTLLSGRSTSEVTEEQIRDAAR
RVEALPPTEPRVLQIRALVLGGALDWLKDNKASTNHILGFPFTSHGLRLGVEASLRSLARVAPTQRHRYTLVDMANKVRPTSTF

11111111111111111111111111111111111111111111111111111111111111111111111111000000000000000000000000000000000000000000000000000000000000000000000000000000000000000000000000000000000000
00000000000000000000000000000000000000000000000000000000000000000000000000000000000000000000000000000000000000000000000000000000000000000000000000000000000000000000000000000000
00000000000000000000000000000000000000000000000000000000000000000000000000000000000000000000000000000000000000000000000000000000000000000000000000000000000000000000000000000000
00000000000000000000000000000000000000000000000000000000000000000000000000000000000000000000000000000000000000000000000000000000000000000000000000000000000000000000000000000000
00000000000000000000000000000000000000000000000000000000000000000000000000000000000000000000000000000000000000000000000000000000000000000000000000000000000000000000000000000000
0000000000000000000000000000000000000000000000
11111111111111111111111111111111111111111111111111111111111111111111111111000000000000000000000000000000000000000000000000000000000000000000000000000000000000000000000000000000000000
00000000000000000000000000000000000000000000000000000000000000000000000000000000000000000000000000000000000000000000000000000000000000000000000000000000000000000000000000000000
00000000000000000000000000000000000000000000000000000000000000000000000000000000000000000000000000000000000000000000000000000000000000000000000000000000000000000000000000000000
00000000000000000000000000000000000000000000000000000000000000000000000000000000000000000000000000000000000000000000000000000000000000000000000000000000000000000000000000000000
00000000000000000000000000000000000000000000000000000000000000000000000000000000000000000000000000000000000000000000000000000000000000000000000000000000000000000000000000000000
0000000000000000000000000000000000000000000000
00000000000000000000000000000000000000000000000000000000000000000000000000000000000000000000000000000000000000000000000000000000000000000000000000000000000000000000000000000000
00000000000000000000000000000000000000000000000000000000000000000000000000000000000000000000000000000000000000000000000000000000000000000000000000000000000000000000000000000000
00000000000000000000000000000000000000000000000000000000000000000000000000000000000000000000000000000000000000000000000000000000000000000000000000000000000000000000000000000000
00000000000000000000000000000000000000000000000000000000000000000000000000000000000000000000000000000000000000000000000000000000000000000000000000000000000000000000000000000000
00000000000000000000000000000000000000000000000000000000000000000000000000000000000000000000000000000000000000000000000000000000000000000000000000000000000000000000000000000000
0000000000000000000000000000000000000000000000
>P19594
MTKFTILLISLLFCIAHTCSASKWQHQQDSCRKQLQGVNLTPCEKHIMEKIQGRGDDDDDDDDDNHILRTMRGRINYIRRNEGKDEDEEEEGHMQKCCTEMSELRSPKCQCKALQKIMENQSEELEEKQKKKMEKELINLATMCRFGPMIQCDLSSDD
XXXXXXXXXXXXXXXXXXXXXXXX111111111111111111111111111111111111111000000000000000000000000000000000000000000000000000000000000000000000000000000000000000000000000000
XXXXXXXXXXXXXXXXXXXXXXXX111111111111111111111111111111111111111000000000000000000000000000000000000000000000000000000000000000000000000000000000000000000000000000
XXXXXXXXXXXXXXXXXXXXXXXX0000000000000000000000000000000000000000000000000000000000000000000000000000000000000000000000000000000000000000000
>P06899
MPEPAKSAPAPKKGSKKAVTKAQKKDGKKRKRSRKESYSIYVYKVLKQVHPDTGISSKAMGIMNSFVNDIFERIAGEASRLAHYNKRSTITSREIQTAVRLLLPGELAKHAVSEGTKAVTKYTSAK
111111111111111111111111111111111111111111111111111111111111111111111100000000000000000000000000000000000000000000000000000000
111111111111111111111111111111111111111111111111111111111111111111111100000000000000000000000000000000000000000000000000000000
000000000000000000000000000000000000000000000000000000000000000000000000000000000000000000000000000000000000000000000000000000
>Q96SD1
MSSFEGQMAEYPTISIDRFDRENLRARAYFLSHCHKDHMKGLRAPTLKRRLECSLKVYLYCSPVTKELLLTSPKYRFWKKRIISIEIETPTQISLVDEASGEKEEIVVTLLPAGHCPGSVMFLFQGNNGTVLYTGDFRLAQGEAARMELLHSGGRVKDIQSVYLDTTFCDP
RFYQIPSREECLSGVLELVRSWITRSPYHVVWLNCKAAYGYEYLFTNLSEELGVQVHVNKLDMFRNMPEILHHLTTDRNTQIHACRHPKAEEYFQWSKLPCGITSRNRIPLHIISIKPSTMWFGERSRKTNVIVRTGESSYRACFSFHSSYSEIKDFLSYLCPVNAYPNVIP
VGTTMDKVVEILKPLCRSSQSTEPKYKPLGKLKRARTVHRDSEEEDDYLFDDPLPIPLRHKVPYPETFHPEVFSMTAVSEKQPEKLRQTPGCCRAECMQSSRFTNFVDCEESNSESEEEVGIPASLQGDLGSVLHLQKADGDVPQWEVFFKRNDEITDESLENFPSSTV
AGGSQSPKLFSDSDGESTHISSQNSSQSTHITEQGSQGWDSQSDTVLLSSQERNSGDITSLDKADYRPTIKENIPASLMEQNVICPKDTYSDLKSRDKDVTIVPSTGEPTTLSSETHIPEEKSLLNLSTNADSQSSSDFEVPSTPEAELPKREHLQYLYEKLATGESIAVKKR
KCSLLDT
00000000000000000000000000000000000000000000000000000000000000000000000000000000000000000000000000000000000000000000000000000000000000000000000000000000000000000000000000000000
00000000000000000000000000000000000000000000000000000000000000000000000000000000000000000000000000000000000000000000000000000000000000000000000000000000000000000000000000000000
00000000000000000000000000000000000000000000000000000000000000000000000000000000000000000000000000000000000000000000000000000000000000011111111111111111111111111111111111111111111111111
11111111111111111111111111111111111111111111111XXXXXXXXXXXXXXXXXXXXXXXXXXXXXXXXXXXXXXXXXXXXXXXXXXXXXXXXXXXXXXXXXXXXXXXXXXXXXXXXXXXXXXXXXXXXXXXXXXXXXXXXXXX
00000000000000000000000000000000000000000000000000000000000000000000000000000000000000000000000000000000000000000000000000000000000000000000000000000000000000000000000000000000
00000000000000000000000000000000000000000000000000000000000000000000000000000000000000000000000000000000000000000000000000000000000000000000000000000000000000000000000000000000
00000000000000000000000000000000000000000000000000000000000000000000000000000000000000000000000000000000000000000000000000000000000000000000000000000000000000000000000000000000
00000000000000000000000000000000000000000000000XXXXXXXXXXXXXXXXXXXXXXXXXXXXXXXXXXXXXXXXXXXXXXXXXXXXXXXXXXXXXXXXXXXXXXXXXXXXXXXXXXXXXXXXXXXXXXXXXXXXXXXXXXX
00000000000000000000000000000000000000000000000000000000000000000000000000000000000000000000000000000000000000000000000000000000000000000000000000000000000000000000000000000000
00000000000000000000000000000000000000000000000000000000000000000000000000000000000000000000000000000000000000000000000000000000000000000000000000000000000000000000000000000000
00000000000000000000000000000000000000000000000000000000000000000000000000000000000000000000000000000000000000000000000000000000000000000000000000000000000000000000000000000000
00000000000000000000000000000000000000000000000XXXXXXXXXXXXXXXXXXXXXXXXXXXXXXXXXXXXXXXXXXXXXXXXXXXXXXXXXXXXXXXXXXXXXXXXXXXXXXXXXXXXXXXXXXXXXXXXXXXXXXXXXXX
>O30916
MQIQSFYHSASLKTQEAFKSLQKTLYNGMQILSGQGKAPAKAPDARPEIIVLREPGATWGNYLQHQKASNHSLHNLYNLQRDLLTVAATVLGKQDPVLTSMANQMELAKVKADRPATKQEEAAAKALKKNLIELIAARTQQQDGLPAKEAHRFAAVAFRDAQVK
QLNNQPWQTIKNTLTHNGHHYTNTQLPAAEMKIGAKDIFPSAYEGKGVCSWDTKNIHHANNLWMSTVSVHEDGKDKTLFCGIRHGVLSPYHEKDPLLRHVGAENKAKEVLTAALFSKPELLNKALAGEAVSLKLVSVGLLTASNIFGKEGTMVEDQMRAWQSLT
QPGKMIHLKIRNKDGDLQTVKIKPDVAAFNVGVNELALKLGFGLKASDSYNAEALHQLLGNDLRPEARPGGWVGEWLAQYPDNYEVVNTLARQIKDIWKNNQHHKDGGEPYKLAQRLAMLAHEIDAVPAWNCKSGKDRTGMMDSEIKREIISLHQTHMLSAP
GSLPDSGGQKIFQKVLLNSGNLEIQKQNTGGAGNKVMKNLSPEVLNLSYQKRVGDENIWQSVKGISSLITS
111111111111111111111111111111111111111111111111111111111111111111111111111111111111111111111111111111111111111111111111111111111111111111111111111111111111111111111XX111111111
11111XXXXXXXXXXXXXXXXXXXXXXXXXXXXXXXXXXXXXXXXXXXXXXXXXXXXXXXXXXXXXXXXXXXXXXXXXXXXXXXXXXXXXXXXXXXXXXXXXXXXXXXXXXXXXXXXXXXXXXXXXXXXXXXXXXXXXXXXXXXXXXXXXXXXXXXXXXX
XXXXXXXXXXXXXXXXXXXXXXXXXXXXXXXXXXXXXXXXXXXXXXXXXXXXXXXXXXXXXXXXXXXXXXXXXXXXXXXXXXXXXXXXXXXXXXXXXXXXXXXXXXXXXXXXXXXXXXXXXXXXXXXXXXXXXXXXXXXXXXXXXXXXXXXXXXXXXXXXXXXX
XXXXXXXXXXXXXXXXXXXXXXXXXXXXXXXXXXXXXXXXX
0000000000000000000000000000000111111111111111111111111111111111111111111111111111111111111111111111111111111111111111111111111111111111111111111111111111111111111111XX000000000
00000XXXXXXXXXXXXXXXXXXXXXXXXXXXXXXXXXXXXXXXXXXXXXXXXXXXXXXXXXXXXXXXXXXXXXXXXXXXXXXXXXXXXXXXXXXXXXXXXXXXXXXXXXXXXXXXXXXXXXXXXXXXXXXXXXXXXXXXXXXXXXXXXXXXXXXXXXXXXX
XXXXXXXXXXXXXXXXXXXXXXXXXXXXXXXXXXXXXXXXXXXXXXXXXXXXXXXXXXXXXXXXXXXXXXXXXXXXXXXXXXXXXXXXXXXXXXXXXXXXXXXXXXXXXXXXXXXXXXXXXXXXXXXXXXXXXXXXXXXXXXXXXXXXXXXXXXXXXXXXXXXXX
XXXXXXXXXXXXXXXXXXXXXXXXXXXXXXXXXXXXXXXXX
000000000000000000000000000000000000000000000000000000000000000000000000000000000000000000000000000000000000000000000000000000000000000000000000000000000000000000000XX000000000
00000XXXXXXXXXXXXXXXXXXXXXXXXXXXXXXXXXXXXXXXXXXXXXXXXXXXXXXXXXXXXXXXXXXXXXXXXXXXXXXXXXXXXXXXXXXXXXXXXXXXXXXXXXXXXXXXXXXXXXXXXXXXXXXXXXXXXXXXXXXXXXXXXXXXXXXXXXXXXX
XXXXXXXXXXXXXXXXXXXXXXXXXXXXXXXXXXXXXXXXXXXXXXXXXXXXXXXXXXXXXXXXXXXXXXXXXXXXXXXXXXXXXXXXXXXXXXXXXXXXXXXXXXXXXXXXXXXXXXXXXXXXXXXXXXXXXXXXXXXXXXXXXXXXXXXXXXXXXXXXXXXXX
XXXXXXXXXXXXXXXXXXXXXXXXXXXXXXXXXXXXXXXXX
>F9UST4
MATLATKKATLVAALKDLQRVTVAFSGGIDSTLVLKMALDVLGRDNVTAVVANSELFTDEEFDKAMSLAEELGANVQGTTLDYLSDDHIKNNTPDSWYYAKKMFYSRLNDIAANNGSAAVLDGMIKNDENDYRPGLKARSEAGARSLLQEADFFKTDVRALAQEL
GLTNWNKVASCSVSSRFPYGTTLTHDNIAQVMAAEKYLRSLGFPTVRVRFHNDIARIELPEARIGDFLVFNDRVNRQLQSLGFRYVTLDLGGFRSGRMNDTLTKAQLATFA
00000000000000000000000000000000000000000000000000000000000000000000000000000000000000000000000000000000000000000000000000000001111111111111111111110000000000000000000000000000
0000000000000000000000000000000000000000000000000000000000000000000000000000000000000000000000000000
00000000000000000000000000000000000000000000000000000000000000000000000000000000000000000000000000000000000000000000000000000000000000000000000000000000000000000000000000000000
0000000000000000000000000000000000000000000000000000000000000000000000000000000000000000000000000000
00000000000000000000000000000000000000000000000000000000000000000000000000000000000000000000000000000000000000000000000000000000000000000000000000000000000000000000000000000000
0000000000000000000000000000000000000000000000000000000000000000000000000000000000000000000000000000
>P24255
MKQGLQLRLSQQLAMTPQLQQAIRLLQLSTLELQQELQQALESNPLLEQIDTHEEIDTRETQDSETLDTADALEQKEMPEELPLDASWDTIYTAGTPSGTSGDYIDDELPVYQGETTQTLQDYLMWQVELTPFSDTDRAIATSIVDAVDETGYLTVPLEDILESIGDEE
IDIDEVEAVLKRIQRFDPVGVAAKDLRDCLLIQLSQFDKTTPWLEEARLIISDHLDLLANHDFRTLMRVTRLKEDVLKEAVNLIQSLDPRPGQSIQTGEPEYVIPDVLVRKHNGHWTVELNSDSIPRLQINQHYASMCNNARNDGDSQFIRSNLQDAKWLIKSLESRND
TLLRVSRCIVEQQQAFFEQGEEYMKPMVLADIAQAVEMHESTISRVTTQKYLHSPRGIFELKYFFSSHVNTEGGGEASSTAIRALVKKLIAAENPAKPLSDSKLTSLLSEQGIMVARRTVAKYRESLSIPPSNQRKQLV
11111111111111111111111111111111111111111111111111111111111111111111111111111111111111111111111111111111111111111111111111111100000000000000000000000000000000000000000000000000
00000000000000000000000000000000000000000000000000000000000000000000000000000000000000000000000000000000000000000000000000000000000000000000000000000000000000000000000000000000
00000000000000000000000000000000000000000000000000000000000000000000000000000000000000000000000000000000000000000000000000000
11111111111111111111111111111111111111111111111111111111111111111111111111111111111111111111111111111111111111111111111111111100000000000000000000000000000000000000000000000000
00000000000000000000000000000000000000000000000000000000000000000000000000000000000000000000000000000000000000000000000000000000000000000000000000000000000000000000000000000000
00000000000000000000000000000000000000000000000000000000000000000000000000000000000000000000000000000000000000000000000000000
00000000000000000000000000000000000000000000000000000000000000000000000000000000000000000000000000000000000000000000000000000000000000000000000000000000000000000000000000000000
00000000000000000000000000000000000000000000000000000000000000000000000000000000000000000000000000000000000000000000000000000000000000000000000000000000000000000000000000000000
00000000000000000000000000000000000000000000000000000000000000000000000000000000000000000000000000000000000000000000000000000
>O43516
MPVPPPPAPPPPPTFALANTEKPTLNKTEQAGRNALLSDISKGKKLKKTVTNDRSAPILDKPKGAGAGGGGGGFGGGGGFGGGGGGGGGGSFGGGGPPGLGGLFQAGMPKLRSTANRDNDSGGSRPPLLPPGGRSTSAKPFSPPSGPGRFPVPSPGHRSGPPE
PQRNRMPPPRPDVGSKPDSIPPPVPSTPRPIQSSPHNRGSPPVPGGPRQPSPGPTPPPFPGNRGTALGGGSIRQSPLSSSSPFSNRPPLPPTPSRALDDKPPPPPPPVGNRPSIHREAVPPPPPQNNKPPVPSTPRPSASSQAPPPPPPPSRPGPPPLPPSSSGNDETP
RLPQRNLSLSSSTPPLPSPGRSGPLPPPPSERPPPPVRDPPGRSGPLPPPPPVSRNGSTSRALPATPQLPSRSGVDSPRSGPRPPLPPDRPSAGAPPPPPPSTSIRNGFQDSPCEDEWESRFYFHPISDLPPPEPYVQTTKSYPSKLARNESRSGSNRRERGAPPLPPIPR
X1111111111111111111111111111111111111111111111111111111111111111111111111111111111111111111111111111XXXXXXXXXXXXXXXXXXXXXXXXXXXXXXXXXXXXXXXXXXXXXXXXXXXXXXXXXXXXXXXXXXXXXXXXXX
XXXXXXXXXXXXXXXXXXXXXXXXXXXXXXXXXXXXXXXXXXXXXXXXXXXXXXXXXXXXXXXXXXXXXXXXXXXXXXXXXXXXXXXXXXXXXXXXXXXXXXXXXXXXXXXXXXXXXXXXXXXXXXXXXXXXXXXXXXXXXXXXXXXXXXXXXXXXXXXXXXXX
XXXXXXXXXXXXXXXXXXXXXXXXXXXXXXXXXXXXXXXXXXXXXXXXXXXXXXXXXXXXX11111111111111111111111111111111111111111111111111111111111111111111111111111111111111111111111111111111111111111111111
X1111111111111111111111111111111111111111111111111111111111111111111111111111111111111111111111111111XXXXXXXXXXXXXXXXXXXXXXXXXXXXXXXXXXXXXXXXXXXXXXXXXXXXXXXXXXXXXXXXXXXXXXXXXX
XXXXXXXXXXXXXXXXXXXXXXXXXXXXXXXXXXXXXXXXXXXXXXXXXXXXXXXXXXXXXXXXXXXXXXXXXXXXXXXXXXXXXXXXXXXXXXXXXXXXXXXXXXXXXXXXXXXXXXXXXXXXXXXXXXXXXXXXXXXXXXXXXXXXXXXXXXXXXXXXXXXX
XXXXXXXXXXXXXXXXXXXXXXXXXXXXXXXXXXXXXXXXXXXXXXXXXXXXXXXXXXXXX11111111111111111111111111111111111111111111111111111111111111111111111111111111111111111111111111111111111111111111111
X0000000000000000000000000000000000000000000000000000000000000000000000000000000000000000000000000000XXXXXXXXXXXXXXXXXXXXXXXXXXXXXXXXXXXXXXXXXXXXXXXXXXXXXXXXXXXXXXXXXXXXXXXXXX
XXXXXXXXXXXXXXXXXXXXXXXXXXXXXXXXXXXXXXXXXXXXXXXXXXXXXXXXXXXXXXXXXXXXXXXXXXXXXXXXXXXXXXXXXXXXXXXXXXXXXXXXXXXXXXXXXXXXXXXXXXXXXXXXXXXXXXXXXXXXXXXXXXXXXXXXXXXXXXXXXXXX
XXXXXXXXXXXXXXXXXXXXXXXXXXXXXXXXXXXXXXXXXXXXXXXXXXXXXXXXXXXXX00000000000000000000000000000000000000000000000000000000000000000000000000000000000000000000000000000000000000000000000
>P0AE98
MRVKHAVVLLMLISPLSWAGTMTFQFRNPNFGGNPNNGAFLLNSAQAQNSYKDPSYNDDFGIETPSALDNFTQAIQSQILGGLLSNINTGKPGRMVTNDYIVDIANRDGQLQLNVTDRKTGQTSTIQVSGLQNNSTDF
XXXXXXXXXXXXXXXXXXXXXXXX111111111111111111111111111XXXXXXXXXXXXXXXXXXXXXXXXXXXXXXXXXXXXXXXXXXXXXXXXXXXXXXXXXXXXXXXXXXXXXXXXXXXXXXXXXXXXXXXXXXXXXXXXX
XXXXXXXXXXXXXXXXXXXXXXXX000000000000000000000000000XXXXXXXXXXXXXXXXXXXXXXXXXXXXXXXXXXXXXXXXXXXXXXXXXXXXXXXXXXXXXXXXXXXXXXXXXXXXXXXXXXXXXXXXXXXXXXXXXXXXX
XXXXXXXXXXXXXXXXXXXXXXXX000000000000000000000000000XXXXXXXXXXXXXXXXXXXXXXXXXXXXXXXXXXXXXXXXXXXXXXXXXXXXXXXXXXXXXXXXXXXXXXXXXXXXXXXXXXXXXXXXXXXXXXXXXXXX
>Q14978
MADAGIRRVVPSDLYPLVLGFLRDNQLSEVANKFAKATGATQQDANASSLLDIYSFWLKSAKVPERKLQANGPVAKKAKKKASSSDSEDSSEEEEEVQGPPAKKAAVPAKRVGLPPGKAAAKASESSSSEESSDDDDEEDQKKQPVQKGVKPQAKAAKAPPKKAKS
SDSDSDSSSEDEPPKNQKPKITPVTVKAQTKAPPKPARAAPKIANGKAASSSSSSSSSSSDDSEEEKAAATPKKTVPKKQVVAKAPVKAATTPTRKSSSSEDSSSDEEEEQKKPMKNKPGPYSSVPPPSAPPPKKSLGTQPPKKAVEKQQPVESSEDSSDESDSSSEEE
KKPPTKAVVSKATTKPPPAKKAAESSSDSSDSDSSEDDEAPSKPAGTTKNSSNKPAVTTKSPAVKPAAAPKQPVGGGQKLLTRKADSSSSEEESSSSEEEKTKKMVATTKPKATAKAALSLPAKQAPQGSRDSSSDSDSSSSEEEEEKTSKSAVKKKPQKVAGGAAPSK
PASAKKGKAESSNSSSSDDSSEEEEEKLKGKGSPRPQAPKANGTSALTAQNGKAAKNSEEEEEEKKKAAVVVSKSGSLKKRKQNEAAKEAETPQAKKIKLQTPNTFPKRKKGEKRASSPFRRVREEEIEVDSRVADNSFDAKRGAAGDWGERANQVLKFTKGKSFRH
EKTKKKRGSYRGGSISVQVNSIKFDSE
11111111111111111111111111111111111111111111111111111111111111111111111111111111111111111111111111111111111111111111111111111111111111111111111111111111111111111111111111111111
11111111111111111111111111111111111111111111111111111111111111111111111111111111111111111111111111111111111111111111111111111111111111111111111111111111111111111111111111111111
11111111111111111111111111111111111111111111111111111111111111111111111111111111111111111111111111111111111111111111111111111111111111111111111111111111111111111111111111111111
1111111111111111111111111111111111111111111111111111111111111111111111111111111111111111111111111111111111111111111111111111111111111111111111111111111111111111111111111111
11111111111111111111111111111111111111111111111111111111111111111111111111111111111111111111111111111111111111111111111111111111111111111111111111111111111111111111111111111111
11111111111111111111111111111111111111111111111111111111111111111111111111111111111111111111111111111111111111111111111111111111111111111111111111111111111111111111111111111111
11111111111111111111111111111111111111111111111111111111111111111111111111111111111111111111111111111111111111111111111111111111111111111111111111111111111111111111111111111111
1111111111111111111111111111111111111111111111111111111111111111111111111111111111111111111111111111111111111111111111111111111111111111111111111111111111111111111111111111
00000000000000000000000000000000000000000000000000000000000000000000000000000000000000000000000000000000000000000000000000000000000000000000000000000000000000000000000000000000
00000000000000000000000000000000000000000000000000000000000000000000000000000000000000000000000000000000000000000000000000000000000000000000000000000000000000000000000000000000
00000000000000000000000000000000000000000000000000000000000000000000000000000000000000000000000000000000000000000000000000000000000000000000000000000000000000000000000000000000
0000000000000000000000000000000000000000000000000000000000000000000000000000000000000000000000000000000000000000000000000000000000000000000000000000000000000000000000000000
>Q8RY59
MEAKIVKVLDSSRCEDGFGKKRKRAASYAAYVTGVSCAKLQNVPPPNGQCQIPDKRRRLEGENKLSAYENRSGKALVRYYTYFKKTGIAKRVMMYENGEWNDLPEHVICAIQNELEEKSAAIEFKLCGHSFILDFLHMQRLDMETGAKTPLAWIDNAGKCFFPEIYE
SDERTNYCHHKCVEDPKQNAPHDIKLRLEIDVNGGETPRLNLEECSDESGDNMMDDVPLAQRSSNEHYDEATEDSCSRKLEAAVSKWDETDAIVVSGAKLTGSEVLDKDAVKKMFAVGTASLGHVPVLDVGRFSSEIAEARLALFQKQVEITKKHRGDANVRYAW
LPAKREVLSAVMMQGLGVGGAFIRKSIYGVGIHLTAADCPYFSARYCDVDENGVRYMVLCRVIMGNMELLRGDKAQFFSGGEEYDNGVDDIESPKNYIVWNINMNTHIFPEFVVRFKLSNLPNAEGNLIAKRDNSGVTLEGPKDLPPQLESNQGARGSGSANSV
GSSTTRPKSPWMPFPTLFAAISHKVAENDMLLINADYQQLRDKKMTRAEFVRKLRVIVGDDLLRSTITTLQNQPKSKEIPGSIRDHEEGAGGL
00000000000000000000000000000000000000000000000000000000000000000000000000000000000000000000000000000000000000000000000000000000000000000000000000000000000000000000000000000000
00000000000000000000000000000000000000000000000000000000000000000000000000000000000000000000000000000000000000000000000000000000000000000000000000000000000000000000000000000000
0000000000000000000000000000000000000000000000000000000000000000000000000000000000000000000000000000000000000000000001111111111111111111111111111111111111111111111XXXXXXXXXXXXXXX
XXXXXXXXXXXXXXXXXXXXXXXXXXXXXXXXXXXXXXX1111111111111111111111

00000000000000000000000000000000000000000000000000000000000000000000000000000000000000000000000000000000000000000000000000000000000000000000000000000000000000000000000000000000
00000000000000000000000000000000000000000000000000000000000000000000000000000000000000000000000000000000000000000000000000000000000000000000000000000000000000000000000000000000
000000000000000000000000000000000000000000000000000000000000000000000000000000000000XXXXXXXXXXXXXXXXXXXXXXXXXXXXXXXXXXXXXXXXXXXXXXXXXXXXXXXXXXXXXXXXXXXXXXXXXXXXXXXXXXXXXXXXXXXXXXXX
XXXXXXXXXXXXXXXXXXXXXXXXXXXXXXXXXXXXXXXX0000000000000000000000
00000000000000000000000000000000000000000000000000000000000000000000000000000000000000000000000000000000000000000000000000000000000000000000000000000000000000000000000000000000
00000000000000000000000000000000000000000000000000000000000000000000000000000000000000000000000000000000000000000000000000000000000000000000000000000000000000000000000000000000
000000000000000000000000000000000000000000000000000000000000000000000000000000000000XXXXXXXXXXXXXXXXXXXXXXXXXXXXXXXXXXXXXXXXXXXXXXXXXXXXXXXXXXXXXXXXXXXXXXXXXXXXXXXXXXXXXXXXXXXXXXXX
XXXXXXXXXXXXXXXXXXXXXXXXXXXXXXXXXXXXXXXX0000000000000000000000
>P56621
MADVEEWLTHARKVTQEASIGVDVTSIQECISAEPAQRVLVARRDAWRAICCAAFAALVAFAAINRVATIMLEKPAPTWVATPSAASPFGLLIGK
X1111111111111111111111111111111111111111111111111111111111111111111111111111111111111111111111
X1111111111111111111111111111110000000000000000000000000000000000000000000000000000000000000000
X0000000000000000000000000000000000000000000000000000000000000000000000000000000000000000000000
>P46669
MSQVKRANENRETARFIKKHKKQVTNPIDEKNGTSNCIVRVPIALYVSLAPMYLENPLQGVMKQHLNPLVMKYNNKVGGVVLGYEGLKILDADPLSKEDTSEKLIKITPDTPFGFTWCHVNLYVWQPQVGDVLEGYIFIQSASHIGLLIHDAFNASIKKNNIPVDWTF
VHNDVEEDADVINTDENNGNNNNEDNKDSNGGSNSLGKFSFGNRSLGHWVDSNGEPIDGKLRFTVRNVHTTGRVVSVDGTLISDADEEGNGYNSSRSQAESLPIVSNKKIVFDDEVSIENKESHKELDLPEVKEDNGSEIVYEENTSESNDGESSDSD
0000000000000000000000000000000000000000000000000000000000000000000000000000000000000000000000111111111111111111000000000000000000000000000000000000000000000000000000000000000
000000000000000000000000000000000000000000000000000000000000000000000000000000000000000000000000000000000000000000000000000000000000000000000000000000
0000000000000000000000000000000000000000000000000000000000000000000000000000000000000000000000111111111111111111000000000000000000000000000000000000000000000000000000000000000
000000000000000000000000000000000000000000000000000000000000000000000000000000000000000000000000000000000000000000000000000000000000000000000000000000
00000000000000000000000000000000000000000000000000000000000000000000000000000000000000000000000000000000000000000000000000000000000000000000000000000000000000000000000000000000
000000000000000000000000000000000000000000000000000000000000000000000000000000000000000000000000000000000000000000000000000000000000000000000000000000
>P03086
MVLRQLSRKASVKVSKTWSGTKKRAQRILIFLLEFLLDFCTGEDSVDGKKRQRHSGLTEQTYSALPEPKAT
XXXXXXXXXXXXXXXXXXXXXXXXXXXXXXXXXXXXXXXXX111111111111111111111111111111
XXXXXXXXXXXXXXXXXXXXXXXXXXXXXXXXXXXXXXXXX000000000000000000000000000000
XXXXXXXXXXXXXXXXXXXXXXXXXXXXXXXXXXXXXXXXX000000000000000000000000000000
>O14140
MSRAALPSLENLEDDDEFEDFATENWPMKDTELDTGDDTLWENNWDDEDIGDDDFSVQLQAELKKKGVAAN
111111111111111111111111111111111111111111111111111111XXXXXXXXXXXXXXXXX
111111111111111111111111111111111111111111111111111111XXXXXXXXXXXXXXXXX
000000000000000000000000000000000000000000000000000000XXXXXXXXXXXXXXXXX
>P48439
MNWLFLVSLVFFCGVSTHPALAMSSNRLLKLANKSPKKIIPLKDSSFENILAPPHENAYIVALFTATAPEIGCSLCLELESEYDTIVASWFDDHPDAKSSNSDTSIFFTKVNLEDPSKTIPKAFQFFQLNNVPRLFIFKPNSPSILDHSVISISTDTGSERMKQIIQAIKQFSQV
NDFSLHLPMDWTPIITSTIITFITVLLFKKQSKLMFSIISSRIIWATLSTFFIICMISAYMFNQIRNTQLAGVGPKGEVMYFLPNEFQHQFAIETQVMVLIYGTLAALVVVLVKGIQFLRSHLYPETKKAYFIDAILASFCALFIYVFFAALTTVFTIKSPAYPFPLLRLSAPFK
XXXXXXXXXXXXXXXXXXXXXXXXXXX11111111111111111111111111111111111111111111111111111111111111111111111111111111111111111111111111111111111111111111111111111111111111111111111111
1111111111111111111111111111111111111111XXXXXXXXXXXXXXXXXXXXXXXXXXXXXXXXXXXXXXXXXXXXXXXXXXXXXXXXXXXXXXXXXXXXXXXXXXXXXXXXXXXXXXXXXXXXXXXXXXXXXXXXXXXXXXXXXXXXXXXXXXXXXXXX
X
XXXXXXXXXXXXXXXXXXXXXXXXXXX00000000000000000000000000000000000000000000000000000000000000000000000000000000000000000000000000000000000000000000000000000000000000000000000000
0000000000000000000000000000000000000000XXXXXXXXXXXXXXXXXXXXXXXXXXXXXXXXXXXXXXXXXXXXXXXXXXXXXXXXXXXXXXXXXXXXXXXXXXXXXXXXXXXXXXXXXXXXXXXXXXXXXXXXXXXXXXXXXXXXXXXXXXXXXXXX
X
XXXXXXXXXXXXXXXXXXXXXXXXXXX00000000000000000000000000000000000000000000000000000000000000000000000000000000000000000000000000000000000000000000000000000000000000000000000000
0000000000000000000000000000000000000000XXXXXXXXXXXXXXXXXXXXXXXXXXXXXXXXXXXXXXXXXXXXXXXXXXXXXXXXXXXXXXXXXXXXXXXXXXXXXXXXXXXXXXXXXXXXXXXXXXXXXXXXXXXXXXXXXXXXXXXXXXXXXXXX
X
>B9KDD4
MKLQQNFTDNNSIKYTCILILIAFAFSVLCRLYWVAWASEFYEFFFNDQLMITTNDGYAFAEGARDMIAGFHQPNDLSYFGSSLSTLTYWLYSILPFSFESIILYMSAFFASLIVVPIILIAREYKLTTYGFIAALLGSIANSYYNRTMSGYYDTDMLVLVLPMLILLTFIRLTIN
KDIFTLLLSPVFIMIYLWWYPSSYSLNFAMIGLFGLYTLVFHRKEKIFYLTIALMIIALSMLAWQYKLALIVLLFAIFAFKEEKINFYMIWALIFISILILHLSGGLDPVLYQLKFYVFKASDVQNLKDAAFMYFNVNETIMEVNTIDPEVFMQRISSSVLVFILSFIGFILLCKDHK
SMLLALPMLALGFMALRAGLRFTIYAVPVMALGFGYFLYAFFNFLEKKQIKLSLRNKNILLILIAFFSISPALMHIYYYKSSTVFTSYEASILNDLKNKAQREDYVVAWWDYGYPIRYYSDVKTLIDGGKHLGKDNFFSSFVLSKEQIPAANMARLSVEYTEKSFKENYPDVL
KAMVKDYNKTSAKDFLESLNDKDFKFDTNKTRDVYIYMPYRMLRIMPVVAQFANTNPDNGEQEKSLFFSQANAIAQDKTTGSVMLDNGVEIINDFRALKVEGASIPLKAFVDIESITNGKFYYNEIDSKAQIYLLFLREYKSFVILDESLYNSSYIQMFLLNQYDQDLFE
QITNDTRAKIYRLKR
00000000000000000000000000000000000000000000000000000000000000000000000000000000000000000000000000000000000000000000000000000000000000000000000000000000000000000000000000000000
00000000000000000000000000000000000000000000000000000000000000000000000000000000000000000000000000000000111111111111111111111111111111111111111111111111000000000000000000000000
00000000000000000000000000000000000000000000000000000000000000000000000000000000000000000000000000000000000000000000000000000000000000000000000000000000000000000000000000000000
00000000000000000000000000000000000000000000000000000000000000000000000000000000000000000000000000000000000000000000000000000000000000000000000000000000000000000000000000000000
00000000
00000000000000000000000000000000000000000000000000000000000000000000000000000000000000000000000000000000000000000000000000000000000000000000000000000000000000000000000000000000
00000000000000000000000000000000000000000000000000000000000000000000000000000000000000000000000000000000111111111111111111111111111111111111111111111111000000000000000000000000
00000000000000000000000000000000000000000000000000000000000000000000000000000000000000000000000000000000000000000000000000000000000000000000000000000000000000000000000000000000
00000000000000000000000000000000000000000000000000000000000000000000000000000000000000000000000000000000000000000000000000000000000000000000000000000000000000000000000000000000
00000000
00000000000000000000000000000000000000000000000000000000000000000000000000000000000000000000000000000000000000000000000000000000000000000000000000000000000000000000000000000000
00000000000000000000000000000000000000000000000000000000000000000000000000000000000000000000000000000000000000000000000000000000000000000000000000000000000000000000000000000000
00000000000000000000000000000000000000000000000000000000000000000000000000000000000000000000000000000000000000000000000000000000000000000000000000000000000000000000000000000000
00000000000000000000000000000000000000000000000000000000000000000000000000000000000000000000000000000000000000000000000000000000000000000000000000000000000000000000000000000000
00000000
>Q47184
MDTSTTASVASANASTSTSMAYDLGSMSKDDVIDLFNKLGVFQAAILMFAYMYQAQSDLSIAKFADMNEASKESTTAQKMANLVDAKIADVQSSSDKNAKAQLPDEVISYINDPRNDITISGIDNINAQLGAGDLQTVKAAISAKANNLTTTVNNSQLEIQQMSN
TLNLLTSARSDMQSLQYRTISGISLGK
11111111111111111111111111111100000000000000000000000000000011111111111111111111111111111111111111111111111111111111111111111111111111111111111111000000000000000000000000000000
000000000000000000
11111111111111111111111111111100000000000000000000000000000011111111111111111111111111111111111111111111111111111111111111111111111111111111111111000000000000000000000000000000
000000000000000000
00000000000000000000000000000000000000000000000000000000000000000000000000000000000000000000000000000000000000000000000000000000000000000000000000000000000000000000000000000000
000000000000000000
>O55000
MGSGPIDPKELLKGLDSFLTRDGEVKSVDGIAKIFSLMKEARKMVSRCTYLNIILQTRAPEVLVKFIDVGGYKLLNSWLTYSKTTNNIPLLQQILLTLQHLPLTVDHLKQNNTAKLVKQLSKSSEDEELRKLASVLVSDWMAVIRSQSSTQPAEKDKKKRKEEGKSRTTLP
ERPLTEVKAETRAEEAPEKKKEKPKSLRTTAPSHAKFRSTGLELDTPSLVPVKKNSSTVVVSDKYNLKPIPLKRQSATAAPGDAAPPAEKKYKPLNTTPNTTKEIKVKIIPPQPMEGLGFLDALNSAPVPGIKIKKKKKVLSPTAAKPSPFEGKTSTEPSTAKPSSPEPAAPAE
PMDTDRPGTPVPAVEVPELMDAASSEPGALDAKPVESPGDPNQLTRKGRKRKTVTWPEEGKLREYFYFELDETERVNVNKIKDFGEAAKREILSDRHAFETARRLSHDNMEEKVPWVCPRPLVLPSPLVIPGSNSQERYIQAEREKGILQELFLNKESPHEPDPEPYE
PIPPKLIPLDEECAMDETPYVETLEPGGSGGSPDGAGGSKLPPVLANLMGSMGAGKSPQGPGGGGINVQEILTSIMGSPNNHPSEELLKQPDYSDKLKQMLVPHGLLGPGPVANGFPPGGPGGPKGMQHFPPGPGGPMPGPHGGPGGPVGPRLLGPPPPSRG
GDPFWDGPGDPMRGGPMRGGPGPGPGPYHRGRGGRGGNEPPPPPPFRGARGGRSGGGPPNGRGGPGGGGMVGGGGHRPHEGPGGSMGSGHRSHEGPGGSMGSGHRSHEGPGHGGPHGHRPHDVPSHRGHDHRGPPPHEHRGHDGHGGGGHR
GHDGGHSHGGDMSNRPVCRHFMMKGNCRYENNCAFYHPGVNGPPLP
XXXXXXXXXXXXXXXXXXXXXXXXXXXXXXXXXXXXXXXXXXXXXXXXXXXXXXXXXXXXXXXXXXXXXXXXXXXXXXXXXXXXXXXXXXXXXXXXXXXXXXXXXXXXXXXXXXXXXXXXXXXXXXXXXXXXXXXXXXXXXXXXXXXXXXXXXXXXXXXXXXXXXXXXXXXX
XXXXXXXXXXXXXXXXXXXXXXXXXXXXXXXXXXXXXXXXXXXXXXXXXXXXXXXXXXXXXXXXXXXXXXXXXXXXXXXXXXXXXXXXXXXXXXXXXXXXXXXXXXXXXXXXXXXXXXXXXXXXXXXXXXXXXXXXXXX1111111111111111111111111111111111111
1111111111111111111111111111111111111111111111111111111111111111111111111111111111111111XXXXXXXXXXXXXXXXXXXXXXXXXXXXXXXXXXXXXXXXXXXXXXXXXXXXXXXXXXXXXXXXXXXXXXXXXXXXXXXXXXXXXXXX
XXXXXXXXXXXXXXXXXXXXXXXXXXXXXXXXXXXXXXXXXXXXXXXXXXXXXXXXXXXXXXXXXXXXXXXXXXXXXXXXXXXXXXXXXXXXXXXXXXXXXXXXXXXXXXXXXXXXXXXXXXXXXXXXXXXXXXXXXXXXXXXXXXXXXXXXXXXXXXXXXXXXXXXXXXXX
XXXXXXXXXXXXXXXXXXXXXXXXXXXXXXXXXXXXXXXXXXXXXXXXXXXXXXXXXXXXXXXXXXXXXXXXXXXXXXXXXXXXXXXXXXXXXXXXXXXXXXXXXXXXXXXXXXXXXXXXXXXXXXXXXXXXXXXXXXXXXXXXXXXXXXXXXXXXXXXXXXXXXXXXXXXX
XXXXXXXXX
XXXXXXXXXXXXXXXXXXXXXXXXXXXXXXXXXXXXXXXXXXXXXXXXXXXXXXXXXXXXXXXXXXXXXXXXXXXXXXXXXXXXXXXXXXXXXXXXXXXXXXXXXXXXXXXXXXXXXXXXXXXXXXXXXXXXXXXXXXXXXXXXXXXXXXXXXXXXXXXXXXXXXXXXXXXX
XXXXXXXXXXXXXXXXXXXXXXXXXXXXXXXXXXXXXXXXXXXXXXXXXXXXXXXXXXXXXXXXXXXXXXXXXXXXXXXXXXXXXXXXXXXXXXXXXXXXXXXXXXXXXXXXXXXXXXXXXXXXXXXXXXXXXXXXXXX1111111111111111111111111111111111111
1111111111111111111111111111111111111111111111111111111111111111111111111111111111111111XXXXXXXXXXXXXXXXXXXXXXXXXXXXXXXXXXXXXXXXXXXXXXXXXXXXXXXXXXXXXXXXXXXXXXXXXXXXXXXXXXXXXXXX
XXXXXXXXXXXXXXXXXXXXXXXXXXXXXXXXXXXXXXXXXXXXXXXXXXXXXXXXXXXXXXXXXXXXXXXXXXXXXXXXXXXXXXXXXXXXXXXXXXXXXXXXXXXXXXXXXXXXXXXXXXXXXXXXXXXXXXXXXXXXXXXXXXXXXXXXXXXXXXXXXXXXXXXXXXX
XXXXXXXXXXXXXXXXXXXXXXXXXXXXXXXXXXXXXXXXXXXXXXXXXXXXXXXXXXXXXXXXXXXXXXXXXXXXXXXXXXXXXXXXXXXXXXXXXXXXXXXXXXXXXXXXXXXXXXXXXXXXXXXXXXXXXXXXXXXXXXXXXXXXXXXXXXXXXXXXXXXXXXXXXXX
XXXXXXXXX
XXXXXXXXXXXXXXXXXXXXXXXXXXXXXXXXXXXXXXXXXXXXXXXXXXXXXXXXXXXXXXXXXXXXXXXXXXXXXXXXXXXXXXXXXXXXXXXXXXXXXXXXXXXXXXXXXXXXXXXXXXXXXXXXXXXXXXXXXXXXXXXXXXXXXXXXXXXXXXXXXXXXXXXXXXXX
XXXXXXXXXXXXXXXXXXXXXXXXXXXXXXXXXXXXXXXXXXXXXXXXXXXXXXXXXXXXXXXXXXXXXXXXXXXXXXXXXXXXXXXXXXXXXXXXXXXXXXXXXXXXXXXXXXXXXXXXXXXXXXXXXXXXXXXXXXX0000000000000000000000000000000000000
0000000000000000000000000000000000000000000000000000000000000000000000000000000000000000XXXXXXXXXXXXXXXXXXXXXXXXXXXXXXXXXXXXXXXXXXXXXXXXXXXXXXXXXXXXXXXXXXXXXXXXXXXXXXXXXXXXXXXX
XXXXXXXXXXXXXXXXXXXXXXXXXXXXXXXXXXXXXXXXXXXXXXXXXXXXXXXXXXXXXXXXXXXXXXXXXXXXXXXXXXXXXXXXXXXXXXXXXXXXXXXXXXXXXXXXXXXXXXXXXXXXXXXXXXXXXXXXXXXXXXXXXXXXXXXXXXXXXXXXXXXXXXXXXXXX
XXXXXXXXXXXXXXXXXXXXXXXXXXXXXXXXXXXXXXXXXXXXXXXXXXXXXXXXXXXXXXXXXXXXXXXXXXXXXXXXXXXXXXXXXXXXXXXXXXXXXXXXXXXXXXXXXXXXXXXXXXXXXXXXXXXXXXXXXXXXXXXXXXXXXXXXXXXXXXXXXXXXXXXXXXX
XXXXXXXXX
>O75807
MAPGQAPHQATPWRDAHPFFLLSPVMGLLSRAWSRLRGLGPLEPWLVEAVKGAALVEAGLEGEARTPLAIPHTPWGRRPEEEAEDSGGPGEDRETLGLKTSSSLPEAWGLLDDDDGMYGEREATSVPRGQGSQFADGQRAPLSPSLLIRTLQGSDKNPGEEKA
EEEGVAEEEGVNKFSYPPSHRECCPAVEEEDDEEAVKKEAHRTSTSALSPGSKPSTWVSCPGEEENQATEDKRTERSKGARKTSVSPRSSGSDPRSWEYRSGEASEEKEEKAHKETGKGEAAPGPQSSAPAQRPQLKSWWCQPSDEEEGEVKALGAAEKDGEAEC
PPCIPPPSAFLKAWVYWPGEDTEEEDEEEDEDSDSGSDEEEGEAEASSSTPATGVFLKSWVYQPGEDTEEEDEDSDTGSAEDEREAETSASTPPASAFLKAWVYRPGEDTEEEDEDVDSEDKEDDSEAALGEAESDPHPSHPDQRAHFRGWGYRPGKETEEEE
AAEDWGEAEPCPFRVAIYVPGEKPPPPWAPPRLPLRLQRRLKRPETPTHDPDPETPLKARKVRFSEKVTVHFLAVWAGPAQAARQGPWEQLARDRSRFARRITQAQEELSPCLTPAARARAWARLRNPPLAPIPALTQTLPSSSVPSSPVQTTPLSQAVATPSRSSA
AAAAALDLSGRRG
XXXXXXXXXXXXXXXXXXXXXXXXXXXXXXXXXXXXXXXXXXXXXXXXXXXXXXXXXXXXXXXXXXXXXXXXXXXXXXXXXXXXXXXXXXXXXXXXXXXXXXXXXXXXXXXXXXXXXXXXXXXXXXXXXXXXXXXXXXXXXXXXXXXXXXXXXXXXXXXXXXXXXXXXXX
XXXXXXXXXXXXXXXXXXXXXXXXXXXXXXXXXXXXXXXXXXXXXXXXXXXXXXXXXXXXXXXXXXXXXXXXXXXXXXXXXXXXXXXXXXXXXXXXXXXXXXXXXXXXXXXXXXXXXXXXXXXXXXXXXXXXXXXXXXXXXXXXXXXXXXXXXXXXXXXXXXXXXXXXXXX
XXXXXXXXXXXXXXXXXXXXXXXXXXXXXXXXXXXXXXXXXXXXXXXXXXXXXXXXXXXXXXXXXXXXXXXXXXXXXXXXXXXXXXXXXXXXXXXXXXXXXXXXXXXXXXXXXXXXXXXXXXXXXXXXXXXXXXXXXXXXXXXXXXXXXXXXXXXXXXXXXXXXXX1111
1111111111111111111111111111111111111111111111111111111111111111111111111111111111111111111111111111111111111111111XXXXXXXXXXXXXXXXXXXXXXXXXXXXXXXXXXXXXXXXXXXX
XXXXXXXXXXXXXXXXXXXXXXXXXXXXXXXXXXXXXXXXXXXXXXXXXXXXXXXXXXXXXXXXXXXXXXXXXXXXXXXXXXXXXXXXXXXXXXXXXXXXXXXXXXXXXXXXXXXXXXXXXXXXXXXXXXXXXXXXXXXXXXXXXXXXXXXXXXXXXXXXXXXXXXXXXXXX
XXXXXXXXXXXXXXXXXXXXXXXXXXXXXXXXXXXXXXXXXXXXXXXXXXXXXXXXXXXXXXXXXXXXXXXXXXXXXXXXXXXXXXXXXXXXXXXXXXXXXXXXXXXXXXXXXXXXXXXXXXXXXXXXXXXXXXXXXXXXXXXXXXXXXXXXXXXXXXXXXXXXXXXXXXX
XXXXXXXXXXXXXXXXXXXXXXXXXXXXXXXXXXXXXXXXXXXXXXXXXXXXXXXXXXXXXXXXXXXXXXXXXXXXXXXXXXXXXXXXXXXXXXXXXXXXXXXXXXXXXXXXXXXXXXXXXXXXXXXXXXXXXXXXXXXXXXXXXXXXXXXXXXXXXXXXXXXXXXX1111
1111111111111111111111111111111111111111111111111111111111111111111111111111111111111111111111111111111111111111111XXXXXXXXXXXXXXXXXXXXXXXXXXXXXXXXXXXXXXXXXXXX
XXXXXXXXXXXXXXXXXXXXXXXXXXXXXXXXXXXXXXXXXXXXXXXXXXXXXXXXXXXXXXXXXXXXXXXXXXXXXXXXXXXXXXXXXXXXXXXXXXXXXXXXXXXXXXXXXXXXXXXXXXXXXXXXXXXXXXXXXXXXXXXXXXXXXXXXXXXXXXXXXXXXXXXXXXXX
XXXXXXXXXXXXXXXXXXXXXXXXXXXXXXXXXXXXXXXXXXXXXXXXXXXXXXXXXXXXXXXXXXXXXXXXXXXXXXXXXXXXXXXXXXXXXXXXXXXXXXXXXXXXXXXXXXXXXXXXXXXXXXXXXXXXXXXXXXXXXXXXXXXXXXXXXXXXXXXXXXXXXXXXXXXX
XXXXXXXXXXXXXXXXXXXXXXXXXXXXXXXXXXXXXXXXXXXXXXXXXXXXXXXXXXXXXXXXXXXXXXXXXXXXXXXXXXXXXXXXXXXXXXXXXXXXXXXXXXXXXXXXXXXXXXXXXXXXXXXXXXXXXXXXXXXXXXXXXXXXXXXXXXXXXXXXXXXXXXX0000
0000000000000000000000000000000000000000000000000000000000000000000000000000000000000000000000000000000000000000000XXXXXXXXXXXXXXXXXXXXXXXXXXXXXXXXXXXXXXXXXXXX
>P36100
MDRPIDDIVKNLLKFVVRGFYGGSFVLVLDAILFHSVLAEDDLKQLLSINKTELGPLIARLRSDRLISIHKQREYPPNSKSVERVYYYVKYPHAIDAIKWKVHQVVQRLKDDLDKNSEPNGYMCPICLTKYTQLEAVQLLNFDRTEFLCSLCDEPLVEDDSGKKNKEKQDK
LNRLMDQIQPIIDSLKKIDDSRIEENTFEIALARLIPPQNQSHAAYTYNPKKGSTMFRPGDSAPLPNLMGTALGNDSSRRAGANSQATLHINITTASDEVAQRELQERQAEEKRKQNAVPEWHKQSTIGKTALGRLDNEEEFDPVVTASAMDSINPDNEPAQETSYQ
NNRTLTEQEMEERENEKTLNDYYAALAKKQAKLNKEEEEEEEEEEDEEEEEEEEMEDVMDDNDETARENALEDEFEDVTDTAGTAKTESNTSNDVKQESINDKTEDAVNATATASGPSANAKPNDGDDDDDDDDDEMDIEFEDV
00000000000000000000000000000000000000000000000000000000000000000000000000000000000000000000000000000000000000000000000000000000000000000000000000000000000000000000000000000000
00000000000000000011111111111111111111111111111111111111111111111111111111111111111111111111111111111111111111111111111111111111111111111111111111111111111111111111111111111111
1111111111111111111111111111111111111111111111111111111111111111111111111111111111111111111111111111111111111111111111111111111111
00000000000000000000000000000000000000000000000000000000000000000000000000000000000000000000000000000000000000000000000000000000000000000000000000000000000000000000000000000000
00000000000000000000000000000000000000000000000000000000000000000000000000000000000000000000000000000000000000000000000000000000000000000000000000000000000000000000000000000000
0000000000000000000000000000000000000000000000000000000000000000000000000000000000000000000000000000000000000000000000000000000000

00000000000000000000000000000000000000000000000000000000000000000000000000000000000000000000000000000000000000000000000000000000000000000000000000000000000000000000000000000000
00000000000000000000000000000000000000000000000000000000000000000000000000000000000000000000000000000000000000000000000000000000000000000000000000000000000000000000000000000000
0000000000000000000000000000000000000000000000000000000000000000000000000000000000000000000000000000000000000000000000000000000000
>P25638
MSQFEKQKEQGNSLFKQGLYREAVHCYDQLITAQPQNPVGYSNKAMALIKLGEYTQAIQMCQQGLRYTSTAEHVAIRSKLQYRLELAQGAVGSVQIPVVEVDELPEGYDRS
000000000000000000000000000000000000000000000000000000000000000000000000000000000000000000001111111111111111111
000000000000000000000000000000000000000000000000000000000000000000000000000000000000000000001111111111111111111
000000000000000000000000000000000000000000000000000000000000000000000000000000000000000000000000000000000000000
>O13916
MLRRNPTAIQITAEDVLAYDEEKLRQTLDSESTTEEALQKNEESTRLSPEKKKIIERRIGITQIFDSSMHPSQGGAAQS
0000000000000000000000001111111111111111111111111111111111111111111111111111111
0000000000000000000000000000000000000000000000000000000000000000000000000000000
0000000000000000000000000000000000000000000000000000000000000000000000000000000
>P02283
MPPKTSGKAAKKAGKAQKNITKTDKKKKRKRKESYAIYIYKVLKQVHPDTGISSKAMSIMNSFVNDIFERIAAEASRLAHYNKRSTITSREIQTAVRLLLPGELAKHAVSEGTKAVTKYTSSK
X11111111111111111111111111000000000000000000000000000000000000000000000000000000000000000000000000000000000000000000000000
X00000000000000000000000000000000000000000000000000000000000000000000000000000000000000000000000000000000000000000000000000
X00000000000000000000000000000000000000000000000000000000000000000000000000000000000000000000000000000000000000000000000000
>P05386
MASVSELACIYSALILHDDEVTVTEDKINALIKAAGVNVEPFWPGLFAKALANVNIGSLICNVGAGGPAPAAGAAPAGGPAPSTAAAPAEEKKVEAKKEESEESDDDMGFGLFD
000000000000000000000000000000000000000000000000000000000000000000011111111111111111111111111111111111111111111111
000000000000000000000000000000000000000000000000000000000000000000011111111111111111111111111111111111111111111111
000000000000000000000000000000000000000000000000000000000000000000000000000000000000000000000000000000000000000000
>P49848
MAEEKKLKLSNTVLPSESMKVVAESMGIAQIQEETCQLLTDEVSYRIKEIAQDALKFMHMGKRQKLTTSDIDYALKLKNVEPLYGFHAQEFIPFRFASGGGRELYFYEEKEVDLSDIINTPLPRVPLDVCLKAHWLSIEGCQPAIPENPPPAPKEQQKAEATEPLKSAKP
GQEEDGPLKGKGQGATTADGKGKEKKAPPLLEGAPLRLKPRSIHELSVEQQLYYKEITEACVGSCEAKRAEALQSIATDPGLYQMLPRFSTFISEGVRVNVVQNNLALLIYLMRMVKALMDNPTLYLEKYVHELIPAVMTCIVSRQLCLRPDVDNHWALRDFAARLV
AQICKHFSTTTNNIQSRITKTFTKSWVDEKTPWTTRYGSIAGLAELGHDVIKTLILPRLQQEGERIRSVLDGPVLSNIDRIGADHVQSLLLKHCAPVLAKLRPPPDNQDAYRAEFGSLGPLLCSQVVKARAQAALQAQQVNRTTLTITQPRPTLTLSQAPQPGPRTPGLL
KVPGSIALPVQTLVSARAAAPPQPSPPPTKFIVMSSSSSAPSTQQVLSLSTSAPGSGSTTTSPVTTTVPSVQPIVKLVSTATTAPPSTAPSGPGSVQKYIVVSLPPTGEGKGGPTSHPSPVPPPASSPSPLSGSALCGGKQEAGDSPPPAPGTPKANGSQPNSGSPQPAP
11111111111111111111111111111111111111111111111111111111111111111111111111111111111111111111111111111111111111111111111111111111111111111111111111111111111111111111111111111111
11111111111111111111111111111111111111111111111111111111111111111111110000000000000000000000000000000000000000000000000000000000000000000000000000000000000000000000000000000000
00000000000000000000000000000000000000000000000000000000000000000000001111111111111111111111111111111111111111111111111111111111111111111111111111111111111111111111111111111111
11111111111111111111111111111111111111111111111111111111111111111111111111111111111111111111111111111111111111111111111111111111111111111111111111111
11111111111111111111111111111111111111111111111111111111111111111111111111111111111111111111111111111111111111111111111111111111111111111111111111111111111111111111111111111111
11111111111111111111111111111111111111111111111111111111111111111111110000000000000000000000000000000000000000000000000000000000000000000000000000000000000000000000000000000000
00000000000000000000000000000000000000000000000000000000000000000000000000000000000000000000000000000000000000000000000000000000000000000000000000000000000000000000000000000000
00000000000000000000000000000000000000000000000000000000000000000000000000000000000000000000000000000000000000000000000000000000000000000000000000000
00000000000000000000000000000000000000000000000000000000000000000000000000000000000000000000000000000000000000000000000000000000000000000000000000000000000000000000000000000000
00000000000000000000000000000000000000000000000000000000000000000000000000000000000000000000000000000000000000000000000000000000000000000000000000000000000000000000000000000000
00000000000000000000000000000000000000000000000000000000000000000000000000000000000000000000000000000000000000000000000000000000000000000000000000000000000000000000000000000000
00000000000000000000000000000000000000000000000000000000000000000000000000000000000000000000000000000000000000000000000000000000000000000000000000000
>Q6VMQ6
MDSLEEPQKKVFKARKTMRVSDRQQLEAVYKVKEELLKTDVKLLNGNHENGDLDPTSPLENMDYIKDKEEVNGIEEICFDPEGSKAEWKETPCILSVNVKNKQDDDLNCEPLSPHNITPEPVSKLPAEPVSGDPAPGDLDAGDPASGVLASGDSTSGDPTSSEPSSS
DAASGDATSGDAPSGDVSPGDATSGDATADDLSSGDPTSSDPIPGEPVPVEPISGDCAADDIASSEITSVDLASGAPASTDPASDDLASGDLSSSELASDDLATGELASDELTSESTFDRTFEPKSVPVCEPVPEIDNIEPSSNKDDDFLEKNGADEKLEQIQSKDSLDEK
NKADNNIDANEETLETDDTTICSDRPPENEKKVEEDIITELALGEDAISSSMEIDQGEKNEDETSADLVETINENVIEDNKSENILENTDSMETDEIIPILEKLAPSEDELTCFSKTSLLPIDETNPDLEEKMESSFGSPSKQESSESLPKEAFLVLSDEEDISGEKDESEVISQN
ETCSPAEVESNEKDNKPEEEEQVIHEDDERPSEKNEFSRRKRSKSEDMDNVQSKRRRYMEEEYEAEFQVKITAKGDINQKLQKVIQWLLEEKLCALQCAVFDKTLAELKTRVEKIECNKRHKTVLTELQAKIARLTKRFEAAKEDLKKRHEHPPNPPVSPGKTVNDVNS
NNNMSYRNAGTVRQMLESKRNVSESAPPSFQTPVNTVSSTNLVTPPAVVSSQPKLQTPVTSGSLTATSVLPAPNTATVVATTQVPSGNPQPTISLQPLPVILHVPVAVSSQPQLLQSHPGTLVTNQPSGNVEFISVQSPPTVSGLTKNPVSLPSLPNPTKPNNVPSV
PSPSIQRNPTASAAPLGTTLAVQAVPTAHSIVQATRTSLPTVGPSGLYSPSTNRGPIQMKIPISAFSTSSAAEQNSNTTPRIENQTNKTIDASVSKKAADSTSQCGKATGSDSSGVIDLTMDDEESGASQDPKKLNHTPVSTMSSSQPVSRPLQPIQPAPPLQPSGVPTS
GPSQTTIHLLPTAPTTVNVTHRPVTQVTTRLPVPRAPANHQVVYTTLPAPPAQAPLRGTVMQAPAVRQVNPQNSVTVRVPQTTTYVVNNGLTLGSTGPQLTVHHRPPQVHTEPPRPVHPAPLPEAPQPQRLPPEAASTSLPQKPHLKLARVQSQNGIVLSWSVL
EVDRSCATVDSYHLYAYHEEPSATVPSQWKKIGEVKALPLPMACTLTQFVSGSKYYFAVRAKDIYGRFGPFCDPQSTDVISSTQSS
XXXXXXXXXXXXXXXXXXXXXXXXXXXXXXXXXXXXXXXXXXXXXXXXXXXXXXXXXXXXXXXXXXXXXXXXXXXXXXXXXXXXXXXXXXXXXXXXXXXXXXXXXXXXXXXXXXXXXXXXXXXXXXXXXXXXXXXXXXXXXXXXXXXXXXXXXXXXXXXXXXXXXXXXXXXXXXXXXXXXXXXX
XXXXXXXXXXXXXXXXXXXXXXXXXXXXXXXXXXXXXXXXXXXXXXXXXXXXXXXXXXXXXXXXXXXXXXXXXXXXXXXXXXXXXXXXXXXXXXXXXXXXXXXXXXXXXXXXXXXXXXXXXXXXXXXXXXXXXXXXXXXXXXXXXXXXXXXXXXXXXXXXXXXXXXXXXXXXXXXXXXXXXXXX
XXXXXXXXXXXXXXXXXXXXXXXXXXXXXXXXXXXXXXXXXXXXXXXXXXXXXXXXXXXXXXXXXXXXXXXXXXXXXXXXXXXXXXXXXXXXXXXXXXXXXXXXXXXXXXXXXXXXXXXXXXXXXXXXXXXXXXXXXXXXXXXXXXXXXXXXXXXXXXXXXXXXXXXXXXXXXXXXXXXXXXX
XXXXXXXXXXXXXXXXXXXXXXXXXXXXXXXXXXXXXXXXXXXXXXXXXXXXXXXXXXXXXXXXXXXXXXXXXXXXXXXXXXXXXXXXXXXXXXXXXXXXXXXXXXXXXXXXXXXXXXXXXXXXXXXXXXXXXXXXXXXXXXXXXXXXXXXXXXXXXXXXXXXXXXXXXXXXXXXXXXXXXX
XXXXXXXXXXXXXXXXXXXXXXXXXXXXXXXXXXXXXXXXXXXXXXXXXXXXXXXXXXXXXXXXXXXXXXXXXXXXXXXXXXXXXXXXXXXXXXXXXXXXXXXXXXXXXXXXXXXXXXXXXXXXXXXXXXXXXXXXXXXXXXXXXXXXXXXXXXXXXXXXXXXXXXXXXXXXXXXXXXXXXXX
XXXXXXXXXXXXXXXXXXXXXXXXXXXXXXXXXXXXXXXXXXXXXXXXXXXXXXXXXXXXXXXXXXXXXXXXXXXXXXXXXXXXXXXXXXXXXXXXXXXXXXXXXXXXXXXXXXXXXXXXXXXXXXXXXXXXXXXXXXXXXXXXXXXXXXXXXXXXXXXXXXXXXXXXXXXXXXXXXXXXXX
XXXXXXXXXXXXXXXXXXXXXXXXXXXXXXXXXXXXXXXXXXXXXXXXXXXXXXXXXXXXXXXXXXXXXXXXXXXXXXXXX111111111111111111111111111XXXXXXXXXXXXXXXXXXXXXXXXXXXXXXXXXXXXXXXXXXXXXXXXXXXXXXXXXXXXXXXXXXXXXXXX
XXXXXXXXXXXXXXXXXXXXXXXXXXXXXXXXXXXXXXXXXXXXXXXXXXXXXXXXXXXXXXXXXXXXXXXXXXXXXXXXXXXXXXXXXXXXXXXXXXXXXXXXXXXXXXXXXXXXXXXXXXXXXXXXXXXXXXXXXXXXXXXXXXXXXXXXXXXXXXXXXXXXXXXXXXXXXXXXXXXXXX
XXXXXXXXXXXXXXXXXXXXXXXXXXXXXXXXXXXXXXXXXXXXXXXXXXXXXXXXXXXXXXXXXXXXXXXX
XXXXXXXXXXXXXXXXXXXXXXXXXXXXXXXXXXXXXXXXXXXXXXXXXXXXXXXXXXXXXXXXXXXXXXXXXXXXXXXXXXXXXXXXXXXXXXXXXXXXXXXXXXXXXXXXXXXXXXXXXXXXXXXXXXXXXXXXXXXXXXXXXXXXXXXXXXXXXXXXXXXXXXXXXXXXXXXXXXXXXXXX
XXXXXXXXXXXXXXXXXXXXXXXXXXXXXXXXXXXXXXXXXXXXXXXXXXXXXXXXXXXXXXXXXXXXXXXXXXXXXXXXXXXXXXXXXXXXXXXXXXXXXXXXXXXXXXXXXXXXXXXXXXXXXXXXXXXXXXXXXXXXXXXXXXXXXXXXXXXXXXXXXXXXXXXXXXXXXXXXXXXXXX
XXXXXXXXXXXXXXXXXXXXXXXXXXXXXXXXXXXXXXXXXXXXXXXXXXXXXXXXXXXXXXXXXXXXXXXXXXXXXXXXXXXXXXXXXXXXXXXXXXXXXXXXXXXXXXXXXXXXXXXXXXXXXXXXXXXXXXXXXXXXXXXXXXXXXXXXXXXXXXXXXXXXXXXXXXXXXXXXXXXXXX
XXXXXXXXXXXXXXXXXXXXXXXXXXXXXXXXXXXXXXXXXXXXXXXXXXXXXXXXXXXXXXXXXXXXXXXXXXXXXXXXXXXXXXXXXXXXXXXXXXXXXXXXXXXXXXXXXXXXXXXXXXXXXXXXXXXXXXXXXXXXXXXXXXXXXXXXXXXXXXXXXXXXXXXXXXXXXXXXXXXXX
XXXXXXXXXXXXXXXXXXXXXXXXXXXXXXXXXXXXXXXXXXXXXXXXXXXXXXXXXXXXXXXXXXXXXXXXXXXXXXXXXXXXXXXXXXXXXXXXXXXXXXXXXXXXXXXXXXXXXXXXXXXXXXXXXXXXXXXXXXXXXXXXXXXXXXXXXXXXXXXXXXXXXXXXXXXXXXXXXXXXX
XXXXXXXXXXXXXXXXXXXXXXXXXXXXXXXXXXXXXXXXXXXXXXXXXXXXXXXXXXXXXXXXXXXXXXXXXXXXXXXXXXXXXXXXXXXXXXXXXXXXXXXXXXXXXXXXXXXXXXXXXXXXXXXXXXXXXXXXXXXXXXXXXXXXXXXXXXXXXXXXXXXXXXXXXXXXXXXXXXXXX
XXXXXXXXXXXXXXXXXXXXXXXXXXXXXXXXXXXXXXXXXXXXXXXXXXXXXXXXXXXXXXXXXXXXXXXXXXXXXXXXX000000000000000000000000000XXXXXXXXXXXXXXXXXXXXXXXXXXXXXXXXXXXXXXXXXXXXXXXXXXXXXXXXXXXXXXXXXXXXXXXX
XXXXXXXXXXXXXXXXXXXXXXXXXXXXXXXXXXXXXXXXXXXXXXXXXXXXXXXXXXXXXXXXXXXXXXXXXXXXXXXXXXXXXXXXXXXXXXXXXXXXXXXXXXXXXXXXXXXXXXXXXXXXXXXXXXXXXXXXXXXXXXXXXXXXXXXXXXXXXXXXXXXXXXXXXXXXXXXXXXXXX
XXXXXXXXXXXXXXXXXXXXXXXXXXXXXXXXXXXXXXXXXXXXXXXXXXXXXXXXXXXXXXXXXXXXXXXX
XXXXXXXXXXXXXXXXXXXXXXXXXXXXXXXXXXXXXXXXXXXXXXXXXXXXXXXXXXXXXXXXXXXXXXXXXXXXXXXXXXXXXXXXXXXXXXXXXXXXXXXXXXXXXXXXXXXXXXXXXXXXXXXXXXXXXXXXXXXXXXXXXXXXXXXXXXXXXXXXXXXXXXXXXXXXXXXXXXXXX
XXXXXXXXXXXXXXXXXXXXXXXXXXXXXXXXXXXXXXXXXXXXXXXXXXXXXXXXXXXXXXXXXXXXXXXXXXXXXXXXXXXXXXXXXXXXXXXXXXXXXXXXXXXXXXXXXXXXXXXXXXXXXXXXXXXXXXXXXXXXXXXXXXXXXXXXXXXXXXXXXXXXXXXXXXXXXXXXXXXX
XXXXXXXXXXXXXXXXXXXXXXXXXXXXXXXXXXXXXXXXXXXXXXXXXXXXXXXXXXXXXXXXXXXXXXXXXXXXXXXXXXXXXXXXXXXXXXXXXXXXXXXXXXXXXXXXXXXXXXXXXXXXXXXXXXXXXXXXXXXXXXXXXXXXXXXXXXXXXXXXXXXXXXXXXXXXXXXXXXXX
XXXXXXXXXXXXXXXXXXXXXXXXXXXXXXXXXXXXXXXXXXXXXXXXXXXXXXXXXXXXXXXXXXXXXXXXXXXXXXXXXXXXXXXXXXXXXXXXXXXXXXXXXXXXXXXXXXXXXXXXXXXXXXXXXXXXXXXXXXXXXXXXXXXXXXXXXXXXXXXXXXXXXXXXXXXXXXXXXXX
XXXXXXXXXXXXXXXXXXXXXXXXXXXXXXXXXXXXXXXXXXXXXXXXXXXXXXXXXXXXXXXXXXXXXXXXXXXXXXXXXXXXXXXXXXXXXXXXXXXXXXXXXXXXXXXXXXXXXXXXXXXXXXXXXXXXXXXXXXXXXXXXXXXXXXXXXXXXXXXXXXXXXXXXXXXXXXXXXX
XXXXXXXXXXXXXXXXXXXXXXXXXXXXXXXXXXXXXXXXXXXXXXXXXXXXXXXXXXXXXXXXXXXXXXXXXXXXXXXXXXXXXXXXXXXXXXXXXXXXXXXXXXXXXXXXXXXXXXXXXXXXXXXXXXXXXXXXXXXXXXXXXXXXXXXXXXXXXXXXXXXXXXXXXXXXXXXXXXXX
XXXXXXXXXXXXXXXXXXXXXXXXXXXXXXXXXXXXXXXXXXXXXXXXXXXXXXXXXXXXXXXXXXXXXXXXXXXXXXXXX000000000000000000000000000XXXXXXXXXXXXXXXXXXXXXXXXXXXXXXXXXXXXXXXXXXXXXXXXXXXXXXXXXXXXXXXXXXXXXXXX
XXXXXXXXXXXXXXXXXXXXXXXXXXXXXXXXXXXXXXXXXXXXXXXXXXXXXXXXXXXXXXXXXXXXXXXXXXXXXXXXXXXXXXXXXXXXXXXXXXXXXXXXXXXXXXXXXXXXXXXXXXXXXXXXXXXXXXXXXXXXXXXXXXXXXXXXXXXXXXXXXXXXXXXXXXXXXXXXXXXX
XXXXXXXXXXXXXXXXXXXXXXXXXXXXXXXXXXXXXXXXXXXXXXXXXXXXXXXXXXXXXXXXXXXXXXXX
>Q68DK7
MTMRSAVFKAAAAPAGGNPEQRLDYERAAALGGPEDEPGAAEAHFLPRHRKLKEPGPPLASSQGGSPAPSPAGCGGKGRGLLLPAGAAPGQQEESWGGSVPLPCPPPATKQAGIGGEPAAAGAGCSPRPKYQAVLPIQTGSLVAAAKEPTPWAGDKGGAASP
AATASDPAGPPPLPLPGPPPLAPTATAGTLAASEGRWKSMRKSPLGGGGGSGASSQAACLKQILLLQLDLIEQQQQQLQAKEKEIEELKSERDTLLARIERMERRMQLVKKDNEKERHKLFQGYETEEREETELSEKIKLECQPELSETSQTLPPKPFSCGRSGKGHKR
KSPFGSTERKTPVKKLAPEFSKVKTKTPKHSPIKEEPCGSLSETVCKRELRSQETPEKPRSSVDTPPRLSTPQKGPSTHPKEKAFSSEIEDLPYLSTTEMYLCRWHQPPPSPLPLRESSPKKEETVARCLMPSSVAGETSVLAVPSWRDHSVEPLRDPNPSDLLENLDDSVF
SKRHAKLELDEKRRKRWDIQRIREQRILQRLQLRMYKKKGIQESEPEVTSFFPEPDDVESLMITPFLPVVAFGRPLPKLTPQNFELPWLDERSRCRLEIQKKQTPHRTCRK
00000000000000000000000000000000000000000000000000000000000000000000000000000000000000000000000000000000000000000000000000000000000000000000000000000000000000000000000000000000
00000000000000000000000000000000000000000000000000000000000000000000000000000000000000000000000000000000000000000000000000000000000000000000000000000000000000000000000000000000
00000000000000000000000000000000000000000000000000000000000000000000000000000000000000000000000000000000000000000000000000000000000000000000000000000000000000000000000000000000
00000000000000000000000000111111111111000000000000000000000000000000000000000000000000
00000000000000000000000000000000000000000000000000000000000000000000000000000000000000000000000000000000000000000000000000000000000000000000000000000000000000000000000000000000
00000000000000000000000000000000000000000000000000000000000000000000000000000000000000000000000000000000000000000000000000000000000000000000000000000000000000000000000000000000
00000000000000000000000000000000000000000000000000000000000000000000000000000000000000000000000000000000000000000000000000000000000000000000000000000000000000000000000000000000
00000000000000000000000000000000000000000000000000000000000000000000000000000000000000
00000000000000000000000000000000000000000000000000000000000000000000000000000000000000000000000000000000000000000000000000000000000000000000000000000000000000000000000000000000
00000000000000000000000000000000000000000000000000000000000000000000000000000000000000000000000000000000000000000000000000000000000000000000000000000000000000000000000000000000
00000000000000000000000000000000000000000000000000000000000000000000000000000000000000000000000000000000000000000000000000000000000000000000000000000000000000000000000000000000
00000000000000000000000000000000000000000000000000000000000000000000000000000000000000
>Q8N5Y2
MSASEGMKFKFHSGEKVLCFEPDPTKARVLYDAKIVDVIVGKDEKGRKIPEYLIHFNGWNRSWDRWAAEDHVLRDTDENRRLQRKLARKAVARLRSTGRKKKRCRLPGVDSVLKGLPTEEKDENDENSLSSSSDCSENKDEEISEESDIEEKTEVKEEPELQTRREME
ERTITIEIPEVLKKQLEDDCYYINRRKRLVKLPCQTNIITILESYVKHFAINAAFSANERPRHHHVMPHANMNVHYIPAEKNVDLCKEMVDGLRITFDYTLPLVLLYPYEQAQYKKVTSSKFFLPIKESATSTNRSQEELSPSPPLLNPSTPQSTESQPTTGEPATPKRRKAEP
EALQSLRRSTRHSANCDRLSESSASPQPKRRQQDTSASMPKLFLHLEKKTPVHSRSSSPIPLTPSKEGSAVFAGFEGRRTNEINEVLSWKLVPDNYPPGDQPPPPSYIYGAQHLLRLFVKLPEILGKMSFSEKNLKALLKHFDLFLRFLAEYHDDFFPESAYVAACEAHYS
TKNPRAIY
00000000000000000000000000000000000000000000000000000000000000000000000000000000000000000000000000000000000000000000000000000000000000000000000000000000000000000000000000000000
00000000000000000000000000000000000000000000000000111111111111111111100000000000000000000000000000000000000000000000000000000000000000000000000000000000000000000000000000000000
0000000000000000000000000000000000000000000000000000000000000000000000000000000000000000000000000000000000000000000000000000000000000000000000000000000000000000000000000
00000000000000000000000000000000000000000000000000000000000000000000000000000000000000000000000000000000000000000000000000000000000000000000000000000000000000000000000000000000
00000000000000000000000000000000000000000000000000000000000000000000000000000000000000000000000000000000000000000000000000000000000000000000000000000000000000000000000000000000
0000000000000000000000000000000000000000000000000000000000000000000000000000000000000000000000000000000000000000000000000000000000000000000000000000000000000000000000000
00000000000000000000000000000000000000000000000000000000000000000000000000000000000000000000000000000000000000000000000000000000000000000000000000000000000000000000000000000000
00000000000000000000000000000000000000000000000000000000000000000000000000000000000000000000000000000000000000000000000000000000000000000000000000000000000000000000000000000000
0000000000000000000000000000000000000000000000000000000000000000000000000000000000000000000000000000000000000000000000000000000000000000000000000000000000000000000000000
>Q9VZU1
MHSEKHTPDIRELMPVREHRCERCPSLVFGNLSHYQLHLRRRHQEVIPPSVIGPIVAFHCPVEKCIYHVATKGARSFTSLRLLRQHYQKSHLDKNYKCLACGGKFLLQHHLEKHQCSKHKCPVCELTYNSKAGLRTHMRRKNHLVHHESDKVPIPSLATWKRLNPQPI
PVSADSLTAHSLKTGSDLHPSEEYINNLPSNLAGVTELYAEIPNPEANMPSTLELDTNLPAAEEHILCFLPIMDVSYALEMSSQKLDMETQTEEDDLNEIRNEVLAPLLRDIETQTPDTRGDIGTMTDDFPEEQEPVAVGSHFHAYSETEPMFDLQTSAHMYTQTCDD
LFEELGLSHIQTQTHWPDGLYNTQHTQTCDEIMDELFPDNFQSTCTQTRWLD
XXXXXXXXXXXXXXXXXXXXXXXXXXXXXXXXXXXXXXXXXXXXXXXXXXXXXXXXXXXXXXXXXXXXXXXXXXXXXXXXXXXXXXXXXXXXXXXXXXXXXXXXXXXXXXXXXXXXXXXXXXXXXXXXXXXXXXXXXXXXXXXXXXXXXXXXXXXXXXXXXXXXXXXXXXXXXXXXXXXXXXXX
XXXXXXXXXXXXXXXXXXXXXXXXXXXXXXXXXXXXXXXXXXXXXXXXXXXXXXXXXXXXXXXXXXXXXXXXXXXXXX111111111111111111111111111111111111111111111111111111111111111111111111111111111111111111111111111111111111111111111
11111111111111111111111111111111111111111
XXXXXXXXXXXXXXXXXXXXXXXXXXXXXXXXXXXXXXXXXXXXXXXXXXXXXXXXXXXXXXXXXXXXXXXXXXXXXXXXXXXXXXXXXXXXXXXXXXXXXXXXXXXXXXXXXXXXXXXXXXXXXXXXXXXXXXXXXXXXXXXXXXXXXXXXXXXXXXXXXXXXXXXXXXXXXXXXXXXXXXXXX
XXXXXXXXXXXXXXXXXXXXXXXXXXXXXXXXXXXXXXXXXXXXXXXXXXXXXXXXXXXXXXXXXXXXXXXXXXXXXX111111111111111111111111111111111111111111111111111111111111111111111111111111111111111111111111111111111111111111111
11111111111111111111111111111111111111111
XXXXXXXXXXXXXXXXXXXXXXXXXXXXXXXXXXXXXXXXXXXXXXXXXXXXXXXXXXXXXXXXXXXXXXXXXXXXXXXXXXXXXXXXXXXXXXXXXXXXXXXXXXXXXXXXXXXXXXXXXXXXXXXXXXXXXXXXXXXXXXXXXXXXXXXXXXXXXXXXXXXXXXXXXXXXXXXXXXXXXXXXX
XXXXXXXXXXXXXXXXXXXXXXXXXXXXXXXXXXXXXXXXXXXXXXXXXXXXXXXXXXXXXXXXXXXXXXXXXXXXXX000000000000000000000000000000000000000000000000000000000000000000000000000000000000000000000000000000000000000000000
00000000000000000000000000000000000000000
>O43313
MAASEAAAAAGSAALAAGARAVPAATTGAAAAASGPWVPPGPRLRGSRPRPAGATQQPAVPAPPAGELIQPSVSELSRAVRTNILCTVRGCGKILPNSPALNMHLVKSHRLQDGIVNPTIRKDLKTGPKFYCCPIEGCPRGPERPFSQFSLVKQHFMKMHAEKKH
KCSKCSNSYGTEWDLKRHAEDCGKTFRCTCGCPYASRTALQSHIYRTGHEIPAEHRDPPSKKRKMENCAQNQKLSNKTIESLNNQPIPRPDTQELEASEIKLEPSFEDSCGSNTDKQTLTTPPRYPQKLLLPKPKVALVKLPVMQFSVMPVFVPTADSSAQPVVLGVD
QGSATGAVHLMPLSVGTLILGLDSEACSLKESLPLFKIANPIAGEPISTGVQVNFGKSPSNPLQELGNTCQKNSISSINVQTDLSYASQNFIPSAQWATADSSVSSCSQTDLSFDSQVSLPISVHTQTFLPSSKVTSSIAAQTDAFMDTCFQSGGVSRETQTSGIESPTDD
HVQMDQAGMCGDIFESVHSSYNVATGNIISNSLVAETVTHSLLPQNEPKTLNQDIEKSAPIINFSAQNSMLPSQNMTDNQTQTIDLLSDLENILSSNLPAQTLDHRSLLSDTNPGPDTQLPSGPAQNPGIDFDIEEFFSASNIQTQTEESELSTMTTEPVLESLDIETQT
DFLLADTSAQSYGCRGNSNFLGLEMFDTQTQTQTDLNFFLDSSPHLPLGSILKHSSFSVSTDSSDTETQTEGVSTAKNIPALESKVQLNSTETQTMSSGFETLGSLFFTSNETQTAMDDFLLADLAWNTMESQFSSVETQTSAEPHTVSNF
00000000000000000000000000000000000000000000000000000000000000000000000000000000000000000000000000000000000000000000000000000000000000000000000000000000000000000000000000000000
00000000000000000000000000000000000000000000000000000000000000000000000000000000000000000000000000000000000000000000000000000000000000000000000000000000000000000000000000000000
00000000011111111111111111111111111111111111111111111111111111111111111111111111111111111111111111111111111111111111111111111111111111111111111111111111111111111111111111111111
11111111111111111111111111111111111111111111111111111111111111111111111111111111111111111111111111111111111111111111111111111111111111111111111111111111111111111111111111111111
11111111111111111111111111111111111111111111111111111111111111111111111111111111111111111111111111111111111111111

00000000000000000000000000000000000000000000000000000000000000000000000000000000000000000000000000000000000000000000000000000000000000000000000000000000000000000000000000000
00000000000000000000000000000000000000000000000000000000000000000000000000000000000000000000000000000000000000000000000000000000000000000000000000000000000000000000000000000
00000000011111111111111111111111111111111111111111111111111111111111111111111111111111111111111111111111111111111111111111111111111111111111111111111111111111111111111111111111
11111111111111111111111111111111111111111111111111111111111111111111111111111111111111111111111111111111111111111111111111111111111111111111111111111111111111111111111111111111
11111111111111111111111111111111111111111111111111111111111111111111111111111111111111111111111111111111111111111111111
00000000000000000000000000000000000000000000000000000000000000000000000000000000000000000000000000000000000000000000000000000000000000000000000000000000000000000000000000000
00000000000000000000000000000000000000000000000000000000000000000000000000000000000000000000000000000000000000000000000000000000000000000000000000000000000000000000000000000
00000000000000000000000000000000000000000000000000000000000000000000000000000000000000000000000000000000000000000000000000000000000000000000000000000000000000000000000000000
00000000000000000000000000000000000000000000000000000000000000000000000000000000000000000000000000000000000000000000000000000000000000000000000000000000000000000000000000000
00000000000000000000000000000000000000000000000000000000000000000000000000000000000000000000000000000000000000000000000
>Q9KMA5
MSNRDLFAELSSALVEAKQHSEGKLTLKTHHVNDVGELNISPDEIVSIREQFNMSRGVFARLLHTSSRTLENWEQGRSVPNGQAVTLLKLVQRHPETLSHIAEL
11111111111111111111111111111111110000000000000000000000000000000000000000000000000000000000000000000000
11111111111111111111111111111111110000000000000000000000000000000000000000000000000000000000000000000000
00000000000000000000000000000000000000000000000000000000000000000000000000000000000000000000000000000000
>Q9KMA6
MKSVFVESTIFEKYRDEYLSDEEYRLFQAELMLNPKLGDVIQGTGGLRKIRVASKGKGKRGGSRIIYYFLDEKRRFYLLTIYGKNEMSDLNANQRKQLMAFMEAWRNEQS
00000000000000000000000000000000000000000000000000001111111111000000000000000000000000000000000000000000000000
00000000000000000000000000000000000000000000000000000000000000000000000000000000000000000000000000000000000000
00000000000000000000000000000000000000000000000000000000000000000000000000000000000000000000000000000000000000
>Q96273
MQSAKEKISDMASTAKEKLNIGGAKAQGHAEKTMARTKKEKKLAQEREKSKEAQAKADLHQSKAEHAADAQVHGHHLPGHSTYPTRATGANYPPGQI
1111111111111111111111111111111111111111111111111111111111111111111111111111111111111111111111111
0000000000000000000000000000000000000000000000000000000000000000000000000000000000000000000000000
0000000000000000000000000000000000000000000000000000000000000000000000000000000000000000000000000
>Q9SLJ2
MAGLINKIGDALHIGGGNKEGEHKKEEEHKKHVDEHKSGEHKEGIVDKIKDKIHGGEGKSHDGEGKSHDGEKKKKKDKKEKKHHDDGHHSSSSDSDSD
11111111111111111111111111111111111111111111111111111111111111111111111111111111111111111111111111
00000000000000000000000000000000000000000000000000000000000000000000000000000000000000000000000000
00000000000000000000000000000000000000000000000000000000000000000000000000000000000000000000000000
>O14519
MSYKPNLAAHMPAAALNAAGSVHSPSTSMATSSQYRQLLSDYGPPSLGYTQGTGNSQVPQSKYAELLAIIEELGKEIRPTYAGSKSAMERLKRGIIHARGLVRECLAETERNARS
111111111111111111111111111111111111111111111111111111111111XXXXXXXXXXXXXXXXXXXXXXXXXXXXXXXXXXXXXXXXXXXXXXXXXXXXXXX
000000000000000000000000000000000000000000000000000000000000XXXXXXXXXXXXXXXXXXXXXXXXXXXXXXXXXXXXXXXXXXXXXXXXXXXXXXX
000000000000000000000000000000000000000000000000000000000000XXXXXXXXXXXXXXXXXXXXXXXXXXXXXXXXXXXXXXXXXXXXXXXXXXXXXXX
>Q99LJ8
MTGLYELVWRVLHALLCLHLTLTSWLRVRFGTWNWIWRRCCRAASAAVLAPLGFTLRKPRAVGRNRRHHRHPHGGPGPGPGPAATHPRLRWRADVRSLQKLPVHMGLLVTEEVQEPSFSDIASLVVWCMAVGISYISVYDHQGIFKRNNSRLMDEILKQQQEL
LGQDCSKYSAEFANSNDKDDQDLNCPSAVKVLSPEDGKADIVRAAQDFCQLVAQQQRKPTDLDVDLLGSLLSSHGFPDPDLVLKFGPVDSTLGFLPWQIRLTEIVSLPSHLNISYEDFFSALRQYAACEQRLGK
XXXXXXXXXXXXXXXXXXXXXXXXXXXXXXXXXXXXXXX11111111111111111111111111111111111111111111111111111111111111111111111111111111110000000000000000000000000000000000000000000000000000000
0000000000000000000000000000000000000000000000000000000000000000000000000000000000000000000000000000000000000000000000000
XXXXXXXXXXXXXXXXXXXXXXXXXXXXXXXXXXXXXXX00000000000000000000000000000000000000000000000000000000000000000000000000000000000000000000000000000000000000000000000000000000000000000
0000000000000000000000000000000000000000000000000000000000000000000000000000000000000000000000000000000000000000000000000
XXXXXXXXXXXXXXXXXXXXXXXXXXXXXXXXXXXXXXX00000000000000000000000000000000000000000000000000000000000000000000000000000000000000000000000000000000000000000000000000000000000000000
0000000000000000000000000000000000000000000000000000000000000000000000000000000000000000000000000000000000000000000000000
>P08392
MASENKQRPGSPGPTDGPPPTPSPDRDERGALGWGAETEEGGDDPDHDPDHPHDLDDARRDGRAPAAGTDAGEDAGDAVSPRQLALLASMVEEAVRTIPTPDPAASPPRTPAFRADDDDGDEYDDAADAAGDRAPARGREREAPLRGAYPDPTDRLSPRP
PAQPPRRRRHGRWRPSASSTSSDSGSSSSSSASSSSSSDEDEDDDGNDAADHAREARAVGRGPSSAAPAAPGRTPPPPGPPPLSEAAPKPRAAARTPAASAGRIERRRARAAVAGRDATGRFTAGQPRRVELDADATSGAFYARYRDGYVSGEPWPGAGPPPP
GRVLYGGLGDSRPGLWGAPEAEEARRRFEASGAPAAVWAPELGDAAQQYALITRLLYTPDAEAMGWLQNPRVVPGDVALDQACFRISGAARNSSSFITGSVARAVPHLGYAMAAGRFGWGLAHAAAAVAMSRRYDRAQKGFLLTSLRRAYAPLLARENAALT
GAAGSPGAGADDEGVAAVAAAAPGERAVPAGYGAAGILAALGRLSAAPASPAGGDDPDAARHADADDDAGRRAQAGRVAVECLAACRGILEALAEGFDGDLAAVPGLAGARPASPPRPEGPAGPASPPPPHADAPRLRAWLRELRFVRDALVLMRLRGDLR
VAGGSEAAVAAVRAVSLVAGALGPALPRDPRLPSSAAAAAADLLFDNQSLRPLLAAAASAPDAADALAAAAASAAPREGRKRKSPGPARPPGGGGPRPPKTKKSGADAPGSDARAPLPAPAPPSTPPGPEPAPAQPAAPRAAAAQARPRPVAVSRRPAEGPDPL
GGWRRQPPGPSHTAAPAAAALEAYCSPRAVAELTDHPLFPVPWRPALMFDPRALASIAARCAGPAPAAQAACGGGDDDDNPHPHGAAGGRLFGPLRASGPLRRMAAWMRQIPDPEDVRVVVLYSPLPGEDLAGGGASGGPPEWSAERGGLSCLLAALANR
LCGPDTAAWAGNWTGAPDVSALGAQGVLLLSTRDLAFAGAVEFLGLLASAGDRRLIVVNTVRACDWPADGPAVSRQHAYLACELLPAVQCAVRWPAARDLRRTVLASGRVFGPGVFARVEAAHARLYPDAPPLRLCRGGNVRYRVRTRFGPDTPVPMSPREY
RRAVLPALDGRAAASGTTDAMAPGAPDFCEEEAHSHAACARWGLGAPLRPVYVALGREAVRAGPARWRGPRRDFCARALLEPDDDAPPLVLRGDDDGPGALPPAPPGIRWASATGRSGTVLAAAGAVEVLGAEAGLATPPRREVVDWEGAWDEDDGGAFE
GDGVL
XXXXXXXXXXXXXXXXXXXXXXXXXXXXXXXXXXXXXXXXXXXXXXXXXXXXXXXXXXXXXXXXXXXXXXXXXXXXXXXXXXXXXXXXXXXXXXXXXXXXXXXXXXXXXXXXXXXXXXXXXXXXXXXXXXXXXXXXXXXXXXXXXXXXXXXXXXXXXXXXXXXXXXX
XXXXXXXXXXXXXXXXXXXXXXXXXXXXXXXXXXXXXXXXXXXXXXXXXXXXXXXXXXXXXXXXXXXXXXXXXXXXXXXXXXXXXXX11111111111111111111111111110000000000000000000000000000000000000000000000000000000000000
00000000000000000000000000000000000000000000000000000000000000000000000000000000000000000000000000000000000000000000000000000000000000000000000000000000000000000000000000000
00000000000000000000000000000000000000000000000000000000000000000000000000000000000000000000000000000000000000000000000000000000000000000000000000000000000000000000000000000
00000000000000000000000000000000000000000000000000000000000000000000000000000000000000000000000000000000000000000000000000000000000000000000000000000000000000000000000000000
00000000000000000000000000000000000000000000000000000000000000000000000000000000000000000000000000000000000000000000000000000000000000000000000000000000000000000000000000000
00000000000000000000000000000000000000000000000000000000000000000000000000000000000000000000000000000000000000000000000000000000000000000000000000000000000000000000000000000
000000000000000000000000000000000000000000000000000000000000000000000000
XXXXXXXXXXXXXXXXXXXXXXXXXXXXXXXXXXXXXXXXXXXXXXXXXXXXXXXXXXXXXXXXXXXXXXXXXXXXXXXXXXXXXXXXXXXXXXXXXXXXXXXXXXXXXXXXXXXXXXXXXXXXXXXXXXXXXXXXXXXXXXXXXXXXXXXXXXXXXXXXXXXXXXXXXX
XXXXXXXXXXXXXXXXXXXXXXXXXXXXXXXXXXXXXXXXXXXXXXXXXXXXXXXXXXXXXXXXXXXXXXXXXXXXXXXXXXXXXXX00000000000000000000000000000000000000000000000000000000000000000000000000000000000000000
00000000000000000000000000000000000000000000000000000000000000000000000000000000000000000000000000000000000000000000000000000000000000000000000000000000000000000000000000000
00000000000000000000000000000000000000000000000000000000000000000000000000000000000000000000000000000000000000000000000000000000000000000000000000000000000000000000000000000
00000000000000000000000000000000000000000000000000000000000000000000000000000000000000000000000000000000000000000000000000000000000000000000000000000000000000000000000000000
00000000000000000000000000000000000000000000000000000000000000000000000000000000000000000000000000000000000000000000000000000000000000000000000000000000000000000000000000000
00000000000000000000000000000000000000000000000000000000000000000000000000000000000000000000000000000000000000000000000000000000000000000000000000000000000000000000000000000
000000000000000000000000000000000000000000000000000000000000000000000000
XXXXXXXXXXXXXXXXXXXXXXXXXXXXXXXXXXXXXXXXXXXXXXXXXXXXXXXXXXXXXXXXXXXXXXXXXXXXXXXXXXXXXXXXXXXXXXXXXXXXXXXXXXXXXXXXXXXXXXXXXXXXXXXXXXXXXXXXXXXXXXXXXXXXXXXXXXXXXXXXXXXXXXXXXX
XXXXXXXXXXXXXXXXXXXXXXXXXXXXXXXXXXXXXXXXXXXXXXXXXXXXXXXXXXXXXXXXXXXXXXXXXXXXXXXXXXXXXXX11111111111111111111111111110000000000000000000000000000000000000000000000000000000000000
00000000000000000000000000000000000000000000000000000000000000000000000000000000000000000000000000000000000000000000000000000000000000000000000000000000000000000000000000000
00000000000000000000000000000000000000000000000000000000000000000000000000000000000000000000000000000000000000000000000000000000000000000000000000000000000000000000000000000
00000000000000000000000000000000000000000000000000000000000000000000000000000000000000000000000000000000000000000000000000000000000000000000000000000000000000000000000000000
00000000000000000000000000000000000000000000000000000000000000000000000000000000000000000000000000000000000000000000000000000000000000000000000000000000000000000000000000000
00000000000000000000000000000000000000000000000000000000000000000000000000000000000000000000000000000000000000000000000000000000000000000000000000000000000000000000000000000
000000000000000000000000000000000000000000000000000000000000000000000000
>Q8WUP2
MASKPEKRVASSVFITLAPPRRDVAVAEEVRQAVCEARRGRPWEAPAPMKTPEAGLAGRPSPWTTPGRAAATVPAAPMQLFNGGCPPPPPVLDGEDVLPDLDLLPPPPPPPPVLLPSEEEAPAPMGASLIADLEQLHLSPPPPPPQAPAEGPSVQPGPLRPMEEE
LPPPPAEPVEKGASTDICAFCHKTVSPRELAVEAMKRQYHAQCFTCRTCRRQLAGQSFYQKDGRPLCEPCYQDTLERCGKCGEVVRDHIIRALGQAFHPSCFTCVTCARCIGDESFALGSQNEVYCLDDFYRKFAPVCSICENPIIPRDGKDAFKIECMGRNFHENCY
RCEDCRILLSVEPTDQGCYPLNNHLFCKPCHVKRSAAGCC
11111111111111111111111111111111111111111111111111111111111111111111111111111111111111111111111111000000000000000000000000000000000000000000000000000000000000000000000000000000
00000000000000000000000000000000000000000000000000000000000000000000000000000000000000000000000000000000000000000000000000000000000000000000000000000000000000000000000000000
000000000000000000000
00001111111111111000000000000000000000000000000000000000000000000000000000000000000000000000000000000000000000000000000000000000000000000000000000000000000000000000000000000
00000000000000000000000000000000000000000000000000000000000000000000000000000000000000000000000000000000000000000000000000000000000000000000000000000000000000000000000000000
000000000000000000000
00000000000000000000000000000000000000000000000000000000000000000000000000000000000000000000000000000000000000000000000000000000000000000000000000000000000000000000000000000
00000000000000000000000000000000000000000000000000000000000000000000000000000000000000000000000000000000000000000000000000000000000000000000000000000000000000000000000000000
000000000000000000000
>Q9UMX1
MAELRPSGAPGPTAPPAPGPTAPPAFASLFPPGLHAIYGECRRLYPDQPNPLQVTAIVKYWLGGPDPLDYVSMYRNVGSPSANIPEHWHYISFGLSDLYGDNRVHEFTGTDGPSGFGFELTFRLKRETGESAPPTWPAELMQGLARYVFQSENTFCSGDHVSWHS
PLDNSESRIQHMLLTEDPQMQPVQTPFGVVTFLQIVGVCTEELHSAQQWNGQGILELLRTVPIAGGPWLITDMRRGETIFEIDPHLQERVDKGIETDGSNLSGVSAKCAWDDLSRPPEDDEDSRSICIGTQPRRLSGKDTEQIRETLRRGLEINSKPVLPPINPQRQN
GLAHDRAPSRKDSLESDSSTAIIPHELIRTRQLESVHLKFNQESGALIPLCLRGRLLHGRHFTYKSITGDMAITFVSTGVEGAFATEEHPYAAHGPWLQILLTEEFVEKMLEDLEDLTSPEEFKLPKEYSWPEKKLKVSILPDVVFDSPLH
00000000000000000000000000000000000000000000000000000000000000000000000000000000000000000000000000000000000000000000000000000000000000000000000000000000000000000000000000000
00000000000000000000000000000000000000000000000000000000000000000000000000000000000000000111111111111111111111111111111111111111111111111111111111111111111111111111111111111111
11111110000000000000000000000000000000000000000000000000000000000000000000000000000000000000000000000000000000000000000000000000000
00000000000000000000000000000000000000000000000000000000000000000000000000000000000000000000000000000000000000000000000000000000000000000000000000000000000000000000000000000
00000000000000000000000000000000000000000000000000000000000000000000000000000000000000000000000000000000000000000000000000000000000000000000000000000000000000000000000000000
000000000000000000000000000000000000000000000000000000000000000000000000000000000000000000000000000000000000000000000000000000000000
00000000000000000000000000000000000000000000000000000000000000000000000000000000000000000000000000000000000000000000000000000000000000000000000000000000000000000000000000000
00000000000000000000000000000000000000000000000000000000000000000000000000000000000000000000000000000000000000000000000000000000000000000000000000000000000000000000000000000
000000000000000000000000000000000000000000000000000000000000000000000000000000000000000000000000000000000000000000000000000000000000
>Q9N4U5
MADNPTNLRNADFRKLLTSARSDRPAVSAFAKPADPKTGDDKPASFKHKHLKPAKFKKPQAAAHGKAKKEKTEADEDEAALKNILKNYRDRAAERRKQGDEKEDPSKLTAAYRAVPGDARSAQDQADLRKQAILESKYLGGDLEHTHLVKGLDYSLLNKVRSEIDK
SDDDDDDDIDTAFDEKVTSSSSSSKPSEASLLAQELAQSHSENRMVRSLHRVLFKNEVPLHNQLFAKGRMAYVVELEDEETDIPTTLLRSLHDLPRAESAQSIQANNLIILKLSHVLSHLRAEPKKKKKEEFRVQLGSRDAPGAAAAAPGAKGDSIYDDLDDYVPSRKSR
DSRDAGRRGSRRDRSRDRSRDRDRDRDRDNRDRYFEKSANSRREEEQNRREQQREREERAEQERRREREKEREQEKAKEREKKRKELEESSGYDECYPGGLVEMGGAWDSDEEADYSKMDAGPKKNQAVNRWDFDTEEEYASYMEGREALPKAAYQYGVKNG
EGGRKNKKQSAVSDAKRLDRELNEINKIMDKRKAGGDGAGGGGDYKKPKY
XXXXXXXXXXXXXXXXXXXXXXXXXXXXXXXXXXXXXXXXXXXXXXXXXXXXXXXXXXXXXXXXXXXXXXXXXXXXXXXXXXXXXXXXXXXXXXXXXXXXXXXXXXXXXXXXXXXXXXXXXXXXXXXXXXXXXXXXXXXXXXXXXXXXXXXXXXXXXXXXX1111111111
1111111111111111111111111111111111111111111111111100000000000000000000000000000000000000000000000000000000000000000000000000000000000000000000000000000000000000000000000000000
00000000000000000000000000000000000000000000000000000000000000000000000000000000000000000000000000000000000000000000000000000000000000000000000000000000000000000000000000000
00000000000000000000000
XXXXXXXXXXXXXXXXXXXXXXXXXXXXXXXXXXXXXXXXXXXXXXXXXXXXXXXXXXXXXXXXXXXXXXXXXXXXXXXXXXXXXXXXXXXXXXXXXXXXXXXXXXXXXXXXXXXXXXXXXXXXXXXXXXXXXXXXXXXXXXXXXXXXXXXXXXXXXXXXXXXX1111111111
1111111111111111111111111111111111111111111111111100000000000000000000000000000000000000000000000000000000000000000000000000000000000000000000000000000000000000000000000000000
00000000000000000000000000000000000000000000000000000000000000000000000000000000000000000000000000000000000000000000000000000000000000000000000000000000000000000000000000000
00000000000000000000000
XXXXXXXXXXXXXXXXXXXXXXXXXXXXXXXXXXXXXXXXXXXXXXXXXXXXXXXXXXXXXXXXXXXXXXXXXXXXXXXXXXXXXXXXXXXXXXXXXXXXXXXXXXXXXXXXXXXXXXXXXXXXXXXXXXXXXXXXXXXXXXXXXXXXXXXXXXXXXXXXXXXX000000000
00000000000000000000000000000000000000000000000000000000000000000000000000000000000000000000000000000000000000000000000000000000000000000000000000000000000000000000000000000
00000000000000000000000000000000000000000000000000000000000000000000000000000000000000000000000000000000000000000000000000000000000000000000000000000000000000000000000000000
00000000000000000000000
>Q9QWH1
MENELPVPHTSNRASVTTNTSGTNSSSGCISSSGGGGGSGGRPTAPQISVYSGIPDRQTVQVIQQALHRQPSTAAQYLQQMYAAQQQHLMLQTAALQQQHLSSAQLQSLAAVQQASLVANRQGSTPGSSVSSQAPAQSSSLNLAASPAAAQLINRAQSVNSAA
ASGLAQQAVLLGNTSSPALTASQAQMYLRAQMLIFTPTATVATVQPELCTGSPARPPTPAQVQNLTLRTQQTPAAAASGPPPTQPVLPSLALKPTPSSSQPLPAPPQGRTMAQGSPAGAKPSGTDNAPETLKAGDGNCNMEGRPGPGRAVPAVATHPLIAPAY
AHLQSHQLLPQPPAKHPQPQFVAQQQPQPPRPAPQVQSQPQLASVSPSLALQSSPEDHALPLGSVTQALPLQCSTTHVHKPGNSQQCHLPTLDTGSQNGHPEGGSHPPQRRFQHTSAVILQVQPASPVTPQQCAPDDWKEVVPAEKSVPVARPGPSPHQQAI
IPAIPGGLPGPKSPNIQQCPAHETGQGIVHALTDLSSPGMTSGNGNSASSIAGTAPQNGENKPPQAIVKPQILTHVIEGFVIQEGAEPFPVGRSSLLVGNLKKKYAQGFLPEKPPQQDHTTTTDSEMEEPYLQESKEEGTPLKLKCELCGRVDFAYKFKRSKRFCSMAC
AKRYNVGCTKRVGLFHSDRSKLQKAGTTTHNRRRASKASLPTLTKDTKKQPSGTVPLSVTAALQLAHSQEDSSRCSDNSSYEEPLSPISASSSTSRRRQGQRDLDLPDMHMRDLVGVGHHFLPSEPTKWNVEDVYEFIRSLPGCQEIAEEFRAQEIDGQALLLLKEDH
LMSAMNIKLGPALKIYARISMLKDS

1111111111111111111111111111111111111111111111111111111111111111111111111111111111111111111111000000000000000000000000000000000000000000000000000000000000000000000000000000000000000000000
00000000000000000000000000000000000000000000000000000000000000000000000000000000000000000000000000000000000000000000000000000000000000000000000000000000000000000000000000000000000000000
00000000000000000000000000000000000000000000000000000000000000000000000000000000000000000000000000000000000000000000000000000000000000000000000000000000000000000000000000000000000000000
00000000000000000000000000000000000000000000000000000000000000000000000000000000000000000000000000000000000000000000000000000000000000000000000000000000000000000000000000000000000000000
000000000000000000000000000000000000000000000000000000000000000000000000000000000000000000000000000000000000000000000000000000000000000000000000000000000
1111111111111111111111111111111111111111111111111111111111111111111111111111111111111111111111000000000000000000000000000000000000000000000000000000000000000000000000000000000000000000000
00000000000000000000000000000000000000000000000000000000000000000000000000000000000000000000000000000000000000000000000000000000000000000000000000000000000000000000000000000000000000000
00000000000000000000000000000000000000000000000000000000000000000000000000000000000000000000000000000000000000000000000000000000000000000000000000000000000000000000000000000000000000000
00000000000000000000000000000000000000000000000000000000000000000000000000000000000000000000000000000000000000000000000000000000000000000000000000000000000000000000000000000000000000000
000000000000000000000000000000000000000000000000000000000000000000000000000000000000000000000000000000000000000000000000000000000000000000000000000000000
00000000000000000000000000000000000000000000000000000000000000000000000000000000000000000000000000000000000000000000000000000000000000000000000000000000000000000000000000000000000000000
00000000000000000000000000000000000000000000000000000000000000000000000000000000000000000000000000000000000000000000000000000000000000000000000000000000000000000000000000000000000000000
00000000000000000000000000000000000000000000000000000000000000000000000000000000000000000000000000000000000000000000000000000000000000000000000000000000000000000000000000000000000000000
00000000000000000000000000000000000000000000000000000000000000000000000000000000000000000000000000000000000000000000000000000000000000000000000000000000000000000000000000000000000000000
000000000000000000000000000000000000000000000000000000000000000000000000000000000000000000000000000000000000000000000000000000000000000000000000000000000
>Q01924
MNNKMFLNKEAGFLAHTKRKRRFAVTLVGVFFMLLASAGAIGFGQVAYAADEKTVPHRVSQNPEFPWYGYDFYKGPYTRYHNLQLNLNGSKTYQAYCFNLKRFEPKKEGSYFPNWYKRWDGSEETFVKYADNPRKDNESSRVIDVELEKNILRVLYNGYPNNGN
GIMEGLEPLNAILVTQNAVWYYSDNSSIFNTDNFFTTEAKDLNIKPEQLSLMRVALKKLIDPKLSEESLKPVPSTFRLNIFESQDKLYQNLLSAEFVPENPPKPGETPEHGPKTPELDGTPIPEGPQRPNESLEPTLPPVMLDGQEVPEVPSESLEPALPPLMPELDGQEV
PEVPSESLEPALPPLMPELDGQEVPEKPSVDLPIEDPRYEFNNKDQSPLAGESGETEYITEVYGNQQNPVDIDKKLPNETGFSGNMVETEDTKEPGVLMGGQSESVEFTKDTQTGMSGQTTPQVETEDTKEPGVLMGGQSESVEFTKDTQTGMSGQTASQVETE
DTKEPGVLMGGQSESVEFTKDTQTGMSGQTTPQVETEDTKEPGVLMGGQSESVEFTKDTQTGMSGFSETVTIVEDTRPKLVFHFDNNEPKVEENREKPTKNITPILPATGDIENVLAFLGILILSVLPIFSLLKKQTKQ
00000000000000000000000000000000000000000000000000000000000000000000000000000000000000000000000000000000000000000000000000000000000000000000000000000000000000000000000000000000000000000
00000000000000000000000000000000000000000000000000000000000000000000000000000000000000000000000000000000000000000000000000000000000000000000000000000000000000000000000000000000000000000
00000000000000000000000000000000000000000000000000000000000000000000111111111111111111111111111111111111111111111111111111111111111111111111111111111111111111111111111111111111111111111
111111111111111111111111111111111111111111111111111111111111111111XXXXXXXXXXXXXXXXXXXXXXXXXXXXXXXXXXXXXXXXXXXXXXXXXX
00000000000000000000000000000000000000000000000000000000000000000000000000000000000000000000000000000000000000000000000000000000000000000000000000000000000000000000000000000000000000000
00000000000000000000000000000000000000000000000000000000000000000000000000000000000000000000000000000000000000000000000000000000000000000000000000000000000000000000000000000000000000000
00000000000000000000000000000000000000000000000000000000000000000000000000000000000000000000000000000000000000000000000000000000000000000000000000000000000000000000000000000000000000000
0000000000000000000000000000000000000000000000000000000000000000000XXXXXXXXXXXXXXXXXXXXXXXXXXXXXXXXXXXXXXXXXXXXXXXXX
00000000000000000000000000000000000000000000000000000000000000000000000000000000000000000000000000000000000000000000000000000000000000000000000000000000000000000000000000000000000000000
00000000000000000000000000000000000000000000000000000000000000000000000000000000000000000000000000000000000000000000000000000000000000000000000000000000000000000000000000000000000000000
00000000000000000000000000000000000000000000000000000000000000000000000000000000000000000000000000000000000000000000000000000000000000000000000000000000000000000000000000000000000000000
0000000000000000000000000000000000000000000000000000000000000000000XXXXXXXXXXXXXXXXXXXXXXXXXXXXXXXXXXXXXXXXXXXXXXXXX
>Q8IV16
MKALGAVLLALLLCGRPGRGQTQQEEEEEDEDHGPDDYDEEDEDEVEEEETNRLPGGRSRVLLRCYTCKSLPRDERCNLTQNCSHGQTCTTLIAHGNTESGLLTTHSTWCTDSCQPITKTVEGTQVTMTCCQSSLCNVPPWQSSRVQDPTGKGAGGPRGSSETVG
AALLLNLLAGLGAMGARRP
XXXXXXXXXXXXXXXXXXXXXXXXXXXXXX111111111111111111111111111111110000000000000000000000000000000000000000000000000000000000000000000000000000000000000000000000000000000000000000000000000000
00000000
XXXXXXXXXXXXXXXXXXXXXXXXXXXXXXX0000000000000000000000000000000000000000000000000000000000000000000000000000000000000000000000000000000000000000000000000000000000000000000000000000000000000
00000000
XXXXXXXXXXXXXXXXXXXXXXXXXXXXXXX0000000000000000000000000000000000000000000000000000000000000000000000000000000000000000000000000000000000000000000000000000000000000000000000000000000000000
00000000
>Q9C0C7
MKVVPEKNAVRILWGRERGARAMGAQRLLQELVEDKTRWMKWEGKRVELPDSPRSTFLLAFSPDRTLLASTHVNHNIYITEVKTGKCVHSLIGHRRTPWCVTFHPTISGLIASGCLDGEVRIWDLHGGSESWFTDSNNAIASLAFHPTAQLLLIATANEIHFWDWS
RREPFAVVKTASEMERVRLVRFDPLGHYLLTAIVNPSNQQGDDEPEIPIDGTELSHYRQRALLQSQPVRRTPLLHNFLHMLSSRSSGIQVGEQSTVQDSATPSPPPPPPQPSTERPRTSAYIRLRQRVSYPTAECCQHLGILCLCSRCSGTRVPSLLPHQDSVPPASARA
TTPSFSFVQTEPFHPPEQASSTQQDQGLLNRPSAFSTVQSSTAGNTLRNLSLGPTRRSLGGPLSSHPSRYHREIAPGLTGSEWTRTVLSLNSRSEAESMPPPRTSASSVSLLSVLRQQEGGSQASVYTSATEGRGFPASGLATESDGGNGSSQNNSGSIRHELQCDLRR
FFLEYDRLQELDQSLSGEAPQTQQAQEMLNNNIESERPGPSHQPTPHSSENNSNLSRGHLNRCRACHNLLTFNNDTLRWERTTPNYSSGEASSSWQVPSSFESVPSSGSQLPPLERTEGQTPSSSRLELSSSASPQEERTVGVAFNQETGHWERIYTQSSRSGTVSQ
EALHQDMPEESSEEDSLRRRLLESSLISLSRYDGAGSREHPIYPDPARLSPAAYYAQRMIQYLSRRDSIRQRSMRYQQNRLRSSTSSSSSDNQGPSVEGTDLEFEDFEDNGDRSRHRAPRNARMSAPSLGRFVPRRFLLPEYLPYAGIFHERGQPGLATHSSVNRVLAG
AVIGDGQSAVASNIANTTYRLQWWDFTKFDLPEISNASVNVLVQNCKIYNDASCDISADGQLLAAFIPSSQRGFPDEGILAVYSLAPHNLGEMLYTKRFGPNAISVSLSPMGRYVMVGLASRRILLHPSTEHMVAQVFRLQQAHGGETSMRRVFNVLYPMPADQR
RHVSINSARWLPEPGLGLAYGTNKGDLVICRPEALNSGVEYYWDQLNETVFTVHSNSRSSERPGTSRATWRTDRDMGLMNAIGLQPRNPATSVTSQGTQTLALQLQNAETQTEREVPEPGTAASGPGEGEGSEYGASGEDALSRIQRLMAEGGMTAVVQREQS
TTMASMGGFGNNIIVSHRIHRSSQTGTEPGAAHTSSPQPSTSRGLLPEAGQLAERGLSPRTASWDQPGTPGREPTQPTLPSSSPVPIPVSLPSAEGPTLHCELTNNNHLLDGGSSRGDAAGPRGEPRNR
00000000000000000000000000000000000000000000000000000000000000000000000000000000000000000000000000000000000000000000000000000000000000000000000000000000000000000000000000000000000000000
00000000000000000000000000000000000000000000000000000000000000000000000000000000000000000000000000000000000000000000000000000000000000000000000000000000000000000000000000000000000000000
00000000000000000000000000000000000000000000000000000000000000000000000000000000000000000000000000000000000000000000000000000000000000000000000000000000000000000000000000000000000000011
1111111111111XXXXXXXXXXXXXXXXXXXXXXXXXXXXXXXXXXXXXXXXXXXXXXXXXXXXXXXXXXXXXXXXXXXXXXXXXXXXXXXXXXXXXXXXXXXXXXXXXXXXXXXXXXXXXXXXXXXXXXXXXXXXXXXXXXXXXXXXXXXXXXXXXXXXXXXXXXXXXXXXX
XXXXXXXXXXXXXXXXXXXXXXXXXXXXXXXXXXXXXXXXXXXXXXXXXXXXXXXXXXXXXXXXXXXXXXXXXXXXXXXXXXXXXXXXXXXXXXXXXXXXXXXXXXXXXXXXXXXXXXXXXXXXXXXXXXXXXXXXXXXXXXXXXXXXXXXXXXXXXXXXXXXXXXXXXXXXXXXXX
XXXXXXXXXXXXXXXXXXXXXXXXXXXXXXXXXXXXXXXXXXXXXXXXXXXXXXXXXXXXXXXXXXXXXXXXXXXXXXXXXXXXXXXXXXXXXXXXXXXXXXXXXXXXXXXXXXXXXXXXXXXXXXXXXXXXXXXXXXXXXXXXXXXXXXXXXXXXXXXXXXXXXXXXXXXXXXXXX
XXXXXXXXXXXXXXXXXXXXXXXXXXXXXXXXXXXXXXXXXXXXXXXXXXXXXXXXXXXXXXXXXXXXXXXXXXXXXXXXXXXXXXXXXXXXXXXXXXXXXXXXXXXXXXXXXXXXXXXXXXXXXXXXXXXXXXXXXXXXXXXXXXXXXXXXXXXXXXXXXXXXXXXXXXXXXXXXX
XXXXXXXXXXXXXXXXXXXXXXXXXXXXXXXXXXXXXXXXXXXXXXXXXXXXXXXXXXXXXXXXXXXXXXXXXXXXXXXXXXXXXXXXXXXXXXXXXXXXXXXXXXXXXXXXXXXXXXXXXXXXXXXXXXXXXXXXXXXXXXXXXXXXXXXXXXXXXXXXXXXXXXXXXXXXXXXXX
XXXXXXXXXXXXXXXXXXXXXXXXXXXXXXXXXXXXXXXXXXXXXXXXXXXXXXXXXXXXXXXXXXXXXXXXXXXXXXXX
00000000000000000000000000000000000000000000000000000000000000000000000000000000000000000000000000000000000000000000000000000000000000000000000000000000000000000000000000000000000000000
00000000000000000000000000000000000000000000000000000000000000000000000000000000000000000000000000000000000000000000000000000000000000000000000000000000000000000000000000000000000000000
00000000000000000000000000000000000000000000000000000000000000000000000000000000000000000000000000000000000000000000000000000000000000000000000000000000000000000000000000000000000000000
0000000000000XXXXXXXXXXXXXXXXXXXXXXXXXXXXXXXXXXXXXXXXXXXXXXXXXXXXXXXXXXXXXXXXXXXXXXXXXXXXXXXXXXXXXXXXXXXXXXXXXXXXXXXXXXXXXXXXXXXXXXXXXXXXXXXXXXXXXXXXXXXXXXXXXXXXXXXXXXXXXXXXX
XXXXXXXXXXXXXXXXXXXXXXXXXXXXXXXXXXXXXXXXXXXXXXXXXXXXXXXXXXXXXXXXXXXXXXXXXXXXXXXXXXXXXXXXXXXXXXXXXXXXXXXXXXXXXXXXXXXXXXXXXXXXXXXXXXXXXXXXXXXXXXXXXXXXXXXXXXXXXXXXXXXXXXXXXXXXXXXXXX
XXXXXXXXXXXXXXXXXXXXXXXXXXXXXXXXXXXXXXXXXXXXXXXXXXXXXXXXXXXXXXXXXXXXXXXXXXXXXXXXXXXXXXXXXXXXXXXXXXXXXXXXXXXXXXXXXXXXXXXXXXXXXXXXXXXXXXXXXXXXXXXXXXXXXXXXXXXXXXXXXXXXXXXXXXXXXXXXX
XXXXXXXXXXXXXXXXXXXXXXXXXXXXXXXXXXXXXXXXXXXXXXXXXXXXXXXXXXXXXXXXXXXXXXXXXXXXXXXXXXXXXXXXXXXXXXXXXXXXXXXXXXXXXXXXXXXXXXXXXXXXXXXXXXXXXXXXXXXXXXXXXXXXXXXXXXXXXXXXXXXXXXXXXXXXXXXX
XXXXXXXXXXXXXXXXXXXXXXXXXXXXXXXXXXXXXXXXXXXXXXXXXXXXXXXXXXXXXXXXXXXXXXXXXXXXXXXXXXXXXXXXXXXXXXXXXXXXXXXXXXXXXXXXXXXXXXXXXXXXXXXXXXXXXXXXXXXXXXXXXXXXXXXXXXXXXXXXXXXXXXXXXXXXXXXX
XXXXXXXXXXXXXXXXXXXXXXXXXXXXXXXXXXXXXXXXXXXXXXXXXXXXXXXXXXXXXXXXXXXXXXXXXXXXXXXX
00000000000000000000000000000000000000000000000000000000000000000000000000000000000000000000000000000000000000000000000000000000000000000000000000000000000000000000000000000000000000000
00000000000000000000000000000000000000000000000000000000000000000000000000000000000000000000000000000000000000000000000000000000000000000000000000000000000000000000000000000000000000000
00000000000000000000000000000000000000000000000000000000000000000000000000000000000000000000000000000000000000000000000000000000000000000000000000000000000000000000000000000000000000000
0000000000000XXXXXXXXXXXXXXXXXXXXXXXXXXXXXXXXXXXXXXXXXXXXXXXXXXXXXXXXXXXXXXXXXXXXXXXXXXXXXXXXXXXXXXXXXXXXXXXXXXXXXXXXXXXXXXXXXXXXXXXXXXXXXXXXXXXXXXXXXXXXXXXXXXXXXXXXXXXXXXXXX
XXXXXXXXXXXXXXXXXXXXXXXXXXXXXXXXXXXXXXXXXXXXXXXXXXXXXXXXXXXXXXXXXXXXXXXXXXXXXXXXXXXXXXXXXXXXXXXXXXXXXXXXXXXXXXXXXXXXXXXXXXXXXXXXXXXXXXXXXXXXXXXXXXXXXXXXXXXXXXXXXXXXXXXXXXXXXXXXX
XXXXXXXXXXXXXXXXXXXXXXXXXXXXXXXXXXXXXXXXXXXXXXXXXXXXXXXXXXXXXXXXXXXXXXXXXXXXXXXXXXXXXXXXXXXXXXXXXXXXXXXXXXXXXXXXXXXXXXXXXXXXXXXXXXXXXXXXXXXXXXXXXXXXXXXXXXXXXXXXXXXXXXXXXXXXXXXX
XXXXXXXXXXXXXXXXXXXXXXXXXXXXXXXXXXXXXXXXXXXXXXXXXXXXXXXXXXXXXXXXXXXXXXXXXXXXXXXXXXXXXXXXXXXXXXXXXXXXXXXXXXXXXXXXXXXXXXXXXXXXXXXXXXXXXXXXXXXXXXXXXXXXXXXXXXXXXXXXXXXXXXXXXXXXXXXX
XXXXXXXXXXXXXXXXXXXXXXXXXXXXXXXXXXXXXXXXXXXXXXXXXXXXXXXXXXXXXXXXXXXXXXXXXXXXXXXXXXXXXXXXXXXXXXXXXXXXXXXXXXXXXXXXXXXXXXXXXXXXXXXXXXXXXXXXXXXXXXXXXXXXXXXXXXXXXXXXXXXXXXXXXXXXXXXX
XXXXXXXXXXXXXXXXXXXXXXXXXXXXXXXXXXXXXXXXXXXXXXXXXXXXXXXXXXXXXXXXXXXXXXXXXXXXXXXX
>P28324
MDSAITLWQFLLQLLQKPQNKHMICWTSNDGQFKLLQAEEVARLWGIRKNKPNMNYDKLSRALRYYYVKNIIKKVNGQKFVYKFVSYPEILNMDPMTVGRIEGDCESLNFSEVSSSSKDVENGGKDKPPQPGAKTSSRNDYIHSGLYSSFTLNSLNSSNVKLFKLIK
TENPAEKLAEKKSPQEPTPSVIKFVTTPSKKPPVEPVAATISIGPSISPSSEETIQALETLVSPKLPSLEAPTSASNVMTAFATTPPISSIPPLQEPPRTPSPPLSSHPDIDTDIDSVASQPMELPENLSLEPKDQDSVLLEKDKVNNSSRSKKPKGLELAPTLVITSSDPSPLGILS
PSLPTASLTPAFFSQTPIILTPSPLLSSIHFWSTLSPVAPLSPARLQGANTLFQFPSVLNSHGPFTLSGLDGPSTPGPFSPDLQKT
0000000000000000000000000000000000000000000000000000000000000000000000000000001111111111111111111111111111111111111111111XXXXXXXXXXXXXXXXXXXXXXXXXXXXXXXXXXXXXXXXXXXXXXXXXXXXXXXXXXXXXXXX
XXXXXXXXXXXXXXXXXXXXXXXXXXXXXXXXXXXXXXXXXXXXXXXXXXXXXXXXXXXXXXXXXXXXXXXXXXXXXXXXXXXXXXXXXXXXXXXXXXXXXXXXXXXXXXXXXXXXXXXXXXXXXXXXXXXXXXXXXXXXXXXXXXXXXXXXXXXXXXXXXXXXXXXXXXXXXXXX
XXXXXXXXXXXXXXXXXXXXXXXXXXXXXXXXXXXXXXXXXXXXXXXXXXXXXXXXXXXXXXXXXXXXXXXXXXXXXXXXXXXXXXXXX
000000000000000000000000000000000000000000000000000000000000000000000000000000000000000000000000000000000000000000000000XXXXXXXXXXXXXXXXXXXXXXXXXXXXXXXXXXXXXXXXXXXXXXXXXXXXXXXXXXXXXXXX
XXXXXXXXXXXXXXXXXXXXXXXXXXXXXXXXXXXXXXXXXXXXXXXXXXXXXXXXXXXXXXXXXXXXXXXXXXXXXXXXXXXXXXXXXXXXXXXXXXXXXXXXXXXXXXXXXXXXXXXXXXXXXXXXXXXXXXXXXXXXXXXXXXXXXXXXXXXXXXXXXXXXXXXXXXXXXXXXX
XXXXXXXXXXXXXXXXXXXXXXXXXXXXXXXXXXXXXXXXXXXXXXXXXXXXXXXXXXXXXXXXXXXXXXXXXXXXXXXXXXXXXXXXX
000000000000000000000000000000000000000000000000000000000000000000000000000000000000000000000000000000000000000000000000XXXXXXXXXXXXXXXXXXXXXXXXXXXXXXXXXXXXXXXXXXXXXXXXXXXXXXXXXXXXXXXX
XXXXXXXXXXXXXXXXXXXXXXXXXXXXXXXXXXXXXXXXXXXXXXXXXXXXXXXXXXXXXXXXXXXXXXXXXXXXXXXXXXXXXXXXXXXXXXXXXXXXXXXXXXXXXXXXXXXXXXXXXXXXXXXXXXXXXXXXXXXXXXXXXXXXXXXXXXXXXXXXXXXXXXXXXXXXXXXX
XXXXXXXXXXXXXXXXXXXXXXXXXXXXXXXXXXXXXXXXXXXXXXXXXXXXXXXXXXXXXXXXXXXXXXXXXXXXXXXXXXXXXXXXX
>B7T1D9
MNGIRWIASYPKAGNTWLRCMLAAYITGKAPQTWKDMETVSLELEGMLHLGDMPPTEPTKPVLVKTHLKADVPVLGLYSEATAKVLYLVRNPRDILLSAMRMTAISRDDMESSRTFARDFIANEGLRMRGRGGGAGLGSWPENVRIWTESSRDRFPNADVLT
MRYEDLKGDPVARFSEIVEFLDLGDPVDIEDIRRAVAACTLERMRELEKRSQQQGQWASMTGGRGGEKHPFVGEGRYDQSLSFLGEDIESAYQELLHGDSEFAHYAKQYGYAG
00000000000000000000000000000000000000000000000000000000000000000000000000000000000000000000000000000000000000000000000000000000000000000000000000000000000000000000000000000000000000000
00000000000000000000000000000000000011111111111111111111111111000000000000000000000000000000000000000000
00000000000000000000000000000000000000000000000000000000000000000000000000000000000000000000000000000000000000000000000000000000000000000000000000000000000000000000000000000000000000000
00000000000000000000000000000000000000000000000000000000000000000000000000000000000000000000000000000000
00000000000000000000000000000000000000000000000000000000000000000000000000000000000000000000000000000000000000000000000000000000000000000000000000000000000000000000000000000000000000000
00000000000000000000000000000000000000000000000000000000000000000000000000000000000000000000000000000000
>P46014
MAMIGMNIIGLFMVLMLLLISLIILFACKPWRYFSRFRSSSRFSSTFKVGDLQRPLISDDGNLIQGQTSEVTREYDLEGACYQNDGLLHSSLTEGRFYKQRLPSSSPHFSQGESFVLEVISEPSDNALVGQTLKLPAEKGSLAEVQTYDWQNNRNENLQYNLEKDRLINL
SPRLVEDQRSWLFLEVIAGPAIGLQHAVNSTSSSKLPVKLGRVSPSDLALKDSEVSGKHAQITWNSTKFKWELVDMGSLNGTLVNSHSISHPDLGSRKWGNPVELASDDIITLGTTTKVYVRISSQNEFQIPFKIGVASDPMAMRRGGRKLPMEDVCHYKWPLPGA
NKFGLFCVCDGHGGSGAAQSAIKIIPEVLANILSDSLRKEKVLSKRDASDVLRDMFAKTEARLEEHQYEGCTATVLLVWKDNEENFFAQCANLGDSACVIHLGGRYIQMTEDHRVVSLSERKRFQEAGLALRDGETRLFGINLARMLGDKFPKQQDSRFSAEPYISEP
LRIDQSSKDVFAVLASDGLWDVVSPKKAVQLVLQMRDKERGRESSAEKIANGLLNEARAMRTKDNTSIIYLDFDTSL
00000000000000000000000000000000000000000000000000000000000000000000000000000000000000000000000000000000000000000000000000000000000000000000000000000000000000000000000000000000000000000
0000000000000000000000000000000000000000000000000000000000000000000000000000000000000000000000000000000000000000000111111111111111000000000000000000000000000000000000000000000000000000000
00000000000000000000000000000000000000000000000000000000000000000000000000000000000000000000000000000000000000000000000000000000000000000000000000000000000000000000000000000000000000000
00000000000000000000000000000000000000000000000000000000
00000000000000000000000000000000000000000000000000000000000000000000000000000000000000000000000000000000000000000000000000000000000000000000000000000000000000000000000000000000000000000
00000000000000000000000000000000000000000000000000000000000000000000000000000000000000000000000000000000000000000000000000000000000000000000000000000000000000000000000000000000000000000
00000000000000000000000000000000000000000000000000000000000000000000000000000000000000000000000000000000000000000000000000000000000000000000000000000000000000000000000000000000000000000
00000000000000000000000000000000000000000000000000000000
00000000000000000000000000000000000000000000000000000000000000000000000000000000000000000000000000000000000000000000000000000000000000000000000000000000000000000000000000000000000000000
00000000000000000000000000000000000000000000000000000000000000000000000000000000000000000000000000000000000000000000000000000000000000000000000000000000000000000000000000000000000000000
00000000000000000000000000000000000000000000000000000000000000000000000000000000000000000000000000000000000000000000000000000000000000000000000000000000000000000000000000000000000000000
00000000000000000000000000000000000000000000000000000000
>P53330
MDPQTLITKANKVSYYGNPTSKESWRYDWYQPSKVSSNVQQPQQQLGDMENNLEKYPFRYKTWLRNQEDEKNLQRESCEDILDLKEFDRRILKKSLMTSHTKGDTSKATGAPSANQGDEALSVDDIRGAVGNSEAIPGLSAGVNNDNTKESKDVKMN
00000000000000000000000000000001111111111111111111111100000000000000000000000000000000000000000011111111111111111111111111111111111111111111111111111111111111111111111
000000000000000000000000000000000000000000000000000000000000000000000000000000000000000000000000000000000000000000000000000000000000000000000000000000000000000000000
000000000000000000000000000000000000000000000000000000000000000000000000000000000000000000000000000000000000000000000000000000000000000000000000000000000000000000000
>Q9Z0P7
MAELRPSVAPGPAAPPASGPSAPPAFASLFPPGLHAIYGECRRLYPDQPNPLQVTAIVKYWLGGPDPLDYVSMYRNMGSPSANIPEHWHYISFGLSDLYGDNRVHEFTGTDGPSGFGFELTFRLKRETGESAPPTWPAELMQGLARYVFQSENTFCSGDHVSWHS
PLDNSESRIQHMLLTEDPQMQPVRTPFGVVTFLQIVGVCTEELHSAQQWNGQGILELLRTVPIAGGPWLITDMRRGETIFEIDPHLQERVDKGIETDGSNLSGVSAKCAWDDLSRPPEDEEDSRSICLGTQPRRLSGKDTEQIRETLRRGLEINSKPVLPPINSQRQN
GLTHDRAPSRKDSLGSDSSTAIIPHELIRTRQLESVHLKFNQESGALIPLCLRGRLLHGRHFTYKSITGDMAITFVSTGVEGAFATEEHPYAAHGPWLQILLTEEFVEKMLEDLEDLTSPEEFKLPKEYSWPEKKLKVSILPDVVFDSPLH
00000000000000000000000000000000000000000000000000000000000000000000000000000000000000000000000000000000000000000000000000000000000000000000000000000000000000000000000000000000000000000
0000000000000000000000000000000000000000000000000000000000000000000000000000111111111111111111111111111111111111111111111111111111111111111111111111111111111111111111111111111111111
1111111000000000000000000000000000000000000000000000000000000000000000000000000000000000000000000000000000000000000000000000000000000
00000000000000000000000000000000000000000000000000000000000000000000000000000000000000000000000000000000000000000000000000000000000000000000000000000000000000000000000000000000000000000
00000000000000000000000000000000000000000000000000000000000000000000000000000000000000000000000000000000000000000000000000000000000000000000000000000000000000000000000000000000000000000
0000000000000000000000000000000000000000000000000000000000000000000000000000000000000000000000000000000000000000000000000000000000000000000

00000000000000000000000000000000000000000000000000000000000000000000000000000000000000000000000000000000000000000000000000000000000000000000000000000000000000000000000000000000
00000000000000000000000000000000000000000000000000000000000000000000000000000000000000000000000000000000000000000000000000000000000000000000000000000000000000000000000000000000
000000000000000000000000000000000000000000000000000000000000000000000000000000000000000000000000000000000000000000000000000000000000
>G0SCY6
MAAEDRQPADIVEGATAGDVEEEVAPAAKSAEDRKAAAALSKLDAHADEDMAPAREVDQEAVKNAMSALSGASTEKKEVKKVKVDPADVNLLVEELELSKAKATELLKAHDGDAIKAMKAYIQPAF
111111111111111111111111100000000000000000111111111111110000000000000000000000000000000000000000000000000000000000000000000000
111111111111111111111111100000000000000000000000000000000000000000000000000000000000000000000000000000000000000000000000000000
000000000000000000000000000000000000000000000000000000000000000000000000000000000000000000000000000000000000000000000000000000
>G0S4M4
MPQPLSTREANLFRTVIRHYEDKQYKRGLKAAEQILKKNPKHGDTMSMKALILNAQGKTEEAFALAKEALTIDMKSYICWHVYGILYRTNKNFDEAIKAYKFALKLEPESHQIQRDLAVLQIQMRDYAGYVQSRLNMLKARPQIRQNWTALAIAYHLEGNLEKAEHIL
TTYEKSLTTPPPKTDLEHSEALLYKNTIIAERGDIERALQHLETDCKHCLDRLAVMELRASYLSKLARKDEAAKAYRALLDRNPEHMDYYKGLISALDISADDEEAQKAVYDEYAAKYPRSDAAKRLPLNFLSGERFRTTAKAYLTLMFDKGVPSTFANLKHLYSDSFKKET
LASLAEEYLNEYVNARPSDNQADGDGSKGKGAALYYLAQHYNYYMSRDLTRALEYVEKAIELDPKNVDFHMTKARIFKHQGDLAKAAETMDYARSLDPKDRYINSKAAKYQLRNNENEKALATMGLFTRAETAGGPLADLTDMQCIWFLTEDGEAWQRRGNTA
LALKRYHTVFSIFDTWQEDQFDFHSFSLRKGQIRAYVDMVRWEDRLREHPFYFRAALDAVNLYLSMYDKPQSANGANGTEAANPNGEDAAEKKKAAKKARKEAQKAEREAAERAAKQDPNKPGAQKGKEEDIKKKDDDPNGEKLAATKDPLGDAMKFLNYILQ
FSPKNIDGQIAGFEVYIRKKKYLLALRCLKAASAIDKNHPNVLEQAAKLRKIVSSALGSMAPKLREVIQAELVGVPGA
00000000000000000000000000000000000000000000000000000000000000000000000000000000000000000000000000000000000000000000000000000000000000000000000000000000000000000000000000000000
00000000000000000000000000000000000000000000000000000000000000000000000000000000000000000000000000000000000000000000000000000000000000000000000000000000000000000000000000000000
00000000000000000000000000000000000000000000000000000000000000000000000000000000000000000000000000000000000000000000000000000000000000000000000000000000000000000000000000000000
00000000000000000000000000000000000000000000111111111111111111111111111111111111111111111111111111111111111111111111100000000000000000000000000000000000000000000000000000000000000
0000000000000000000000000000000000000000
00000000000000000000000000000000000000000000000000000000000000000000000000000000000000000000000000000000000000000000000000000000000000000000000000000000000000000000000000000000
00000000000000000000000000000000000000000000000000000000000000000000000000000000000000000000000000000000000000000000000000000000000000000000000000000000000000000000000000000000
00000000000000000000000000000000000000000000000000000000000000000000000000000000000000000000000000000000000000000000000000000000000000000000000000000000000000000000000000000000
00000000000000000000000000000000000000000000000000000000000000000000000000000000000000000000000000000000000000000000000000000000000000000000000000000000000000000000000000000000
0000000000000000000000000000000000000000
00000000000000000000000000000000000000000000000000000000000000000000000000000000000000000000000000000000000000000000000000000000000000000000000000000000000000000000000000000000
00000000000000000000000000000000000000000000000000000000000000000000000000000000000000000000000000000000000000000000000000000000000000000000000000000000000000000000000000000000
00000000000000000000000000000000000000000000000000000000000000000000000000000000000000000000000000000000000000000000000000000000000000000000000000000000000000000000000000000000
00000000000000000000000000000000000000000000000000000000000000000000000000000000000000000000000000000000000000000000000000000000000000000000000000000000000000000000000000000000
0000000000000000000000000000000000000000
>P47093
MSATLKDYLNKRVVIIKVDGECLIASLNGFDKNTNLFITNVFNRISKEFICKAQLLRGSEIALVGLIDAENDDSLAPIDEKKVPMLKDTKNKIENEHVIWEKVYESKTK
0000000000000000000000000000000000000000000000000000000000000000000000000000001111111111111111111111111111111
0000000000000000000000000000000000000000000000000000000000000000000000000000000000000000000000000000000000000
0000000000000000000000000000000000000000000000000000000000000000000000000000000000000000000000000000000000000
>Q06406
MSGKASTEGSVTTEFLSDIIGKTVNVKLASGLLYSGRLESIDGFMNVALSSATEHYESNNNKLLNKFNSDVFLRGTQVMYISEQKI
11111111110000000000000000000000000000000000000000000000000000000000000000000000000000
00000000000000000000000000000000000000000000000000000000000000000000000000000000000000
00000000000000000000000000000000000000000000000000000000000000000000000000000000000000
>Q5V913
MSNMDIDGINTGTIDKAPEEITSGTSGTTRPIIRPATLAPPSNKRTRNPSPERATTISEADVGRKTQKKQTPTEIKKSVYNMVVKLGEFYNQMMVKAGLNDDMERNLIQNAHAVERILLAATDDKKTEFQKKKNARDVKEGKEEIDHNKTGGTFYKMVRDDKTIYFS
PIRVTFLKEEVKTMYKTTMGSDGFSGLNHIMIGHSQMNDVCFQRSKALKRVGLDPSLISTFAGSTLPRRSGATGVAIKGGGTLVAEAIRFIGRAMADRGLLRDIKAKTAYEKILLNLKNKCSAPQQKALVDQVIGSRNPGIADIEDLTLLARSMVVVRPSVASKVVLPIS
IYAKIPQLGFNVEEYSMVGYEAMALYNMATPVSILRVGDDAKDKSQLFFMSCFGAAYEDLRVLSALTGTEFKPRSALKCKGFHVPAKEQVEGMGAALMSIKLQFWAPMTRSGGNEVGGDGGSGQISCSPVFAVERPIALSKQAVRRMLSMNIEGRDADVKGNLL
KMMNDSMAKKTNGNAFIGKKMFQISDKNKTNPVEIPIKQTIPNFFFGRDTAEDYDDLDY
11111111111111111111111111111111111111111111111111111111111111111111111100000000000000000000000000000000000000000000000000000000000000000000000000000000000000000000000000000000
00000000000000000000000000000000000000000000000000000000000000000000000000000000000000000000000000000000000000000000000000000000000000000000000000000000000000000000000000000000
00000000000000000000000000000000000000000000000000000000000000000000000000000000000000000000000000000000000000000000000000000000000000000000000000000000000000000000000000000000
00000000000000000000000000000000
11111111111111111111111111111111111111111111111111111111111111111111111110000000000000000000000000000000000000000000000000000000000000000000000000000000000000000000000000000000
00000000000000000000000000000000000000000000000000000000000000000000000000000000000000000000000000000000000000000000000000000000000000000000000000000000000000000000000000000000
00000000000000000000000000000000000000000000000000000000000000000000000000000000000000000000000000000000000000000000000000000000000000000000000000000000000000000000000000000000
00000000000000000000000000000000
00000000000000000000000000000000000000000000000000000000000000000000000000000000000000000000000000000000000000000000000000000000000000000000000000000000000000000000000000000000
00000000000000000000000000000000000000000000000000000000000000000000000000000000000000000000000000000000000000000000000000000000000000000000000000000000000000000000000000000000
00000000000000000000000000000000000000000000000000000000000000000000000000000000000000000000000000000000000000000000000000000000000000000000000000000000000000000000000000000000
00000000000000000000000000000000
>C6ZFX3
MARSFTNIKAISALVAEEFSNSLARRGYAATAQSAGRVGASMSGKMGSTKSGEEKAAAREKVSWVPDPVTGYYKPENIKEIDVAELRSAVLGKN
1111111111111111111111111111111111111111111111111111111111111111111111111111111111111111111111
0000000000000000000000000000000000000000000000000000000000000000000000000000000000000000000000
0000000000000000000000000000000000000000000000000000000000000000000000000000000000000000000000
>Q2Q4X9
MSQEQPRRPQAGQDPIKYGDVLPVSGDLSQKPITPEDAAMMQSAESRVLGQTQPGGVASVMQSAATRNEQAGIVGHKDVTDVTGDRGVTVTETQVPGRRIITETVGGQVVGQFVEPTPVQVGLTGAVRESALTIGEALEATAHTVGDKPVEQSDASAIQAAE
VRATGSNVITPGGLASMAQSAAAFNAECQREEEKIKMGNVLTGATAKLPADKAATRQDAAGVASAEMRNNPDATATPGGVAASVAAAARLNENVGNVM
11111111111111111111111111111111111111111111111111111111111111111111111111111111111111111111111111111111111111111111111111111111111111111111111111111111111111111111111111111111
111111111111111111111111111111111111111111111111111111111111111111111111111111111111
00000000000000000000000000000000000000000000000000000000000000000000000000000000000000000000000000000000000000000000000000000000000000000000000000000000000000000000000000000000
000000000000000000000000000000000000000000000000000000000000000000000000000000000000
00000000000000000000000000000000000000000000000000000000000000000000000000000000000000000000000000000000000000000000000000000000000000000000000000000000000000000000000000000000
000000000000000000000000000000000000000000000000000000000000000000000000000000000000
>Q9UJX4
MASVHESLYFNPMMTNGVVHANVFGIKDWVTPYKIAVLVLLNEMSRTGEGAVSLMERRRLNQLLLPLLQGPDITLSKLYKLIEESCPQLANSVQIRIKLMAEGELKDMEQFFDDLSDSFSGTEPEVHKTSVVGLFLRHMILAYSKLSFSQVFKLYTALQQYFQNGEKK
TVEDADMELTSRDEGERKMEKEELDVSVREEEVSCSGPLSQKQAEFFLSQQASLLKNDETKALTPASLQKELNNLLKFNPDFAEAHYLSYLNNLRVQDVFSSTHSLLHYFDRLILTGAESKSNGEEGYGRSLRYAALNLAALHCRFGHYQQAELALQEAIRIAQESNDH
VCLQHCLSWLYVLGQKRSDSYVLLEHSVKKAVHFGLPYLASLGIQSLVQQRAFAGKTANKLMDALKDSDLLHWKHSLSELIDISIAQKTAIWRLYGRSTMALQQAQMLLSMNSLEAVNAGVQQNNTESFAVALCHLAELHAEQGCFAAASEVLKHLKERFPPNSQ
HAQLWMLCDQKIQFDRAMNDGKYHLADSLVTGITALNSIEGVYRKAVVLQAQNQMSEAHKLLQKLLVHCQKLKNTEMVISVLLSVAELYWRSSSPTIALPMLLQALALSKEYRLQLYASETVLNLAFAQLILGIPEQALSLLHMAIEPILADGAILDKGRAMFLVAKC
QVASAASYDQPKKAEALEAAIENLNEAKNYFAKVDCKERIRDVVYFQARLYHTLGKTQERNRCAMLFRQLHQELPSHGVPLINHL
11111111111111111111111111000000000000000000000000000000000000000000000000000000000000000000000000000000000000000000000000000000000000000000000000000000000000000000000111111111
111111111111111111111111111111111XXXXXXXXXXXXXXXXXXXXXXXXXXXXXXXXXXXXXXXXXXXXXXXXXXXXXXXXXXXXXXXXXXXXXXXXXXXXXXXXXXXXXXXXXXXXXXXXXXXXXXXXXXXXXXXXXXXXXXXXXXXXXXXXXXXX
XXX111111111111111111111111111111111111111111111111111111111111XXXXXXXXXXXXXXXXXXXXXXXXXXXXXXXXXXXXXXXXXXXXXXXXXXXXXXXXXXXXXXXXXXXXXXXXXXXXXXXXXXXXXXXXXXXXXXXXXXXXXXXXXXXXX
XXXXXXXXXXXXXXXXXXXXXXXXXXXXXXXXXXXXXXXXXXXXXXXXXXXXXXXXXXXXXXXXXXXXXXXXXXXXXXXXXXXXXXXXXXXXXXXXXXXXXXXXXXXXXXXXXXXXXXXXXXXXXXXXXXXXXXXXXXXXXXXXXXXXXXXXXXXXXXXXXXXXXXXX
XXXXXXXXXXXXXXXXXXXXXXXXXXXXXXXXXXXXXXXXXXXXXXXXXXXXXXXXXXXXX
00000000000000000000000000000000000000000000000000000000000000000000000000000000000000000000000000000000000000000000000000000000000000000000000000000000000000000000000000000000
0000000000000000000000000000XXXXXXXXXXXXXXXXXXXXXXXXXXXXXXXXXXXXXXXXXXXXXXXXXXXXXXXXXXXXXXXXXXXXXXXXXXXXXXXXXXXXXXXXXXXXXXXXXXXXXXXXXXXXXXXXXXXXXXXXXXXXXXXXXXXXXXXXXXXXX
XXX000000000000000000000000000000000000000000000000000000000000XXXXXXXXXXXXXXXXXXXXXXXXXXXXXXXXXXXXXXXXXXXXXXXXXXXXXXXXXXXXXXXXXXXXXXXXXXXXXXXXXXXXXXXXXXXXXXXXXXXXXXXXXXXXX
XXXXXXXXXXXXXXXXXXXXXXXXXXXXXXXXXXXXXXXXXXXXXXXXXXXXXXXXXXXXXXXXXXXXXXXXXXXXXXXXXXXXXXXXXXXXXXXXXXXXXXXXXXXXXXXXXXXXXXXXXXXXXXXXXXXXXXXXXXXXXXXXXXXXXXXXXXXXXXXXXXXXXXXX
XXXXXXXXXXXXXXXXXXXXXXXXXXXXXXXXXXXXXXXXXXXXXXXXXXXXXXXXXXXXX
00000000000000000000000000000000000000000000000000000000000000000000000000000000000000000000000000000000000000000000000000000000000000000000000000000000000000000000000000000000
0000000000000000000000000000XXXXXXXXXXXXXXXXXXXXXXXXXXXXXXXXXXXXXXXXXXXXXXXXXXXXXXXXXXXXXXXXXXXXXXXXXXXXXXXXXXXXXXXXXXXXXXXXXXXXXXXXXXXXXXXXXXXXXXXXXXXXXXXXXXXXXXXXXXXXX
XXX000000000000000000000000000000000000000000000000000000000000XXXXXXXXXXXXXXXXXXXXXXXXXXXXXXXXXXXXXXXXXXXXXXXXXXXXXXXXXXXXXXXXXXXXXXXXXXXXXXXXXXXXXXXXXXXXXXXXXXXXXXXXXXXXX
XXXXXXXXXXXXXXXXXXXXXXXXXXXXXXXXXXXXXXXXXXXXXXXXXXXXXXXXXXXXXXXXXXXXXXXXXXXXXXXXXXXXXXXXXXXXXXXXXXXXXXXXXXXXXXXXXXXXXXXXXXXXXXXXXXXXXXXXXXXXXXXXXXXXXXXXXXXXXXXXXXXXXXXX
XXXXXXXXXXXXXXXXXXXXXXXXXXXXXXXXXXXXXXXXXXXXXXXXXXXXXXXXXXXXX
>P30260
MTVLQEPVQAAIWQALNHYAYRDAVFLAERLYAEVHSEEALFLLATCYYRSGKAYKAYRLLKGHSCTTPQCKYLLAKCCVDLSKLAEGEQILSGGVFNKQKSHDDIVTEFGDSACFTLSLLGHVYCKTDRLAKGSECYQKSLSLNPFLWSPFESLCEIGEKPDPDQTFKF
TSLQNFSNCLPNSCTTQVPNHSLSHRQPETVLTETPQDTIELNRLNLESSNSKYSLNTDSSVSYIDSAVISPDTVPLGTGTSILSKQVQNKPKTGRSLLGGPAALSPLTPSFGILPLETPSPGDGSYLQNYTNTPPVIDVPSTGAPSKKSVARIGQTGTKSVFSQSGNSREVT
PILAQTQSSGPQTSTTPQVLSPTITSPPNALPRRSSRLFTSDSSTTKENSKKLKMKFPPKIPNRKTKSKTNKGGITQPNINDSLEITKLDSSIISEGKISTITPQIQAFNLQKAAAEGLMSLLREMGKGYLALCSYNCKEAINILSHLPSHHYNTGWVLCQIGRAYFELSEYMQ
AERIFSEVRRIENYRVEGMEIYSTTLWHLQKDVALSVLSKDLTDMDKNSPEAWCAAGNCFSLQREHDIAIKFFQRAIQVDPNYAYAYTLLGHEFVLTEELDKALACFRNAIRVNPRHYNAWYGLGMIYYKQEKFSLAEMHFQKALDINPQSSVLLCHIGVVQHALKKS
EKALDTLNKAIVIDPKNPLCKFHRASVLFANEKYKSALQELEELKQIVPKESLVYFLIGKVYKKLGQTHLALMNFSWAMDLDPKGANNQIKEAIDKRYLPDDEEPITQEEQIMGTDESQESSMTDADDTQLHAAESDEF
00000000000000000000000000000000000000000000000000000000000000000000000000000000000000000000000000000000000000000000000000000000000000000000000000000000000000000000111111
11111111111111111111111111111111111111111111111111111111111111111111111111111111111111111111111111111111111111111111111111111111111111111111111111111111111111111111111111111111
11111111111111111111111111111111111111111111111111111111111111111111111111111111111111111111111111111111110000000000000000000000000000000000000000000000000000000000000000000000
00000000000000000000000000000000000000000000000000000000000000000000000000000000000000000000000000000000000000000000000000000000000000000000000000000000000000000000000000000000
000000000000000000000000000000000000000011111111111111111111111111111111111111111111111111111111111111111111111111111111
00000000000000000000000000000000000000000000000000000000000000000000000000000000000000000000000000000000000000000000000000000000000000000000000000000000000000000000000000000000
00000000000000000000000000000000000000000000000000000000000000000000000000000000000000000000000000000000000000000000000000000000000000000000000000000000000000000000000000000000
00000000000000000000000000000000000000000000000000000000000000000000000000000000000000000000000000000000000000000000000000000000000000000000000000000000000000000000000000000000
00000000000000000000000000000000000000000000000000000000000000000000000000000000000000000000000000000000000000000000000000000000000000000000000000000000000000000000000000000000
000000000000000000000000000000000000000000000000000000000000000000000000000000000000000000000000000000000000000000000000
00000000000000000000000000000000000000000000000000000000000000000000000000000000000000000000000000000000000000000000000000000000000000000000000000000000000000000000000000000000
00000000000000000000000000000000000000000000000000000000000000000000000000000000000000000000000000000000000000000000000000000000000000000000000000000000000000000000000000000000
00000000000000000000000000000000000000000000000000000000000000000000000000000000000000000000000000000000000000000000000000000000000000000000000000000000000000000000000000000000
00000000000000000000000000000000000000000000000000000000000000000000000000000000000000000000000000000000000000000000000000000000000000000000000000000000000000000000000000000000
000000000000000000000000000000000000000000000000000000000000000000000000000000000000000000000000000000000000000000000000
>P30281
MELLCCEGTRHAPRAGPDPRLLGDQRVLQSLLRLEERYVPRASYFQCVQREIKPHMRKMLAYWMLEVCEEQRCEEEVFPLAMNYLDRYLSCVPTRKAQLQLLGAVCMLLASKLRETTPLTIEKLCIYTDHAVSPRQLRDWEVLVLGKLKWDLAAVIAHDFLAFILHR
LSLPRDRQALVKKHAQTFLALCATDYTFAMYPPSMIATGSIGAAVQGLGACSMSGDELTELLAGITGTEVDCLRACQEQIEAALRESLREASQTSSSPAPKAPRGSSSQGPSQTSTPTDVTAIHL
00000000000000000000000000000000000000000000000000000000000000000000000000000000000000000000000000000000000000000000000000000000000000000000000000000000000000000000000000000000
00000000000000000000000000000000000000000000000000000000000000000000000000000011111111111111111111111111111111111111
00000000000000000000000000000000000000000000000000000000000000000000000000000000000000000000000000000000000000000000000000000000000000000000000000000000000000000000000000000000
00000000000000000000000000000000000000000000000000000000000000000000000000000000000000000000000000000000000000000000
00000000000000000000000000000000000000000000000000000000000000000000000000000000000000000000000000000000000000000000000000000000000000000000000000000000000000000000000000000000
00000000000000000000000000000000000000000000000000000000000000000000000000000000000000000000000000000000000000000000
>Q9UKT4
MSRRPCSCALRPPRCSCSASPSAVTAAGRPRPSDSCKEESSTLSVKMKCDFNCNHVHSGLKLVKPDDIGRLVSYTPAYLEGSCKDCIKDYERLSCIGSPIVSPRIVQLETESKRLHNKENQHVQQTLNSTNEIEALETSRLYEDSGYSSFSLQSGLSEHEEGSLLEENFGDSL
QSCLLQIQSPDQYPNKNLLPVLHFEKVVCSTLKKNAKRNPKVDREMLKEIIARGNFRLQNIIGRKMGLECVDILSELFRRGLRHVLATILAQLSDMDLINVSKVSTTWKKILEDDKGAFQLYSKAIQRVTENNNKFSPHASTREYVMFRTPLASVQKSAAQTSLKKDAQ
TKLSNQGDQKGSTYSRHNEFSEVAKTLKKNESLKACIRCNSPAKYDCYLQRATCKREGCGFDYCTKCLCNYHTTKDCSDGKLLKASCKIGPLPGTKKSKKNLRRL
11111111111111111111111111111111111111111111111111111111111111111111111111111111111111111111111111111111111111111111111111111111111111111111111111111111111111111111111111111111
11111111111111111111111111111111111111111111111111111111111111111111111111111111111111111111111111111111111111111111111111111XXXXXXXXXXXXX11111111111111111111111111111111111111
111XXXXXXXXXXXXXXXXXXXXXXXXXXXXXXXXXXXXXXXXXXXXXXXXXXXXXXXXXXXXXXXXXXXXXXXXXXXXXXXXXXX11111111111

00000000000000000000000000000000000000000000000000000000000000000000000000000000000000000000000000000000000000000000000000000000000000000000000000000000000000000000000000000000
000000000000000000000000000000000000000000000000000000000000000000000000000000000000000000000000000000000000000000000000000000000000000XXXXXXXXXXXXXXX00000000000000000000000000
000XXXXXXXXXXXXXXXXXXXXXXXXXXXXXXXXXXXXXXXXXXXXXXXXXXXXXXXXXXXXXXXXXXXXXXXXXXXXXXXXXXXXXXX00000000000
00000000000000000000000000000000000000000000000000000000000000000000000000000000000000000000000000000000000000000000000000000000000000000000000000000000000000000000000000000000
000000000000000000000000000000000000000000000000000000000000000000000000000000000000000000000000000000000000000000000000000000000000000XXXXXXXXXXXXXXX00000000000000000000000000
000XXXXXXXXXXXXXXXXXXXXXXXXXXXXXXXXXXXXXXXXXXXXXXXXXXXXXXXXXXXXXXXXXXXXXXXXXXXXXXXXXXXXXXX00000000000
>Q13042
MNLERLRKRVRQYLDQQQYQSALFWADKVASLSREEPQDIYWLAQCLYLTAQYHRAAHALRSRKLDKLYEACRYLAARCHYAAKEHQQALDVLDMEEPINKRLFEKYLKDESGFKDPSSDWEMSQSSIKSSICLLRGKIYDALDNRTLATYSYKEALKLDVYCFEAFD
LLTSHHMLTAQEEKELLESLPLSKLCNEEQELLRFLFENKLKKYNKPSETVIPESVDGLQENLDVVVSLAERHYYNCDFKMCYKLTSVVMEKDPFHASCLPVHIGTLVELNKANELFYLSHKLVDLYPSNPVSWFAVGCYYLMVGHKNEHARRYLSKATTLEKTYGPAWI
AYGHSFAVESEHDQAMAAYFTAAQLMKGCHLPMLYIGLEYGLTNNSKLAERFFSQALSIAPEDPFVMHEVGVVAFQNGEWKTAEKWFLDALEKIKAIGNEVTVDKWEPLLNNLGHVCRKLKKYAEALDYHRQALVLIPQNASTYSAIGYIHSLMGNFENAVDYFH
TALGLRRDDTFSVTMLGHCIEMYIGDSEAYIGADIKDKLKCYDFDVHTMKTLKNIISPPWDFREFEVEKQTAEETGLTPLETSRKTPDSRPSLEETFEIEMNESDMMLETSMSDHST
000000000000000000000000000000000000000000000000000000000000000000000000000000000000000000000111111111111111111111111111111110000000000000000000000000000000000000000000000000000
00000000000000000000000000000000000000000000000000000000000000000000000000000000000000000000000000000000000000000000000000000000000000000000000000000000000000000000000000000000
00000000000000000000000000000000000000000000000000000000000000000000000000000000000000000000000000000000000000000000000000000000000000000000000000000000000000000000000000000001
11111111111111111111111111111111111111111111111111111111111111111111111111111111111111111111
00000000000000000000000000000000000000000000000000000000000000000000000000000000000000000000000000000000000000000000000000000000000000000000000000000000000000000000000000000000
00000000000000000000000000000000000000000000000000000000000000000000000000000000000000000000000000000000000000000000000000000000000000000000000000000000000000000000000000000000
00000000000000000000000000000000000000000000000000000000000000000000000000000000000000000000000000000000000000000000000000000000000000000000000000000000000000000000000000000000
00000000000000000000000000000000000000000000000000000000000000000000000000000000000000000000
00000000000000000000000000000000000000000000000000000000000000000000000000000000000000000000000000000000000000000000000000000000000000000000000000000000000000000000000000000000
00000000000000000000000000000000000000000000000000000000000000000000000000000000000000000000000000000000000000000000000000000000000000000000000000000000000000000000000000000000
00000000000000000000000000000000000000000000000000000000000000000000000000000000000000000000000000000000000000000000000000000000000000000000000000000000000000000000000000000000
00000000000000000000000000000000000000000000000000000000000000000000000000000000000000000000
>P60006
MSTLFPSLFPRVTETLWFNLDRPCVEETELQQQEQQHQAWLQSIAEKDNNLVPIGKPASEHYDDEEEEDDEDDEDSEEDSEDDEDMQDMDEMNDYNESPDDGEVNEVDMEGNEQDQDQWMI
XXXXXXXXXXXXXXXXXXXXXXXXXXXXXXXXXXXXXXXXXXXXXXXXXXXXXXXXXXXXXXXXXXXXXXXXXXXXXXX111111111111111111111111111111111111111111
XXXXXXXXXXXXXXXXXXXXXXXXXXXXXXXXXXXXXXXXXXXXXXXXXXXXXXXXXXXXXXXXXXXXXXXXXXXXXXX000000000000000000000000000000000000000000
XXXXXXXXXXXXXXXXXXXXXXXXXXXXXXXXXXXXXXXXXXXXXXXXXXXXXXXXXXXXXXXXXXXXXXXXXXXXXXX000000000000000000000000000000000000000000
>Q92973
MVWDRQTKMEYEWKPDEQGLQQILQLLKESQSPDTTIQRTVQQKLEQLNQYPDFNNYLIFVLTKLKSEDEPTRSLSGLILKNNVKAHFQNFPNGVTDFIKSECLNNIGDSSPLIRATVGILITTIASKGELQNWPDLLPKLCSLLDSEDYNTCEGAFGALQKICEDSAEIL
DSDVLDRPLNIMIPKFLQFFKHSSPKIRSHAVACVNQFIISRTQALMLHIDSFIENLFALAGDEEPEVRKNVCRALVMLLEVRMDRLLPHMHNIVEYMLQRTQDQDENVALEACEFWLTLAEQPICKDVLVRHLPKLIPVLVNGMKYSDIDIILLKGDVEEDETIPDSEQ
DIRPRFHRSRTVAQQHDEDGIEEEDDDDDEIDDDDTISDWNLRKCSAAALDVLANVYRDELLPHILPLLKELLFHHEWVVKESGILVLGAIAEGCMQGMIPYLPELIPHLIQCLSDKKALVRSITCWTLSRYAHWVVSQPPDTYLKPLMTELLKRILDSNKRVQEAACS
AFATLEEEACTELVPYLAYILDTLVFAFSKYQHKNLLILYDAIGTLADSVGHHLNKPEYIQMLMPPLIQKWNMLKDEDKDLFPLLECLSSVATALQSGFLPYCEPVYQRCVNLVQKTLAQAMLNNAQPDQYEAPDKDFMIVALDLLSGLAEGLGGNIEQLVARSNILTL
MYQCMQDKMPEVRQSSFALLGDLTKACFQHVKPCIADFMPILGTNLNPEFISVCNNATWAIGEISIQMGIEMQPYIPMVLHQLVEIINRPNTPKTLLENTAITIGRLGYVCPQEVAPMLQQFIRPWCTSLRNIRDNEEKDSAFRGICTMISVNPSGVIQDFIFFCDAV
ASWINPKDDLRDMFCKILHGFKNQVGDENWRRFSDQFPLPLKERLAAFYGV
00000000000000000000000000000000000000000000000000000000000000000000000000000000000000000000000000000000000000000000000000000000000000000000000000000000000000000000000000000000
00000000000000000000000000000000000000000000000000000000000000000000000000000000000000000000000000000000000000000000000000000000000000001111111111111111111111111111111111111111
11111111111111111111110000000000000000000000000000000000000000000000000000000000000000000000000000000000000000000000000000000000000000000000000000000000000000000000000000000000
00000000000000000000000000000000000000000000000000000000000000000000000000000000000000000000000000000000000000000000000000000000000000000000000000000000000000000000000000000000
00000000000000000000000000000000000000000000000000000000000000000000000000000000000000000000000000000000000000000000000000000000000000000000000000000000000000000000000000000000
000000000000000000
00000000000000000000000000000000000000000000000000000000000000000000000000000000000000000000000000000000000000000000000000000000000000000000000000000000000000000000000000000000
00000000000000000000000000000000000000000000000000000000000000000000000000000000000000000000000000000000000000000000000000000000000000000000000000000000000000000000000000000000
00000000000000000000000000000000000000000000000000000000000000000000000000000000000000000000000000000000000000000000000000000000000000000000000000000000000000000000000000000000
00000000000000000000000000000000000000000000000000000000000000000000000000000000000000000000000000000000000000000000000000000000000000000000000000000000000000000000000000000000
00000000000000000000000000000000000000000000000000000000000000000000000000000000000000000000000000000000000000000000000000000000000000000000000000000000000000000000000000000000
000000000000000000
00000000000000000000000000000000000000000000000000000000000000000000000000000000000000000000000000000000000000000000000000000000000000000000000000000000000000000000000000000000
00000000000000000000000000000000000000000000000000000000000000000000000000000000000000000000000000000000000000000000000000000000000000000000000000000000000000000000000000000000
00000000000000000000000000000000000000000000000000000000000000000000000000000000000000000000000000000000000000000000000000000000000000000000000000000000000000000000000000000000
00000000000000000000000000000000000000000000000000000000000000000000000000000000000000000000000000000000000000000000000000000000000000000000000000000000000000000000000000000000
00000000000000000000000000000000000000000000000000000000000000000000000000000000000000000000000000000000000000000000000000000000000000000000000000000000000000000000000000000000
000000000000000000
>Q9Y3B4
MAMQAAKRANIRLPPEVNRILYIRNLPYKITAEEMYDIFGKYGPIRQIRVGNTPETRGTAYVVYEDIFDAKNACDHLSGFNVCNRYLVVLYYNANRAFQKMDTKKKEEQLKLLKEKYGINTDPPK
11111111111111111111000000000000000000000000000000000000000000000000000000000000000000000000000000001111111111111111111111111
00000000000000000000000000000000000000000000000000000000000000000000000000000000000000000000000000000000000000000000000000000
00000000000000000000000000000000000000000000000000000000000000000000000000000000000000000000000000000000000000000000000000000
>Q8IW19
MSGGFELQPRDGGPRVALAPGETVIGRGPLLGITDKRVSRRHAILEVAGGQLRIKPIHTNPCFYQSSEKSQLLPLKPNLWCYLNPGDSFSLLVDKYIFRILSIPSEVEMQCTLRNSQVLDEDNILNETPKSPVINLPHETTGASQLEGSTEIAKTQMTPTNSVSFLGENRD
CNKQQPILAERKRILPTWMLAEHLSDQNLSVPAISGGNVIQGSGKEEICKDKSQLNTTQQGRRQLISSGSSENTSAEQDTGEECKNTDQEESTISSKEMPQSFSAITLSNTEMNNIKTNAQRNKLPIEELGKVSKHKIATKRTPHKEDEAMSCSENCSSAQGDSLQDES
QGSHSESSSNPSNPETLHAKATDSVLQGSEGNKVKRTSCMYGANCYRKNPVHFQHFSHPGDSDYGGVQIVGQDETDDRPECPYGPSCYRKNPQHKIEYRHNTLPVRNVLDEDNDNVGQPNEYDLNDSFLDDEEEDYEPTDEDSDWEPGKEDEEKEDVEELLKE
AKRFMKRK
00000000000000000000000000000000000000000000000000000000000000000000000000000000000000000000000000000000000000000000000000000000000000000000000000000000000000000000000000000000
00000000000000000000000000000000000000000000000000000000000000000000000000000000000000000000000000000000000000000000000000000000000000000000000000000000000000000000000000000000
0000000000000000000000000000000000000000000000000111111111111111111111111XXXXXXXXXXXXXXXXXXXXXXXXXXXXXXXXXXX111111111111111111111111111111111111111111111111111
00000000000000000000000000000000000000000000000000000000000000000000000000000000000000000000000000000000000000000000000000000000000000000000000000000000000000000000000000000000
00000000000000000000000000000000000000000000000000000000000000000000000000000000000000000000000000000000000000000000000000000000000000000000000000000000000000000000000000000000
0000000000000000000000000000000000000000000000000000000000000000000000000XXXXXXXXXXXXXXXXXXXXXXXXXXXXXXXXXXX111111111111111111111111111111111111111111111111111
00000000000000000000000000000000000000000000000000000000000000000000000000000000000000000000000000000000000000000000000000000000000000000000000000000000000000000000000000000000
00000000000000000000000000000000000000000000000000000000000000000000000000000000000000000000000000000000000000000000000000000000000000000000000000000000000000000000000000000000
0000000000000000000000000000000000000000000000000000000000000000000000000XXXXXXXXXXXXXXXXXXXXXXXXXXXXXXXXXXX000000000000000000000000000000000000000000000000000
>P36118
MAIKKRNKIRLPSGSPEEVGIDGSAHKPMQQIKPLVSNDSEDDDNDICVLQPIKFKKVPKRDITFDGEQAIKEDNSHYEDLYHSKKNTNASTRNKDDLLILNMEDLMEGNHHLLSDSSEAGSSSEGEHISSIPTRGEIAKLKAQKSLSRRKISESDVTTERDYVKLLDSED
KREIMETIRLNGGLKRNNEKEITNFSDDEMQGFQDEMLALTDNQIAIQKDSKRKIIEKAINEVPYRTNEEWETQLLSKGNINKSNEKIITPLPVLFPDDDESGNSIERINEMVSKICLQRKKVEMRLQALEKTKIDLEKSKASLINKLIGN
11111111111111111111111111111111111111111111111111111111111111111111111111111111111111111111111111111111111111111111111111111111111111111111111111111111111111111111111111111111
11111111111111111111111111111111111111111111111111111111111111111111111111111111111111111111111111111111111111111111111111111111111111111111111111
11111111111111111111111111111111111111111111111111111111111111111111111111111111111111111111111111111111111111111111111111111111111111111111111111111111111111111111111111111111
11111111111111111111111111111111111111111111111111111111111111111111111111111111111111111111111111111111111111111111111111111111111111111111111111
00000000000000000000000000000000000000000000000000000000000000000000000000000000000000000000000000000000000000000000000000000000000000000000000000000000000000000000000000000000
00000000000000000000000000000000000000000000000000000000000000000000000000000000000000000000000000000000000000000000000000000000000000000000000000
>Q6GX35
MTNSISGDQPTVTTFTSSTTSASGASGSLGASSVSTTANATVTQTANATNSAATSSIQTTGETVVNYTNSASAPTVTVSTSSSSTQATATSNKTSQAVAGKITSPDTSESSETSSTSSSDHIPSDYEPISTTENIYENIYESIDDSSTSGPENTSGGAAALNSLRGSSYSNYD
DAAADYEPISTTENIYESIDDSSTSDPENTSGGAAALNSLRGSSYSNYDDAAADYEPISTTENIYENIYESIDDSSTSGPENTSGGAAALNSLRGSSYSNYDDAAADYEPISTTENIYESIDDSSTSDPENTSGGAAAALNSLRGSSYSNYDDAAADYEPISTTENIYESIDDSS
TSDPENTSGGAAALNSLRGSSYSNYDDAAADYEPISTTENIYENIYESIDGSSTSDPENTSGGAAAALNSLRGSSYTTGPRNEGVFGPGPEGLPDMSLPSYDPTNKTSLLTFLSNPHVKSKMLENSGHFVFIDTDRSSFILVPNGNWDQVCSIKVQNGKTKEDLDIKDLE
NMCAKFCTGFNKFSGDWDSRVEPMMSAKAGVASGGNLPNTVIINNKFKTCVAYGPWNSREASSGYTPSAWRRGHQVNFGEIFEKANDFNKINWGTQAGPSSEDDGISFSNETPGAGPAAAPSPTPSSIPVINVNVNVGGTNVNIRDTNVNTTNTTPTTQSTD
ASTDTSDIDNINTNNQTDDINTTDKDSDGAGGVNGDISETESSSGDDSGSVSSSESDKNASVGNDGPAMKDILSAVRKHLDVVYPGDNGGSTEGPLQANQTLGDIVQDMETTGTSQETVVSPWKGSTSSTGSAGGSGSVQTLLPSPPPTPSTTTLRTGTGATTTSL
MMGGPIKADIITTGGGGRIPGGGTLEKLLPRIRAHLDISFDGQGDLVSTEEPQLGSIVNKFRKETGSGGIVASVESAPGKPGSAQVLTGTGGDKGNLFQAAAAVTQALGNVAGKVNLAIQGQKLSSLVNDDGKGSVGRDLFQAATQTTQALSSLIDTVG
XXXXXXXXXXXXXXXXXXXXXXXXXXXXXXXXXXXXXXXXXXXXXXXXXXXXXXXXXXXXXXXXXXXXXXXXXXXXXXXXXXXXXXXXXXXXXXXXXXXXXXXXXXXXXXXXXXXXXXXXXXXXXXXXXXXXXXXXXXXXXXXXXXXXXXXXXXXXXXXXXXXXXXXX
XXXXXXXXXXXXXXXXXXXXXXXXXXXXXXXXXXXXXXXXXXXXXXXXXXXXXXXXXXXXXXXXXXXXXXXXXXXXXXXXXXXXXXXXXXXXXXXXXXXXXXXXXXXXXXXXXXXXXXXXXXXXXXXXXXXXXXXXXXXXXXXXXXXXXXXXXXXXXXXXXXXXXXXXX
XXXXXXXXXXXXXXXXXXXXXXXXXXXXXXXXXXXXXXXXXXXXXXXXXXXXXXXXXXXXXXXXXXXXXXXXXXXXXXXXXXXXXXXXXXXXXXXXXXXXXXXXXXXXXXXXXXXXXXXXXXXXXXXXXXXXXXXXXXXXXXXXXXXXXXXXXXXXXXXXXXXXXXXXX
XXXXXXXXXXXXXXXXXXXXXXXXXXXXXXXXXXXXXXXXXXXXXXXXXXXXXXXXXXXXXXXXXXXXXXXXXXXXXXXXXXXXXXXXXXXXXXXXXXXXXXXXXXXXXXXXXXXXXXXXXXXXXXXXXXXXXXXXXXXXXXXXXXXXXXXXXXXXXXXXXXXXXXXXX
XXXXXXXXXXXXXXXXXXXXXXXXXXXXXXXXXXXXXXXXXXXXXXXXXXXXXXXXXXXXXXXXXXXXXXXXXXXXXXXXXXXXXXXXXXXXXXXXXXXXXXXXXXXXXXXXXXXXXXXXXXXXXXXXXXXXXXXXXXXXXXXXXXXXXXXXXXXXXXXXXXXXXXXXX
XXXXXXXXXXXXXXXXXXXXXXXXXXXXXXXXXXXXXXXXXXXXXXXXXXXXXXXXXXXXXXXXXXXXXXXXXXXXX1111111111111111111111111111111111111111111111111111111111111111111XXXXXXXXXXXXXXXXXXXXXXXXXXXXXXXX
XXXXXXXXXXXXXXXXXXXXXXXXXXXXXXXXXXXXXXXXXXXXXXXXXXXXXXXXXXXXXXXXXXXXXXXXXXXXXXXXXXXXXXXXXXXXXXXXXXXXXXXXXXXXXXXXXXXXXXXXXXXXXXXXXXXXXXXXXXXXXX
XXXXXXXXXXXXXXXXXXXXXXXXXXXXXXXXXXXXXXXXXXXXXXXXXXXXXXXXXXXXXXXXXXXXXXXXXXXXXXXXXXXXXXXXXXXXXXXXXXXXXXXXXXXXXXXXXXXXXXXXXXXXXXXXXXXXXXXXXXXXXXXXXXXXXXXXXXXXXXXXXXXXXXXXXX
XXXXXXXXXXXXXXXXXXXXXXXXXXXXXXXXXXXXXXXXXXXXXXXXXXXXXXXXXXXXXXXXXXXXXXXXXXXXXXXXXXXXXXXXXXXXXXXXXXXXXXXXXXXXXXXXXXXXXXXXXXXXXXXXXXXXXXXXXXXXXXXXXXXXXXXXXXXXXXXXXXXXXXXXXX
XXXXXXXXXXXXXXXXXXXXXXXXXXXXXXXXXXXXXXXXXXXXXXXXXXXXXXXXXXXXXXXXXXXXXXXXXXXXXXXXXXXXXXXXXXXXXXXXXXXXXXXXXXXXXXXXXXXXXXXXXXXXXXXXXXXXXXXXXXXXXXXXXXXXXXXXXXXXXXXXXXXXXXXXX
XXXXXXXXXXXXXXXXXXXXXXXXXXXXXXXXXXXXXXXXXXXXXXXXXXXXXXXXXXXXXXXXXXXXXXXXXXXXXXXXXXXXXXXXXXXXXXXXXXXXXXXXXXXXXXXXXXXXXXXXXXXXXXXXXXXXXXXXXXXXXXXXXXXXXXXXXXXXXXXXXXXXXXXXX
XXXXXXXXXXXXXXXXXXXXXXXXXXXXXXXXXXXXXXXXXXXXXXXXXXXXXXXXXXXXXXXXXXXXXXXXXXXXXXXXXXXXXXXXXXXXXXXXXXXXXXXXXXXXXXXXXXXXXXXXXXXXXXXXXXXXXXXXXXXXXXXXXXXXXXXXXXXXXXXXXXXXXXXXX
XXXXXXXXXXXXXXXXXXXXXXXXXXXXXXXXXXXXXXXXXXXXXXXXXXXXXXXXXXXXXXXXXXXXXXXXXXXXX1111111111111111111111111111111111111111111111111111111111111111111XXXXXXXXXXXXXXXXXXXXXXXXXXXXXXXX
XXXXXXXXXXXXXXXXXXXXXXXXXXXXXXXXXXXXXXXXXXXXXXXXXXXXXXXXXXXXXXXXXXXXXXXXXXXXXXXXXXXXXXXXXXXXXXXXXXXXXXXXXXXXXXXXXXXXXXXXXXXXXXXXXXXXXXXXXXXXXX
XXXXXXXXXXXXXXXXXXXXXXXXXXXXXXXXXXXXXXXXXXXXXXXXXXXXXXXXXXXXXXXXXXXXXXXXXXXXXXXXXXXXXXXXXXXXXXXXXXXXXXXXXXXXXXXXXXXXXXXXXXXXXXXXXXXXXXXXXXXXXXXXXXXXXXXXXXXXXXXXXXXXXXXXX
XXXXXXXXXXXXXXXXXXXXXXXXXXXXXXXXXXXXXXXXXXXXXXXXXXXXXXXXXXXXXXXXXXXXXXXXXXXXXXXXXXXXXXXXXXXXXXXXXXXXXXXXXXXXXXXXXXXXXXXXXXXXXXXXXXXXXXXXXXXXXXXXXXXXXXXXXXXXXXXXXXXXXXXXX
XXXXXXXXXXXXXXXXXXXXXXXXXXXXXXXXXXXXXXXXXXXXXXXXXXXXXXXXXXXXXXXXXXXXXXXXXXXXXXXXXXXXXXXXXXXXXXXXXXXXXXXXXXXXXXXXXXXXXXXXXXXXXXXXXXXXXXXXXXXXXXXXXXXXXXXXXXXXXXXXXXXXXXXX
XXXXXXXXXXXXXXXXXXXXXXXXXXXXXXXXXXXXXXXXXXXXXXXXXXXXXXXXXXXXXXXXXXXXXXXXXXXXXXXXXXXXXXXXXXXXXXXXXXXXXXXXXXXXXXXXXXXXXXXXXXXXXXXXXXXXXXXXXXXXXXXXXXXXXXXXXXXXXXXXXXXXXXXX
XXXXXXXXXXXXXXXXXXXXXXXXXXXXXXXXXXXXXXXXXXXXXXXXXXXXXXXXXXXXXXXXXXXXXXXXXXXXXXXXXXXXXXXXXXXXXXXXXXXXXXXXXXXXXXXXXXXXXXXXXXXXXXXXXXXXXXXXXXXXXXXXXXXXXXXXXXXXXXXXXXXXXXXX
XXXXXXXXXXXXXXXXXXXXXXXXXXXXXXXXXXXXXXXXXXXXXXXXXXXXXXXXXXXXXXXXXXXXXXXXXXXXX0000000000000000000000000000000000000000000000000000000000000000000XXXXXXXXXXXXXXXXXXXXXXXXXXXXXXXX
XXXXXXXXXXXXXXXXXXXXXXXXXXXXXXXXXXXXXXXXXXXXXXXXXXXXXXXXXXXXXXXXXXXXXXXXXXXXXXXXXXXXXXXXXXXXXXXXXXXXXXXXXXXXXXXXXXXXXXXXXXXXXXXXXXXXXXXXXXXXXX
>P55980
MTNETIDQTRTPDQTQSQTAFDPQQFINNLQVAFIKVDNVVASFDPDQKPIVDKNDRDNRQAFDGISQLREEYSNKAIKNPTKKNQYFSDFIDKSNDLINKDNLIDVESSTKSFQKFGDQRYQIFTSWVSHQKDPSKINTRSIRNFMENIIQPPIPDDKEKAEFLKSAK
QSFAGIIIGNQIRTDQKFMGVFDESLKERQEAEKNGGPTGGDWLDIFLSFIFNKKQSSDVKEAINQEPVPHVQPDIATTTTDIQGLPPEARDLLDERGNFSKFTLGDMEMLDVEGVADIDPNYKFNQLLIHNNALSSVLMGSHNGIEPEKVSLLYAGNGGFGDKHD
WNATVGYKDQQGNNVATLINVHMKNGSGLVIAGGEKGINNPSFYLYKEDQLTGSQRALSQEEIRNKVDFMEFLAQNNTKLDNLSEKEKEKFQNEIEDFQKDSKAYLDALGNDRIAFVSKKDTKHSALITEFNNGDLSYTLKDYGKKADKALDREKNVTLQGSLKHD
GVMFVDYSNFKYTNASKNPNKGVGATNGVSHLEAGFNKVAVFNLPDLNNLAITSFVRRNLENKLTAKGLSLQEANKLIKDFLSSNKELAGKALNFNKAVAEAKSTGNYDEVKKAQKDLEKSLRKREHLEKEVEKKLESKSGNKNKMEAKAQANSQKDEIFALINKEA
NRDARAIAYTQNLKGIKRELSDKLEKISKDLKDFSKSFDEFKNGKNKDFSKAEETLKALKGSVKDLGINPEWISKVENLNAALNEFKNGKNKDFSKVTQAKSDLENSVKDVIINQKVTDKVDNLNQAVSVAKAMGDFSRVEQVLADLKNFSKEQLAQQAQKNEDFNT
GKNSELYQSVKNSVNKTLVGNGLSGIEATALAKNFSDIKKELNEKFKNFNNNNNGLKNSTEPIYAKVNKKKTGQVASPEEPIYTQVAKKVNAKIDRLNQIASGLGGVGQAAGFPLKRHDKVDDLSKVGLSASPEPIYATIDDLGGPFPLKRHDKVDDLSKVGRSRNQE
LAQKIDNLNQAVSEAKAGFFGNLEQTIDKLKDSTKKNVMNLYVESAKKVPASLSAKLDNYAINSHTRINSNIQNGAINEKATGMLTQKNPEWLKLVNDKIVAHNVGSVSLSEYDKIGFNQKNMKDYSDSFKFSTKLNNAVKDIKSGFTHFLANAFSTGYYCLARENA
EHGIKNVNTKGGFQKS
00000000000000000000000000000000000000000000000000000000000000000000000000000000000000000000000000000000000000000000000000000000000000000000000000000000000000000000000000000000
00000000000000000000000000000000000000000000000000000000000000000000000000000000000000000000000000000000000000000000000000000000000000000000000000000000000000000000000000000000
00000000000000000000000000000000000000000000000000000000000000000000000000000000000000000000000000000000000000000000000000000000000000000000000000000000000000000000000000000000
00000000000000000000000000000000000000000000000000000000000000000000000000000000000000000000000000000000000000000000000000000000000000000000000000000000000000000000000000000000
00000000000000000000000000000000000000000000000000000000000000000000000000000000000000000000000000000000000000000000000000000000000000000000000000000000000000000000000000001111
11111111111111111111111111111111111111111111111111111111111111111111111111111111111111111111111111111111111111111111111111111111111111111111111111111111111111111111111111111111
1111111111111111111111111111111111111111111111111111111111111111111111111111111111111111111111111111111111111111111111111111111111
00000000000000000000000000000000000000000000000000000000000000000000000000000000000000000000000000000000000000000000000000000000000000000000000000000000000000000000000000000000
00000000000000000000000000000000000000000000000000000000000000000000000000000000000000000000000000000000000000000000000000000000000000000000000000000000000000000000000000000000
00000000000000000000000000000000000000000000000000000000000000000000000000000000000000000000000000000000000000000000000000000000000000000000000000000000000000000000000000000000
00000000000000000000000000000000000000000000000000000000000000000000000000000000000000000000000000000000000000000000000000000000000000000000000000000000000000000000000000000000
00000000000000000000000000000000000000000000000000000000000000000000000000000000000000000000000000000000000000000000000000000000000000000000000000000000000000000000000000001111

111111111111111111111111111111111111111111111111111111111111111111111111111111111111111111111111111111111111111111111111111111111111111111111111111111111111111111111
11111111111111111111111111111111111111111111111111111111111111111111111111111111111111111111111111111111111111111111111111
000000000000000000000000000000000000000000000000000000000000000000000000000000000000000000000000000000000000000000000000000000000000000000000000000000000000000000000
000000000000000000000000000000000000000000000000000000000000000000000000000000000000000000000000000000000000000000000000000000000000000000000000000000000000000000000
000000000000000000000000000000000000000000000000000000000000000000000000000000000000000000000000000000000000000000000000000000000000000000000000000000000000000000000
000000000000000000000000000000000000000000000000000000000000000000000000000000000000000000000000000000000000000000000000000000000000000000000000000000000000000000000
000000000000000000000000000000000000000000000000000000000000000000000000000000000000000000000000000000000000000000000000000000000000000000000000000000000000000000000
000000000000000000000000000000000000000000000000000000000000000000000000000000000000000000000000000000000000000000000000000000000000000000000000000000000000000000000
00000000000000000000000000000000000000000000000000000000000000000000000000000000000000000000000000000000000000000000000000
>Q9UJX5
MLRFPTCFPSFRVVGEKQLPQEIIFLVWSPKRDLIALANTAGEVLLHRLASFHRVWSFPPNENTGKEVTCLAWRPDGKLLAFALADTKKIVLCDVEKPESLHSFSVEAPVSCMHWMEVTVESSVLTSFYNAEDESNLLLPKLPTLPKNYSNTSKIFSEENSDEIIKLLGDV
RLNILVLGGSSGFIELYAYGMFKIARVTGIAGTCLALCLSSDLKSLSVVTEVSTNGASEVSYFQLETNLLYSFLPEVTRMARKFTHISALLQYINLSLTCMCEAWEEILMQMDSRLTKFVQEKNTTTSVQDEFMHLLLWGKASAELQTLLMNQLTVKGLKKLGQSIESSYS
SIQKLVISHLQSGSESLLYHLSELKGMASWKQKYEPLGLDAAGIEEAITAVGSFILKANELLQVIDSSMKNFKAFFRWLYVAMLRMTEDHVLPELNKMTQKDITFVAEFLTEHFNEAPDLYNRKGKYFNVERVGQYLKDEDDDLVSPPNTEGNQWYDFLQNSSHLKE
SPLLFPYYPRKSLHFVKRRMENIIDQCLQKPADVIGKSMNQAICIPLYRDTRSEDSTRRLFKFPFLWNNKTSNLHYLLFTILEDSLYKMCILRRHTDISQSVSNGLIAIKFGSFTYATTEKVRRSIYSCLDAQFYDDETVTVVLKDTVGREGRDRLLVQLPLSLVYNSEDSAEY
QFTGTYSTRLDEQCSAIPTRTMHFEKHWRLLESMKAQYVAGNGFRKVSCVLSSNLRHVRVFEMDIDDEWELDESSDEEEEASNKPVKIKEEVLSESEAENQQAGAAALAPEIVIKVEKLDPELDS
000000000000000000000000000000000000000000000000000000000000000000000000000000000000000000000000000000000000000000000000000000000000000000000000000000000000000000000
000000000000000000000000000000000000000000000000000000000000000000000000000000000000000000000000000000001111111111111000000000000000000000000000000000000000000000000
00000000000000000000000000000000000000000000000000000000000000000000111111111111110000000000001111111111111XXXXXXX111111111111111111111111111111111111111111110000000
000000000000000000000000000000000000000000000000000000000000000000000000000000000000000000000000000000000000000000000000000000000000000000000000000000000000000000000
0000000000000000000000000000000000000000000000000011111111111111111111111111111111111111111111111
000000000000000000000000000000000000000000000000000000000000000000000000000000000000000000000000000000000000000000000000000000000000000000000000000000000000000000000
000000000000000000000000000000000000000000000000000000000000000000000000000000000000000000000000000000000000000000000000000000000000000000000000000000000000000000000
00000000000000000000000000000000000000000000000000000000000000000000000000000000000000000000000XXXXXX0000000000000000000000000000000000000000000000000000000000000000
000000000000000000000000000000000000000000000000000000000000000000000000000000000000000000000000000000000000000000000000000000000000000000000000000000000000000000000
0000000000000000000000000000000000000000000000000000000000000000000000000000000000000000000000000
000000000000000000000000000000000000000000000000000000000000000000000000000000000000000000000000000000000000000000000000000000000000000000000000000000000000000000000
000000000000000000000000000000000000000000000000000000000000000000000000000000000000000000000000000000000000000000000000000000000000000000000000000000000000000000000
00000000000000000000000000000000000000000000000000000000000000000000000000000000000000000000000XXXXXX0000000000000000000000000000000000000000000000000000000000000000
000000000000000000000000000000000000000000000000000000000000000000000000000000000000000000000000000000000000000000000000000000000000000000000000000000000000000000000
0000000000000000000000000000000000000000000000000000000000000000000000000000000000000000000000000
>Q9UJX3
MDPGDAAILESSLRILYRLFESVLPPLPAALQSRMNVIDHVRDMAAAGLHSNVRLLSSLLLTMSNNNPELFSPPQKYQLLVYHADSLFHDKEYRNAVSKYTMALQQKKALSKTSKVRPSTGNSASTPQSQCLPSEIEVKYKMAECYTMLKQDKDAIAILDGIPSRQRTP
KINMMLANLYKKAGQERPSVTSYKEVLRQCPLALDAILGLLSLSVKGAEVASMTMNVIQTVPNLDWLSVWIKAYAFVHTGDNSRAISTICSLEKKSLLRDNVDLLGSLADLYFRAGDNKNSVLKFEQAQMLDPYLIKGMDVYGYLLAREGRLEDVENLGCRLFNISD
QHAEPWVVSGCHSFYSKRYSRALYLGAKAIQLNSNSVQALLLKGAALRNMGRVQEAIIHFREAIRLAPCRLDCYEGLIECYLASNSIREAMVMANNVYKTLGANAQTLTLLATVCLEDPVTQEKAKTLLDKALTQRPDYIKAVVKKAELLSREQKYEDGIALLRNALAN
QSDCVLHRILGDFLVAVNEYQEAMDQYSIALSLDPNDQKSLEGMQKMEKEESPTDATQEEDVDDMEGSGEEGDLEGSDSEAAQWADQEQWFGMQ
111111111111111111111111111111111110000000000000000000000000000000000000000000000000000000000001111111111111111111100000000000000000000000000000000000000000000000000
000000000000000000000000000000000000000000000000000000000000000000000000000000000000000000000000000000000000000000000000000000000000000000000000000000000000000000000
000000000000000000000000000000000000000000000000000000000000000000000000000000000000000000000000000000000000000000000000000000000000000000000000000000000000000000000
0000000000001111111111111111111111111111111111111111111111111111111
000000000000000000000000000000000000000000000000000000000000000000000000000000000000000000000000000000000000000000000000000000000000000000000000000000000000000000000
000000000000000000000000000000000000000000000000000000000000000000000000000000000000000000000000000000000000000000000000000000000000000000000000000000000000000000000
000000000000000000000000000000000000000000000000000000000000000000000000000000000000000000000000000000000000000000000000000000000000000000000000000000000000000000000
0000000000000000000000000000000000000000000000000000000000000000000
000000000000000000000000000000000000000000000000000000000000000000000000000000000000000000000000000000000000000000000000000000000000000000000000000000000000000000000
000000000000000000000000000000000000000000000000000000000000000000000000000000000000000000000000000000000000000000000000000000000000000000000000000000000000000000000
000000000000000000000000000000000000000000000000000000000000000000000000000000000000000000000000000000000000000000000000000000000000000000000000000000000000000000000
0000000000000000000000000000000000000000000000000000000000000000000
>Q96FI4
MPEGPELHLASQFVNEACRALVFGGCVEKSSVSRNPEVPFESSAYRISASARGKELRLILSPLPGAQPQQEPLALVFRFGMSGSFQLVPREELPRHAHLRFYTAPPGPRLALCFVDIRRFGRWDLGGKWQPGRGPCVLQEYQQFRENVLRNLADKAFDRPICEALLD
QRFFNGIGNYLRAEILYRLKIPPFEKARSVLEALQQHRPSPELTLSQKIRTKLQNPDLLELCHSVPKEVVQLGGKGYGSESGEEDFAAFRAWLRCYGMPGMSSLQDRHGRTIWFQGDPGPLAPKGRKSRKKKSKATQLSPEDRVEDALPPSKAPSRTRRAKRDLPKRT
ATQRPEGTSLQQDPEAPTVPKKGRRKGRQAASGHCRPRKVKADIPSLEPEGTSAS
000000000000000000000000000000000000000000000000000000000000000000000000000000000000000000000000000000000000000000000000000000000000000000000000000000000000000000000
000000000000000000000000000000000000000000000000000000000000000000000000000000000000000000000000000000000000000000000000000000000111111111111111111111111111111111111111
11111111111111111111111111111111111X
000000000000000000000000000000000000000000000000000000000000000000000000000000000000000000000000000000000000000000000000000000000000000000000000000000000000000000000
000000000000000000000000000000000000000000000000000000000000000000000000000000000000000000000000000000000000000000000000000000000000000000000000000000000000000000000
00000000000000000000000000000000000X
000000000000000000000000000000000000000000000000000000000000000000000000000000000000000000000000000000000000000000000000000000000000000000000000000000000000000000000
000000000000000000000000000000000000000000000000000000000000000000000000000000000000000000000000000000000000000000000000000000000000000000000000000000000000000000000
00000000000000000000000000000000000X
>Q5UPT2
MSKKNVDPFSDSDSSSEPPSIFSSDNEENSDVDNSVIINDKNTKSDEADIKYMDEDESSDSESESESKKKSKKSKKSKKSKKSVTKKKNNLLVGNRIITEYILIDANNYHFKSWIECFPDCKVNLKLLLFRPEWFDFFKYVESKTYFPQLESKLSSYLEKRQRIVPYPELLFNT
MNVLPPGKIKVVILGQDPYPGSCISGVPYAMGCSFSVPLNCPVPKSLANIYTNLIKFNHMRKAPKHGCLASWILQGTFMINSAFTTVLNESGVHARTWESFTADLIDYLTDNYDDLIFVAWGAHAHKLCQRVDPKKHYIITSSHPSPYSVSNTMTSMSYGPNPKKVT
YPSFNSVDHFGKINEHLKSRNKKPIFWDL
111111111111111111111111111111111111111111111111111111111111111111111111111111111111111111111111000000000000000000000000000000000000000000000000000000000000000000000
000000000000000000000000000000000000000000000000000000000000000000000000000000000000000000000000000000000000000000000000000000000000000000000000000000000000000000000
00000000000000000
000000000000000000000000000000000000000000000000000000000000000000000000000000000000000000000000000000000000000000000000000000000000000000000000000000000000000000000
000000000000000000000000000000000000000000000000000000000000000000000000000000000000000000000000000000000000000000000000000000000000000000000000000000000000000000000
00000000000000000
000000000000000000000000000000000000000000000000000000000000000000000000000000000000000000000000000000000000000000000000000000000000000000000000000000000000000000000
000000000000000000000000000000000000000000000000000000000000000000000000000000000000000000000000000000000000000000000000000000000000000000000000000000000000000000000
00000000000000000
>Q9UBK2
MAWDMCNQDSESVWSDIECAALVGEDQPLCPDLPELDLSELDVNDLDTDSFLGGLKWCSDQSEIISNQYNNEPSNIFEKIDEENEANLLAVLTETLDSLPVDEDGLPSFDALTDGDVTTDNEASPSSMPDGTPPPQEAEEPSLLKKLLLAPANTQLSYNECSGLSTQ
NHANHNHRIRTNPAIVKTENSWSNKAKSICQQQKPQRRPCSELLKYLTTNDDPPHTKPTENRNSSRDKCTSKKKSHTQSQSQHLQAKPTTLSLPLTPESPNDPKGSPFENKTIERTLSVELSGTAGLTPPTTPPHKANQDNPFRASPKLKSSCKTVVPPPSKKPRYSESS
GTQGNNSTKKGPEQSELYAQLSKSSVLTGGHEERKTKRPSLRLFGDHDYCQSINSKTEILINISQELQDSRQLENKDVSSDWQGQICSSTDSDQCYLRETLEASKQVSPCSTRKQLQDQEIRAELNKHFGHPSQAVFDDEADKTGELRDSDFSNEQFSKLPMFINSGLA
MDGLFDDSEDESDKLSYPWDGTQSYSLFNVSPSCSSFNSPCRDSVSPPKSLFSQRPQRMRSRSRSFSRHRSCSRSPYSRSRSRSPGSRSSSRSCYYYESSHYRHRTHRNSPLYVRSRSRSPYSRRPRYDSYEEYQHERLKREEYRREYEKRESERAKQRERQRQKAIEERR
VIYVGKIRPDTTRTELRDRFEVFGEIEECTVNLRDDGDSYGFITYRYTCDAFAALENGYTLRRSNETDFELYFCGRKQFFKSNYADLDSNSDDFDPASTKSKYDSLDFDSLLKEAQRSLRR
X1111111111111111111111111111111111111111111111111111111111111111111111111111111111111111111111111111111111111111111111111111111111111111111111111111111111111111111
111111111111111111111111111111111111111111100000000000000000000000000000000000000000000000000000000000000000000000000000000000000000000000000000000000000000000000000
000000000000000000000000000000000000000000000000000000000000000000000000000000000000000000000000000000000000000000000000000000000000000000000000000000000000000000000
000000000000000000000000000000000000000000000000000000000000000000000000000000000000000000000000000000000000000000000000000000000000000000000000000000000000000000000
0000000000000000000000000000000000000000000000000000000000000000000000000000000000000000
X1111111111111111111111111111111111111111111111111111111111111111111111111111111111111111111111111111111111111111111111111111111111111111111111111111111111111111111
111111111111111111111111111111111111111111100000000000000000000000000000000000000000000000000000000000000000000000000000000000000000000000000000000000000000000000000
000000000000000000000000000000000000000000000000000000000000000000000000000000000000000000000000000000000000000000000000000000000000000000000000000000000000000000000
000000000000000000000000000000000000000000000000000000000000000000000000000000000000000000000000000000000000000000000000000000000000000000000000000000000000000000000
0000000000000000000000000000000000000000000000000000000000000000000000000000000000000000
X0000000000000000000000000000000000000000000000000000000000000000000000000000000000000000000000000000000000000000000000000000000000000000000000000000000000000000000
000000000000000000000000000000000000000000000000000000000000000000000000000000000000000000000000000000000000000000000000000000000000000000000000000000000000000000000
000000000000000000000000000000000000000000000000000000000000000000000000000000000000000000000000000000000000000000000000000000000000000000000000000000000000000000000
000000000000000000000000000000000000000000000000000000000000000000000000000000000000000000000000000000000000000000000000000000000000000000000000000000000000000000000
0000000000000000000000000000000000000000000000000000000000000000000000000000000000000000
>P22059
MAATELRGVVGPGPAAIAALGGGGAGPPVVGGGGGRGDAGPGSGAASGTVVAAAAGGPGPGAGGVAAAGPAPAPPTGGSGGSGAGGSGSAREGWLFKWTNYIKGYQRRWFVLSNGLLSYYRSKAEMRHTCRGTINLATANITVEDSCNFIISNGGAQTYHL
KASSEVERQRWVTALELAKAKAVKMLAESDESGDEESVSQTDKTELQNTLRTLSSKVEDLSTCNDLIAKHGTALQRSLSELESLKLPAESNEKIKQVNERATLFRITSNAMINACRDFLMLAQTHSKKWQKSLQYERDQRIRLEETLEQLAKQHNHLERAFRGATVLPA
NTPGNVGSGKDQCCSGKGDMSDEDDENEFFDAPEIITMPENLGHKRTGSNISGASSDISLDEQYKHQLEETKKEKRTRIPYKPNYSLNLWSIMKNCIGKELSKIPMPVNFNEPLSMLQRLTEDLEYHELLDRAAKCENSLEQLCYVAAFTVSSYSTTVFRTSKPFNPLL
GETFELDRLEENGYRSLCEQVSHHPPAAAHHAESKNGWTLRQEIKITSKFRGKYLSIMPLGTIHCIFHATGHHYTWKKVTTTVHNIIVGKLWIDQSGEIDIVNHKTGDKCNLKFVPYSYFSRDVARKVTGEVTDPSGKVHFALLGTWDEKMECFKVQPVIGENGGDA
RQRGHEAEESRVMLWKRNPLPKNAENMYYFSELALTLNAWESGTAPTDSRLRPDQRLMENGRWDEANAEKQRLEEKQRLSRKKREAEAMKATEDGTPYDPYKALWFERKKDPVTKELTHIYRGEYWECKEKQDWSSCPDIF
000000000000000000000000000000000000000000000000000000000000000000000000000000000000000000000000000000000000000000000000000000000000000000000000000000000000000000000
000000000000000000000000000000000000000000000000000000000000000000000000000000000000000000000000000000000000000000000000000000000000000000000000000000000000001111111
111111111111111111111111110000000000000000000000000000000000000000000000000000000000000000000000000000000000000000000000000000000000000000000000000000000000000000000
000000000000000000000000000000000000000000000000000000000000000000000000000000000000000000000000000000000000000000000000000000000000000000000000000000000000000000000
0000000000000000000000000000000000000000000000000000000000000000000000000000000000000000000000000
000000000000000000000000000000000000000000000000000000000000000000000000000000000000000000000000000000000000000000000000000000000000000000000000000000000000000000000
000000000000000000000000000000000000000000000000000000000000000000000000000000000000000000000000000000000000000000000000000000000000000000000000000000000000001111111
111111111111111111111111110000000000000000000000000000000000000000000000000000000000000000000000000000000000000000000000000000000000000000000000000000000000000000000
000000000000000000000000000000000000000000000000000000000000000000000000000000000000000000000000000000000000000000000000000000000000000000000000000000000000000000000
0000000000000000000000000000000000000000000000000000000000000000000000000000000000000000000000000
000000000000000000000000000000000000000000000000000000000000000000000000000000000000000000000000000000000000000000000000000000000000000000000000000000000000000000000
000000000000000000000000000000000000000000000000000000000000000000000000000000000000000000000000000000000000000000000000000000000000000000000000000000000000000000000
000000000000000000000000000000000000000000000000000000000000000000000000000000000000000000000000000000000000000000000000000000000000000000000000000000000000000000000
000000000000000000000000000000000000000000000000000000000000000000000000000000000000000000000000000000000000000000000000000000000000000000000000000000000000000000000
0000000000000000000000000000000000000000000000000000000000000000000000000000000000000000000000000
>Q9VMA3
MQMAEAEQENGAGALKIATNAGATDRPAHQQLPLPVEDQQDEVLTPAEKGKFEYPPPPPPPTPVQAPLATKATALNASQEHDDDEANSEKWEDPCAPPPPPPLPTSAFLATGLGYLKLPAFKLKDALEKAITKLEANKRTLKASPESSRSIKNKNVVALEMLPRRS
NPETIGDGSMLASTSTAVMLQTKKPAVIVEMERRCKIINLLAKQNQILESISGEAIPMHGPSKHLHEDEGLTLQVLSARASTPYTQPSSMLSCTAVSCDLEHDSPRKQVASKEAVPEQQSSQVQQKRPPSTGIHKPGSLRAPKAVRPTTAPVVSSKPVKSYTRSRLMDI
RNGMFNALMHRSKESFVMPRIATCDDIELEGRLRRMNIWRTSDGTRFRTRSTTANLNMNNNNNNECMPAFFKNKNKPNLISDESIIQSQPPQPQTEFQDPAIVNQRRIGSGRLNHSKWGYNDEDYHSYHNGKSQHMEEVNSKNSKNMTVLQFFDNGEISSQP
QRRPNTPVMGMSINRSENDTLHSNESSEDLSRANENYVKRVMSGFLVVSKPKSRDVEDRHHRRYRNQNEEPEWFSCGPTSRLDTIELCGFDEDEEKMLKEGNKNHGLGETERETSKQKMDHKYKWTHAEPMGRSKYMPKHDTNNNHNVENMNNVMATEH
QQQKEEKRPGSGRSFQFDKFNQSQQNYESSSYVNHQPPQTQPQQMQQQSNTNTNNSKFMSFFANEGNSSSSSLNEFFKQAINQGHGNNPEQPKSLGHIGQMPSVDQLEAKWRRNSLNNVGETANKQTDNFQKLIGSLSSAKPQSQAVGYDAISNFIMQQ
QQYQQQQQKQHLIIQQQQQHTAFLASLQLKAILGRADTQLLLLRLTKGEISKHGLLVQLANPRLTDMDREAITAVLQFTNTQQQQQQHKQQLDMLSSTVIASQLQNLHNLAIVQQTLAARQQPQHNPQTQAPHQLSQEDLQAHANVIMRNAVMKRKIEEQTS
KLINGGAKHQAQQQYLNRGQQRQARPDANSNALLHALISGGGNNHASGYPMNGQPQKHHSNLRFGDNQNFQSFESNQPHFATQYKQQYQQSQQQHPHQQPQQLNSLHQNNAGAVNSFNKAQMQAQSAISMLPNSGDEFH
000000000000000000000000000000000000000000000000000000000000000000000000000000000000000000000000000000000000000000000000000000000000000000000000000000000000000000000
000000000000000000000000000000000000000000000000000000000000000000000000000000000000000000000000000000000000000000000000000000000000000000000000000011111111111111
1111111111110000000000000000000000000000000000000000000000000000000000000000000000000000000000000000000000000000000000000000000000000000000000000000000000000000000
000000000000000000000000000000000000000000000000000000000000000000000000000000000000000000000000000000000000000000000000000000000000000000000000000000000000000000000
000000000000000000000000000000000000000000000000000000000000000000000000000000000000000000000000000000000000000000000000000000000000000000000000000000000000000000000

00000000000000000000000000000000000000000000000000000000000000000000000000000000000000000000000000000000000000000000000000000000000000000000000000000000000000000000000000000000
0000000000000000000000000000000000000000000000000000000000000
00000000000000000000000000000000000000000000000000000000000000000000000000000000000000000000000000000000000000000000000000000000000000000000000000000000000000000000000000000000
00000000000000000000000000000000000000000000000000000000000000000000000000000000000000000000000000000000000000000000000000000000000000000000000000000000000000000111111111111
11111111100000000000000000000000000000000000000000000000000000000000000000000000000000000000000000000000000000000000000000000000000000000000000000000000000000000000000000000000
00000000000000000000000000000000000000000000000000000000000000000000000000000000000000000000000000000000000000000000000000000000000000000000000000000000000000000000000000000000
00000000000000000000000000000000000000000000000000000000000000000000000000000000000000000000000000000000000000000000000000000000000000000000000000000000000000000000000000000000
00000000000000000000000000000000000000000000000000000000000000000000000000000000000000000000000000000000000000000000000000000000000000000000000000000000000000000000000000000000
0000000000000000000000000000000000000000000000000000000000000
00000000000000000000000000000000000000000000000000000000000000000000000000000000000000000000000000000000000000000000000000000000000000000000000000000000000000000000000000000000
00000000000000000000000000000000000000000000000000000000000000000000000000000000000000000000000000000000000000000000000000000000000000000000000000000000000000000000000000000000
00000000000000000000000000000000000000000000000000000000000000000000000000000000000000000000000000000000000000000000000000000000000000000000000000000000000000000000000000000000
00000000000000000000000000000000000000000000000000000000000000000000000000000000000000000000000000000000000000000000000000000000000000000000000000000000000000000000000000000000
00000000000000000000000000000000000000000000000000000000000000000000000000000000000000000000000000000000000000000000000000000000000000000000000000000000000000000000000000000000
00000000000000000000000000000000000000000000000000000000000000000000000000000000000000000000000000000000000000000000000000000000000000000000000000000000000000000000000000000000
0000000000000000000000000000000000000000000000000000000000000
>Q9NK80
MMHKLKYRDKLKWLLALLVLIGTCFIQTRGQTRDPRFYSRPGVDYHWPNPGDPDYRTYTFNDRRYGHYQPNGYGANYPGRNPPGQYPQGMPNEDRFRFDPNDPNARTQFPGVLAGWREDLQGKQRRDSLTLERDVFVTTNYGQVQGFKVYMYDNPDPKSF
YRPYHSTVDRVMGECSVFLGIPYALPPTFEGRFKPPRVHRGWQLLQAVDFGPACPQPVRYTGATKGIMDMDEDCLYLNVYSPKTGAGVAQKYPVMVYIHGGEFIRGASNLFQGHILASFYDVVVVTLNYRLGALGFLSTGDENSPGNYGILDQAMALRWVYDNI
EFFNGDRNSITLFGPGAGGASAGLLMVAPQTRNIVRRVIAQSGSALADWALIQDKYRAQNTSRVLGQLLGCSIESSWKLVNCLRTGRSFYELGNAEFSPQVGSFPWGPVLDHNFTLPGDDWYEGWREKDWRFLTKTPETLIRAGKFNRNIQYMTGVTTQEAAFF
VAQNESLSPYYELDGRFFDQKIREHVFRYNYTLNPNGVYEAIKYMYTFWPDPNNNTIIRDQYINMLSDLYYRAPVDQMVKLMLEQKVPVYMYVLNTTVEALNLPQWRKYPHDIERYFLTGAPFMDTEFFPKKEHLQRNMWTDNDRNMSHFFMQTYTNFARYG
NPTPQQVLGMHFQRAYQGEIRYLNINTTYNSSILLNYRQTECAFWTQYLPTVIGVLVPTYPPTTEYWWEPKEPLQIAFWSMSVACFFLIVLVVICCIMWRNAKRQSDRFYDEDVFINGEGLEPEQDTRGVDNAHMVTNHHALRSRDNIYEYRDSPSTKTLASKAHT
DTTSLRSPSSLAMTQKSSSQASLKSGISLKETNGHLVKQSERAATPRSQQNGSIAKVASPPVEEKRLLQPLSSTPVTQLQAEPAKRVPTAASVSGSSRSTTPVPSARSTTTHTTTATLSSQPAAQPRRTHLVEGVPQTSV
00000000000000000000000000000000000000000000000000000000000000000000000000000000000000000000000000000000000000000000000000000000000000000000000000000000000000000000000000000000
00000000000000000000000000000000000000000000000000000000000000000000000000000000000000000000000000000000000000000000000000000000000000000000000000000000000000000000000000000000
00000000000000000000000000000000000000000000000000000000000000000000000000000000000000000000000000000000000000000000000000000000000000000000000000000000000000000000000000000000
00000000000000000000000000000000000000000000000000000000000000000000000000000000000000000000000000000000000000000000000000000000000000000000000000000000000000000000000000000000
00000000000000000000000000000000011111111111111111111111111111111111111111111111111111111111111111111111111111111111111111111111111111111111111111111111111111111111111111111111
1111111111111111111111111111111111111111111111111111111111111111111111111111
00000000000000000000000000000000000000000000000000000000000000000000000000000000000000000000000000000000000000000000000000000000000000000000000000000000000000000000000000000000
00000000000000000000000000000000000000000000000000000000000000000000000000000000000000000000000000000000000000000000000000000000000000000000000000000000000000000000000000000000
00000000000000000000000000000000000000000000000000000000000000000000000000000000000000000000000000000000000000000000000000000000000000000000000000000000000000000000000000000000
00000000000000000000000000000000000000000000000000000000000000000000000000000000000000000000000000000000000000000000000000000000000000000000000000000000000000000000000000000000
00000000000000000000000000000000000000000000000000000000000000000000000000000000000000000000000000000000000000000000000000000000000000000000000000000000000000000000000000000000
0000000000000000000000000000000000000000000000000000000000000000000000000000
00000000000000000000000000000000000000000000000000000000000000000000000000000000000000000000000000000000000000000000000000000000000000000000000000000000000000000000000000000000
00000000000000000000000000000000000000000000000000000000000000000000000000000000000000000000000000000000000000000000000000000000000000000000000000000000000000000000000000000000
00000000000000000000000000000000000000000000000000000000000000000000000000000000000000000000000000000000000000000000000000000000000000000000000000000000000000000000000000000000
00000000000000000000000000000000000000000000000000000000000000000000000000000000000000000000000000000000000000000000000000000000000000000000000000000000000000000000000000000000
00000000000000000000000000000000000000000000000000000000000000000000000000000000000000000000000000000000000000000000000000000000000000000000000000000000000000000000000000000000
0000000000000000000000000000000000000000000000000000000000000000000000000000
>P33328
MSSSVPYDPYVPPEESNSGANPNSQNKTAALRQEIDDTVGIMRDNINKVAERGERLTSIEDKADNLAISAQGFKRGANRVRKQMWWKDLKMRMCLFLVVIILLVVIIVPIVVHFS
1111111111111111111111111111111111111111111111111111111111111111111111111111111111111111111XXXXXXXXXXXXXXXXXXXXXXXX
1111111111111111111111111111111111111111111111111111111111111111111111111111111111111111111XXXXXXXXXXXXXXXXXXXXXXXX
0000000000000000000000000000000000000000000000000000000000000000000000000000000000000000000XXXXXXXXXXXXXXXXXXXXXXXX
>P08263
MAEKPKLHYFNARGRMESTRWLLAAAGVEFEEKFIKSAEDLDKLRNDGYLMFQQVPMVEIDGMKLVQTRAILNYIASKYNLYGKDIKERALIDMYIEGIADLGEMILLLPVCPPEEKDAKLALIKEKIKNRYFPAFEKVLKSHGQDYLVGNKLSRADIHLVELLYYVEEL
DSSLISSFPLLKALKTRISNLPTVKKFLQPGSPRKPPMDEKSLEEARKIFRF
00000000000000000000000000000000000000000000000000000000000000000000000000000000000000000000000000000000000000000000000000000000000000000000000000000000000000000000000000000000
0000000000000000000000000000000111111111111111
00000000000000000000000000000000000000000000000000000000000000000000000000000000000000000000000000000000000000000000000000000000000000000000000000000000000000000000000000000000
0000000000000000000000000000000000000000000000
00000000000000000000000000000000000000000000000000000000000000000000000000000000000000000000000000000000000000000000000000000000000000000000000000000000000000000000000000000000
0000000000000000000000000000000000000000000000
>A0A1P8ATS2
MNLFERFSRVVKSYANALISSFEDPEKILEQTVIEMNSDLTKMRQATAQVLASQKQLQNKYKAAQQSSDDWYKRAQLALAKGDEDLAREALKRRKSFADNATALKTQLDQQKGVVDNLVSNTRLLESKIQEAKAKKDTLLARARTAKTATKVQEMIGTVNTSGALS
AFEKMEEKVMAMESEADALTQIGTDELEGKFQMLETSSVDDDLADLKKELSGSSKKGELPPGRSTVAASTRYPFKDSEIENELNELRRKANDF
00000000000000000000000000000000000000000000000000000000000000000000000000000000000000000000000000000000000000000000000000000000000000000000000000000000000000000000000000000000
00000000000000000000000000000000000000000000000001111111111111111111111111111111111
00000000000000000000000000000000000000000000000000000000000000000000000000000000000000000000000000000000000000000000000000000000000000000000000000000000000000000000000000000000
00000000000000000000000000000000000000000000000000000000000000000000000000000000000
00000000000000000000000000000000000000000000000000000000000000000000000000000000000000000000000000000000000000000000000000000000000000000000000000000000000000000000000000000000
00000000000000000000000000000000000000000000000000000000000000000000000000000000000
>Q02297-10
MEIYSPDMSEVAAERSSSPSTQLSADPSLDGLPAAEDMPEPQTEDGRTPGLVGLAVPCCACLEAERLRGCLNSEKICIVPILACLVSLCLCIAGLKWVFVDKIFEYDSPTHLDPGGLGQDPIISLDATAASAVWVSSEAYTSPVSRAQSESEVQVTVQGDKAVVSFEPSA
APTPKNRIFAFSFLPSTAPSFPSPTRNPEVRTPKSATQPQTTETNLQTAPKLSTSTSTTGTSHLVKCAEKEKTFCVNGGECFMVKDLSNPSRYLCKCPNEFTGDRCQNYVMASFYSTSTPFLSLPE
11111111111111111111111111111111111111111111111111111111111111111111111111111111111111111100000000000000000000000000000000000000000000000000000000000000000000000000000000000000
000000000000000000000000000000000000000000000000000000000000000000000000000000000000000000000000000000000000000000000000
00000000000000000000000000000000000000000000000000000000000000000000000000000000000000000000000000000000000000000000000000000000000000000000000000000000000000000000000000000000
000000000000000000000000000000000000000000000000000000000000000000000000000000000000000000000000000000000000000000000000
00000000000000000000000000000000000000000000000000000000000000000000000000000000000000000000000000000000000000000000000000000000000000000000000000000000000000000000000000000000
000000000000000000000000000000000000000000000000000000000000000000000000000000000000000000000000000000000000000000000000
>Q5RJL0
MTDTPVTLSGSECNGDRPPENGQQPSSQTRKTTDADETQTYYGVEPSLQHLPAKENQEESGNSKGNVLPRGSEDEKILNENTEENLFVVHQAIQDLSLQETSAEDTVFQEGHPWKKIPLNSHNLDMSRQKERIVHQHLEQREDESAAHQATEIEWLGFQKSSQV
DILHSKCDEEEEVWNEEINEEDVDECAEDEGEDEVRVIEFKRKYREGSPLKEESLAREDSPLSSPSSQPGTPDEQLVLGKKGDIARNSYSRYNTISYRKIRKGNTKQRIDEFESMMHL
11111111111111111111111111111111111111111111111111111111111111111111111111111111111111111111111111111111111111111111111111111111111111111111111111111111111111111111111111111111
1111111111111111111111111111111111111111111111111111111111111111111111111111111111111111111111111111111111
11111111111111111111111111111111111111111111111111111111111111111111111111111111111111111111111111111111111111111111111111111111111111111111111111111111111111111111111111111111
1111111111111111111111111111111111111111111111111111111111111111111111111111111111111111111111111111111111
00000000000000000000000000000000000000000000000000000000000000000000000000000000000000000000000000000000000000000000000000000000000000000000000000000000000000000000000000000000
0000000000000000000000000000000000000000000000000000000000000000000000000000000000000000000000000000000000
>Q9UJX6
MAAAVVVAEGDSDSRPGQELLVAWNTVSTGLVPPAALGLVSSRTSGAVPPKEEELRAAVEVLRGHGLHSVLEEWFVEVLQNDLQANISPEFWNAISQCENSADEPQCLLLLLDAFGLLESRLDPYLRSLELLEKWTRLGLLMGTGAQGLREEVHTMLRGVLFFSTP
RTFQEMIQRLYGCFLRVYMQSKRKGEGGTDPELEGELDSRYARRRYYRLLQSPLCAGCSSDKQQCWCRQALEQFHQLSQVLHRLSLLERVSAEAVTTTLHQVTRERMEDRCRGEYERSFLREFHKWIERVVGWLGKVFLQDGPARPASPEAGNTLRRWRCHVQR
FFYRIYASLRIEELFSIVRDFPDSRPAIEDLKYCLERTDQRQQLLVSLKAALETRLLHPGVNTCDIITLYISAIKALRVLDPSMVILEVACEPIRRYLRTREDTVRQIVAGLTGDSDGTGDLAVELSKTDPASLETGQDSEDDSGEPEDWVPDPVDADPGKSSSKRRSSDIISLL
VSIYGSKDLFINEYRSLLADRLLHQFSFSPEREIRNVELLKLRFGEAPMHFCEVMLKDMADSRRINANIREEDEKRPAEEQPPFGVYAVILSSEFWPPFKDEKLEVPEDIRAALEAYCKKYEQLKAMRTLSWKHTLGLVTMDVELADRTLSVAVTPVQAVILLYFQDQAS
WTLEELSKAVKMPVALLRRRMSVWLQQGVLREEPPGTFSVIEEERPQDRDNMVLIDSDDESDSGMASQADQKEEELLLFWTYIQAMLTNLESLSLDRIYNMLRMFVVTGPALAEIDLQELQGYLQKKVRDQQLVYSAGVYRLPKNCS
11111111111111XXXXXXXXXXXXXXX11111111111111111111111XXXXXXXXXXXXXXXXXXXXXXXXXXXXXXXXXXXXXXXXXXXXXXXXXXXXXXXXXXXXXXXXXXXXXXXXXXXXXXXXXXXXXXXXXXXXXXXXXXXXXXXXXXXXXXXXXXXXX
XXXXXXXXXXXXXXXXXXXXXXXXXXXXXXXXXXXXXXXXXXXXXXXXXXXXXXXXX11111111111XXXXXXXXXXXXXXXXXXXXXXXXXXXXXXXXXXXXXXXXXXXXXXXXXXXXXXXXXXXXXXXXXXXXXXXXXXXX111111111111111XXXXXXXXXXXXXXXXXX
XXXXXXXXXXXXXXXXXXXXXXXXXXXXXXXXXXXXXXXXXXXXXXXXXXXXXXXXXXXXXXXXXXXXXXXXXXXXXXXXXXXXXXXXXXXXXXXXXXXXXXXXXXXXXXXXXXXXXXXXXXXXXXXXXXX1111111111111111000000000000000000000000
00000000000000000000000000000000000000000000000000000000000000000000000000000000000000000000000000000000000000000000000000000000000000000000000000000000000000000000000000000000
000000000000000000000000000000000111111111111111111111111111111111111111111111111111111111111111111111111111111111111111111111111111
00000000000000XXXXXXXXXXXXXXX00000000000000000000000XXXXXXXXXXXXXXXXXXXXXXXXXXXXXXXXXXXXXXXXXXXXXXXXXXXXXXXXXXXXXXXXXXXXXXXXXXXXXXXXXXXXXXXXXXXXXXXXXXXXXXXXXXXXXXXXXXXXXXXXXXXX
XXXXXXXXXXXXXXXXXXXXXXXXXXXXXXXXXXXXXXXXXXXXXXXXXXXXXXXXX00000000000XXXXXXXXXXXXXXXXXXXXXXXXXXXXXXXXXXXXXXXXXXXXXXXXXXXXXXXXXXXXXXXXXXXXXXXXXXXX000000000000000XXXXXXXXXXXXXXXXXX
XXXXXXXXXXXXXXXXXXXXXXXXXXXXXXXXXXXXXXXXXXXXXXXXXXXXXXXXXXXXXXXXXXXXXXXXXXXXXXXXXXXXXXXXXXXXXXXXXXXXXXXXXXXXXXXXXXXXXXXXXXXXXXXXXXX0000000000000000000000000000000000000000
00000000000000000000000000000000000000000000000000000000000000000000000000000000000000000000000000000000000000000000000000000000000000000000000000000000000000000000000000000000
000000000000000000000000000000000000000000000000000000000000000000000000000000000000000000000000000000000000000000000000000000
00000000000000XXXXXXXXXXXXXXX00000000000000000000000XXXXXXXXXXXXXXXXXXXXXXXXXXXXXXXXXXXXXXXXXXXXXXXXXXXXXXXXXXXXXXXXXXXXXXXXXXXXXXXXXXXXXXXXXXXXXXXXXXXXXXXXXXXXXXXXXXXXXXXXXXXX
XXXXXXXXXXXXXXXXXXXXXXXXXXXXXXXXXXXXXXXXXXXXXXXXXXXXXXXXX00000000000XXXXXXXXXXXXXXXXXXXXXXXXXXXXXXXXXXXXXXXXXXXXXXXXXXXXXXXXXXXXXXXXXXXXXXXXXXXX000000000000000XXXXXXXXXXXXXXXXXX
XXXXXXXXXXXXXXXXXXXXXXXXXXXXXXXXXXXXXXXXXXXXXXXXXXXXXXXXXXXXXXXXXXXXXXXXXXXXXXXXXXXXXXXXXXXXXXXXXXXXXXXXXXXXXXXXXXXXXXXXXXXXXXXXXXX0000000000000000000000000000000000000000
00000000000000000000000000000000000000000000000000000000000000000000000000000000000000000000000000000000000000000000000000000000000000000000000000000000000000000000000000000000
000000000000000000000000000000000000000000000000000000000000000000000000000000000000000000000000000000000000000000000000000000
>Q57904
MFGSIFDIYYKTLDAIFMPIIKVLHPALAILIIAIIVSLIINIATKLLVDQKRVAELKKEIQEFQVKFKKMSKNPEMMEKLQEEQQRIMQLNAELMKMSFRPMIYTWVPIILIFIYLRHVYGFGGVYQELNPGWNGVVVYLPIILSKILFIDFWHWLGSIFYKGGFKIVSNTAL
GWLGWYILCSFATSTVLRKILGIK
00000000000000000000000000000000000000000000111111111111111111111111111111111111111111111111111111110000000000000000000000000000000000000000000000000000000000000000000000000000
0000000000000000000000
00000000000000000000000000000000000000000000000000000000000000000000000000000000000000000000000000000000000000000000000000000000000000000000000000000000000000000000000000000000
0000000000000000000000
00000000000000000000000000000000000000000000000000000000000000000000000000000000000000000000000000000000000000000000000000000000000000000000000000000000000000000000000000000000
0000000000000000000000
>O75554
MADYWKSQPKKFCDYCKCWIADNRPSVEFHERGKNHKENVAKRISEIKQKSLDKAKEEEKASKEFAAMEAAALKAYQEDLKRLGLESEILEPSITPVTSTIPPTSTSNQQKEKKEKKKRKKDPSKGRWVEGITSEGYHYYYDLISGASQWEKPEGFQGDLKKTAVKTV
WVEGLSEDGFTYYYNTETGESRWEKPDDFIPHTSDLPSSKVNENSLGTLDESKSSDSHSDSDGEQEAEEGGVSTETEKPKIKFKEKNKNSDGGSDPETQKEKSIQKQNSLGSNEEKSKTLKKSNPYGEWQEIKQEVESHEEVDLELPSTENEYVSTSEADGGGEPKVVF
KEKTVTSLGVMADGVAPVFKKRRTENGKSRNLRQRGDDQ
00000000000000000000000000000000000000000000000000000000000000000000000000000000000000000000000000000000000000000000000000000000000000000000000000000000000000000000000000000000
00000000000000000000000000000000000000000000000000000000000000000000000000000000000000000000000001111111111111111111111111111111111111111111111111111111111111111111111111111111
111111111111111111111111
00000000000000000000000000000000000000000000000000000000000000000000000000000000000000000000000000000000000000000000000000000000000000000000000000000000000000000000000000000000
00000000000000000000000000000000000000000000000000000000000000000000000000000000000000000000000001111111111111111111111111111111111111111111111111111111111111111111111111111111
111111111111111111111111
00000000000000000000000000000000000000000000000000000000000000000000000000000000000000000000000000000000000000000000000000000000000000000000000000000000000000000000000000000000
00000000000000000000000000000000000000000000000000000000000000000000000000000000000000000000000000000000000000000000000000000000000000000000000000000000000000000000000000000000
000000000000000000000000
>B6KJB6
MLQMARYTANICAVSICSLVLVVALSVGILPTPDWKDRVKMGGTESGPFVLQVCASDPLLHDPKERESGSDSTRGYHGGSSSGGSSSRQGTTVRSDAGLSSQSSQSSASTSAKTSENHRQGPAFLTSVFRKGETPALDWVPYGTLEGAKWHPGQQKSKRRSSATT
SRRQGASHSGNPGQLPAPRGGLQPTTTLSGTGGQPRTDSTDEGAAATSVIPNRSGYPQPVPYLIHPVGFLSGDYRSLGMLGLVPSVYTATSVQHMVGQPGTIIPLVLLPGKQEPEGLVSTGTLSDSLVYEPLGVVNLGTEMPNQGSTSQSGAVASRKRPAGGANG

PDKRRRVEPAGLTESRLRPEPSLSSLTEKGSTAFSTRPPSSRSALEGLTQETVEMLLDTPSYPISSVVSSPPPARKSSTSSSQHLEARLSQSRGPPRTRPPFNPWSTKTGLLERRGVSELPPLYIPRPLASGYRNPADSRKHSTVTPQTTPPARKSSTSSSQHLEARLSQSRG
PPRTRPPFNPWSTKTGLLERRGVSELPPLRIVKPPTKGN
XXXXXXXXXXXXXXXXXXXXXXXXXXXXXXXXXXXXXXXXXXXXXXXXXXXXXXXXXXXXXXXXXXXXXXXXXXXXXXXXXXXXXXXXXXXXXXXXXXXXXXXXXXXXXXXXXXXXXXXXXXXXXXXXXXXXXXXXXXXXXXXXXXXXXXXXXX
XXXXXXXXXXXXXXXXXXXXXXXXXXXXXXXXXXXXXXXXXXXXXXXXXXXXXXXXXXXXXXXXXXXXXXXXXXXXXXXXXXXXXXXXXXXXXXXXXXXXXXXXXXXXXXXXXXXXXXXXXXXXXXXXXXXXXXXXXXXXXXXXXXXXX11111111
11111111111111111111111111111111111111111111111111111111111111111111111111111111111111111111111111111111111111111111111111111111111111111111111111111111111111111111111111111111
1111111111111111111111
XXXXXXXXXXXXXXXXXXXXXXXXXXXXXXXXXXXXXXXXXXXXXXXXXXXXXXXXXXXXXXXXXXXXXXXXXXXXXXXXXXXXXXXXXXXXXXXXXXXXXXXXXXXXXXXXXXXXXXXXXXXXXXXXXXXXXXXXXXXXXXXXXXXXXXXXXXXXX
XXXXXXXXXXXXXXXXXXXXXXXXXXXXXXXXXXXXXXXXXXXXXXXXXXXXXXXXXXXXXXXXXXXXXXXXXXXXXXXXXXXXXXXXXXXXXXXXXXXXXXXXXXXXXXXXXXXXXXXXXXXXXXXXXXXXXXXXXXXXXXXXXXXXXX11111111
11111111111111111111111111111111111111111111111111111111111111111111111111111111111111111111111111111111111111111111111111111111111111111111111111111111111111111111111111111111
1111111111111111111111
XXXXXXXXXXXXXXXXXXXXXXXXXXXXXXXXXXXXXXXXXXXXXXXXXXXXXXXXXXXXXXXXXXXXXXXXXXXXXXXXXXXXXXXXXXXXXXXXXXXXXXXXXXXXXXXXXXXXXXXXXXXXXXXXXXXXXXXXXXXXXXXXXXXXXXXXXXXXX
XXXXXXXXXXXXXXXXXXXXXXXXXXXXXXXXXXXXXXXXXXXXXXXXXXXXXXXXXXXXXXXXXXXXXXXXXXXXXXXXXXXXXXXXXXXXXXXXXXXXXXXXXXXXXXXXXXXXXXXXXXXXXXXXXXXXXXXXXXXXXXXXXXXXXX00000000
00000000000000000000000000000000000000000000000000000000000000000000000000000000000000000000000000000000000000000000000000000000000000000000000000000000000000000000000000000000
0000000000000000000000
>O57173
MLSMFMCNNIVDYVDGIVQDIEDEASNNVDHDYVYPLPENMVYRFDKSTNILDYLSTERDHVMMAVRYYMSKQRLDDLYRQLPTKTRSYIDIINIYCDKVSNDYNRDMNIMYDMASTKSFTVYDINNEVNTILMDNKGLGVRLATISFITELGRRCMNPVKTIKM
FTLLSHTICDDCFVDYITDISPPDNTIPNTSTREYLKLIGITAIMFATYKTLKYMIG
11111111111111111111111111111111111111111111111111000000000000000000000000000000000000000000000000000000000000000000000000000000000000000000000000000000000000000000000000000000
0000000000000000000000000000000000000000000000
00000000000000000000000000000000000000000000000000000000000000000000000000000000000000000000000000000000000000000000000000000000000000000000000000000000000000000000000000000000
0000000000000000000000000000000000000000000000
00000000000000000000000000000000000000000000000000000000000000000000000000000000000000000000000000000000000000000000000000000000000000000000000000000000000000000000000000000000
0000000000000000000000000000000000000000000000
>Q9DBZ9
MAEPLLRKTFSRLRGREKLPRKKSEAKDRGHPAQRSEPKPPEPEPRVLEGSQAGAEVPPSPETPRSPTRGAYLQSLEPSSRRWVLGGAKPPEEISLGPRTPSSGEPAGEIWYNPIPEEDPRPPAPEPLGSQLASSEPEGPNIQGAAPTSPPTKTSRTKSPGPARRLSMK
MKKLPELRRRLSLRSTRTGRDRERTAPAGSVISRYRLDSSVGTPGQASVAGGSRSPRGGYLSDGDSPERPGGPPSPTAFRPYEVGPSARTPPAALWGRLSLHLYGLGGLRPSPGATPRDLCCLLQVDGVARARTGPLRSGPDFLRLDHTFHLELEAARLLRALVLAWD
PGVRRHRPCAQGTVLLPTIFRGCQAQQLAVRLEPQGFLYAKLTLSEQQEAPATAEPRVFGLPLQLLVEREQSPGQVPLIIRKCVGQIECRGLRVVGLYRLCGSAAVKKELRDAFEQDSAAVCLSEDVYPDINVITGILKDYLRELPTPLITQPLYQVVLEAMAQGHPSRA
SLGPEGTRGLLRCLPDVERATLTLLLDHLRLVSSFHTHNRMTPQNXAVCFGPVLLPARQTPSRPRLRSSGPGVTSAVDFKRHIEVLHYLLQSWPDTRRPSDTPDGAVAPYLRPKRQPPLHLPLAGPEVVTRPRGRGGPESPPSNRYAGDWSVCGGDLLPCGRDFLSG
PDYDHVTGSDSEEDDDETGEPRGTTDFEDEFDAPFNPHLNLKDFDALILDLERELSKQINVCL
11111111111111111111111111111111111111111111111111111111111111111111111111111111111111111111111111111111111111111111111111111111111111111111111111111111111111111111111111111111
11111111111111111111111111111111111111111111111111111111111111111111111111111111111111111111111111111111110000000000000000000000000000000000000000000000000000000000000000000000
00000000000000000000000000000000000000000000000000000000000000000000000000000000000000000000000000000000000000000000000000000000000000000000000000000000000000000000000000000000
00000000000000000000000000000000000000000000000000000000000000000000000000000000000000000000000000000000000000000000000000000000000000000000000000000000000000000000000000000000
000000000000000000000000000000000
11111111111111111111111111111111111111111111111111111111111111111111111111111111111111111111111111111111111111111111111111111111111111111111111111111111111111111111111111111111
11111111111111111111111111111111111111111111111111111111111111111111111111111111111111111111111111111111110000000000000000000000000000000000000000000000000000000000000000000000
00000000000000000000000000000000000000000000000000000000000000000000000000000000000000000000000000000000000000000000000000000000000000000000000000000000000000000000000000000000
00000000000000000000000000000000000000000000000000000000000000000000000000000000000000000000000000000000000000000000000000000000000000000000000000000000000000000000000000000000
000000000000000000000000000000000
00000000000000000000000000000000000000000000000000000000000000000000000000000000000000000000000000000000000000000000000000000000000000000000000000000000000000000000000000000000
00000000000000000000000000000000000000000000000000000000000000000000000000000000000000000000000000000000000000000000000000000000000000000000000000000000000000000000000000000000
00000000000000000000000000000000000000000000000000000000000000000000000000000000000000000000000000000000000000000000000000000000000000000000000000000000000000000000000000000000
00000000000000000000000000000000000000000000000000000000000000000000000000000000000000000000000000000000000000000000000000000000000000000000000000000000000000000000000000000000
000000000000000000000000000000000
>P06401
MTELKAKGPRAPHVAGGPPSPEVGSPLLCRPAAGPFPGSQTSDTLPEVSAIPISLDGLLFPRPCQGQDPSDEKTQDQQSLSDVEGAYSRAEATRGAGGSSSSPPEKDSGLLDSVLDTLLAPSGPGQSQPSPPACEVTSSWCLFGPELPEDPPAAPATQRVLSPLMSRS
GCKVGDSSGTAAAHKVLPRGLSPARQLLLPASESPHWSGAPVKPSPQAAAVEVEEEDGSESEESAGPLLKGKPRALGGAAAGGGAAAVPPGAAAGGVALVPKEDSRFSAPRVALVEQDAPMAPGRSPLATTVMDFIHVPILPLNHALLAARTRQLLEDESYDGGA
GAASAFAPPRSSPCASSTPVAVGDFPDCAYPPDAEPKDDAYPLYSDFQPPALKIKEEEEGAEASARSPRSYLVAGANPAAFPDFPLGPPPPLPPRATPSRPGEAAVTAAPASASVSSASSSGSTLECILYKAEGAPPQQGPFAPPPCKAPGASGCLLPRDGLPSTSASAA
AAGAAPALYPALGLNGLPQLGYQAAVLKEGLPQVYPPYLNYLRPDSEASQSPQYSFESLPQKICLICGDEASGCHYGVLTCGSCKVFFKRAMEGQHNYLCAGRNDCIVDKIRRKNCPACRLRKCCQAGMVLGGRKFKKFNKVRVVRALDAVALPQPVGVPNESQAL
SQRFTFSPGQDIQLIPPLINLLMSIEPDVIYAGHDNTKPDTSSSLLTSLNQLGERQLLSVVKWSKSLPGFRNLHIDDQITLIQYSWMSLMVFGLGWRSYKHVSGQMLYFAPDLILNEQRMKESSFYSLCLTMWQIPQEFVKLQVSQEEFLCMKVLLLLNTIPLEGLRSQT
QFEEMRSSYIRELIKAIGLRQKGVVSSSQRFYQLTKLLDNLHDLVKQLHLYCLNTFIQSRALSVEFPEMMSEVIAAQLPKILAGMVKPLLFHKK
11111111111111111111111111111111111111111111111111111111111111111111111111111111111111111111111111111111111111111111111111111111111111111111111111111111111111111111111111111111
11111111111111111111111111111111111111111111111111111111111111111111111111111111111111111111111111111111111111111111111111111111111111111111111111111111111111111111111111111111
11111111111111111111111111111111111111111111111111111111111111111111111111111111111111111111111111111111111111111111111111111111111111111111111111111111111111111111111111111111
11111111111111111111111111111111111111111111111111111000000000000000000000000000000000000000000000000000111111111111111111111110000000000000000000000000000000000000000000000000
00000000000000000000000000000000000000000000000000000000000000000000000000000000000000000000000000000000000000000000000000000000000000000000000000000000000000000000000000000000
00000000000000000000000000000000000000000000000000000
11111111111111111111111111111111111111111111111111111111111111111111111111111111111111111111111111111111111111111111111111111111111111111111111111111111111111111111111111111111
11111111111111111111111111111111111111111111111111111111111111111111111111111111111111111111111111111111111111111111111111111111111111111111111111111111111111111111111111111111
11111111111111111111111111111111111111111111111111111111111111111111111111111111111111111111111111111111111111111111111111111111111111111111111111111111111111111111111111111111
11111111111111111111111111111111111111111111111111111000000000000000000000000000000000000000000000000000111111111111111111111110000000000000000000000000000000000000000000000000
00000000000000000000000000000000000000000000000000000000000000000000000000000000000000000000000000000000000000000000000000000000000000000000000000000000000000000000000000000000
00000000000000000000000000000000000000000000000000000
00000000000000000000000000000000000000000000000000000000000000000000000000000000000000000000000000000000000000000000000000000000000000000000000000000000000000000000000000000000
00000000000000000000000000000000000000000000000000000000000000000000000000000000000000000000000000000000000000000000000000000000000000000000000000000000000000000000000000000000
00000000000000000000000000000000000000000000000000000000000000000000000000000000000000000000000000000000000000000000000000000000000000000000000000000000000000000000000000000000
00000000000000000000000000000000000000000000000000000000000000000000000000000000000000000000000000000000111111111111111111111110000000000000000000000000000000000000000000000000
00000000000000000000000000000000000000000000000000000000000000000000000000000000000000000000000000000000000000000000000000000000000000000000000000000000000000000000000000000000
00000000000000000000000000000000000000000000000000000
>M5BF30
MEAKIVKVLDSSRCEDGFGKKRKRAASYAAYVTGVSCAKLQNVPPPNGQCQIPDKRRRLEGENKLSAYENRSGKALVRYYTYFKKTGIAKRVMMYENGEWNDLPEHVICAIQNELEEKSAAIEFKLCGHSFILDFLHMQRLDMETGAKTPLAWIDNAGKCFFPEIYE
SDERTNYCHHKCVEDPKQNAPHDIKLRLEIDVNGGETPRLNLEECSDESGDNMMDDVPLAQRSSNEHYDEATEDSCSRKLEAAVSKWDETDAIVVSGAKLTGSEVLDKDAVKKMFAVGTASLGHVPVLDVGRFSSEIAEARLALFQKQVEITKKHRGDANVRYAW
LPAKREVLSAVMMQGLGVGGAFIRKSIYGVGIHLTAADCPYFSARYCDVDENGVRYMVLCRVIMGNMELLRGDKAQFFSGGEEYDNGVDDIESPKNYIVWNINMNTHIFPEFVVRFKLSNLPNAEGNLIAKRDNSGVTLEGPKDLPPQLESNQGARGSGSANSV
GSSTTRPKSPWMPFPTLFAAISHKVAENDMLLINADYQQLRDKKMTRAEFVRKLRVIVGDDLLRSTITTLQNQPKSKEIPGSIRDHEEGAGGL
00000000000000000000000000000000000000000000000000000000000000000000000000000000000000000000000000000000000000000000000000000000000000000000000000000000000000000000000000000000
00000000000000000000000000000000000000000000000000000000000000000000000000000000000000000000000000000000000000000000000000000000000000000000000000000000000000000000000000000000
00000000000000000000000000000000000000000000000000000000000000000000000000000000000000000000000000000000000000000000000000000000000000000000000000000000000000000000000000000000
0000000000000000000000000000000000000000011111111111111111111
00000000000000000000000000000000000000000000000000000000000000000000000000000000000000000000000000000000000000000000000000000000000000000000000000000000000000000000000000000000
00000000000000000000000000000000000000000000000000000000000000000000000000000000000000000000000000000000000000000000000000000000000000000000000000000000000000000000000000000000
00000000000000000000000000000000000000000000000000000000000000000000000000000000000000000000000000000000000000000000000000000000000000000000000000000000000000000000000000000000
0000000000000000000000000000000000000000000000000000000000000
00000000000000000000000000000000000000000000000000000000000000000000000000000000000000000000000000000000000000000000000000000000000000000000000000000000000000000000000000000000
00000000000000000000000000000000000000000000000000000000000000000000000000000000000000000000000000000000000000000000000000000000000000000000000000000000000000000000000000000000
00000000000000000000000000000000000000000000000000000000000000000000000000000000000000000000000000000000000000000000000000000000000000000000000000000000000000000000000000000000
0000000000000000000000000000000000000000000000000000000000000
>Q9XTY3
MIGAVSLVFFTVGVAAIYGLDLENAGDFVLPSGDFIDLISRDSENLPDLSSFGVKHISGGAGEGEQKLLEEDNFQERQEVKVDNVLPAYCEPPNPCPVGFTKEHGCLEEFENSAEFSRNYQADQHCICDQEHMFNCAEKEAQDVSESLQKILEENNMHANTIAKKFH
DKRSSDEYVPRRKRSIPSSAHKVNPYLQGEPLRSMQKKNGKHSW
XXXXXXXXXXXXXXXX1111111111111111111111111111111111111111111111111111111111111111111111111111111111111111111111111111111111111111111111111111111111111111111111111111111111111111
11111111111111111111111111111111111
XXXXXXXXXXXXXXXX1111111111111111111111111111111111111111111111111111111111111111111111111111111111111111111111111111111111111111111111111111111111111111111111111111111111111111
11111111111111111111111111111111111
XXXXXXXXXXXXXXXX0000000000000000000000000000000000000000000000000000000000000000000000000000000000000000000000000000000000000000000000000000000000000000000000000000000000000000
00000000000000000000000000000000000
>Q04964
MQNSQDYFYAQNRCQQQQAPSTLRTVTMAEFRRVPLPPMAEVPMLSTQNSMGSSASASASSLEMWEKDLEERLNSIDHDMNNNKFGSGELKSMFNQGKVEEMDF
11111111111111111111111111111111111111111111111111111111111111111111111111111111111111111111111111111111
00000000000000000000000000000000000000000000000000000000000000000000000000000000000000000000000000000000
00000000000000000000000000000000000000000000000000000000000000000000000000000000000000000000000000000000
>Q9CXY6
MRGDRGRGRGGRFGSRGGPGGGFRPFVPHIPFDFYLCEMAFPRVKPAPDETSFSEALLKRNQDLAPNSAEQASILSLVTKINNVIDNLIVAPGTFEVQIEEVRQVGSYKKGTMTTGHNVADLVVILKILPTLEAVAALGNKVVESLRAQDPSEVLTMLTNETGFEISSS
DATVKILITTVPPNLRKLDPELHLDIKVLQSALAAIRHARWFEENASQSTVKVLIRLLKDLRIRFPGFEPLTPWILDLLGHYAVMNNPTRQPLALNVAYRRCLQILAAGLFLPGSVGITDPCESGNFRVHTVMTLEQQDMVCYTAQTLVRILSHGGFRKILGQEGDASYL
ASEISTWDGVIVTPSEKAYEKPPEKKEGEEEEENTEEPPQGEEEESMETQE
XXXXXXXXXXXXXXXXXXXXXXXXX11111111111111000000000000000000000000000000000000000000000000000000000000000000000000000000000000000000000000000000000000000000000000000000000000000
00000000000000000000000000000000000000000000000000000000000000000000000000000000000000000000000000000000000000000000000000000000000000000000000000000000000000000000111111
11111111111111111111111111111111111111
XXXXXXXXXXXXXXXXXXXXXXXXX000000000000000000000000000000000000000000000000000000000000000000000000000000000000000000000000000000000000000000000000000000000000000000000000000000
00000000000000000000000000000000000000000000000000000000000000000000000000000000000000000000000000000000000000000000000000000000000000000000000000000000000000000000000000000000
00000000000000000000000000000000000000
XXXXXXXXXXXXXXXXXXXXXXXXX000000000000000000000000000000000000000000000000000000000000000000000000000000000000000000000000000000000000000000000000000000000000000000000000000000
00000000000000000000000000000000000000000000000000000000000000000000000000000000000000000000000000000000000000000000000000000000000000000000000000000000000000000000000000000000
00000000000000000000000000000000000000
>Q12905
MRGDRGRGRGGRFGSRGGPGGGFRPFVPHIPFDFYLCEMAFPRVKPAPDETSFSEALLKRNQDLAPNSAEQASILSLVTKINNVIDNLIVAPGTFEVQIEEVRQVGSYKKGTMTTGHNVADLVVILKILPTLEAVAALGNKVVESLRAQDPSEVLTMLTNETGFEISSS
DATVKILITTVPPNLRKLDPELHLDIKVLQSALAAIRHARWFEENASQSTVKVLIRLLKDLRIRFPGFEPLTPWILDLLGHYAVMNNPTRQPLALNVAYRRCLQILAAGLFLPGSVGITDPCESGNFRVHTVMTLEQQDMVCYTAQTLVRILSHGGFRKILGQEGDASYL
ASEISTWDGVIVTPSEKAYEKPPEKKEGEEEEENTEEPPQGEEEESMETQE
00000000000000000000000000000000000000000000000000000000000000000000000000000000000000000000000000000000000000000000000000000000000000000000000000000000000000000000000000000000
00000000000000000000000000000000000000000000000000000000000000000000000000000000000000000000000000000000000000000000000000000000000000000000000000000000000000000000000000000000
00000000011111111111111111111111111111
00000000000000000000000000000000000000000000000000000000000000000000000000000000000000000000000000000000000000000000000000000000000000000000000000000000000000000000000000000000
00000000000000000000000000000000000000000000000000000000000000000000000000000000000000000000000000000000000000000000000000000000000000000000000000000000000000000000000000000000
00000000000000000000000000000000000000
00000000000000000000000000000000000000000000000000000000000000000000000000000000000000000000000000000000000000000000000000000000000000000000000000000000000000000000000000000000
00000000000000000000000000000000000000000000000000000000000000000000000000000000000000000000000000000000000000000000000000000000000000000000000000000000000000000000000000000000
00000000000000000000000000000000000000
>P41969

MDPSVTLWQFLLQLLREQGNGHIISWTSRDGGEFKLVDAEEVARLWGLRKNKTNMNYDKLSRALRYYYDKNIIRKVSGQKFVYKFVSYPEVAGCSTEDCPPQPEVSVTSAIAMAPATVHAGPGDTATGKPGTPKGAGMTGQGGLARSSRNEYMRSGLYSTFTIQ
SLQPQPQPPIPPRPASVLPNTTPAGVPAPASGSRSTSPNPLEACLEAEEAGLPLQVILTPPEAPNQKSEELSLDPSFGHPQPPEVKVEGPKEELEAARAGGFSSEAVKAEPEVSASEGLLARLPAILTENTAQVCGLSTSTTEITQPQKGRKPRDLELPLSPSLLGGQGPER
TPGSGTSSGLQAPGPALTPSLLPTHTLTPVLLTPSSLPPSIHFWSTLSPIAPRSPAKLSFQFPSSGSAQVHIPSISVDGLSTPVVLSPGPQKP
[illegible]
>P34233
MSSLYIKTPLHALSAGPDSHANSSYYDNLLLPSFSNLSSNISRNNITTDNNINSASPRKYSFHSLNVSPILSPISLANEILGKKSNTAPASPHHMDYNPISSLTPGNSPEFNKASLSQISFTNPLNYGSGLGFSSNSQPRLPLLDRLSSVSLSKRPERPQQSLPSLRHLQLLPSPL
LQENAARFPDTSKRTSNWKTDLTHWCKDTNYQDYVKIREEVAHFKPLSIPNLTNNQNNDSFNYGKELESTRSSKFHSPSKESFDRTKLIPSILEAKDQFKDLSNNAWSITPPVTPPMSPPTNRTMERTTLRGVEASFFEGKSSNNDSIFNPIISEKLVQEVKHQRQLRG
NSFPMPNASHKKTNSFKALQIKKLLANRDILSNNSKSNVRKPSKNKISKQASNVFGNTARQLVMKLDNASYSSVSASSSPSPSTPTKSGKMRSRSSSPVRPKAYTPSPRSPNYHRFALDSPPQSPRRSSNSSITKKGSRRSSGSSPTRHTTRVCVSCHSSDSPCWRPSW
SPRKQDQLCNSCGLRYKKTHTRCLNDLCRKIPTKGEINIMKSNGIDKEFVPERNCEIEGYRCLFCNYITETVEN
[illegible]
>Q8NQJ3
MSDNNGTPEPQVETTSVFRADLLKEMESSTGTAPASTGAENLPAGSALLVVKRGPNAGARFLLDQPTTTAGRHPESDIFLDDVTVSRRHAEFRINEGEFEVVDVGSLNGTYVNREPRNAQVMQTGDEIQIGKFRLVFLAGPAE
[illegible]
>Q868N5
MLLLLTLLLFAGTVAADFQHNWQVGNEYTYLVRSRTLTSLGDLSDVHTGILIKALLTVQAKDSNVLAAKVWNGQYARVQQSMPDGWETEISDQMLELRDLPISGKPFQIRMKHGLIRDLIVDRDVPTWEVNILKSIVGQLQVDTQGENAVKVNSVQVPTDDEPYA
SFKAMEDSVGGKCEVLYDIAPLSDFVIHRSPELVPMPTLKGDGRHMEVIKIKNFDNCDQRINYHFGMTDNSRLEPGTNKNGKFFSRSSTSRIVISESLKHFTIQSSVTTSKMMVSPRLYDRQNGLVLSRMNLTLAKMEKTSKPLPMVDNPESTGNLVYIYNNPFSDVE
ERRVSKTAMNSNQIVSDNSLSSSEEKLKQDILNLRTDISSSSSSISSSEENDFWQPKPTLEDAPQNSLLPNFVGYKGKHIGKSGKVDVINAAKELIFQIANELEDASNIPVHATLEKFMILCNLMRTMNRKQISELESNMQISPNELKPNDKSQVIKQNTWTVFRDAITQ
TGTGPAFLTIKEWIERGTTKSMEAANIMSKLPKTVRTPTDSYIRSFFELLQNPKVSNEQFLNTAATLSFCEMIHNAQVNKRSIHNNYPVHTFGRLTSKHDNSLYDEYIPFLERELRKAHQEKDSPRIQTYIMALGMIGEPKILSVFEPYLEGKQQMTVFQRTLMVGSLGK
LTETNPKLARSVLYKIYLNTMESHEVRCTAVFLLMKTNPPLSMLQRMAEFTKLDTNRQVNSAVKSTIQSLMKLKSPEWKDLAKKARSVNHLLTHHEYDYELSRGYIDEKILENQNIITHMILNYVGSEDSVIPRILYLTWYSSNGDIKVPSTKVLAMISSVKSFMELSLRS
VKDRETIISAAEKIAEELKIVPEELVPLEGNLMINNKYALKFFPFDKHILDKLPTLISNYIEAVKEGKFMNVNMLDTYESVHSFPTETGLPFVYTFNVIKLTKTSGTVQAQINPDFAFIVNSNLRLTFSKNVQGRVGFVTPFEHRHFISGIDSNLHVYAPLKISLDVNTPKGN
MQWKIWPMKGEEKSRLFHYSVVPFVSNHDILNLRPLSMEKGTRPMIPDDNTSLALPKNEGPFRLNVETAKTNEEMWELIDTEKLTDRLPYPWTMDNERYVKVDMYMNLEGEQKDPVIFSTSFSDSKVMTRPDTDSENWTPKMMAVEPTDKQANSKTRRQEM
MREAGRGIESAKSYVVDVRVHVPGESESETVLTLAWSESNVESKGRLLGFWRVEMPRSNADYEVCIGSQIMVSPETLLSYDEKMDQKPKMDFNVDIRYGKNCGKGERIDMNGKLRQSPRLKELVGATSIIKDCVEDMKRGNKILRTCQKAVVLSMLLDEVDISME
VPSDALIALYSQGLFSLSEIDNLDVSLDVSNPKNAGKKKIDVRAKLNEYLDKADVIVNTPIMDAHFKDVKLSDFGFSTEDILDTADEDLLINNVFYEDETSCMLDKTRAQTFDGKDYPLRLGPCWHAVMTTYPRINPDNHNEKLHIPKDKSVSVLSRENEAGQKEVKVL
LGSDKIKFVPGTTSQPEVFVNGEKIVVSRNKAYQKVEENEIIFEIYKMGDRFIGLTSDKFDVSLALDGERVMLKASEDYRYSVRGLCGNFDHDSTNDFVGPKNCLFRKPEHFVASYALISNQCEGDSLNVAKSLQDHDCIRQERTQQRNVISDSESGRLDTEMSTWGY
HHNVNKHCTIHRTQVKETDDKICFTMRPVVSCASGCTACVETKSKPYKFHCMEKNEAAMKLKKRIEKGANPDLSQKPVSTTEELTVPFVCKA
[illegible]
>P9WJA9
MTDMNPDIEKDQTSDEVTVETTSVFRADFLSELDAPAQAGTESAVSGVEGLPPGSALLVVKRGPNAGSRFLLDQAITSAGRHPDSDIFLDDVTVSRRHAEFRLENNEFNVVDVGSLNGTYVNREPVDSAVLANGDEVQIGKFRLVFLTGPKQGEDDGSTGGP
[illegible]
>P21374
MSRNVDKANSVLVRFQEQQAESAGGYKDYSRYQRPRNVSKVKSIKEANEWKRQVSKEIKQKSTRIYDPSLNEMQIAELNDELNNLFKEWKRWQWHIDHTLMEKKTKRKRLEDSHVLMNSGKLINGKRYFGRALELPEVKEWLKQSQRQNDGGSINTKCIPKDR
NDFYYHGKVTAALTEFEANWTSILKAHYNVPVNEDEEEMSRQTQEIHVPTLADMEHWLVQRRKKKLMDELNL
[illegible]
>Q80ZV0
MAGGRDRGDLAARQLVFLLPEHLKDASKKKKKSSLLFVKLANPHSGEGATYLIDMCLQQLFEIKVFKEKHHSWFINQSVQSGGLLHFATPMDPLFLLLHYLLKAGKEGKYQPLDQVVVDDTFPDCTLLLRFPELEKSLRHVTEEKEVNSKKYYKYSSEKTLKWLEKKVN
QTVVALKANNVNVGARVQSSAYFSGGQVSRDKEEDYVRYAHGLISDYIPKELSDDLSKFLKLPEPPASLTNPPSKKLKLSDEPVEAKEDYTKFNTKDLKTGKKNSKMTAAQKALAKVDKSGMKSIDAFFGAKNKKTGKI
[illegible]
>Q9BRG1
MAMSFEWPWQYRFPPFFTLQPNVDTRQKQLAAWCSLVLSFCRLHKQSSMTVMEAQESPLFNNVKLQRKLPVESIQIVLEELRKKGNLEWLDKSKSSFLIMWRRPEEWGKLIYQWVSRSGQNNSVFTLYELTNGEDTEDEEFHGLDEATLLRALQALQQEHKAEII
TVSDGRGVKFF
[illegible]
>P47142
MSALPPVYSFPPLYTRQPNSLTRRQQISTWIDIISQYCKTKKIWYMSVDGTVINDNELDSGSTDNDDSKKISKNLFNNEDIQRSVSQVFIDEIWSQMTKEGKCLPIDQSGRRSSNTTTTRYFILWKSLDSWASLILQWFEDSGKLNQVITLYELSEGDETVNWEFHRM
PESLLYYCLKPLCDRNRATMLKDENDKVIAIKVV
[illegible]
>Q06208
MEHVDSDFAPIRRSKKVVDSDKIVKAISDDLEQKNFTVLRKLNLVPIKKSVSSPKVCKPSPVKERVDHVFYQKFKSMALQELGTNYLSISYVPSLSKFLSKNLRSMKNCIVFFDKVEHIHQYAGIDRAVSETLSLVDINVVIIEMNDYLMKEGIQSSKSKECIESMGQASYS
GQLDFEASEKPSNHTSDLMMMVMRKINNDESIDHIVYFKFEQLDKLSTSTIIEPSKLTEFINVLSVLEKSNNIAFKVLIYSNNVSISSLLSTSLKKKLNTKYTVFEMPILTCAQEQEYLKKMIKFTFDSGSKLLQSYNSLVTCQLNNKESNLAIFFEFLKVFPHPFTYLFNAYTEI
IVQSRTFDELLDKIRNRLTIKNYPHSAYNFKKNQRLPLKLTRKVHDR
[illegible]

[illegible]
>P47910
MASLLLGTPSMACHSVSFSFSHSQSVAGVSLSSPPTLSLSSSVSTSPALPLIYCGRGDRKTAKGKRFNHSFGNARPKNKNKGRGPPKAPIFPKGDPSQKED
[illegible]
>Q06433
MNNNKRSKLSTVPSSRPIRVGFVGLTSGKSWVAKTHFLAIQQLSSQFQIVALYNPTLKSSLQTIEQLQLKHATGFDSLESFAQYKDIDMIVVSVKVPEHYEVVKNILEHSSQNLNLRYLYVEWALAASVQQAEELYSISQQRANLQTIICLQGRKSPYIVRAKELISEGCIG
DINSIEISGNGGWYGYERPMRSPEYLYDIESGVNLISNSFGHTIDVLQYITGSYFQKINAMISNNIPTQFLLDENGKRTKETISKTCPDHLLFQGILENGKVPVSCSFKGGTPVKKLTKNLVIDIHGTKGDLKIEGDAGFVEISNLVLYFYGIKNGNGSSNGTDNNGAAAIK
DKEKVTKSPSPSTGTSEEEQTMEVFHLRNYNSVVGNILRIYESIADYHFLGKPESKSSRGPDDLFASTKFDKQGFRFEGFPTFKDAIILHRLIDAVFRSDKEEKTLDVSKIMI
[illegible]
>P14736
MNEDLPKEYFELIRKALNEKEAEKAPLSRRRRVRRKNQPLPDAKKKFKTGLNELPRESVVTVNLDSSDDGVVTVPTDDSVEEIQSSEEDYDSEEFEDVTDGNEVAGVEDISVEIKPSSKRNSDARRTSRNVCSNEERKRRKYFHMLYLVCLMVHGFIRNEWINSKRLSR
KLSNLVPEKVFELLHPQKDEELPLRSTRKLLDGLKKCMELWQKHWKITKKYDNVGLYMRTWKEIEMSANNKRKFKTLKRSDFLRAVSKGHGDPDISVQGFVAMLRACNVNARLIMSCQPPDFTNMKIDTSLNGNNAYKDMVKYPIFWCEVWDKFSKKWITVDP
VNLKTIEQVRLHSKLAPKGVACCERNMLRYVIAYDRKYGCRDVTRRYAQWMNSKVRKRRITKDDFGEKWFRKVITALHHRKRTKIDDYEDQYFFQRDESEGIPDSVQDLKNHPYYVLEQDIKQTQIVKPGCKECGYLKVHGKVGKVLKVYAKRDIADLKSARQWY
MNGRILKTGSRCKKVIKRTVGRPKGEAEEEDERLYSFEDTELYIPPLASASGEITKNTFGNIEVFAPTMIPGNCCLVENPVAIKAARFLGVEFAPAVTSFKFERGSTVKPVLSGIVVAKWLREAIETAIDGIEFIQEDDNRKEHLLGALESWNTLLLKLRIRSKLNSTYGKIAE
EEPNVTKEQNIADNHDNTETFMGGGFLPGIANHEARPYSEPSEPEDSLDYVSVDKAESATDDDVGEDYSDFMKELEMSEESD
[illegible]
>P20239
MARWQRKASVSSPCGRSIYRFLSLLFTLVTSVNSVSLPQSENPAFPGTLICDKDEVRIEFSSRFDMEKWNPSVVDTLGSEILNCTYALDLERFVLKFPYETCTIKVVGGYQVNIRVGDTTTDVRYKDDMYHFFCPAIQAETHEISEIVVCRRDLISFSFPQLFSRLADENQN
VSEMGWIVKIGNGTRAHILPLKDAIVQGFNLLIDSQKVTLHVPANATGIVHYVQESSYLYTVQLELLFSTTGQKIVFSSHAICAPDLSVACNATHMTLTIPEFPGKLESVDFGQWSIPEDQWHANGIDKEATNGLRLNFRKSLLKTKPSEKCPFYQFYLSSLKLTFYFQGN
MLSTVIDPECHCESPVSIDELCAQDGFMDFEVYSHQTKPALNLDTLLVGNSSCQPIFKVQSVGLARFHIPLNGCGTRQKFEGDKVIYENEIHALWENPPSNIVFRNSEFRMTVRCYYIRDSMLLNAHVKGHPSPEAFVKPGPLVLVLQTYPDQSYQRPYRKDEYPLVR
YLRQPIYMEVKVLSRNDPNIKLVLDDCWATSSEDPASAPQWQIVMDGCEYELDNYRTTFHPAGSSAAHSGHYQRFDVKTFAFVSEARGLSSLIYFHCSALICNQVSLDSPLCSVTCPASLRSKREANKEDTMTVSLPGPILLLSDVSSSKGVDPSSSEITKDIIAKDIASK
TLGAVAALVGSAVILGFICYLYKKRTIRFNH
[illegible]
>P0C1H4
MPEPAKSAPAPKKGSKKAVTKTQKKGDKKRRKSRKESYSIYVYKVLKQVHPDTGISSKAMGIMNSFVNDIFERIAGEASRLAHYNKRSTITSREIQTAVRLLLPGELAKHAVSEGTKAVTKYTSSK
[illegible]
>O53492
MADRVLRGSRLGAVSYETDRNHDLAPRQIARYRTDNGEEFEVPFADDAEIPGTWLCRNGMEGTLIEGDLPEPKKVKPPRTHWDMLLERRSIEELEELLKERLELIRSRRRG
[illegible]
>P12081
MAERAALEELVKLQGERVRGLKQQKASAELIEEEVAKLLKLKAQLGPDESKQKFVLKTPKGTRDYSPRQMAVREKVFDVIIRCFKRHGAEVIDTPVFELKETLMGKYGEDSKLIYDLKDQGGELLSLRYDLTVPFARYLAMNKLTNIKRYHIAKVYRRDNPAMTRGRYR
EFYQCDFDIAGNFDPMIPDAECLKIMCEILSSLQIGDFLVKVNDRRILDGMFAICGVSDSKFRTICSSVDKLDKVSWEEVKNEMVGEKGLAPEVADRIGDYVQQHGGVSLVEQLLQDPKLSQNKQALEGLGDLKLLFEYLTLFGIDDKISFDLSLARGLDYYTGVIYEAV
LLQTPAQAGEEPLGVGSVAAGGRYDGLVGMFDPKGRKVPCVGLSIGVERIFSIVEQRLEALEEKIRTTETQVLVASAQKKLLEERLKLVSELWDAGIKAELLYKKNPKLLNQLQYCEEAGIPLVAIIGEQELKDGVIKLRSVTSREEVDVRREDLVEEIKRRTGQPLCIC
[illegible]
>Q5VZK9
MTEESSDVPRELIESIKDVIGRKIKISVKKKVKLEVKGDKVENKVLVLTSCRAFLVTARIPTKLELTFSYLEIHGVVCSKSAQMIVETEKCSISMKMASPEDVSEVLAHIGTCLRKIFPGLSPVRIMKKVSMEPSERLASLQALWDSQTVAEQGPCGGFSQMYACVCDWLG
FSYREEVQWDVDTIYLTQDTRELNLQDFSHLDHRDLIPIIAALEYNQWFTKLSSKDLKLSTDVCEQILRVVSRSNRLEELVLENAGLRTDFAQKLASALAHNPNSGLHTINLAGNPLEDRGVSSLSIQFAKLPKGLKHLNLSKTSLSPKGVNSLSQSLSANPLTASTLVHLD
LSGNVLRGDDLSHMYNFLAQPNAIVHLDLSNTECSLDMVCGALLRGCLQYLAVLNLSRTVFSHRKGKEVPPSFKQFFSSSLALMHINLSGTKLSPEPLKALLLGLACNHNLKGVSLDLSNCELRSGGAQVLEGCIAEIHNITSLDISDNGLESDLSTLIVWLSKNRSIQHL
ALGKNFNNMKSKNLTPVLDNLVQMIQDEESPLQSLSLADSKLKTEVTIIINALGSNTSLTKVDISGNGMGDMGAKMLAKALQINTKLRTVIWDKNNITAQGFQDIAVAMEKNYTLRFMPIPMYDASQALKTNPEKTEDALQKIENYLLRNHETRKYLQEQAYRLQQ
GIVTSTTQQMIDRICVKVQDHLNSLRNCGGDAIQEDLKSAERLMRDAKNSKTLLPNLYHVGGASWAGASGLLSSPIQETLESMAGEVTRVVDEQLKALLESMVDAAENLCPNVMKKAHIRQDLIHASTEKISIPRTFVKNVLLEQSGIDILNKISEVKLTVASFLSDRIV
DEILDALSHCHHKLADHFSRRGKTLPQQESLEIELAEEKPVKRSIITVEELTEIERLEDLDTCMMTPKSKRKSIHSRMLRPVSRAFEMEFDLDKALEEVPIHIEDPPFPSLRQEKRSSGFISELPSEEGKKLEHFTKLRPKRNKKQPQTQAAVCAANIVSQDGEQNGLMGRV
DEGVDEFFTKKVTKMDSKKWSTRGSESHELNEGGDEKKKRDSRKSSGFLNLIKSRSKSERPPTILMTEEPSSPKGAVRSPPVDCPRKDTKAAEHNGNSERIEEIKTPDSFEESQGEEIGKVERSDSKSSPQAGRRYGVQVMGSGLLAEMKAKQEKRAACQKKLGND
AVSQDSSSPALSGVERSDGGGAVPKLHPGLPENRFGLGTPEKNTKAEPKAEAGSRSRSSSSTPTSPKPLLQSPKPSLAARPVIPQKPRTASRPDDIPDSPSSPKVALLPPVLKKVPSDKERDGQSSPQPSPRTFSQEVSRRSWGQQAQEYQEQKQRSSSKDGHQGSKS
NDSGEEAEKEFIFV
[illegible]
>Q99741
MPQTRSQAQATISFPKRKLSRALNKAKNSSDAKLEPTNVQTVTCSPRVKALPLSPRKRLGDDNLCNTPHLPPCSPPKQGKKENGPPHSHTLKGRRLVFDNQLTIKSPSKRELAKVHQNKILSSVRKSQEITTNSEQRCPLKKESACVRLFKQEGTCYQQAKLVLNTAVP
DRLPAREREMDVIRNFLREHICGKKAGSLYLSGAPGTGKTACLSRILQDLKKELKGFKTIMLNCMSLRTAQAVFPAIAQEICQEEVSRPAGKDMMRKLEKHMTAEKGPMIVLVLDEMDQLDSKGQDVLYTLFEWPWLSNSHLVLIGIANTLDLTDRILPRLQAREKC
KPQLLNFPPYTRNQIVTILQDRLNQVSRDQVLDNAAVQFCARKVSAVSGDVRKALDVCRRAIEIVESDVKSQTILKPLSECKSPSEPLIPKRVGLIHISQVISEVDGNRMTLSQEGAQDSFPLQQKILVCSLMLLIRQLKIKEVTLGKLYEAYSKVCRKQQVAAVDQSECLS
LSGLLEARGILGLKRNKETRLTKVFFKIEEKEIEHALKDKALIGNILATGLP
[illegible]

00000000000000000000000000000000000000000000000000000000000000000000000000000000000000000000000000000000000000000000000000000000000000000000000000000000000000000000000000000000
000000000000000000000000000000000
XXXXXXXXXXXXXXXXXXXXXXXXXXXXXXXXXXXXXXXXXXXXXXXXXXXXXXXXXXXXXXXXXXXXXX0000000000000000000000000000000000000000000000000000000000000000000000000000000000000000000000000000000000000000000000
00000000000000000000000000000000000000000000000000000000000000000000000000000000000000000000000000000000000000000000000000000000000000000000000000000000000000000000000000000000
00000000000000000000000000000000000000000000000000000000000000000000000000000000000000000000000000000000000000000000000000000000000000000000000000000000000000000000000000000000
000000000000000000000000000000000
XXXXXXXXXXXXXXXXXXXXXXXXXXXXXXXXXXXXXXXXXXXXXXXXXXXXXXXXXXXXXXXXXXXXXX0000000000000000000000000000000000000000000000000000000000000000000000000000000000000000000000000000000000000000000000
00000000000000000000000000000000000000000000000000000000000000000000000000000000000000000000000000000000000000000000000000000000000000000000000000000000000000000000000000000000
00000000000000000000000000000000000000000000000000000000000000000000000000000000000000000000000000000000000000000000000000000000000000000000000000000000000000000000000000000000
000000000000000000000000000000000
>Q07666
MQRRDDPAARMSRSSGRSGSMDPSGAHPSVRQTPSRQPPLPHRSRGGGGGSRGGARASPATQPPPLLPPSATGPDATVGGPAPTPLLPPSATASVKMEPENKYLPELMAEKDSLDPSFTHAMQLLTAEIEKIQKGDSKKDDEENYLDLFSHKNMKLKERVLIPVK
QYPKFNFVGKILGPQGNTIKRLQEETGAKISVLGKGSMRDKAKEEELRKGGDPKYAHLNMDLHVFIEVFGPPCEAYALMAHAMEEVKKFLVPDMMDDICQEQFLELSYLNGVPEPSRGRGVPVRGRGAAPPPPPVPRGRGVGPPRGALVRGTPVRGAITRGATV
TRGVPPPPTVRGAPAPRARTAGIQRIPLPPPPAPETYEEYGYDDTYAEQSYEGYEGYYSQSQGDSEYYDYGHGEVQDSYEAYGQDDWNGTRPSLKAPPARPVKGAYREHPYGRY
00000000000000000000000000000000000000000000000000000000000000000000000000000000000000000000000000000000000000000000000000000000000000000000000000000000000000000000000000000000
00000000000000000000000000000000000000000000000000000000000000000000000000000000000000000000000000000000000000000000000000000000000000000000000000000000000000000000000000000000
00000000000011111111111111111111111111111111111111111111111111111XXXXXXXXXXXXXXXXXXXXXXXX
00000000000000000000000000000000000000000000000000000000000000000000000000000000000000000000000000000000000000000000000000000000000000000000000000000000000000000000000000000000
00000000000000000000000000000000000000000000000000000000000000000000000000000000000000000000000000000000000000000000000000000000000000000000000000000000000000000000000000000000
00000000000000000000000000111111111110000000000000000000000000000000XXXXXXXXXXXXXXXXXXXXXXXX
00000000000000000000000000000000000000000000000000000000000000000000000000000000000000000000000000000000000000000000000000000000000000000000000000000000000000000000000000000000
00000000000000000000000000000000000000000000000000000000000000000000000000000000000000000000000000000000000000000000000000000000000000000000000000000000000000000000000000000000
0000000000000000000000000000000000000000000000000000000000000000000XXXXXXXXXXXXXXXXXXXXXXXX
>P9WKX3
MVIGLSTGSDDDDVEVIGGVDPRLIAVQENDSDESSLTDLVEQPAKVMRIGTMIKQLLEEVRAAPLDEASRNRLRDIHATSIRELEDGLAPELREELDRLTLPFNEDAVPSDAELRIAQAQLVGWLEGLFHGIQTALFAQQMAARAQLQQMRQGALPPGVGKSGQ
HGHGTGQYL
11111111111111111111111111111111111100000000000000000000000000000000000000000000000000000000000000000000000000000000000000000000000000000000000000000000000000011111111111111
00000000000000000000000000000000000000000000000000000000000000000000000000000000000000000000000000000000000000000000000000000000000000000000000000000000000000000000000000
00000000000000000000000000000000000000000000000000000000000000000000000000000000000000000000000000000000000000000000000000000000000000000000000000000000000000000000000000
>Q13029
MNQNTTEPVAATETLAEVPEHVLRGLPEEVRLFPSAVDKTRIGVWATKPILKGKKFGPFVGDKKKRSQVKNNVYMWEVYYPNLGWMCIDATDPEKGNWLRYVNWACSGEEQNLFPLEINRAIYYKTLKPIAPGEELLVWYNGEDNPEIAAAIEEERASARSKRSSP
KSRKGKKKSQENKNKGNKIQDIQLKTSEPDFTSANMRDSAEGPKEDEEKPSASALEQPATLQEVASQEVPPELATPAPAWEPQPEPDERLEAAACEVNDLGEEEEEEEEEDEEEEEDDDDDELEDEGEEEASMPNENSVKEPEIRCDEKPEDLLEEPKTTSEETLEDC
SEVTPAMQIPRTKEEANGDVFETFMFPCQHCERKFTTKQGLERHMHIHISTVNHAFKCKYCGKAFGTQINRRRHERRHEAGLKRKPSQTLQPSEDLADGKASGENVASKDDSSPPSLGPDCLIMNSEKASQDTINSSVVEENGEVKELHPCKYCKKVFGTHTNMRR
HQRRVHERHLIPKGVRRKGGLEEPQPPAEQAQATQNVYVPSTEPEEEGEADDVYIMDISSNISENLNYYIDGKIQTNNNTSNCDVIEMESASADLYGINCLLTPVTVEITQNIKTTQVPVTEDLPKEPLGSTNSEAKKRRTASPPALPKIKAETDSDPMVPSCSLSLPLSI
STTEAVSFHKEKSVYLSSKLKQLLQTQDKLTPAGISATEIAKLGPVCVSAPASMLPVTSSRFKRRTSSPPSSPQHSPALRDFGKPSDGKAAWTDAGLTSKKSKLESHSDSPAWSLSGRDERETVSPPCFDEYKMSKEWTASSAFSSVCNQQPLDLSSGVKQKAEGTGKT
PVQWESVLDLSVHKKHCSDSEGKEFKESHSVQPTCSAVKKRKPTTCMLQKVLLNEYNGIDLPVENPADGTRSPSPCKSLEAQPDPDLGPGSGFPAPTVESTPDVCPSSPALQTPSLSSGQLPPLLIPTDPSSPPPCPPVLTVATPPPPLLPTVPLPAPSSSASPHPCPSPL
SNATAQSPLPILSPTVSPSPSPIPPVEPLMSAASPGPPTLSSSSSSSSSSSFSSSSSSSSPSPPPLSAISSVVSSGDNLEASLPMISFKQEELENEGLKPREEPQSAAEQDVVVQETFNKNFVCNVCESPFLSIKDLTKHLSIHAEEWPFKCEFCVQLFKDKTDLSEHRFLLHG
VGNIFVCSVCKKEFAFLCNLQQHQRDLHPDKVCTHHEFESGTLRPQNFTDPSKAHVEHMQSLPEDPLETSKEEEELNDSSEELYTTIKIMASGIKTKDPDVRLGLNQHYPSFKPPPFQYHHRNPMGIGVTATNFTTHNIPQTFTTAIRCTKCGKGVDNMPELHKHILA
CASASDKKRYTPKKNPVPLKQTVQPKNGVVVLDNSGKNAFRRMGQPKRLNFSVELSKMSSNKLKLNALKKKNQLVQKAILQKNKSAKQKADLKNACESSSHICPYCNREFTYIGSLNKHAAFSCPKKPLSPPKKKVSHSSKKGGHSSPASSDKNSNSNHRRRTADAE
IKMQSMQTPLGKTRARSSGPTQVPLPSSSFRSKQNVKFAASVKSKKPSSSSLRNSSPIRMAKITHVEGKKPKAVAKNHSAQLSSKTSRSLHVRVQKSKAVLQSKSTLASKKRTDRFNIKSRERSGGPVTRSLQLAAAADLSENKREDGSAKQELKDFSYSLRLASRCSPP
AAPYITRQYRKVKAPAAAQFQGPPFFKE
00000000000000000000000000000000000000000000000000000000000000000000000000000000000000000000000000000000000000000000000000000000000000000000000000000000000000000000000000000000
0000000000000000001111111111111111111111111111111111111111111111111111111111111111111111111111111111111111111111111111111111111111111111111111111111111111111111XXXXXXXXXX
XXXXXXXXXXXXXXXXXXXXXXXXXXXXXXXXXXXXXXXXXXXXXXXXXXXXXXXXXXXXXXXXXXXXXXXXXXXXXXXXXXXXXXXXXXXXXXXXXXXXXXXXXXXXXXXXXXXXXXXXXXXXXXXXXXXXXXXXXXXXXXXXXXXXXXXXXXXXXXXXXXXXXXXXXXXX
XXXXXXXXXXXXXXXXXXXXXXXXXXXXXXXXXXXXXXXXXXXXXXXXXXXXXXXXXXXXXXXXXXXXXXXXXXXXXXXXXXXXXXXXXXXXXXXXXXXXXXXXXXXXXXXXXXXXXXXXXXXXXXXXXXXXXXXXXXXXXXXXXXXXXXXXXXXXXXXXXXXXXXXXXXXX
XXXXXXXXXXXXXXXXXXXXXXXXXXXXXXXXXXXXXXXXXXXXXXXXXXXXXXXXXXXXXXXXXXXXXXXXXXXXXXXXXXXXXXXXXXXXXXXXXXXXXXXXXXXXXXXXXXXXXXXXXXXXXXXXXXXXXXXXXXXXXXXXXXXXXXXXXXXXXXXXXXXXXXXXXXXXX
XXXXXXXXXXXXXXXXXXXXXXXXXXXXXXXXXXXXXXXXXXXXXXXXXXXXXXXXXXXXXXXXXXXXXXXXXXXXXXXXXXXXXXXXXXXXXXXXXXXXXXXXXXXXXXXXXXXXXXXXXXXXXXXXXXXXXXXXXXXXXXXXXXXXXXXXXXXXXXXXXXXXXXXXXXXX
XXXXXXXXXXXXXXXXXXXXXXXXXXXXXXXXXXXXXXXXXXXXXXXXXXXXXXXXXXXXXXXXXXXXXXXXXXXXXXXXXXXXXXXXXXXXXXXXXXXXXXXXXXXXXXXXXXXXXXXXXXXXXXXXXXXXXXXXXXXXXXXXXXXXXXXXXXXXXXXXXXXXXXXXXXXX
XXXXXXXXXXXXXXXXXXXXXXXXXXXXXXXXXXXXXXXXXXXXXXXXXXXXXXXXXXXXXXXXXXXXXXXXXXXXXXXXXXXXXXXXXXXXXXXXXXXXXXXXXXXXXXXXXXXXXXXXXXXXXXXXXXXXXXXXXXXXXXXXXXXXXXXXXXXXXXXXXXXXXXXXXXXX
XXXXXXXXXXXXXXXXXXXXXXXXXXXXXXXXXXXXXXXXXXXXXXXXXXXXXXXXXXXXXXXXXXXXXXXXXXXXXXXXXXXXXXXXXXXXXXXXXXXXXXXXXXXXXXXXXXXXXXXXXXXXXXXXXXXXXXXXXXXXXXXXXXXXXXXXXXXXXXXXXXXXXXXXXXXX
XXXXXXXXXXXXXXXXXXXXXXXXXXXXXXXXXXXXXXXXXXXXXXXXXXXXXXXXXXXXXXXXXXXXXXXXXXXXXXXXXXXXXXXXXXXXXXXXXXXXXXXXXXXXXXXXXXXXXXXXXXXXXXXXXXXXXXXXXXXXXXXXXXXXXXXXXXXXXXXXXXXX
00000000000000000000000000000000000000000000000000000000000000000000000000000000000000000000000000000000000000000000000000000000000000000000000000000000000000000000000000000000
0000000000000000001111111111111111111111111111111111111111111111111111111111111111111111111111111111111111111111111111111111111111111111111111111111111111111111XXXXXXXXXX
XXXXXXXXXXXXXXXXXXXXXXXXXXXXXXXXXXXXXXXXXXXXXXXXXXXXXXXXXXXXXXXXXXXXXXXXXXXXXXXXXXXXXXXXXXXXXXXXXXXXXXXXXXXXXXXXXXXXXXXXXXXXXXXXXXXXXXXXXXXXXXXXXXXXXXXXXXXXXXXXXXXXXXXXXXXXX
XXXXXXXXXXXXXXXXXXXXXXXXXXXXXXXXXXXXXXXXXXXXXXXXXXXXXXXXXXXXXXXXXXXXXXXXXXXXXXXXXXXXXXXXXXXXXXXXXXXXXXXXXXXXXXXXXXXXXXXXXXXXXXXXXXXXXXXXXXXXXXXXXXXXXXXXXXXXXXXXXXXXXXXXXXXX
XXXXXXXXXXXXXXXXXXXXXXXXXXXXXXXXXXXXXXXXXXXXXXXXXXXXXXXXXXXXXXXXXXXXXXXXXXXXXXXXXXXXXXXXXXXXXXXXXXXXXXXXXXXXXXXXXXXXXXXXXXXXXXXXXXXXXXXXXXXXXXXXXXXXXXXXXXXXXXXXXXXXXXXXXXXX
XXXXXXXXXXXXXXXXXXXXXXXXXXXXXXXXXXXXXXXXXXXXXXXXXXXXXXXXXXXXXXXXXXXXXXXXXXXXXXXXXXXXXXXXXXXXXXXXXXXXXXXXXXXXXXXXXXXXXXXXXXXXXXXXXXXXXXXXXXXXXXXXXXXXXXXXXXXXXXXXXXXXXXXXXXXX
XXXXXXXXXXXXXXXXXXXXXXXXXXXXXXXXXXXXXXXXXXXXXXXXXXXXXXXXXXXXXXXXXXXXXXXXXXXXXXXXXXXXXXXXXXXXXXXXXXXXXXXXXXXXXXXXXXXXXXXXXXXXXXXXXXXXXXXXXXXXXXXXXXXXXXXXXXXXXXXXXXXXXXXXXXXX
XXXXXXXXXXXXXXXXXXXXXXXXXXXXXXXXXXXXXXXXXXXXXXXXXXXXXXXXXXXXXXXXXXXXXXXXXXXXXXXXXXXXXXXXXXXXXXXXXXXXXXXXXXXXXXXXXXXXXXXXXXXXXXXXXXXXXXXXXXXXXXXXXXXXXXXXXXXXXXXXXXXXXXXXXXXX
XXXXXXXXXXXXXXXXXXXXXXXXXXXXXXXXXXXXXXXXXXXXXXXXXXXXXXXXXXXXXXXXXXXXXXXXXXXXXXXXXXXXXXXXXXXXXXXXXXXXXXXXXXXXXXXXXXXXXXXXXXXXXXXXXXXXXXXXXXXXXXXXXXXXXXXXXXXXXXXXXXXXXXXXXXXX
XXXXXXXXXXXXXXXXXXXXXXXXXXXXXXXXXXXXXXXXXXXXXXXXXXXXXXXXXXXXXXXXXXXXXXXXXXXXXXXXXXXXXXXXXXXXXXXXXXXXXXXXXXXXXXXXXXXXXXXXXXXXXXXXXXXXXXXXXXXXXXXXXXXXXXXXXXXXXXXXXXXX
00000000000000000000000000000000000000000000000000000000000000000000000000000000000000000000000000000000000000000000000000000000000000000000000000000000000000000000000000000000
0000000000000000000000000000000000000000000000000000000000000000000000000000000000000000000000000000000000000000000000000000000000000000000000000000000000000000000000XXXXXXXXXX
XXXXXXXXXXXXXXXXXXXXXXXXXXXXXXXXXXXXXXXXXXXXXXXXXXXXXXXXXXXXXXXXXXXXXXXXXXXXXXXXXXXXXXXXXXXXXXXXXXXXXXXXXXXXXXXXXXXXXXXXXXXXXXXXXXXXXXXXXXXXXXXXXXXXXXXXXXXXXXXXXXXXXXXXXXXX
XXXXXXXXXXXXXXXXXXXXXXXXXXXXXXXXXXXXXXXXXXXXXXXXXXXXXXXXXXXXXXXXXXXXXXXXXXXXXXXXXXXXXXXXXXXXXXXXXXXXXXXXXXXXXXXXXXXXXXXXXXXXXXXXXXXXXXXXXXXXXXXXXXXXXXXXXXXXXXXXXXXXXXXXXXXX
XXXXXXXXXXXXXXXXXXXXXXXXXXXXXXXXXXXXXXXXXXXXXXXXXXXXXXXXXXXXXXXXXXXXXXXXXXXXXXXXXXXXXXXXXXXXXXXXXXXXXXXXXXXXXXXXXXXXXXXXXXXXXXXXXXXXXXXXXXXXXXXXXXXXXXXXXXXXXXXXXXXXXXXXXXXX
XXXXXXXXXXXXXXXXXXXXXXXXXXXXXXXXXXXXXXXXXXXXXXXXXXXXXXXXXXXXXXXXXXXXXXXXXXXXXXXXXXXXXXXXXXXXXXXXXXXXXXXXXXXXXXXXXXXXXXXXXXXXXXXXXXXXXXXXXXXXXXXXXXXXXXXXXXXXXXXXXXXXXXXXXXXX
XXXXXXXXXXXXXXXXXXXXXXXXXXXXXXXXXXXXXXXXXXXXXXXXXXXXXXXXXXXXXXXXXXXXXXXXXXXXXXXXXXXXXXXXXXXXXXXXXXXXXXXXXXXXXXXXXXXXXXXXXXXXXXXXXXXXXXXXXXXXXXXXXXXXXXXXXXXXXXXXXXXXXXXXXXXX
XXXXXXXXXXXXXXXXXXXXXXXXXXXXXXXXXXXXXXXXXXXXXXXXXXXXXXXXXXXXXXXXXXXXXXXXXXXXXXXXXXXXXXXXXXXXXXXXXXXXXXXXXXXXXXXXXXXXXXXXXXXXXXXXXXXXXXXXXXXXXXXXXXXXXXXXXXXXXXXXXXXXXXXXXXXX
XXXXXXXXXXXXXXXXXXXXXXXXXXXXXXXXXXXXXXXXXXXXXXXXXXXXXXXXXXXXXXXXXXXXXXXXXXXXXXXXXXXXXXXXXXXXXXXXXXXXXXXXXXXXXXXXXXXXXXXXXXXXXXXXXXXXXXXXXXXXXXXXXXXXXXXXXXXXXXXXXXXXXXXXXXXX
XXXXXXXXXXXXXXXXXXXXXXXXXXXXXXXXXXXXXXXXXXXXXXXXXXXXXXXXXXXXXXXXXXXXXXXXXXXXXXXXXXXXXXXXXXXXXXXXXXXXXXXXXXXXXXXXXXXXXXXXXXXXXXXXXXXXXXXXXXXXXXXXXXXXXXXXXXXXXXXXXXXX
>P03714
MADFDNLFDAAIARADETIRGYMGTSATITSGEQSGAVIRGVFDDPENISYAGQGVRVEGSSPSLFVRTDEVRQLRRGDTLTIGEENFWVDRVSPDDGGSCHLWLGRGVPPAVNRRR
11111111111111111111111XXXXXXXXXXXXXXXXXXXXX11111111111111111XXXXXXXXXXXXXXXXXXXXXXXXXXXXXXXXXXXXXXXXXXXXXXXXXXXXXXXXX
00000000000000000000000XXXXXXXXXXXXXXXXXXXXX00000000000000000XXXXXXXXXXXXXXXXXXXXXXXXXXXXXXXXXXXXXXXXXXXXXXXXXXXXXXXXX
00000000000000000000000XXXXXXXXXXXXXXXXXXXXX00000000000000000XXXXXXXXXXXXXXXXXXXXXXXXXXXXXXXXXXXXXXXXXXXXXXXXXXXXXXXXX
>P58753
MASSTSLPAPGSRPKKPLGKMADWFRQTLLKKPKKRPNSPESTSSDASQPTSQDSPLPPSLSSVTSPSLPPTHASDSGSSRWSKDYDVCVCHSEEDLVAAQDLVSYLEGSTASLRCFLQLRDATPGGAIVSELCQALSSSHCRVLLITPGFLQDPWCKYQMLQALTEA
PGAEGCTIPLLSGLSRAAYPPELRFMYYVDGRGPDGGFRQVKEAVMRYLQTLS
XXXXXXXXXXXXX11111111111111111111000000000000000000000000000000000000000000000000000000000000000000000000000000000000000000000000000000000000000000000000000000000000000000
000000000000000000000000000000000000000000000
XXXXXXXXXXXXX000000000000000000000000000000000000000000000000000000000000000000000000000000000000000000000000000000000000000000000000000000000000000000000000000000000000000
000000000000000000000000000000000000000000000
XXXXXXXXXXXXX000000000000000000000000000000000000000000000000000000000000000000000000000000000000000000000000000000000000000000000000000000000000000000000000000000000000000
000000000000000000000000000000000000000000000
>O54928
MDKVGKMWNNLKYRCQNLFSHEGGSRNENVEMNPNRCPSVKEKSISLGEAAPQQESSPLRENVALQLGLSPSKTFSRRNQNCAAEIPQVVEISIEKDSDSGATPGTRLARRDSYSRHAPWGGKKKHSCSTKTQSSLDTEKKFGRTRSGLQRRERRYGVSSMQDM
DSVSSRAVGSRSLRQRLQDTVGLCFPMRTYSKQSKPLFSNKRKIHLSELMLEKCPFPAGSDLAQKWHLIKQHTAPVSPHSTFFDTFDPSLVSTEDEEDRLRERRRLSIEEGVDPPPNAQIHTFEATAQVNPLYKLGPKLAPGMTEISGDGSAIPQTNCDSEEDSTTLCLQ
SRRQKQRQVSGDSHAHVSRQGAWKVHTQIDYIHCLVPDLLQITGNPCYWGVMDRYEAEALLEGKPEGTFLLRDSAQEDYLFSVSFRRYNRSLHARIEQWNHNFSFDAHDPCVFHSSTVTGLLEHYKDPSSCMFFEPLLTISLNRTFPFSLQYICRAVICRCTTYDGID
GLPLPSMLQDFLKEYHYKQKVRVRWLEREPVKAK
XXXXXXXXXXXXXXXXXXXXXXXXXXXXXXXXXXXXXXXXXXXXXXXXXXXXXXXXXXXXXXXXXXXXXXXXXXXXXXXXXXXXXXXXXXXXXXXXXXXXXXXXXXXXXXXXXXXXXXXXXXXXXXXXXXXXXXXXXXXXXXXXXXXXXXXXXXXXXXXXXXXXXXXXXXXX
XX11111111111111111111111111111111111111111111111111111111111111111111111100000000000000000000000000000000000000000000000000000000000000000000000000000000000000000000000000000000
00000000000000000000000000000000000000000000000000000000000000000000000000000000000000000000000000000000000000000000000000000000000000000000000000000000000000000000000000000000
000000000000
XXXXXXXXXXXXXXXXXXXXXXXXXXXXXXXXXXXXXXXXXXXXXXXXXXXXXXXXXXXXXXXXXXXXXXXXXXXXXXXXXXXXXXXXXXXXXXXXXXXXXXXXXXXXXXXXXXXXXXXXXXXXXXXXXXXXXXXXXXXXXXXXXXXXXXXXXXXXXXXXXXXXXXXXXXXX
XX11111111111111111111111111111111111111111111111111111111111111111111111100000000000000000000000000000000000000000000000000000000000000000000000000000000000000000000000000000000
00000000000000000000000000000000000000000000000000000000000000000000000000000000000000000000000000000000000000000000000000000000000000000000000000000000000000000000000000000000
000000000000
XXXXXXXXXXXXXXXXXXXXXXXXXXXXXXXXXXXXXXXXXXXXXXXXXXXXXXXXXXXXXXXXXXXXXXXXXXXXXXXXXXXXXXXXXXXXXXXXXXXXXXXXXXXXXXXXXXXXXXXXXXXXXXXXXXXXXXXXXXXXXXXXXXXXXXXXXXXXXXXXXXXXXXXXXXXX
XX00000000000000000000000000000000000000000000000000000000000000000000000000000000000000000000000000000000000000000000000000000000000000000000000000000000000000000000000000000000
00000000000000000000000000000000000000000000000000000000000000000000000000000000000000000000000000000000000000000000000000000000000000000000000000000000000000000000000000000000
000000000000
>Q3LS93
MEVKVTLIVAIVAALAISAHAQRDFNERRGKENDTERGQGGFGGRPGGMQMGGRRQDGGPMGEMRFDGPESGAPQMDGRRQNGGPMGGRRFDGPVFGGSRPDGAGGRPFFGQGGRRGDGEEETDAAQQIGDGLGGRGQFDGPGRRHHGRKPFGDR
PFGRRNHTEGHQGHNETGDHPHDGHGRGHHGHRQGPPQDRPEEQPFGQRNESSDEDGRPHPRHHGRHHQHHHRNHTEGHQGHNETGDHPHRHHNKTGDGDQDRPMFEMRPFRFNPFGRKPFGDRPFGRRNGTEEGSARRDGQRRPYGNRGRWGE
NESEEEEHPTTESVTTSSPP
XXXXXXXXXXXXXXXXXXXX11111111111111111111111111111111111111111111111111111111111111111111111111111111111111111111111111111111111111111111111111111111111111111111111111111111
11111111111111111111111111111111111111111111111111111111111111111111111111111111111111111111111111111111111111111111111111111111111111111111111111111111111111111
XXXXXXXXXXXXXXXXXXXX00000000000000000000000000000000000000000000000000000000000000000000000000000000000000000000000000000000000000000000000000000000000000000000000000000000
00000000000000000000000000000000000000000000000000000000000000000000000000000000000000000000000000000000000000000000000000000000000000000000000000000000000000000
XXXXXXXXXXXXXXXXXXXX00000000000000000000000000000000000000000000000000000000000000000000000000000000000000000000000000000000000000000000000000000000000000000000000000000000
00000000000000000000000000000000000000000000000000000000000000000000000000000000000000000000000000000000000000000000000000000000000000000000000000000000000000000
>Q87P35
MVNINTSHMQSLSQLTSHLATQNQADDKEIRAHNGKDIFVKEGAPKNKSVSARISHQNKAKDIVVNLLTKQGIPKDVAKQMLQNVLNGENKLTLGNLKNLERLSSGPSSHWPAAQTDTSSSKTSKASSSVALINEHKDNLQKLGDFACTDLNTFGQKGSQFYETK
GAPRLKALYANQARIAQDQRATMSASAVQAAAQRGNAPQLSTLQNIAKYVQNAKAGCCTTFAFAAAAEMIQGMSGTPENQPKVEVVAFKKGHSGTHLYVLVGRQEGSDIKDPSTWNKDVKIVDPWAASAFGATMFGDAQRPPVSNMFPPTEVIFDSHKLG
1111111111111111111111111111111111111111111111111111111111111111111111111111111111111111111111111111111111111111111111111111XXXXXXXXXXXXXXXXXXXXXXXXXXXXXXXXXXXXXXXXXXXXXXXXXXXX
XXXXXXXXXXXXXXXXXXXXXXXXXXXXXXXXXXXXXXXXXXXXXXXXXXXXXXXXXXXXXXXXXXXXXXXXXXXXXXXXXXXXXXXXXXXXXXXXXXXXXXXXXXXXXXXXXXXXXXXXXXXXXXXXXXXXXXXXXXXXXXXXXXXXXXXXXXXXXXX
0000000000000000000000000000000000000000000000000000000000000000000000000000000000000000000000000000000000000000000000000000XXXXXXXXXXXXXXXXXXXXXXXXXXXXXXXXXXXXXXXXXXXXXXXXXXXX
XXXXXXXXXXXXXXXXXXXXXXXXXXXXXXXXXXXXXXXXXXXXXXXXXXXXXXXXXXXXXXXXXXXXXXXXXXXXXXXXXXXXXXXXXXXXXXXXXXXXXXXXXXXXXXXXXXXXXXXXXXXXXXXXXXXXXXXXXXXXXXXXXXXXXXXXXXXXXXX
0000000000000000000000000000000000000000000000000000000000000000000000000000000000000000000000000000000000000000000000000000XXXXXXXXXXXXXXXXXXXXXXXXXXXXXXXXXXXXXXXXXXXXXXXXXXXX
XXXXXXXXXXXXXXXXXXXXXXXXXXXXXXXXXXXXXXXXXXXXXXXXXXXXXXXXXXXXXXXXXXXXXXXXXXXXXXXXXXXXXXXXXXXXXXXXXXXXXXXXXXXXXXXXXXXXXXXXXXXXXXXXXXXXXXXXXXXXXXXXXXXXXXXXXXXXXXXX
>D2JX42
MAEEKHHHHLFHHHKEEKPAEEVIYSETAYSGGGDYASGYTETVVAESASDEYEKYKKEEKHHKHKEHLGEMGAVAAGAFALYEKHEAKKDPDHAHKHKIEEEIAAAVAVGSGGYAFHEHHEKKDAKKEAEEASGKKHHHHLF
11111111111111111111111111111111111111111111111111111111111111111111111111111111111111111111111111111111111111111111111111111111111111111111111
00000000000000000000000000000000000000000000000000000000000000000000000000000000000000000000000000000000000000000000000000000000000000000000000
00000000000000000000000000000000000000000000000000000000000000000000000000000000000000000000000000000000000000000000000000000000000000000000000
>A0ZWU1

MNSLQKGSYKVIPGSSFSKNSNGLLSIKYNFIPESVDPSRRGVLEKAQEAYRLRLPSTFDDDRPHIFEGSCQRARNVDCVLIFNAKTKTFTLEHIDEIARLNALRNPKVSKTVPSNAITQSDNSQISESKSTSQSAVTTNSTRRKEKELEASKDGKIKPSSSNTRYPAISSKGPI
TTDTNDEPDMEVMELDDFAKELELGFDQEFNSIDDPSTVSQTASKPISLRGLSSQERDYASSAQAEGISSASEDED
XXXXXXXXXXXXXXXXXXXXXXXXXXXXXXXXXXXXXXXXXXXXXXXXXXXXXXXXXXXXXXXXXXXXXXXXXXXXXXXXXXXXXXXXXXXXXXXXXXXXXXXXXXXXXXXXXXXXXXXXXXXXXXXXXXXXXXXXXXXXXXXXXXXXXXXXXXXX1111111111111111
111111111111111111111111111111111111111111111111111111111111111111111111111111
XXXXXXXXXXXXXXXXXXXXXXXXXXXXXXXXXXXXXXXXXXXXXXXXXXXXXXXXXXXXXXXXXXXXXXXXXXXXXXXXXXXXXXXXXXXXXXXXXXXXXXXXXXXXXXXXXXXXXXXXXXXXXXXXXXXXXXXXXXXXXXXXXXXXXXXXXXXXX0000000000000000
000000000000000000000000000000000000000000000000000000000000000000000000000000
XXXXXXXXXXXXXXXXXXXXXXXXXXXXXXXXXXXXXXXXXXXXXXXXXXXXXXXXXXXXXXXXXXXXXXXXXXXXXXXXXXXXXXXXXXXXXXXXXXXXXXXXXXXXXXXXXXXXXXXXXXXXXXXXXXXXXXXXXXXXXXXXXXXXXXXXXXXXX0000000000000000
000000000000000000000000000000000000000000000000000000000000000000000000000000
>Q9BUH6
MDPLSPPLCTLPPGPEPPRFVCYCEGEESGEGDRGGFNLYVTDAAELWSTCFTPDSLAALKARFGLSAAEDITPRFRAACEQQAVALTLQEDRASLTLSGGPSALAFDLSKVPGPEAAPRLRALTLGLAKRVWSLERRLAAAEETAVSPRKSPRPAGPQLFLPDPDPQR
GGPGPGVRRRCPGESLINPGFKSKKPAGGVDFDET
0000000000000000000000000000000000000000000000000000000000000000000000000000000000000000000000000000000000000000000000000000000000000000000000001111111111111111111111111111111111
1111111111111111111111111111
0000000000000000000000000000000000000000000000000000000000000000000000000000000000000000000000000000000000000000000000000000000000000000000000001111111111111111111111111111111111
1111111111111111111111111111
00000000000000000000000000000000000000000000000000000000000000000000000000000000000000000000000000000000000000000000000000000000000000000000000000000000000000000000000000000000
0000000000000000000000000000
>P49005
MFSEQAAQRAHTLLSPPSANNATFARVPVATYTNSSQPFRLGERSFSRQYAHIYATRLIQMRPFLENRAQQHWGSGVGVKKLCELQPEEKCCVVGTLFKAMPLQPSILREVSEEHNLLPQPPRSKYIHPDDELVLEDELQRIKLKGTIDVSKLVTGTVLAVFGSVRDD
GKFLVEDYCFADLAPQKPAPPLDTDRFVLLVSGLGLGGGGGESLLGTQLLVDVVTGQLGDEGEQCSAAHVSRVILAGNLLSHSTQSRDSINKAKYLTKKTQAASVEAVKMLDEILLQLSASVPVDVMPGEFDPTNYTLPQQPLHPCMFPLATAYSTLQLVTNPYQATI
DGVRFLGTSGQNVSDIFRYSSMEDHLEILEWTLRVRHISPTAPDTLGCYPFYKTDPFIFPECPHVYFCGNTPSFGSKIIRGPEDQTVLLVTVPDFSATQTACLVNLRSLACQPISFSGFGAEDDDLGGLGLGP
00000000000000000000000000000000000000000111111111111000000000000000000000000000000000000000111111111111111111110000000000000000000000000000000000000000000000000000000000000000
00000000000000000000000000000000000000000000000000000000000000000000000000000000000000000000000000000000000000000000000000000000000000000000000000000000000000000000000000000000
000000000000000000000000000000000000000000000000000000000000000000000000000000000000000000000000000000000000000000000
00000000000000000000000000000000000000000000000000000000000000000000000000000000000000000000000000000000000000000000000000000000000000000000000000000000000000000000000000000000
00000000000000000000000000000000000000000000000000000000000000000000000000000000000000000000000000000000000000000000000000000000000000000000000000000000000000000000000000000000
000000000000000000000000000000000000000000000000000000000000000000000000000000000000000000000000000000000000000000000
00000000000000000000000000000000000000000000000000000000000000000000000000000000000000000000000000000000000000000000000000000000000000000000000000000000000000000000000000000000
00000000000000000000000000000000000000000000000000000000000000000000000000000000000000000000000000000000000000000000000000000000000000000000000000000000000000000000000000000000
000000000000000000000000000000000000000000000000000000000000000000000000000000000000000000000000000000000000000000000
>Q9I832
MKLTLLCLCFISITAAWPVSKSKQHAISASSEEKYDPRSHHTHRYHQDHVDSQSQEHLQQTQNDLASLQQTHYSSEENADVPEQPDFPDVPSKSQETVDDDDDDDNDSNDTDESDEVFTDFPTEAPVAPFNRGDNAGRGDSVAYGFRAKAHVVKASKIRKAARK
LIEDDATTEDGDSQPAGLWWPKESREQNSRELPQHQSVENDSRPKFDSREVDGGDSKASAGVDSRESQGSVPAVDASNQTLESAEDAEDRHSIENNEVTR
XXXXXXXXXXXXXXXXXXXXXXXXXXXXXXXXXXXXXXXXXXXXXXXXXXXXXXXXXXXXXXXXXXXXXXXXXXXX1111111111111111111111111111111XXXXXXXXXXXXXXXXXXXXXXXXXXXXXXXXXXXXXXXXXXXXXXXXXXXXXXXXXXXXXXXXXX
XXXXXXXXXXXXXXXXXXXXXXXXXXXXXXXXXXXXXXXXXXXXXXXXXXXXXXXXXXXXXXXXXXXXXXXXXXXXXXXXXXXXXXXXXXX
XXXXXXXXXXXXXXXXXXXXXXXXXXXXXXXXXXXXXXXXXXXXXXXXXXXXXXXXXXXXXXXXXXXXXXXXXXXX1111111111111111111111111111111XXXXXXXXXXXXXXXXXXXXXXXXXXXXXXXXXXXXXXXXXXXXXXXXXXXXXXXXXXXXXXXXXX
XXXXXXXXXXXXXXXXXXXXXXXXXXXXXXXXXXXXXXXXXXXXXXXXXXXXXXXXXXXXXXXXXXXXXXXXXXXXXXXXXXXXXXXXXXX
XXXXXXXXXXXXXXXXXXXXXXXXXXXXXXXXXXXXXXXXXXXXXXXXXXXXXXXXXXXXXXXXXXXXXXXXXXXX0000000000000000000000000000000XXXXXXXXXXXXXXXXXXXXXXXXXXXXXXXXXXXXXXXXXXXXXXXXXXXXXXXXXXXXXXXXXX
XXXXXXXXXXXXXXXXXXXXXXXXXXXXXXXXXXXXXXXXXXXXXXXXXXXXXXXXXXXXXXXXXXXXXXXXXXXXXXXXXXXXXXXXXXX
>P25644
MSFFGLENSGNARDGPLDFEESYKGYGEHELEENDYLNDETFGDNVQVGTDFDFGNPHSSGSSGNAIGGNGVGATARSYVAATAEGISGPRTDGTAAAGPLDLKPMESLWSTAPPPAMAPSPQSTMAPAPAPQQMAPLQPILSMQDLERQQRQMQQQFM
NFHAMGHPQGLPQGPPQQQFPMQPASGQPGPSQFAPPPPPPGVNVNMNQMPMGPVQVPVQASPSPIGMSNTPSPGPVVGATKMPLQSGRRSKRDLSPEEQRRLQIRHAKVEKILKYSGLMTPRDKDFITRYQLSQIVTEDPYNEDFYFQVYKIIQRGGITSE
SNKGLIARAYLEHSGHRLGGRYKRTDIALQRMQSQVEKAVTVAKERPSKLKDQQAAAGNSSQDNKQANTVLGKISSTLNSKNPRRQLQIPRQQPSDPDALKDVTDSLTNVDLASSGSSSTGSSAAAVASKQRRRSSYAFNNGNGATNLNKSGGKKFILELIETVYEEI
LDLEANLRNGQQTDSTAMWEALHIDDSSYDVNPFISMLSFDKGIKIMPRIFNFLDKQQKLKILQKIFNELSHLQIIILSSYKTTPKPTLTQLKKVDLFQMIILKIIVSFLSNNSNFIEIMGLLLQLIRNNNVSFLTTSKIGLNLITILISRAALIKQDSSRSNILSSPEISTWNEIYDK
LFTSLESKIQLIFPPREYNDHIMRLQNDKFMDEAYIWQFLASLALSGKLNHQRIIIDEVRDEIFATINEAETLQKKEKELSVLPQRSQELDTELKSIIYNKEKLYQDLNLFLNVMGLVYRDGEISELK
XXXX111111111111111111110000000000000000000000000000001111111111111111111111111XXXXXXXXXXXXXXXXXXXXXXXXXXXXXXXXXXXXXXXXXXXXXXXXXXXXXXXXXXXXXXXXXXXXXXXXXXXXXXXXXXXXXXXXXXXXXXXXXXXXX
XXXXXXXXXXXXXXXXXXXXXXXXXXXXXXXXXXXXXXXXXXXXXXXXXXXXXXXXXXXXXXXXXXXXXXXXXXXXXXXXXXXXXXXXXXXXXXXXXXXXXXXXXXXXXXXXXXXXXXXXXXXXXXXXXXXXXXXXXXXXXXXXXXXXXXXXXXXXXXXXXXXXXXXX
XXXXXXXXXXXXXXXXXXXXXXXXXXXXXXXXXXXXXXXXXXXXXXXXXXXXXXXXXXXXXXXXXXXXXXXXXXX1111111111111111111111111111111111111111111100000000000000000000000000000000000000000000000000000000
00000000000000000000000000000000000000000000000000000000000000000000000000000000000000000000000000000000000000000000000000000000000000000000000000000000000000000000000000000000
000000000000000000000000000000000000000000000000000000000000000000000000000000000000000000000000000
XXXX000000000000000000000000000000000000000000000000000000000000000000000000000XXXXXXXXXXXXXXXXXXXXXXXXXXXXXXXXXXXXXXXXXXXXXXXXXXXXXXXXXXXXXXXXXXXXXXXXXXXXXXXXXXXXXXXXXXXXXXXXXXXXX
XXXXXXXXXXXXXXXXXXXXXXXXXXXXXXXXXXXXXXXXXXXXXXXXXXXXXXXXXXXXXXXXXXXXXXXXXXXXXXXXXXXXXXXXXXXXXXXXXXXXXXXXXXXXXXXXXXXXXXXXXXXXXXXXXXXXXXXXXXXXXXXXXXXXXXXXXXXXXXXXXXXXXXXXXX
XXXXXXXXXXXXXXXXXXXXXXXXXXXXXXXXXXXXXXXXXXXXXXXXXXXXXXXXXXXXXXXXXXXXXXXXXXX0000000000000000000000000000000000000000000000000000000000000000000000000000000000000000000000000000
00000000000000000000000000000000000000000000000000000000000000000000000000000000000000000000000000000000000000000000000000000000000000000000000000000000000000000000000000000000
000000000000000000000000000000000000000000000000000000000000000000000000000000000000000000000000000
XXXX000000000000000000000000000000000000000000000000000000000000000000000000000XXXXXXXXXXXXXXXXXXXXXXXXXXXXXXXXXXXXXXXXXXXXXXXXXXXXXXXXXXXXXXXXXXXXXXXXXXXXXXXXXXXXXXXXXXXXXXXXXXXXX
XXXXXXXXXXXXXXXXXXXXXXXXXXXXXXXXXXXXXXXXXXXXXXXXXXXXXXXXXXXXXXXXXXXXXXXXXXXXXXXXXXXXXXXXXXXXXXXXXXXXXXXXXXXXXXXXXXXXXXXXXXXXXXXXXXXXXXXXXXXXXXXXXXXXXXXXXXXXXXXXXXXXXXXXXXXX
XXXXXXXXXXXXXXXXXXXXXXXXXXXXXXXXXXXXXXXXXXXXXXXXXXXXXXXXXXXXXXXXXXXXXXXXXXX0000000000000000000000000000000000000000000000000000000000000000000000000000000000000000000000000000
00000000000000000000000000000000000000000000000000000000000000000000000000000000000000000000000000000000000000000000000000000000000000000000000000000000000000000000000000000000
000000000000000000000000000000000000000000000000000000000000000000000000000000000000000000000000000
>Q7Q3R9
MASKLFVLAFLCLALVVVVQSAPQYARGDVPTYDEEDFDEESLKPHSSSSSDDGEEEFDPSLLEEHADAPTARDPGRNPEFLRNSNTDEQASAPAASSSESDE
XXXXXXXXXXXXXXXXXXXXX1111111111111111111111111111111111111111111111111111111111111111111111111111111111
XXXXXXXXXXXXXXXXXXXXX00000000000000000000000000000000000011111111111111111111111111111111110000000000000
XXXXXXXXXXXXXXXXXXXXX0000000000000000000000000000000000000000000000000000000000000000000000000000000000
>P85800
SDQGDVAEPKMHKTAPPFDFEAIPEEYLDDES
11111111111111111111111111111111
00000000001111111111111111111111
00000000000000000000000000000000
>Q6F3E8
MLKNIVLLLAAGLSGPAVLAKPKWSPLFEEATNALEARSGGHQTAVPKVSKQGLGGDFEEIPSDEIIKRALEDRSGGHQTAVPKISKQGLGGDFEEIPSDEIIERALEARSDGHQTAVPKMSKQGLGGDIEEIPSDEIIERALEARSGGHQTAVPKMSKQGLGGDSEEIP
SNEIIERALEARSGGHETAVPKMSKQGLGGDFEEIPSDEIIERALQARG
00000000000000000000000000000000000000000000000000000000000000000000000000000000000000000000000000000000000000000000000000000000000000000000111111111XXXXXXXXXXXXXXXXXXXXXXXXXX
XXXXXXXXXXXXXXXXXXXXXXXXXXXXXXXXXXXXXXXXXXX
00000000000000000000000000000000000000000000000000000000000000000000000000000000000000000000000000000000000000000000000000000000000000000000111111111XXXXXXXXXXXXXXXXXXXXXXXXXX
XXXXXXXXXXXXXXXXXXXXXXXXXXXXXXXXXXXXXXXXXXX
00000000000000000000000000000000000000000000000000000000000000000000000000000000000000000000000000000000000000000000000000000000000000000000000000000XXXXXXXXXXXXXXXXXXXXXXXXXX
XXXXXXXXXXXXXXXXXXXXXXXXXXXXXXXXXXXXXXXXXXX
>O43715
MNSVGEACTDMKREYDQCFNRWFAEKFLKGDSSGDPCTDLFKRYQQCVQKAIKEKEIPIEGLEFMGHGKEKPENSS
0000000000000000000000000000000000000000000000000000011111111111111111111111
0000000000000000000000000000000000000000000000000000000000000000000000000000
0000000000000000000000000000000000000000000000000000000000000000000000000000
>P38968
MVKLAEFSRTATFAWSHDKIPLLVSGTVSGTVDANFSTDSSLELWSLLAADSEKPIASLQVDSKFNDLDWSHNNKIIAGALDNGSLELYSTNEANNAINSMARFSNHSSSVKTVKFNAKQDNVLASGGNNGEIFIWDMNKCTESPSNYTPLTPGQSMSSVDEVISLA
WNQSLAHVFASAGSSNFASIWDLKAKKEVIHLSYTSPNSGIKQQLSVVEWHPKNSTRVATATGSDNDPSILIWDLRNANTPLQTLNQGHQKGILSLDWCHQDEHLLLSSGRDNTVLLWNPESAEQLSQFPARGNWCFKTKFAPEAPDLFACASFDNKIEVQTLQN
LTNTLDEQETETKQQESETDFWNNVSREESKEKPTVFHLQAPTWYGEPSPAAHWAFGGKLVQITPDGKGVSITNPKISGLESNTTLSEALKTKDFKPLINQRLVKVIDDVNEEDWNLLEKLSMDGTEEFLKEALAFDNDESDAQDDANNEKEDDGEEFFQQIETNF
QPEGDFSLSGNIEQTISKNLVSGNIKSAVKNSLENDLLMEAMVIALDSNNERLKESVKNAYFAKYGSKSSLSRILYSISKREVDDLVENLDVSQWKFISKAIQNLYPNDIAQRNEMLIKLGDRLKENGHRQDSLTLYLAAGSLDKVASIWLSEFPDLEDKLKKDNKTIYEA
HSECLTEFIERFTVFSNFINGSSTINNEQLIAKFLEFINLTTSTGNFELATEFLNSLPSDNEEVKTEKARVLIASGKSLPAQNPATATTSKAKYTNAKTNKNVPVLPTPGMPSTTSIPSMQAPFYGMTPGASANALPPKPYVPATTTSAPVHTEGKYAPPSQPSMASPFVN
KTNSSTRLNSFAPPPNPYATATVPATNVSTTSIPQNTFAPIQPGMPIMGDYNAQSSSIPSQPPINAVSGQTPHLNRKANDGWNDLPLKVKEKPSRAKAVSVAPPNILSTPTPLNGIPANAASTMPPPPLSRAPSSVSMVSPPPLHKNSRVPSLVATSESPRASISNPYA
PPQSSQQFPIGTISTANQTSNTAQVASSNPYAPPPQQRVATPLSGGVPPAPLPKASNPYAPTATTQPNGSSYPPTGPYTNNHTMTSPPPVFNKPPTGPPPISMKKRSNKLASIEQNPSQGATYPPTLSSSASPLQPSQPPTLASQVNTSAENVSHEIPADQQPIVDFL
KEELARVTPLTPKEYSKQLKDCDKRLKILFYHLEKQDLLTQPTIDCLHDLVALMKEKKYKEAMVIHANIATNHAQEGGNWLTGVKRLIGIAEATLN
00000000000000000000000000000000000000000000000000000000000000000000000000000000000000000000000000000000000000000000000000000000000000000000000000000000000000000000000000000000
00000000000000000000000000000000000000000000000000000000000000000000000000000000000000000000000000000000000000000000000000000000000000000000000000000000000000000000000000000000
00000000000000000000000000000000000000000000000000000000000000000000000000000000000000000000000000000000000000000000000000000000000000000000000000000000000000000000000000000000
00000000000000000000000000000000000000000000000000000000000000000000000000000000000000000000000000000000000000000000000000000000000000000000000000000000000000000000000000000000
00000000000000000000000000000000000000000000000000000000000000000000000000000000000000000000000000000000000000000000000000000000000000000000000000000000000000000000000000000000
00000000000000000000000000000000000000000000000000000000000000011111111111111111111111111111111111111111111111111111111111111111111111111111111111111111111111111111111111111111
11111111111111111111111111111111111111111111111111111111111111111111111111111111111111111111111111111111111111111111111111111111111111111111111111111111111111111111111111111111
11111111111111111111111111111111111111111110000000000000000000000000000000000000000000000000000000000000000000000000000000000000000000000000000000000000000000000000000000000000
00000000000000000000000000000000000000000
00000000000000000000000000000000000000000000000000000000000000000000000000000000000000000000000000000000000000000000000000000000000000000000000000000000000000000000000000000000
00000000000000000000000000000000000000000000000000000000000000000000000000000000000000000000000000000000000000000000000000000000000000000000000000000000000000000000000000000000
00000000000000000000000000000000000000000000000000000000000000000000000000000000000000000000000000000000000000000000000000000000000000000000000000000000000000000000000000000000
00000000000000000000000000000000000000000000000000000000000000000000000000000000000000000000000000000000000000000000000000000000000000000000000000000000000000000000000000000000
00000000000000000000000000000000000000000000000000000000000000000000000000000000000000000000000000000000000000000000000000000000000000000000000000000000000000000000000000000000
00000000000000000000000000000000000000000000000000000000000000000000000000000000000000000000000000000000000000000000000000000000000000000000000000000000000000000000000000000000
00000000000000000000000000000000000000000000000000000000000000000000000000000000000000000000000000000000000000000000000000000000000000000000000000000000000000000000000000000000
00000000000000000000000000000000000000000000000000000000000000000000000000000000000000000000000000000000000000000000000000000000000000000000000000000000000000000000000000000000
00000000000000000000000000000000000000000
00000000000000000000000000000000000000000000000000000000000000000000000000000000000000000000000000000000000000000000000000000000000000000000000000000000000000000000000000000000
00000000000000000000000000000000000000000000000000000000000000000000000000000000000000000000000000000000000000000000000000000000000000000000000000000000000000000000000000000000
00000000000000000000000000000000000000000000000000000000000000000000000000000000000000000000000000000000000000000000000000000000000000000000000000000000000000000000000000000000
00000000000000000000000000000000000000000000000000000000000000000000000000000000000000000000000000000000000000000000000000000000000000000000000000000000000000000000000000000000
00000000000000000000000000000000000000000000000000000000000000000000000000000000000000000000000000000000000000000000000000000000000000000000000000000000000000000000000000000000
00000000000000000000000000000000000000000000000000000000000000000000000000000000000000000000000000000000000000000000000000000000000000000000000000000000000000000000000000000000
00000000000000000000000000000000000000000000000000000000000000000000000000000000000000000000000000000000000000000000000000000000000000000000000000000000000000000000000000000000
00000000000000000000000000000000000000000000000000000000000000000000000000000000000000000000000000000000000000000000000000000000000000000000000000000000000000000000000000000000
00000000000000000000000000000000000000000
>Q92540
MSLQSAQYLRQAEVLKADMTDSKLGPAEVWTSRQALQDLYQKMLVTDLEYALDKKVEQDLWNHAFKNQITTLQGQAKNRANPNRSEVQANLSLFLEAASGFYTQLLQELCTVFNVDLPCRVKSSQLGIISNKQTHTSAIVKPQSSSCSYICQHCLVHLGDIARYRN
QTSQAESYYRHAAQLVPSNGQPYNQLAILASSKGDHLTTIFYYCRSIAVKFPFPAASTNLQKALSKALESRDEVKTKWGVSDFIKAFIKFHGHVYLSKSLEKLSPLREKLEEQFKRLLFQKAFNSQQLVHVTVINLFQLHHLRDFSNETEQHTYSQDEQLCWTQLLALFM
SFLGILCKCPLQNESQEESYNAYPLPAVKVSMDWLRLRPRVFQEAVVDERQYIWPWLISLLNSFHPHEEDLSSISATPLPEEFELQGFLALRPSFRNLDFSKGHQGITGDKEGQQRRIRQQRLISIGKWIADNQPRLIQCENEVGKLLFITEIPELILEDPSEAKENLILQET
SVIESLAADGSPGLKSVLSTSRNLSNNCDTGEKPVVTFKENIKTREVNRDQGRSFPPKEVRRDYSKGITVTKNDGKKDNNKRKTETKKCTLEKLQETGKQNVAVQVKSQTELRKTPVSEARKTPVTQTPTQASNSQFIPIHHPGAFPPLPSRPGFPPPTYVIPPPVAFS
MGSGYTFPAGVSVPGTFLQPTAHSPAGNQVQAGKQSHIPYSQQRPSGPGPMNQGPQQSQPPSQQPLTSLPAQPTAQSTSQLQVQALTQQQQSPTKAVPALGKSPPHHSGFQQYQQADASKQLWNPPQVQGPLGKIMPVKQPYYLQTQDPIKLFEPSLQPP
VMQQQPLEKKMKPFPMEPYNHNPSEVKVPEFYWDSSYSMADNRSVMAQQANIDRRGKRSPGVFRPEQDPVPRMPFEKSLLEKPSELMSHSSSFLSLTGFSLNQERYPNNSMFNEVYGKNLTSSSKAELSPSMAPQETSLYSLFEGTPWSPSLPASSDHSTPASQ
SPHSSNPSSLPSSPPTHNHNSVPFSNFGPIGTPDNRDRRTADRWKTDKPAMGGFGIDYLSATSSSESSWHQASTPSGTWTGHGPSMEDSSAVLMESLKSIWSSSMMHPGPSALEQLLMQQKQKQQRGQGTMNPPH
00000000000000000000000000000000000000000000000000000000000000000000000000000000000000000000000000000000011111111111111111111111111111111111111111111111000000000000000000000000
00000000000000000000000000000000000000000000000000000000000000000000000000000000000000000000000000000000000000000000000000000000000000000000000000000000000000000000000000000000
00000000000000000000000000000000000000000000000000000000000000000000000000000000000000000000000000000000000000000000000000000000000000000000000000000000000000000000000000000000

00000000000000000000000000000000000000000000000000000000000000000000000000000000000000000000000000000000000000000000000000000000000000000000000000000000000000000000000000000000
00000000000000000000000000000000000000000000000000000000000000000000000000000000000000000000000000000000000000000000000000000000000000000000000000000000000000000000000000000000
00000000000000000000000000000000000000000000000000000000000000000000000000000000000000000000000000000000000000000000000000000000000000000000000000000000000000000000000000000000
000000000000000000000000000000000000000000000000000000000000000000000000000000000
00000000000000000000000000000000000000000000000000000000000000000000000000000000000000000000000000000000000000000000000000000000000000000000000000000000000000000000000000000000
00000000000000000000000000000000000000000000000000000000000000000000000000000000000000000000000000000000000000000000000000000000000000000000000000000000000000000000000000000000
00000000000000000000000000000000000000000000000000000000000000000000000000000000000000000000000000000000000000000000000000000000000000000000000000000000000000000000000000000000
00000000000000000000000000000000000000000000000000000000000000000000000000000000000000000000000000000000000000000000000000000000000000000000000000000000000000000000000000000000
00000000000000000000000000000000000000000000000000000000000000000000000000000000000000000000000000000000000000000000000000000000000000000000000000000000000000000000000000000000
00000000000000000000000000000000000000000000000000000000000000000000000000000000000000000000000000000000000000000000000000000000000000000000000000000000000000000000000000000000
000000000000000000000000000000000000000000000000000000000000000000000000000000000
00000000000000000000000000000000000000000000000000000000000000000000000000000000000000000000000000000000000000000000000000000000000000000000000000000000000000000000000000000000
00000000000000000000000000000000000000000000000000000000000000000000000000000000000000000000000000000000000000000000000000000000000000000000000000000000000000000000000000000000
00000000000000000000000000000000000000000000000000000000000000000000000000000000000000000000000000000000000000000000000000000000000000000000000000000000000000000000000000000000
00000000000000000000000000000000000000000000000000000000000000000000000000000000000000000000000000000000000000000000000000000000000000000000000000000000000000000000000000000000
00000000000000000000000000000000000000000000000000000000000000000000000000000000000000000000000000000000000000000000000000000000000000000000000000000000000000000000000000000000
00000000000000000000000000000000000000000000000000000000000000000000000000000000000000000000000000000000000000000000000000000000000000000000000000000000000000000000000000000000
000000000000000000000000000000000000000000000000000000000000000000000000000000000
>Q9M605
MAYQQDPCANPTRQTGKTGGQTDQYGNPVHQTEALGAYGAGTGTGMHGGEHQQQPHQQPGVLHRSGSSSSEDDGQGGRRKKGMKEKIKERIPGMGRKDEQKQTSATSTPGQGQQQKGMMEKIKEKLPGAH
XXXXXXXXXXXXXXXXXXXXXXXXXXXXXXXXXXXXXXXXXXXXXXXXXXXXXXXXXXXXXXXXXXXXXXXXXXXXXXX111111111111111111111111111111111111111111111111111
XXXXXXXXXXXXXXXXXXXXXXXXXXXXXXXXXXXXXXXXXXXXXXXXXXXXXXXXXXXXXXXXXXXXXXXXXXXXXXX000000000000000000000000000000000000000000000000000
XXXXXXXXXXXXXXXXXXXXXXXXXXXXXXXXXXXXXXXXXXXXXXXXXXXXXXXXXXXXXXXXXXXXXXXXXXXXXXX000000000000000000000000000000000000000000000000000
>Q96270
MASHQEQSYKAGETRGKAQEKTGEAMGTMGDKTQAAKDKTQETAQSAQQKAHETAQSAKDKTSQAAQTTQERAQESKDKTGSYMSETGEAIKNKAHDAAEYTKETAEAGKEKTSGILGQTGEQVKQMAMGATDAVKHTLGLRTDEGNKEHVSSAPSTTTT
TTTRETQRK
1111111111111111111111111111111111111111111111111111111111111111111111111111111111111111111111111111111111111111111111111111111111111111111111111111111111111111111111111
0000000000000000000000000000000000000000000000000000000000000000000000000000000000000000000000000000000000000000000000000000000000000000000000000000000000000000000000000
0000000000000000000000000000000000000000000000000000000000000000000000000000000000000000000000000000000000000000000000000000000000000000000000000000000000000000000000000
>Q5NJL5
MAAMFTTRNAIFRFSKSFPNVPSLSLPKPSRVFVASASHQSDWRNAADGKRNSSMDWAYNSTSKARQDADEIADRERKTLNGDVDSEDVKQYVRDAKERTKEAANRAAENADSAGVKSRDYAYDAKEKTKDAANRAAENVESAGEKAKDYAYDAKERTKDAA
NRAAENAESVGEKARDYAYDAKERTKEAAQNAGETAKDYAYGAKERTKEAAESAGGTARDYAYDATDKTKEAVGTVADKTKEGAKKTAEMTKEGAEKTAETTGEVAGAATEALKSAGEMAKRTAQGAWETAKDATQKIKETVVGKDDDDNDRGGGVGAVVD
EYDVELKRKGYGESKGYDMSKGYGENKGYDQNRGY
11111111111111111111111111111111111111111111111111111111111111111111111111111111111111111111111111111111111111111111111111111111111111111111111111111111111111111111111111111111
11111111111111111111111111111111111111111111111111111111111111111111111111111111111111111111111111111111111111111111111111111111111111111111111111111111111111111111111111111111
111111
00000000000000000000000000000000000000000000000000000000000000000000000000000000000000000000000000000000000000000000000000000000000000000000000000000000000000000000000000000000
00000000000000000000000000000000000000000000000000000000000000000000000000000000000000000000000000000000000000000000000000000000000000000000000000000000000000000000000000000000
000000
00000000000000000000000000000000000000000000000000000000000000000000000000000000000000000000000000000000000000000000000000000000000000000000000000000000000000000000000000000000
00000000000000000000000000000000000000000000000000000000000000000000000000000000000000000000000000000000000000000000000000000000000000000000000000000000000000000000000000000000
000000
>P01042
MKLITILFLCSRLLLSLTQESQSEEIDCNDKDLFKAVDAALKKYNSQNQSNNQFVLYRITEATKTVGSDTFYSFKYEIKEGDCPVQSGKTWQDCEYKDAAKAATGECTATVGKRSSTKFSVATQTCQITPAEGPVVTAQYDCLGCVHPISTQSPDLEPILRHGIQYFNNN
TQHSSLFMLNEVKRAQRQVVAGLNFRITYSIVQTNCSKENFLFLTPDCKSLWNGDTGECTDNAYIDIQLRIASFSQNCDIYPGKDFVQPPTKICVGCPRDIPTNSPELEETLTHTITKLNAENNATFYFKIDNVKKARVQVVAGKKYFIDFVARETTCSKESNEELTESCET
KKLGQSLDCNAEVYVVPWEKKIYPTVNCQPLGMISLMKRPPGFSPFRSSRIGEIKEETTVSPPHTSMAPAQDEERDSGKEQGHTRRHDWGHEKQRKHNLGHGHKHERDQGHGHQRGHGLGHGHEQQHGLGHGHKFKLDDDLEHQGGHVLDHGHKHKHGH
GHGKHKNKGKKNGKHNGWKTEHLASSSEDSTTPSAQTQEKTEGPTPIPSLAKPGVTVTFSDFQDSDLIATMMPPISPAPIQSDDDWIPDIQIDPNGLSFNPISDFPDTTSPKCPGRPWKSVSEINPTTQMKESYYFDLTDGLS
00000000000000000000000000000000000000000000000000000000000000000000000000000000000000000000000000000000000000000000000000000000000000000000000000000000000000000000000000000000
00000000000000000000000000000000000000000000000000000000000000000000000000000000000000000000000000000000000000000000000000000000000000000000000000000000000000000000000000000000
00000000000000000000000000000000000000000000000000000000000000000000000000000000000111111111111111111111111111111111111111111111111111111111111111111111111111111111111111111111
111XXXXXXXXXXXXXXXXXXXXXXXXXXXXXXXXXXXXXXXXXXXXXXXXXXXXXXXXXXXXXXXXXXXXXXXXXXXXXXXXXXXXXXXXXXXXXXXXXXXXXXXXXXXXXXXXX
00000000000000000000000000000000000000000000000000000000000000000000000000000000000000000000000000000000000000000000000000000000000000000000000000000000000000000000000000000000
00000000000000000000000000000000000000000000000000000000000000000000000000000000000000000000000000000000000000000000000000000000000000000000000000000000000000000000000000000000
00000000000000000000000000000000000000000000000000000000000000000000000000000000000000000000000000000000000000000000000000000000000000000000000000000000000000000000000000000000
000XXXXXXXXXXXXXXXXXXXXXXXXXXXXXXXXXXXXXXXXXXXXXXXXXXXXXXXXXXXXXXXXXXXXXXXXXXXXXXXXXXXXXXXXXXXXXXXXXXXXXXXXXXXXXXXXX
00000000000000000000000000000000000000000000000000000000000000000000000000000000000000000000000000000000000000000000000000000000000000000000000000000000000000000000000000000000
00000000000000000000000000000000000000000000000000000000000000000000000000000000000000000000000000000000000000000000000000000000000000000000000000000000000000000000000000000000
00000000000000000000000000000000000000000000000000000000000000000000000000000000000000000000000000000000000000000000000000000000000000000000000000000000000000000000000000000000
000XXXXXXXXXXXXXXXXXXXXXXXXXXXXXXXXXXXXXXXXXXXXXXXXXXXXXXXXXXXXXXXXXXXXXXXXXXXXXXXXXXXXXXXXXXXXXXXXXXXXXXXXXXXXXXXXX
>P47047
MDSTDLFDVFEETPVELPTDSNGEKNADTNVGDTPDHTQDKKHGLEEEKEEHEENNSENKKIKSNKSKTEDKNKKVVVPVLADSFEQEASREVDASKGLTNSETLQVEQDGKVRLSHQVRHQVALPPNYDYTPIAEHKRVNEARTYPFTLDPFQDTAISCIDRGESV
LVSAHTSAGKTVVAEYAIAQSLKNKQRVIYTSPIKALSNQKYRELLAEFGDVGLMTGDITINPDAGCLVMTTEILRSMLYRGSEVMREVAWVIFDEVHYMRDKERGVVWEETIILLPDKVRYVFLSATIPNAMEFAEWICKIHSQPCHIVYTNFRPTPLQHYLFPAHGD
GIYLVVDEKSTFREENFQKAMASISNQIGDDPNSTDSRGKKGQTYKGGSAKGDAKGDIYKIVKMIWKKKYNPVIVFSFSKRDCEELALKMSKLDFNSDDEKEALTKIFNNAIALLPETDRELPQIKHILPLLRRGIGIHHSGLLPILKEVIEILFQEGFLKVLFATETFSIGLN
MPAKTVVFTSVRKWDGQQFRWVSGGEYIQMSGRAGRRGLDDRGIVIMMIDEKMEPQVAKGMVKGQADRLDSAFHLGYNMILNLMRVEGISPEFMLEHSFFQFQNVISVPVMEKKLAELKKDFDGIEVEDEENVKEYHEIEQAIKGYREDVRQVVTHPANALS
FLQPGRLVEISVNGKDNYGWGAVVDFAKRINKRNPSAVYTDHESYIVNVVVNTMYIDSPVNLLKPFNPTLPEGIRPAEEGEKSICAVIPITLDSIKSIGNLRLYMPKDIRASGQKETVGKSLREVNRRFPDGIPVLDPVKNMKIEDEDFLKLMKKIDVLNTKLSSNPLTNS
MRLEELYGKYSRKHDLHEDMKQLKRKISESQAVIQLDDLRRRKRVLRRLGFCTPNDIIELKGRVACEISSGDELLLTELIFNGNFNELKPEQAAALLSCFAFQERCKEAPRLKPELAEPLKAMREIAAKIAKIMKDSKIEVVEKDYVESFRHELMEVVYEWCRGATFTQICK
MTDVYEGSLIRMFKRLEELVKELVDVANTIGNSSLKEKMEAVLKLIHRDIVSAGSLYL
00000000000000000000000000000000000000000000000000000000000000000000000000000000000000000000000000000000000000000000000000000000000000000000000000000000000000000000000000000000
00000000000000000000000000000000000000000000000000000000000000000000000000000000000000000000000000000000000000000000000000000000000000000000000000000000000000000000000000000000
00000000111111111111111111111111111111100000000000000000000000000000000000000000000000000000000000000000000000000000000000000000000000000000000000000000000000000000000000000000
00000000000000000000000000000000000000000000000000000000000000000000000000000000000000000000000000000000000000000000000000000000000000000000000000000000000000000000111111
11111111100000000000000000000000000000000000000000000000000000000000000000000000000000000000000000000000000000000000000000000000000000000000000000000000000000000000000000000000
00000000000000000000000000000000000000000000000000000000000000111111111111111111100000000000000000000000000000000000000000000000000000000000000000000000000000000000000000000000000
00000000000000000
00000000000000000000000000000000000000000000000000000000000000000000000000000000000000000000000000000000000000000000000000000000000000000000000000000000000000000000000000000000
00000000000000000000000000000000000000000000000000000000000000000000000000000000000000000000000000000000000000000000000000000000000000000000000000000000000000000000000000000000
00000000000000000000000000000000000000000000000000000000000000000000000000000000000000000000000000000000000000000000000000000000000000000000000000000000000000000000000000000000
00000000000000000000000000000000000000000000000000000000000000000000000000000000000000000000000000000000000000000000000000000000000000000000000000000000000000000000000000000000
00000000000000000000000000000000000000000000000000000000000000000000000000000000000000000000000000000000000000000000000000000000000000000000000000000000000000000000000000000000
00000000000000000000000000000000000000000000000000000000000000000000000000000000000000000000000000000000000000000000000000000000000000000000000000000000000000000000000000000000
00000000000000000
00000000000000000000000000000000000000000000000000000000000000000000000000000000000000000000000000000000000000000000000000000000000000000000000000000000000000000000000000000000
00000000000000000000000000000000000000000000000000000000000000000000000000000000000000000000000000000000000000000000000000000000000000000000000000000000000000000000000000000000
00000000111111111111111111111111111111100000000000000000000000000000000000000000000000000000000000000000000000000000000000000000000000000000000000000000000000000000000000000000
00000000000000000000000000000000000000000000000000000000000000000000000000000000000000000000000000000000000000000000000000000000000000000000000000000000000000000000111111
11111111100000000000000000000000000000000000000000000000000000000000000000000000000000000000000000000000000000000000000000000000000000000000000000000000000000000000000000000000
00000000000000000000000000000000000000000000000000000000000000111111111111111111100000000000000000000000000000000000000000000000000000000000000000000000000000000000000000000000000
00000000000000000
>P51587
MPIGSKERPTFFEIFKTRCNKADLGPISLNWFEELSSEAPPYNSEPAEESEHKNNNYEPNLFKTPQRKPSYNQLASTPIIFKEQGLTLPLYQSPVKELDKFKLDLGRNVPNSRHKSLRTVKTKMDQADDVSCPLLNSCLSESPVVLQCTHVTPQRDKSVVCGSLFHTPKFV
KGRQTPKHISESLGAEVDPDMSWSSSLATPPTLSSTVLIVRNEEASETVFPHDTTANVKSYFSNHDESLKKNDRFIASVTDSENTNQREAASHGFGKTSGNSFKVNSCKDHIGKSMPNVLEDEVYETVVDTSEEDSFSLCFSKCRTKNLQKVRTSKTRKKIFHEANADE
CEKSKNQVKEKYSFVSEVEPNDTDPLDSNVANQKPFESGSDKISKEVVPSLACEWSQLTLSGLNGAQMEKIPLLHISSCDQNISEKDLLDTENKRKKDFLTSENSLPRISSLPKSEKPLNEETVVNKRDEEQHLESHTDCILAVKQAISGTSPVASSFQGIKKSIFRIRESPKE
TFNASFSGHMTDPNFKKETEASESGLEIHTVCSQKEDSLCPNLIDNGSWPATTTQNSVALKNAGLISTLKKKTNKFIYAIHDETSYKGKKIPKDQKSELINCSAQFEANAFEAPLTFANADSGLLHSSVKRSCSQNDSEEPTLSLTSSFGTILRKCSRNETCSNNTVISQDL
DYKEAKCNKEKLQLFITPEADSLSCLQEGQCENDPKSKKVSDIKEEVLAAACHPVQHSKVEYSDTDFQSQKSLLYDHENASTLILTPTSKDVLSNLVMISRGKESYKMSDKLKGNNYESDVELTKNIPMEKNQDVCALNENYKNVELLPPEKYMRVASPSRKVQFNQN
TNLRVIQKNQEETTSISKITVNPDSEELFSDNENNFVFQVANERNNLALGNTKELHETDLTCVNEPIFKNSTMVLYGDTGDKQATQVSIKKDLVYVLAEENKNSVKQHIKMTLGQDLKSDISLNIDKIPEKNNDYMNKWAGLLGPISNHSFGGSFRTASNKEIKLSEHN
IKKSKMFFKDIEEQYPTSLACVEIVNTLALDNQKKLSKPQSINTVSAHLQSSVVVSDCKNSHITPQMLFSKQDFNSNHNLTPSQKAEITELSTILEESGSQFEFTQFRKPSYILQKSTFEVPENQMTILKTTSEECRDADLHVIMNAPSIGQVDSSKQFEGTVEIKRKFAGLL
KNDCNKSASGYLTDENEVGFRGFYSAHGTKLNVSTEALQKAVKLFSDIENISEETSAEVHPISLSSSKCHDSVVSMFKIENHNDKTVSEKNNKCQLILQNNIEMTTGTFVEEITENYKRNTENEDNKYTAASRNSHNLEFDGSDSSKNDTVCIHKDETDLLFTDQHNICL
KLSGQFMKEGNTQIKEDLSDLTFLEVAKAQEACHGNTSNKEQLTATKTEQNIKDFETSDTFFQTASGKNISVAKESFNKIVNFFDQKPEELHNFSLNSELHSDIRKNKMDILSYEETDIVKHKILKESVPVGTGNQLVTFQGQPERDEKIKEPTLLGFHTASGKKVKIAKE
SLDKVKNLFDEKEQGTSEITSFSHQWAKTLKYREACKDLELACETIEITAAPKCKEMQNSLNNDKNLVSIETVVPPKLLSDNLCRQTENLKTSKSIFLKVKVHENVEKETAKSPATCYTNQSPYSVIENSALAFYTSCSRKTSVSQTSLLEAKKWLREGIFDGQPERINTADY
VGNYLYENNSNSTIAENDKNHLSEKQDTYLSNSSMSNSYSYHSDEVYNDSGYLSKNKLDSGIEPVLKNVEDQKNTSFSKVISNVKDANAYPQTVNEDICVEELVTSSSPCKNKNAAIKLSISNSNNFEVGPPAFRIASGKIVCVSHETIKKVKDIFTDSFSKVIKENNENKS
KICQTKIMAGCYEALDDSEDILHNSLDNDECSTHSHKVFADIQSEEILQHNQNMSGLEKVSKISPCDVSLETSDICKCSIGKLHKSVSSANTCGIFSTASGKSVQVSDASLQNARQVFSEIEDSTKQVFSKVLFKSNEHSDQLTREENTAIRTPEHLISQKGFSYNVVNSSA
FSGFSTASGKQVSILESSLHKVKGVLEEFDLIRTEHSLHYSPTSRQNVSKILPRVDKRNPEHCVNSEMEKTCSKEFKLSNNLNVEGGSSENNHSIKVSPYLSQFQQDKQQLVLGTKVSLVENIHVLGKEQASPKNVKMEIGKTETFSDVPVKTNIEVCSTYSKDSENYFET
EAVEIAKAFMEDDELTDSKLPSHATHSLFTCPENEEMVLSNSRIGKRRGEPLILVGEPSIKRNLLNEFDRIIENQEKSLKASKSTPDGTIKDRRLFMHHVSLEPITCVPFRTTKERQEIQNPNFTAPGQEFLSKSHLYEHLTLEKSSSNLAVSGHPFYQVSATRNEKMRHLIT
TGRPTKVFVPPFKTKSHFHRVEQCVRNINLEENRQKQNIDGHGSDDSKNKINDNEIHQFNKNNSNQAAAVTFTKCEEEPLDLITSLQNARDIQDMRIKKKQRQRVFPQPGSLYLAKTSTLPRISLKAAVGGQVPSACSHKQLYTYGVSKHCIKINSKNAESFQFHTED
YFGKESLWTGKGIQLADGGWLIPSNDGKAGKEEFYRALCDTPGVDPKLISRIWVYNHYRWIIWKLAAMECAFPKEFANRCLSPERVLLQLKYRYDTEIDRSRRSAIKKIMERDDTAAKTLVLCVSDIISLSANISETSSNKTSSADTQKVAIIELTDGWYAVKAQLDPPLL
AVLKNGRLTVGQKIILHGAELVGSPDACTPLEAPESLMLKISANSTRPARWYTKLGFFPDPRPFPLPLSSLFSDGGNVGCVDVIIQRAYPIQWMEKTSSGLYIFRNEREEEKEAAKYVEAQQKRLEALFTKIQEEFEEHEENTTKPYLPSRALTRQQVRALQDGAELYEAV
KNAADPAYLEGYFSEEQLRALNNHRQMLNDKKQAQIQLEIRKAMESAEQKEQGLSRDVTTVWKLRIVSYSKKEKDSVILSIWRPSSDLYSLLTEGKRYRIYHLATSKSKSKSERANIQLAATKKTQYQQLPVSDEILFQIYQPREPLHFSKFLDPDFQPSCSEVDLIGFVVS
VVKKTGLAPFVYLSDECYNLLAIKFWIDLNEDIIKPHMLIAASNLQWRPESKSGLLTLFAGDFSVFSASPKEGHFQETFNKMKNTVENIDILCNEAENKLMHILHANDPKWSTPTKDCTSGPYTAQIIPGTGNKLLMSSPNCEIYYQSPLSLCMAKRKSVSTPVSAQMT
SKSCKGEKEIDDQKNCKKRRALDFLSRLPLPPPVSPICTFVSPAAQKAFQPPRSCGTKYETPIKKKELNSPQMTPFKKFNEISLLESNSIADEELALINTQALLSGSTGEKQFISVSESTRTAPTSSEDYLRLKRRCTTSLIKEQESSQASTEECEKNKQDTITTKKYI
XXXXXXXXXXXXXXXXXXX1111111111111111111000000000000000000000000000000000000000000000000000000000000000000000000000000000000000000000000000000000000000000000000000000000000
00000000000000000000000000000000000000000000000000000000000000000000000000000000000000000000000000000000000000000000000000000000000000000000000000000000000000000000000000000000
00000000000000000000000000000000000000000000000000000000000000000000000000000000000000000000000000000000000000000000000000000000000000000000000000000000000000000000000000000000
00000000000000000000000000000000000000000000000000000000000000000000000000000000000000000000000000000000000000000000000000000000000000000000000000000000000000000000000000000000
00000000000000000000000000000000000000000000000000000000000000000000000000000000000000000000000000000000000000000000000000000000000000000000000000000000000000000000000000000000
00000000000000000000000000000000000000000000000000000000000000000000000000000000000000000000000000000000000000000000000000000000000000000000000000000000000000000000000000000000
00000000000000000000000000000000000000000000000000000000000000000000000000000000000000000000000000000000000000000000000000000000000000000000000000000000000000000000000000000000
00000000000000000000000000000000000000000000000000000000000000000000000000000000000000000000000000000000000000000000000000000000000000000000000000000000000000000000000000000000
00000000000000000000000000000000000000000000000000000000000000000000000000000000000000000000000000000000000000000000000000000000000000000000000000000000000000000000000000000000
00000000000000000000000000000000000000000000000000000000000000000000000000000000000000000000000000000000000000000000000000000000000000000000000000000000000000000000000000000000
00000000000000000000000000000000000000000000000000000000000000000000000000000000000000000000000000000000000000000000000000000000000000000000000000000000000000000000000000000000
00000000000000000000000000000000000000000000000000000000000000000000000000000000000000000000000000000000000000000000000000000000000000000000000000000000000000000000000000000000
00000000000000000000000000000000000000000000000000000000000000000000000000000000000000000000000000000000000000000000000000000000000000000000000000000000000000000000000000000000
00000000000000000000000000000000000000000000000000000000000000000000000000000000000000000000000000000000000000000000000000000000000000000000000000000000000000000000000000000000
00000000000000000000000000000000000000000000000000000000000000000000000000000000000000000000000000000000000000000000000000000000000000000000000000000000000000000000000000000000
00000000000000000000000000000000000000000000000000000000000000000000000000000000000000000000000000000000000000000000000000000000000000000000000000000000000000000000000000000000
00000000000000000000000000000000000000000000000000000000000000000000000000000000000000000000000000000000000000000000000000000000000000000000000000000000000000000000000000000000

0000000000000000000000000000000000000000000000000000000000000000000000000000000000000000000000000000000000000000000000000000000000000000000000000000000000000000000000000000000000
00000000000000000000000000000000000000000000000000000000000000000000000000
XXXXXXXXXXXXXXXXXXXX11111111111111111110000000000000000000000000000000000000000000000000000000000000000000000000000000000000000000000000000000000000000000000000000000
0000000000000000000000000000000000000000000000000000000000000000000000000000000000000000000000000000000000000000000000000000000000000000000000000000000000000000000000000000000000
0000000000000000000000000000000000000000000000000000000000000000000000000000000000000000000000000000000000000000000000000000000000000000000000000000000000000000000000000000000000
0000000000000000000000000000000000000000000000000000000000000000000000000000000000000000000000000000000000000000000000000000000000000000000000000000000000000000000000000000000000
0000000000000000000000000000000000000000000000000000000000000000000000000000000000000000000000000000000000000000000000000000000000000000000000000000000000000000000000000000000000
0000000000000000000000000000000000000000000000000000000000000000000000000000000000000000000000000000000000000000000000000000000000000000000000000000000000000000000000000000000000
0000000000000000000000000000000000000000000000000000000000000000000000000000000000000000000000000000000000000000000000000000000000000000000000000000000000000000000000000000000000
0000000000000000000000000000000000000000000000000000000000000000000000000000000000000000000000000000000000000000000000000000000000000000000000000000000000000000000000000000000000
0000000000000000000000000000000000000000000000000000000000000000000000000000000000000000000000000000000000000000000000000000000000000000000000000000000000000000000000000000000000
0000000000000000000000000000000000000000000000000000000000000000000000000000000000000000000000000000000000000000000000000000000000000000000000000000000000000000000000000000000000
0000000000000000000000000000000000000000000000000000000000000000000000000000000000000000000000000000000000000000000000000000000000000000000000000000000000000000000000000000000000
0000000000000000000000000000000000000000000000000000000000000000000000000000000000000000000000000000000000000000000000000000000000000000000000000000000000000000000000000000000000
0000000000000000000000000000000000000000000000000000000000000000000000000000000000000000000000000000000000000000000000000000000000000000000000000000000000000000000000000000000000
0000000000000000000000000000000000000000000000000000000000000000000000000000000000000000000000000000000000000000000000000000000000000000000000000000000000000000000000000000000000
0000000000000000000000000000000000000000000000000000000000000000000000000000000000000000000000000000000000000000000000000000000000000000000000000000000000000000000000000000000000
0000000000000000000000000000000000000000000000000000000000000000000000000000000000000000000000000000000000000000000000000000000000000000000000000000000000000000000000000000000000
0000000000000000000000000000000000000000000000000000000000000000000000000000000000000000000000000000000000000000000000000000000000000000000000000000000000000000000000000000000000
0000000000000000000000000000000000000000000000000000000000000000000000000000000000000000000000000000000000000000000000000000000000000000000000000000000000000000000000000000000000
0000000000000000000000000000000000000000000000000000000000000000000000000000000000000000000000000000000000000000000000000000000000000000000000000000000000000000000000000000000000
0000000000000000000000000000000000000000000000000000000000000000000000000000000000000000000000000000000000000000000000000000000000000000000000000000000000000000000000000000000000
00000000000000000000000000000000000000000000000000000000000000000000000000
XXXXXXXXXXXXXXXXXXXX00000000000000000000000000000000000000000000000000000000000000000000000000000000000000000000000000000000000000000000000000000000000000000000000000
0000000000000000000000000000000000000000000000000000000000000000000000000000000000000000000000000000000000000000000000000000000000000000000000000000000000000000000000000000000000
0000000000000000000000000000000000000000000000000000000000000000000000000000000000000000000000000000000000000000000000000000000000000000000000000000000000000000000000000000000000
0000000000000000000000000000000000000000000000000000000000000000000000000000000000000000000000000000000000000000000000000000000000000000000000000000000000000000000000000000000000
0000000000000000000000000000000000000000000000000000000000000000000000000000000000000000000000000000000000000000000000000000000000000000000000000000000000000000000000000000000000
0000000000000000000000000000000000000000000000000000000000000000000000000000000000000000000000000000000000000000000000000000000000000000000000000000000000000000000000000000000000
0000000000000000000000000000000000000000000000000000000000000000000000000000000000000000000000000000000000000000000000000000000000000000000000000000000000000000000000000000000000
0000000000000000000000000000000000000000000000000000000000000000000000000000000000000000000000000000000000000000000000000000000000000000000000000000000000000000000000000000000000
0000000000000000000000000000000000000000000000000000000000000000000000000000000000000000000000000000000000000000000000000000000000000000000000000000000000000000000000000000000000
0000000000000000000000000000000000000000000000000000000000000000000000000000000000000000000000000000000000000000000000000000000000000000000000000000000000000000000000000000000000
0000000000000000000000000000000000000000000000000000000000000000000000000000000000000000000000000000000000000000000000000000000000000000000000000000000000000000000000000000000000
0000000000000000000000000000000000000000000000000000000000000000000000000000000000000000000000000000000000000000000000000000000000000000000000000000000000000000000000000000000000
0000000000000000000000000000000000000000000000000000000000000000000000000000000000000000000000000000000000000000000000000000000000000000000000000000000000000000000000000000000000
0000000000000000000000000000000000000000000000000000000000000000000000000000000000000000000000000000000000000000000000000000000000000000000000000000000000000000000000000000000000
0000000000000000000000000000000000000000000000000000000000000000000000000000000000000000000000000000000000000000000000000000000000000000000000000000000000000000000000000000000000
0000000000000000000000000000000000000000000000000000000000000000000000000000000000000000000000000000000000000000000000000000000000000000000000000000000000000000000000000000000000
0000000000000000000000000000000000000000000000000000000000000000000000000000000000000000000000000000000000000000000000000000000000000000000000000000000000000000000000000000000000
0000000000000000000000000000000000000000000000000000000000000000000000000000000000000000000000000000000000000000000000000000000000000000000000000000000000000000000000000000000000
0000000000000000000000000000000000000000000000000000000000000000000000000000000000000000000000000000000000000000000000000000000000000000000000000000000000000000000000000000000000
00000000000000000000000000000000000000000000000000000000000000000000000000
>Q99075
MKLLPSVVLKLFLAAVLSALVTGESLERLRRGLAAGTSNPDPPTVSTDQLLPLGGGRDRKVRDLQEADLDLLRVTLSSKPQALATPNKEEHGKRKKKGKGLGKKRDPCLRKYKDFCIHGECKYVKELRAPSCICHPGYHGERCHGLSLPVENRLYTYDHTTILAVVAVVL
SSVCLLVIVGLLMFRYHRRGGYDVENEEKVKLGMTNSH
XXXXXXXXXXXXXXXXXXXXXXXXXXXXXXXXXXXXXXXXXXXXXXXXXXXXXXXXXXXXXXXXXXXXXXXXXXX1111111111111111111111111111111111000000000000000000000000000000000000000000000000000000000000000000
000000000000000000000000000000000
XXXXXXXXXXXXXXXXXXXXXXXXXXXXXXXXXXXXXXXXXXXXXXXXXXXXXXXXXXXXXXXXXXXXXXXXXXX0000000000000000000000000000000000000000000000000000000000000000000000000000000000000000000000000000
000000000000000000000000000000000
XXXXXXXXXXXXXXXXXXXXXXXXXXXXXXXXXXXXXXXXXXXXXXXXXXXXXXXXXXXXXXXXXXXXXXXXXXX0000000000000000000000000000000000000000000000000000000000000000000000000000000000000000000000000000
000000000000000000000000000000000
>O95149
MEELSQALASSFSVSQDLNSTAAPHPRLSQYKSKYSSLEQSERRRRLLELQKSKRLDYVNHARRLAEDDWTGMESEEENKKDDEEMDIDTVKKLPKHYANQLMLSEWLIDVPSDLGQEWIVVVCPVGKRALIVASRGSTSAYTKSGYCVNRFSSLLPGGNRRNSTAK
DYTILDCIYNEVNQTYYVLDVMCWRGHPFYDCQTDFRFYWMHSKLPEEEGLGEKTKLNPFKFVGLKNFPCTPESLCDVLSMDFPFEVDGLLFYHKQTHYSPGSTPLVGWLRPYMVSDVLGVAVPAGPLTTKPDYAGHQLQQIMEHKKSQKEGMKEKLTHKASEN
GHYELEHLSTPKLKGSSHSPDHPGCLMEN
0000000000000000000000000000000000000000000000000000000000000000000000000000111111111111111111110000000000000000000000000000000000000000000000000000000000000000000000000000000
0000000000000000000000000000000000000000000000000000000000000000000000000000000000000000000000000000000000000000001111111111111111111111111111111111111111111111111111111111110000
00000000
0000000000000000000000000000000000000000000000000000000000000000000000000000000000000000000000000000000000000000000000000000000000000000000000000000000000000000000000000000000000
0000000000000000000000000000000000000000000000000000000000000000000000000000000000000000000000000000000000000000000000000000000000000000000000000000000000000000000000000000000000
00000000
0000000000000000000000000000000000000000000000000000000000000000000000000000000000000000000000000000000000000000000000000000000000000000000000000000000000000000000000000000000000
0000000000000000000000000000000000000000000000000000000000000000000000000000000000000000000000000000000000000000000000000000000000000000000000000000000000000000000000000000000000
00000000
>Q2GI62
MDIDNSNISTADIRSNTDGLIDIIMRILGFGNKNIVQPQDLGSEIYQQEQEDDTVSQPSLEPFVAESEVSKVEQEKTNPEVLIKDLQDVASHESGVSDQPAQVVTERENEIESHQGETEKESGITESHQKEDEIVSQSSSEPFVAESEVSKVEQEETNPEVLIKDLQDVAS
HESGVSDQPAQVVTERESEIESHQGETEKESGITESHQKEDEIVSQPSSEPFVAESEVSKVEQEETNPEVLIKDLQDVASHESGVSDQPAQVVTERESEIESHQGETEKESGITESHQKEDEIVSQPSSEPFVAESEVSKVEQEETNPEVLIKDLQDVASHESGVSDQPA
QVVTERESEIESHQGETEKESGITESHQKEDEIVSQPSSEPFVAESEVSKVEQEKTNPEILVEDLPLGQVIPVVVEKDEMFAPSFNPIVIKEEDKVCETCEQEFEIVKDSQTVKGSEDIISPIECLESMDSIVSTIFESGMLCPMSKPGQYVCGYEMYMYGFQDVKDLLGGL
LSNVPVCCNVSLYFMEHNYFTNHENINHNVVNDIV
XXXXXXXXXXXXXXXXXXXXXXXXXXXXXXXXXXXXXXXXXXXXXXXXXXXXXXXXXXXXXXXXXXXXXXXXXXXXXXXXXXXXXXXXXXXXXXXXXXXXXXXXXXXXXXXXXXXXXXXXXXXXXXXXXXXXXXXXX11111111111111111111111111111111
111111111111111111111111111111111111111111111111XXXXXXXXXXXXXXXXXXXXXXXXXXXXXXXXXXXXXXXXXXXXXXXXXXXXXXXXXXXXXXXXXXXXXXXXXXXXXXXXXXXXXXXXXXXXXXXXXXXXXXXXXXXXXXXXXXXXXXXXXXXXX
XXXXXXXXXXXXXXXXXXXXXXXXXXXXXXXXXXXXXXXXXXXXXXXXXXXXXXXXXXXXXXXXXXXXXXXXXXXXXXXXXXXXXXXXXXXXXXXXXXXXXXXXXXXXXXXXXXXXXXXXXXXXXXXXXXXXXXXXXXXXXXXXXXXXXXXXXXXXXXXXXXXXXXXXXXXX
XXXXXXXXXXXXXXXXXXXXXXXXXXXXXX
XXXXXXXXXXXXXXXXXXXXXXXXXXXXXXXXXXXXXXXXXXXXXXXXXXXXXXXXXXXXXXXXXXXXXXXXXXXXXXXXXXXXXXXXXXXXXXXXXXXXXXXXXXXXXXXXXXXXXXXXXXXXXXXXXXXXXXXXX00000000000000000000000000000000
000000000000000000000000000000000000000000000000XXXXXXXXXXXXXXXXXXXXXXXXXXXXXXXXXXXXXXXXXXXXXXXXXXXXXXXXXXXXXXXXXXXXXXXXXXXXXXXXXXXXXXXXXXXXXXXXXXXXXXXXXXXXXXXXXXXXXXXXXXXXX
XXXXXXXXXXXXXXXXXXXXXXXXXXXXXXXXXXXXXXXXXXXXXXXXXXXXXXXXXXXXXXXXXXXXXXXXXXXXXXXXXXXXXXXXXXXXXXXXXXXXXXXXXXXXXXXXXXXXXXXXXXXXXXXXXXXXXXXXXXXXXXXXXXXXXXXXXXXXXXXXXXXXXXXXXXXXX
XXXXXXXXXXXXXXXXXXXXXXXXXXXXXX
XXXXXXXXXXXXXXXXXXXXXXXXXXXXXXXXXXXXXXXXXXXXXXXXXXXXXXXXXXXXXXXXXXXXXXXXXXXXXXXXXXXXXXXXXXXXXXXXXXXXXXXXXXXXXXXXXXXXXXXXXXXXXXXXXXXXXXXXX11111111111111111111111111111111
111111111111111111111111111111111111111111111111XXXXXXXXXXXXXXXXXXXXXXXXXXXXXXXXXXXXXXXXXXXXXXXXXXXXXXXXXXXXXXXXXXXXXXXXXXXXXXXXXXXXXXXXXXXXXXXXXXXXXXXXXXXXXXXXXXXXXXXXXXXXX
XXXXXXXXXXXXXXXXXXXXXXXXXXXXXXXXXXXXXXXXXXXXXXXXXXXXXXXXXXXXXXXXXXXXXXXXXXXXXXXXXXXXXXXXXXXXXXXXXXXXXXXXXXXXXXXXXXXXXXXXXXXXXXXXXXXXXXXXXXXXXXXXXXXXXXXXXXXXXXXXXXXXXXXXXXXXX
XXXXXXXXXXXXXXXXXXXXXXXXXXXXXX
>P52926
MSARGEGAGQPSTSAQGQPAAPAPQKRGRGRPRKQQQEPTGEPSPKRPRGRPKGSKNKSPSKAAQKKAEATGEKRPRGRPRKWPQQVVQKKPAQEETEETSSQESAEED
1111111111111111111111111111111111111111111111111111111111111111111111111111111111111111111111111111111111111
0000000000000000000000000000000000000000000000000000000000000000000000000000000000000000000000000000000000000
1111111111111111111111111111111111111111111111111111111111111111111111111111111111111111111111111111111111111
>Q96AY2
MALKKSSPSLDSGDSDSEELPTFAFLKKEPSSTKRRQPEREEKIVVVDISDCEASCPPAPELFSPPVPEIAETVTQTQPVRLLSSESEDEEEFIPLAQRLTCKFLTHKQLSPEDSSSPVKSVLDHQNNEGASCDWKKPFPKIPEVPLHDTPERSAADNKDLILDPCCQLPAYL
STCPGQSSSLAVTKTNSDILPPQKKTKPSQKVQGRGSHGCRQQRQARQKESTLRRQERKNAALVTRMKAQRPEECLKHIIVVLDPVLLQMEGGGQLLGALQTMECRCVIEAQAVPCSVTWRRRAGPSEDREDWVEEPTVLVLLRAEAFVSMIDNGKQGSLDST
MKGKETLQGFVTDITAKTAGKALSLVIVDQEKCFSAQNPPRRGKQGANKQTKKQQQRQPEASIGSMVSRVDAEEALVDLQLHTEAQAQIVQSWKELADFTCAFTKAVAEAPFKKLRDETTFSFCLESDWAGGVKVDLAGRGLALVWRRQIQQLNRVSLEMASA
VVNAYPSPQLLVQAYQQCFSDKERQNLLADIQVRRGEGVTSTSRRIGPELSRRIYLQMTTLQPHLSLDSAD
0000000000000000000000000000000000000000000000000000000000000000000000000000000000000000000000000000000000000000000000000000000000000000000000000000000000000000000000000000000000
0000000000000000000000000000000000000000000000000000000000000000000000000000000000000000000000000000000000000000000000000000000000000000000000000000000000000000000000000000000000
0000000000000000000000000000000000000000000000000000000000000000000000111111111110000000000000000000000000000000000000000000000000000000000000000000000000000000000000000000000
000000000000000000000000000000000000000000
0000000000000000000000000000000000000000000000000000000000000000000000000000000000000000000000000000000000000000000000000000000000000000000000000000000000000000000000000000000000
0000000000000000000000000000000000000000000000000000000000000000000000000000000000000000000000000000000000000000000000000000000000000000000000000000000000000000000000000000000000
0000000000000000000000000000000000000000000000000000000000000000000000000000000000000000000000000000000000000000000000000000000000000000000000000000000000000000000000000000000000
000000000000000000000000000000000000000000
0000000000000000000000000000000000000000000000000000000000000000000000000000000000000000000000000000000000000000000000000000000000000000000000000000000000000000000000000000000000
0000000000000000000000000000000000000000000000000000000000000000000000000000000000000000000000000000000000000000000000000000000000000000000000000000000000000000000000000000000000
0000000000000000000000000000000000000000000000000000000000000000000000000000000000000000000000000000000000000000000000000000000000000000000000000000000000000000000000000000000000
000000000000000000000000000000000000000000
>Q9UPR3
MSQGPPTGESSEPEAKVLHTKRLYRAVVEAVHRLDLILCNKTAYQEVFKPENISLRNKLRELCVKLMFLHPVDYGRKAEELLWRKVYYEVIQLIKTNKKHIHSRSTLECAYRTHLVAGIGFYQHLLLYIQSHYQLELQCCIDWTHVTDPLIGCKKPVSASGKEMDWAQM
ACHRCLVYLGDLSRYQNELAGVDTELLAERFYYQALSVAPQIGMPFNQLGTLAGSKYYNVEAMYCYLRCIQSEVSFEGAYGNLKRLYDKAAKMYHQLKKCETRKLSPGKKRCKDIKRLLVNFMYLQSLLQPKSSSVDSELTSLCQSVLEDFNLCLFYLPSSPNLSLASED
EEEYESGYAFLPDLLIFQMVIICLMCVHSLERAGSKQYSAAIAFTLALFSHLVNHVNIRLQAELEEGENPVPAFQSDGTDEPESKEPVEKEEEPDPEPPPVTPQVGEGRKSRKFSRLSCLRRRRHPPKVGDDSDLSEGFESDSSHDSARASEGSDSGSDKSLEGGGTAFD
AETDSEMNSQESRSDLEDMEEEEGTRSPTLEPPRGRSEAPDSLNGPLGPSEASIASNLQAMSTQMFQTKRCFRLAPTFSNLLLQPTTNPHTSASHRPCVNGDVDKPSEPASEEGSESEGSESSGRSCRNERSIQEKLQVLMAEGLLPAVKVFLDWLRTNPDLIIVCAQ
SSQSLWNRLSVLLNLLPAAGELQESGLALCPEVQDLLEGCELPDLPSSLLLPEDMALRNLPPLRAAHRRFNFDTDRPLLSTLEESVVRICCIRSFGHFIARLQGSILQFNPEVGIFVSIAQSEQESLLQQAQAQFRMAQEEARRNRLMRDMAQLRLQLEVSQLEGSLQQ
PKAQSAMSPYLVPDTQALCHHLPVIRQLATSGRFIVIIPRTVIDGLDLLKKEHPGARDGIRYLEAEFKKGNRYIRCQKEVGKSFERHKLKRQDADAWTLYKILDSCKQLTLAQGAGEEDPSGMVTIITGLPLDNPSVLSGPMQAALQAAAHASVDIKNVLDFYKQWKEI
G
0000000000000000000000000000000000000000000000000000000000000000000000000000000000000000000000000000000000000000000000000000000000000000000000000000000000000000000000000000000000
0000000000000000000000000000000000000000000000000000000000000000000000000000000000000000000000000000000000000000000000000000000000000000000000000000000000000000000000000000000000
0000000000000000000000000000000000000000000000000000000000000000000000000000000000000000000000000000000000000000000000000000000000000000000000000000000000000000000000000000000000
0000000000000000000000000000000000000000000000000000000000000000000000000000000000000000000000000000000000000000000000000000000000000000000000000000000000000000000000000000000000
0000000000000000000000000000000000000000000000000000000000000000000000000000000000000000000000000000000000000000000000000000000000000000000000000000000000000000000000000000000000
00000000000000000000000000000000000000000001111111111111111111111100000000000000000000000000000000000000000000000000000000000000000000000
0000000000000000000000000000000000000000000000000000000000000000000000000000000000000000000000000000000000000000000000000000000000000000000000000000000000000000000000000000000000
0000000000000000000000000000000000000000000000000000000000000000000000000000000000000000000000000000000000000000000000000000000000000000000000000000000000000000000000000000000000
0000000000000000000000000000000000000000000000000000000000000000000000000000000000000000000000000000000000000000000000000000000000000000000000000000000000000000000000000000000000
0000000000000000000000000000000000000000000000000000000000000000000000000000000000000000000000000000000000000000000000000000000000000000000000000000000000000000000000000000000000
0000000000000000000000000000000000000000000000000000000000000000000000000000000000000000000000000000000000000000000000000000000000000000000000000000000000000000000000000000000000
0000000000000000000000000000000000000000000000000000000000000000000000000000000000000000000000000000000000000000000000000000000000000000
0000000000000000000000000000000000000000000000000000000000000000000000000000000000000000000000000000000000000000000000000000000000000000000000000000000000000000000000000000000000
0000000000000000000000000000000000000000000000000000000000000000000000000000000000000000000000000000000000000000000000000000000000000000000000000000000000000000000000000000000000
0000000000000000000000000000000000000000000000000000000000000000000000000000000000000000000000000000000000000000000000000000000000000000000000000000000000000000000000000000000000
0000000000000000000000000000000000000000000000000000000000000000000000000000000000000000000000000000000000000000000000000000000000000000000000000000000000000000000000000000000000

00000000000000000000000000000000000000000000000000000000000000000000000000000000000000000000000000000000000000000000000000000000000000000000000000000000000000000000000000000000
0000000000000000000000000000000000000000000000000000000000000000000000000000000000000000000000000000000000000000000000000000000000000000
>P0C077
MAYFLDFDERALKEWRKLGSTVREQLKKKLVEVLESPRIEANKLRGMPDCYKIKLRSSGYRLVYQVIDEKVVVFVISVGKRERSEVYSEAVKRIL
00000000000000000000000000000000000000000000000000000000000000000000000000000000000001111111111
00000000000000000000000000000000000000000000000000000000000000000000000000000000000000000000000
00000000000000000000000000000000000000000000000000000000000000000000000000000000000000000000000
>Q9UKT5
MAGSEPRSGTNSPPPPFSDWGRLEAAILSGWKTFWQSVSKERVARTTSREEVDEAASTLTRLPIDVQLYILSFLSPHDLCQLGSTNHYWNETVRDPILWRYFLLRDLPSWSSVDWKSLPDLEILKKPISEVTDGAFFDYMAVYRMCCPYTRRASKSSRPMYGAVTSFL
HSLIIQNEPRFAMFGPGLEELNTSLVLSLMSSEELCPTAGLPQRQIDGIGSGVNFQLNNQHKFNILILYSTTRKERDRAREEHTSAVNKMFSRHNEGDDQQGSRYSVIPQIQKVCEVVDGFIYVANAEAHKRHEWQDEFSHIMAMTDPAFGSSGRPLLVLSCISQGD
VKRMPCFYLAHELHLNLLNHPWLVQDTEAETLTGFLNGIEWILEEVESKRAR
00000000000000000000000000000000000000000000000000000000000000000000000000000000000000000000000000000000000000000000000000000000000000000000000000000000000000000000000000000000
00000000000000000000000000000000000000000000000000000000000000000111111111111111111111110000000000000000000000000000000000000000000000000000000000000000000000000000000000000000
00000000000000000000000000000000000
00000000000000000000000000000000000000000000000000000000000000000000000000000000000000000000000000000000000000000000000000000000000000000000000000000000000000000000000000000000
00000000000000000000000000000000000000000000000000000000000000000000000000000000000000000000000000000000000000000000000000000000000000000000000000000000000000000000000000000000
00000000000000000000000000000000000
00000000000000000000000000000000000000000000000000000000000000000000000000000000000000000000000000000000000000000000000000000000000000000000000000000000000000000000000000000000
00000000000000000000000000000000000000000000000000000000000000000000000000000000000000000000000000000000000000000000000000000000000000000000000000000000000000000000000000000000
00000000000000000000000000000000000
>P77522
MSRNTEATDDVKTWTGGPLNYKEGFFTQLATDELAKGINEEVVRAISAKRNEPEWMLEFRLNAYRAWLEMEEPHWLKAHYDKLNYQDYSYYSAPSCGNCDDTCASEPGAVQQTGANAFLSKEVEAAFEQLGVPVREGKEVAVDAIFDSVSVATTYREKLAEQGI
IFCSFGEAIHDHPELVRKYLGTVVPGNDNFFAALNAAVASDGTFIYVPKGVRCPMELSTYFRINAEKTGQFERTILVADEDSYVSYIEGCSAPVRDSYQLHAAVVEVIIHKNAEVKYSTVQNWFPGDNNTGGILNFVTKRALCEGENSKMSWTQSETGSAITWKYPSCI
LRGDNSIGEFYSVALTSGHQQADTGTKMIHIGKNTKSTIISKGISAGHSQNSYRGLVKIMPTATNARNFTQCDSMLIGANCGAHTFPYVECRNNSAQLEHEATTSRIGEDQLFYCLQRGISEEDAISMIVNGFCKDVFSELPLEFAVEAQKLLAISLEHSVG
11111111111111111111111111111111111111111000000000000000000000000000000000000000000000000011111111111111111111111111111111111111111111111111111111111100000000000000000000000000
00000000000000000000000000000000000000000000000000000000000000000000000000000000000000000000000000000000000000000000000000000000000000000000000000000000000000000000000000000000
00000000000000000000000000000000000000000000000000000000000000000000000000000000000000000000000000000000000000000000000000000000000000000000000
00000000000000000000000000000000000000000000000000000000000000000000000000000000000000000000000000000000000000000000000000000000000000000000000000000000000000000000000000000000
00000000000000000000000000000000000000000000000000000000000000000000000000000000000000000000000000000000000000000000000000000000000000000000000000000000000000000000000000000000
00000000000000000000000000000000000000000000000000000000000000000000000000000000000000000000000000000000000000000000000000000000000000000000000
00000000000000000000000000000000000000000000000000000000000000000000000000000000000000000000000000000000000000000000000000000000000000000000000000000000000000000000000000000000
00000000000000000000000000000000000000000000000000000000000000000000000000000000000000000000000000000000000000000000000000000000000000000000000000000000000000000000000000000000
00000000000000000000000000000000000000000000000000000000000000000000000000000000000000000000000000000000000000000000000000000000000000000000000
>Q8BH64
MFSWLKKGGARGQRPEAIRTVTSSLKELYRTKLLPLEEHYRFGSFHSPALEDADFDGKPMVLVAGQYSTGKTSFIQYLLEQEVPGSRVGPEPTTDCFVAVMHGETEGTVPGNALVVDPEKPFRKLNPFGNTFLNRFMCAQLPNQVLESISIIDTPGILSGAKQRVSRG
YDFPAVLRWFAERVDLIILLFDAHKLEISDEFSEAIGALRGHEDKIRVVLNKADMVETQQLMRVYGALMWALGKVVGTPEVLRVYIGSFWSQPLLVPDNRRLFELEEQDLFRDIQGLPRHAALRKLNDLVKRARLVRVHAYIISYLKKEMPTVFGKENKKKQLILKLPVI
FAKIQLEHHISPGDFPDCQKMQELLMAHDFTKFHSLKPKLLEALDDMLAQDIAKLMPLLRQEELESVEAGVQGGAFEGTRMGPFVERGPDEAIEDGEEGSEDDAEWVVTKDKSKYDEIFYNLAPADGKLSGSKAKTWMVGTKLPNSVLGRIWKLSDVDRDGMLD
DEEFALASHLIEAKLEGHGLPTNLPRRLVPPSKRRQKGSAE
11111111111111111100000000000000000000000000000000000000000000000000000000000000000000000000000000000000001111111111111111111111000000000000000000000000000000000000000000000000
00000000000000000000000000000000000000000000000000000000000000000000000000000000000000000000000000000000000000000000000000000000000000000000000000000000000000000000000000000000
00000000000000000000000000000000000000000000000000000000000000000000000000000000000000000000000000000000000000000000000000000000000000000000000000000000000000000000000000000000
000000000000000
00000000000000000000000000000000000000000000000000000000000000000000000000000000000000000000000000000000000000000000000000000000000000000000000000000000000000000000000000000000
00000000000000000000000000000000000000000000000000000000000000000000000000000000000000000000000000000000000000000000000000000000000000000000000000000000000000000000000000000000
00000000000000000000000000000000000000000000000000000000000000000000000000000000000000000000000000000000000000000000000000000000000000000000000000000000000000000000000000000000
000000000000000
00000000000000000000000000000000000000000000000000000000000000000000000000000000000000000000000000000000000000000000000000000000000000000000000000000000000000000000000000000000
00000000000000000000000000000000000000000000000000000000000000000000000000000000000000000000000000000000000000000000000000000000000000000000000000000000000000000000000000000000
00000000000000000000000000000000000000000000000000000000000000000000000000000000000000000000000000000000000000000000000000000000000000000000000000000000000000000000000000000000
000000000000000
>Q9NRY2
MAANSSGQGFQNKNRVAILAELDKEKRKLLMQNQSSTNHPGASIALSRPSLNKDFRDHAEQQHIAAQQKAALQHAHAHSSGYFITQDSAFGNLILPVLPRLDPE
11111111111111111111111111111111111111111111111111111111111111111111111111100000000000000000000000000000
00000000000000000000000000000000000000000000000000000000000000000000000000000000000000000000000000000000
00000000000000000000000000000000000000000000000000000000000000000000000000000000000000000000000000000000
>A0A2K8FR49
MASHGQSYRAGEATGRAEEKADQMMGKTRGATEAMKNKAQQAKDKTMETAQAAKEKTSEKAYEAKDKTSNMAQSAKGQAQQSKDQTGSFASDKAQAAKDKAYQAAQATKEKASDMTEYAKETAEAGKEKTGGILQRTGEQVKSMAQGAADAVKNTFG
MGEPEEDDPIKTTRP
1111111111111111111111111111111111111111111111111111111111111111111111111111111111111111111111111111111111111111111111111111111111111111111111111111111111111111111111111111
0000000000000000000000000000000000000000000000000000000000000000000000000000000000000000000000000000000000000000000000000000000000000000000000000000000000000000000000000000
0000000000000000000000000000000000000000000000000000000000000000000000000000000000000000000000000000000000000000000000000000000000000000000000000000000000000000000000000000
>S6AWC2
MEGEKNSSGDLMSSSKLVAEAAKTAYEKKSVEGIDKEKVAAASADILDSAAKYGKLEDKPVGQYLEKAEGYLKQYSSGGTEKEKTDAPAAADAPKPDAPKEAAPAPAPAAEEEKSSDGFGLDDVMKGAASLSGKKSGEEEKESGGGGGFMKMAQGFMK
11111111111111111111111111111111111111111111111111111111111111111111111111111111111111111111111111111111111111111111111111111111111111111111111111111111111111
00000000000000000000000000000000000000000000000000000000000000000000000000000000000000000000000000000000000000000000000000000000000000000000000000000000000000
00000000000000000000000000000000000000000000000000000000000000000000000000000000000000000000000000000000000000000000000000000000000000000000000000000000000000
>Q9HZJ8
MQTSHSLPSAQLPLFQEAFWASNGAPLLDDVIDSPSSASIEEPAAFSELSLSGLPGHCLTLLAPILRELSEEQDARWLTLIAPPASLTHEWLRRAGLNRERILLLQAKDNAAALALSCEALRLGRSHTVVSWLEPLSRAARKQLSRAAQLGQAQSLNIRLG
11111111111111111111111111111111111111111110000000000000000000000000000000000000000000000000000000000000000000000000000000000000000000000000000000000000000000000
00000000000000000000000000000000000000000000000000000000000000000000000000000000000000000000000000000000000000000000000000000000000000000000000000000000000000000
00000000000000000000000000000000000000000000000000000000000000000000000000000000000000000000000000000000000000000000000000000000000000000000000000000000000000000
>A0A0P6V0V4
MSRFNLLDEPWISVIYDEKGSTKDVSLQDLFTNAHQYKELAGDTKTQDFAVLRVLLAVLHTVFSRFDIDGNAYEYLTIDEGWNQLEPVDEMDIENYEEALYETWEKLWTNKRFPNIVNQYLEKWRDRFYLFDQKYPFFQVTKEDIAGDKISKAKGTSILGKNINRIISE
SGNKIALFSPKDEENKNTLTAAELARWLITFQGYSGVSDKVIFGNEKYTPSKGWLFDIGAIYIKGNTLFETLLLNYISPYNECGNVENIQRPCWERKSSDIIKSYLDEKDITNIASLYTVWSKGIYIDPDFNLNKPFSFDIVKLPDINHRDNFLEPMTLWKYNVSGKNRDSYT
PKKHLLNQSLWRSFGLLSIKDTDLQHRKPGVIEWLTYIDDIIGNRLSNIVAISMQDDGNPQSRLPTDEVIDSIFINDLVLTDLDEGGWVPRINEVVEETKKIISRTYKTYINDIKEIRNISNGSYTQQIVESLYFKIDQPFRQWLASIQPEDDKDSKIQEWRVLLKKIVKAEAE
VILHQGGARDYLGIQKDGRIKNIATAYNSFDFWLRQQLK
00000000000000000000000000000000000000000000000000000000000000000000000000000000000000000000000000000000000000000000000000000000000111111111111110000000000000000000000000000000000
00000000000000000000000000000000000000000000000000000000000000000000000000000000000000000000000000000000000000000000000000000000000000000000000000000000000000000000000000000000
00000000000000000000000000000000000000000000000000000000000000011111111110000000000000000000000000000000000000000000000000000000000000000000000000000000000000000000000000000000
000000000000000000000000000
00000000000000000000000000000000000000000000000000000000000000000000000000000000000000000000000000000000000000000000000000000000000000000000000000000000000000000000000000000000
00000000000000000000000000000000000000000000000000000000000000000000000000000000000000000000000000000000000000000000000000000000000000000000000000000000000000000000000000000000
00000000000000000000000000000000000000000000000000000000000000000000000000000000000000000000000000000000000000000000000000000000000000000000000000000000000000000000000000000000
000000000000000000000000000
00000000000000000000000000000000000000000000000000000000000000000000000000000000000000000000000000000000000000000000000000000000000111111111111110000000000000000000000000000000000
00000000000000000000000000000000000000000000000000000000000000000000000000000000000000000000000000000000000000000000000000000000000000000000000000000000000000000000000000000000
00000000000000000000000000000000000000000000000000000000000000000000000000000000000000000000000000000000000000000000000000000000000000000000000000000000000000000000000000000000
000000000000000000000000000
>A4C1A5
MAKSQQTFSKSEKEKKRLKKRQDKQKKMDARKADRDEHGPSGIQFAYVDHNGNLTDTPPDPELKVEYELEDIQISVSKKEDLPEEDPVRKGKVSFFDSSKGFGFIIDMENNEKYFTHVSGLIDDIVENDKVSFELERGMRGMNAVKVTKI
111111111111111111111111111111111111111111111111111111111111111111111111111111111111100000000000000000000000000000000000000000000000000000000000000000
000000000000000000000000000000000000000000000000000000000000000000000000000000000000000000000000000000000000000000000000000000000000000000000000000000
000000000000000000000000000000000000000000000000000000000000000000000000000000000000000000000000000000000000000000000000000000000000000000000000000000
>P27058
MGTPSYDIKNKGDDMQEEPKVKLHHEKGGDEKEKIIEKETPSQDINNKDTISSYVLRDDTQEIPKMEHEEGGYVKEKIVEKETISQYIIKIEGDDDAQEKLKVEYEEEEYEKEKIVEKETPSQDINNKGDDAQEKPKVEHEEGDDKETPSQDIIKMEGEGALEITKVVCEKI
IVREDLAVQSKPPSKRDPPKMQTDNNKL
11111111111111111111111111111111111111111111111111111111111111111111111111111111111111111111111111111111111111111111111111111111111111111111111111111111111111111111111111111111
111111111111111111111111
00000000000000000000000000000000000000000000000000000000000000000000000000000000000000000000000000000000000000000000000000000000000000000000000000000000000000000000000000000000
000000000000000000000000
00000000000000000000000000000000000000000000000000000000000000000000000000000000000000000000000000000000000000000000000000000000000000000000000000000000000000000000000000000000
000000000000000000000000
>M1GUG5
TSANSRTHGATSTSTHGATSTAKPAASTPPKAAATSTIKPTVTTPKAAATSTTEPTVTTKPSPAKPAASNTAKPAASTPKKPHDER
11111111111111111111111111111111111111111111111111111111111111111111111111111111111111
00000000000000000000000000000000000000000000000000000000000000000000000000000000000000
00000000000000000000000000000000000000000000000000000000000000000000000000000000000000
>Q09514
MGATLCAPKKKKTQVGASWVGNESENPFDLALNKKDRTLLRETWQRLDDPKDIVGLIFLDIVNDIEPDLKKVFGVDRAPRAAMLKMPKFGGHILRFYEFMEQLTSMLGTSENLTGAWQLVRKTGRSHVRQGFLEQNQNQMEKNYFEIVINVFIERLIPFLTGEQEL
PSSEGKENKKVRFAQNYTTSQITDVWKKFLNTVISQMTDSFELERAKQKSAQTTKALAPHQHIEISERKKKRVAEKQSEIENTAVSNEPKAQEQMFEDPF
00000000000000000000000000000000000000000000000000000000000000000000000000000000000000000000000000000000000000000000000000000000000000000000000000000000000000000011111111111
111110000000000000000000000000000000000001111111111111111111111111111111111111111111111111
00000000000000000000000000000000000000000000000000000000000000000000000000000000000000000000000000000000000000000000000000000000000000000000000000000000000000000000000000000000
000000000000000000000000000000000000000000000000000000000000000000000000000000000000000000
00000000000000000000000000000000000000000000000000000000000000000000000000000000000000000000000000000000000000000000000000000000000000000000000000000000000000000000000000000000
000000000000000000000000000000000000000000000000000000000000000000000000000000000000000000
>Q02554
MARTKSRKRSGNNQNKNASVVNNKAEIAAMIDARRLEQKKKGGVTNSKGKTNKVVDAKLEKEFKDVLQRFQVQENDTPKEITKDEKNNHVVIVEKNPVMNRKHTAEDELEDTPSDGIEEHLSARKRRKTEKPSLSQLKSQVPYPQIIEWYDCDARYPGLLASIKCT
KNVIPVPSHWQSKKEYLSGRSLLGKRPFELPDIIKKTNIEQMRSTLPQSGLDGQDEKSLKEASRARVQPKMGALDLDYKKLHDVFFKIGANWKPDHLLCFGDVYYENRNLFEETNWKRMVDHKRPGRISQELRAIMNLPEGQLPPWCMKMKDIGLPTGYPDLKIA
GLNWDITNLKGDVYGKIIPNHHSRSKKQGRNYFGALISFETPEFENSKEDTQANAENGRQDDKIDDEVEHKLDHFQEDISEVTSAEEKLERNEEESEKQLYTVLK
00000000000000000000000000000000000000000000000000000000000000000000000000000000000000000000000000000000000000000000000000000000000000000000000000000000000000000000000000000000
00000000000000000000000000000000000000000000000000000000000000000000000000000000000000000000000000000000000000000000000000000000000000000000000000000000000000000000000000000011
1111111111111111XXXXXXXXXXXXXXXXXXXXXXXXXXXXXXXXXXXXXXXXXXXXXXXXXXXXXXXXXXXXXX
00000000000000000000000000000000000000000000000000000000000000000000000000000000000000000000000000000000000000000000000000000000000000000000000000000000000000000000000000000000
00000000000000000000000000000000000000000000000000000000000000000000000000000000000000000000000000000000000000000000000000000000000000000000000000000000000000000000000000000000
0000000000000000XXXXXXXXXXXXXXXXXXXXXXXXXXXXXXXXXXXXXXXXXXXXXXXXXXXXXXXXXXXXXX

00000000000000000000000000000000000000000000000000000000000000000000000000000000000000000000000000000000000000000000000000000000000000000000000000000000000000000000000000000000
00000000000000000000000000000000000000000000000000000000000000000000000000000000000000000000000000000000000000000000000000000000000000000000000000000000000000000000000000000000
0000000000000000000XXXXXXXXXXXXXXXXXXXXXXXXXXXXXXXXXXXXXXXXXXXXXXXXXXXXXXXXXXXXXXXXX
>Q6CMF0
MDQQFVAQLEQALGAIVQPTGSLKDATKTLQSQFYTQSAALPALIHILQNSSNDGIKQLAGVEARKQVSKHWGSLDAATQTSVKQSLLNSAFNEGKDAVRHANARVIASIGSEELDEKKWPELIPNLLQAACDSNPKIRETAIFIILSLLESFNANLALHIDDFLNLFAQ
TINDSASLETRSLSAQALSYVSSLIEEEGEINPQYAAKFASLIPSVVQVLDATIREGDTTNTKLIFNCLNDFLLLDSQLTGNTIADLVKLALQIAVNSDVDEDIRVFAVQFVTSALVYRKSKINQAKLGPEITLAALKVASEEIDVEDELTNEDEAGENEENTPALTALRLISNAS
GELSPSQVGVPIIEHLPTMLSSSNPFERRSILLAISVLVTGSPDYTLSQFDKIIPATVTGLKDSEAVVQLAALKCIVQLSTNLQDEVARYHEQYLPLVIDIIDSAKHVVIYKYATLALDGLLEFIAHNDIIKYLDPLMNKLFQMLETQQSPKLRAAIVSAIGSCAFAAGSGFVPYF
KTSVQYLQQFIQNVSQIEGLSEDDIELKALTFENISTMGRAVKSAAFAEYAEPLVNAAYEAIKTDSARLRESGYAFIANMAKVYGKDFAPFLQTIIPEIFKTLEQEEYQFNFDGDEDFLEGIEDLDEEELQSKFTVNTGIAYEKEVAAAALSELAIASKEHFLEYVEPSLKVLA
EQVNESYGLKETALHSMWAIVKAVLLTANLKEGEYPKGVPSGSYVDASALAVIQTVREVSLNNVIEEVETSMVISVFQDLSEMLRLFGPIIIMDNGDSTHLDQLCREALSVLKGEHACQTIHFEEDVPEDEDLDASETEATLLDVALDIYVALSTNLVGGFAQVFTTAKP
VILQLCQSKSKNKRSFAVGALSEIALGMRDENPFIQELLEALIISLTNDKSLEVRCNASYGVGLLIEYSSFDVSAIYSPVLKSLYEILSVADEKNLATEDDEATKEIVDRTFSNVCGCVARMILKHQNLVPLEHTIPALLSHLPFNTAFEEYDPIFKLFLKLFQEQNSTIINEAPKV
IAIFATVFEKESERIELETNSTLGREENLEKRKQFQSEEIKQQVIELLKHLNQQFNGAVAQNPVLAQVIA
11111111111111111111111111111111111111111111111111111111111111111111100000000000000000000000000000000000000000000000000000000000000000000000000000000000000000000000000000000000
00000000000000000000000000000000000000000000000000000000000000000000000000000000000000000000000000000000000000000000000000000000000000000000000000000000000000000000000000000000
00000000000000000000000000000000000000000000000000000000000000000000000000000000000000000000000000000000000000000000000000000000000000000000000000000000000000000000000000000000
00000000000000000000000000000000000000000000000000000000000000000000011111111111111111111111111111111000000000000000000000000000000000000000000000000000000000000000000000000000
00000000000000000000000000000000000000000000000000000000000000000000001111111111111000000000000000000000000000000000000000000000000000000000000000000000000000000000000000000000
00000000000000000000000000000000000000000000000000000000000000000000000000000000000000000000000000000000000000000000000000000000000000000000000000000000000000000000000000000000
000000000000000000000000000000000000000000000000000000000
00000000000000000000000000000000000000000000000000000000000000000000000000000000000000000000000000000000000000000000000000000000000000000000000000000000000000000000000000000000
00000000000000000000000000000000000000000000000000000000000000000000000000000000000000000000000000000000000000000000000000000000000000000000000000000000000000000000000000000000
00000000000000000000000000000000000000000000000000000000000000000000000000000000000000000000000000000000000000000000000000000000000000000000000000000000000000000000000000000000
00000000000000000000000000000000000000000000000000000000000000000000000000000000000000000000000000000000000000000000000000000000000000000000000000000000000000000000000000000000
00000000000000000000000000000000000000000000000000000000000000000000000000000000000000000000000000000000000000000000000000000000000000000000000000000000000000000000000000000000
00000000000000000000000000000000000000000000000000000000000000000000000000000000000000000000000000000000000000000000000000000000000000000000000000000000000000000000000000000000
000000000000000000000000000000000000000000000000000000000
00000000000000000000000000000000000000000000000000000000000000000000000000000000000000000000000000000000000000000000000000000000000000000000000000000000000000000000000000000000
00000000000000000000000000000000000000000000000000000000000000000000000000000000000000000000000000000000000000000000000000000000000000000000000000000000000000000000000000000000
00000000000000000000000000000000000000000000000000000000000000000000000000000000000000000000000000000000000000000000000000000000000000000000000000000000000000000000000000000000
00000000000000000000000000000000000000000000000000000000000000000000000000000000000000000000000000000000000000000000000000000000000000000000000000000000000000000000000000000000
00000000000000000000000000000000000000000000000000000000000000000000000000000000000000000000000000000000000000000000000000000000000000000000000000000000000000000000000000000000
00000000000000000000000000000000000000000000000000000000000000000000000000000000000000000000000000000000000000000000000000000000000000000000000000000000000000000000000000000000
000000000000000000000000000000000000000000000000000000000
>Q14493
MACRPRSPPRHQSRCDGDASPPSPARWSLGRKRRADGRRWRPEDAEEAEHRGAERRPESFTTPEGPKPRSRCSDWASAVEEDEMRTRVNKEMARYKRKLLINDFGRERKSSSGSSDSKESMSTVPADFETDESVLMRRQKQINYGKNTIAYDRYIKEVPRHLRQ
PGIHPKTPNKFKKYSRRSWDQQIKLWKVALHFWDPPAEEGCDLQEIHPVDLESAESSEPQTSSQDDFDVYSGTPTKVRHMDSQVEDEFDLEACLTEPLRDFSAMS
XXXXXXXXXXXXXXXXXXXXXXXXXXXXXXXXXXXXXXXXXXXXXXXXXXXXXXXXXXXXXXXXXXXXXXXXXXXXXXXXXXXXXXXXXXXXXXXXXXXXXXXXXXXXXXXXXXXXXXXXXXXXXXXXXXXXXXXXXXXXXXXXXXXXXXXXXXXXXXXXXXXXXXXX
XXXXXXXXXXXXXXXXXXXXXXXXX1111111111111111111111111111XXXXXXXXXXXXXXXXXXXXXXXXXXXXXXXXXXXXXXXXXXXXXX
XXXXXXXXXXXXXXXXXXXXXXXXXXXXXXXXXXXXXXXXXXXXXXXXXXXXXXXXXXXXXXXXXXXXXXXXXXXXXXXXXXXXXXXXXXXXXXXXXXXXXXXXXXXXXXXXXXXXXXXXXXXXXXXXXXXXXXXXXXXXXXXXXXXXXXXXXXXXXXXXXXXXXXXXXX
XXXXXXXXXXXXXXXXXXXXXXXXX0000000000000000000000000000XXXXXXXXXXXXXXXXXXXXXXXXXXXXXXXXXXXXXXXXXXXXXX
XXXXXXXXXXXXXXXXXXXXXXXXXXXXXXXXXXXXXXXXXXXXXXXXXXXXXXXXXXXXXXXXXXXXXXXXXXXXXXXXXXXXXXXXXXXXXXXXXXXXXXXXXXXXXXXXXXXXXXXXXXXXXXXXXXXXXXXXXXXXXXXXXXXXXXXXXXXXXXXXXXXXXXXXXXXX
XXXXXXXXXXXXXXXXXXXXXXXXX0000000000000000000000000000XXXXXXXXXXXXXXXXXXXXXXXXXXXXXXXXXXXXXXXXXXXXXX
>E2IHW6
MTNSISGDQPTVTTFTSSTTSASGASGSLGASSVSTTANATVTQTANATNSAATSSIQTTGETVVNYTNSASAPTVTVSTSSSSTQATATSNKTSQAVAGKITSPDTSESSETSSTSSSDHIPSDYEPISTTENIYENIYESIDDSSTSGPENTSGGAAALNSLRGSSYSNYD
DAAADYEPISTTENIYESIDDSSTSDPENTSGGAAALNSLRGSSYSNYDDAAADYEPISTTENIYENIYESIDDSSTSGPENTSGGAAALNSLRGSSYSNYDDAAADYEPISTTENIYESIDDSSTSDPENTSGGAAAALNSLRGSSYSNYDDAAADYEPISTTENIYESIDDSS
TSDPENTSGGAAALNSLRGSSYSNYDDAAADYEPISTTENIYENIYESIDGSSTSDPENTSGGAAAALNSLRGSSYTTGPRNEGVFGPGPEGLPDMSLPSYDPTNKTSLLTFLSNPHVKSKMLENSGHFVFIDTDRSSFILVPNGNWDQVCSIKVQNGKTKEDLDIKDLE
NMCAKFCTGFNKFSGDWDSRVEPMMSAKAGVASGGNLPNTVIINNKFKTCVAYGPWNSREASSGYTPSAWRRGHQVNFGEIFEKANDFNKINWGTQAGPSSEDDGISFSNETPGAGPAAAPSPTPSSIPVINVNVNVGGTNVNIRDTNVNTTNTTPTTQSTD
ASTDTSDIDNINTNNQTDDINTTDKDSDGAGGVNGDISETESSSGDDSGSVSSSESDKNASVGNDGPAMKDILSAVRKHLDVVYPGDNGGSTEGPLQANQTLGDIVQDMETTGTSQETVVSPWKGSTSSTGSAGGSGSVQTLLPSPPPTPSTTTLRTGTGATTTSL
MMGGPIKADIITTGGGGRIPGGGTLEKLLPRIRAHLDISFDGQGDLVSTEEPQLGSIVNKFRKETGSGGIVASVESAPGKPGSAQVLTGTGGDKGNLFQAAAAVTQALGNVAGKVNLAIQGQKLSSLVNDDGKGSVGRDLFQAATQTTQALSSLIDTVG
XXXXXXXXXXXXXXXXXXXXXXXXXXXXXXXXXXXXXXXXXXXXXXXXXXXXXXXXXXXXXXXXXXXXXXXXXXXXXXXXXXXXXXXXXXXXXXXXXXXXXXXXXXXXXXXXXXXXXXXXXXXXXXXXXXXXXXXXXXXXXXXXXXXXXXXXXXXXXXXXXXXXXXXXXX
XXXXXXXXXXXXXXXXXXXXXXXXXXXXXXXXXXXXXXXXXXXXXXXXXXXXXXXXXXXXXXXXXXXXXXXXXXXXXXXXXXXXXXXXXXXXXXXXXXXXXXXXXXXXXXXXXXXXXXXXXXXXXXXXXXXXXXXXXXXXXXXXXXXXXXXXXXXXXXXXXXXXXXXXXX
XXXXXXXXXXXXXXXXXXXXXXXXXXXXXXXXXXXXXXXXXXXXXXXXXXXXXXXXXXXXXXXXXXXXXXXXXXXXXXXXXXXXXXXXXXXXXXXXXXXXXXXXXXXXXXXXXXXXXXXXXXXXXXXXXXXXXXXXXXXXXXXXXXXXXXXXXXXXXXXXXXXXXXXXXX
XXXXXXXXXXXXXXXXXXXXXXXXXXXXXXXXXXXXXXXXXXXXXXXXXXXXXXXXXXXXXXXXXXXXXXXXXXXXXXXXXXXXXXXXXXXXXXXXXXXXXXXXXXXXXXXXXXXXXXXXXXXXXXXXXXXXXXXXXXXXXXXXXXXXXXXXXXXXXXXXXXXXXXXXXX
XXXXXXXXXXXXXXXXXXXXXXXXXXXXXXXXXXXXXXXXXXXXXXXXXXXXXXXXXXXXXXXXXXXXXXXXXXXXXXXXXXXXXXXXXXXXXXXXXXXXXXXXXXXXXXXXXXXXXXXXXXXXXXXXXXXXXXXXXXXXXXXXXXXXXXXXXXXXXXXXXXXXXXXXXX
XXXXXXXXXXXXXXXXXXXXXXXXXXXXXXXXXXXXX11111111111111111111111111111111111111111111111111111111111111111111111111111111111111111111111111XXXXXXXXXXXXXXXXXXXXXXXXXXXXXXXXXXXXXXXXXXXXX
XXXXXXXXXXXXXXXXXXXXXXXXXXXXXXXXXXXXXXXXXXXXXXXXXXXXXXXXXXXXXXXXXXXXXXXXXXXXXXXXXXXXXXXXXXXXXXXXXXXXXXXXXXXXXXXXXXXXXXXXXXXXXXXXXXXXXXXXXXXXX
XXXXXXXXXXXXXXXXXXXXXXXXXXXXXXXXXXXXXXXXXXXXXXXXXXXXXXXXXXXXXXXXXXXXXXXXXXXXXXXXXXXXXXXXXXXXXXXXXXXXXXXXXXXXXXXXXXXXXXXXXXXXXXXXXXXXXXXXXXXXXXXXXXXXXXXXXXXXXXXXXXXXXXXXXX
XXXXXXXXXXXXXXXXXXXXXXXXXXXXXXXXXXXXXXXXXXXXXXXXXXXXXXXXXXXXXXXXXXXXXXXXXXXXXXXXXXXXXXXXXXXXXXXXXXXXXXXXXXXXXXXXXXXXXXXXXXXXXXXXXXXXXXXXXXXXXXXXXXXXXXXXXXXXXXXXXXXXXXXXX
XXXXXXXXXXXXXXXXXXXXXXXXXXXXXXXXXXXXXXXXXXXXXXXXXXXXXXXXXXXXXXXXXXXXXXXXXXXXXXXXXXXXXXXXXXXXXXXXXXXXXXXXXXXXXXXXXXXXXXXXXXXXXXXXXXXXXXXXXXXXXXXXXXXXXXXXXXXXXXXXXXXXXXXXX
XXXXXXXXXXXXXXXXXXXXXXXXXXXXXXXXXXXXXXXXXXXXXXXXXXXXXXXXXXXXXXXXXXXXXXXXXXXXXXXXXXXXXXXXXXXXXXXXXXXXXXXXXXXXXXXXXXXXXXXXXXXXXXXXXXXXXXXXXXXXXXXXXXXXXXXXXXXXXXXXXXXXXXXXX
XXXXXXXXXXXXXXXXXXXXXXXXXXXXXXXXXXXXXXXXXXXXXXXXXXXXXXXXXXXXXXXXXXXXXXXXXXXXXXXXXXXXXXXXXXXXXXXXXXXXXXXXXXXXXXXXXXXXXXXXXXXXXXXXXXXXXXXXXXXXXXXXXXXXXXXXXXXXXXXXXXXXXXXXX
XXXXXXXXXXXXXXXXXXXXXXXXXXXXXXXXXXXXX11111111111111111111111111111111111111111111111111111111111111111111111111111111111111111111111111XXXXXXXXXXXXXXXXXXXXXXXXXXXXXXXXXXXXXXXXXXXXX
XXXXXXXXXXXXXXXXXXXXXXXXXXXXXXXXXXXXXXXXXXXXXXXXXXXXXXXXXXXXXXXXXXXXXXXXXXXXXXXXXXXXXXXXXXXXXXXXXXXXXXXXXXXXXXXXXXXXXXXXXXXXXXXXXXXXXXXXXXXXX
XXXXXXXXXXXXXXXXXXXXXXXXXXXXXXXXXXXXXXXXXXXXXXXXXXXXXXXXXXXXXXXXXXXXXXXXXXXXXXXXXXXXXXXXXXXXXXXXXXXXXXXXXXXXXXXXXXXXXXXXXXXXXXXXXXXXXXXXXXXXXXXXXXXXXXXXXXXXXXXXXXXXXXXXX
XXXXXXXXXXXXXXXXXXXXXXXXXXXXXXXXXXXXXXXXXXXXXXXXXXXXXXXXXXXXXXXXXXXXXXXXXXXXXXXXXXXXXXXXXXXXXXXXXXXXXXXXXXXXXXXXXXXXXXXXXXXXXXXXXXXXXXXXXXXXXXXXXXXXXXXXXXXXXXXXXXXXXXXXX
XXXXXXXXXXXXXXXXXXXXXXXXXXXXXXXXXXXXXXXXXXXXXXXXXXXXXXXXXXXXXXXXXXXXXXXXXXXXXXXXXXXXXXXXXXXXXXXXXXXXXXXXXXXXXXXXXXXXXXXXXXXXXXXXXXXXXXXXXXXXXXXXXXXXXXXXXXXXXXXXXXXXXXXXX
XXXXXXXXXXXXXXXXXXXXXXXXXXXXXXXXXXXXXXXXXXXXXXXXXXXXXXXXXXXXXXXXXXXXXXXXXXXXXXXXXXXXXXXXXXXXXXXXXXXXXXXXXXXXXXXXXXXXXXXXXXXXXXXXXXXXXXXXXXXXXXXXXXXXXXXXXXXXXXXXXXXXXXXXX
XXXXXXXXXXXXXXXXXXXXXXXXXXXXXXXXXXXXXXXXXXXXXXXXXXXXXXXXXXXXXXXXXXXXXXXXXXXXXXXXXXXXXXXXXXXXXXXXXXXXXXXXXXXXXXXXXXXXXXXXXXXXXXXXXXXXXXXXXXXXXXXXXXXXXXXXXXXXXXXXXXXXXXXX
XXXXXXXXXXXXXXXXXXXXXXXXXXXXXXXXXXXXX00000000000000000000000000000000000000000000000000000000000000000000000000000000000000000000000000XXXXXXXXXXXXXXXXXXXXXXXXXXXXXXXXXXXXXXXXXXXXX
XXXXXXXXXXXXXXXXXXXXXXXXXXXXXXXXXXXXXXXXXXXXXXXXXXXXXXXXXXXXXXXXXXXXXXXXXXXXXXXXXXXXXXXXXXXXXXXXXXXXXXXXXXXXXXXXXXXXXXXXXXXXXXXXXXXXXXXXXXXXX
>Q9H2Y7
MPVGRIECPSSPSFPRDISHECRVCGVTEVGLSAYAKHISGQLHKDNVDAQEREDDGKGEEEEEDYFDKELIQLIKQRKEQSRQDEPSNSNQEINSDDRRPQWRREDRIPYQDRESYSQPAWHHRGPPQRDWKWEKDGFNNTRKNSFPHSLRNGGGPRGRSG
WHKGVAGGSSTWFHNHSNSGGGWLSNSGAVDWNHNGTGRNSSWLSEGTGGFSSWHMNNSNGNWKSSVRSTNNWNYSGPGDKFQPGRNRNSNCQMEDMTMLWNKKSNKSNKYSHDRYNWQRQENDKLGTVATYRGPSEGFTSDKFPSEGLLDFNF
EQLESQTTKQADTATSKVSGKNGSAAREKPRRWTPYPSQKTLDLQSGLKDITGNKSEMIEKPLFDFSLITTGIQEPQTDETRNSPTQKTQKEIHTGSLNHKASSDSAASFEVVRQCPTAEKPEQEHTPNKMPSLKSPLLPCPATKSLSQKQDPKNISKNTKTNFFSPGE
HSNPSNKPTVEDNHGPYISKLRSSCPHVLKGNKSTFGSQKQSGDNLNDTLRKAKEVLQCHESLQNPLLSTSKSTRNYAKASRNVEESEKGSLKIEFQVHALEDESDGETSDTEKHGTKIGTLGSATTELLSGSTRTADEKEEDDRILKTSRELSTSPCNPIVRQKESELQM
TSAASPHPGLLLDLKTSLEDAQVDDSIKSHVSYETEGFESASLDAELQKSDISQPSGPLLPELSKLGFPASLQRDLTRHISLKSKTGVHLPEPNLNSARRIRNISGHRKSETEKESGLKPTLRQILNASRRNVNWEQVIQQVTKKKQELGKGLPRFGIEMVPLVQNEQEALD
LDGEPDLSSLEGFQWEGVSISSSPGLARKRSLSESSVIMDRAPSVYSFFSEEGTGKENEPQQMVSPSNSLRAGQSQKATMHLKQEVTPRAASLRTGERAENVATQRRHSAQLSSDHIIPLMHLAKDLNSQERSIPPSENQNSQESNGEGNCLSSSASSALAISSLADA
ATDSSCTSGAEQNDGQSIRKKRRATGDGSSPELPSLERKNKRRKIKGKKERSQVDQLLNISLREEELSKSLQCMDNNLLQARAALQTAYVEVQRLLMLKQQITMEMSALRTHRIQILQGLQETYEPSEHPDQVPCSLTRERRNSRSQTSIDAALLPTPFFPLFLEPPSSH
VSPSPTGASLQITTSPTFQTHGSVPAPDSSVQIKQEPMSPEQDENVNAVPPSSACNVSKELLEANREISDSCPVYPVITARLSLPESTESFHEPSQELKFSVEQRNTRNRENSPSSQSAGLSSINKEGEEPTKGNSGSEACTSSFLRLSFASETPLEKEPHSPADQPEQQAE
STLTSAETRGSKKKKKLRKKKSLRAAHVPENSDTEQDVLTVKPVRKVKAGKLIKGGKVTTSTWEDSRTGREQESVRDEPDSDSSLEVLEIPNPQLEVVAIDSSESGEEKPDSPSKKDIWNSTEQNPLETSRSGCDEVSSTSEIGTRYKDGIPVSVAETQTVISSIKGSKNSS
EISSEPGDDDEPTEGSFEGHQAAVNAIQIFGNLLYTCSADKTVRVYNLVSRKCIGVFEGHTSKVNCLLVTQTSGKNAALYTGSSDHTIRCYNVKSRECVEQLQLEDRVLCLHSRWRILYAGLANGTVVTFNIKNNKRLEIFECHGPRAVSCLATAQEGARKLLVVGSYDC
TISVRDARNGLLLRTLEGHSKTILCMKVVNDLVFSGSSDQSVHAHNIHTGELVRIYKGHNHAVTVVNILGKVMVTACLDKFVRVYELQSHDRLQVYGGHKDMIMCMTIHKSMIYTGCYDGSIQAVRLNLMQNYRCWWHGCSLIFGVVDHLKQHLLTDHTNPNFQ
TLKCRWKNCDAFFTARKGSKQDAAGHIERHAEDDSKIDS
XXXXXXXXXXXXXXXXXXXXXXXXXXXXXXXXXXXXXXXXXXXXXXXXXXXXXXXXXXXXXXXXXXXXXXXXXXXXXXXXXXXXXXXXXXXXXXXXXXXXXXXXXXXXXXXXXXXXXXXXXXXXXXXXXXXXXXXXXXXXXXXXXXXXXXXXXXXXXXXXXXXXXXXX
XXXXXXXXXXXXXXXXXXXXXXXXXXXXXXXXXXXXXXXXXXXXXXXXXXXXXXXXXXXXXXXXXXXXXXXXXXXXXXXXXXXXXXXXXXXXXXXXXXXXXXXXXXXXXXXXXXXXXXXXXXXXXXXXXXXXXXXXXXXXXXXXXXXXXXXXXXXXXXXXXXXXXXXXX
XXXXXXXXXXXXXXXXXXXXXXXXXXXXXXXXXXXXXXXXXXXXXXXXXXXXXXXXXXXXXXXXXXXXXXXXXXXXXXXXXXXXXXXXXXXXXXXXXXXXXXXXXXXXXXXXXXXXXXXXXXXXXXXXXXXXXXXXXXXXXXXXXXXXXXXXXXXXXXXXXXXXXXXXX
XXXXXXXXXXXXXXXXXXXXXXXXXXXXXXXXXXXXXXXXXXXXXXXXXXXXXXXXXXXXXXXXXXXXXXXXXXXXXXXXXXXXXXXXXXXXXXXXXXXXXXXXXXXXXXXXXXXXXXXXXXXXXXXXXXXXXXXXXXXXXXXXXXXXXXXXXXXXXXXXXXXXXXXXX
XXXXXXXXXXXXXXXXXXXXXXXXXXXXXXXXXXXXXXXXXXXXXXXXXXXXXXXXXXXXXXXXXXXXXXXXXXXXXXXXXXXXXXXXXXXXXXXXXXXXXXXXXXXXXXXXXXXXXXXXXXXXXXXXXXXXXXXXXXXXXXXXXXXXXXXXXXXXXXXXXXXXXXXXX
XXXXXXXXXXXXXXXXXXXXXXXXXXXXXXXXXXXXXXXXXXXXXXXXXXXXXXXXXXXXXXXXXXXXXXXXXXXXXXXXXXXXXXXXXXXXXXXXXXXXXXXXXXXXXXXXXXXXXXXXXXXXXXXXXXXXXXXXXXXXXXXXXXXXXXXXXXXXXXXXXXXXXXXX
XXXXXXXXXXXXXXXXXXXXXXXXXXXXXXXXXXXXXXXXXXXXXXXXXXXXXXXXXXXXXXXXXXXXXXXXXXXXXXXXXXXXXXXXXXXXXXXXXXXXXXXXXXXXXXXXXXXXXXXXXXXXXXXXXXXXXXXXXXXXXXXXXXXXXXXXXXXXXXXXXXXXXXXX
XXXXXXXXXXXXXXXXXXXXXXXXXXXXXXXXXXXXXXXXXXXXXXXXXXXXXXXXXXXXXXXXXXXXXXXXXXXXXXXXXXXXXXXXXXXXXXXXXXXXXXXXXXXXXXXXXXXXXXXXXXXXXXXXXXXXXXXXXXXXXXXXXXXXXXXXXXXXXXXXXXXXXXXX
XXXXXXXXXXXXXXXXXXXXXXXXXXXXXXXXXXXXXXXXXXXXXXXXXXXXXXXXXXXX1111111111111111111111111111111111111111111111111111111111111111111111111111111111111111111111111111111111111111111111111111
1111111111111111111111111111111111111111111111111111111111111111111111111111111111111111111111111111111111111111111111111111111111111111110000000000000000000000000000000000000000
00000000000000000000000000000000000000000000000000000000000000000000000000000000000000000000000000000000000000000000000000000000000000000000000000000000000000000000000000000000
00000000000000000000000000000000000000000000000000000000000000000000000000000000000000000000000000000000000000000000000000000000000000000000000000000000
XXXXXXXXXXXXXXXXXXXXXXXXXXXXXXXXXXXXXXXXXXXXXXXXXXXXXXXXXXXXXXXXXXXXXXXXXXXXXXXXXXXXXXXXXXXXXXXXXXXXXXXXXXXXXXXXXXXXXXXXXXXXXXXXXXXXXXXXXXXXXXXXXXXXXXXXXXXXXXXXXXXXXXXXXX
XXXXXXXXXXXXXXXXXXXXXXXXXXXXXXXXXXXXXXXXXXXXXXXXXXXXXXXXXXXXXXXXXXXXXXXXXXXXXXXXXXXXXXXXXXXXXXXXXXXXXXXXXXXXXXXXXXXXXXXXXXXXXXXXXXXXXXXXXXXXXXXXXXXXXXXXXXXXXXXXXXXXXXXXX
XXXXXXXXXXXXXXXXXXXXXXXXXXXXXXXXXXXXXXXXXXXXXXXXXXXXXXXXXXXXXXXXXXXXXXXXXXXXXXXXXXXXXXXXXXXXXXXXXXXXXXXXXXXXXXXXXXXXXXXXXXXXXXXXXXXXXXXXXXXXXXXXXXXXXXXXXXXXXXXXXXXXXXXX
XXXXXXXXXXXXXXXXXXXXXXXXXXXXXXXXXXXXXXXXXXXXXXXXXXXXXXXXXXXXXXXXXXXXXXXXXXXXXXXXXXXXXXXXXXXXXXXXXXXXXXXXXXXXXXXXXXXXXXXXXXXXXXXXXXXXXXXXXXXXXXXXXXXXXXXXXXXXXXXXXXXXXXXXX
XXXXXXXXXXXXXXXXXXXXXXXXXXXXXXXXXXXXXXXXXXXXXXXXXXXXXXXXXXXXXXXXXXXXXXXXXXXXXXXXXXXXXXXXXXXXXXXXXXXXXXXXXXXXXXXXXXXXXXXXXXXXXXXXXXXXXXXXXXXXXXXXXXXXXXXXXXXXXXXXXXXXXXXX
XXXXXXXXXXXXXXXXXXXXXXXXXXXXXXXXXXXXXXXXXXXXXXXXXXXXXXXXXXXXXXXXXXXXXXXXXXXXXXXXXXXXXXXXXXXXXXXXXXXXXXXXXXXXXXXXXXXXXXXXXXXXXXXXXXXXXXXXXXXXXXXXXXXXXXXXXXXXXXXXXXXXXXXX
XXXXXXXXXXXXXXXXXXXXXXXXXXXXXXXXXXXXXXXXXXXXXXXXXXXXXXXXXXXXXXXXXXXXXXXXXXXXXXXXXXXXXXXXXXXXXXXXXXXXXXXXXXXXXXXXXXXXXXXXXXXXXXXXXXXXXXXXXXXXXXXXXXXXXXXXXXXXXXXXXXXXXXXX
XXXXXXXXXXXXXXXXXXXXXXXXXXXXXXXXXXXXXXXXXXXXXXXXXXXXXXXXXXXXXXXXXXXXXXXXXXXXXXXXXXXXXXXXXXXXXXXXXXXXXXXXXXXXXXXXXXXXXXXXXXXXXXXXXXXXXXXXXXXXXXXXXXXXXXXXXXXXXXXXXXXXXXXX
XXXXXXXXXXXXXXXXXXXXXXXXXXXXXXXXXXXXXXXXXXXXXXXXXXXXXXXXXXXX1111111111111111111111111111111111111111111111111111111111111111111111111111111111111111111111111111111111111111111111111111
1111111111111111111111111111111111111111111111111111111111111111111111111111111111111111111111111111111111111111111111111111111111111111110000000000000000000000000000000000000000
00000000000000000000000000000000000000000000000000000000000000000000000000000000000000000000000000000000000000000000000000000000000000000000000000000000000000000000000000000000
00000000000000000000000000000000000000000000000000000000000000000000000000000000000000000000000000000000000000000000000000000000000000000000000000000000
XXXXXXXXXXXXXXXXXXXXXXXXXXXXXXXXXXXXXXXXXXXXXXXXXXXXXXXXXXXXXXXXXXXXXXXXXXXXXXXXXXXXXXXXXXXXXXXXXXXXXXXXXXXXXXXXXXXXXXXXXXXXXXXXXXXXXXXXXXXXXXXXXXXXXXXXXXXXXXXXXXXXXXXXXX
XXXXXXXXXXXXXXXXXXXXXXXXXXXXXXXXXXXXXXXXXXXXXXXXXXXXXXXXXXXXXXXXXXXXXXXXXXXXXXXXXXXXXXXXXXXXXXXXXXXXXXXXXXXXXXXXXXXXXXXXXXXXXXXXXXXXXXXXXXXXXXXXXXXXXXXXXXXXXXXXXXXXXXXXXX
XXXXXXXXXXXXXXXXXXXXXXXXXXXXXXXXXXXXXXXXXXXXXXXXXXXXXXXXXXXXXXXXXXXXXXXXXXXXXXXXXXXXXXXXXXXXXXXXXXXXXXXXXXXXXXXXXXXXXXXXXXXXXXXXXXXXXXXXXXXXXXXXXXXXXXXXXXXXXXXXXXXXXXXX
XXXXXXXXXXXXXXXXXXXXXXXXXXXXXXXXXXXXXXXXXXXXXXXXXXXXXXXXXXXXXXXXXXXXXXXXXXXXXXXXXXXXXXXXXXXXXXXXXXXXXXXXXXXXXXXXXXXXXXXXXXXXXXXXXXXXXXXXXXXXXXXXXXXXXXXXXXXXXXXXXXXXXXXXX
XXXXXXXXXXXXXXXXXXXXXXXXXXXXXXXXXXXXXXXXXXXXXXXXXXXXXXXXXXXXXXXXXXXXXXXXXXXXXXXXXXXXXXXXXXXXXXXXXXXXXXXXXXXXXXXXXXXXXXXXXXXXXXXXXXXXXXXXXXXXXXXXXXXXXXXXXXXXXXXXXXXXXXXX
XXXXXXXXXXXXXXXXXXXXXXXXXXXXXXXXXXXXXXXXXXXXXXXXXXXXXXXXXXXXXXXXXXXXXXXXXXXXXXXXXXXXXXXXXXXXXXXXXXXXXXXXXXXXXXXXXXXXXXXXXXXXXXXXXXXXXXXXXXXXXXXXXXXXXXXXXXXXXXXXXXXXXXXX
XXXXXXXXXXXXXXXXXXXXXXXXXXXXXXXXXXXXXXXXXXXXXXXXXXXXXXXXXXXXXXXXXXXXXXXXXXXXXXXXXXXXXXXXXXXXXXXXXXXXXXXXXXXXXXXXXXXXXXXXXXXXXXXXXXXXXXXXXXXXXXXXXXXXXXXXXXXXXXXXXXXXXXXX
XXXXXXXXXXXXXXXXXXXXXXXXXXXXXXXXXXXXXXXXXXXXXXXXXXXXXXXXXXXXXXXXXXXXXXXXXXXXXXXXXXXXXXXXXXXXXXXXXXXXXXXXXXXXXXXXXXXXXXXXXXXXXXXXXXXXXXXXXXXXXXXXXXXXXXXXXXXXXXXXXXXXXXXX
XXXXXXXXXXXXXXXXXXXXXXXXXXXXXXXXXXXXXXXXXXXXXXXXXXXXXXXXXXXX0000000000000000000000000000000000000000000000000000000000000000000000000000000000000000000000000000000000000000000000000000
00000000000000000000000000000000000000000000000000000000000000000000000000000000000000000000000000000000000000000000000000000000000000000000000000000000000000000000000000000000
00000000000000000000000000000000000000000000000000000000000000000000000000000000000000000000000000000000000000000000000000000000000000000000000000000000000000000000000000000000
00000000000000000000000000000000000000000000000000000000000000000000000000000000000000000000000000000000000000000000000000000000000000000000000000000000
>Q9NRJ3
MQQRGLAIVALAVCAALHASEAILPIASSCCTEVSHHISRRLLERVNMCRIQRADGDCDLAAVILHVKRRRICVSPHNHTVKQWMKVQAAKKNGKGNVCHRKKHHGKRNSNRAHQGKHETYGHKTPY
1111111111111111111111111111111111111111111111111111111111111111111111111111111111111111111111111111111111111111111111111111111
0000000000000000000000000000000000000000000000000000000000000000000000000000000000000000000000000000000000000000000000000000000
0000000000000000000000000000000000000000000000000000000000000000000000000000000000000000000000000000000000000000000000000000000
>P52272
MAAGVEAAAEVAATEIKMEEESGAPGVPSGNGAPGPKGEGERPAQNEKRKEKNIKRGGNRFEPYANPTKRYRAFITNIPFDVKWQSLKDLVKEKVGEVTYVELLMDAEGKSRGCAVVEFKMEESMKKAAEVLNKHSLSGRPLKVKEDPDGEHARRAMQKVMA
TTGGMGMGPGGPGMITIPPSILNNPNIPNEIIHALQAGRLGSTVFVANLDYKVGWKKLKEVFSMAGVVVRADILEDKDGKSRGIGTVTFEQSIEAVQAISMFNGQLLFDRPMHVKMDERALPKGDFFPPERPQQLPHGLGGIGMGLGPGGQPIDANHLNKGIG
MGNIGPAGMGMEGIGFGINKMGGMEGPFGGGMENMGRFGSGMNMGRINEILSNALKRGEIIAKQGGGGGGGSVPGIERMGPGIDRLGGAGMERMGAGLGHGMDRVGSEIERMGLVMDRMGSVERMGSGIERMGPLGLDHMASSIERMGQTMERI
GSGVERMGAGMGFGLERMAAPIDRVGQTIERMGSGVERMGPAIERMGLSMERMVPAGMGAGLERMGPVMDRMATGLERMGANNLERMGLERMGANSLERMGLERMGANSLERMGPAMGPALGAGIERMGLAMGGGGGASFDRAIEMERGNFG
GSFAGSFGGAGGHAPGVARKACQIFVRNLPFDFTWKMLKDKFNECGHVLYADIKMENGKSKGCGVVKFESPEVAERACRMMNGMKLSGREIDVRIDRNA
XXXXXXXXXXXXXXXXXXXXXXXXXXXXXXXXXXXXXXXXX1111111111111111111111111111111100000000000000000000000000000000000000000000000000000000000000000000000000000000000000000000000000000000000
00000000000000000000000000000000000000000000000000000000000000000000000000000000000000000000000000000000000000000000000000000000000000000000000000000000000000000000000000000000
00000000000000000000000000000000000000000000000000000000000000000000000000000000000000000000000000000000000000000000000000000000000000000000000000000000000000000000000000000000
00000000000000000000000000000000000000000000000000000000000000000000000000000000000000000000000000000000000000000000000000000000000000000000000000000000000000000000000000000000
000000000000000000000000000

XXXXXXXXXXXXXXXXXXXXXXXXXXXXXXXXXXXXXXXX111111111111111111111111111111000000000000000000000000000000000000000000000000000000000000000000000000000000000000000000000000000000000000000000000000000000000000000000000000
0000000000000000000000000000000000000000000000000000000000000000000000000000000000000000000000000000000000000000000000000000000000000000000000000000000000000000000000000000000000000000000000
0000000000000000000000000000000000000000000000000000000000000000000000000000000000000000000000000000000000000000000000000000000000000000000000000000000000000000000000000000000000000000000000
0000000000000000000000000000000000000000000000000000000000000000000000000000000000000000000000000000000000000000000000000000000000000000000000000000000000000000000000000000000000000000000000
000000000000000000000000000
XXXXXXXXXXXXXXXXXXXXXXXXXXXXXXXXXXXXXXXX000000000000000000000000000000000000000000000000000000000000000000000000000000000000000000000000000000000000000000000000000000000000000000000000000000000000000000000000000000
0000000000000000000000000000000000000000000000000000000000000000000000000000000000000000000000000000000000000000000000000000000000000000000000000000000000000000000000000000000000000000000000
0000000000000000000000000000000000000000000000000000000000000000000000000000000000000000000000000000000000000000000000000000000000000000000000000000000000000000000000000000000000000000000000
0000000000000000000000000000000000000000000000000000000000000000000000000000000000000000000000000000000000000000000000000000000000000000000000000000000000000000000000000000000000000000000000
000000000000000000000000000
>P30429
MLCEIECRALSTAHTRLIHDFEPRDALTYLEGKNIFTEDHSELISKMSTRLERIANFLRIYRRQASELGPLIDFFNYNNQSHLADFLEDYIDFAINEPDLLRPVVIAPQFSRQMLDRKLLLGNVPKQMTCYIREYHVDRVIKKLDEMCDLDSFFLFLHGRAGSGKSVIASQAL
SKSDQLIGINYDSIVWLKDSGTAPKSTFDLFTDILLMLARVVSDTDDSHSITDFINRVLSRSEDDLLNFPSVEHVTSVVLKRMICNALIDRPNTLFVFDDVVQEETIRWAQELRLRCLVTTRDVEISNAASQTCEFIEVTSLEIDECYDFLEAYGMPMPVGEKEEDVLNKTI
ELSSGNPATLMMFFKSCEPKTFEKMAQLNNKLESRGLVGVECITPYSYKSLAMALQRCVEVLSDEDRSALAFAVVMPPGVDIPVKLWSCVIPVDICSNEEEQLDDEVADRLKRLSKRGALLSGKRMPVLTFKIDHIIHMFLKHVVDAQTIANGISILEQRLLEIGNNNV
SVPERHIPSHFQKFRRSSASEMYPKTTEETVIRPEDFPKFMQLHQKFYDSLKNFACC
1111111111111111111111111111111111111111111111111111111111111111111111111111111111111111111111111111111111111111111111111111111111111111000000000000000000000000000000000000000000000000000000
0000000000000000000000000000000000000000000000000000000000000000000000000000000000000000000000000000000000000000000000000000000000000000000000000000000000000000000000000000000000000000000000
0000000000000000000000000000000000000000000000000000000000000000000000000000000000000000000000000000000000000000000000000000000000000000000000000000000000000000000000000000000000000000000000
0000000000000000000000000000000000000000000000000000000000000000000000000000000000000000000000000000000000000000000000000000000000000000000000000000000000000000000000000000000000000000000000
00000000000000000000000000000000000000000000000
0000000000000000000000000000000000000000000000000000000000000000000000000000000000000000000000000000000000000000000000000000000000000000000000000000000000000000000000000000000000000000000000
0000000000000000000000000000000000000000000000000000000000000000000000000000000000000000000000000000000000000000000000000000000000000000000000000000000000000000000000000000000000000000000000
0000000000000000000000000000000000000000000000000000000000000000000000000000000000000000000000000000000000000000000000000000000000000000000000000000000000000000000000000000000000000000000000
0000000000000000000000000000000000000000000000000000000000000000000000000000000000000000000000000000000000000000000000000000000000000000000000000000000000000000000000000000000000000000000000
00000000000000000000000000000000000000000000000
0000000000000000000000000000000000000000000000000000000000000000000000000000000000000000000000000000000000000000000000000000000000000000000000000000000000000000000000000000000000000000000000
0000000000000000000000000000000000000000000000000000000000000000000000000000000000000000000000000000000000000000000000000000000000000000000000000000000000000000000000000000000000000000000000
0000000000000000000000000000000000000000000000000000000000000000000000000000000000000000000000000000000000000000000000000000000000000000000000000000000000000000000000000000000000000000000000
0000000000000000000000000000000000000000000000000000000000000000000000000000000000000000000000000000000000000000000000000000000000000000000000000000000000000000000000000000000000000000000000
00000000000000000000000000000000000000000000000
>O74774
MSRHRDVKNLDLDDYELDEEPGEEELTEEQEEEFRSAVATVRETLLGVPISEKEIADTVWYYYFDVEKSVNYLLQKASSKAGAKEKQNTDSQKEKKQNKSKEALADAKDPLDESSNGIKNLSLNKNDEPAFQTNGEVKMKNSSESDNQPEKKKIKKQNPTDLVSVPEI
FEQSNPKPVVHLVVTGHVDSGKSTMLGRIMFELGEINSRSMQKLHNEAANSGKGSFSYAWLLDTTEEERARGVTMDVASTTFESDKKIYEIGDAPGHRDFISGMIAGASSADFAVLVVDSSQNNFERGFLENGQTREHAYLLRALGISEIVVSVNKLDLMSWSEDR
FQEIKNIVSDFLIKMVGFKTSNVHFVPISAISGTNLIQKDSSDLYKWYKGPTLLSALDQLVPPEKPYRKPLRLSIDDVYRSPRSVTVTGRVEAGNVQVNQVLYDVSSQEDAYVKNVIRNSDPSSTWAVAGDTVTLQLADIEVNQLRPGDILSNYENPVRRVRSFVAEIQT
FDIHGPILSGSTLVLHLGRTVTSVSLKIVTVNNKRSRHIASRKRALVRISFLDGLFPLCLAEECPALGRFILRRSGDTVAAGIVKELC
1111111111111111111111111111111111111111111111111111111111111111111111111111111111111111111111111111111111111111111111111111111111111111111111111111111111111111111111100000000000000000000000
0000000000000000000001111111111111111111111111111111111111111111111111111111111111111111111111111111111111000000000000000000000000000000000000000000000000000000000000000000000000000000000000
0000000000000000000000000000000000000000000000000000000000000000000000000000000000000000000000000000000000000000000000000000000000000000000000000000000000000000000000000000000000000000000000
000000000000000000000000000000000000000000000000000000000000000000000
0000000000000000000000000000000000000000000000000000000000000000000000000000000000000000000000000000000000000000000000000000000000000000000000000000000000000000000000000000000000000000000000
0000000000000000000000000000000000000000000000000000000000000000000000000000000000000000000000000000000000000000000000000000000000000000000000000000000000000000000000000000000000000000000000
0000000000000000000000000000000000000000000000000000000000000000000000000000000000000000000000000000000000000000000000000000000000000000000000000000000000000000000000000000000000000000000000
000000000000000000000000000000000000000000000000000000000000000000000
0000000000000000000000000000000000000000000000000000000000000000000000000000000000000000000000000000000000000000000000000000000000000000000000000000000000000000000000000000000000000000000000
0000000000000000000000000000000000000000000000000000000000000000000000000000000000000000000000000000000000000000000000000000000000000000000000000000000000000000000000000000000000000000000000
0000000000000000000000000000000000000000000000000000000000000000000000000000000000000000000000000000000000000000000000000000000000000000000000000000000000000000000000000000000000000000000000
000000000000000000000000000000000000000000000000000000000000000000000
>P41814
MNALTTIDFNQHVIVRLPSKNYKIVELKPNTSVSLGKFGAFEVNDIIGYPFGLTFEIYYDGEEVSSDENRDSKPKNKIPIGKVRLLSQEIKDVNNDKDDGQSEPPLSIKEKSVSLELSSIDSSATNQNLVNMGSKAQELTVEEIEKMKQESLSSKEIIDKIIKSHKSFHNKTVYS
QEKYVNRKKQKFAKYFTVEYLSSSNLLQFLIDKGDIQRVLDMSQESMGMLLNLANIQSEGNYLCMDETGGLLVYFLLERMFGGDNESKSKGKVIVIHENEHANLDLLKFANYSEKFIKEHVHTISLLDFFEPPTLQEIQSRFTPLPKEEARALKGGKKNSYYRKLRWYNT
QWQILELTGEFLYDGLVMATTLHLPTLVPKLAEKIHGSRPIVCYGQFKETLLELAHTLYSDLRFLAPSILETRCRPYQSIRGKLHPLMTMKGGGGYLMWCHRVIPAPEPVSENATAADSSEKLAEHGAKKQKI
0000000000000000000000000000000000000000000000000000000000000000000000000000000000000011111111111111111XXXXXXXXX1111111111111111111111111111111111111111111111111111111111111111111111111111
1111111111110000000000000000000000000000000000000000000000000000000000000000000000000000000000000000000000000000000000000000000000000000000000000000000000000000000000000000000000000000000000
0000000000000000000000000000000000000000000000000000000000000000000000000000000000000000000000000000000000000000001111111111111111111111111
000000000000000000000000000000000000000000000000000000000000000000000000000000000000XXXXXXXXX0000000000000000000000000000000000000000000000000000000000000000000000000000000000000000000000000000
0000000000000000000000000000000000000000000000000000000000000000000000000000000000000000000000000000000000000000000000000000000000000000000000000000000000000000000000000000000000000000000000
0000000000000000000000000000000000000000000000000000000000000000000000000000000000000000000000000000000000000000000000000000000000000000
000000000000000000000000000000000000000000000000000000000000000000000000000000000000XXXXXXXXX0000000000000000000000000000000000000000000000000000000000000000000000000000000000000000000000000000
0000000000000000000000000000000000000000000000000000000000000000000000000000000000000000000000000000000000000000000000000000000000000000000000000000000000000000000000000000000000000000000000
0000000000000000000000000000000000000000000000000000000000000000000000000000000000000000000000000000000000000000000000000000000000000000
>P04383
MENKGEKIAMNPTVQTLAQKGDKLAVKLVTRGWASLSTNQKRRAEMLAGYTPAILAFTPRRPRMTNPPPRTSRNSPGQAGKSMTMSKTELLSTVKGTTGVIPSFEDWVVSPRNVAVFPQLSLLATNFNKYRITALTVKYSPACSFETNGRVALGFNDDASDTPPT
TKVGFYDLGKHVETAAQTAKDLVIPVDGKTRFIRDSASDDAKLVDFGRIVLSTYGFDKADTVVGELFIQYTIVLSDPTKTAKISQASNDKVSDGPTYVVPSVNGNELQLRVVAAGKWCIIVRGTVEGGFTKPTLIGPGISGDVDYESARPIAVCELVTQMEGQILKITKTS
AEQPLQWVVYRM
1111111111111111111111111111111111111111111111111111111111111111111111111111111111111111111111111111111111111111000000000000000000000000000000000000000000000000000000000000000000000000000000
00000000000000000000000000000000000000000000000000000000000000000000000000000000000000000000000000000000000000000000000000000000000000000000000000000000000000000000000000000000000000000
0000000000000000000000000000000000000000000000000000000000000000000000000000000000000000000000000000000000000000000000000000000000000000000000000000000000000000000000000000000000000000000000
00000000000000000000000000000000000000000000000000000000000000000000000000000000000000000000000000000000000000000000000000000000000000000000000000000000000000000000000000000000000000000
0000000000000000000000000000000000000000000000000000000000000000000000000000000000000000000000000000000000000000000000000000000000000000000000000000000000000000000000000000000000000000000000
00000000000000000000000000000000000000000000000000000000000000000000000000000000000000000000000000000000000000000000000000000000000000000000000000000000000000000000000000000000000000000
>Q9JPP1
MFERSVIAMACIFALSACGGGGGGSPDVKSADTLSKPAAPVVAEKETEVKEDAPQAGSQGQGAPSTQGSQDMAAVSAENTGNGGAATTDKPKNEDEGPQNDMLQNSAESANQTGNNQPADSSDSAPASNPAPANGGSNFGRVDLANGVLIDGPSQNITLT
HCKGDSCNGDNLLDEEAPSKSEFENLNESERIEKYKKDGKSDKFTNLVATAVQANGTNKYVIIYKDKSASSSFARFRRSARSRRSLPAEMPLIPVNQADTLIVDGEAVSLTGHSGNIFAPEGNYRYLTYGAEKLPGGSYALRVQGEPAKGEMLAGTAVYNGEVLHFHT
ENGRPYPTRGRFAAKVDFGSKSVDGIIDSGDDLHMGTQKFKAAIDGNGFKGTWTENGGGDVSGRFYGPAGEEVAGKYSYRPTDAEKGGFGVFAGKKEQD
XXXXXXXXXXXXXXXXXXXXXXXXXXXXXXXXXXXXXXXXXXXXXXXXXXXXXXXXXXXXXXXXXXXXXXXXXXXXXXXXXXXXXXXXXXXXXXXXXXXXXXXXXXXXXXXXXXXXXXXXXXXXXXXXXXXXXXXXXX11111111111111111111111111111111111111111111111111
1111111111111111111111111111111111111111111111111111111111111111111111111111111111111111111111111111111111111111111111111111111111111111111111100000000000000000000000000000000000000000000000
000000000000000000000000000000000000000000000000000000000000000000000000000000000000
XXXXXXXXXXXXXXXXXXXXXXXXXXXXXXXXXXXXXXXXXXXXXXXXXXXXXXXXXXXXXXXXXXXXXXXXXXXXXXXXXXXXXXXXXXXXXXXXXXXXXXXXXXXXXXXXXXXXXXXXXXXXXXXXXXXXXXXXXXXXXXXXXX000000000000000000000000000000000000000000000
0000000000000000000000000000000000000000000000000000000000000000000000000000000000000000000000000000000000000000000000000000000000000000000000000000000000000000000000000000000000000000000000
000000000000000000000000000000000000000000000000000000000000000000000000000000000000
XXXXXXXXXXXXXXXXXXXXXXXXXXXXXXXXXXXXXXXXXXXXXXXXXXXXXXXXXXXXXXXXXXXXXXXXXXXXXXXXXXXXXXXXXXXXXXXXXXXXXXXXXXXXXXXXXXXXXXXXXXXXXXXXXXXXXXXXXXXXXXXXXX000000000000000000000000000000000000000000000
0000000000000000000000000000000000000000000000000000000000000000000000000000000000000000000000000000000000000000000000000000000000000000000000000000000000000000000000000000000000000000000000
000000000000000000000000000000000000000000000000000000000000000000000000000000000000
>P27472
MSRDLQNHLLFETATEVANRVGGIYSVLKSKAPITVAQYKDHYHLIGPLNKATYQNEVDILDWKKPEAFSDEMRPVQHALQTMESRGVHFVYGRWLIEGAPKVILFDLDSVRGYSNEWKGDLWSLVGIPSPENDFETNDAILLGYTVAWFLGEVAHLDSQHAIVA
HFHEWLAGVALPLCRKRRIDVVTIFTTHATLLGRYLCASGSFDFYNCLESVDVDHEAGRFGIYHRYCIERAAAHSADVFTTVSQITAFEAEHLLKRKPDGILPNGLNVIKFQAFHEFQNLHALKKEKINDFVRGHFHGCFDFDLDNTLYFFIAGRYEYKNKGADMFIEAL
ARLNYRLKVSGSKKTVVAFIVMPAKNNSFTVEALKGQAEVRALENTVHEVTTSIGKRIFDHAIRYPHNGLTTELPTDLGELLKSSDKVMLKRRILALRRPEGQLPPIVTHNMVDDANDLILNKIRQVQLFNSPSDRVKMIFHPEFLNANNPILGLDYDEFVRGCHLGVFP
SYYEPWGYTPAECTVMGVPSITTNVSGFGAYMEDLIETNQAKDYGIYIVDRRFKAPDESVEQLVDYMEEFVKKTRRQRINQRNRTERLSDLLDWKRMGLEYVKARQLALRRGYPDQFRELVGEELNDSNMDALAGGKKLKVARPLSVPGSPRDLRSNSTVYMTPG
DLGTLQEVNNADDYFSLGVNPAADDDDDGPYADDS
0000000000000000000000000000000000000000000000000000000000000000000000000000000000000000000000000000000000000000000000000000000000000000000000000000000000000000000000000000000000000000000000
0000000000000000000000000000000000000000000000000000000000000000000000000000000000000000000000000000000000000000000000000000000000000000000000000000000000000000000000000000000000000000000000
0000000000000000000000000000000000000000000000000000011111111111000000000000000000000000000000000000000000000000000000000000000000000000000000000000000000000000000000000000000000000000000000
0000000000000000000000000000000000000000000000000000000000000000001111111111111111111111111111111111111111111111111111111111111111111111111111111111111111111111111111111111111111111111111111
1
0000000000000000000000000000000000000000000000000000000000000000000000000000000000000000000000000000000000000000000000000000000000000000000000000000000000000000000000000000000000000000000000
0000000000000000000000000000000000000000000000000000000000000000000000000000000000000000000000000000000000000000000000000000000000000000000000000000000000000000000000000000000000000000000000
0000000000000000000000000000000000000000000000000000000000000000000000000000000000000000000000000000000000000000000000000000000000000000000000000000000000000000000000000000000000000000000000
0000000000000000000000000000000000000000000000000000000000000000000000000000000000000000000000000000000000000000000000000000000000000000000000000000000000000000000000000000000000000000000000
0
0000000000000000000000000000000000000000000000000000000000000000000000000000000000000000000000000000000000000000000000000000000000000000000000000000000000000000000000000000000000000000000000
0000000000000000000000000000000000000000000000000000000000000000000000000000000000000000000000000000000000000000000000000000000000000000000000000000000000000000000000000000000000000000000000
0000000000000000000000000000000000000000000000000000000000000000000000000000000000000000000000000000000000000000000000000000000000000000000000000000000000000000000000000000000000000000000000
0000000000000000000000000000000000000000000000000000000000000000000000000000000000000000000000000000000000000000000000000000000000000000000000000000000000000000000000000000000000000000000000
0
>O94316
MMEEDLYDEFGNYIGPENEEDEEELFPQAPSPTIAQVPSFEEVIPDEELEDVERAEEMALSHLEPQNAVVLHEDKQYYPSAEEVYGSNVDIMVQEQDTQPLSQPIIEPIRHKRIAIETTNVPDTVYKKEFLFGLLTGTDDVRSFIVAGHLHHGKSALLDLLVYYTHPDTK
PPKRRSLRYTDTHYLERERVMSIKSTPLTLAVSDMKGKTFAFQCIDTPGHVDFVDEVAAPMAISDGVVLVVDVIEGVMINTTRIIKHAILHDMPIVLVLNKVDRLILELRLPPNDAYHKLRHVIDEVNDNICQISKDLKYRVSPELGNVCFASCDLGYCFTLSSFAKLYIDR
HGGIDVDLFSKRLWGDIYFDSKTRKFAKQSLDGSGVRSFVHFILEPLYKLHTLTISDEAEKLKKHLSSFQIYLKPKDYLLDPKPLLQLICASFFGFPVGFVNAVTRHIPSPRENAARKASQSYIGPINSSIGKAILEMSREESAPLVMHVTKLYNTVDANNFYAFARVYSGQV
KKGQKVKVLGENYSLEDEEDMVVAHIAEICVPCARYRLHVDGAVAGMLVLLGGVDNSISKTATIVSDNLKDDPYIFRPIAHMSESVFKVAVEPHNPSELPKLLDGLRKTNKSYPLSITKVEESGEHTIFGTGEMYMDCLLYDLRTLYSEIEIRVSDPVARFCETAVDTSSIK
CFSDTPNKKNRITMVVEPLEKGISNDIENGKVNINWPQKRISEFFQKNYDWDLLASRSIWAFGPDDRGTNILRDDTLSTDVDKNVLNSVKEYIKQGFQWGTREGPLCDETIRNVNFRLMDVVLAPEQIYRGGGQIIPTARRVCYSSFLTASPRLMEPVYMVEVHAP
ADSLPIIYDLLTRRRGHVLQDIPRPGSPLYLVRALIPVIDSCGFETDLRVHTQGQAMCQMVFDHWQVVPGDPLDKSIKPKPLEPARGSDLARDFLIKTRRRKGLVEDVSTTRYFDQEMIDSLKEAGVVLSL
1111111111111111111111111111111111111111111111111111111111111111111111111111111111111111111111111100000000000000000000000000000000000000000000000000000000000000000000000000000000000000000000
0000000000000000000000000000000000000000000000000000000000000000000000000000000000000000000000000000000000000000000000000000000000000000000000000000000000000000000000000000000000000000000000
0000000000000000000000000000000000000000000000000000000000000000000000000000000000000000000000000000000000000000000000000000000000000000000000000000000000000000000000000000000000000000000000
0000000000000000000000000000000000000000000000000000000000000000000000000000000000000000000000000000000000000000000000000000000000000000000000000000000000000000000000000000000000000000000000
0000000000000000000000000000000000000000000000000000000000000000000000000000000000000000000000000000000000000000000000000000000000000000000000000000000000000000000000000000000000000000000000
0000000000000000000000000000000000000000000000000000000000000000000000000000000000000000000000000000000000000000000000000000000000000000000000000000000000000000000000000000000000000000000000
0000000000000000000000000000000000000000000000000000000000000000000000000000000000000000000000000000000000000000
0000000000000000000000000000000000000000000000000000000000000000000000000000000000000000000000000000000000000000000000000000000000000000000000000000000000000000000000000000000000000000000000
0000000000000000000000000000000000000000000000000000000000000000000000000000000000000000000000000000000000000000000000000000000000000000000000000000000000000000000000000000000000000000000000
0000000000000000000000000000000000000000000000000000000000000000000000000000000000000000000000000000000000000000000000000000000000000000000000000000000000000000000000000000000000000000000000
0000000000000000000000000000000000000000000000000000000000000000000000000000000000000000000000000000000000000000000000000000000000000000000000000000000000000000000000000000000000000000000000
0000000000000000000000000000000000000000000000000000000000000000000000000000000000000000000000000000000000000000000000000000000000000000000000000000000000000000000000000000000000000000000000
0000000000000000000000000000000000000000000000000000000000000000000000000000000000000000000000000000000000000000000000000000000000000000000000000000000000000000000000000000000000000000000000
0000000000000000000000000000000000000000000000000000000000000000000000000000000000000000000000000000000000000000
0000000000000000000000000000000000000000000000000000000000000000000000000000000000000000000000000000000000000000000000000000000000000000000000000000000000000000000000000000000000000000000000
0000000000000000000000000000000000000000000000000000000000000000000000000000000000000000000000000000000000000000000000000000000000000000000000000000000000000000000000000000000000000000000000
0000000000000000000000000000000000000000000000000000000000000000000000000000000000000000000000000000000000000000000000000000000000000000000000000000000000000000000000000000000000000000000000
0000000000000000000000000000000000000000000000000000000000000000000000000000000000000000000000000000000000000000000000000000000000000000000000000000000000000000000000000000000000000000000000
0000000000000000000000000000000000000000000000000000000000000000000000000000000000000000000000000000000000000000000000000000000000000000000000000000000000000000000000000000000000000000000000
0000000000000000000000000000000000000000000000000000000000000000000000000000000000000000000000000000000000000000000000000000000000000000000000000000000000000000000000000000000000000000000000
0000000000000000000000000000000000000000000000000000000000000000000000000000000000000000000000000000000000000000
>A0A045GWT8
MAQEQTKRGGGGGDDDDIAGSTAAGQERREKLTEETDDLLDEIDDVLEENAEDFVRAYVQKGGQ

11111111111111111111111111111111111111111111111111111111111XXXXX
00000000000000000000011111111111111111111111111111111111111XXXXX
00000000000000000000000000000000000000000000000000000000000XXXXX
>Q02199
MFGLNKASSTPAGGLFGQASGASTGNANTGFSFGGTQTGQNTGPSTGGLFGAKPAGSTGGLGASFGQQQQQSQTNAFGGSATTGGGLFGNKPNNTANTGGGLFGANSNSNSGSLFGSNNAQTSRGLFGNNNTNNINNSSSGMNNASAGLFGSKPAGGTSL
FGNTSTSSAPAQNQGMFGAKPAGTSLFGNNAGNTTTGGGLFGSKPTGATSLFGSSNNNNNNNNSNNIMSASGGLFGNQQQQLQQQPQMQCALQNLSQLPITPMTRISELPPQIRQEIEQLDQYIQKQVQISHHLKADTIDHDELIDSIPRDVAYLLKSESATSQ
YLKQDLKKISSFKSLIDEDLLDTQTFSVLLQQLLTPGSKISSNDLDKFFQKKIHLYEKKLEDYCRILSDIETAVNGIDTDLFGAPNNPNSTAITADLGSSEAENLLQLKTGLAAIVSTVIEEFTLFMDIAERIAVLHQKTKTLASLSI
XXXXXXXXXXXXXXXXXXXXXXXXXXXXXXXXXXXXXXXXXXXXXXXXXXXXXXXXXXXXXXXXXXXXXXXXXXXXXXXXXXXXXXXXXXXXXXXXXXXXXXXXXXXXXXXXXXXXXXXX111111111111111111111111111111111XXXXXXXXXXXXXXXXXXX
XXXXXXXXXXXXXXXXXXXXXXXXXXXXXXXXXXXXXXXXXXXXXXXXXXXXXXXXXXXXXXXXXXXXXXXXXXXXXXXXXXXXXXXXXXXXXXXXXXXXXXXXXXXXXXXXXXXXXXXXXXXXXXXXXXXXXXXXXXXXXXXXXXXXXXXXXXXXXXXXXXXXXXXXXX
XXXXXXXXXXXXXXXXXXXXXXXXXXXXXXXXXXXXXXXXXXXXXXXXXXXXXXXXXXXXXXXXXXXXXXXXXXXXXXXXXXXXXXXXXXXXXXXXXXXXXXXXXXXXXXXXXXXXXXXXXXXXXXXX
XXXXXXXXXXXXXXXXXXXXXXXXXXXXXXXXXXXXXXXXXXXXXXXXXXXXXXXXXXXXXXXXXXXXXXXXXXXXXXXXXXXXXXXXXXXXXXXXXXXXXXXXXXXXXXXXXXXXXXXX000000000000000000000000000000000XXXXXXXXXXXXXXXXXXX
XXXXXXXXXXXXXXXXXXXXXXXXXXXXXXXXXXXXXXXXXXXXXXXXXXXXXXXXXXXXXXXXXXXXXXXXXXXXXXXXXXXXXXXXXXXXXXXXXXXXXXXXXXXXXXXXXXXXXXXXXXXXXXXXXXXXXXXXXXXXXXXXXXXXXXXXXXXXXXXXXXXXXXXXXXXX
XXXXXXXXXXXXXXXXXXXXXXXXXXXXXXXXXXXXXXXXXXXXXXXXXXXXXXXXXXXXXXXXXXXXXXXXXXXXXXXXXXXXXXXXXXXXXXXXXXXXXXXXXXXXXXXXXXXXXXXXXXXXXXXX
XXXXXXXXXXXXXXXXXXXXXXXXXXXXXXXXXXXXXXXXXXXXXXXXXXXXXXXXXXXXXXXXXXXXXXXXXXXXXXXXXXXXXXXXXXXXXXXXXXXXXXXXXXXXXXXXXXXXXXXX000000000000000000000000000000000XXXXXXXXXXXXXXXXXXX
XXXXXXXXXXXXXXXXXXXXXXXXXXXXXXXXXXXXXXXXXXXXXXXXXXXXXXXXXXXXXXXXXXXXXXXXXXXXXXXXXXXXXXXXXXXXXXXXXXXXXXXXXXXXXXXXXXXXXXXXXXXXXXXXXXXXXXXXXXXXXXXXXXXXXXXXXXXXXXXXXXXXXXXXXXXX
XXXXXXXXXXXXXXXXXXXXXXXXXXXXXXXXXXXXXXXXXXXXXXXXXXXXXXXXXXXXXXXXXXXXXXXXXXXXXXXXXXXXXXXXXXXXXXXXXXXXXXXXXXXXXXXXXXXXXXXXXXXXXXXX
>Q03281
MDHRNLDPKTLKVSQLRRVLVENDVAFPANARKPVLVKLFEEKVRQRLQSSPEASKVRTSIQKVVKSGAKNADRKKTLKSKKLESSSSESKTVKDENVETNKRKREQISTDNEAKMQIQEEKSPKKKRKKRSSKANKPPESPPQSKSDGKATSADLTSELETVEELHKK
DSSDDKPRVKELPKPELPNLKVSNEFLAQLNKELASAATENYDHSIKSTDLSSIRIETEEPVGPSTGAETRNESEVMENINLEVQPEVKEAKEELTKISETFDNQDEEDTSRLSSKKNIRSPKGRTRHFIANKTKRGIDIMKPFIAHLFIWLWNGAIFLSIICPILFGLWYREQ
RIQVGYCGHEKPLKSLAISAFPQTERVDSVLQAYRPNCLECPEHGICSSFMNVECEPGYEPKSSILETYGIIPFPKYCAKDESKEKEVDELVWKVNEYLKKKNAQHECGEGENLFESGETETKLYDIFSHSRPSWESQREFNDHWKNVLEILKKKDDIIWLPLDFETNGKR
EKSKSNNTNYIYRSTSKKWVTLQCHLEGDIQEYITKYGGSLFITLGVLFLIKKIQSTLDNYVQGEQIIEKLVKEAIDKLKDVKKNKGEEPFLTTVQLRATLLSDIPNIKEQNNLWAQTKEKIMKEQSENIELYLLEENGEIMTCWEWKE
XXXXXXXXXXXXXXXXXXXXXXXXXXXXXXXXXXXXXXXXXXXXXXXXXXXXXXXXXXXXXXXXXXXXXXXXXXXXXXXXXXXXXXXXXXXXXXXXXX111111111111111111111111111111111111111111XXXXXXXXXXXXXXXXXXXXXXXXXXXXXXXX
XXXXXXXXXXXXXXXXXXXXXXXXXXXXXXXXXXXXXXXXXXXXXXXXXXXXXXXXXXXXXXXXXXXXXXXXXXXXXXXXXXXXXXXXXXXXXXXXXXXXXXXXXXXXXXXXXXXXXXXXXXXXXXXXXXXXXXXXXXXXXXXXXXXXXXXXXXXXXXXXXXXXXXXXXXX
XXXXXXXXXXXXXXXXXXXXXXXXXXXXXXXXXXXXXXXXXXXXXXXXXXXXXXXXXXXXXXXXXXXXXXXXXXXXXXXXXXXXXXXXXXXXXXXXXXXXXXXXXXXXXXXXXXXXXXXXXXXXXXXXXXXXXXXXXXXXXXXXXXXXXXXXXXXXXXXXXXXXXXXXXXXX
XXXXXXXXXXXXXXXXXXXXXXXXXXXXXXXXXXXXXXXXXXXXXXXXXXXXXXXXXXXXXXXXXXXXXXXXXXXXXXXXXXXXXXXXXXXXXXXXXXXXXXXXXXXXXXXXXXXXXXXXXXXXXXXXXXXXXXXXXXXXXXXXXXX
XXXXXXXXXXXXXXXXXXXXXXXXXXXXXXXXXXXXXXXXXXXXXXXXXXXXXXXXXXXXXXXXXXXXXXXXXXXXXXXXXXXXXXXXXXXXXXXXXX000000000000000000000000000000000000000000XXXXXXXXXXXXXXXXXXXXXXXXXXXXXXXX
XXXXXXXXXXXXXXXXXXXXXXXXXXXXXXXXXXXXXXXXXXXXXXXXXXXXXXXXXXXXXXXXXXXXXXXXXXXXXXXXXXXXXXXXXXXXXXXXXXXXXXXXXXXXXXXXXXXXXXXXXXXXXXXXXXXXXXXXXXXXXXXXXXXXXXXXXXXXXXXXXXXXXXXXXXXX
XXXXXXXXXXXXXXXXXXXXXXXXXXXXXXXXXXXXXXXXXXXXXXXXXXXXXXXXXXXXXXXXXXXXXXXXXXXXXXXXXXXXXXXXXXXXXXXXXXXXXXXXXXXXXXXXXXXXXXXXXXXXXXXXXXXXXXXXXXXXXXXXXXXXXXXXXXXXXXXXXXXXXXXXXXXX
XXXXXXXXXXXXXXXXXXXXXXXXXXXXXXXXXXXXXXXXXXXXXXXXXXXXXXXXXXXXXXXXXXXXXXXXXXXXXXXXXXXXXXXXXXXXXXXXXXXXXXXXXXXXXXXXXXXXXXXXXXXXXXXXXXXXXXXXXXXXXXXXXXX
XXXXXXXXXXXXXXXXXXXXXXXXXXXXXXXXXXXXXXXXXXXXXXXXXXXXXXXXXXXXXXXXXXXXXXXXXXXXXXXXXXXXXXXXXXXXXXXXXX000000000000000000000000000000000000000000XXXXXXXXXXXXXXXXXXXXXXXXXXXXXXXX
XXXXXXXXXXXXXXXXXXXXXXXXXXXXXXXXXXXXXXXXXXXXXXXXXXXXXXXXXXXXXXXXXXXXXXXXXXXXXXXXXXXXXXXXXXXXXXXXXXXXXXXXXXXXXXXXXXXXXXXXXXXXXXXXXXXXXXXXXXXXXXXXXXXXXXXXXXXXXXXXXXXXXXXXXXXX
XXXXXXXXXXXXXXXXXXXXXXXXXXXXXXXXXXXXXXXXXXXXXXXXXXXXXXXXXXXXXXXXXXXXXXXXXXXXXXXXXXXXXXXXXXXXXXXXXXXXXXXXXXXXXXXXXXXXXXXXXXXXXXXXXXXXXXXXXXXXXXXXXXXXXXXXXXXXXXXXXXXXXXXXXXXX
XXXXXXXXXXXXXXXXXXXXXXXXXXXXXXXXXXXXXXXXXXXXXXXXXXXXXXXXXXXXXXXXXXXXXXXXXXXXXXXXXXXXXXXXXXXXXXXXXXXXXXXXXXXXXXXXXXXXXXXXXXXXXXXXXXXXXXXXXXXXXXXXXXX
>P06437
MHQGAPSWGRRWFVVWALLGLTLGVLVASAAPTSPGTPGVAAATQAANGGPATPAPPPLGAAPTGDPKPKKNKKPKNPTPPRPAGDNATVAAGHATLREHLRDIKAENTDANFYVCPPPTGATVVQFEQPRRCPTRPEGQNYTEGIAVVFKENIAPYKFKAT
MYYKDVTVSQVWFGHRYSQFMGIFEDRAPVPFEEVIDKINAKGVCRSTAKYVRNNLETTAFHRDDHETDMELKPANAATRTSRGWHTTDLKYNPSRVEAFHRYGTTVNCIVEEVDARSVYPYDEFVLATGDFVYMSPFYGYREGSHTEHTTYAADRFKQVDGFY
ARDLTTKARATAPTTRNLLTTPKFTVAWDWVPKRPSVCTMTKWQEVDEMLRSEYGGSFRFSSDAISTTFTTNLTEYPLSRVDLGDCIGKDARDAMDRIFARRYNATHIKVGQPQYYQANGGFLIAYQPLLSNTLAELYVREHLREQSRKPPNPTPPPPGASANASVE
RIKTTSSIEFARLQFTYNHIQRHVNDMLGRVAIAWCELQNHELTLWNEARKLNPNAIASVTVGRRVSARMLGDVMAVSTCVPVAADNVIVQNSMRISSRPGACYSRPLVSFRYEDQGPLVEGQLGENNELRLTRDAIEPCTVGHRRYFTFGGGYVYFEEYAYSHQL
SRADITTVSTFIDLNITMLEDHEFVPLEVYTRHEIKDSGLLDYTEVQRRNQLHDLRFADIDTVIHADANAAMFAGLGAFFEGMGDLGRAVGKVVMGIVGGVVSAVSGVSSFMSNPFGALAVGLLVLAGLAAAFFAFRYVMRLQSNPMKALYPLTTKELKNPTNPDA
SGEGEEGGDFDEAKLAEAREMIRYMALVSAMERTEHKAKKKGTSALLSAKVTDMVMRKRRNTNYTQVPNKDGDADEDDL
XXXXXXXXXXXXXXXXXXXXXXXXXXXXXX111111111111111111111111111111111111111111111111111111111111111111111111111111000000000000000000000000000000000000000000000000000000000000000000
00000000000000000000000000000000000000000000000000000000000000000000000000000000000000000000000000000000000000000000000000000000000000000000000000000000000000000000000000000000
000000000000000000000000000000000000000000000000000000000000000000000000000000000000000000000000000000000000011111111111111111111111111111111110000000000000000000000000000000000
00000000000000000000000000000000000000000000000000000000000000000000000000000000000000000000000000000000000000000000000000000000000000000000000000000000000000000000000000000000
00000000000000000000000000000000000000000000000000000000000000000000000000000000000000000000000000000000000000000000000000000000000000000000000000000000000000000000000000000000
000000000000000000000000
XXXXXXXXXXXXXXXXXXXXXXXXXXXXXX00000000000000000000000000000000000000000000000000000000000000000000000000000000000000000000000000000000000000000000000000000000000000000000000000
00000000000000000000000000000000000000000000000000000000000000000000000000000000000000000000000000000000000000000000000000000000000000000000000000000000000000000000000000000000
00000000000000000000000000000000000000000000000000000000000000000000000000000000000000000000000000000000000000000000000000000000000000000000000000000000000000000000000000000000
00000000000000000000000000000000000000000000000000000000000000000000000000000000000000000000000000000000000000000000000000000000000000000000000000000000000000000000000000000000
00000000000000000000000000000000000000000000000000000000000000000000000000000000000000000000000000000000000000000000000000000000000000000000000000000000000000000000000000000000
000000000000000000000000
XXXXXXXXXXXXXXXXXXXXXXXXXXXXXX00000000000000000000000000000000000000000000000000000000000000000000000000000000000000000000000000000000000000000000000000000000000000000000000000
00000000000000000000000000000000000000000000000000000000000000000000000000000000000000000000000000000000000000000000000000000000000000000000000000000000000000000000000000000000
00000000000000000000000000000000000000000000000000000000000000000000000000000000000000000000000000000000000000000000000000000000000000000000000000000000000000000000000000000000
00000000000000000000000000000000000000000000000000000000000000000000000000000000000000000000000000000000000000000000000000000000000000000000000000000000000000000000000000000000
00000000000000000000000000000000000000000000000000000000000000000000000000000000000000000000000000000000000000000000000000000000000000000000000000000000000000000000000000000000
000000000000000000000000
>Q2TAZ0
MSRWLWPWSNCVKERVCRYLLHHYLGHFFQEHLSLDQLSLDLYKGSVALRDIHLEIWSVNEVLESMESPLELVEGFVGSIEVAVPWAALLTDHCTVRVSGLQLTLQPRRGPAPGAADSQSWASCMTTSLQLAQECLRDGLPEPSEPPQPLEGLEMFAQTIETVLRR
IKVTFLDTVVRVEHSPGDGERGVAVEVRVQRLEYCDEAVRDPSQAPPVDVHQPPAFLHKLLQLAGVRLHYEELPAQEEPPEPPLQIGSCSGYMELMVKLKQNEAFPGPKLEVAGQLGSLHLLLTPRQLQQLQELLSAVSLTDHEGLADKLNKSRPLGAEDLWLIEQD
LNQQLQAGAVAEPLSPDPLTNPLLNLDNTDLFFSMAGLTSSVASALSELSLSDVDLASSVRSDMASRRLSAQAHPAGKMAPNPLLDTMRPDSLLKMTLGGVTLTLLQTSAPSSGPPDLATHFFTEFDATKDGPFGSRDFHHLRPRFQRACPCSHVRLTGTAVQLSW
ELRTGSRGRRTTSMEVHFGQLEVLECLWPRGTSEPEYTEILTFPGTLGSQASARPCAHLRHTQILRRVPKSRPRRSVACHCHSELALDLANFQADVELGALDRLAALLRLATVPAEPPAGLLTEPLPAMEQQTVFRLSAPRATLRLRFPIADLRPEPDPWAGQAVRAE
QLRLELSEPQFRSELSSGPGPPVPTHLELTCSDLHGIYEDGGKPPVPCLRVSKALDPKSTGRKYFLPQVVVTVNPQSSSTQWEVAPEKGEELELSVESPCELREPEPSPFSSKRTMYETEEMVIPGDPEEMRTFQSRTLALSRCSLEVILPSVHIFLPSKEVYESIYNRINND
LLMWEPADLLPTPDPAAQPSGFPGPSGFWHDSFKMCKSAFKLANCFDLTPDSDSDDEDAHFFSVGASGGPQAAAPEAPSLHLQSTFSTLVTVLKGRITALCETKDEGGKRLEAVHGELVLDMEHGTLFSVSQYCGQPGLGYFCLEAEKATLYHRAAVDDYPLPSHL
DLPSFAPPAQLAPTIYPSEEGVTERGASGRKGQGRGPHMLSTAVRIHLDPHKNVKEFLVTLRLHKATLRHYMALPEQSWHSQLLEFLDVLDDPVLGYLPPTVITILHTHLFSCSVDYRPLYLPVRVLITAETFTLSSNIIMDTSTFLLRFILDDSALYLSDKCEVETLDLRRD
YVCVLDVDLLELVIKTWKGSTEGKLSQPLFELRCSNNVVHVHSCADSCALLVNLLQYVMSTGDLHPPPRPPSPTEIAGQKLSESPASLPSCPPVETALINQRDLADALLDTERSLRELAQPSGGHLPQASPISVYLFPGERSGAPPPSPPVGGPAGSLGSCSEEKEDEREE
EGDGDTLDSDEFCILDAPGLGIPPRDGEPVVTQLHPGPIVVRDGYFSRPIGSTDLLRAPAHFPVPSTRVVLREVSLVWHLYGGRDFGPHPGHRARTGLSGPRSSPSRCSGPNRPQNSWRTQGGSGRQHHVLMEIQLSKVSFQHEVYPAEPATGPAAPSQELEERPL
SRQVFIVQELEVRDRLASSQINKFLYLHTSERMPRRAHSNMLTIKALHVAPTTNLGGPECCLRVSLMPLRLNVDQDALFFLKDFFTSLVAGINPVVPGETSAEARPETRAQPSSPLEGQAEGVETTGSQEAPGGGHSPSPPDQQPIYFREFRFTSEVPIWLDYHGKHV
TMDQVGTFAGLLIGLAQLNCSELKLKRLCCRHGLLGVDKVLGYALNEWLQDIRKNQLPGLLGGVGPMHSVVQLFQGFRDLLWLPIEQYRKDGRLMRGLQRGAASFGSSTASAALELSNRLVQAIQATAETVYDILSPAAPVSRSLQDKRSARRLRRGQQPADLREG
VAKAYDTVREGILDTAQTICDVASRGHEQKGLTGAVGGVIRQLPPTVVKPLILATEATSSLLGGMRNQIVPDAHKDHALKWRSDSAQD
XXXXXXXXXXXXXXXXXXXXXXXXXXXXXXXXXXXXXXXXXXXXXXXXXXXXXXXXXXXXXXXXXXXXXXXXXXXXXXXXXXXXXXXXXXXXXXXXXXXXXXXXXXXXXXXXXXXXXXXXXXXXXXXXXXXXXXXXXXXXXXXXXXXXXXXXXXXXXXXXXXXXXXXXXXX
XXXXXXXXXXXXXXXXXXXXXXXXXXXXXXXXXXXXXXXXXXXXXXXXXXXXXXXXXXXXXXXXXXXXXXXXXXXXXXXXXXXXXXXXXXXXXXXXXXXXXXXXXXXXXXXXXXXXXXXXXXXXXXXXXXXXXXXXXXXXXXXXXXXXXXXXXXXXXXXXXXXXXXXXXXX
XXXXXXXXXXXXXXXXXXXXXXXXXXXXXXXXXXXXXXXXXXXXXXXXXXXXXXXXXXXXXXXXXXXXXXXXXXXXXXXXXXXXXXXXXXXXXXXXXXXXXXXXXXXXXXXXXXXXXXXXXXXXXXXXXXXXXXXXXXXXXXXXXXXXXXXXXXXXXXXXXXXXXXXXXXX
XXXXXXXXXXXXXXXXXXXXXXXXXXXXXXXXXXXXXXXXXXXXXXXXXXXXXXXXXXXXXXXXXXXXXXXXXXXXXXXXXXXXXXXXXXXXXXXXXXXXXXXXXXXXXXXXXXXXXXXXXXXXXXXXXXXXXXXXXXXXXXXXXXXXXXXXXXXXXXXXXXXXXXXXXXXX
XXXXXXXXXXXXXXXXXXXXXXXXXXXXXXXXXXXXXXXXXXXXXXXXXXXXXXXXXXXXXXXXXXXXXXXXXXXXXXXXXXXXXXXXXXXXXXXXXXXXXXXXXXXXXXXXXXXXXXXXXXXXXXXXXXXXXXXXXXXXXXXXXXXXXXXXXXXXXXXXXXXXXXXXXXX
XXXXXXXXXXXXXXXXXXXXXXXXXXXXXXXXXXXXXXXXXXXXXXXXXXXXXXXXXXXXXXXXXXXXXXXXXXXXXXXXXXXXXXXXXXXXXXXXXXXXXXXXXXXXXXXXXXXXXXXXXXXXXXXXXXXXXXXXXXXXXXXXXXXXXXXXXXXXXXXXXXXXXXXXXXXX
XXXXXXXXXXXXXXXXXXXXXXXXXXXXXXXXXXXXXXXXXXXXXXXXXXXXXXXXXXXXXXXXXXXXXXXXXXXXXXXXXXXXXXXXXXXXXXXXXXXXXXXXXXXXXXXXXXXXXXXXXXXXXXXXXXXXXXXXXXXXXXXXXXXXXXXXXXXXXXXXXXXXXXXXXXXX
XXXXXXXXXXXXXXXXXXXXXXXXXXXXXXXXXXXXXXX111111111111111111111111111111XXXXXXXXXXXXXXXXXXXXXXXXXXXXXXXXXXXXXXXXXXXXXXXXXXXXXXXXXXXXXXXXXXXXXXXXXXXXXXXXXXXXXXXXXXXXXXXXXXXXXXXXXXXXXXXXXXXXXXXXXXX
XXXXXXXXXXXXXXXXXXXXXXXXXXXXXXXXXXXXXXXXXXXXXXXXXXXXXXXXXXXXXXXXXXXXXXXXXXXXXXXXXXXXXXXXXXXXXXXXXXXXXXXXXXXXXXXXXXXXXXXXXXXXXXXXXXXXXXXXXXXXXXXXXXXXXXXXXXXXXXXXXXXXXXXXXXXX
XXXXXXXXXXXXXXXXXXXXXXXXXXXXXXXXXXXXXXXXXXXXXXXXXXXXXXXXXXXXXXXXXXXXXXXXXXXXXXXXXXXXXXXXXXXXXXXXXXXXXXXXXXXXXXXXXXXXXXXXXXXXXXXXXXXXXXXXXXXXXXXXXXXXXXXXXXXXXXXXXXXXXXXXXXXX
XXXXXXXXXXXXXXXXXXXXXXXXXXXXXXXXXXXXXXXXXXXXXXXXXXXXXXXXXXXXXXXXXXXXXXXXXXXXXXXXXXXXXXXXXXXXXXXXXXXXXXXXXXXXXXXXXXXXXXXXXXXXXXXXXXXXXXXXXXXXXXXXXXXXXXXXXXXXXXXXXXXXXXXXXXXX
XXXXXXXXXXXXXXXXXXXXXXXXXXXXXXXXXXXXXXXXXXXXX
XXXXXXXXXXXXXXXXXXXXXXXXXXXXXXXXXXXXXXXXXXXXXXXXXXXXXXXXXXXXXXXXXXXXXXXXXXXXXXXXXXXXXXXXXXXXXXXXXXXXXXXXXXXXXXXXXXXXXXXXXXXXXXXXXXXXXXXXXXXXXXXXXXXXXXXXXXXXXXXXXXXXXXXXXXXX
XXXXXXXXXXXXXXXXXXXXXXXXXXXXXXXXXXXXXXXXXXXXXXXXXXXXXXXXXXXXXXXXXXXXXXXXXXXXXXXXXXXXXXXXXXXXXXXXXXXXXXXXXXXXXXXXXXXXXXXXXXXXXXXXXXXXXXXXXXXXXXXXXXXXXXXXXXXXXXXXXXXXXXXXXXX
XXXXXXXXXXXXXXXXXXXXXXXXXXXXXXXXXXXXXXXXXXXXXXXXXXXXXXXXXXXXXXXXXXXXXXXXXXXXXXXXXXXXXXXXXXXXXXXXXXXXXXXXXXXXXXXXXXXXXXXXXXXXXXXXXXXXXXXXXXXXXXXXXXXXXXXXXXXXXXXXXXXXXXXXXXXX
XXXXXXXXXXXXXXXXXXXXXXXXXXXXXXXXXXXXXXXXXXXXXXXXXXXXXXXXXXXXXXXXXXXXXXXXXXXXXXXXXXXXXXXXXXXXXXXXXXXXXXXXXXXXXXXXXXXXXXXXXXXXXXXXXXXXXXXXXXXXXXXXXXXXXXXXXXXXXXXXXXXXXXXXXXXX
XXXXXXXXXXXXXXXXXXXXXXXXXXXXXXXXXXXXXXXXXXXXXXXXXXXXXXXXXXXXXXXXXXXXXXXXXXXXXXXXXXXXXXXXXXXXXXXXXXXXXXXXXXXXXXXXXXXXXXXXXXXXXXXXXXXXXXXXXXXXXXXXXXXXXXXXXXXXXXXXXXXXXXXXXXXX
XXXXXXXXXXXXXXXXXXXXXXXXXXXXXXXXXXXXXXXXXXXXXXXXXXXXXXXXXXXXXXXXXXXXXXXXXXXXXXXXXXXXXXXXXXXXXXXXXXXXXXXXXXXXXXXXXXXXXXXXXXXXXXXXXXXXXXXXXXXXXXXXXXXXXXXXXXXXXXXXXXXXXXXXXXXX
XXXXXXXXXXXXXXXXXXXXXXXXXXXXXXXXXXXXXXXXXXXXXXXXXXXXXXXXXXXXXXXXXXXXXXXXXXXXXXXXXXXXXXXXXXXXXXXXXXXXXXXXXXXXXXXXXXXXXXXXXXXXXXXXXXXXXXXXXXXXXXXXXXXXXXXXXXXXXXXXXXXXXXXXXXX
XXXXXXXXXXXXXXXXXXXXXXXXXXXXXXXXXXXXXXX000000000000000000000000000000XXXXXXXXXXXXXXXXXXXXXXXXXXXXXXXXXXXXXXXXXXXXXXXXXXXXXXXXXXXXXXXXXXXXXXXXXXXXXXXXXXXXXXXXXXXXXXXXXXXXXXXXXXXXXXXXXXXXXXXXXXX
XXXXXXXXXXXXXXXXXXXXXXXXXXXXXXXXXXXXXXXXXXXXXXXXXXXXXXXXXXXXXXXXXXXXXXXXXXXXXXXXXXXXXXXXXXXXXXXXXXXXXXXXXXXXXXXXXXXXXXXXXXXXXXXXXXXXXXXXXXXXXXXXXXXXXXXXXXXXXXXXXXXXXXXXXXXX
XXXXXXXXXXXXXXXXXXXXXXXXXXXXXXXXXXXXXXXXXXXXXXXXXXXXXXXXXXXXXXXXXXXXXXXXXXXXXXXXXXXXXXXXXXXXXXXXXXXXXXXXXXXXXXXXXXXXXXXXXXXXXXXXXXXXXXXXXXXXXXXXXXXXXXXXXXXXXXXXXXXXXXXXXXX
XXXXXXXXXXXXXXXXXXXXXXXXXXXXXXXXXXXXXXXXXXXXXXXXXXXXXXXXXXXXXXXXXXXXXXXXXXXXXXXXXXXXXXXXXXXXXXXXXXXXXXXXXXXXXXXXXXXXXXXXXXXXXXXXXXXXXXXXXXXXXXXXXXXXXXXXXXXXXXXXXXXXXXXXXXXX
XXXXXXXXXXXXXXXXXXXXXXXXXXXXXXXXXXXXXXXXXXXXX
XXXXXXXXXXXXXXXXXXXXXXXXXXXXXXXXXXXXXXXXXXXXXXXXXXXXXXXXXXXXXXXXXXXXXXXXXXXXXXXXXXXXXXXXXXXXXXXXXXXXXXXXXXXXXXXXXXXXXXXXXXXXXXXXXXXXXXXXXXXXXXXXXXXXXXXXXXXXXXXXXXXXXXXXXXXXX
XXXXXXXXXXXXXXXXXXXXXXXXXXXXXXXXXXXXXXXXXXXXXXXXXXXXXXXXXXXXXXXXXXXXXXXXXXXXXXXXXXXXXXXXXXXXXXXXXXXXXXXXXXXXXXXXXXXXXXXXXXXXXXXXXXXXXXXXXXXXXXXXXXXXXXXXXXXXXXXXXXXXXXXXXXXX
XXXXXXXXXXXXXXXXXXXXXXXXXXXXXXXXXXXXXXXXXXXXXXXXXXXXXXXXXXXXXXXXXXXXXXXXXXXXXXXXXXXXXXXXXXXXXXXXXXXXXXXXXXXXXXXXXXXXXXXXXXXXXXXXXXXXXXXXXXXXXXXXXXXXXXXXXXXXXXXXXXXXXXXXXXXX
XXXXXXXXXXXXXXXXXXXXXXXXXXXXXXXXXXXXXXXXXXXXXXXXXXXXXXXXXXXXXXXXXXXXXXXXXXXXXXXXXXXXXXXXXXXXXXXXXXXXXXXXXXXXXXXXXXXXXXXXXXXXXXXXXXXXXXXXXXXXXXXXXXXXXXXXXXXXXXXXXXXXXXXXXXXX
XXXXXXXXXXXXXXXXXXXXXXXXXXXXXXXXXXXXXXXXXXXXXXXXXXXXXXXXXXXXXXXXXXXXXXXXXXXXXXXXXXXXXXXXXXXXXXXXXXXXXXXXXXXXXXXXXXXXXXXXXXXXXXXXXXXXXXXXXXXXXXXXXXXXXXXXXXXXXXXXXXXXXXXXXXXX
XXXXXXXXXXXXXXXXXXXXXXXXXXXXXXXXXXXXXXXXXXXXXXXXXXXXXXXXXXXXXXXXXXXXXXXXXXXXXXXXXXXXXXXXXXXXXXXXXXXXXXXXXXXXXXXXXXXXXXXXXXXXXXXXXXXXXXXXXXXXXXXXXXXXXXXXXXXXXXXXXXXXXXXXXXXX
XXXXXXXXXXXXXXXXXXXXXXXXXXXXXXXXXXXXXXXXXXXXXXXXXXXXXXXXXXXXXXXXXXXXXXXXXXXXXXXXXXXXXXXXXXXXXXXXXXXXXXXXXXXXXXXXXXXXXXXXXXXXXXXXXXXXXXXXXXXXXXXXXXXXXXXXXXXXXXXXXXXXXXXXXXXX
XXXXXXXXXXXXXXXXXXXXXXXXXXXXXXXXXXXXXXX000000000000000000000000000000XXXXXXXXXXXXXXXXXXXXXXXXXXXXXXXXXXXXXXXXXXXXXXXXXXXXXXXXXXXXXXXXXXXXXXXXXXXXXXXXXXXXXXXXXXXXXXXXXXXXXXXXXXXXXXXXXXXXXXXXXXX
XXXXXXXXXXXXXXXXXXXXXXXXXXXXXXXXXXXXXXXXXXXXXXXXXXXXXXXXXXXXXXXXXXXXXXXXXXXXXXXXXXXXXXXXXXXXXXXXXXXXXXXXXXXXXXXXXXXXXXXXXXXXXXXXXXXXXXXXXXXXXXXXXXXXXXXXXXXXXXXXXXXXXXXXXXXX
XXXXXXXXXXXXXXXXXXXXXXXXXXXXXXXXXXXXXXXXXXXXXXXXXXXXXXXXXXXXXXXXXXXXXXXXXXXXXXXXXXXXXXXXXXXXXXXXXXXXXXXXXXXXXXXXXXXXXXXXXXXXXXXXXXXXXXXXXXXXXXXXXXXXXXXXXXXXXXXXXXXXXXXXXXXX
XXXXXXXXXXXXXXXXXXXXXXXXXXXXXXXXXXXXXXXXXXXXXXXXXXXXXXXXXXXXXXXXXXXXXXXXXXXXXXXXXXXXXXXXXXXXXXXXXXXXXXXXXXXXXXXXXXXXXXXXXXXXXXXXXXXXXXXXXXXXXXXXXXXXXXXXXXXXXXXXXXXXXXXXXXXX
XXXXXXXXXXXXXXXXXXXXXXXXXXXXXXXXXXXXXXXXXXXXX
>P32337
MSALPEEVNRTLLQIVQAFASPDNQIRSVAEKALSEEWITENNIEYLLTFLAEQAAFSQDTTVAALSAVLFRKLALKAPPSSKLMIMSKNITHIRKEVLAQIRSSLLKGFLSERADSIRHKLSDAIAECVQDDLPAWPELLQALIESLKSGNPNFRESSFRILTTVPYLITAVDI
NSILPIFQSGFTDASDNVKIAAVTAFVGYFKQLPKSEWSKLGILLPSLLNSLPRFLDDGKDDALASVFESLIELVELAPKLFKDMFDQIIQFTDMVIKNKDLEPPARTTALELLTVFSENAPQMCKSNQNYGQTLVMVTLIMMTEVSIDDDDAAEWIESDDTDDEEEVTY
DHARQALDRVALKLGGEYLAAPLFQYLQQMITSTEWRERFAAMMALSSAAEGCADVLIGEIPKILDMVIPLINDPHPRVQYGCCNVLGQISTDFSPFIQRTAHDRILPALISKLTSECTSRVQTHAAAALVNFSEFASKDILEPYLDSLLTNLLVLLQSNKLYVQEQALTTI
AFIAEAAKNKFIKYYDTLMPLLLNVLKVNNKDNSVLKGKCMECATLIGFAVGKEKFHEHSQELISILVALQNSDIDEDDALRSYLEQSWSRICRILGDDFVPLLPIVIPPLLITAKATQDVGLIEEEEAANFQQYPDWDVVQVQGKHIAIHTSVLDDKVSAMELLQSYATLL
RGQFAVYVKEVMEEIALPSLDFYLHDGVRAAGATLIPILLSCLLAATGTQNEELVLLWHKASSKLIGGLMSEPMPEITQVYHNSLVNGIKVMGDNCLSEDQLAAFTKGVSANLTDTYERMQDRHGDGDEYNENIDEEEDFTDEDLLDEINKSIAAVLKTTNGHYLKNL
ENIWPMINTFLLDNEPILVIFALVVIGDLIQYGGEQTASMKNAFIPKVTECLISPDARIRQAASYIIGVCAQYAPSTYADVCIPTLDTLVQIVDFPGSKLEENRSSTENASAAIAKILYAYNSNIPNVDTYTANWFKTLPTITDKEAASFNYQFLSQLIENNSPIVCAQSNISAV
VDSVIQALNERSLTEREGQTVISSVKKLLGFLPSSDAMAIFNRYPADIMEKVHKWFA
00000000000000000000000000000000000000000000000000000000000000000000000000000001111111111111000000000000000000000000000000000000000000000000000000000000000000000000000000000000000
00000000000000000000000000000000000000000000000000000000000000000000000000000000000000000000000000000000000000000000000000000000000000000000000000000000000000000000000000000000
00000000000000000000000000000000000000000000000000000000000000000000000000000000000000000000000000000000000000000000000000000000000000000000000000000000000000000000000000000000
00000000000000000000000000000000000000000000000000000000000000000000000000000000000000000000000000000000000000000000000000000000000000000000000000000000000000000000000000000000
00000000000000000000000000000000000000000000000000000000000000000000000000000000000000000000000000000000000000000000000000000000000000000000000000000000000000000000000000000000
00000000000000000000000000000000000000000000000000000000000000000000000000000000000000000000000000000000000000000000000000000000000000000000000000000000000000000000000000000000
000000000000000000000000000000000
00000000000000000000000000000000000000000000000000000000000000000000000000000000000000000000000000000000000000000000000000000000000000000000000000000000000000000000000000000000
00000000000000000000000000000000000000000000000000000000000000000000000000000000000000000000000000000000000000000000000000000000000000000000000000000000000000000000000000000000
00000000000000000000000000000000000000000000000000000000000000000000000000000000000000000000000000000000000000000000000000000000000000000000000000000000000000000000000000000000
00000000000000000000000000000000000000000000000000000000000000000000000000000000000000000000000000000000000000000000000000000000000000000000000000000000000000000000000000000000
00000000000000000000000000000000000000000000000000000000000000000000000000000000000000000000000000000000000000000000000000000000000000000000000000000000000000000000000000000000

[illegible]
>P47054
MKWSAIPFQTLYRSIESGEFDFDLFKEVLPDLQNLNLNTDKLKNNASRSQLEKGEIELSDGSTFKVNQEFIFEAISLSDELNLDEIVACELILSGDTTANNGKVQYFLRRQYILQIVSFIVNCFHEDTELYQELIKNGALVSNILSAFKFIHTQLSEIKQQINKAQILENYNALF
QQNIKFRRDFLLREYDILSQILYGLVDKGAIMKNKDFILSLLHHVSELDSNDFFIIYYTPAFFHLFASLRVLPDADVKLLHSQFMKDLKDDSIYTKPVKVALIFIFFAYFIGWCKEDPKRRADTMDFKTDVDEPMTSAVELGAIEQILIFAADTSIVEQDKSMELFYDIRSLLER
HIPRLIPKQLLDDEKIFSQTTNSTYNPASATDNMSGRGLWNPSYPGMMSTTGTARLNSMPNNVNEYSYTTIVLSDQTQEFFLSSFDDVLQTIITDCAFLLTKIKDAEEDSLLSGEDLTLDDISLKADLERFFLSIYFFYASRPEYSCTFWSDKESNAYGFIEWCSRCNDNL
MRSCFYLMVSSLSFGPENALNVYHYFGENSSISWKNIAQCLSDYTKKISNFNSSLHKRQQFSESTHNDIDSTAVALEEGLNEEAVIFLSSLLTLVGSVTYQVDEDVKSSLSKVFSDVLFEFTKINTPLVGAAFKVISNLVPKLESSRTKFWSFLDSLIFKDSSLNYSSESYRNA
FTNVLTKYSDVLGFLQLFHNLISIHSRENNSEYMVFGKLAFPTRLGQGYRKVGIWPYFDYIFNDILAHVDQIVDIRNKRAVQLPILKIIYTGLCSFDYSVILNSIPAAANLDALVDCENFFNYVQECPAIPIFNYIFTEKIYKSIFNVVDVGVDQLSIELEGGKNQAELLQLAVK
IINKVLDYQETYVEELFPIVKKHGKTDYFLPKNYSLHGLRSFYDAIFFNIPLVAHLGLYVGVDDQILATNSLRILAKLSERSNGSVASLSKRNKLLTIFDSVDESARIKDAFITQLESSITDAGVLALKLELLDFLTSNLSNYSRTMTISHLLLGFQVSNVISLGPNLATFISSGTSLL
DSLISVLEASLNSITKDNIDYAPMRLATAALEIILKLCRNPLTSGLLYSYLIKENFFERIMILDPQVTRFTTWNGSPFDNSTEEKCKNFIESESVGAFLSFLAYRNYWTQYLGLFIHKISFSGTKSEVLTYVNYLISNTMYSVRLFSFLDPLNYGNICEPKETLSIFTNVPLNLEQV
TLNKYCSGNIYDFHKMENLMRLIKRVRAESLHSNSFSLTVSKEQFLKDADVECIKAKSHFTNIISRNKALELNLSVLHSWVQLVQIIVTDGKLEPSTRSNFILEVFGTIIPKISDYIEFNITFSEELVSLAVFLFDIYNRDRKLITDKGTVDGRLYQLFKTCIQGINSPLSSVALRS
DFYILANHYLSRVLSDQVGSEKVLQDLRLGSKKLVEIIWNDVVYGEGTSRVTGILLLDSLIQLANRSKENFILDSLMKTTRLLIIRSLKNTDALLNSTTEHINIDDLLYELTAFKATVFFLIRVAETRGGASALIENNLFRIIAELSFLKVDPDLGLDLMFDEVYVQNSKFLKVN
VTLDNPLLVDKDANGVSLFELIVPIFQLISAVLVSMGSSNKAVVQTVKGLLNTYKRLVIGIFKRDLLREKEDKKNSSDPNNQSLNEMVKLIVMLCTLTGYQNND
[illegible]
>P35729
MACLSRIDANLLQYYEKPEPNNTVDLYVSNNSNNNGLKEGDKSISTPVPQPYGSEYSNCLLLSNSEYICYHFSSRSTLLTFYPLSDAYHGKTINIHLPNASMNQRYTLTIQEVEQQLLVNVILKDGSFLTLQLPLSFLFSSANTLNGEWFHLQNPYDFTVRVPHFLFYVSP
QFSVVFLEDGGLLGLKKVDGVHYEPLLFNDNSYLKSLTRFFSRSSKSDYDSVISCKLFHERYLIVLTQNCHLKIWDLTSFTLIQDYDMVSQSDSDPSHFRKVEAVGEYLSLYNNTLVTLLPLENGLFQMGTLLVDSSGILTYTFQNNIPTNLSASAIWSIVDLVLTRPLELNV
EASYLNLIVLWKSGTASKLQILNVNDESFKNYEWIESVNKSLVDLQSEHDLDIVTKTGDVERGFCNLKSRYGTQIFERAQQILSENKIIMAHNEDEEYLANLETILRDVKTAFNEASSITLYGDEIILVNCFQPYNHSLYKLNTTVENWFYNMHSETDGSELFKYLRTLNGF
ASTLSNDVLRSISKKFLDIITGELPDSMTTVEKFTDIFKNCLENQFEITNLKILFDELNSFDIPVVLNDLINNQMKPGIFWKKDFISAIKFDGFTSIISLESLHQLLSIHYRITLQVLLTFVLFDLDTEIFGQHISTLLDLHYKQFLLLNLYRQDKCLLAEVLLKDSSEFSFGVKFFNY
GQLIAYIDSLNSNVYNASITENSFFMTFFRSYIIENTSHKNIRFFLENVECPFYLRHNEVQEFMFAMTLFSCGNFDQSYEIFQLHDYPEAINDKLPTFLEDLKSENYHGDSIWKDLLCTFTVPYRHSAFYYQLSLLFDRNNSQEFALKCISKSAEYSLKEIQIEELQDFKEKQHI
HYLNLLIHFRMFEEVLDVLRLGHECLSDTVRTNFLQLLQEDIYSRDFFSTLLRLCNAHSDNGELYLRTVDIKIVDSILSQNLRSGDWECFKKLYCFRMLNKSERAAAEVLYQYILMQADLDVIRKRKCYLMVINVLSSFDSAYDQWILNGSKVVTLTDLRDELRGL
[illegible]
>P32566
MDLFKRKVKEWVYSLSTDDHYAEYNPDETPTFNMGKRLNSNNGQVNPSQMHLNSVDEEMSMGFQNGVPSNEDINIDEFTSTESNDGVSETLLAWRHIDFWTSEHNPDLNATLSDPCTQNDITHAEEDLEVSFPNPVKASFKIHDGQEDLESMTGTSGLFYGFQ
LMTLDQVVAMTQAWRNVAKNLNKRSQQGLSHVTSTGSSSSMERLNGNKFKLPNIPDQKSIPPNAVQPVYAHPAWIPLITDNAGNHIGVDLAPGPNGKYAQIITFGRDFDTKFVIAENWGEFLLSFANDLEAGNWYLVDDNDDYFSGDGELVFRDKKSNGPIQD
YFEVLKRRTWIKYQENLRSQQKSQPDTSLQEQKYVPASQKKVAAEQPSTLNAESIKGEDSGSADVQSVQDHESVKIVKTEPSEAETTTVNTESLGQAEHEIKADNVDIKQESERKEDEKQPKVEEKEHVENEHVTESAKKDDDVNKQTEEMNKKEENEIRSDDAK
VEEAREEFENIAL
[illegible]
>O15234
MADRRRQRASQDTEDEESGASGSDSGGSPLRGGGSCSGSAGGGGSGSLPSQRGGRTGALHLRRVESGGAKSAEESECESEDGIEGDAVLSDYESAEDSEGEEGYSEEENSKVELKSEANDAVNSSTKEEKGEEKPDTKSTVTGERQSGDGQESTEPVENKVGKK
GPKHLDDDEDRKNPAYIPRKGLFFEHDLRGQTQEEEVRPKGRQRKLWKDEGRWEHDKFREDEQAPKSRQELIALYGYDIRSAHNPDDIKPRRIRKPRYGSPPQRDPNWNGERLNKSHRHQGLGGTLPPRTFINRNAAGTGRMSAPRNYSRSGGFKEGRAGFRPV
EAGGQHGGRSGETVKHEISYRSRRLEQTSVRDPSPEADAPVLGSPEKEEAASEPPAAAPDAAPPPPDRPIEKKSYSRARRTRTKVGDAVKLAEEVPPPPEGLIPAPPVPETTPTPPTKTGTWEAPVDSSTSGLEQDVAQLNIAEQNWSPGQPSFLQPRELRGMPNHI
HMGAGPPPQFNRMEEMGVQGGRAKRYSSQRQRPVPEPPAPPVHISIMEGHYYDPLQFQGPIYTHGDSPAPLPPQGMLVQPGMNLPHPGLHPHQTPAPLPNPGLYPPPVSMSPGQPPPQQLLAPTYFSAPGVMNFGNPSYPYAPGALPPPPPPHLYPNTQAP
SQVYGGVTYYNPAQQQVQPKPSPPRRTPQPVTIKPPPPEVVSRGSS
[illegible]
>Q9BS26
MHPAVFLSLPDLRCSLLLLVTWVFTPVTTEITSLDTENIDEILNNADVALVNFYADWCRFSQMLHPIFEEASDVIKEEFPNENQVVFARVDCDQHSDIAQRYRISKYPTLKLFRNGMMMKREYRGQRSVKALADYIRQQKSDPIQEIRDLAEITTLDRSKRNIIGYFEQK
DSDNYRVFERVANILHDDCAFLSAFGDVSKPERYSGDNIIYKPPGHSAPDMVYLGAMTNFDVTYNWIQDKCVPLVREITFENGEELTEEGLPFLILFHMKEDTESLEIFQNEVARQLISEKGTINFLHADCDKFRHPLLHIQKTPADCPVIAIDSFRHMYVFGDFKDVLIP
GKLKQFVFDLHSGKLHREFHHGPDPTDTAPGEQAQDVASSPPESSFQKLAPSEYRYTLLRDRDEL
[illegible]
>P53136
MRLLVSCVDSGSIKEVLCNIGTDTSVQSALQPFHVAPHLAEGLKAYVDRMWVISEDEAILARNSGVVELVKISKHLKENEALQVDPKGESKNEKSLSDDLPKFDISEFEITSSVSDLFDDAKLESLSSKSVKRTKLVDGFVTLCPIKKDSSNNTFVAATKSGLLHIIKKGEDK
KLIKLASLGLKAPVEFLQLYDLEDTDTDKYIFAYGGEENLIKLVEIDSSFQSLKQIWEAKNVKNDRLDMRVPVWPMALRFLEPSPGKTEKGKLNYQFAAITRWSHLTKYSTQHGRKPFAQIDLLPNREPLSQMEVFDAKGENVVSSLGNFQSETFNELNVITTDYKKNV
FKFDGNGRMLGKVGRDDITGSSTYIHVHDGKYLLQGGLDRYVRIFDIKTNKMLVKVYVGSRINFIVMLDDVEIEMPLSPSAKAAKGKQKRKVTELEEDADELWNKLEGKVAASKASKKSKI

00000000000000000000000000000000000000000000000000000000000000000000000000000000000000000000000000000000000000000000000000000000000000000000000000000000000000000000000000000000
00000000000000000000000000000000000000000000000000000000000000000000000000000000000000000000000000000000000000000000000000000000000000000000000000000000000000000000000000000000
000000000000000000000000000000000000000000000000000000000000000000000000000000000111111111111111111111111111111
00000000000000000000000000000000000000000000000000000000000000000000000000000000000000000000000000000000000000000000000000000000000000000000000000000000000000000000000000000000
00000000000000000000000000000000000000000000000000000000000000000000000000000000000000000000000000000000000000000000000000000000000000000000000000000000000000000000000000000000
000000000000000000000000000000000000000000000000000000000000000000000000000000000000000000000000000000000000000
00000000000000000000000000000000000000000000000000000000000000000000000000000000000000000000000000000000000000000000000000000000000000000000000000000000000000000000000000000000
00000000000000000000000000000000000000000000000000000000000000000000000000000000000000000000000000000000000000000000000000000000000000000000000000000000000000000000000000000000
000000000000000000000000000000000000000000000000000000000000000000000000000000000000000000000000000000000000000
>P0ADV9
MSKARRWVIIVLSLAVLVMIGINMAEKDDTAQVVVNNNDPTYKSEHTDTLVYNPEGALSYRLIAQHVEYYSDQAVSWFTQPVLTTFDKDKIPTWSVKADKAKLTNDRMLYLYGHVEVNALVPDSQLRRITTDNAQINLVTQDVTSEDLVTLYGTTFNSSGLKMRG
NLRSKNAELIEKVRTSYEIQNKQTQP
00000000000000000000001111111111111111111111111111111111110000000000000000000000000000000000000000000000000000000000000000000000000000000000000000000000000000000000000000000000
000000000000000
00000000000000000000000000000000000000000000000000000000000000000000000000000000000000000000000000000000000000000000000000000000000000000000000000000000000000000000000000000000
000000000000000
00000000000000000000000000000000000000000000000000000000000000000000000000000000000000000000000000000000000000000000000000000000000000000000000000000000000000000000000000000000
000000000000000
>Q2TUW1
MAEEKHHKHRLFHHHKDEDNKPVETDTGYDNTSYSKPSDDYDSGFNKPSYESSGGGYEGGYNKTSYSSDEPASGGGYEGGYKKTGYSGGIDETSGGYGAGGGGYSDVTDGGYGKPSGGYGAAGGGGVYPDTTTDGGYSKTSGGGYDDEVDYKKEEKHHKHLE
HLGELGAASAAAYALHEKHKAEKDPEHAHRHKIEEEVAAAAAVGSGGFAFHEHHEKKEAKEQDEEAHGKKHHHLFG
11111111111111111111111111111111111111111111111111111111111111111111111111111111111111111111111111111111111111111111111111111111111111111111111111111111111111111111111111111111
11111111111111111111111111111111111111111111111111111111111111
00000000000000000000000000000000000000000000000000000000000000000000000000000000000000000000000000000000000000000000000000000000000000000000000000000000000000000000000000000000
00000000000000000000000000000000000000000000000000000000000000
00000000000000000000000000000000000000000000000000000000000000000000000000000000000000000000000000000000000000000000000000000000000000000000000000000000000000000000000000000000
00000000000000000000000000000000000000000000000000000000000000
>P02808
MKFLVFAFILALMVSMIGADSSEEKFLRRIGRFGYGYGPYQPVPEQPLYPQPYQPQYQQYTF
XXXXXXXXXXXXXXXXXXX1111111111111111111111111111111111111111111
XXXXXXXXXXXXXXXXXXX0000000000000000000000000000000000000000000
XXXXXXXXXXXXXXXXXXX0000000000000000000000000000000000000000000
>A0A140GKJ0
MSWALEMADTFLDNMRVGPRTYADVRDEINKRGREDREAARTAVHDPERPLLRSPGLLPEIAPNASLGVVHRRTGGTVTDSPRNPVTR
XXXXXXXXXXXXXXXXXXXXXXXXXXXXXXXXXXXXXXXXXXXXXXXXXXXXXXX111111111111111111111111111111111
XXXXXXXXXXXXXXXXXXXXXXXXXXXXXXXXXXXXXXXXXXXXXXXXXXXXXXX000000000000000000000000000000000
XXXXXXXXXXXXXXXXXXXXXXXXXXXXXXXXXXXXXXXXXXXXXXXXXXXXXXX000000000000000000000000000000000
>P32521
MYNPYQQQGMGYQQQQQQQQQPNGFYPQQQQGQSSNQPQGQPQPQQQMAFNQPQATGIGGMPQSFGNSFSSMPQQPQTGYNNNGNNGSVYGNGNFGQQPQQQQQQAKPQHTGYVPNSSMPMMNTTGTMPPPNPAQQPQLQSIQPQGT
GYYQAANTANVHSVQPLQSQGTGYYVSTPNLISSNQTQQPLQAQGTGYYQSQPQQVPPPQQAQSLQPLKPQQTGFYLQPQNQAPLEPLKPTATGFVNSFANNGLNNDIKIPAIRLSFITAQDQAKFETLFRSIVTNGSNTVSGANCRKILMRSGLPPSQLARIWTL
CDTSKAGELLFPEFALAMHLINDVLQGDTIPYELDSKTKNEVSSFIDAINLSIANQDSSANDAPKTPFDEFITAGVQNLQPQPTGYMPQTSFGIPLQSQITGGGVASALNPQSTGFMAPTTFNMSMNTGTPGLNPQITGGAPASMQPNITGNALQPQTTGMMPQ
TTGMMPQTTGMMPQTSFGVNLGPQLTGGALQSQYTGGYGSVMPQQSGPASMPNLSFNQQGLQSQLTGLQPQPTGFLPPSNFSATMPLTAQKTGFGNNEIYTKSNFNNNLIDNSSQDKISTEEKSLFYKIFETFDTQNKGLLDSPTAVEIFRKSGLNRADLEQIW
NLCDINNTGQLNKQEFALGMHLVYGKLNGKPIPNVLPSSLIPSSTKLLDNLKNQLKTEPTTTKEKPSFGKIDALSYKNNDDDVLPNYRNRRKVYSAKNEEQSSFSSPSAKSVNHSSSTLQTDDISVDKTVEKKTAKPKYAGFSREINLKNIASLENEIKNISNPENCYDSSIP
SDLTSRFDAIIAKLPNLFNEISTIDNEITNAKIQLYRKKNPSSIIGSGPNGEITENDRKKAKSRALLRARMSALTGKSTESEDSLSMEDEQQSAEIKRIQQENGKNQEIIKDIRSSISDISASLKSTMTGSNMISNQEFERWEFGIGLEDGVREFLDDLKSNSNKSVTESSPFVP
SSTPTPVDDRSSSPSYSQFKTAEERAAYLKEQAKKRMKEKLAKFDKNRRNVTQSSRSISSENSREQPQQIAGSSNLVEPRATPFQEEKYVEVAQPTQPVQSTQPVQPTQPVQPTQPVQPTQPVQPTQPVQPTQPVQNVYNAKQESDDEDEDDEEKRLQEELKRLK
LKKKADKEKRLAALRKQIEDAQNESDEEETNGKDNFGGHVNVPQAAPVAPSAAFSQNSTNAPRSVHAAVTPAAGKNSTGLPSTTMGHNPYFKDASASSTSTFDARAAEMQRRIQRGLDEDEDDGWSDEDESNNRVAVDNKVEEAKIGHPDHARAPPVTAAPL
PSVTPVPPAVPVPQANTSNEKSSPIPIAPIPPSVTQEPPVPLAPPLPAVDGFQEPPIPSAPAIATAVQKSGSSTPALAGGVLPPPPPLPTQQASTSEPIIAHVDNYNGAEKGTGAYGSDSDDDVLSIPESVGTDEEEEGAQPVSTAGIPSIPPAGIPPPPPLP
00000000000000000000000000000000000000000000000000000000000000000000000000000000000000000000000000000000000000000000000000000000000000000000000000000000000000000000000000000000
00000000000000000000000000000000000000000000000000000000000000000000000000000000000000000000000000000000000000000000000000000000000000000000000000000000000000000000000000000000
00000000000000000000000000000000000000000000000000000000000000000000000000000000000000000000000000000000000000000000000000000000000000000000000000000000000000000000000000000000
00000000000000000000000000000000000000000000000000000000000000000000000000000000000000000000000000000000000000000000000000000000000000000000000000000000000000000000000000000000
00000000000000000000000000000000000000000000000000000000000000000000000000000000000000000000000000000000000000000000000000000000000000000000000000000000000000000000000000000000
00000000000000000000000000000000000000000000000000000000000000000000000000000000000000000000000000000000000000000000000000000000000000000000000000000000000000000000000000000000
00000000000000000000000000000000000000000000000000000000000000000000000000000000000000000000000000000000000000000000000000000000000000111111111111111111111111111111111111111111
11111111111111111111111111111111111111111111111111111111111111111111111111111111111111111111111111111111111111111111111111111111111111111111111111111111111111111111111111111111
111111111111111111111111111111111111111111111111111111111111111111111111
00000000000000000000000000000000000000000000000000000000000000000000000000000000000000000000000000000000000000000000000000000000000000000000000000000000000000000000000000000000
00000000000000000000000000000000000000000000000000000000000000000000000000000000000000000000000000000000000000000000000000000000000000000000000000000000000000000000000000000000
00000000000000000000000000000000000000000000000000000000000000000000000000000000000000000000000000000000000000000000000000000000000000000000000000000000000000000000000000000000
00000000000000000000000000000000000000000000000000000000000000000000000000000000000000000000000000000000000000000000000000000000000000000000000000000000000000000000000000000000
00000000000000000000000000000000000000000000000000000000000000000000000000000000000000000000000000000000000000000000000000000000000000000000000000000000000000000000000000000000
00000000000000000000000000000000000000000000000000000000000000000000000000000000000000000000000000000000000000000000000000000000000000000000000000000000000000000000000000000000
00000000000000000000000000000000000000000000000000000000000000000000000000000000000000000000000000000000000000000000000000000000000000000000000000000000000000000000000000000000
00000000000000000000000000000000000000000000000000000000000000000000000000000000000000000000000000000000000000000000000000000000000000000000000000000000000000000000000000000000
000000000000000000000000000000000000000000000000000000000000000000000000
00000000000000000000000000000000000000000000000000000000000000000000000000000000000000000000000000000000000000000000000000000000000000000000000000000000000000000000000000000000
00000000000000000000000000000000000000000000000000000000000000000000000000000000000000000000000000000000000000000000000000000000000000000000000000000000000000000000000000000000
00000000000000000000000000000000000000000000000000000000000000000000000000000000000000000000000000000000000000000000000000000000000000000000000000000000000000000000000000000000
00000000000000000000000000000000000000000000000000000000000000000000000000000000000000000000000000000000000000000000000000000000000000000000000000000000000000000000000000000000
00000000000000000000000000000000000000000000000000000000000000000000000000000000000000000000000000000000000000000000000000000000000000000000000000000000000000000000000000000000
00000000000000000000000000000000000000000000000000000000000000000000000000000000000000000000000000000000000000000000000000000000000000000000000000000000000000000000000000000000
00000000000000000000000000000000000000000000000000000000000000000000000000000000000000000000000000000000000000000000000000000000000000000000000000000000000000000000000000000000
00000000000000000000000000000000000000000000000000000000000000000000000000000000000000000000000000000000000000000000000000000000000000000000000000000000000000000000000000000000
000000000000000000000000000000000000000000000000000000000000000000000000
>P24385
MEHQLLCCEVETIRRAYPDANLLNDRVLRAMLKAEETCAPSVSYFKCVQKEVLPSMRKIVATWMLEVCEEQKCEEEVFPLAMNYLDRFLSLEPVKKSRLQLLGATCMFVASKMKETIPLTAEKLCIYTDNSIRPEELLQMELLLVNKLKWNLAAMTPHDFIEHFLSKM
PEAEENKQIIRKHAQTFVALCATDVKFISNPPSMVAAGSVVAAVQGLNLRSPNNFLSYYRLTRFLSRVIKCDPDCLRACQEQIEALLESSLRQAQQNMDPKAAEEEEEEEEEVDLACTPTDVRDVDI
11111111111111111111111000000000000000000000000000000000000000000000000000000000000000000000000000000000000000000000000000000000000000000000000000000000000000000000000000000000
00000000000000000000000000000000000000000000000000000000000000000000000000000000000000000000000000000000000000000000000000000000000000000000
11111111111111111111111000000000000000000000000000000000000000000000000000000000000000000000000000000000000000000000000000000000000000000000000000000000000000000000000000000000
00000000000000000000000000000000000000000000000000000000000000000000000000000000000000000000000000000000000000000000000000000000000000000000
00000000000000000000000000000000000000000000000000000000000000000000000000000000000000000000000000000000000000000000000000000000000000000000000000000000000000000000000000000000
00000000000000000000000000000000000000000000000000000000000000000000000000000000000000000000000000000000000000000000000000000000000000000000
>Q92905
MAASGSGMAQKTWELANNMQEAQSIDEIYKYDKKQQQEILAAKPWTKDHHYFKYCKISALALLKMVMHARSGGNLEVMGLMLGKVDGETMIIMDSFALPVEGTETRVNAQAAAYEYMAAYIENAKQVGRLENAIGWYHSHPGYGCWLSGIDVSTQMLNQ
QFQEPFVAVVIDPTRTISAGKVNLGAFRTYPKGYKPPDEGPSEYQTIPLNKIEDFGVHCKQYYALEVSYFKSSLDRKLLELLWNKYWVNTLSSSSLLTNADYTTGQVFDLSEKLEQSEAQLGRGSFMLGLETHDRKSEDKLAKATRDSCKTTIEAIHGLMSQVIKDKLFN
QINIS
00000000000000000000000000000000000000000000000000000000000000000000000000000000000000000000000000000000000000000000000000000000000000000000000000000000000000000000000000000000
0000000000000000000000000000000000000000000000000000000000000000000000000000000000000000000000000000000000111111111111100000000000000000000000000000000000000
00000000000000000000000000000000000000000000000000000000000000000000000000000000000000000000000000000000000000000000000000000000000000000000000000000000000000000000000000000000
00000000000000000000000000000000000000000000000000000000000000000000000000000000000000000000000000000000000000000000000000000000000000000000000000000000000000
00000000000000000000000000000000000000000000000000000000000000000000000000000000000000000000000000000000000000000000000000000000000000000000000000000000000000000000000000000000
00000000000000000000000000000000000000000000000000000000000000000000000000000000000000000000000000000000000000000000000000000000000000000000000000000000000000
>Q05471
MTTSRKSHAKDKKAGGEQDLADLKFRYDLLTNELFHLREFVSLVDYDPTHFNDSESFQKFLRETHLSLEERGEKFTDDVAKKGTNGDLTRRRRNLRTSTVVSSETTNEKKGDIELKLESIAPLVRNKCEELKYKLSDHSNRKSIVPQKRPIQHLKKREAAKSLKFKSERKEN
PLPLHEHIAEERYDHIAKVEEPSEAFTIKCPSDDSSFENTSEHYSDNFYFTTSSEEEDIKKKRGRKKKKPRIKLVVHPPKQTITNPLHVVKPGYESLHEYIASFKSLEDDLTLEEYNKYIDEQRRLLSRLKKGIENGALKYDKETDSLQPITSKEIKTIITYKPDPISYFYKQQDLQI
HTDHLINQGIHMSKLFRSSTKARIARAKKVSQMIEQHFKHVAGAEERKAKEEERHKKSLARFAVQAVKKRWNMAEKAYRILRKDEEEQLKRIEGKQHLSKMLEKSTQLLEAQLNQVNDDGRSSTPSSDSNDVLSESDDDMDDELSTSSDEDEEVDADVGLENSPAS
TEATPTDESLNLIQLKEKYGHFNGSSTVYDSRNKDEKFPTLDKHESSSSESSVMTGEESSIYSSSENESQNENDRESDDKTPSVGLSALFGKGEESDGDLDLDDSEDFTVNSSSVEGEELEKDQVDNSAATFERAGDFVHTQNENRDDIKDVEEDAETKVQEEQLSVV
DVPVPSLLRGNLRTYQKQGLNWLASLYNNHTNGILADEMGLGKTIQTISLLAYLACEKENWGPHLIVVPTSVLLNWEMEFKRFAPGFKVLTYYGSPQQRKEKRKGWNKPDAFHVCIVSYQLVVQDQHSFKRKRWQYMVLDEAHNIKNFRSTRWQALLNFNTQR
RLLLTGTPLQNNLAELWSLLYFLMPQTVIDGKKVSGFADLDAFQQWFGRPVDKIIETGQNFGQDKETKKTVAKLHQVLRPYLLRRLKADVEKQMPAKYEHIVYCKLSKRQRFLYDDFMSRAQTKATLASGNFMSIVNCLMQLRKVCNHPNLFEVRPILTSFVLEHCV
ASDYKDVERTLLKLFKKNNQVNRVDLDFLNLVFTLNDKDLTSYHAEEISKLTCVKNFVEEVNKLRETNKQLQEEFGEASFLNFQDANQYFKYSNKQKLEGTVDMLNFLKMVNKLRCDRRPIFGKNLIDLLTKDRRVKYDKSSIIDNELIKPLQTRVLDNRKIIDTFAVLTP
SAVSLDMRKLALGLNDDSSVGENTRLKVMQNCFEVSNPLHQLQTKLTIAFPDKSLLQYDCGKLQKLAILLQQLKDNGHRALIFTQMTKVLDVLEQFLNYHGYLYMRLDGATKIEDRQILTERFNTDSRITVFILSSRSGGLGINLTGADTVIFYDSDWNPAMDKQCQD
RCHRIGQTRDVHIYRFVSEHTIESNILKKANQKRQLDNVVIQEGDFTTDYFSKLSVRDLLGSELPENASGGDKPLIADADVAAKDPRQLERLLAQAEDEDDVKAANLAMREVEIDNDDFDESTEKKAANEEEENHAELDEYEGTAHVDEYMIRFIANGYYY
00000000000000000000000000000000000000000000000000000000000000000000000000000000000000000000000000000000000000000000000000000000000000000000000000000000000000000000000000000000
00000000000000000000000000000000000000000000000000000000000000000000000000000000000000000000000000000000000000000000000000000000000000000000000000000000000000000000000000000000
00000000000000000000000000000000000000000000000000000000000000000000000000000000000000000000000000000000000000000000000000000000000000000000000000000000000000000000000000000000
00000000000000000000000000000000000000000000000000000000000000000000011111111111111111111111111111000000000000000000000000000000000000000000000000000000000000000000000000000000
00000000000000000000000000000000000000000000000000000000000000000000000000000000000000000000000000000000000000000000000000000000000000000000000000000000000000000000000000000000
00000000000000000000000000000000000000000000000000000000000000000000000000000000000000000000000000000000000000000000000000000000000000000000000000000000000000000000000000000000
00000000000000000000000000000000000000000000000000000000000000000000000000000000000000000000000000000000000000000000000000000000000000000000000000000000000000000000000000000000
00000000000000000000000000000000000000000000000000000000000000000000000000000000000000000000000000000000000000000000000000000000000000000000000000000000000000000000000000000000
0000000000000000000000000000000000000000000000000000000000000000000000000000000000000000000000000000000000
00000000000000000000000000000000000000000000000000000000000000000000000000000000000000000000000000000000000000000000000000000000000000000000000000000000000000000000000000000000
00000000000000000000000000000000000000000000000000000000000000000000000000000000000000000000000000000000000000000000000000000000000000000000000000000000000000000000000000000000
00000000000000000000000000000000000000000000000000000000000000000000000000000000000000000000000000000000000000000000000000000000000000000000000000000000000000000000000000000000
00000000000000000000000000000000000000000000000000000000000000000000011111111111111111111111111111000000000000000000000000000000000000000000000000000000000000000000000000000000
00000000000000000000000000000000000000000000000000000000000000000000000000000000000000000000000000000000000000000000000000000000000000000000000000000000000000000000000000000000
00000000000000000000000000000000000000000000000000000000000000000000000000000000000000000000000000000000000000000000000000000000000000000000000000000000000000000000000000000000
00000000000000000000000000000000000000000000000000000000000000000000000000000000000000000000000000000000000000000000000000000000000000000000000000000000000000000000000000000000
00000000000000000000000000000000000000000000000000000000000000000000000000000000000000000000000000000000000000000000000000000000000000000000000000000000000000000000000000000000
0000000000000000000000000000000000000000000000000000000000000000000000000000000000000000000000000000000000
00000000000000000000000000000000000000000000000000000000000000000000000000000000000000000000000000000000000000000000000000000000000000000000000000000000000000000000000000000000
00000000000000000000000000000000000000000000000000000000000000000000000000000000000000000000000000000000000000000000000000000000000000000000000000000000000000000000000000000000
00000000000000000000000000000000000000000000000000000000000000000000000000000000000000000000000000000000000000000000000000000000000000000000000000000000000000000000000000000000
00000000000000000000000000000000000000000000000000000000000000000000000000000000000000000000000000000000000000000000000000000000000000000000000000000000000000000000000000000000
00000000000000000000000000000000000000000000000000000000000000000000000000000000000000000000000000000000000000000000000000000000000000000000000000000000000000000000000000000000
00000000000000000000000000000000000000000000000000000000000000000000000000000000000000000000000000000000000000000000000000000000000000000000000000000000000000000000000000000000
00000000000000000000000000000000000000000000000000000000000000000000000000000000000000000000000000000000000000000000000000000000000000000000000000000000000000000000000000000000

00000000000000000000000000000000000000000000000000000000000000000000000000000000000000000000000000000000000000000000000000000000000000000000000000000000000000000000000000000000
0000000000000000000000000000000000000000000000000000000000000000000000000000000000000000000000000000000000
>P60761
MDCCTESACSKPDDDILDIPLDDPGANAAAAKIQASFRGHMARKKIKSGECGRKGPGPGGPGGAGGARGGAGGGPSGD
XXXXXXXXXXXXXXXXXXXXXXXXXXXXX111111111111111111XXX111111111111XXXXXXXXXXXXXXXX
XXXXXXXXXXXXXXXXXXXXXXXXXXXXX000000000000000000XXX000000000000XXXXXXXXXXXXXXXX
XXXXXXXXXXXXXXXXXXXXXXXXXXXXX000000000000000000XXX000000000000XXXXXXXXXXXXXXXX
>P50498
MKVIKTLSIINFFIFVTFNIKNESKYSNTFINNAYNMSIRRSMAESKPSTGAGGSAGGSAGGSAGGSAGGSAGGSAGSGDGNGADAEGSSSTPATTTTTKTTTTTTTTNDAEASTSTSSENPNHKNAETNPKGKGEVQEPNQANKETQNNSNVQQDSQTKSNVPP
TQDADTKSPTAQPEQAENSAPTAEQTESPELQSAPENKGTGQHGHMHGSRNNHPQNTSDSQKECTDGNKENCGAATSLLNNSSNIASINKFVVLISATLVLSFAIFI
11111111111111111111111111111111111111111111111111111111111111111111111111111111111111111111111111111111111111111111111111111111111111111111111111111111111111111111111111111111
111111111111111111111111111111111111111111111111111111111111111111111111111111111111111111111111
00000000000000000000000000000000000000000000000000000000000000000000000000000000000000000000000000000000000000000000000000000000000000000000000000000000000000000000000000000000
000000000000000000000000000000000000000000000000000000000000000000000000000000000000000000000000
00000000000000000000000000000000000000000000000000000000000000000000000000000000000000000000000000000000000000000000000000000000000000000000000000000000000000000000000000000000
000000000000000000000000000000000000000000000000000000000000000000000000000000000000000000000000
>Q0JRZ9
MVMAYFVENFWGEKNSGFDVLYHNMKHGQISTKELADFVRERATIEEAYSRSMTKLAKSASNYSQLGTFAPVWDVFKTSTEKLANCHLDLVRKLQELIKEVQKYGEEQVKSHKKTKEEVAGTLEAVQTIQSITQALQKSKENYNAKCVEQERLKKEGATQREIEKA
AVKSKKATDTYKLYVEKYALAKADFEQKMTETAQKFQDIEETHLIHIKEIIGSLSNAIKEIHLQIGQVHEEFINNMANTTVESLIQKFAESKGTGKERPGLIEFEECDTASAVEGIKPRKRKTFALPGIIKKEKDAESVECPDADSLNIPDVDEEGYSIKPETNQNDTKENHFY
SSSDSDSEDEEPKKYRIEIKPMHPNNSHHTMASLDELKVSIGNITLSPAISRHSPVQMNRNLSNEELTKSKPSAPPNEKGTSDLLAWDPLFGPSLDSSSSSSLTSSSSARPTTPLSVGTIVPPPRPASRPKLTSGKLSGINEIPRPFSPPVTSNTSPPPAAPLARAESSSSISSS
ASLSAANTPTVGVSRGPSPVSLGNQDTLPVAVALTESVNAYFKGADPTKCIVKITGDMTMSFPSGIIKVFTSNPTPAVLCFRVKNISRLEQILPNAQLVFSDPSQCDSNTKDFWMNMQAVTVYLKKLSEQNPAASYYNVDVLKYQVSSNGIQSTPLNLATYWKCSAS
TTDLRVDYKYNPEAMVAPSVLSNIQVVVPVDGGVTNMQSLPPAIWNAEQMKAFWKLSSISEKSENGGSGSLRAKFDLSEGPSKPTTLAVQFLSEGSTLSGVDFELVGTGYRLSLIKKRFATGRYLADC
00000000000000000000000000000000000000000000000000000000000000000000000000000000000000000000000000000000000000000000000000000000000000000000000000000000000000000000000000000000
00000000000000000000000000000000000000000000000000000000000000000000111111111111111100000000000000000000000000000000000000000000000000000000000000000000000000000000000000000000
00000000000000000000000000000000000000000000000000000000000000000000000000000000000000000000000000000000000000000000000000000000000000000000000000000000000000000000000000000000
00000000000000000000000000000000000000000000000000000000000000000000000000000000000000000000000000000000000000000000000000000000000000000000000000000000000000000000000000000000
00000000000000000000000000000000000000000000000000000000000000000000000000000000000000000000000000000000000000000000000000000000000000000000000000000000000000000000000000000000
0000000000000000000000000000000000000000000000000000000000000000000000000000000000000000000000000000000000
00000000000000000000000000000000000000000000000000000000000000000000000000000000000000000000000000000000000000000000000000000000000000000000000000000000000000000000000000000000
00000000000000000000000000000000000000000000000000000000000000000000000000000000000000000000000000000000000000000000000000000000000000000000000000000000000000000000000000000000
00000000000000000000000000000000000000000000000000000000000000000000000000000000000000000000000000000000000000000000000000000000000000000000000000000000000000000000000000000000
00000000000000000000000000000000000000000000000000000000000000000000000000000000000000000000000000000000000000000000000000000000000000000000000000000000000000000000000000000000
00000000000000000000000000000000000000000000000000000000000000000000000000000000000000000000000000000000000000000000000000000000000000000000000000000000000000000000000000000000
0000000000000000000000000000000000000000000000000000000000000000000000000000000000000000000000000000000000
00000000000000000000000000000000000000000000000000000000000000000000000000000000000000000000000000000000000000000000000000000000000000000000000000000000000000000000000000000000
00000000000000000000000000000000000000000000000000000000000000000000000000000000000000000000000000000000000000000000000000000000000000000000000000000000000000000000000000000000
00000000000000000000000000000000000000000000000000000000000000000000000000000000000000000000000000000000000000000000000000000000000000000000000000000000000000000000000000000000
00000000000000000000000000000000000000000000000000000000000000000000000000000000000000000000000000000000000000000000000000000000000000000000000000000000000000000000000000000000
00000000000000000000000000000000000000000000000000000000000000000000000000000000000000000000000000000000000000000000000000000000000000000000000000000000000000000000000000000000
0000000000000000000000000000000000000000000000000000000000000000000000000000000000000000000000000000000000
>O43663
MRRSEVLAEESIVCLQKALNHLREIWELIGIPEDQRLQRTEVVKKHIKELLDMMIAEEESLKERLIKSISVCQKELNTLCSELHVEPFQEEGETTILQLEKDLRTQVELMRKQKKERKQELKLLQEQDQELCEILCMPHYDIDSASVPSLEELNQFRQHVTTLRETKASRREE
FVSIKRQIILCMEALDHTPDTSFERDVVCEDEDAFCLSLENIATLQKLLRQLEMQKSQNEAVCEGLRTQIRELWDRLQIPEEEREAVATIMSGSKAKVRKALQLEVDRLEELKMQNMKKVIEAIRVELVQYWDQCFYSQEQRQAFAPFCAEDYTESLLQLHDAEIVRLK
NYYEVHKELFEGVQKWEETWRLFLEFERKASDPNRFTNRGGNLLKEEKQRAKLQKMLPKLEEELKARIELWEQEHSKAFMVNGQKFMEYVAEQWEMHRLEKERAKQERQLKNKKQTETEMLYGSAPRTPSKRRGLAPNTPGKARKLNTTTMSNATANSSIRPIF
GGTVYHSPVSRLPPSGSKPVAASTCSGKKTPRTGRHGANKENLELNGSILSGGYPGSAPLQRNFSINSVASTYSEFAKDPSLSDSSTVGLQRELSKASKSDATSGILNSTNIQS
00000000000000000000000000000000000000000000000000000000000000000000000000000000000000000000000000000000000000000000000000000000000000000000000000000000000000000000000000000000
00000000000000000000000000000000000000000000000000000000000000000000000000000000000000000000000000000000000000000000000000000000000000000000000000000000000000000000000000000000
00000000000000000000000000000000000000000000000000000000000000001111111111111111111111111111111111111111111111111111111111111111111111111111111111111111111111111111111111111111
11111111111111111111111111111111111111111111111111111111111111111111111111111111111111111111
00000000000000000000000000000000000000000000000000000000000000000000000000000000000000000000000000000000000000000000000000000000000000000000000000000000000000000000000000000000
00000000000000000000000000000000000000000000000000000000000000000000000000000000000000000000000000000000000000000000000000000000000000000000000000000000000000000000000000000000
00000000000000000000000000000000000000000000000000000000000000001111111111111111111111111111111111111111111111111111111111111111111111111111111111111111111111111111111111111111
11111111111111111111111111111111111111111111111111111111111111111111111111111111111111111111
00000000000000000000000000000000000000000000000000000000000000000000000000000000000000000000000000000000000000000000000000000000000000000000000000000000000000000000000000000000
00000000000000000000000000000000000000000000000000000000000000000000000000000000000000000000000000000000000000000000000000000000000000000000000000000000000000000000000000000000
00000000000000000000000000000000000000000000000000000000000000000000000000000000000000000000000000000000000000000000000000000000000000000000000000000000000000000000000000000000
00000000000000000000000000000000000000000000000000000000000000000000000000000000000000000000
>P53865
MTDFDLMNFPFHERLDSPVSENGEIKDGEPIPQNWLNENHVGKSILPLFVNPEDVINCNFSNARDSYEENKSPSMDQMNYARNTSYQESPGLQERPKNEKDKSPIGTDVHKKDVPNFIHSTPRENSSKHFTRANEQASAQPTDEHTSPDISIEDCNGAKIFLQNSLS
KEDFRMLENVILGYQKKVIELGRDNLRQEERANSLQKELEAATKSNDKTLDNKKKIEEQTVLIENLTKDLSLNKEMLEKANDTIQTKHTALLSLTDSLRKAELFEIPIGILFFDLYDSEENSSKLDHILQEKYPNIKGFLCASQQEELSRISQRFKNAKAEAEDLRNELENKKIE
IQTMREKNNTLIGTNKTLSKQNKILCDKFDKLTIDEKEILKGCNEEIKIKLERLNERLGSWEKSKEKYETSLKDKEKMLADAEKKTNTLSKELDNLRSRFGNLEGNTSERITIKNILQSRPDISAEECNFLMVEQIDSANLTTLQNTVKEIVLAVGIPYPKLRRKIPLLAIKLKYE
NIMLSNFAQRLHRQVYSQEMNLKKFTDQAYYDFMSTRRMDSIDHHLERCLDHLYDHILEKMVK
00000000000000000000000000000000000000000000000000000000000000000000000000000000000000000000000000000000000000000000000000000000000000000000000000000000000000000000000000000000
00000000000000000000000000000000000000000000000000000000000000000000000000000000000000000000000000000000000000000000000000000000000000000000000000000000000000000000000000000000
00000000000000000000000000000000000000000000000000000000000000000000000000000000000000000000000000000000000000000000000000000000000000000000000000000000000000000000000000000000
00000000000000000000000000000000000000000001111111111
00000000000000000000000000000000000000000000000000000000000000000000000000000000000000000000000000000000000000000000000000000000000000000000000000000000000000000000000000000000
00000000000000000000000000000000000000000000000000000000000000000000000000000000000000000000000000000000000000000000000000000000000000000000000000000000000000000000000000000000
00000000000000000000000000000000000000000000000000000000000000000000000000000000000000000000000000000000000000000000000000000000000000000000000000000000000000000000000000000000
00000000000000000000000000000000000000000000000000000
00000000000000000000000000000000000000000000000000000000000000000000000000000000000000000000000000000000000000000000000000000000000000000000000000000000000000000000000000000000
00000000000000000000000000000000000000000000000000000000000000000000000000000000000000000000000000000000000000000000000000000000000000000000000000000000000000000000000000000000
00000000000000000000000000000000000000000000000000000000000000000000000000000000000000000000000000000000000000000000000000000000000000000000000000000000000000000000000000000000
00000000000000000000000000000000000000000000000000000
>O60200
MGNIMSASFAPECTDLKTKYDSCFNEWYSEKFLKGKSVENECSKQWYAYTTCVNAALVKQGIKPALDEAREEAPFENGGKLKEVDK
000000000000000000000000000000000000000000000000000000000000000000000011111111111XXXXX
000000000000000000000000000000000000000000000000000000000000000000000000000000000XXXXX
000000000000000000000000000000000000000000000000000000000000000000000000000000000XXXXX
>Q05776
MVLLHKSTHIFPTDFASVSRAFFNRYPNPYSPHVLSIDTISRNVDQEGNLRTTRLLKKSGKLPTWVKPFLRGITETWIIEVSVVNPANSTMKTYTRNLDHTGIMKVEEYTTYQFDSATSSTIADSRVKFSSGFNMGIKSKVEDWSRTKFDENVKKSRMGMAFVIQKLEE
ARNPQF
00000000000000000000000000000000000000000000000000000000000000111111111100000000000000000000000000000000000000000000000000000000000000000000000000000000000000000000000000000
00000000000000000000000000000000000000000000000000000000000000000000000000000000000000000000000000000000000000000000000000000000000000000000000000000000000000000000000000000
00000000000000000000000000000000000000000000000000000000000000000000000000000000000000000000000000000000000000000000000000000000000000000000000000000000000000000000000000000
>K7J0R2
MWSPAILLLLIGATFANQQNGWTNGKQYTYAINSRTIATFNQQSKYLSGIVIEAYLTVQPNGEDTLRAKIWQPRYSPIHTQLENGWDSEIPQNLINLQTFPLSGKPFEIKTKNGVVRDLIVDKDVPTWEVNVLKGIVSQLQIDTSGENVKKSKRNQLPEENQPFAFFK
AMEDSVGGKCEVLYDISPLPEQVLQNKPELAPMPELREDGDMISLVKTKNYSNCEQRAGYHFNINGRNAWEPGSNENRKYLSRSSVSRVIISGNLRKYTIQSSVTTNKVVHHADNQEENQQGMVASRMNLTLHKVEDMSEPMESPVNPQSTGNLVYNYNSPIDS
ISARRPNKYNQKGRSDEKNKNSDESDSESDSDGSVFDNNDDSYLQPKPKLTDAPLSPLLPFFIGNNGNSILKNKKVDAVKSATSIAQEIGNEMQNPDIMFAEQTLEKFTILSKLIRTMNSEQIASVQRSLYERAQSLNQLKQNNPEQLSRRNAWVAFRDAVAQAGTG
PALVNIKQWVQNKQIEGTEATHVIDTLAKSVRIPTPEYMDTYFELIKMEEVKRELIVRDAAVLSFADLIRHAVVNKKSAHNHYPVHAFGRLLPKNFRQLHEKYIPYLEEELLKAVDAGDSRRIHTYTIALGKTAHPRVLAVFEPYLEGKKPISPYQRLVMVLSLNKLASIFP
KVGRSVLYKIYSNTADYHEIRTAAVYLLMQSNPSASMLQRMAEFTNYDTSKYVNSAVKSTIESLAQLHDNHEYQGLLDSARAAQPLLTSESYGPQYSKQMFFNLRNPLTQSDYFIQASTIGSEDSIIPKGVYVITIPTYNGMKMPKIEIGGEVSSLKNLWNFVQQRISN
SQRSDSNEKPENQKYSPENLAKLLGIYGEETEQIEGFAFINDKFANHFLTFDNHTLEKIPGMLRQLAEDMKQGRSFDATKLKNFEVTISFPTETGFPFRFTVKNPTITSVSGVSHLKTTSGSGSRSEWPKASLSGNVRIVYGLQTQKRLGFVTPFEHQEYMVGIDKDMQ
VYLPVRSEIEYDVNKGETRLRIQPNENLDEFKIIQYRTQPFTSKHDILNLEPITKDSNTATVHKNRATSSQIELNDNNNKQRLQFNWERQMRHLEEEIGNSYNKRQNAMEAMCKLTQSISSMFYLNSVDSEYQKYSVKVSPGSDMSAEMRISHDSMITENSENTDNS
ESWSPNAKTVHLERSLSEQERKQTLLKEASKNINSAEANVVDISLQLNGDMQSSVALTAAFADSNVDRKSRALLYASVETKGGQDYHVSAGFEGKNPNIESLDFEEILKANDRREYDLNVHYGIGTNENDENKQNRIKVRGEIKQTEERKKQIRQSHDARVCMKQQ
SLHGDKMTSACKRINKRASLADAGDFTVTFPNKSPMREIVMSAWDAAERMTQSVSHSWKNRMIKEEDNKVKVTFEMSPNDEKVDVTVKTPEGQIQLNNIKVALISNKNNGNVKDNRNEDDEELNKLNDNVCQLDKTQARTFDNHRYPLQLGSCWHIAMTPY
PKHDPDTPSKKLEIPENMQVSILTRENENGQKELKITLGESLIELSASGPRQTHAKVNGNKVHYSKHKSYKEKKHGKVLFELFELSDESLKLVSKKYDIEIVYDGYRAQIETGERYRDSVRGLCGNNDGESMNDQQTPKGCLLQKPEEFSATYALTNDDQCQGPAIRNA
DEAKKSQCSYQTIRPGNVISEKEAGRETELSQDSDGAKHCMTHRTKIIRSKNEICFSLRPIPTCLSKCSPSSIKSKAIPFHCVAKNSASQKVAERVEKGANPDLTQKSVSKTLTEQLPINCKA
00000000000000000000000000000000000000000000000000000000000000000000000000000000000000000000000000000000000000000000000000000000000000000000000000000000000000000000000000000000
00000000000000000000000000000000000000000000000000000000000000000000000000000000000000000000000000000000000000000000000000000000000000000000000000000000000000011111111111111111
11111111111111111111111111111111111111111111111111111111111000000000000000000000000000000000000000000000000000000000000000000000000000000000000000000000000000000000000000000000
00000000000000000000000000000000000000000000000000000000000000000000000000000000000000000000000000000000000000000000000000000000000000000000000000000000000000000000000000000000
00000000000000000000000000000000000000000000000000000000000000000000000000000000000000000000000000000000000000000000000000000000000000000000000000000000000000000000000000000000
00000000000000000000000000000000000000000000000000000000000000000000000000000000000000000000000000000000000000000000000000000000000000000000000000000000000000000000000000000000
00000000000000000000000000000000000000000000000000000000000000000000000000000000000000000000000000000000000000000000000000000000000000000000000000000000000000000000000000000000
00000000000000000000000000000000000000000000000000000000000000000000000000000000000000000000000000000000000000000000000000000000000000000000000000000000000000000000000000000000
00000000000000000000000000000000000000000000000000000000000000000000000000000000000000000000000000000000000000000000000000000000000000000000000000000000000000000000000000000000
00000000000000000000000000000000000000000000000000000000000000000000000000000000000000000000000000000000000000000000000000000000000000000000000000000000000000000000000000000000
00000000000000000000000000000000000000000000000000000000000000000000000000000000000000000000000000000000000000000000000000000000000000000000000000000000000000000000000000000000
00000000000000000000000000000000000
00000000000000000000000000000000000000000000000000000000000000000000000000000000000000000000000000000000000000000000000000000000000000000000000000000000000000000000000000000000
00000000000000000000000000000000000000000000000000000000000000000000000000000000000000000000000000000000000000000000000000000000000000000000000000000000000000000000000000000000
00000000000000000000000000000000000000000000000000000000000000000000000000000000000000000000000000000000000000000000000000000000000000000000000000000000000000000000000000000000
00000000000000000000000000000000000000000000000000000000000000000000000000000000000000000000000000000000000000000000000000000000000000000000000000000000000000000000000000000000
00000000000000000000000000000000000000000000000000000000000000000000000000000000000000000000000000000000000000000000000000000000000000000000000000000000000000000000000000000000
00000000000000000000000000000000000000000000000000000000000000000000000000000000000000000000000000000000000000000000000000000000000000000000000000000000000000000000000000000000
00000000000000000000000000000000000000000000000000000000000000000000000000000000000000000000000000000000000000000000000000000000000000000000000000000000000000000000000000000000
00000000000000000000000000000000000000000000000000000000000000000000000000000000000000000000000000000000000000000000000000000000000000000000000000000000000000000000000000000000
00000000000000000000000000000000000000000000000000000000000000000000000000000000000000000000000000000000000000000000000000000000000000000000000000000000000000000000000000000000
00000000000000000000000000000000000000000000000000000000000000000000000000000000000000000000000000000000000000000000000000000000000000000000000000000000000000000000000000000000
00000000000000000000000000000000000000000000000000000000000000000000000000000000000000000000000000000000000000000000000000000000000000000000000000000000000000000000000000000000
00000000000000000000000000000000000
00000000000000000000000000000000000000000000000000000000000000000000000000000000000000000000000000000000000000000000000000000000000000000000000000000000000000000000000000000000
00000000000000000000000000000000000000000000000000000000000000000000000000000000000000000000000000000000000000000000000000000000000000000000000000000000000000000000000000000000
00000000000000000000000000000000000000000000000000000000000000000000000000000000000000000000000000000000000000000000000000000000000000000000000000000000000000000000000000000000
00000000000000000000000000000000000000000000000000000000000000000000000000000000000000000000000000000000000000000000000000000000000000000000000000000000000000000000000000000000
00000000000000000000000000000000000000000000000000000000000000000000000000000000000000000000000000000000000000000000000000000000000000000000000000000000000000000000000000000000
00000000000000000000000000000000000000000000000000000000000000000000000000000000000000000000000000000000000000000000000000000000000000000000000000000000000000000000000000000000
00000000000000000000000000000000000000000000000000000000000000000000000000000000000000000000000000000000000000000000000000000000000000000000000000000000000000000000000000000000
00000000000000000000000000000000000000000000000000000000000000000000000000000000000000000000000000000000000000000000000000000000000000000000000000000000000000000000000000000000
00000000000000000000000000000000000000000000000000000000000000000000000000000000000000000000000000000000000000000000000000000000000000000000000000000000000000000000000000000000
00000000000000000000000000000000000000000000000000000000000000000000000000000000000000000000000000000000000000000000000000000000000000000000000000000000000000000000000000000000
00000000000000000000000000000000000000000000000000000000000000000000000000000000000000000000000000000000000000000000000000000000000000000000000000000000000000000000000000000000
00000000000000000000000000000000000
>C4PB33

MWLPLTVLLLAGIVSADYDHGWHVNNEYIYLVRSRTLVNLNELSDQHTGILMKALLTIQVKDPQMLTAKVSQSQYARILKSLPEGWDAEISDQMLELRDMPLSGKPFNIKLKHGVIRDIIVDRTIPTWEVNILKSIVSQLQVDSLGENAIRTSEMQIPTDEHPYGMFR
AMEDSVGGKCEVLYDITPLPEQNVYVQPELVPVPDLKREGQYIDIRKSKNFNKCDQRMNYQFGITGNKYWEAGSNKNGKFFSQSAMSRIIISGTLKSYTIQSAVTTNIMYISPRFYDHEHGMVTSRMNLTLAAVKKITNPLPKPNSPESTGNLVYIYSNPYSDMEERR
VGKVVEDSDNMMLSDSISSISSSEEATKGQNYRSLSSDSSSSSSFSNSEEDHYWQPKPTMEDAPQNPLSSIYIGYMGKYIGKSNEVDVVEKSKELISQIANEMEDPNDVYENHILEKYTVLCNLLRTMNKEQMLQVDKHVRLSPHELKSMDKTQVLKQNAWTVLKS
AVAQAGTGPAFLVIKNWIEMKEVDSKRGADLLAKLPKTARAPTAEYIMEFFKLATGETVKNDLALNSSAIIAFSELVYNAQVSRKGLHNHYPVHTYGRLTPKHNKAVTEYYIPYLETELKKAVESGQSTVIQTYIMALGNIGHPKILPVLEPYLEGKVRMTVFQRTLMVS
TLAKLAENFPKLARSILYKIYLNTMEEHQVRCTAVFILMQTDPPTLMLQRMAEFTKIDKNKYVNSAVKSTLESLANLKDAEYQTLAKKARVAKNLLSPSDYSYHYSRGYITESIMDEGNIISHMMLKYIGSDDSLIPNAIYYAVFSTYGDFKLPPFEVVTMVSSIRSILELNT
SPEEKERIKLAAEKIAEQLNIISDEFIPLEGNIMWNGKYGARFLPFDQSCLSLLRELLLMYLKGESEGKLINRLGSYDITYGFPTETGLPFVYNFELPMLLKITGAMNNEAKNLKTIKMKTDFRILYAMKIQGRMSFVTPFEHQEYIAGVDVDFNLHLPMKLTLDIDLPKQSF
EIKIWPLKGEDKAQLLHYSVVPYVANHNILSLRPLLTEKTAQMIIPDDIYLDTISDSDLLKVVLETDKSYDYKNWLDLDIDDLFNVVIAPWSSDNDNYRKVDVFLNLKREQVAPFVLKMSYDFMEMAPTAEDAKLWTPKATAMEPSDKHPDSEVRRKQWMDEAAK
GVKSAKSQVIDIRLEVPVTSEETIMNVITVATSNSEIEKKGRTLVYWNFETLFEVCAASQTKVTPDNTVFYEEVAQLKPKVEFNADFRIGKVCSTGEQLNINSVATQSKELRERIKNSSLIKTCEKQMQQGNKILRACQNAAAISMILDQITISVDFQSQHFINFITKALNV
MMNIDYLNEIAYIHTELSDLKVAGKRKIDIVANLTNDFESADVLISTPNMNIYANDIDLSALEISAEDVLMAADEDMDIQNLLYNEDEPACILDKTRAQTFDSKEYPLRLGKCWHVVVTTYPRVNPNNPNEKMRMHKFDSVSILTRDMENGQREMKVLLGDKELKF
VPTSSQPKIFVNEQLIKVTKDMSWQEKMDDEVLYEIFQINDHSVGLVSDEYELNLVYDGKRIMMKAGDKFRKAIRGLCGNYDGKLINDFMAPEKCVFRKPEQFIASYALTKEECESEFFENSKLLMDQDCVYEEKNHLSNVISDKESGRRDIEESNWGYHKQTKSKQ
CTIMKAHMKEIEHMICFTIRQVPSCAPGCWATEMKPKDYQYHCMKRNAASLALKARIEKGAKPDLSQKSVTLTEPINVPLACKA
XXXXXXXXXXXXXXXXXXXXXXXXXXXXXXXXXXXXXXXXXXXXXXXXXXXXXXXXXXXXXXXXXXXXXXXXXXXXXXXXXXXXXXXXXXXXXXXXXXXXXXXXXXXXXXXXXXXXXXXXXXXXXXXXXXXXXXXXXXXXXXXXXXXXXXXXXXXXXXXXXXXXXXX
XXXXXXXXXXXXXXXXXXXXXXXXXXXXXXXXXXXXXXXXXXXXXXXXXXXXXXXXXXXXXXXXXXXXXXXXXXXXXXXXXXXXXXXXXXXXXXXXXXXXXXXXXXXXXXXXXXXXXXXXXXXXXXXXXXXXXXXXXXXXXXXXXXXXXXXXXXXXXXXXXXXXXXXX
XXXXXXXXXXXXXXX1111111111111111111111111111111111111100000000000000000000000000000000000000000000000000000000000000000000000000000000000000000000000000000000000000000000000000
[illegible]
00000000000000000000
XXXXXXXXXXXXXXXXXXXXXXXXXXXXXXXXXXXXXXXXXXXXXXXXXXXXXXXXXXXXXXXXXXXXXXXXXXXXXXXXXXXXXXXXXXXXXXXXXXXXXXXXXXXXXXXXXXXXXXXXXXXXXXXXXXXXXXXXXXXXXXXXXXXXXXXXXXXXXXXXXXXXXXXXXXX
XXXXXXXXXXXXXXXXXXXXXXXXXXXXXXXXXXXXXXXXXXXXXXXXXXXXXXXXXXXXXXXXXXXXXXXXXXXXXXXXXXXXXXXXXXXXXXXXXXXXXXXXXXXXXXXXXXXXXXXXXXXXXXXXXXXXXXXXXXXXXXXXXXXXXXXXXXXXXXXXXXXXXXXXXX
XXXXXXXXXXXXXXX[illegible]
[illegible]
00000000000000000000
XXXXXXXXXXXXXXXXXXXXXXXXXXXXXXXXXXXXXXXXXXXXXXXXXXXXXXXXXXXXXXXXXXXXXXXXXXXXXXXXXXXXXXXXXXXXXXXXXXXXXXXXXXXXXXXXXXXXXXXXXXXXXXXXXXXXXXXXXXXXXXXXXXXXXXXXXXXXXXXXXXXXXXXXXXX
XXXXXXXXXXXXXXXXXXXXXXXXXXXXXXXXXXXXXXXXXXXXXXXXXXXXXXXXXXXXXXXXXXXXXXXXXXXXXXXXXXXXXXXXXXXXXXXXXXXXXXXXXXXXXXXXXXXXXXXXXXXXXXXXXXXXXXXXXXXXXXXXXXXXXXXXXXXXXXXXXXXXXXXXXX
XXXXXXXXXXXXXXX[illegible]
[illegible]
00000000000000000000
>O17428
MWCPLFLVLLAGAATAEHLQAWKTDTEYQYAVRGRTLSALHDVADQYSGIIMRALLTIQPKSDGTLEAKVTKARYAQIHTKLPGGWNSEIPDHKLEMKQFPMSEKVFEIKMKHGVVRDLIVDKDVPTWEVNVLKSIVSQLQVDTQGENLMASKYNQEPEDEGVT
AMFKTMEDTVGGRCEVLYDINPLPEFVLKQRPELVPMPDIRGDGEIIDIVKTKNYSNCEQRSGYHFGIPGTNKWEPSSGASGNFLSRSSVSRVIVTGNLKSFTIQSSVSTNKVILSPNFHENRKGMVSSRVNVTLVKIGAPSTGDWSTPANPESTGNLVYNYNNPFAG
VGEDRRASRPQDNRNSEEKVRSLYNLYRRNRINDNDDDSSASDSSKSAKSLESNEEQLYWQPKPTLNEAPAMAMLPFFIGNHGKSIHKTDEIEPITMAKTLASQIGSDFQDPNSITDEQTLEKFTLLVRIIRTMSTQQIAEAERDLYQSNNEIDPHDESQSVRRGTWA
AFRDAVAQAGTAPALVTITLWIKQKKIRGVEAASVVGVLAKTARTPTREYIDVFFELATMPETIHEPFLNTTALFTFSELVYRSQMDNPSSHTRYPVHTFGRFSDKINPEVFRMYVPYLAEKLKTSIEKNENAKAHTIVSLGNIAHPKILAVFEPYLEGKMPASTFQRLL
MVISLNKLATLKPKLARGVFYRIYKNTGEAHQLRCAAVTALMSTNPPASMLQRMAEFTNEDHSKHVNAAVKSAIESASELETPQWQELAENARNAKPLLNKESYGFEYSKLYLTDFIEREMNVAYQAQASFISSDDSYVPEALFVKARAIFGGFTFPRTSAGAMVSS
AKDLLSAFEDAFKGEKTSHKHSHVEFSPENVAKLLKIKSEPNEDVEGAFMLQSAYNNKFMSLDKNTLKNLPELSSKALKALKDGHHFNTARIESYEMTMSFPMESGFPFVYTMKVPSMLKLAGSVKGDSNEDSLDGHVHLRAVYSVQFQSKLGFVTPFEHQHYMA
GINKNYQAYVPLRVNVNYEDSDKRVTLKIQPIEQNEKFKLWHHSVVPFTSRHDILKMSPVLKDKETQKIITEEVSKNEYNFGSDKHGVKFTVLAEADNDNSPLNFEDAKWERNPLTQLFHLFVEQPVYHKVDIFMQPSKSSEQSIILSTAFETLQDDTTKYDEDRSKHI
KAEAPKVENKKLNDPQRRRKLLKEAAKGIAAADAFAVDIGLEYPGQAPVQAFATLAFASSEVDEKSRGLVYWRLTDAGNDFEFESCTSLEARSPRTSDFGKDMLDQPEFRSRKFDIDLRYGQTCEKGYKVEIQGSQEQTEKYRDTIKRLPTAIECIRAAKKGMKSLPAC
HDVSVKMTMLDKTRITIKYDKDSREMYQLFDDYLDSMSDADDDLKSSKKRLNAVKDDDKAYDDTVKIAVDLSPNDNNGRFSYESDFVKLEARDVPINGIGPLVNVHPDLETAERLDMDMSDEPVRQRICVLDSHSATTFDESTYPINLGKCWHVVMTTFPKTNKN
SGSAEPIDEDMALSILVRDAGDKKKDIKVTLGDKELQFSHADSKNKVTLDGKKVDLSEKRSYRHKNGKDIDFEVLQRPDGTLGLVSSKYDIDAWYDGQRVQIKASGKYRSDIRGLCGNFDGEPDNDFTSPKDCVLLKPEEFAASYALTTKDCHGSALEHARKASQAVC
SQKSPRPGNVVSDRDAGRKYSENSNWGYHSRQNSDDEQGKNDSSDHKRCNTLRTKVIEEEDQICFSLRPLPTCAEGCTANRTKPKVMPMHCMPKNIAAERMADRIKQGANPDFSQKSYTKKNGFDIPVGCHAA
XXXXXXXXXXXXXXXXXXXXXXXXXXXXXXXXXXXXXXXXXXXXXXXXXXXXXXXXXXXXXXXXXXXXXXXXXXXXXXXXXXXXXXXXXXXXXXXXXXXXXXXXXXXXXXXXXXXXXXXXXXXXXXXXXXXXXXXXXXXXXXXXXXXXXXXXXXXXXXXXXXXXXXXXXX
XXXXXXXXXXXXXXXXXXXXXXXXXXXXXXXXXXXXXXXXXXXXXXXXXXXXXXXXXXXXXXXXXXXXXXXXXXXXXXXXXXXXXXXXXXXXXXXXXXXXXXXXXXXXXXXXXXXXXXXXXXXXXXXXXXXXXXXXXXXXXXXXXXXXXXXXXXXXXXXXXXXXXXXXX
XXXXXXXXXX[illegible]
[illegible]
XXXXXXXXXXXXXXXXXXXXXXXXXXXXXXXXXXXXXXXXXXXXXXXXXXXXXXXXXXXXXXXXXXXXXXXXXXXXXXXXXXXXXXXXXXXXXXXXXXXXXXXXXXXXXXXXXXXXXXXXXXXXXXXXXXXXXXXXXXXXXXXXXXXXXXXXXXXXXXXXXXXXXXXXXX
XXXXXXXXXXXXXXXXXXXXXXXXXXXXXXXXXXXXXXXXXXXXXXXXXXXXXXXXXXXXXXXXXXXXXXXXXXXXXXXXXXXXXXXXXXXXXXXXXXXXXXXXXXXXXXXXXXXXXXXXXXXXXXXXXXXXXXXXXXXXXXXXXXXXXXXXXXXXXXXXXXXXXXXXXX
XXXXXXXXXX[illegible]
[illegible]
XXXXXXXXXXXXXXXXXXXXXXXXXXXXXXXXXXXXXXXXXXXXXXXXXXXXXXXXXXXXXXXXXXXXXXXXXXXXXXXXXXXXXXXXXXXXXXXXXXXXXXXXXXXXXXXXXXXXXXXXXXXXXXXXXXXXXXXXXXXXXXXXXXXXXXXXXXXXXXXXXXXXXXXXXX
XXXXXXXXXXXXXXXXXXXXXXXXXXXXXXXXXXXXXXXXXXXXXXXXXXXXXXXXXXXXXXXXXXXXXXXXXXXXXXXXXXXXXXXXXXXXXXXXXXXXXXXXXXXXXXXXXXXXXXXXXXXXXXXXXXXXXXXXXXXXXXXXXXXXXXXXXXXXXXXXXXXXXXXXX
XXXXXXXXXX[illegible]
[illegible]
>Q98148
MAPPGMRLRSGRSTGAPLTRGSCRKRNRSPERCDLGDDLHLQPRRKHVADSIDGRECGPHTLPIPGSPTVFTSGLPAFVSSPTLPVAPIPSPAPATPLPPPALLPPVTTSSSPIPPSHPVSPGTTDTHSPSPALPPTQSPESSQRPPLSSPTGRPDSSTPMRPPPSQQTTP
PHSPTTPPPEPPSKSSPDSLAPSTLRSLRKRRLSSPQGPSTLNPICQSPPVSPPRCDFANRSVYPPWATESPIYVGSSSDGDTPPRQPPTSPISIGSSSPSEGSWGDDTAMLVLLAEIAEEASKNEKECSENNQAGEDNGDNEISKESQVDKDDNDNKDDEEEQETDEE
DEEDDEEDDEEDDEEDDEEDDEEDDEEDDEEDDEEDDEEDDEEDDEEEDEEEDEEEDEEEEDEEDDDDEDNEDEEDDEEEDKKEDEEDGGDGNKTLSIQSSQQQQEPQQQEPQQQEPQQQEPLQEPQQQEPQQQEPQQQEPLQEPQQQEPQQQEPLQEP
QQQEPQQQEPQQQEPQQQEPQQQEPQQQEPQQQEPQQQEPQQQEPQQQEPQQQEPQQQEPQQREPQQREPQQREPQQREPQQREPQQREPQQREPQQREPQQREPQQQDEQQQDEQQQDEQQQDEQQQDEQQQDEQQQDEQQQDEQQQDEQQQDEQQQ
DEQQQDEQQQDEQQQDEQQQDEQQQDEQQQDEQQQDEQQQDEQQQDEQQQDEQQQDEQEQQDEQEQQDEQEQQDEQEQQDEQQQDEQQQQDEQQQDEQQQDEQQQQDEQQQQDEQQQQDEQQQQDEQEQQEEQEQQEEQEQQEEQEQLEEQEQLEDQEQLEEQEQLEEQEQE
LEEQEQLEEQEQLEEQEQLEEQEQLEEQEQLEEQEQLEEQEQVEEQEQEQEEQEQLEEVEEQEQEEQEQLEEVEEQEQEEQEQLEEVEEQEQEEQEQLEEVEEQEQQGVEQQEQETVEEPIILHGSSSEDEMEVDYPVVSTHEQIASS
PPGDNTPDDDPQPGPSREYRYVLRTSPPHRPGVRMRRVPVTHPKKPHPRYQQPPVPYRQIDDCPAKARPQHIFYRRFLGKDGRRDPKCQWKFAVIFWGNDPYGLKKLSQAFQFGGVKAGPVSCLPHPGPDQSPITYCVYVYCQNKDTSKKVQMARLAWEASH
PLAGNLQSSIVKFKKPLPLTQPGENQGPGDSPQEMT
[illegible]
>K7ZP88
MNKIYKVIWNATLLAWVAVSELAKGKTKSTTSKSKAKSLSSSVIVGGIILTTPLSLIAATVQVGGGTNSGTTATASTNCADLYNYQNPENSGSGAAGNYNAGNPSVCSIAIGENAQGGTSGTGGSPGIAIGGNSKATGGLSVAIGGYAQATNVGSIALGTAALSSGFN
SLAISRQAAATNNYSIAIGTTSVSKGVGSIAMGHSTNASGDQSIAIGSSDAVNSATATTTYDGTTNTQASGSKSIAIGASAKASTNNSIALGAGSVTSAQSGNSYLTGVGASATNGVSVGTSTATRRIQNVADGSAASDAVTVAQLDKAYDDTNGRLAAALGTGSG
AAYNAANNTYTAPTNIGGTGKNTIDDAIKATQRSVVAGSNIVVTPTTASDGSISYSVATSATPTFTSITVNNAPTAGTDATNKTYVDSKAAASRTEVAAGSNVSGVVKTTGANGQDVYTVNANGTTASAGSSAVTVTPGTKDANNVTDYKVDLSATTKTDIQKGVD
AKNAVDTAGLKFKGDTATTSNTKKLGDTVSITGDTNISTVATTDGVQVKLNPNLDLGATGSVKTGNTTINNAGVTADQVTVGGVVINNTSGINAGGKAITNVAAPTNNTDAANKKYVDDAGTALTNLGFGLKAQDGTTVNKKLGEAVDIVGSNSNISTKVNAGK
VEVALSNTLDLGTTGSVTTGSTVINNAGVTATQVTANKVTINNAPTAGTDATNKTYVDSKAAASRTEVAAGSNVSGVVKTTGANGQDIYAVNANGTTASAGSSAVTVTPGTKDANNVTDYKVDLSATTKTDIQKGVDAKNAVDTAGLKFKGDTATTSNTKKLGD
TVSITGDTNISTVATTDGVQVKLNPNLDLGATGSVKTGNTTINNAGVTADQVTVGGVVINNTSGINAGGKAITNVAAPTNNTDAANKKYVDDAGTALTNLGFGLKAQDGTTVNKKLGEAVDIVGSNSNISTKVNAGKVEVALSNTLDLGTTGSVTTGSTVINNAG
VTATQVTANKVTVNNAPTAGTDATNKTYVDSKAAASRTEVAAGSNVSGVVKTTGANGQDVYTVNANGTTASAGSSAVTVTPGTKDANNVTDYKVDLSATTKTDIQKGVDAKNAVDTAGLKFKGDTATTSNTKKLGDTVSITGDTNISTVATTDGVQVKLNPNL
DLGATGSVKTGNTTINNAGVTADQVTVGGVVINNTSGINAGGKAITNVAAPTNNTDAANKKYVDDAGTALTNLGFGLKAQDGTTVNKKLGEAVEVVGADSNITTKVAGGQVAIELNKNLNNLTGITVNDGTNGTNGSTVIGKDGISVKDGSGNTIAGVDNTALT

VKDGSGNTETSINQAINTLNAAQGETDKFAVKYDKNADGSVNYNNITLAGTTASSTQDATTGKITTTGGTSLNNVASAGDYKDVANASKGVNAGDLNNAVVDATNAATSKGFALQAADGAKVQKNLGEAVEVVGADSNITTKVAGGQVAIELNKNLNNLTGIT
VNDGTNGTNGSTVIGKDGISVKDGSGNTIAGVDNTALTVKDGSGNTETSINQAINTLNAAQGETDKFAVKYDKNTDGSTNYNSITAGNGNGTAATIGTDTAGNSVVTSGGTKISNVANGVNASDAVNKGQLDSLSTGLTNTGFGLKAADGNTVNKKLGEAVDVV
GADSNITTKVAGGQVAIELNKNLNNLTGITVNDGTNGTNGSTVIGKDGISIKDGSGNTIAGVDNTALTVKDGSGNTETSINQAINTLNAAQGETDKFAVKYDKNADGSANYNNITLAGTTASSTQDATTGKITTTGGTSLNNVASAGDYKDVANASKGVNAGDLN
NAVVDATNAATSKGFALQAADGAKVQKNLGEAVEVVGADSNITTKVVGGQVAIELNKNLNNLTGITVNDGTNGTNGSTVIGKDGISVKDGSGNTIAGVDNTALTVKDGSGNTETSINQAINTLNAAQGETDKFAVKYDKNADGSVNYNNITLAGTTASSTQDAT
TGKITTTGGTSLNNVASAGDYKDVANASKGVNAGDLNNAVVDATNAATSKGFALQAADGAKVQKNLGEAVEVVGADSNITTKVAGGQVAIELNKNLNNLTGITVNDGTNGTNGSTVIGKDGISVKDGSGNTIAGVDNTALTVKDGSGNTETSINQAINTLNAA
QGETDKFAVKYDKNADGSVNYNNITLAGTTASSTQDATTGKITTTGGTSLNNVASAGDYKDVANASKGVNAGDLNNAVVDATNAATSKGFALQAADGAKVQKNLGEAVEVVGADSNITTKVAGGQVAIELNKNLNNLTGITVNDGTNGTNGSTVIGKDGISVK
DGSGNTIAGVDNTALTVKDGSGNTETSINQAINTLNAAQGETDKFAVKYDKNADGSANYNNVTLAGTNGTIISNVKAGAVTSTSTDAINGSQLYGVANSVKNAIGGSTTIDATTGAITTTNIGGTGSNTIDGAISSIKDSATKAKTTVSAGDNVVVTSGTNADGSTN
YEVATAKDVNFDKVTVGSVVVDKSSNTIKGLSNTTWNGTAVSGQAATEDQLKTVSDAQGETDKFAVKYDKNADGSANYNSITAGNGNGTAATIGTDTAGNSVVTSGGTKISNVANGVNASDAVNKGQLDSLSTGLTNTGFGLKAADGNTVNKKLGEAVDVVG
ADSNITTKVAGGQVAIELNKNLNNLTGITVNDGTNGTNGSTVIGKDGISIKDGSGNTIAGVDNTALTVKDSSGNTETSINQAINTLNAAQGETDKFAVKYDKNADGSVNYNNVTLAGTNGTIIRNVKAGAVTSTSTDAINGSQLYDIANSVKNAIGGSTTRDVTTGAI
TTTNIGGTGSNTIDGAISSIKDSATKAKTTISAGDNVVVTSGTNADGSTNYEVATAKDVNFDKVTVGNVVVDKANDTIQGLSNKDLNSTDFATKGRAATEEQLKAVITSNITEVVDGNGNKVNIIDQVVNTKPDNKNQDSLFLTYDKQGQETTDRLTIGQTVQKMN
TDGIKFFHTNADTSKGDLGTTNDSSAGGLNSTAIGVNAIVANGADSSVALGHNTKVNGKQSIAIGSGAEALGNQSISIGTGNKVTGDHSGAIGDPTIVNGANSYSVGNNNQVLTDDTFVLGNNVTKTIAGSVVLGNGSAATTGAGEAGYALSVATNADKAAITKTT
SSTGAVAVGDASSGIYRQITGVAAGSVDSDAVNVAQLKAVGNQVVTTQTTLVNSLGGNAKVNADGTITGPTYNVAQGNQTNVGDALTALDNAINTAATTSKSTVSNGQNIVVSKSKNADGSDNYEVSTAKDLTVDSVKAGDTVLNNAGITIGNNAVVLNNTGL
TISGGPSVTLAGIDAGNKTIQNVANAVNATDAVNKGQLDSAINNVNNNVNELANNAVKYDDASKDKITLGGGATGTTITNVKDGTVAQGSKDAVNGGQLWNVQQQVDQNTTDISNIKNDINNGTVGLVQQAGKDAPVTVAKDTGGTTVNVAGTDGNRVVT
GVKEGAVNATSKDAVNGSQLNTTNQAVVNYLGGGAGYDNITGSFTAPSYTVGDSKYNNVGGAIDALNQADQALNSKIDNVSNKLDNAFRITNNRIDDVEKKANAGIAAAMALESAPYVPGKYTYAAGAAYHGGENAVGVTLRKTADNGRWSITGGVAAASQG
DASVRIGISGVID
00000000000000000000000000000000000000000000000000000000000000000000000000000000000000000000000000000000000000000000000000000000000000000000000000000000000000000000000000000000000000
00000000000000000000000000000000000000000000000000000000000000000000000000000000000000000000000000000000000000000000000000000000000000000000000000000000000000000000000000000000000000
00000000000000000000000000000000000000000000000000000000000000000000000000000000000000000000000000000000000000000000000000000000000000000000000000000000000000000000000000000000000000
00000000000000000000000000000000000000000000000000000000000000000000000000000000000000000000000000000000000000000000000000000000000000000000000000000000000000000000000000000000000000
00000000000000000000000000000000000000000000000000000000000000000000000000000000000000000000000000000000000000000000000000000000000000000000000000000000000000000000000000000000000000
00000000000000000000000000000000000000000000000000000000000000000000000000000000000000000000000000000000000000000000000000000000000000000000000000000000000000000000000000000000000000
00000000000000000000000000000000000000000000000000000000000000000000000000000000000000000000000000000000000000000000000000000000000000000000000000000000000000000000000000000000000000
00000000000000000000000000000000000000000000000000000000000000000000000000000000000000000000000000000000000000000000000000000000000000000000000000000000000000000000000000000000000000
00000000000000000000000000000000000000000000000000000000000000000000000000000000000000000000000000000000000000000000000000000000000000000000000000000000000000000000000000000000000000
00000000000000000000000000000000000000000000000000000000000000000000000000000000000000000000000000000000000000000000000000000000000000000000000000000000000000000000000000000000000000
00000000000000000000000000000000000000000000000000000000000000000000000000000000000000000000000000000000000000000000000000000000000000000000000000000000000000000000000000000000000000
00000000000000000000000000000000000000000000000000000000000000000000000000000000000000000000000000000000000000000000000000000000000000000000000000000000000000000000000000000000000000
00000000000000000000000000000000000000000000000000000000000000000000000000000000000000000000000000000000000000000000000000000000000000000000000000000000000000000000000000000000000000
00000000000000000000000000000000000000000000000000000000000000000000000000000000000000000000000000000000000000000000000000000000000000000000000000000000000000000000000000000000000000
00000000000000000000000000000000000000000000000000000000000000000000000000000000000000000000000000000000000000000000000000000000000000000000000000000000000000000000000000000000000000
00000000000000000000000000000000000000000000000000000000000000000000000000000000000000000000000000000000000000000000000000000000000000000000000000000000000000000000000000000000000000
00000000000000000000000000000000000000000000000000000000000000000000000000000000000000000000000000000000000000000000000000000000000000000000000000000000000000000000000000000000000000
00000000000000000000000000000000000000000000000000000000000000000000000000000000000000000000000000000000000000000000000000000000000000000000000000000000000000000000000000000000000000
00000000000000000000000000000000000000000000000000000000000000000000000000000000000000000000000000000000000000000000000000000000000000000000000000000000000000000000000000000000000000
00000000000000000000000000000000000000000000000000000000000000000000000000000000000000000000000000000000000000000000000000000000000000000000000000000000000000000000000000000000000000
00000000000000000000000000000000000000000000000000000000000000000000000000000000000000000000000000000000000000000000
00000000000000000000000000000000000000000000000000000000000000000000000000000000000000000000000000000000000000000000000000000000000000000000000000000000000000000000000000000000000000
00000000000000000000000000000000000000000000000000000000000000000000000000000000000000000000000000000000000000000000000000000000000000000000000000000000000000000000000000000000000000
00000000000000000000000000000000000000000000000000000000000000000000000000000000000000000000000000000000000000000000000000000000000000000000000000000000000000000000000000000000000000
00000000000000000000000000000000000000000000000000000000000000000000000000000000000000000000000000000000000000000000000000000000000000000000000000000000000000000000000000000000000000
00000000000000000000000000000000000000000000000000000000000000000000000000000000000000000000000000000000000000000000000000000000000000000000000000000000000000000000000000000000000000
00000000000000000000000000000000000000000000000000000000000000000000000000000000000000000000000000000000000000000000000000000000000000000000000000000000000000000000000000000000000000
00000000000000000000000000000000000000000000000000000000000000000000000000000000000000000000000000000000000000000000000000000000000000000000000000000000000000000000000000000000000000
00000000000000000000000000000000000000000000000000000000000000000000000000000000000000000000000000000000000000000000000000000000000000000000000000000000000000000000000000000000000000
00000000000000000000000000000000000000000000000000000000000000000000000000000000000000000000000000000000000000000000000000000000000000000000000000000000000000000000000000000000000000
00000000000000000000000000000000000000000000000000000000000000000000000000000000000000000000000000000000000000000000000000000000000000000000000000000000000000000000000000000000000000
00000000000000000000000000000000000000000000000000000000000000000000000000000000000000000000000000000000000000000000000000000000000000000000000000000000000000000000000000000000000000
00000000000000000000000000000000000000000000000000000000000000000000000000000000000000000000000000000000000000000000000000000000000000000000000000000000000000000000000000000000000000
00000000000000000000000000000000000000000000000000000000000000000000000000000000000000000000000000000000000000000000000000000000000000000000000000000000000000000000000000000000000000
00000000000000000000000000000000000000000000000000000000000000000000000000000000000000000000000000000000000000000000000000000000000000000000000000000000000000000000000000000000000000
00000000000000000000000000000000000000000000000000000000000000000000000000000000000000000000000000000000000000000000000000000000000000000000000000000000000000000000000000000000000000
00000000000000000000000000000000000000000000000000000000000000000000000000000000000000000000000000000000000000000000000000000000000000000000000000000000000000000000000000000000000000
00000000000000000000000000000000000000000000000000000000000000000000000000000000000000000000000000000000000000000000000000000000000000000000000000000000000000000000000000000000000000
00000000000000000000000000000000000000000000000000000000000000000000000000000000000000000000000000000000000000000000000000000000000000000000000000000000000000000000000000000000000000
00000000000000000000000000000000000000000000000000000000000000000000000000000000000000000000000000000000000000000000000000000000000000000000000000000000000000000000000000000000000000
00000000000000000000000000000000000000000000000000000000000000000000000000000000000000000000000000000000000000000000000000000000000000000000000000000000000000000000000000000000000000
00000000000000000000000000000000000000000000000000000000000000000000000000000000000000000000000000000000000000000000
00000000000000000000000000000000000000000000000000000000000000000000000000000000000000000000000000000000000000000000000000000000000000000000000000000000000000000000000000000000000000
00000000000000000000000000000000000000000000000000000000000000000000000000000000000000000000000000000000000000000000000000000000000000000000000000000000000000000000000000000000000000
00000000000000000000000000000000000000000000000000000000000000000000000000000000000000000000000000000000000000000000000000000000000000000000000000000000000000000000000000000000000000
00000000000000000000000000000000000000000000000000000000000000000000000000000000000000000000000000000000000000000000000000000000000000000000000000000000000000000000000000000000000000
00000000000000000000000000000000000000000000000000000000000000000000000000000000000000000000000000000000000000000000000000000000000000000000000000000000000000000000000000000000000000
00000000000000000000000000000000000000000000000000000000000000000000000000000000000000000000000000000000000000000000000000000000000000000000000000000000000000000000000000000000000000
00000000000000000000000000000000000000000000000000000000000000000000000000000000000000000000000000000000000000000000000000000000000000000000000000000000000000000000000000000000000000
00000000000000000000000000000000000000000000000000000000000000000000000000000000000000000000000000000000000000000000000000000000000000000000000000000000000000000000000000000000000000
00000000000000000000000000000000000000000000000000000000000000000000000000000000000000000000000000000000000000000000000000000000000000000000000000000000000000000000000000000000000000
00000000000000000000000000000000000000000000000000000000000000000000000000000000000000000000000000000000000000000000000000000000000000000000000000000000000000000000000000000000000000
00000000000000000000000000000000000000000000000000000000000000000000000000000000000000000000000000000000000000000000000000000000000000000000000000000000000000000000000000000000000000
00000000000000000000000000000000000000000000000000000000000000000000000000000000000000000000000000000000000000000000000000000000000000000000000000000000000000000000000000000000000000
00000000000000000000000000000000000000000000000000000000000000000000000000000000000000000000000000000000000000000000000000000000000000000000000000000000000000000000000000000000000000
00000000000000000000000000000000000000000000000000000000000000000000000000000000000000000000000000000000000000000000000000000000000000000000000000000000000000000000000000000000000000
00000000000000000000000000000000000000000000000000000000000000000000000000000000000000000000000000000000000000000000000000000000000000000000000000000000000000000000000000000000000000
00000000000000000000000000000000000000000000000000000000000000000000000000000000000000000000000000000000000000000000000000000000000000000000000000000000000000000000000000000000000000
00000000000000000000000000000000000000000000000000000000000000000000000000000000000000000000000000000000000000000000000000000000000000000000000000000000000000000000000000000000000000
00000000000000000000000000000000000000000000000000000000000000000000000000000000000000000000000000000000000000000000000000000000000000000000000000000000000000000000000000000000000000
00000000000000000000000000000000000000000000000000000000000000000000000000000000000000000000000000000000000000000000000000000000000000000000000000000000000000000000000000000000000000
00000000000000000000000000000000000000000000000000000000000000000000000000000000000000000000000000000000000000000000000000000000000000000000000000000000000000000000000000000000000000
00000000000000000000000000000000000000000000000000000000000000000000000000000000000000000000000000000000000000000000
>Q3S2T9
MYVGRIGATTYISRPADSRATPKVIKTQGSITTSNLTSLTTMAQSTYPNEPIVVVGSGCRFPGGANTPSKLWELLREPRDVRSKIPKERFDVDAFYHPDGKHHGRTNAPYAYMLQEDLRAFDGPFFNIQAGEAESMDPQQRLLLETVYEAVSDAGMRIQDLQGSSTA
VYVGMMTHDYETVSTRDLESIPTYSATGVAVSVASNRISYFFDWHGPSMTIDTACSSSLVAVHLAVQQLRSGQSSMAIAAGANMILGPMTFVLESKLNMLSPSGRSRMWDAGADGYARGEAVCSVVLKTLSQALRDGDSIECVIRETGVNQDGRTTGITMPNHS
AQEALIRATYSKAGLDITNPEDRCQFFEAHGTGTPAGDPQEAEAIATAFFGHKKEASDAENAETPLFVGSVKTVVGHTEGTAGLAGLMKASFAVQHGVIPPNLLFENISPRVAPFYSNLKIATETTPWPTIKPGQPRRVSVNSFGFGGTNAHAIIEEYIKSDQKVPASR
QPVEYSDSPSTLNLPLVLSAKSQRSMKTTLESMVQFLQSNPEVNLRDLSWTLLRKRSILPFRRAIVGHSHEAIRAALEAAIEDGIVVSDFSADVKGKPSVLGVFTGQGAQWPGMLKELIVGSSYVRSIAEELDHSLQTLPEKYRPSWTILEQLMLEDEASNVRHASFSQP
LCCAVQIVLVRLLKAAGIQFAAVVGHSSGEIACAFATGLISASLAIRIAHLRGVVSAEHAASASGGRGSMLAAGMSYEEAKELCELDAFESRICVAASNSPDSVTFSGDADAIEHLQGVLEDEATFARLLRVDTAYHSHHMLPCAAPYMQALEECGCAVADGDGQVE
EGSWYSSVKDSNEPMGLADVTAEYWKDNLVSPVLFSQAVQRAAIMHRPLDVGIEVGCHPALKGPCLATIKDALSDVDLAYTGCLERGKNDMNAFSQALAYLWEQFGIPSLDADRFISTIAPERSCVSLSKQLPTYSWDHSRSYWTESRATRQHLRGPKPHLLLGKL
SEYSTPLTFQWLNFVRPRDIEWLDGHALQGQVVFPAAGYIVMAMEAAMEIANSHQVQVQLLEILDMSIDKAVVFDDEDSLVELNLTAEVTSGIGKGDRMILSFIIDSCLSREGDLSTSAKGQLVVTLDEGHLQVTPDNEKQLLPPPEEEHPHMNRVNINSFYHELDL
MGYDYSKDFRRLHSMRRADARASGILEFIPLNDEVHGRPLLLHPAPLDIAFQTVIGAYSSPGDRRLRCLYVPTHIDRIALVPSLCLATAASGCDKIAFNTINTYDKGDFLSGDIVAFDAEQTSLFHVENIVFKPFSPPTASTDHPIFAKWSWGPLTPETLLDNPNHWATA
QDKEAIPIIERIVYFYIKLFLQQLTREDREQAAFHLQRQIVWCEQVVADAHEGRHQWYDAAWENDTEAQIEQLCARSSYHPHVRLVQRVGQNLLATIRSNGNPFDLMDHDGLLTEFYTNTLSFGPALHYAQDLVGQIAHRYQSMDILEIGAGTGGATKYVLATPQL
GFNSYTYTDISTGFFEKAREQFAAFEDRMEFEPLDIRRSPAEQGFTEHVYDLIIASNVLHATPDLEKTMAHARSLLKPGGQMVILEITHRNHTRLGFIFGLFADWWAGIDDGRTMEPFVSFDRWDEILKHVGFSGIDSRTKDRDADLFPTSVFSTHAVNSTIDYLHKPL
DAPVKDSYPPLVVVGGQTPKTQRILDEIKAVMPNRQIQLHQRLVDLLDAEDMQAKFTFVVLTELDEELFAGLTEDSFEAVKLLLMYAGNMLWLTENAWVKRPHQASTIGMLRSIRREHPDIGVHIMDVDSAENLDAHFLVEQVLRLEEDIDELAATTTWTQEPEV
FWCNGRAWIPRLKHDKSRNNRMNSSRRQIFETLNPSKIPVALKKAAASSSYYLESAETWPVPGAVTAGDRKTVHVRLSHPHALRVGHLGFFYLVQGHVLKGDQALPVVALAERNASIVHVRSDYVHVLEDTAVSANNGSFILAAAAAVLAETVIHSAKSLGADASV
LVLNAPGFCAQTLLRAARDSGLRVHLATTSSSTDPSPGADRCVRLHPRDTDRRLKQLLPRGTQAFFDLSTDPSSEGLTQRLPNVLIPSCVRHSTEYLLRDTASAGGKATLPAAYWERVASLANHSLSTHFKENDNASNGCQVLSCTDIVARNNKSRLNASTVISWPDD
AALPARIRPIDTETLFAAEKTYLLVGLTGDLGRSLGRWMVLHGARRIVLTSRNPQVSPNWVAHVEELGGQVTVLSMDVTSEDSVDSGLAKLQDLKLPPIGGIAFGPLVLQDVMLKNMDLQMMEMVLKPKVEGARILHEKFSDPASSNPLDFFVMFSSIVAVMGN
PGQANYSAANCYLQALAQRRCASGLAASTIDIGAVYGVGFVTRAELEEDFNAIRFMFDSVEEHELHSLFAEAVVSGRRAMHQQQQFKTVLDMADIELTTGIPPLDPTLKDRITFFDDARVGNFKIPERRGKAGDNAAGSKGSVKEQLLQATSLDQVRQIVIDGLSEK
LRVTLQIPDGESVHPTIPLIDQGVDSLGAVTVGTWFSKQLYLDLPLLRVLGGASVADLADDAAARLPPSSIPLVAASEGGAETSDNDTSGPEGTDLSASTTITEPSSADEEDEKQEDDNDNSVLALHPLSLGQEYAWRLQKAADDSTIFNNTIGMFMTGSIDAKRLSKA
LRAVLRRHEIFRTGFAAVGNNADATSLAQIVFGRTKNKVQVIQVADRAGAEEGYRQLVQTQYDITAGDTLRLVDFFWGKDEHLFVVAYHRFVGDGSTTENIFVEASQLYGGVTLDKHVPQFADLATRQREALESGQMDADLAYWESMHHQPTGVVSPVLPRML
LGEDGLNSPNHARQPNSWKQHEAIARLDPMVAFRIRERSRKHKATPMQFYLAAYHVLLARLTGSSDFSIGLADTNRTNVDELAGMGFFANLLPLRFRNFVPHITFGEHLVATKDKVREAMQHARVPYGVLLERLGFEVPGATAETAEPAPLFQAVFDYKQGQAES
GSIGSAKMTEVIATRERTPYDVVLEMSDDPTKDPLLTVKLQSSVYEVHHPRAFLESYISILSMFSMNPALKLA
00000000000000000000000000000000000000000000000000000000000000000000000000000000000000000000000000000000000000000000000000000000000000000000000000000000000000000000000000000000000000
00000000000000000000000000000000000000000000000000000000000000000000000000000000000000000000000000000000000000000000000000000000000000000000000000000000000000000000000000000000000000
00000000000000000000000000000000000000000000000000000000000000000000000000000000000000000000000000000000000000000000000000000000000000000000000000000000000000000000000000000000000000
00000000000000000000000000000000000000000000000000000000000000000000000000000000000000000000000000000000000000000000000000000000000000000000000000000000000000000000000000000000000000
00000000000000000000000000000000000000000000000000000000000000000000000000000000000000000000000000000000000000000000000000000000000000000000000000000000000000000000000000000000000000
00000000000000000000000000000000000000000000000000000000000000000000000000000000000000000000000000000000000000000000000000000000000000000000000000000000000000000000000000000000000000
00000000000000000000000000000000000000000000000000000000000000000000000000000000000000000000000000000000000000000000000000000000000000000000000000000000000000000000000000000000000000
00000000000000000000000000000000000000000000000000000000000000000000000000000000000000000000000000000000000000000000000000000000000000000000000000000000000000000000000000000000000000
00000000000000000000000000000000000000000000000000000000000000000000000000000000000000000000000000000000000000000000000000000000000000000000000000000000000000000000000000000000000000
00000000000000000000000000000000000000000000000000000000000000000000000000000000000000000000000000000000000000000000000000000000000000000000000000000000000000000000000000000000000000
00000000000000000000000000000000000000000000000000000000000000000000000000000000000000000000000000000000000000000000000000000000000000000000000000000000000000000000000000000000000000
00000000000000000000000000000000000000000000000000000000000000000000000000000000000000000000000000000000000000000000000000000000000000000000000000000000000000000000000000000000000000
00000000000000000000000000000000000000000000000000000000000000000000000000000000000000000000000000000000000000000000000000000000000000000000000000000000000000000000000000000000000000
00000000000000000000000000000000000000000000000000000000000000000000000000000000000000000000000000000000000000000000000000000000000000000000000000000000000000000000000000000000000000
00000000000000000000000000000000000000000000000000000000000000000000000000000000000000000000000000000000000000000000000000000000000000000000000000000000000000000000000000000000000000
00000000000000000000000000000000000000000000000000000000000000000000000000000000000000000000000000000000000000000000000000000000000000000000000000000000000000000000000000000000000000
000000000000000000000000000000000000000000000000000000000000000000000000000000000000000
00000000000000000000000000000000000000000000000000000000000000000000000000000000000000000000000000000000000000000000000000000000000000000000000000000000000000000000000000000000000000
00000000000000000000000000000000000000000000000000000000000000000000000000000000000000000000000000000000000000000000000000000000000000000000000000000000000000000000000000000000000000
00000000000000000000000000000000000000000000000000000000000000000000000000000000000000000000000000000000000000000000000000000000000000000000000000000000000000000000000000000000000000
00000000000000000000000000000000000000000000000000000000000000000000000000000000000000000000000000000000000000000000000000000000000000000000000000000000000000000000000000000000000000
00000000000000000000000000000000000000000000000000000000000000000000000000000000000000000000000000000000000000000000000000000000000000000000000000000000000000000000000000000000000000
00000000000000000000000000000000000000000000000000000000000000000000000000000000000000000000000000000000000000000000000000000000000000000000000000000000000000000000000000000000000000
00000000000000000000000000000000000000000000000000000000000000000000000000000000000000000000000000000000000000000000000000000000000000000000000000000000000000000000000000000000000000
00000000000000000000000000000000000000000000000000000000000000000000000000000000000000000000000000000000000000000000000000000000000000000000000000000000000000000000000000000000000000
00000000000000000000000000000000000000000000000000000000000000000000000000000000000000000000000000000000000000000000000000000000000000000000000000000000000000000000000000000000000000
00000000000000000000000000000000000000000000000000000000000000000000000000000000000000000000000000000000000000000000000000000000000000000000000000000000000000000000000000000000000000
00000000000000000000000000000000000000000000000000000000000000000000000000000000000000000000000000000000000000000000000000000000000000000000000000000000000000000000000000000000000000
00000000000000000000000000000000000000000000000000000000000000000000000000000000000000000000000000000000000000000000000000000000000000000000000000000000000000000000000000000000000000
00000000000000000000000000000000000000000000000000000000000000000000000000000000000000000000000000000000000000000000000000000000000000000000000000000000000000000000000000000000000000

000
000
000
000
000
000
0000000000000000000000000000000000000000000000000000000000000000000000000000000000000000000000000000
000
000
000
000
000
000
000
000
000
000
000
000
000
000
000
000
000
000
000
000
000
0000000000000000000000000000000000000000000000000000000000000000000000000000000000000000000000000000
>Q939N5
MFFKRQKGQYHEVERVTRFKLIKSGKHWLRAATSQFGLLRLMKGSDVSSTEVKVVEEQSVEKSGLNYLKGIIATGAVLGGAVVTSSSVYAEEEQAHEKVIDTRDVLATRGEAVLSEEAATTLSSTEANPVESLSDTLSASESTSASSSVSTSISVSESFSVSGSLSYSTSLSQ
SVSASASASESLSVSSSASDSVSASTSTSASASQSVSASQKSTISTSESTRSESSQQSTEASSQTGRRRTRRAVTESAPNVEYHDVKGDMIQSVTTSFDDTSRLLTWTINLTPRQVKSNLGALVSISGNQETRTVTINGKNAANGGVYNSGGAWNLYTGESVNNNVLRI
TTQVNDTGGEVKLGLRLVTSDKKITKTNLPLEFSQVAATTNGSWDKAGYNTTIVEKDTERPVVNVPSEITVYRGESFEYFATVTDNSNAFDLAKTVVRWLYNNQPGRGTEWLQYSVTQVGNQLKVRIFGNVPIDTTIGDYTRYVVATDAAGNVNATQTEMGNAA
VDKTSVNGQFKLIIRFRIKTPENTVFVNNPNQLTEVEKNLVREAVKKSNPDLRAQDVLNSNYVTGITVSNNGTTTITYRDGRKDIIDGSKFIDTRAGSISKSQSTSNSISVSLSKSESASASLVTSKLNSISSSASVSASTSISTSGSVSASESASTSSSVSASESASTSASVSASE
SASTSASVSASTSASTSASVSASTSASTSASTSASKSASTSASVSASTSASTSASVSASESASTSASVSASTSASTSASVSASTSASTSASVSASESASTSASVSASTSASTSASVSASESASTSASVSASTSASTSASVSASASASTSASVSASTSASTSASVSASASASTSASVS
ASTSASTSASVSASESASTSASVSASESASTSASVSASESASTSASVSASESASTSASVSASTSASTSASVSASESASTSASVSASESASTSASVSASESASTSASVSASESASTSASVSASTSASTSASVSASTSASTSASVSASTSASTSASVSASTSASTSASVSASESASTSAS
VSASESASTSASVSASTSASTSASVSASESASTSASVSASESASTSASESASESASTSASVSASESASTSASVSASESSSTSASVSASESSSTSASVSASESASTSASVSASESASTSASESASESASTSASVSASESASTSASVSASESASTSASVSASESVSTSASVSASESASTS
ASVSASESASTSASESASESASTSASVSASESASTSASVSASESASTSASVSASTSASTSASVSASESASTSASVSASESASTSASVSASESASTSASVSASESVSTSASVSASESASTSASVSASESASTSASESASESASTSASVSASESASTSASVSASESASTSASVSASTSAS
TSASVSASESASTSASVSASESASTSASVSASESASTSASVSASTSASTSASVSASESASTSTSVSTSTSASTSASVSASESASTSASVSASESASTSASVSASTSASTSASVSASESASTSASVSASESASTSASVSASESASTSASVSASESASTSASVSASTSASTSASVSASES
ASTSASVSASESASTSASVSASESASTSASVSASESASTSASVSASESASTSASVSASESASTSASVSASESASTSASVSASESASTSASVSASESASTSASVSASESASTSASVSASESASTSASVSASESASTSASVSASESASTSASVSASESASTSASVSASESASTSASVSAS
ESASTSASVSASESASTSASVSASESASTSASVSASESASTSASVSASESASTSASVSASESASTSASVSASESASTSASVSASESASTSASVSASESASTSASVSASTSTSTSASVSASESASTSASVSASESASTSASVSASESASTSASVSASESASTSASVSASESASTSASVS
ASESASTSASVSASESASTSASVSASESASTSASVSASESASTSASVSASESASTSASVSASTSASTSASVSASESASTSASVSASESASTSASVSASESASTSASVSASESASTSASVSASESASTSASVSASESASTSASVSASESASTSASVSASESASTSASVSASKSASTSES
ASTSASVSASESASTSASVSASESASTSASVSASESVSTSASVSASDSASISASVLASESASTSASVSASESASTSASVSASESASTSASVSASESASTSSSVSASESASTSASVSASESASTSASVSASTSASTSASVSASESASTSASVSASESASTSASVSASESASTSASVSAS
ESASTSASVSASESASTSASVSASESASTSASVSASESASTSASVSASTSASTSASVSASESASTSASVSSSESASTSASVSASESASTSASVSASESASTSASVSASESASTSASVSASESASTSASVSASTSASTSASVSASESASTSASVSASESASTSASVSASESASTSASVS
ASESASTSASVSASTSASTSASVSASESASTSASVSASESASTSASVSASTSASTSASVSASESASTSASVSASESASTSASVSASESASTSASVSASESASTSASVSASESASTSASVSASESASTSASVSASESASTSASVSASMSASTSASVSVSESTSTSASVSANESASTSA
SVSASESASTSASVSASESASTSASVSASESASTSASVSASESASTSASVSASESASTSASVSASESASTSASVSASESASTSASVSASESASTSASVSASTSASTSASVSANESASTSASVSASESASTSASVSASESASTSASVSASESASTSASVSASESAS
TSASVSASTSASTSASVSANESASTSASVSASESASTSASVSASESASTSASVSASESASTSASVSASESASTSASVSASTSASTSASVSASESASTSASASASESASTSASVSASESASTSASVSASESASTSASVSASESASTNASVSVSESMSVSESLSLSISTSVLHSQLNDI
YESELYSLSLSESLSASQSLSQSLSESQSSSASQSMHDRISKGQLPRTGESENKASILALGLGALGLAFKKRKKNESED
000
000
000
000
000
000
000
000
000
000
000
000
000
000
000
000
000
000
000
000
000
0000000000000000000000000000000000000000000000000000000000000000000000000000000000000000000000000000
000
000
000
000
000
000
000
000
000
000
000
000
000
000
000
000
000
000
000
000
000
0000000000000000000000000000000000000000000000000000000000000000000000000000000000000000000000000000
000
000
000
000
000
000
000
000
000
000
000
000
000
000
000
000
000
000
000
000
000
0000000000000000000000000000000000000000000000000000000000000000000000000000000000000000000000000000
>A0A221ZS22
MGRITWCVLVFCATTWASPHSDVSEFLRSAVETVPQHDPIAAPIRSSVESIPLRSLREEGEGRLPLRDAVEKRPISLIDENSFSLKTVENEEPDNNDDTFVELKKFYGAASYLNTKNDERLQHGNANLQEMQQTSGATDRRMVVCYMQSWAAYRAPPLAFTAGLVP
RSCTHLHYAFAAIHPHTYAVLPANEDYDIIKGGYRIATGLKRRLQGLKVMISVGGEGTDRLFSELVQEPNRRSLFIDSAVDFLREHDFDGLDLHWVYPGERDEREKELLTSLLYELREKFSSYGLLLSTVLPPFRYQIEDGYDLSAVSGATDYTILQAWDMTHGKKDEPPA
RALHHSTLHRDPGAASRDQRYDNIEFMVKYIIRHGMAADKLVLGVPLFGRSYTLAASTLPSPGAPVSGWGEEGQYTQTKGMLSYFEICMKERMGDGVVAFDEAGNSFALFDNQWVSFDSQSTVLEKMRFVISTGLAGAAAWSVDMDDFRGLCGTPFPILSTIST
SLNGESIQSDQSTLKIGFCESDTPYLASDEESCAHFHFCAGGINYRMLCEEDRLYDPSTGFCGHLDVAKCIPGQSLRISVQEAERYLSQAYETDFEWNGQSIKDIQAAQLMSNDVDTKKNKESDKKVVCYMTSWAFYRRGEGKFVPEHIDSRLCTHVVFAYASLSPDE
LIAKEFDPWADLTNNLYERVTSLKDVKVLLGLGGWTDSAGDKYSRLVSSDSARSKFIQKLIPFLRMHNFNGLHLDWNYPVCWQSNCKKGAASDKANYAMFVQELSKALHAAGMELGVALSGYKEVIDSAYDLPAISRAADFMSTMTYDYHGGWESSTGHHTPLV
PAPGDALAYYSIEYAIKALLKGGADPKKLLLGLSFYGQSYRLAKVEGSKNPGAPAGGPGEPGEFTKQPGMLAYYEICYRVKNLRWATGRQANAGPYAYSDSQWVGYDDPKSITEKVEWAMRQGLGGVTAWAIDLDDFNNRCCAEPSPLLRAAGRALGRSVPAPG
AECERPPQPVTPAPPTTTTAQADGSLGGGDHSGHDHGQHTSTTWGWVPSSTTPTSAQTWWPPATTPSTTTTTKATTTTTTTRRPTAAPVRPPPDAGGLEGTSCTAGEYRAAPGDCGGYLQCEGGQWRKQRCAPGLHWAASAHRCDWPSFAKCTEESSSNEA
STATSTLAPMTSRPPPRPSTTTRRTTTSTTTTTTTTTTTTPRPTTTRKPTTTSRPSAPSSSEEVSDGKPCNNQGYKPASDCNSYLHCDGNMWIKQQCAPGLHWSPTENQCDWPKYAKCKVSASKPTTSAPRPTYPSPKPTSLIPSGDNPFEDGQCRSNDVHAPAKSC
DSYLLCVGGRWRKQLCPPGLHWDKRTSRCDWVEFAMCQEDKPSATKSPIAQSTTTTTTTTTTRRPTTTTTTTTRRPVSAAIEESHRPSTGCITGTYYPHPKCEKFFVCVNNLMVAQSCAPGLVWNAERSQCDFPSSVSCSDRRQVMSAAMTDASGESTSMQLNDE
VPLYCDNGQYAMLPTDCTRYRHCIFGKFEEFSCSAGLHWNQEKQICDWPSNAKCKAKGTTKPKPTTAPKPESSTRPSKPTKPVYIDNEDHLPEPSPTKPIASAPPTGTLPDLPSKPSLLNSRYKLVCYYTNWSWYRPGIGKYSPEDIDPSLCTHIVYGFAVLGNDGLMT
AHDTWSDYDNRFYERVVEYKRYGIKVSLALGGWNDSAGDKYSKLVNDPAARAKFVQHAVAFLEKYGFDGLDLDWEYPKCWQVDCSKGPDSDKQGFADLVHELSAVLKPKGLLLSAAVSPNKMVIDAGYDVPVLARLLDWIAVMTYDYHGQWDKKTGHVAPL
YYHPDDDTTYFNANYTIHYWMEKGTPASKIVMGMPMYGQSFTIGDKWDSPREQWRKDANGLNIPVSDGGEPGEYTRAKGFLAYYEICDRIRNSGWTVVKDPYQRMGPYAYKGNQWVSFDDVEIIKKKVNFIKSLNLGGGMIWALDLDDYRNRCGQGKHPLL
NAIKTELLNPKIEMEKEMQQKPHKPIYEPPNNIDDDLDDIQVPIYHRPTSRPTSQTSIMTTTTKKPVMVAAPVKEERFKVVCYYTNWAWYRPDNGKYTPGDINPELCTHIIYAFAVLDKEELVIKSHDIWLDVENKFYEKVTALKSHGVKVLLGLGGWDDSAGDKYSR
LVNNVSARRKFVVHAVDFLEQYGFDGLDLDWEYPKCWQVECEKGPDSDKQGFADLVKELRKAFNRRGMLLSAAVSASKRVIDYAYNVPALSMNLDWISLMTYDYHGQWDKKTGHVAPMYVHDKDTDNTFNVNFTVNYWINKGADRKKLVVGVPFYGQSFS
VVEGAGTGLGAPTYAGGEAGDETRARGFLSFYEICERVKVKGWKVHRDPGGRIGPYATHDDQWVSFDDDFMARHKAEYVRAMELGGSMAWSLDLDDFTGKYCGCGKAPLLTTINHVLRGKEAPPPCILEELHESSPMAVPGVPSQPEVPEPEEKPMIIDTDHH
QHVQHVEPSGTSLDGKACSGAIFRADESNCANYYLCVGKRYMQLTCPAPLVWNQNHCDWPEKSQCKGKTHLRIRDEMEPVVQDKPILACYFTNWAYYRHGNGSFGPEQVDPSMCTHIVYAWAHLDPATYKLVPGNPELDIDNDFYGKMTELRMKGVKVIIGA
GGLEDSEDEKWVEMSSSPESIDVFVESVLKFLNRWNFDGLQIAWQYPVCKQIPCTDFDITEKDNFSLLIAKLSKTLHQHNFELSALVSSSPEVAALAYDPEVLTATVDWIALAANDYYASTSGRTSYLVPLESIEMAGINSFNSSLAYWSSVVPAEKLVIGVPAYARSYTL
RSAAHKHIQSPVTGPGIPGHFTRVPGFLAFYELCPGSKCSSWEETRTEDGTFAVHRSQWASYLRPEEIHRVGASAATAGLRGAALWALDLDDFRALCSCEPNPLLAALRQGLLDPNIPPTLCNSE
000
000
000
000
000
000
000
000
000
000
000
000
000
000

000000000000000000000000000000000000000000000000000000000000000000000000000000000000000000000000000000000000000000000000000000000000000000000000000000
000000000000000000000000000000000000000000000000000000000000000000000000000000000000000000000000000000000000000000000000000000000000000000000000000000
000000000000000000000000000000000000000000000000000000000000000000000000000000000000000000000000000000000000000000000000000000000000000000000000000000
000000000000000000000000000000000000000000000000000000000000000000000000000000000000000000000000000000000000000000000000000000000000000000000000000000
000000000000000000000000000000000000000000000000000000000000000000000000000000000000000000000000
000000000000000000000000000000000000000000000000000000000000000000000000000000000000000000000000000000000000000000000000000000000000000000000000000000
000000000000000000000000000000000000000000000000000000000000000000000000000000000000000000000000000000000000000000000000000000000000000000000000000000
000000000000000000000000000000000000000000000000000000000000000000000000000000000000000000000000000000000000000000000000000000000000000000000000000000
000000000000000000000000000000000000000000000000000000000000000000000000000000000000000000000000000000000000000000000000000000000000000000000000000000
000000000000000000000000000000000000000000000000000000000000000000000000000000000000000000000000000000000000000000000000000000000000000000000000000000
000000000000000000000000000000000000000000000000000000000000000000000000000000000000000000000000000000000000000000000000000000000000000000000000000000
000000000000000000000000000000000000000000000000000000000000000000000000000000000000000000000000000000000000000000000000000000000000000000000000000000
000000000000000000000000000000000000000000000000000000000000000000000000000000000000000000000000000000000000000000000000000000000000000000000000000000
000000000000000000000000000000000000000000000000000000000000000000000000000000000000000000000000000000000000000000000000000000000000000000000000000000
000000000000000000000000000000000000000000000000000000000000000000000000000000000000000000000000000000000000000000000000000000000000000000000000000000
000000000000000000000000000000000000000000000000000000000000000000000000000000000000000000000000000000000000000000000000000000000000000000000000000000
000000000000000000000000000000000000000000000000000000000000000000000000000000000000000000000000000000000000000000000000000000000000000000000000000000
000000000000000000000000000000000000000000000000000000000000000000000000000000000000000000000000000000000000000000000000000000000000000000000000000000
000000000000000000000000000000000000000000000000000000000000000000000000000000000000000000000000000000000000000000000000000000000000000000000000000000
000000000000000000000000000000000000000000000000000000000000000000000000000000000000000000000000000000000000000000000000000000000000000000000000000000
000000000000000000000000000000000000000000000000000000000000000000000000000000000000000000000000000000000000000000000000000000000000000000000000000000
000000000000000000000000000000000000000000000000000000000000000000000000000000000000000000000000000000000000000000000000000000000000000000000000000000
000000000000000000000000000000000000000000000000000000000000000000000000000000000000000000000000
000000000000000000000000000000000000000000000000000000000000000000000000000000000000000000000000000000000000000000000000000000000000000000000000000000
000000000000000000000000000000000000000000000000000000000000000000000000000000000000000000000000000000000000000000000000000000000000000000000000000000
000000000000000000000000000000000000000000000000000000000000000000000000000000000000000000000000000000000000000000000000000000000000000000000000000000
000000000000000000000000000000000000000000000000000000000000000000000000000000000000000000000000000000000000000000000000000000000000000000000000000000
000000000000000000000000000000000000000000000000000000000000000000000000000000000000000000000000000000000000000000000000000000000000000000000000000000
000000000000000000000000000000000000000000000000000000000000000000000000000000000000000000000000000000000000000000000000000000000000000000000000000000
000000000000000000000000000000000000000000000000000000000000000000000000000000000000000000000000000000000000000000000000000000000000000000000000000000
000000000000000000000000000000000000000000000000000000000000000000000000000000000000000000000000000000000000000000000000000000000000000000000000000000
000000000000000000000000000000000000000000000000000000000000000000000000000000000000000000000000000000000000000000000000000000000000000000000000000000
000000000000000000000000000000000000000000000000000000000000000000000000000000000000000000000000000000000000000000000000000000000000000000000000000000
000000000000000000000000000000000000000000000000000000000000000000000000000000000000000000000000000000000000000000000000000000000000000000000000000000
000000000000000000000000000000000000000000000000000000000000000000000000000000000000000000000000000000000000000000000000000000000000000000000000000000
000000000000000000000000000000000000000000000000000000000000000000000000000000000000000000000000000000000000000000000000000000000000000000000000000000
000000000000000000000000000000000000000000000000000000000000000000000000000000000000000000000000000000000000000000000000000000000000000000000000000000
000000000000000000000000000000000000000000000000000000000000000000000000000000000000000000000000000000000000000000000000000000000000000000000000000000
000000000000000000000000000000000000000000000000000000000000000000000000000000000000000000000000000000000000000000000000000000000000000000000000000000
000000000000000000000000000000000000000000000000000000000000000000000000000000000000000000000000000000000000000000000000000000000000000000000000000000
000000000000000000000000000000000000000000000000000000000000000000000000000000000000000000000000
>Q15782
MGATTMDQKSLWAGVVVLLLLQGGSAYKLVCYFTNWSQDRQEPGKFTPENIDPFLCSHLIYSFASIENNKVIIKDKSEVMLYQTINSLKTKNPKLKILLSIGGYLFGSKGFHPMVDSSTSRLEFINSIILFLRNHNFDGLDVSWIYPDQKENTHFTVLIHELAEAFQKDFT
KSTKERLLLTAGVSAGRQMIDNSYQVEKLAKDLDFINLLSFDFHGSWEKPLITGHNSPLSKGWQDRGPSSYYNVEYAVGYWIHKGMPSEKVVMGIPTYGHSFTLASAETTVGAPASGPGAAGPITESSGFLAYYEICQFLKGAKITRLQDQQVPYAVKGNQWVGYD
DVKSMETKVQFLKNLNLGGAMIWSIDMDDFTGKSCNQGPYPLVQAVKRSLGSL
000000000000000000000000000000000000000000000000000000000000000000000000000000000000000000000000000000000000000000000000000000000000000000000000000000
000000000000000000000000000000000000000000000000000000000000000000000000000000000000000000000000000000000000000000000000000000000000000000000000000000
0000000000000000000000000000000000000000
000000000000000000000000000000000000000000000000000000000000000000000000000000000000000000000000000000000000000000000000000000000000000000000000000000
000000000000000000000000000000000000000000000000000000000000000000000000000000000000000000000000000000000000000000000000000000000000000000000000000000
0000000000000000000000000000000000000000
000000000000000000000000000000000000000000000000000000000000000000000000000000000000000000000000000000000000000000000000000000000000000000000000000000
000000000000000000000000000000000000000000000000000000000000000000000000000000000000000000000000000000000000000000000000000000000000000000000000000000
0000000000000000000000000000000000000000
>Q7YS85
MGLRVAQTGFVVLVLLQSCAAYKLICYYTSWSQYREGDGSCFPDAIDPFLCTHVIYSFANISNNEIDTWEWNDVTLYDTLNTLKNRNPKLKTLLSVGGWNFGSQRFSKIASKTQSRRTFIKSVPPFLRTHGFDGLDLAWLYPGWRDKRHLTTLVKEMKAEFVREAQA
GTEQLLLSAAVPAGKIAIDRGYDIAQISRHLDFISLLTYDFHGAWRQTVGHHSPLFRGQEDASSDRFSNADYAVSYMLRLGAPANKLVMGIPTFGKSYTLASSKTDVGAPISGPGIPGQFTKEKGILAYYEICDFLHGATTHRFRDQQVPYATKGNQWVAYDDQESVK
NKARYLKNRQLAGAMVWALDLDDFRGTFCGQNLAFPLTNAIKDVLAGV
000000000000000000000000000000000000000000000000000000000000000000000000000000000000000000000000000000000000000000000000000000000000000000000000000000
000000000000000000000000000000000000000000000000000000000000000000000000000000000000000000000000000000000000000000000000000000000000000000000000000000
0000000000000000000000000000000000
000000000000000000000000000000000000000000000000000000000000000000000000000000000000000000000000000000000000000000000000000000000000000000000000000000
000000000000000000000000000000000000000000000000000000000000000000000000000000000000000000000000000000000000000000000000000000000000000000000000000000
0000000000000000000000000000000000
000000000000000000000000000000000000000000000000000000000000000000000000000000000000000000000000000000000000000000000000000000000000000000000000000000
000000000000000000000000000000000000000000000000000000000000000000000000000000000000000000000000000000000000000000000000000000000000000000000000000000
0000000000000000000000000000000000
>Q6TMG6
YKLICYYTSWSQYREGDGSCFPDAIDPFLCTHVIYSFANISNNEIDTWEWNDVTLYDTLNTLKNRNPKLKTLLSVGGWNFGPERFSAIASKTQSRRTFIKSVPPFLRTHGFDGLDLAWLYPGRRDKRHLTTLVKEMKAEFIREAQAGTEQLLLSAAVSAGKIAIDRGYDI
AQISRHLDFISLLTYDFHGAWRQTVGHHSPLFAGNEDASSRFSNADYAVSYMLRLGAPANKLVMGIPTFGRSFTLASSKTDVGAPVSGPGVPGRFTKEKGILAYYEICDFLHGATTHRFRDQQVPYATKGNQWVAYDDQESVKNKARYLKNRQLAGAMVWALDL
DDFRGTFCGQNLTFPLTSAVKDVLAEV
000000000000000000000000000000000000000000000000000000000000000000000000000000000000000000000000000000000000000000000000000000000000000000000000000000
000000000000000000000000000000000000000000000000000000000000000000000000000000000000000000000000000000000000000000000000000000000000000000000000000000
000000000
000000000000000000000000000000000000000000000000000000000000000000000000000000000000000000000000000000000000000000000000000000000000000000000000000000
000000000000000000000000000000000000000000000000000000000000000000000000000000000000000000000000000000000000000000000000000000000000000000000000000000
000000000
000000000000000000000000000000000000000000000000000000000000000000000000000000000000000000000000000000000000000000000000000000000000000000000000000000
000000000000000000000000000000000000000000000000000000000000000000000000000000000000000000000000000000000000000000000000000000000000000000000000000000
000000000
>Q8B3M2
MAFSCGTLSYSAVAQAPSVAYAPRTWEVDEARRRRVIKRLALEQERIRNVLDVAVYDQATWEQEDARDNEFLTEQLNNLYTIYSIAERCTRRPIKEXSPISVSNRFAPLESLKVEVGQEAXECXFKKPKYTRXCKKVKRVATRFVREKVVRPMCSRSPMLLFKLKKIIYD
LHLYRLRKQIRMLRRQKQRDYELECVTNLLQLSNPVQAKPEMDNPNPGPDGEGEVELEKDSNVVLTTQRDPSTSIPAPVSVKWSRWTSNDVVDDYATITSRWYQIAEFVWSKDDPFDKELARLILPRALLSSIEANSDAICDVPNTIPFKVHAYWRGDMEVRVQIN
SNKFQVGQLQATWYYSDHENLNISSKRSVYGFSQMDHALISASASNEAKLVIPYKHVYPFLPTRIVPDWTTGILDMGALNIRVIAPLRMSATGPTTCNVVVFIKLNNSEFTGTSSGKFYASQIRAKPEMDRILNLAEGLLNNTIGGNNMDNPSYQQSPRHFVPTGMH
SLALGTNLVEPLHALRLDAAGTTQHPVGCAPDEDMTVSSIASRYGLIRRVQWKKDHAKGSLLLQLDADPFVEQRIEGTNPISLYWFAPVGVVSSMFMQWRGSLEYRFDIIASQFHTGRLIVGYVPGLTASLQLQMDYMKLKSSSYVVFDLQESNSFTFEVPYVSYRP
WWVRKYGGNYLPSSTDAPSTLFMYVQVPLIPMEAVSDTIDINVYVRGGSSFEVCVPVQPSLGLNWNTDFILRNDEEYRAKTGYAPYYAGVWHSFNNSNSLVFRWGSXSDQIAQWPTISVPRGELAFLRIKDGKQAAVGTQPWRTMVVWPSGHGYNIGIPTYNA
ERARQLAQHLYGGGSLTDEKAKQLFVPANQQGPGKVSNGNPVWEVMRAPLATQRAHIQDFEFIEAIPEGEESRNTTVLDTTTTLQSSGFGRAFFGEAFNDLKTLMRRYQLYGQLLLSVTTDKDIDHCMFTFPCLPQGLALDIGSAGSPHEIFNRCRDGIIPLIASGYR
FYRGDLRYKIVFPSNVNSNIWVQHRPDRRLEGWSAAKIVNCDAVSTGQGVYNHGYASHIQITRVNNVIELEVPFYNATCYNYLQAFNASSAASSYAVSLGEISVGFQATSDDIASIVNKPVTIYYSIGDGMQFSQWVGYQPMMILDQLPAPVVRAVPEGPIAKIKNF
FHQTADEVREAQAAKMREDMGMVVQDVIGELSQAIPDLQQPEVQANVFSLVSQLVHAIIGTSLKTVAWAIVSIFVTLGLIGREMMHSVITVVKRLLEKYHLATQPQESASSSTVISAVPEAPNAEAEEASAWVSIIYNGVCNMLNVAAQKPKQFKDWVKLATVDF
SNNCRGSNQVFVFFKNTFEVLKKMWGYVFCQSNPAARLLKAVNDEPEILKAWVKECLYLDDPKFRMRRAHDQEYIERVFAAHSYGQILLHDLTAEMNQSRNLSVFTRVYDQISKLKTDLMEMGSNPYIRRECFTICMCGASGIGKSYLTDSLCSELLRASRTPVTTGI
KCVVNPLSDYWDQCDFQPVLCVDDMWSVETSTTLDKQLNMLFQVHSPIVLSPPKADLEGKKMRYNPEIFIYNTNKPFPRFDRIAMEAIYRRRNVLIECKASEEKKRGCKHCENDIPIAECSPKMLKDFHHIKFRYAHDVCNSETTWSEWMTYNEFLEWITPVYMA
NRRKANESFKMRVDEMQMLRMDEPLEGDNILNKYVEVNQRLVEEMKAFKERTLWSDLHRVGAEISASVKKALPTISITEKLPHWTVQCGIAKPEMDHAYEVMSSYAAGMNAEIEAHEQVRRSSVECQFAEPQAXRNPDDEGPTIDEELMGDTEFTSQALERLV
DEGYITGKQKKYIAMWCSKRREHTADFDLVWTDNLRVLSAYVHERSSSTRLSTDDVKLYKTISMLHQKYDTTECAKCQHWYAPLTDIYVDDKKLFWCQKEKKTLIDVRKLSKEDVTVQSKLXNLSVPCGEVCMLHSKYFNYLFHKAWLFENPTWRLIYNGTKKGMP
EYFMNCVDEISLDSKFGKVKVWLQAIIDKYLTRPVKMIRDFLFKWWPQVAYVLSLLGIIGITAYEMRNPKPTSEELADHYVNRHCSSDFWSPGLASPQGLKYSEAVTVKAPRIHRLPVTTKPQGSTQQVDAAVNKILQNMVYIGVVFPKVPGSKWRDINFRCLMLH
NRQCLMLRHYIESTAAFPEGTKYYFKYIHNQETRMSGDISGIEIDLLNLPRLYYGGLAGEESFDSNIVLVTMPNRIPECKSIIKFIASHNEHIRAQNDGVLVTGDHTQLLAFENNNKTPISINADGLYEVILQGVYTYPYHGDGVCGSILLSRNLQRPIIGIHVAGTEGLHGF
GVAEPLVHEMFTGKAIESEREPYDRVYELPLRELDESDIGLDTDLYPIGRVDAKLAHAQSPSTGIKKTLIHGTFDVRTEPNPMSSRDPRIAPHDPLKLGCEKHGMPCSPFNRKHLELATNHLKEKLVSVVKPINGCKIRSLQDAXCGVPGLDGFDSISWNTSAGFPLSSLK
PPGTSGKRWLFDIELQDSGCYLLRGMRPELEIQLSTTQLMRKKGIKPHTIFTDCLKDTCLPVEKCRIPGKTRIFSISPVQFTIPFRQYYLDFMASYRAARLNAEHGIGIDVNSLEWTNLATRLSKXGTHIVTGDYKNFGPGLDSDVAASAFEIIIDWVLHYTEEDNKDEMK
RVMWTMAQEILAPSHLYRDLVYRVPCGIPSGSPITDILNTISNCLLIRLAWLGITDLPLSEFSQNVVLVCYGDDLIMNVSDNMIDKFNAVTIGKFFSQYKMEFTDQDKSGNTVKWRTLQTATFLKHGFLKHPTRPVFLANLDKVSVEGTTNWTHARGLGRRTATIEN
AKQALELAFGWGPEYFNYVRNTIKMAFDKLGIYEDLITWEEMDVRCYASA
000000000000000000000000000000000000000000000000000000000000000000000000000000000000000000000000000000000000000000000000000000000000000000000000000000
000000000000000000000000000000000000000000000000000000000000000000000000000000000000000000000000000000000000000000000000000000000000000000000000000000
000000000000000000000000000000000000000000000000000000000000000000000000000000000000000000000000000000000000000000000000000000000000000000000000000000
000000000000000000000000000000000000000000000000000000000000000000000000000000000000000000000000000000000000000000000000000000000000000000000000000000
000000000000000000000000000000000000000000000000000000000000000000000000000000000000000000000000000000000000000000000000000000000000000000000000000000
000000000000000000000000000000000000000000000000000000000000000000000000000000000000000000000000000000000000000000000000000000000000000000000000000000
000000000000000000000000000000000000000000000000000000000000000000000000000000000000000000000000000000000000000000000000000000000000000000000000000000
000000000000000000000000000000000000000000000000000000000000000000000000000000000000000000000000000000000000000000000000000000000000000000000000000000
000000000000000000000000000000000000000000000000000000000000000000000000000000000000000000000000000000000000000000000000000000000000000000000000000000
000000000000000000000000000000000000000000000000000000000000000000000000000000000000000000000000000000000000000000000000000000000000000000000000000000
000000000000000000000000000000000000000000000000000000000000000000000000000000000000000000000000000000000000000000000000000000000000000000000000000000
000000000000000000000000000000000000000000000000000000000000000000000000000000000000000000000000000000000000000000000000000000000000000000000000000000
000000000000000000000000000000000000000000000000000000000000000000000000000000000000000000000000000000000000000000000000000000000000000000000000000000
000000000000000000000000000000000000000000000000000000000000000000000000000000000000000000000000000000000000000000000000000000000000000000000000000000
000000000000000000000000000000000000000000000000000000000000000000000000000000000000000000000000000000000000000000000000000000000000000000000000000000
000000000000000000000000000000000000000000000000000000000000000000000000000000000000000000000000000000000000000000000000000000000000000000000000000000
000000000000000000000000000000000000000000000000000000000000000000000000000000000000000000000000000000000000000000000000000000000000000000000000000000
000000000000000000000000000000000000000000000000000000000000000
000000000000000000000000000000000000000000000000000000000000000000000000000000000000000000000000000000000000000000000000000000000000000000000000000000
000000000000000000000000000000000000000000000000000000000000000000000000000000000000000000000000000000000000000000000000000000000000000000000000000000
000000000000000000000000000000000000000000000000000000000000000000000000000000000000000000000000000000000000000000000000000000000000000000000000000000
000000000000000000000000000000000000000000000000000000000000000000000000000000000000000000000000000000000000000000000000000000000000000000000000000000
000000000000000000000000000000000000000000000000000000000000000000000000000000000000000000000000000000000000000000000000000000000000000000000000000000
000000000000000000000000000000000000000000000000000000000000000000000000000000000000000000000000000000000000000000000000000000000000000000000000000000
000000000000000000000000000000000000000000000000000000000000000000000000000000000000000000000000000000000000000000000000000000000000000000000000000000
000000000000000000000000000000000000000000000000000000000000000000000000000000000000000000000000000000000000000000000000000000000000000000000000000000
000000000000000000000000000000000000000000000000000000000000000000000000000000000000000000000000000000000000000000000000000000000000000000000000000000
000000000000000000000000000000000000000000000000000000000000000000000000000000000000000000000000000000000000000000000000000000000000000000000000000000
000000000000000000000000000000000000000000000000000000000000000000000000000000000000000000000000000000000000000000000000000000000000000000000000000000
000000000000000000000000000000000000000000000000000000000000000000000000000000000000000000000000000000000000000000000000000000000000000000000000000000
000000000000000000000000000000000000000000000000000000000000000000000000000000000000000000000000000000000000000000000000000000000000000000000000000000
000000000000000000000000000000000000000000000000000000000000000000000000000000000000000000000000000000000000000000000000000000000000000000000000000000

0000000000000000000000000000000000000000000000000000000000000000000000000000000000000000000000000000000000000000000000000000000000000000000000000000000000000000000000000000000
0000000000000000000000000000000000000000000000000000000000000000000000000000000000000000000000000000000000000000000000000000000000000000000000000000000000000000000000000000000
00000000000000000000000000000000000000000000000000000000000000000000000000000
0000000000000000000000000000000000000000000000000000000000000000000000000000000000000000000000000000000000000000000000000000000000000000000000000000000000000000000000000000000
0000000000000000000000000000000000000000000000000000000000000000000000000000000000000000000000000000000000000000000000000000000000000000000000000000000000000000000000000000000
0000000000000000000000000000000000000000000000000000000000000000000000000000000000000000000000000000000000000000000000000000000000000000000000000000000000000000000000000000000
0000000000000000000000000000000000000000000000000000000000000000000000000000000000000000000000000000000000000000000000000000000000000000000000000000000000000000000000000000000
0000000000000000000000000000000000000000000000000000000000000000000000000000000000000000000000000000000000000000000000000000000000000000000000000000000000000000000000000000000
0000000000000000000000000000000000000000000000000000000000000000000000000000000000000000000000000000000000000000000000000000000000000000000000000000000000000000000000000000000
0000000000000000000000000000000000000000000000000000000000000000000000000000000000000000000000000000000000000000000000000000000000000000000000000000000000000000000000000000000
0000000000000000000000000000000000000000000000000000000000000000000000000000000000000000000000000000000000000000000000000000000000000000000000000000000000000000000000000000000
0000000000000000000000000000000000000000000000000000000000000000000000000000000000000000000000000000000000000000000000000000000000000000000000000000000000000000000000000000000
0000000000000000000000000000000000000000000000000000000000000000000000000000000000000000000000000000000000000000000000000000000000000000000000000000000000000000000000000000000
0000000000000000000000000000000000000000000000000000000000000000000000000000000000000000000000000000000000000000000000000000000000000000000000000000000000000000000000000000000
0000000000000000000000000000000000000000000000000000000000000000000000000000000000000000000000000000000000000000000000000000000000000000000000000000000000000000000000000000000
0000000000000000000000000000000000000000000000000000000000000000000000000000000000000000000000000000000000000000000000000000000000000000000000000000000000000000000000000000000
0000000000000000000000000000000000000000000000000000000000000000000000000000000000000000000000000000000000000000000000000000000000000000000000000000000000000000000000000000000
0000000000000000000000000000000000000000000000000000000000000000000000000000000000000000000000000000000000000000000000000000000000000000000000000000000000000000000000000000000
0000000000000000000000000000000000000000000000000000000000000000000000000000000000000000000000000000000000000000000000000000000000000000000000000000000000000000000000000000000
0000000000000000000000000000000000000000000000000000000000000000000000000000000000000000000000000000000000000000000000000000000000000000000000000000000000000000000000000000000
00000000000000000000000000000000000000000000000000000000000000000000000000000
>Q8G9Q2
MRDMRVICDRKKLYKSGKVLVTAGIFALMMFGVTTASVSANTIAVDTNHSRTSAQINKSAVDKVNDDKTTLGAAKVVAVATTPATPVADKTVSAPAADKAVDTTSSTTPATDKAVDTTPTTPAADKAVDTTPTTPAADKAVDTTPTTPAANKAVDTTPATAATDK
AVATPATPAADKLANTTPATDKAVATTPATPVANKAADTSSIHDQPLDTNVPTDKSANLVSTTQKSTDNQQVKSTETSHLQEINGKTYFLDDNGQVKKNFTAIIDGKVLYFDKTSGELTANAPQVTKGLVNIDNAHNAAHDLTADNFTNVDGYLTANSWYRPKDIL
KNGTTWTPTTAEDFRPLLMSWWPDKNTQVAYLQYMQSVGMLPDDVKVSNDDNMSTLTDAAMTVQKNIESRIGVSGKTDWLKQDMNKLIDSQANWNIDSESKGNDHLQGGALLYVNDDKTPNANSDYRLLNRTPTNQTGQITDPSKQGGYEMLLANDVD
NSNPVVQAEQLNWLHYMMNIGTIAQNDPTANFDGYRVDAVDNVDADLLQIAGDYFKAAYGTGKTEANANNHISILEDWDNNDSAYIKAHGNNQLTMDFPAHLALKYALNMPLAAQSGLEPLINTSLVKRGKDATENEAQPNYAFIRAHDSEVQTVIAQIIKDKI
NTKSDGLTVTPDEIKQAFTIYNADELKADKEYTAYNIPASYAVLLTNKDTVPRVYYGDLFSDDGQYMSQKSPYYDAITSLLKSRIKYVAGGQSMNMTYLHECFDPAKNETKPQGVLTSVRYGKGAMTADDLGNSDTRQQGIGLVINNKPFLNLNDDEQIVLNMGAA
HKNQAYRPLMLTTKSGLQIYDKDAGAPVVYTNDAGQLIFKSDMVYGVSNPQVSGYFAAWVPVGASDSQDARTQSSQSETKDGDVYHSNAALDSNVIYEGFSNFQAMPEKNDDFTNVKIAQNAKLFKDLGITSFELAPQYRSSTDNSFLDSVIQNGYAFTDRYDV
GYNTPTKYGTVDQLLDSLRALHAQGIQAINDWVPDQIYNLPGEQIVTAVRTNGSGKYDYDSVINNTLYDSRTVGGGEYQEKFGGLFLDQLKKDYPSLFETKQISTNQPMNPDVKIKEWSAKYFNGSNIQGRGAWYVLKDWATNQYFNVSSDNGFLPKQLLGEKTS
TGFITENGKTSFYSTSGYQAKDTFIQDGTNWYYFDNAGYMLTGKQNIHDKNYYFLPNGVELQDAYLFDGNQEFYYNKAGEQVMNQYYQDSQNQWHYFFENGRMAIGLTEVPNADGTHVTQYFDANGVQIKGTAIKDQNNQLRYFDEATGNMVVNSWGQL
ADKSWLYLNAQGVAVTGNQKIDGEEYYFNADGKQVKGNAIIDNNGDQRYYDGDKGVMVVNSWGELPDGSWLYLNDKGIAVTGRQVINNQVNFFGNDGKQIKDAFKLLSDGSWVYLDDKGLITTGAKVINGLNMFFDKDGHQIKGDASTDANGKRHYYDKN
DGHLVTNSWGELPDGSWLYLEEQGDAVTGQRVIDGKTRYFDEDGKQIKNSLKTLANGDKIYLDGDGVAATGLQHVGDKIMYFDEDGKQVVGKFVSAKDGSWYYLNQDGVAAVGPSSINGQSLYFDQDGKQVKYNEVRNSDGTTNYYTGLTGEKLTQDFGELP
DGSWIYLDAQGHTVTGAQIINGQNLYFKADGQQVKGHAYTDQLGHMRFYDPDSGDMLSNRFEQITPGVWAYFGADGVAITGQHDINGQKLFFDETGYQVKGSQRTIDGTLYSFDSQTGNQKRVQTTLLPQAGHYITKNGNDWQYDTNGELAKGLRQDSNG
KLRYFDLTTGIQAKGQFVTIGQETYYFSKDHGDAQLLPMVTEGHYGTITLKQGQDTKTAWVYRDQNNTILKGLQNINGTLQFFDPYTGEQLKGGVAKYDDKLFYFESGKGNLVSTVAGDYQDGHYISQDGQTRYADKQNQLVKGLVTVNGALQYFDNATGNQIK
NQQVIVDGKTYYFDDKGNGEYLFTNTLDMSTNAFSTKNVAFNHDSSSFDHTVDGFLTADTWYRPKSILANGTTWRDSTDKDMRPLITVWWPNKNVQVNYLNFMKANGLLTTAAQYTLHSDQYDLNQAAQDVQVAIERRIASEHGTDWLQKLLFESQNNNPS
FVKQQFIWNKDSEYHGGGDAWFQGGYLKYGNNPLTPTTNSDYRQPGNAFDFLLANDVDNSNPVVQAENLNWLHYLMNFGTITAGQDDANFDSIRIDAVDFIHNDTIQRTYDYLRDAYQVQQSEAKANQHISLVEAGLDAGTSTIHNDALIESNLREAATLSLTN
EPGKNKPLTNMLQDVDGGTLITDHTQNSTENQATPNYSIIHAHDKGVQEKVGAAITDATGADWTNFTDEQLKAGLELFYKDQRATNKKYNSYNIPSIYALMLTNKDTVPRMYYGDMYQDDGQYMANKSIYYDALVSLMTARKSYVSGGQTMSVDNHGLLKSV
RFGKDAMTANDLGTSATRTEGLGVIIGNDPKLQLNDSDKVTLDMGAAHKNQKYRAVILTTRDGLATFNSDQAPTAWTNDQGTLTFSNQEINGQDNTQIRGVANPQVSGYLAVWVPVGASDNQDARTAATTTENHDGKVLHSNAALDSNLIYEGFSNFQPKAT
THDELTNVVIAKNADVFNNWGITSFEMAPQYRSSGDHTFLDSTIDNGYAFTDRYDLGFNTPTKYGTDGDLRATIQALHHANMQVMADVVDNQVYNLPGKEVVSATRAGVYGNDDATGFGTQLYVTNSVGGGQYQEKYAGQYLEALKAKYPDLFEGKAYDYW
YKNYANDGSNPYYTLSHGDRESIPADVAIKQWSAKYMNGTNVLGNGMGYVLKDWHNGQYFKLDGDKSTLPQI
0000000000000000000000000000000000000000000000000000000000000000000000000000000000000000000000000000000000000000000000000000000000000000000000000000000000000000000000000000000
0000000000000000000000000000000000000000000000000000000000000000000000000000000000000000000000000000000000000000000000000000000000000000000000000000000000000000000000000000000
0000000000000000000000000000000000000000000000000000000000000000000000000000000000000000000000000000000000000000000000000000000000000000000000000000000000000000000000000000000
0000000000000000000000000000000000000000000000000000000000000000000000000000000000000000000000000000000000000000000000000000000000000000000000000000000000000000000000000000000
0000000000000000000000000000000000000000000000000000000000000000000000000000000000000000000000000000000000000000000000000000000000000000000000000000000000000000000000000000000
0000000000000000000000000000000000000000000000000000000000000000000000000000000000000000000000000000000000000000000000000000000000000000000000000000000000000000000000000000000
0000000000000000000000000000000000000000000000000000000000000000000000000000000000000000000000000000000000000000000000000000000000000000000000000000000000000000000000000000000
0000000000000000000000000000000000000000000000000000000000000000000000000000000000000000000000000000000000000000000000000000000000000000000000000000000000000000000000000000000
0000000000000000000000000000000000000000000000000000000000000000000000000000000000000000000000000000000000000000000000000000000000000000000000000000000000000000000000000000000
0000000000000000000000000000000000000000000000000000000000000000000000000000000000000000000000000000000000000000000000000000000000000000000000000000000000000000000000000000000
0000000000000000000000000000000000000000000000000000000000000000000000000000000000000000000000000000000000000000000000000000000000000000000000000000000000000000000000000000000
0000000000000000000000000000000000000000000000000000000000000000000000000000000000000000000000000000000000000000000000000000000000000000000000000000000000000000000000000000000
0000000000000000000000000000000000000000000000000000000000000000000000000000000000000000000000000000000000000000000000000000000000000000000000000000000000000000000000000000000
0000000000000000000000000000000000000000000000000000000000000000000000000000000000000000000000000000000000000000000000000000000000000000000000000000000000000000000000000000000
0000000000000000000000000000000000000000000000000000000000000000000000000000000000000000000000000000000000000000000000000000000000000000000000000000000000000000000000000000000
0000000000000000000000000000000000000000000000000000000000000000000000000000000000000000000000000000000000000000000000000000000000000000000000000000000000000000000000000000000
0000000000000000000000000000000000000000000000000000000000000000000000000000000000000000000000000000000000000000000000000000000000000000000000000000000000000000000000000000000
0000000000000000000
0000000000000000000000000000000000000000000000000000000000000000000000000000000000000000000000000000000000000000000000000000000000000000000000000000000000000000000000000000000
0000000000000000000000000000000000000000000000000000000000000000000000000000000000000000000000000000000000000000000000000000000000000000000000000000000000000000000000000000000
0000000000000000000000000000000000000000000000000000000000000000000000000000000000000000000000000000000000000000000000000000000000000000000000000000000000000000000000000000000
0000000000000000000000000000000000000000000000000000000000000000000000000000000000000000000000000000000000000000000000000000000000000000000000000000000000000000000000000000000
0000000000000000000000000000000000000000000000000000000000000000000000000000000000000000000000000000000000000000000000000000000000000000000000000000000000000000000000000000000
0000000000000000000000000000000000000000000000000000000000000000000000000000000000000000000000000000000000000000000000000000000000000000000000000000000000000000000000000000000
0000000000000000000000000000000000000000000000000000000000000000000000000000000000000000000000000000000000000000000000000000000000000000000000000000000000000000000000000000000
0000000000000000000000000000000000000000000000000000000000000000000000000000000000000000000000000000000000000000000000000000000000000000000000000000000000000000000000000000000
0000000000000000000000000000000000000000000000000000000000000000000000000000000000000000000000000000000000000000000000000000000000000000000000000000000000000000000000000000000
0000000000000000000000000000000000000000000000000000000000000000000000000000000000000000000000000000000000000000000000000000000000000000000000000000000000000000000000000000000
0000000000000000000000000000000000000000000000000000000000000000000000000000000000000000000000000000000000000000000000000000000000000000000000000000000000000000000000000000000
0000000000000000000000000000000000000000000000000000000000000000000000000000000000000000000000000000000000000000000000000000000000000000000000000000000000000000000000000000000
0000000000000000000000000000000000000000000000000000000000000000000000000000000000000000000000000000000000000000000000000000000000000000000000000000000000000000000000000000000
0000000000000000000000000000000000000000000000000000000000000000000000000000000000000000000000000000000000000000000000000000000000000000000000000000000000000000000000000000000
0000000000000000000000000000000000000000000000000000000000000000000000000000000000000000000000000000000000000000000000000000000000000000000000000000000000000000000000000000000
0000000000000000000000000000000000000000000000000000000000000000000000000000000000000000000000000000000000000000000000000000000000000000000000000000000000000000000000000000000
0000000000000000000000000000000000000000000000000000000000000000000000000000000000000000000000000000000000000000000000000000000000000000000000000000000000000000000000000000000
0000000000000000000
0000000000000000000000000000000000000000000000000000000000000000000000000000000000000000000000000000000000000000000000000000000000000000000000000000000000000000000000000000000
0000000000000000000000000000000000000000000000000000000000000000000000000000000000000000000000000000000000000000000000000000000000000000000000000000000000000000000000000000000
0000000000000000000000000000000000000000000000000000000000000000000000000000000000000000000000000000000000000000000000000000000000000000000000000000000000000000000000000000000
0000000000000000000000000000000000000000000000000000000000000000000000000000000000000000000000000000000000000000000000000000000000000000000000000000000000000000000000000000000
0000000000000000000000000000000000000000000000000000000000000000000000000000000000000000000000000000000000000000000000000000000000000000000000000000000000000000000000000000000
0000000000000000000000000000000000000000000000000000000000000000000000000000000000000000000000000000000000000000000000000000000000000000000000000000000000000000000000000000000
0000000000000000000000000000000000000000000000000000000000000000000000000000000000000000000000000000000000000000000000000000000000000000000000000000000000000000000000000000000
0000000000000000000000000000000000000000000000000000000000000000000000000000000000000000000000000000000000000000000000000000000000000000000000000000000000000000000000000000000
0000000000000000000000000000000000000000000000000000000000000000000000000000000000000000000000000000000000000000000000000000000000000000000000000000000000000000000000000000000
0000000000000000000000000000000000000000000000000000000000000000000000000000000000000000000000000000000000000000000000000000000000000000000000000000000000000000000000000000000
0000000000000000000000000000000000000000000000000000000000000000000000000000000000000000000000000000000000000000000000000000000000000000000000000000000000000000000000000000000
0000000000000000000000000000000000000000000000000000000000000000000000000000000000000000000000000000000000000000000000000000000000000000000000000000000000000000000000000000000
0000000000000000000000000000000000000000000000000000000000000000000000000000000000000000000000000000000000000000000000000000000000000000000000000000000000000000000000000000000
0000000000000000000000000000000000000000000000000000000000000000000000000000000000000000000000000000000000000000000000000000000000000000000000000000000000000000000000000000000
0000000000000000000000000000000000000000000000000000000000000000000000000000000000000000000000000000000000000000000000000000000000000000000000000000000000000000000000000000000
0000000000000000000000000000000000000000000000000000000000000000000000000000000000000000000000000000000000000000000000000000000000000000000000000000000000000000000000000000000
0000000000000000000000000000000000000000000000000000000000000000000000000000000000000000000000000000000000000000000000000000000000000000000000000000000000000000000000000000000
0000000000000000000
>Q3JLD6
MNRSYRSIWNEALGAWVAASEISSARGKPNKSAVAKVVTAAVLAVVVQAAHASTIGVGTGSLPGGSACGGIGGTGIGPDGTTNYTNRAITDGSGFYSTVAGCSANGSNALGVTLYGAFTTVTGDGGTAMGFAAYAAKWASAVGLQASATGQSSSAFGFNALAS
GANSLALGSSSTAAYANSVAIGQGSVTGAAAPTGTGYLTGSAAPSSEVSVGSSTNLRRITNVADGSAANDAVTVAQLSTGMSTTTSAIASLSTSTSTGLSSARSSIASLSTSTSTGLSSATSSIASLSTSTSTGIGSLSTGLSTTNSNVASLSSGLSSTNSSLTSLSTSASSGIST
AQSGVNSLSTGLSTTNSTVASLSTSTSTGLSSANSSIASLSTSASSGISTAQSGVNSLSTGLSTTNSTVASLSTSTSTGLSSATSSIASLSTSTSTGIGSLSTGLSTTNSNVASLSSGLSSTNSSLTSLSTSASSGISTAQSGVNSLSTGLSTTNSTVASLSTSTSTGLSSATSSIASLS
TSTSTGIGSLSTGLSTTNSNVASLSSGLSSTNSSLTSLSTSASSGISTAQSGVNSLSTGLSTTNSTVASLSTSTSTGLSSATSSIASLSTSTSTGIGSLSSGLSTTNSNVASLSSGLSSTNSSLTSLSTSASSGISTAQSGVNSLSTGLSTANSTVASLSTSTSTGLSSATSSIASLSTST
STGIGSLSTGLSTTNSNVASLSSGLSSTNSSLTSLSTSASSGISTAQSGVNSLSTGLSTTNSTVASLSTSTSTGLSSATSSIASLSTSTSTGIGSLSTGLSTTNSNVASLSSGLSSTNSSLTSLSTSASSGISTAQSGVNSLSTGLSTANSTVASLSTSTSTGLSSATSSIASLSTSTSTG
IGSLSTGLSTTNSNVASLSSGLSSTNSSLTSLSTSASSGISTAQSGVNSLSTGLSTTNSTVASLSTSTSTGLSSATSSIASLSTSTSTGIGSLSTGLSTVATTTNNLGSSTAAALGGGATYNPATGTISAPSYTTYNANGTTTANTSVGSAIDSINANGIKYFHANSTGPDSVATG
ADAVAIGTGATAATANSVALGANSVTAAAVPTSSATVGSTTLGTFAGSAPVGVVSVGAPSAERQITNVAAGQVTASSTDAINGSQLYAVASKLDSTSSSISSLSTGLSSATSSITSLSTSTSTGLSSANSSIASLSTSTSTGIGSLSTGLSTTDSAVASLSTSTSTGLSSATSSI
TSLSTSTSTGIGSLSTGLSTTNSNVASLSTSTSTGLSSATSSIASLSTSTSTGIGSLSTGLSTTNSTVASLSTSTSTGLSSANSSIASLSTSTSTGIGSLSTGLSTTNSNVASLSSGLSSTNSSLTSLSTSASSGISTAQSGVNSLSTGLSTTNSTVASLSTSTSTGLSSATSSIASLSTSTS
TGIGSLSTGLSTTDSTVASLSTSTSTGLSSATSSIASLSTSTSTGIGSLSTGLSTTNSNVASLSSGLSSTNSSLTSLSTSASSGISTAQSGVNSLSTGLSTANSTVASLSTSTSTGLSSANSSIASLSTSTSTGIGSLSTGLSTTDSTVASLSTSTSTGLSSANSSIASLSTSTSTGIGSLS
TGLSTTDSTVASLSTSTSTGLSSANSSIASLSTSTSTGIGSLSTGLSTTNSNVASLSTSTSTGLSSANSSIASLSTSTSTGIGSLSTGLSTTNSNVASLSTSTSTGLNLANSSIASLSTSTSTSIDSLSTGLSTTNSNVASLSTSTSTGLSSANSSIASLSTSTSTGIGSLSTGLSTTNSNV
ASLSTSTSTGLNLANSSIASLSTSTSTGIGSLSTGLSTTNSNVASLSTSTSTGLSSATSSIASLSTSTSTGIGSLSTGLSTTDSTVASLSTSTSTGLSSANSSIASLSTSTSTGIGSLSTGLSTTDSTVASLSTSTSTGLSSATSSITSLSTSTSTGIGSLSTGLSTTNSNVASLSTSTSTGLS
SATSSIASLSTSTSTGIGSLSTGLSTTNSTVASLSTSTSTGLSSATSSIASLSTSTSTSIGSLSTGLSTTDSTVASLSTSTSTGLSSANSSIASLSTSTSTGIGSLSTGLSTTDSTVASLSTSTSTGLSSATSSIASLSTSTSNGIGSLSTGLSTTNSTVASLSTSTSTGLSSATSSIASLSTST
STGIGSLSTGLSTTNSTVASLSTSTSTGIGSLSTGLSTTNSTVASLSTSTSTGIGSLSTGLSSTNSNLVSLSTSASTGIGSLSTSIDTTNSNLASLSTSSSTGIGSLSTSINATNSNLASLSTSASTGIGSLSTSISSITTNTTNLGNSTAAALGGGATYDPATGAISAPSYTTYNANGT
TATNTSVGAAIDNINANGIKYFHANSTDPDSVATGTNSVAIGPNAVANVDYSVAIGSGATTSAAVPVASASVGGLTFGGFAGSAPIGVFSVGAPGAERQITNVAAGRISAASTDAVNGSQLYATNSNVASLSTGLNATNSNLASLSTSTSTAVGSLSTGLSTTNSTVA
SLSTSTSTSIGSLSTGLSTANSNLASLSTSTSTGIGSLSTGLATTNSNVASLSTSVTNINTQLTSLSTSITNNVIRSLPASTGVAADMSAPKATSPSVTAGSNSVALGAGSNDGGRSNVVSVGSDTQQRQITNVAAGTEGTDAVNVNQLNTLSTSMSQSLSNQQTQLNN
LGSQLNQTQQQLQQTDTMARQGIAAVAAMASIPHMDRDSNFAMGVGTSSFLGQKAIAVGMQARITENLKASLNGGFAGNQKVIGAGMLYQWK
0000000000000000000000000000000000000000000000000000000000000000000000000000000000000000000000000000000000000000000000000000000000000000000000000000000000000000000000000000000
0000000000000000000000000000000000000000000000000000000000000000000000000000000000000000000000000000000000000000000000000000000000000000000000000000000000000000000000000000000
0000000000000000000000000000000000000000000000000000000000000000000000000000000000000000000000000000000000000000000000000000000000000000000000000000000000000000000000000000000
0000000000000000000000000000000000000000000000000000000000000000000000000000000000000000000000000000000000000000000000000000000000000000000000000000000000000000000000000000000
0000000000000000000000000000000000000000000000000000000000000000000000000000000000000000000000000000000000000000000000000000000000000000000000000000000000000000000000000000000
0000000000000000000000000000000000000000000000000000000000000000000000000000000000000000000000000000000000000000000000000000000000000000000000000000000000000000000000000000000
0000000000000000000000000000000000000000000000000000000000000000000000000000000000000000000000000000000000000000000000000000000000000000000000000000000000000000000000000000000
0000000000000000000000000000000000000000000000000000000000000000000000000000000000000000000000000000000000000000000000000000000000000000000000000000000000000000000000000000000
0000000000000000000000000000000000000000000000000000000000000000000000000000000000000000000000000000000000000000000000000000000000000000000000000000000000000000000000000000000
0000000000000000000000000000000000000000000000000000000000000000000000000000000000000000000000000000000000000000000000000000000000000000000000000000000000000000000000000000000
0000000000000000000000000000000000000000000000000000000000000000000000000000000000000000000000000000000000000000000000000000000000000000000000000000000000000000000000000000000
0000000000000000000000000000000000000000000000000000000000000000000000000000000000000000000000000000000000000000000000000000000000000000000000000000000000000000000000000000000
0000000000000000000000000000000000000000000000000000000000000000000000000000000000000000000000000000000000000000000000000000000000000000000000000000000000000000000000000000000
0000000000000000000000000000000000000000000000000000000000000000000000000000000000000000000000000000000000000000000000000000000000000000000000000000000000000000000000000000000
0000000000000000000000000000000000000000000000000000000000000000000000000000000000000000000000000000000000000000000000000000000000000000000000000000000000000000000000000000000
00000000000000000000000000000000000000000000000000000000000000000000000000000000000000000000000000000000000000000000
0000000000000000000000000000000000000000000000000000000000000000000000000000000000000000000000000000000000000000000000000000000000000000000000000000000000000000000000000000000
0000000000000000000000000000000000000000000000000000000000000000000000000000000000000000000000000000000000000000000000000000000000000000000000000000000000000000000000000000000
0000000000000000000000000000000000000000000000000000000000000000000000000000000000000000000000000000000000000000000000000000000000000000000000000000000000000000000000000000000
0000000000000000000000000000000000000000000000000000000000000000000000000000000000000000000000000000000000000000000000000000000000000000000000000000000000000000000000000000000
0000000000000000000000000000000000000000000000000000000000000000000000000000000000000000000000000000000000000000000000000000000000000000000000000000000000000000000000000000000

000000000000000000000000000000000000000000000000000000000000000000000000000000000000000000000000000000000000000000000000000000000000000000000000000000000000000000000000000000
000000000000000000000000000000000000000000000000000000000000000000000000000000000000000000000000000000000000000000000000000000000000000000000000000000000000000000000000000000
000000000000000000000000000000000000000000000000000000000000000000000000000000000000000000000000000000000000000000000000000000000000000000000000000000000000000000000000000000
000000000000000000000000000000000000000000000000000000000000000000000000000000000000000000000000000000000000000000000000000000000000000000000000000000000000000000000000000000
000000000000000000000000000000000000000000000000000000000000000000000000000000000000000000000000000000000000000000000000000000000000000000000000000000000000000000000000000000
000000000000000000000000000000000000000000000000000000000000000000000000000000000000000000000000000000000000000000000000000000000000000000000000000000000000000000000000000000
000000000000000000000000000000000000000000000000000000000000000000000000000000000000000000000000000000000000000000000000000000000000000000000000000000000000000000000000000000
000000000000000000000000000000000000000000000000000000000000000000000000000000000000000000000000000000000000000000000000000000000000000000000000000000000000000000000000000000
000000000000000000000000000000000000000000000000000000000000000000000000000000000000000000000000000000000000000000000000000000000000000000000000000000000000000000000000000000
000000000000000000000000000000000000000000000000000000000000000000000000000000000000000000000000000000000000000000000000000000000000000000000000000000000000000000000000000000
0000000000000000000000000000000000000000000000000000000000000000000000000000000000000000000000000000000000000000000
000000000000000000000000000000000000000000000000000000000000000000000000000000000000000000000000000000000000000000000000000000000000000000000000000000000000000000000000000000
000000000000000000000000000000000000000000000000000000000000000000000000000000000000000000000000000000000000000000000000000000000000000000000000000000000000000000000000000000
000000000000000000000000000000000000000000000000000000000000000000000000000000000000000000000000000000000000000000000000000000000000000000000000000000000000000000000000000000
000000000000000000000000000000000000000000000000000000000000000000000000000000000000000000000000000000000000000000000000000000000000000000000000000000000000000000000000000000
000000000000000000000000000000000000000000000000000000000000000000000000000000000000000000000000000000000000000000000000000000000000000000000000000000000000000000000000000000
000000000000000000000000000000000000000000000000000000000000000000000000000000000000000000000000000000000000000000000000000000000000000000000000000000000000000000000000000000
000000000000000000000000000000000000000000000000000000000000000000000000000000000000000000000000000000000000000000000000000000000000000000000000000000000000000000000000000000
000000000000000000000000000000000000000000000000000000000000000000000000000000000000000000000000000000000000000000000000000000000000000000000000000000000000000000000000000000
000000000000000000000000000000000000000000000000000000000000000000000000000000000000000000000000000000000000000000000000000000000000000000000000000000000000000000000000000000
000000000000000000000000000000000000000000000000000000000000000000000000000000000000000000000000000000000000000000000000000000000000000000000000000000000000000000000000000000
000000000000000000000000000000000000000000000000000000000000000000000000000000000000000000000000000000000000000000000000000000000000000000000000000000000000000000000000000000
000000000000000000000000000000000000000000000000000000000000000000000000000000000000000000000000000000000000000000000000000000000000000000000000000000000000000000000000000000
000000000000000000000000000000000000000000000000000000000000000000000000000000000000000000000000000000000000000000000000000000000000000000000000000000000000000000000000000000
000000000000000000000000000000000000000000000000000000000000000000000000000000000000000000000000000000000000000000000000000000000000000000000000000000000000000000000000000000
000000000000000000000000000000000000000000000000000000000000000000000000000000000000000000000000000000000000000000000000000000000000000000000000000000000000000000000000000000
000000000000000000000000000000000000000000000000000000000000000000000000000000000000000000000000000000000000000000000000000000000000000000000000000000000000000000000000000000
>A0A0K0MCJ4
MISVADLDYASRKSSIFLFAPHVGTFTKQSMDKLVRPLAASAHRDWILDTVAGLPTYWDALAVKIPNIGNAIPGRRQLTDLDTWFRHGAGDVTQDDATLPSIVVGPLVVLIQLTQYWRYLELTRPDHLEDSADLQADVVTRQTQPGAKVETLGFCAGLLAAVAVAS
AGNRQEFQKYGAVAVRLAMMAGALIDGQEARDKATRDGGSVSYAIAWRGQKPGEEAARIVKDLNPNAYFAVLYDEARATVTTTRRTAPSLVNRLRAADVTVAEIGIKGRIHSPDSERKNNTDLLVDLCKSFEDLQYADAASLALPTYNNEAEGRPVSRDRGNMT
EMVIRAILVNQCNWYGTFKGATEGREPFVVTLGLERSVPPTLMRSLGPHQVHYEDLADNGIPPAPQSPPRAVIETQPQPQQQQDTTPPKILENDKDAIAVVGMSIKTAGADDLDEFVEMLKTGRSQHEPVTRERLMHDMLFRAAADSDPNHKYYGCFIRDSDAF
DHRFFKRSPRESAAMDPQSRLAMQAAYQAVEQSGYFTETTAAPEGRDKKHVGVYLGVCGMDYDHNSTCHEPSAFTATGALRSFITGRVSHYFGWTGPSMTFDTACSSSAVAIHTACRNLLSGECTAALAGGANTITSMLWFQNLSAGSFVSPTGQCKPFDDAA
DGYCRAEGMAFVFLKKLSDAVRDGNPVLATIPSTAVYQNQNSTPLFVPNSPSLSMLFSDVMRQAGVAPRDISLVEAHGTGTPVGDPAEYESIRTALGGPRVGRTKPLPIGSVKGHIGHTEGASGAIALIKVIMMMRGGFIPPQASFTKMSHNIAVRPDDMMEVVT
QLRPWADPHKIALLNNYGACGSNASLIVAQPPPALRAPTRGAHQHPEGRRYPFWIAGLDARAISAYAAKLIRYLPSSPQDIPTLADISFSMNRQSNRGLAQGFVFSSRSVAELQEKLAQAAGAAGKEAAALVGIEPVRAERPVVLCFGGQVSLFVGLDRALFDSAAV
LRRHLDECDAAVTALGLDSIYPGIFEREPVQDTVKLQTMLFAMQYASAKSWMDCGLQGKVAAVVGHSFGEITALCVAGVLSLEDTIKLVAARARLVRDSWGPDPGAMMAVEADEGLVRELLQEANRSSDGSAGIACYNGPRSFTLAGSTSAVDAVQATMSADA
KFKAIRSKRLNVTNAFHSALVEKLVDSLGQVGKELTFHPPTIPVERATETPFDAASMDWTFVPRHMREPVFFNHAVQRLAKQHPQAIFLEAGSNSTITIMASRALAQSQTSPPDAHHFQAVSITTATGFDGLTDATVALWKQGLRVAFWAHHALQTLEYAQLLLPP
YQFDTSPSSRHWLEMKSPQKVIDKAVKALVAAGTPHQLPQNQTIDPKTLPLWTFVGYQDAPTNKTARFRINTESAQYNTYVLSHVIAQTAPICPGTLECDMMIEALFSLHADAQENGLQPEIRDLVNHTPICVDPSRTVYLELTALYKKRTQWSAHFLSVANGGGD
AHNPQTHAEARLRLRAAGDADYLREFAQFERLVSHGRCRELLGLGLDVEGVEVLQGRGVYRAFDPVVEYGEVYRGVRYLVGRGNECAGHVQLPARHRGDTWLDVALSDSFSQVGGLWVNLMTDVPPGDMYIATGCEVTMRAPRATARGEGDVWHVYGRHA
RQSDKAYTTDLFVFDAATGSLVEVMLGLQYGRVAKASMSRMLARMTTDETVVRTKASLPQLPATNSSPPATAPAPAMAVVEASRNSKKRSKKTEKKEAKPKKTQKDSGWRDITEEVRNLVAHVSGIEASEIGLDSEMADFGIDSLMGMELGREVELTFKCKLDQA
EQMEATSLRKFVAVVAKALFGTDQPAEVEDEASEAEDEDDDDSSSTGEGGTWSEPSQEDGSKESSGLQTPDSSSYTPPPEKAQKAGPVPGNLKPLNAPTAPPAVSNLVLSPADVLAAFGEVKMATDSLMREYEIDKTERTLLAGSNRLCAALVVEALDELGCPIGTA
AAGQPLERVPFLPQHGRLMQCVYEFLERDARLIDIDVASGQLTRTHVAAPRKTSQAILEDLLTSQPEFAVANRLAYHAGKQLAGVLSGKTDGIRVLFGSPEGREVTAAMYCEHPFNRMSYRQMSDVVEGLAERIQSRGGTGETFKVLEMGAGTGGTTLVMAPLLA
SLEARGIMRVEYTFTDLSPSLVANARRRFGKMYPFMRFAVHDIEKSPAEELRGQHLVLASNAIHATHNLVVSASNVRQALRPDGFVMILEMTEVVPFIDLVFGLLEGWWLFDDGRNHAVVPAEHWERELHTAGFGHVDWTDGNLPENAVQKVIFAMASEPQGP
RLPKAAPEQTMELDRGDVAARTAEAERLVTKYSNAWATPRLQALAARRETNQGQPKRNKANLGAVVLVTGATGSLGSHLVQKLAENPEVAQVVCLNRRSSSGLSAVKKQQEAFASRGITLSPGARAKLRVLDADTSQAQLALPPHEYTWLLQHGTHIVHNAWP
MSGTRPISAFEPQLQGMRNLLDLAREMATRDAVHPFRVGFQFVSSIGVVGYAGEPRILEDRVPLSAVLPSGYGEGKWVCERLLDDTLHKYPSLFRPMVVRPGQISGSSNSGFWNPVEHFAFLVKSAQTLRAWPDFDGVLQWIPVDHCASVMADLLKMGVADAP
DAYPVYHIDNPVGQQWKAMSPVLAAALDIPPHAIIPFSSWIKRVRRSPLLAETENPAARLVDFLDSHFERMSCGGLILDTTKAKEHSQTMAKEGPVDPDLARLYVAAWKKMEFLRS
000000000000000000000000000000000000000000000000000000000000000000000000000000000000000000000000000000000000000000000000000000000000000000000000000000000000000000000000000000
000000000000000000000000000000000000000000000000000000000000000000000000000000000000000000000000000000000000000000000000000000000000000000000000000000000000000000000000000000
000000000000000000000000000000000000000000000000000000000000000000000000000000000000000000000000000000000000000000000000000000000000000000000000000000000000000000000000000000
000000000000000000000000000000000000000000000000000000000000000000000000000000000000000000000000000000000000000000000000000000000000000000000000000000000000000000000000000000
000000000000000000000000000000000000000000000000000000000000000000000000000000000000000000000000000000000000000000000000000000000000000000000000000000000000000000000000000000
000000000000000000000000000000000000000000000000000000000000000000000000000000000000000000000000000000000000000000000000000000000000000000000000000000000000000000000000000000
000000000000000000000000000000000000000000000000000000000000000000000000000000000000000000000000000000000000000000000000000000000000000000000000000000000000000000000000000000
000000000000000000000000000000000000000000000000000000000000000000000000000000000000000000000000000000000000000000000000000000000000000000000000000000000000000000000000000000
000000000000000000000000000000000000000000000000000000000000000000000000000000000000000000000000000000000000000000000000000000000000000000000000000000000000000000000000000000
000000000000000000000000000000000000000000000000000000000000000000000000000000000000000000000000000000000000000000000000000000000000000000000000000000000000000000000000000000
000000000000000000000000000000000000000000000000000000000000000000000000000000000000000000000000000000000000000000000000000000000000000000000000000000000000000000000000000000
000000000000000000000000000000000000000000000000000000000000000000000000000000000000000000000000000000000000000000000000000000000000000000000000000000000000000000000000000000
000000000000000000000000000000000000000000000000000000000000000000000000000000000000000000000000000000000000000000000000000000000000000000000000000000000000000000000000000000
000000000000000000000000000000000000000000000000000000000000000000000000000000000000000000000000000000000000000000000000000000000000000000000000000000000000000000000000000000
000000000000000000000000000000000000000000000000000000000000000000000000000000000000000000000000000000000000000000000000000000000000000000000000000000000000000000000000000000
00000000000000000000000000000000000000000000000000000000000000000000000000000000000000000000000000000000
000000000000000000000000000000000000000000000000000000000000000000000000000000000000000000000000000000000000000000000000000000000000000000000000000000000000000000000000000000
000000000000000000000000000000000000000000000000000000000000000000000000000000000000000000000000000000000000000000000000000000000000000000000000000000000000000000000000000000
000000000000000000000000000000000000000000000000000000000000000000000000000000000000000000000000000000000000000000000000000000000000000000000000000000000000000000000000000000
000000000000000000000000000000000000000000000000000000000000000000000000000000000000000000000000000000000000000000000000000000000000000000000000000000000000000000000000000000
000000000000000000000000000000000000000000000000000000000000000000000000000000000000000000000000000000000000000000000000000000000000000000000000000000000000000000000000000000
000000000000000000000000000000000000000000000000000000000000000000000000000000000000000000000000000000000000000000000000000000000000000000000000000000000000000000000000000000
000000000000000000000000000000000000000000000000000000000000000000000000000000000000000000000000000000000000000000000000000000000000000000000000000000000000000000000000000000
000000000000000000000000000000000000000000000000000000000000000000000000000000000000000000000000000000000000000000000000000000000000000000000000000000000000000000000000000000
000000000000000000000000000000000000000000000000000000000000000000000000000000000000000000000000000000000000000000000000000000000000000000000000000000000000000000000000000000
000000000000000000000000000000000000000000000000000000000000000000000000000000000000000000000000000000000000000000000000000000000000000000000000000000000000000000000000000000
000000000000000000000000000000000000000000000000000000000000000000000000000000000000000000000000000000000000000000000000000000000000000000000000000000000000000000000000000000
000000000000000000000000000000000000000000000000000000000000000000000000000000000000000000000000000000000000000000000000000000000000000000000000000000000000000000000000000000
000000000000000000000000000000000000000000000000000000000000000000000000000000000000000000000000000000000000000000000000000000000000000000000000000000000000000000000000000000
000000000000000000000000000000000000000000000000000000000000000000000000000000000000000000000000000000000000000000000000000000000000000000000000000000000000000000000000000000
000000000000000000000000000000000000000000000000000000000000000000000000000000000000000000000000000000000000000000000000000000000000000000000000000000000000000000000000000000
00000000000000000000000000000000000000000000000000000000000000000000000000000000000000000000000000000000
000000000000000000000000000000000000000000000000000000000000000000000000000000000000000000000000000000000000000000000000000000000000000000000000000000000000000000000000000000
000000000000000000000000000000000000000000000000000000000000000000000000000000000000000000000000000000000000000000000000000000000000000000000000000000000000000000000000000000
000000000000000000000000000000000000000000000000000000000000000000000000000000000000000000000000000000000000000000000000000000000000000000000000000000000000000000000000000000
000000000000000000000000000000000000000000000000000000000000000000000000000000000000000000000000000000000000000000000000000000000000000000000000000000000000000000000000000000
000000000000000000000000000000000000000000000000000000000000000000000000000000000000000000000000000000000000000000000000000000000000000000000000000000000000000000000000000000
000000000000000000000000000000000000000000000000000000000000000000000000000000000000000000000000000000000000000000000000000000000000000000000000000000000000000000000000000000
000000000000000000000000000000000000000000000000000000000000000000000000000000000000000000000000000000000000000000000000000000000000000000000000000000000000000000000000000000
000000000000000000000000000000000000000000000000000000000000000000000000000000000000000000000000000000000000000000000000000000000000000000000000000000000000000000000000000000
000000000000000000000000000000000000000000000000000000000000000000000000000000000000000000000000000000000000000000000000000000000000000000000000000000000000000000000000000000
000000000000000000000000000000000000000000000000000000000000000000000000000000000000000000000000000000000000000000000000000000000000000000000000000000000000000000000000000000
000000000000000000000000000000000000000000000000000000000000000000000000000000000000000000000000000000000000000000000000000000000000000000000000000000000000000000000000000000
000000000000000000000000000000000000000000000000000000000000000000000000000000000000000000000000000000000000000000000000000000000000000000000000000000000000000000000000000000
000000000000000000000000000000000000000000000000000000000000000000000000000000000000000000000000000000000000000000000000000000000000000000000000000000000000000000000000000000
000000000000000000000000000000000000000000000000000000000000000000000000000000000000000000000000000000000000000000000000000000000000000000000000000000000000000000000000000000
000000000000000000000000000000000000000000000000000000000000000000000000000000000000000000000000000000000000000000000000000000000000000000000000000000000000000000000000000000
00000000000000000000000000000000000000000000000000000000000000000000000000000000000000000000000000000000
>Q93008
MTATTRGSPVGGNDNQGQAPDGQSQPPLQQNQTSSPDSSNENSPATPPDEQGQGDAPPQLEDEEPAFPHTDLAKLDDMINRPRWVVPVLPKGELEVLLEAAIDLSKKGLDVKSEACQRFFRDGLTISFTKILTDEAVSGWKFEIHRCIINNTHRLVELCVAKLSQD
WFPLLELLAMALNPHCKFHIYNGTRPCESVSSSVQLPEDELFARSPDPRSPKGWLVDLLNKFGTLNGFQILHDRFINGSALNVQIIAALIKPFGQCYEFLTLHTVKKYFLPIIEMVPQFLENLTDEELKKEAKNEAKNDALSMIIKSLKNLASRVPGQEETVKNLEIFRLKMI
LRLLQISSFNGKMNALNEVNKVISSVSYYTHRHGNPEEEEWLTAERMAEWIQQNNILSIVLRDSLHQPQYVEKLEKILRFVIKEKALTLQDLDNIWAAQAGKHEAIVKNVHDLLAKLAWDFSPEQLDHLFDCFKASWTNASKKQREKLLELIRRLAEDDKDGVMAHK
VLNLLWNLAHSDDVPVDIMDLALSAHIKILDYSCSQDRDTQKIQWIDRFIEELRTNDKWVIPALKQIREICSLFGEAPQNLSQTQRSPHVFYRHDLINQLQHNHALVTLVAENLATYMESMRLYARDHEDYDPQTVRLGSRYSHVQEVQERLNFLRFLLKDGQLWLC
APQAKQIWKCLAENAVYLCDREACFKWYSKLMGDEPDLDPDINKDFFESNVLQLDPSLLTENGMKCFERFFKAVNCREGKLVAKRRAYMMDDLELIGLDYLWRVVIQSNDDIASRAIDLLKEIYTNLGPRLQVNQVVIHEDFIQSCFDRLKASYDTLCVLDGDKDSV
NCARQEAVRMVRVLTVLREYINECDSDYHEERTILPMSRAFRGKHLSFVVRFPNQGRQVDDLEVWSHTNDTIGSVRRCILNRIKANVAHTKIELFVGGELIDPADDRKLIGQLNLKDKSLITAKLTQISSNMPSSPDSSSDSSTGSPGNHGNHYSDGPNPEVESCLPG
VIMSLHPRYISFLWQVADLGSSLNMPPLRDGARVLMKLMPPDSTTIEKLRAICLDHAKLGESSLSPSLDSLFFGPSASQVLYLTEVVYALLMPAGAPLADDSSDFQFHFLKSGGLPLVLSMLTRNNFLPNADMETRRGAYLNALKIAKLLLTAIGYGHVRAVAEACQPG
VEGVNPMTQINQVTHDQAVVLQSALQSIPNPSSECMLRNVSVRLAQQISDEASRYMPDICVIRAIQKIIWASGCGSLQLVFSPNEEITKIYEKTNAGNEPDLEDEQVCCEALEVMTLCFALIPTALDALSKEKAWQTFIIDLLLHCHSKTVRQVAQEQFFLMCTRCCM
GHRPLLFFITLLFTVLGSTARERAKHSGDYFTLLRHLLNYAYNSNINVPNAEVLLNNEIDWLKRIRDDVKRTGETGIEETILEGHLGVTKELLAFQTSEKKFHIGCEKGGANLIKELIDDFIFPASNVYLQYMRNGELPAEQAIPVCGSPPTINAGFELLVALAVGCVRNLKQ
IVDSLTEMYYIGTAITTCEALTEWEYLPPVGPRPPKGFVGLKNAGATCYMNSVIQQLYMIPSIRNGILAIEGTGSDVDDDMSGDEKQDNESNVDPRDDVFGYPQQFEDKPALSKTEDRKEYNIGVLRHLQVIFGHLAASRLQYYVPRGFWKQFRLWGEPVNLREQH
DALEFFNSLVDSLDEALKALGHPAMLSKVLGGSFADQKICQGCPHRYECEESFTTLNVDIRNHQNLLDSLEQYVKGDLLEGANAYHCEKCNKKVDTVKRLLIKKLPPVLAIQLKRFDYDWERECAIKFNDYFEFPRELDMEPYTVAGVAKLEGDNVNPESQLIQQSEQ
SESETAGSTKYRLVGVLVHSGQASGGHYYSYIIQRNGGDGERNRWYKFDDGDVTECKMDDDEEMKNQCFGGEYMGEVFDHMMKRMSYRRQKRWWNAYILFYERMDTIDQDDELIRYISELAITTRPHQIIMPSAIERSVRKQNVQFMHNRMQYSMEYFQF
MKKLLTCNGVYLNPPPGQDHLLPEAEEITMISIQLAARFLFTTGFHTKKVVRGSASDWYDALCILLRHSKNVRFWFAHNVLFNVSNRFSEYLLECPSAEVRGAFAKLIVFIAHFSLQDGPCPSPFASPGPSSQAYDNLSLSDHLLRAVLNLLRREVSEHGRHLQQYFNLF
VMYANLGVAEKTQLLKLSVPATFMLVSLDEGPGPPIKYQYAELGKLYSVVSQLIRCCNVSSRMQSSINGNPPLPNPFGDPNLSQPIMPIQQNVADILFVRTSYVKKIIEDCSNSEETVKLLRFCCWENPQFSSTVLSELLWQVAYSYTYELRPYLDLLLQILLIEDSWQTH
RIHNALKGIPDDRDGLFDTIQRSKNHYQKRAYQCIKCMVALFSNCPVAYQILQGNGDLKRKWTWAVEWLGDELERRPYTGNPQYTYNNWSPPVQSNETSNGYFLERSHSARMTLAKACELCPEEVKKATSVQQIEMEESKEPDDQDAPDEHESPPPEDAPLYPH
SPGSQYQQNNHVHGQPYTGPAAHHMNNPQRTGQRAQENYEGSEEVSPPQTKDQ
000000000000000000000000000000000000000000000000000000000000000000000000000000000000000000000000000000000000000000000000000000000000000000000000000000000000000000000000000000
000000000000000000000000000000000000000000000000000000000000000000000000000000000000000000000000000000000000000000000000000000000000000000000000000000000000000000000000000000
000000000000000000000000000000000000000000000000000000000000000000000000000000000000000000000000000000000000000000000000000000000000000000000000000000000000000000000000000000
000000000000000000000000000000000000000000000000000000000000000000000000000000000000000000000000000000000000000000000000000000000000000000000000000000000000000000000000000000
000000000000000000000000000000000000000000000000000000000000000000000000000000000000000000000000000000000000000000000000000000000000000000000000000000000000000000000000000000
000000000000000000000000000000000000000000000000000000000000000000000000000000000000000000000000000000000000000000000000000000000000000000000000000000000000000000000000000000
000000000000000000000000000000000000000000000000000000000000000000000000000000000000000000000000000000000000000000000000000000000000000000000000000000000000000000000000000000
000000000000000000000000000000000000000000000000000000000000000000000000000000000000000000000000000000000000000000000000000000000000000000000000000000000000000000000000000000
000000000000000000000000000000000000000000000000000000000000000000000000000000000000000000000000000000000000000000000000000000000000000000000000000000000000000000000000000000
000000000000000000000000000000000000000000000000000000000000000000000000000000000000000000000000000000000000000000000000000000000000000000000000000000000000000000000000000000
000000000000000000000000000000000000000000000000000000000000000000000000000000000000000000000000000000000000000000000000000000000000000000000000000000000000000000000000000000
000000000000000000000000000000000000000000000000000000000000000000000000000000000000000000000000000000000000000000000000000000000000000000000000000000000000000000000000000000
000000000000000000000000000000000000000000000000000000000000000000000000000000000000000000000000000000000000000000000000000000000000000000000000000000000000000000000000000000
000000000000000000000000000000000000000000000000000000000000000000000000000000000000000000000000000000000000000000000000000000000000000000000000000000000000000000000000000000
00000000000000000000000000000000000000000000000000000000000000000000000000000000000000000000000000000000
000000000000000000000000000000000000000000000000000000000000000000000000000000000000000000000000000000000000000000000000000000000000000000000000000000000000000000000000000000
000000000000000000000000000000000000000000000000000000000000000000000000000000000000000000000000000000000000000000000000000000000000000000000000000000000000000000000000000000
000000000000000000000000000000000000000000000000000000000000000000000000000000000000000000000000000000000000000000000000000000000000000000000000000000000000000000000000000000

0000000000000000000000000000000000000000000000000000000000000000000000000000000000000000000000000000000000000000000000
0000000000000000000000000000000000000000000000000000000000000000000000000000000000000000000000000000000000000000000000
0000000000000000000000000000000000000000000000000000000000000000000000000000000000000000000000000000000000000000000000
0000000000000000000000000000000000000000000000000000000000000000000000000000000000000000000000000000000000000000000000
0000000000000000000000000000000000000000000000000000000000000000000000000000000000000000000000000000000000000000000000
0000000000000000000000000000000000000000000000000000000000000000000000000000000000000000000000000000000000000000000000
0000000000000000000000000000000000000000000000000000000000000000000000000000000000000000000000000000000000000000000000
0000000000000000000000000000000000000000000000000000000000000000000000000000000000000000000000000000000000000000000000
0000000000000000000000000000000000000000000000000000000000000000000000000000000000000000000000000000000000000000000000
0000000000000000000000000000000000000000000000000000000000000000000000000000000000000000000000000000000000000000000000
0000000000000000000000000000000000000000000000000000000000000000000000000000000000000000000000000000000000000000000000
00000000000000000000000000000000000000000000000000000000000000000000000
0000000000000000000000000000000000000000000000000000000000000000000000000000000000000000000000000000000000000000000000
0000000000000000000000000000000000000000000000000000000000000000000000000000000000000000000000000000000000000000000000
0000000000000000000000000000000000000000000000000000000000000000000000000000000000000000000000000000000000000000000000
0000000000000000000000000000000000000000000000000000000000000000000000000000000000000000000000000000000000000000000000
0000000000000000000000000000000000000000000000000000000000000000000000000000000000000000000000000000000000000000000000
0000000000000000000000000000000000000000000000000000000000000000000000000000000000000000000000000000000000000000000000
0000000000000000000000000000000000000000000000000000000000000000000000000000000000000000000000000000000000000000000000
0000000000000000000000000000000000000000000000000000000000000000000000000000000000000000000000000000000000000000000000
0000000000000000000000000000000000000000000000000000000000000000000000000000000000000000000000000000000000000000000000
0000000000000000000000000000000000000000000000000000000000000000000000000000000000000000000000000000000000000000000000
0000000000000000000000000000000000000000000000000000000000000000000000000000000000000000000000000000000000000000000000
0000000000000000000000000000000000000000000000000000000000000000000000000000000000000000000000000000000000000000000000
0000000000000000000000000000000000000000000000000000000000000000000000000000000000000000000000000000000000000000000000
0000000000000000000000000000000000000000000000000000000000000000000000000000000000000000000000000000000000000000000000
00000000000000000000000000000000000000000000000000000000000000000000000
>E2JF22
MEPHVLGAGLYWLLLPCTLLAASLLRFNALSLVYLLFLLLLPWLPGPSRHSIPGHTGRLLRALLCLSLLFLVAHLAFQICLHTVPHLDQFLGQNGSLWVKVSQHIGVTRLDLKDIFNTTRLVAPDLGVLLASSLCLGLCGRLTRKAGQSRRTQELQDDDDDDDDDDEDID
AAPAVGLKGAPALATKRRLWLASRFRVTAHWLLMTSGRTLVIVLLALAGIAHPSAFSSIYLVVFLAICTWWSCHFPLSPLGFNTLCVMVSCFGAGHLICLYCYQTPFIQDMLPPGNIWARLFGLKNFVDLPNYSSPNALVLNTKHAWPIYVSPGILLLLYYTATSLLKLH
KSCPSELRKETPREDEEHELELDHLEPEPQARDATQGEMPMTTEPDLDNCTVHVLTSQSPVRQRPVRPRLAELKEMSPLHGLGHLIMDQSYVCALIAMMVWSIMYHSWLTFVLLLWACLIWTVRSRHQLAMLCSPCILLYGLTLCCLRYVWAMELPELPTTLGPVS
LHQLGLEHTRYPCLDLGAMLLYLLTFWLLLRQFVKEKLLKKQKVPAALLEVTVADTEPTQTQTLLRSLGELVTGIYVKYWIYVCAGMFIVVSFAGRLVVYKIVYMFLFLLCLTLFQVYYTLWRKLLRVFWWLVVAYTMLVLIAVYTFQFQDFPTYWRNLTGFTDEQLGD
LGLEQFSVSELFSSILIPGFFLLACILQLHYFHRPFMQLTDLEHVPPPGTRHPRWAHRQDAVSEAPLLEHQEEEEVFREDGQSMDGPHQATQVPEGTASKWGLVADRLLDLAASFSAVLTRIQVFVRRLLELHVFKLVALYTVWVALKEVSVMNLLLVVLWAFALPYP
RFRPMASCLSTVWTCIIIVCKMLYQLKIVNPHEYSSNCTEPFPNNTNLQPLEINQSLLYRGPVDPANWFGVRKGYPNLGYIQNHLQILLLLVFEAVVYRRQEHYRRQHQQAPLPAQAVCADGTRQRLDQDLLSCLKYFINFFFYKFGLEICFLMAVNVIGQRMNFMVI
LHGCWLVAILTRRRREAIARLWPNYCLFLTLFLLYQYLLCLGMPPALCIDYPWRWSKAIPMNSALIKWLYLPDFFRAPNSTNLISDFLLLLCASQQWQVFSAERTEEWQRMAGINTDHLEPLRGEPNPIPNFIHCRSYLDMLKVAVFRYLFWLVLVVVFVAGATRISIF
GLGYLLACFYLLLFGTTLLQKDTRAQLVLWDCLILYNVTVIISKNMLSLLSCVFVEQMQSNFCWVIQLFSLVCTVKGYYDPKEMMTRDRDCLLPVEEAGIIWDSICFFFLLLQRRIFLSHYFLHVSADLKATALQASRGFALYNAANLKSINFHRQIEEKSLAQLKRQMKRI
RAKQEKYRQSQASRGQLQSKDPQDPSQEPGPDSPGGSSPPRRQWWRPWLDHATVIHSGDYFLFESDSEEEEEALPEDPRPAAQSAFQMAYQAWVTNAQTVLRQRRERARQERAEQLASGGDLNPDVEPVDVPEDEMAGRSHMMQRVLSTMQFLWVLGQ
ATVDGLTRWLRAFTKHHRTMSDVLCAERYLLTQELLRVGEVRRGVLDQLYVGEDEATLSGPVETRDGPSTASSGLGAEEPLSSMTDDTSSPLSTGYNTRSGSEEIVTDAGDLQAGTSLHGSQELLANARTRMRTASELLLDRRLHIPELEEAERFEAQQGRTLRLLRA
GYQCVAAHSELLCYFIIILNHMVTASAASLVLPVLVFLWAMLTIPRPSKRFWMTAIVFTEVMVVTKYLFQFGFFPWNSYVVLRRYENKPYFPPRILGLEKTDSYIKYDLVQLMALFFHRSQLLCYGLWDHEEDRYPKDHCRSSVKDREAKEEPEAKLESQSETGTGHPK
EPVLAGTPRDHIQGKGSIRSKDVIQDPPEDLKPRHTRHISIRFRRRKETPGPKGTAVMETEHEEGEGKETTERKRPRHTQEKSKFRERMKAAGRRLQSFCVSLAQSFYQPLQRFFHDILHTKYRAATDVYALMFLADIVDIIIIIFGFWAFGKHSAATDIASSLSDDQVP
QAFLFMLLVQFGTMVIDRALYLRKTVLGKLAFQVVLVVAIHIWMFFILPAVTERMFSQNAVAQLWYFVKCIYFALSAYQIRCGYPTRILGNFLTKKYNHLNLFLFQGFRLVPFLVELRAVMDWVWTDTTLSLSNWMCVEDIYANIFIIKCSRETEKKYPQPKGQKKKKI
VKYGMGGLIILFLIAIIWFPLLFMSLIRSVVGVVNQPIDVTVTLKLGGYEPLFTMSAQQPSIVPFTPQAYEELSQQFDPYPLAMQFISQYSPEDIVTAQIEGSSGALWRISPPSRAQMKQELYNGTADITLRFTWNFQRDLAKGGTVEYTNEKHTLELAPNSTARRQLAQ
LLEGRPDQSVVIPHLFPKYIRAPNGPEANPVKQLQPDEEEDYLGVRIQLRREQVGTGASGEQAGTKASDFLEWWVIELQDCKADCNLLPMVIFSDKVSPPSLGFLAGYGIVGLYVSIVLVVGKFVRGFFSEISHSIMFEELPCVDRILKLCQDIFLVRETRELELEEELYAK
LIFLYRSPETMIKWTRERE
0000000000000000000000000000000000000000000000000000000000000000000000000000000000000000000000000000000000000000000000
0000000000000000000000000000000000000000000000000000000000000000000000000000000000000000000000000000000000000000000000
0000000000000000000000000000000000000000000000000000000000000000000000000000000000000000000000000000000000000000000000
0000000000000000000000000000000000000000000000000000000000000000000000000000000000000000000000000000000000000000000000
0000000000000000000000000000000000000000000000000000000000000000000000000000000000000000000000000000000000000000000000
0000000000000000000000000000000000000000000000000000000000000000000000000000000000000000000000000000000000000000000000
0000000000000000000000000000000000000000000000000000000000000000000000000000000000000000000000000000000000000000000000
0000000000000000000000000000000000000000000000000000000000000000000000000000000000000000000000000000000000000000000000
0000000000000000000000000000000000000000000000000000000000000000000000000000000000000000000000000000000000000000000000
0000000000000000000000000000000000000000000000000000000000000000000000000000000000000000000000000000000000000000000000
0000000000000000000000000000000000000000000000000000000000000000000000000000000000000000000000000000000000000000000000
0000000000000000000000000000000000000000000000000000000000000000000000000000000000000000000000000000000000000000000000
0000000000000000000000000000000000000000000000000000000000000000000000000000000000000000000000000000000000000000000000
0000000000000000000000000000000000000000000000000000000000000000000000000000000000000000000000000000000000000000000000
00000000000000000000000000000000000000000000000000000000
0000000000000000000000000000000000000000000000000000000000000000000000000000000000000000000000000000000000000000000000
0000000000000000000000000000000000000000000000000000000000000000000000000000000000000000000000000000000000000000000000
0000000000000000000000000000000000000000000000000000000000000000000000000000000000000000000000000000000000000000000000
0000000000000000000000000000000000000000000000000000000000000000000000000000000000000000000000000000000000000000000000
0000000000000000000000000000000000000000000000000000000000000000000000000000000000000000000000000000000000000000000000
0000000000000000000000000000000000000000000000000000000000000000000000000000000000000000000000000000000000000000000000
0000000000000000000000000000000000000000000000000000000000000000000000000000000000000000000000000000000000000000000000
0000000000000000000000000000000000000000000000000000000000000000000000000000000000000000000000000000000000000000000000
0000000000000000000000000000000000000000000000000000000000000000000000000000000000000000000000000000000000000000000000
0000000000000000000000000000000000000000000000000000000000000000000000000000000000000000000000000000000000000000000000
0000000000000000000000000000000000000000000000000000000000000000000000000000000000000000000000000000000000000000000000
0000000000000000000000000000000000000000000000000000000000000000000000000000000000000000000000000000000000000000000000
0000000000000000000000000000000000000000000000000000000000000000000000000000000000000000000000000000000000000000000000
0000000000000000000000000000000000000000000000000000000000000000000000000000000000000000000000000000000000000000000000
00000000000000000000000000000000000000000000000000000000
0000000000000000000000000000000000000000000000000000000000000000000000000000000000000000000000000000000000000000000000
0000000000000000000000000000000000000000000000000000000000000000000000000000000000000000000000000000000000000000000000
0000000000000000000000000000000000000000000000000000000000000000000000000000000000000000000000000000000000000000000000
0000000000000000000000000000000000000000000000000000000000000000000000000000000000000000000000000000000000000000000000
0000000000000000000000000000000000000000000000000000000000000000000000000000000000000000000000000000000000000000000000
0000000000000000000000000000000000000000000000000000000000000000000000000000000000000000000000000000000000000000000000
0000000000000000000000000000000000000000000000000000000000000000000000000000000000000000000000000000000000000000000000
0000000000000000000000000000000000000000000000000000000000000000000000000000000000000000000000000000000000000000000000
0000000000000000000000000000000000000000000000000000000000000000000000000000000000000000000000000000000000000000000000
0000000000000000000000000000000000000000000000000000000000000000000000000000000000000000000000000000000000000000000000
0000000000000000000000000000000000000000000000000000000000000000000000000000000000000000000000000000000000000000000000
0000000000000000000000000000000000000000000000000000000000000000000000000000000000000000000000000000000000000000000000
0000000000000000000000000000000000000000000000000000000000000000000000000000000000000000000000000000000000000000000000
0000000000000000000000000000000000000000000000000000000000000000000000000000000000000000000000000000000000000000000000
00000000000000000000000000000000000000000000000000000000
>Q8TEP8
MEDFRGIAEESFPSFLTNSLFGNSGILENVTLSSNLGLPVAVSTLARDRSSTDNRYPDIQASYLVEGRFSVPSGSSPGSQSDAEPRERLQLSFQDDDSISRKKSYVESQRLSNALSKQSALQMETAGPEEEPAGATESLQGQDLFNRASPLEQAQDSPIDFHLQSWMN
NKEPKIVVLDAGKHFEDKTLKSDLSHTSLLENEKLILPTSLEDSSDDDIDDEMFYDDHLEAYFEQLAIPGMIYEDLEGPEPPEKGFKLPTNGLRQANENGSLNCKFQSENNSSLISLDSHSSETTHKESEESQVICLPGTSNSIGTGDSRRYTDGMLPFSSGTWGTEKEIEN
LKGIVPDLNSECASKDVLVKTLRAIDVKLNSDNFHDANANRGGFDLTDPVKQGAECPHQNKTVLHMDGCLDTETPTVSIQENVDVASLKPISDSGINFTDAIWSPTCERRTCECHESIEKNKDKTDLPQSVVYQNEEGRWVTDLAYYTSFNSKQNLNVSLSDEMNE
DFRSGSEAFDLIAQDEEEFNKEHQFIQEENIDAHNTSVALGDTSWGATINYSLLRKSRSTSDLDKDDASYLRLSLGEFFAQRSEALGCLGGGNNVKRPSFGYFIRSPEKREPIALIRKSDVSRGNLEKEMAHLNHDLYSGDLNEQSQAQLSEGSITLQVEAVESTSQVDE
NDVTLTADKGKTEDTFFMSNKPQRYKDKLPDSGDSMLRISTIASAIAEASVNTDPSQLAAMIKALSNKTRDKTFQEDEKQKDYSHVRHFLPNDLEKSNGSNALDMEKYLKKTEVSRYESALENFSRASMSDTWDLSLPKEQTTQDIHPVDLSATSVSVRAPEENTAAI
VYVENGESENQESFRTINSSNSVTNRENNSAVVDVKTCSIDNKLQDVGNDEKATSISTPSDSYSSVRNPRITSLCLLKDCEEIRDNRENQRQNECVSEISNSEKHVTFENHRIVSPKNSDLKNTSPEHGGRGSEDEQESFRPSTSPLSHSSPSEISGTSSSGCALESFGSAA
QQQQPPCEQELSPLVCSPAGVSRLTYVSEPESSYPTTATDDALEDRKSDITSELSTTIIQGSPAALEERAMEKLREKVPFQNRGKGTLSSIIQNNSDTRKATETTSLSSKPEYVKPDFRWSKDPSSKSGNLLETSEVGWTSNPEELDPIRLALLGKSGLSCQVGSATSHPVS
CQEPIDEDQRISPKDKSTAGREFSGQVSHQTTSENQCTPIPSSTVHSSVADMQNMPAAVHALLTQPSLSAAPFAQRYLGTLPSTGSTTLPQCHAGNATVCGFSGGLPYPAVAGEPVQNSVAVGICLGSNIGSGWMGTSSLCNPYSNTLNQNLLSTTKPFPVPSVGT
NCGIEPWDSGVTSGLGSVRVPEELKLPHACCVGIASQTLLSVLNPTDRWLQVSIGVLSISVNGEKVDLSTYRCLVFKNKAIIRPHATEEIKVLFIPSSPGVFRCTFSVASWPCSTDAETIVQAEALASTVTLTAIAESPVIEVETEKKDVLDFGDLTYGGWKALPLKLINRTH
ATVPIRLIINANAVAWRCFTFSKESVRAPVEVAPCADVVTRLAGPSVVNHMMPASYDGQDPEFLMIWVLFHSPKKQISSSDILDSAEEFSAKVDIEVDSPNPTPVLRSVSLRARAGIARIHAPRDLQTMHFLAKVASSRKQHLPLKNAGNIEVYLDIKVPEQGSHFSV
DPKNLLLKPGEEHEVIVSFTPKDPEACEERILKIFVQPFGPQYEVVLKGEVISSGSKPLSPGPCLDIPSILSNKQFLAWGGVPLGRTQLQKLALRNNSASTTQHLRLLIRGQDQDCFQLQNTFGSEQRLTSNCEIRIHPKEDIFISVLFAPTRLSCMLARLEIKQLGNRSQPGI
KFTIPLSGYGGTSNLILEGVKKLSDSYMVTVNGLVPGKESKIVFSVRNTGSRAAFVKAVGFKDSQKKVLLDPKVLRIFPDKFVLKERTQENVTLIYNPSDRGINNKTATELSTVYLFGGDEISRQQYRRALLHKPEMIKQILPEHSVLQNINFVEAFQDELLVTEVYDLPQR
PNDVQLFYGSMCKIILSVIGEFRDCISSREFLQPSSKASLESTSDLGASGKHGGNVSLDVLPVKGPQGSPLLSRAARPPLDQLASEEPWTVLPEHLILVAPSPCDMAKTGRFQIVNNSVRLLRFELCWPAHCLTVTPQHGCVAPESKLQILVSPNSSLSTKQSMFPWSGL
IYIHCDDGQKKIVKVQIREDLTQVELLTRLTSKPFGILSPVSEPSVSHLVKPMTKPPSTKVEIRNKSITFPTTEPGETSESCLELENHGTTDVKWHLSSLAPPYVKGVDESGDVFRATYAAFRCSPISGLLESHGIQKVSITFLPRGRGDYAQFWDVECHPLKEPHMKHTLRF
QLSGQSIEAENEPENACLSTDSLIKIDHLVKPRRQAVSEASARIPEQLDVTARGVYAPEDVYRFRPTSVGESRTLKVNLRNNSFITHSLKFLSPREPFYVKHSKYSLRAQHYINMPVQFKPKSAGKFEALLVIQTDEGKSIAIRLIGEALGKN
0000000000000000000000000000000000000000000000000000000000000000000000000000000000000000000000000000000000000000000000
0000000000000000000000000000000000000000000000000000000000000000000000000000000000000000000000000000000000000000000000
0000000000000000000000000000000000000000000000000000000000000000000000000000000000000000000000000000000000000000000000
0000000000000000000000000000000000000000000000000000000000000000000000000000000000000000000000000000000000000000000000
0000000000000000000000000000000000000000000000000000000000000000000000000000000000000000000000000000000000000000000000
0000000000000000000000000000000000000000000000000000000000000000000000000000000000000000000000000000000000000000000000
0000000000000000000000000000000000000000000000000000000000000000000000000000000000000000000000000000000000000000000000
0000000000000000000000000000000000000000000000000000000000000000000000000000000000000000000000000000000000000000000000
0000000000000000000000000000000000000000000000000000000000000000000000000000000000000000000000000000000000000000000000
0000000000000000000000000000000000000000000000000000000000000000000000000000000000000000000000000000000000000000000000
0000000000000000000000000000000000000000000000000000000000000000000000000000000000000000000000000000000000000000000000
0000000000000000000000000000000000000000000000000000000000000000000000000000000000000000000000000000000000000000000000
0000000000000000000000000000000000000000000000000000000000000000000000000000000000000000000000000000000000000000000000
0000000000000000000000000000000000000000000000000000000000000000000000000000000000000000000000000000000000000000000000
0000000000000000000000000000000000000000000000000
0000000000000000000000000000000000000000000000000000000000000000000000000000000000000000000000000000000000000000000000
0000000000000000000000000000000000000000000000000000000000000000000000000000000000000000000000000000000000000000000000
0000000000000000000000000000000000000000000000000000000000000000000000000000000000000000000000000000000000000000000000
0000000000000000000000000000000000000000000000000000000000000000000000000000000000000000000000000000000000000000000000
0000000000000000000000000000000000000000000000000000000000000000000000000000000000000000000000000000000000000000000000
0000000000000000000000000000000000000000000000000000000000000000000000000000000000000000000000000000000000000000000000
0000000000000000000000000000000000000000000000000000000000000000000000000000000000000000000000000000000000000000000000
0000000000000000000000000000000000000000000000000000000000000000000000000000000000000000000000000000000000000000000000

000
000
000
000
000
000
00000000000000000000000000000000000000000000000000000000000000000000000000000000000000000000000000000
000
000
000
000
000
000
000
000
000
000
000
000
000
000
00000000000000000000000000000000000000000000000000000000000000000000000000000000000000000000000000000
>V5M2P5
MDEMPDLSHLTPHERMQIENVLMRQKQEEEKQNEIMRRKQDEVVTLEMQIRQRSEQQKKAGVELDATCHICLKTKFADGVGHICHYCNIRCCARCGGKVTLRSNKVIWVCILCRKKQELLSKTGQWINKTAAQQDGFIRRIEPDGSSDISQHPIVDPHDTTDKRP
KLERTRSAAEKENLPMQRAGSMLRRQYSQQEQPTNRRLSASDSGMDPVMSPGQQMQHHQQQQRARMQPMNPQQAQQGYGMQQQQQPRSGGAYPDDDPRYYQGELDGLMRQHPHLAHPSQVQPQHTPQSHSQQQQSQHQQQHLMPQQHRPSP
QQHSQQQQQQQQHSQQFAARQQQHQQQQHQQQVQQSYHAQIHQQPQQHPHPHSQQQQHHQQMQHQHGQSVPQMGGYQKPPPLTRNLTISGGHAMDSSAYSQQQQQQQRRALAGSQYASQQQRSFSSSEEEFQHQLLAAQGAVTVTAGSDYD
GNFGGVQGVQTVLSPGDLQIHANNPVNWQTSADNTRLIGHMILRKYYDGEDILGLKVNGGQPLATGGSVSGAGTGAAIGAGGPCGAIVEKVKRGSVADLEGRIRPGDEILEWNGRGLHNKSADEVYDIIDESRLDAQVELIVSRPIGSGGGSGGSSANVSPISGAS
GGSANSVPARRSSANFPHSGLASSMAGSAVVSSGGRYLQRKAPAVEAIEIHRDKPSVLITSPGSPDIHTSVSGPGSVSGSGLRQRGGVGQTQRLGPSHSTHSHSSSGSGSGSGSSTSSVHAPASAHGPVHASASASGSAPGGLHGTTTAGHHHHPHPHHYHPHQH
PLPHPHQHQGPPAAHLQGHGVGGGIGMGSGSGTTTQPIPIEGRLQLKLGYDQNTLQLIVTLVCATGLSLRQSGAGRNPYAKVFLLPDRSHKSKRRTKTVGTTCEPRWGQTFVYSGLRRCDLNGRLLEVTLWDYVRYGANDFIGEVVIDLAHHILDDEAEWYQLQP
HQDTSYLLRDEGSDVDGLILTPTDHLSPPSTMSRLSDSDTTSDCDIDGMTPGASISSMGSSASPPPLLELDLNERRSRRDMSPQGRKRVAGMVARDYRTVSGIGQSYHNQASATGYYRRGGGNGVGSAIGPGGMSLSQRSHSAAPSDGYHSSGVGGAASGGPAY
GYRSTSPRRGSLSPPDDRYIDYPVLPVHGSSPNAPSVYQGPGGVSSAAASASQQQRFQSRSATATPTGSPKKRQLPQVPQTSRSAMLRDRLGQDFDERLASGGRFGRHRTRQPHHQATYRSTGMGGWERHYTGLSDSDLHSMDARMRPRHSLSPDKDFMGEF
GDSDMESVVSVTSSAFSTQSERPRTSRGLSFPRNWRNLFGVRNSFLGGAGGEPITGLRLHHSEPGRANTIEVDDCDYLPVGGAQQLVLLEQLEQLQQLAALAAADSPPISAGIGIQPPHQVLVTDANSGQLVEQFYVEPGTMAEEMMEPLDPHLHPHSVFYSPSP
HLPPQPFDIDIDTIYPPRRSNGLKPNVQATAAAYPLPLLPSFEQFKRITTPITNFFRSSSPAGVHPHAHDHTSTTPSSLVGYVRDHQDKSCSHCQNGSQPVVLSTHHTLHLTAAGLAIGACQDPCCLADAHPHPQHQPMGYGEPPSSDPAMCGECVDQHFLGGLTG
PQLNLGVVPTYHVHTPTPNAHRRARGQMATPAPPPTQSVLRLSRQLSEDALVAAVPISEAKAQPTSILKKPKLERRRLFHEGQSRSLDYDDLHTKSWGRRRIRYEDEVMPSSGDYPYPHSNPYSSYSPTAAMSRRYGSETNIRQSYMGANVDANHPLSHSHFLVTD
DKIVTITYDSDVGWTRRGVAPSLFRGAHRQRGLADPRNHMSLDLQRTSQQKLYGPAAPYLKRRRNLPHPPSAQNAHNFSPMEPGSGMDGLNAAAAGMVEQGNGMGSGGAHNTTSTHNSNHHNSQASSVSVSQSHNQQLQQQQQLQQQSVRQKGKEGT
TAISGSMMASEQLTKSSSGSGGATSAAAAGHLVVGGTNANNASSSSFANPQPPPPQQQQQLHGSPNNLNNNNKRAPSSTPQQNTNTINDANNLTLDATQQQSQQQQLQQPPNTCTNQNTNPTTTLTTTTTTSSNATNAATTTTANATNATTTATNPTTTTT
NNTNEPNATTTTTTTNANADADADADADAPLDAIDWDAIDAMLDDDFSEYDKDKNGAEEAGGSKSAEGAGAEDKVDGSLSDTANDRKKGGGVDQERSPKGGSGMGKKSNSTSQLSATGRKGRMGFGKKGKNSFTVHRSEEVLPGDITRELRTGGGLGVSLG
GAGGAAGGSGGAGGGGLSRGSSSEVDAIEQFFGDGAGGERFSPSLRNDGALNEFVDGLGPGQLVGRQVLGAPSLGDIQLSLCHQKGCLEVEVIRARGLQQKAQSKMLPAPYVKVYLVSGKRCVDKMKTSSARRTLDPLYQQQLVFKQSYTGCILQITVWGDYGR
IEKKVFMGVAQIMLDDLNLSNIVIGWYKLFGTTSLVCPTNIGLGSRRSSIASLDSLKL
000
000
000
000
000
000
000
000
000
000
000
000
000
000
0000000000000000000000000000000000
000
000
000
000
000
000
000
000
000
000
000
000
000
000
0000000000000000000000000000000000
000
000
000
000
000
000
000
000
000
000
000
000
000
000
0000000000000000000000000000000000
>Q2FUW1
MSKRQKAFHDSLANEKTRVRLYKSGKNWVKSGIKEIEMFKIMGLPFISHSLVSQDNQSISKKMTGYGLKTTAVIGGAFTVNMLHDQQAFAASDAPLTSELNTQSETVGNQNSTTIEASTSTADSTSVTKNSSSVQTSNSDTVSSEKSEKVTSTTNSTSNQQEKLTSTS
ESTSSKNTTSSSDTKSVASTSSTEQPINTSTNQSTASNNTSQSTTPSSVNLNKTSTTSTSTAPVKLRTFSRLAMSTFASAATTTAVTANTITVNKDNLKQYMTTSGNATYDQSTGIVTLTQDAYSQKGAITLGTRIDSNKSFHFSGKVNLGNKYEGHGNGGDGIGFAFSP
GVLGETGLNGAAVGIGGLSNAFGFKLDTYHNTSKPNSAAKANADPSNVAGGGAFGAFVTTDSYGVATTYTSSSTADNAAKLNVQPTNNTFQDFDINYNGDTKVMTVKYAGQTWTRNISDWIAKSGTTNFSLSMTASTGGATNLQQVQFGTFEYTESAVTQVRY
VDVTTGKDIIPPKTYSGNVDQVVTIDNQQSALTAKGYNYTSVDSSYASTYNDTNKTVKMTNAGQSVTYYFTDVKAPTVTVGNQTIEVGKTMNPIVLTTTDNGTGTVTNTVTGLPSGLSYDSATNSIIGTPTKIGQSTVTVVSTDQANNKSTTTFTINVVDTTAPTVTPI
GDQSSEVYSPISPIKIATQDNSGNAVTNTVTGLPSGLTFDSTNNTISGTPTNIGTSTISIVSTDASGNKTTTTFKYEVTRNSMSDSVSTSGSTQQSQSVSTSKADSQSASTSTSGSIVVSTSASTSKSTSVSLSDSVSASKSLSTSESNSVSSSTSTSLVNSQSVSSSMSDSASK
STSLSDSISNSSSTEKSESLSTSTSDSLRTSTSLSDSLSMSTSGSLSKSQSLSTSISGSSSTSASLSDSTSNAISTSTSLSESASTSDSISISNSIANSQSASTSKSDSQSTSISLSTSDSKSMSTSESLSDSTSTSGSVSGSLSIAASQSVSTSTSDSMSTSEIVSDSISTSGSLSASDSKS
MSVSSSMSTSQSGSTSESLSDSQSTSDSDSKSLSQSTSQSGSTSTSTSTSASVRTSESQSTSGSMSASQSDSMSISTSFSDSTSDSKSASTASSESISQSASTSTSGSVSTSTSLSTSNSERTSTSMSDSTSLSTSESDSISESTSTSDSISEAISASESTFISLSESNSTSDSESQS
ASAFLSESLSESTSESTSESVSSSTSESTSLSDSTSESGSTSTSLSNSTSGSTSISTSTSISESTSTFKSESVSTSLSMSTSTSLSDSTSLSTSLSDSTSDSKSDSLSTSMSTSDSISTSKSDSISTSTSLSGSTSESESDSTSSSESKSDSTSMSISMSQSTSGSTSTSTSTSLSDSTSTSLS
LSASMNQSGVDSNSASQSASNSTSTSTSESDSQSTSSYTSQSTSQSESTSTSTSLSDSTSISKSTSQSGSVSTSASLSGSESESDSQSISTSASESTSESASTSLSDSTSTSNSGSASTSTSLNNSASASESDLSSTSLSDSTSASMQSSESDSQSTSASLSDSLSTSTSNRMSTIA
SLSTSVSTSESGSTSESTSESDSTSTSLSDSQSTSRSTSASGSASTSTSTSDSRSTSASTSTSMRTSTSDSQSMSLSTSTSTSMSDSTSLSDSVSDSTSDSTSASTSGSMSVSISLSDSTSTSTSASEVMSASISDSQSMSESVNDSESVSESNSESDSKSMSGSTSVSDSGSLS
VSTSLRKSESVSESSSLSCSQSMSDSVSTSDSSSLSVSTSLRSSESVSESDSLSDSKSTSGSTSTSTSGSLSTSTSLSGSESVSESTSLSDSISMSDSTSTSDSDSLSGSISLSGSTSLSTSDSLSDSKSLSSSQSMSGSESTSTSVSDSQSSSTSNSQFDSMSISASESDSMSTSDSS
SISGSNSTSTSLSTSDSMSGSVSVSTSTSLSDSISGSTSVSDSSSTSTSTSLSDSMSQSQSTSTSASGSLSTSISTSMSMSASTSSSQSTSVSTSLSTSDSISDSTSISISGSQSTVESESTSDSTSISDSESLSTSDSDSTSTSTSDSTSGSTSTSISESLSTSGSGSTSVSDSTSMSES
NSSSVSMSQDKSDSTSISDSESVSTSTSTSLSTSDSTSTSESLSTSMSGSQSISDSTSTSMSGSTSTSESNSMHPSDSMSMHHTHSTSTSRLSSEATTSTSESQSTLSATSEVTKHNGTPAQSEKRLPDTGDSIKQNGLLGGVMTLLVGLGLMKRKKKKDENDQDDSQA
000
000
000
000
000
000
000
000
000
000
000
000
00
000
000
000
000
000
000
000
000
000
000
000
000
00
000
000
000
000

00000000000000000000000000000000000000000000000000000000000000000000000000000000000000000000000000000000000000000000000000000000000000000000000000000000000
00000000000000000000000000000000000000000000000000000000000000000000000000000000000000000000000000000000000000000000000000000000000000000000000000000000000
00000000000000000000000000000000000000000000000000000000000000000000000000000000000000000000000000000000000000000000000000000000000000000000000000000000000
00000000000000000000000000000000000000000000000000000000000000000000000000000000000000000000000000000000000000000000000000000000000000000000000000000000000
00000000000000000000000000000000000000000000000000000000000000000000000000000000000000000000000000000000000000000000000000000000000000000000000000000000000
00000000000000000000000000000000000000000000000000000000000000000000000000000000000000000000000000000000000000000000000000000000000000000000000000000000000
00000000000000000000000000000000000000000000000000000000000000000000000000000000000000000000000000000000000000000000000000000000000000000000000000000000000
00000000000000000000000000000000000000000000000000000000000000000000000000000000000000000000000000000000000000000000000000000000000000000000000000000000000
00000000000000000000000000000000000000000000000000000000000000000000000000000000000000000000000000000000000000000000000000000000000000000000000000000000000
00000000000000000000000000000000000000000000000000000000000000000000000000000000000000000000000000000000000000000000000000000000000000000000000000000000000
00000000000000000000000000000000000000000000000000000000000000000000000000000000000000000000000000000000000000000000000000000000000000000000000
>Q00416
MNSNNPDNNNSNNINNNNKDKDIAPNSDVQLATVYTKAKSYIPQIEQVYQGTNPNIQEAKLLGELLQVLAEVPKGTHLFCDPILEPISIFSLTIFSFNEEATATWLKNHFNPILSVCDKCILNFARGKCKMLQHFAIQRHVPHEHVAKFNDIVCQWRVEAVFPILRNIS
VNDNTGINITNEIETAMYECLCNPHMLRLNKQLKATFEAIFKFFYDTKHRLLDVTNPLSIKTFISGVIFCWCEGSKEENEWSRAFLKDLYSRNFHINLSNLTPDIIEEVYIHILFLQNPANWTEIVVSQFWSRLLPVFNLFDKDVFIEYFQVPKNVESLKKTFKFPLEPIFKM
WYNHLSKSYHDKPLDFLLRGLTMFLNKFGSEFWSKIEPFTFHSILDIIFNRDSFPIKLIKIQDNPIVEHQTEVYFQLTGSVTDLLSWTLPFYHALSPSKRIQMVRKVSMAFLRIIANYPSLKSIPKACLMNSATALLRAVLTIKENERAMLYKNDEFETVLLTKTDSRALLNNP
LIQDIIIRSASNPNDFYPGLGAASASVATSTMMVLAECIDFDILLLCHRTFKLYSGKPISEIPISTNVLENVTNKIDLRSFHDGPLLAKQLLVSLKNINGLLIVPSNTAVAEAHNALNQKFLLLSTRLMEKFADILPGQLSKILADEDASQGFWSCIFSSDKHLYQAATNILYNT
FDVEGRLEGILAILNSNLTVNLKNINVMLQRLINCEFYEPCPRAVRVLMDVVSAFVDPISGVFANFQTLKSQNTEKEFLKFWESCWLFLDTIYKFTLKWASKYDYSELENFTKDTLDLSRSLVDSFREFSDILHDQTKNLLLNVLETFKNMLYWLRLSDEVLLESCVRLIIST
SDLAHEKHVKVDDSLVEMMAKYASKAKRFSNKLTEQQASEILQKAKIFNKALTEEVATEAENYRKEKELSRLGKVIDLTDSVPASPSLSPSLSSTIASSSAESRADYLQRKALSSSITGRPRVAQPKITSFGTFQSSANAKLHRTKPVKPLSKMELARMQLLNNRVVHPPS
APAFHTKSRGLSNKNDDSSSEESDNDIESARELFAIAKAKGKGIQTVDINGKVVKRQTAAELAKQELEHMRKRLNVDMNPLYEIILQWDYTRNSEYPDDEPIGNYSDVKDFFNSPADYQKVMKPLLLLESWQGLCSSRDREDYKPFSIIVGNRTAVSDFYDVYASVAK
QVIQDCGISESDLIVMAYLPDFRPDKRLSSDDFKKAQHTCLAKVRTLKNTKGGNVDVTLRIHRNHSFSKFLTLRSEIYCVKVMQMTTIEREYSTLEGLEYYDLVGQILQAKPSPPVNVDAAEIETVKKSYKLNTSQAEAIVNSVSKEGFSLIQGPPGTGKTKTILGIIGYFLS
TKNASSSNVIKVPLEKNSSNTEQLLKKQKILICAPSNAAVDEICLRLKSGVYDKQGHQFKPQLVRVGRSDVVNVAIKDLTLEELVDKRIGERNYEIRTDPELERKFNNAVTKRRELRGKLDSESGNPESPMSTEDISKLQLKIRELSKIINELGRDRDEMREKNSVNYRNRD
LDRRNAQAHILAVSDIICSTLSGSAHDVLATMGIKFDTVIIDEACQCTELSSIIPLRYGGKRCIMVGDPNQLPPTVLSGAASNFKYNQSLFVRMEKNSSPYLLDVQYRMHPSISKFPSSEFYQGRLKDGPGMDILNKRPWHQLEPLAPYKFFDIISGRQEQNAKTMSYT
NMEEIRVAIELVDYLFRKFDNKIDFTGKIGIISPYREQMQKMRKEFARYFGGMINKSIDFNTIDGFQGQEKEIILISCVRADDTKSSVGFLKDFRRMNVALTRAKTSIWVLGHQRSLAKSKLWRDLIEDAKDRSCLAYACSGFLDPRNNRAQSILRKFNVPVPSEQEDDY
KLPMEYITQGPDEVKSNKDTKKRRVVDEGEEADKAVKKKKKEKKKEKKKSKADDKKKNNKKAESPSTSSGTKKKSSIFGGMSVPSAVVPKTFPDVDSNKKAAAVVGKKKNNKHVCFSDDVSFIPRNDEPEIKVTRSLSSVLKEKQLGLKETRTISPPEISNNEDDDDED
DYTPSISDSSLMKSEANGRNNRVASHNQNFSASIYDDPQVSQAKQTQVPAAITKHRSSNSVLSGGSSRILTASDYGEPNQNGQNGANRTLSQHVGNANQYSTAPVGTGELHETLPAHPQDSYPAEAEDPYDLNPHPQPQSSAFKGPGSGPTGTRNSSRRNASSS
PFIPKKRKPRS
00000000000000000000000000000000000000000000000000000000000000000000000000000000000000000000000000000000000000000000000000000000000000000000000000000000000
00000000000000000000000000000000000000000000000000000000000000000000000000000000000000000000000000000000000000000000000000000000000000000000000000000000000
00000000000000000000000000000000000000000000000000000000000000000000000000000000000000000000000000000000000000000000000000000000000000000000000000000000000
00000000000000000000000000000000000000000000000000000000000000000000000000000000000000000000000000000000000000000000000000000000000000000000000000000000000
00000000000000000000000000000000000000000000000000000000000000000000000000000000000000000000000000000000000000000000000000000000000000000000000000000000000
00000000000000000000000000000000000000000000000000000000000000000000000000000000000000000000000000000000000000000000000000000000000000000000000000000000000
00000000000000000000000000000000000000000000000000000000000000000000000000000000000000000000000000000000000000000000000000000000000000000000000000000000000
00000000000000000000000000000000000000000000000000000000000000000000000000000000000000000000000000000000000000000000000000000000000000000000000000000000000
00000000000000000000000000000000000000000000000000000000000000000000000000000000000000000000000000000000000000000000000000000000000000000000000000000000000
00000000000000000000000000000000000000000000000000000000000000000000000000000000000000000000000000000000000000000000000000000000000000000000000000000000000
00000000000000000000000000000000000000000000000000000000000000000000000000000000000000000000000000000000000000000000000000000000000000000000000000000000000
00000000000000000000000000000000000000000000000000000000000000000000000000000000000000000000000000000000000000000000000000000000000000000000000000000000000
00000000000000000000000000000000000000000000000000000000000000000000000000000000000000000000000000000000000000000000000000000000000000000000000000000000000
00000000000000000000000000000000000000000000000000000000000000000000000000000000000000000000000000000000000000000000000000000000000000000000000000000000000
00000000000000000000000000000000000000000000000000000000000000000000000000000000000000000000000000000000000000000000000000000000000000000000000000000000000
000000000000000000000000000000000000000000000000000000000000000000000000000000000000000000000000000000000
00000000000000000000000000000000000000000000000000000000000000000000000000000000000000000000000000000000000000000000000000000000000000000000000000000000000
00000000000000000000000000000000000000000000000000000000000000000000000000000000000000000000000000000000000000000000000000000000000000000000000000000000000
00000000000000000000000000000000000000000000000000000000000000000000000000000000000000000000000000000000000000000000000000000000000000000000000000000000000
00000000000000000000000000000000000000000000000000000000000000000000000000000000000000000000000000000000000000000000000000000000000000000000000000000000000
00000000000000000000000000000000000000000000000000000000000000000000000000000000000000000000000000000000000000000000000000000000000000000000000000000000000
00000000000000000000000000000000000000000000000000000000000000000000000000000000000000000000000000000000000000000000000000000000000000000000000000000000000
00000000000000000000000000000000000000000000000000000000000000000000000000000000000000000000000000000000000000000000000000000000000000000000000000000000000
00000000000000000000000000000000000000000000000000000000000000000000000000000000000000000000000000000000000000000000000000000000000000000000000000000000000
00000000000000000000000000000000000000000000000000000000000000000000000000000000000000000000000000000000000000000000000000000000000000000000000000000000000
00000000000000000000000000000000000000000000000000000000000000000000000000000000000000000000000000000000000000000000000000000000000000000000000000000000000
00000000000000000000000000000000000000000000000000000000000000000000000000000000000000000000000000000000000000000000000000000000000000000000000000000000000
00000000000000000000000000000000000000000000000000000000000000000000000000000000000000000000000000000000000000000000000000000000000000000000000000000000000
00000000000000000000000000000000000000000000000000000000000000000000000000000000000000000000000000000000000000000000000000000000000000000000000000000000000
00000000000000000000000000000000000000000000000000000000000000000000000000000000000000000000000000000000000000000000000000000000000000000000000000000000000
00000000000000000000000000000000000000000000000000000000000000000000000000000000000000000000000000000000000000000000000000000000000000000000000000000000000
000000000000000000000000000000000000000000000000000000000000000000000000000000000000000000000000000000000
00000000000000000000000000000000000000000000000000000000000000000000000000000000000000000000000000000000000000000000000000000000000000000000000000000000000
00000000000000000000000000000000000000000000000000000000000000000000000000000000000000000000000000000000000000000000000000000000000000000000000000000000000
00000000000000000000000000000000000000000000000000000000000000000000000000000000000000000000000000000000000000000000000000000000000000000000000000000000000
00000000000000000000000000000000000000000000000000000000000000000000000000000000000000000000000000000000000000000000000000000000000000000000000000000000000
00000000000000000000000000000000000000000000000000000000000000000000000000000000000000000000000000000000000000000000000000000000000000000000000000000000000
00000000000000000000000000000000000000000000000000000000000000000000000000000000000000000000000000000000000000000000000000000000000000000000000000000000000
00000000000000000000000000000000000000000000000000000000000000000000000000000000000000000000000000000000000000000000000000000000000000000000000000000000000
00000000000000000000000000000000000000000000000000000000000000000000000000000000000000000000000000000000000000000000000000000000000000000000000000000000000
00000000000000000000000000000000000000000000000000000000000000000000000000000000000000000000000000000000000000000000000000000000000000000000000000000000000
00000000000000000000000000000000000000000000000000000000000000000000000000000000000000000000000000000000000000000000000000000000000000000000000000000000000
00000000000000000000000000000000000000000000000000000000000000000000000000000000000000000000000000000000000000000000000000000000000000000000000000000000000
00000000000000000000000000000000000000000000000000000000000000000000000000000000000000000000000000000000000000000000000000000000000000000000000000000000000
00000000000000000000000000000000000000000000000000000000000000000000000000000000000000000000000000000000000000000000000000000000000000000000000000000000000
00000000000000000000000000000000000000000000000000000000000000000000000000000000000000000000000000000000000000000000000000000000000000000000000000000000000
00000000000000000000000000000000000000000000000000000000000000000000000000000000000000000000000000000000000000000000000000000000000000000000000000000000000
000000000000000000000000000000000000000000000000000000000000000000000000000000000000000000
>Q6GWS2
MEEDIACVKDLVSKYLVDNERLSRQKLAFLVQTEPRMLLMEGLKLLSLCIEVDSCNANGCEHNSEDKSVERILHDHGILTPSLCFVVPDGYKLTGNVLILLECFVRSSPANFEQKYIEDFKKLEQLKEDLKSVDINLIPLIDGRTSFYNEQIPDWVNDKLRDTLFSLLKYAQE
SNSLFEESEYSRLCESLSMTSGRLSGVESLNVLMDNRSNHYEEVIASCHQGINNKLTAHEVKLQIEEEYQVFRNRLRKGEIEGQFLKVDKSQLLNELNNLYADKVVAEDNIEHLIYQFKRASPILRFLYANVDEGNEKRENQTIGECQVQCWRSFLNKVKSLRILNTRRKL
LLIFDALILLASKHDLMKQKCLKGWLGSCFLSVNDRLVSLESTKRDLEKWVERRQQAERSRITQSSQCLSKNQILNSIFQKTILKATTALKDVGISVDHYKIDMEVICLNSYDLIMDFDVSGVVPTISYQRTEEETFPYVMGDVELLGTTDLERLSSLSLALVNSMKTSSTV
KLRQNEFGPARYQVVRCKEAYCQEFSLGNTELQLIYQKTGECSKCYAINDNKVGEVCSFYADPKRYFPAIFSAEVLQTTISTMISWIEDCNELEGQLNNIRSLTKMILVLILAHPSKRSQKLLQNLRYFVMAYVSDYHHKDLIDKIREELITDVEFLLYRLIRTLMNLVLSED
VKSMMTNRFKFILNVSYMCHFITKETPDRLTDQIKCFEKFLEPKVRFGHVSTNPADTATEEELDDMVYNAKKFLSKDGCTSIEGPDYKRPGVSKKYLSLLTSSFNNGSLFKEREVKREIKDPLITSGCATALDLASNKSVVVNKYTDGSRILNYDFNKLTALAVSQLTEVFS
RKGKYLLNKQDYEYKVQQAMSNLVLGSGQLKSDADGADLDEILLDGGASDYFDQLKETVEKIVDQYREPVKLGSGPNGDGQPSINDLDEIVSNKFYIRLIKGELSNHMVEDFDHDILPGKFYEEFCNAVYENSRLKQKYFYCGHMSQCPIGELTKAVSTRTYFNHEYF
QCFKSILLIMNANTLMGRYTHYKSRNLNFKFDMGKLSDDVRISERESNSEALSKALSLTNCTTAMLKNLCFYSQESPQSYDSVGPDTGRLKFSLSYKEQVGGNRELYIGDLRTKMFTRLIEDYFEAISLQLSGSCLNNEKEFENAILSMKLNVSLAHVSYSMDHSKWGP
MMCPFLFLTVLQNLIFLSKDLQADIKGRDYLSTLLMWHMHKMVEIPFNVVTAMMKSFIKAQLGLRKKTKQSITEDFFYSNFQVGVVPSHISSILDMGQGILHNTSDFYALISERFINYAISCICGGTIDAYTSSDDQISLFDQSLTELLQRDPEEFRTLIEFHYYMSDQLN
KFVSPKSVIGRFVAEFKSRFFVWGDEVPLLTKFVAAALHNIKCKEPHQLAETIDTIIDQSVANGVPVHLCNLIQKRTLNLLQYARYPIDPFLLNCETDVRDWVDGNRSYRIMRQIEGLIPNACSKIRSMLRKLYNRLKTGQLHEEFTTNYLSSEHLSSLRNLCELLDVEPPS
ESDLEYSWLNLAAHHPLRMVLRQKIIYSGAVNLDDEKIPTIVKTIQNKLSSTFTRGAQKLLSEAINKSAFQSSIASGFVGLCRTLGSKCVRGPNKENLYIKSIQSLISDVKGIKLLTNSNGIQYWQVPLELRNGSGSESVVSYFRPLLWDYMCISLSTAIELGAWVLGEPKTV
KVLDFFKHNPCDYFPLKPTASKLLEDRVGLNHIIHSLRRLYPSVFEKHILPFMSDLASTKMKWSPRIKFLDLCVALDVNCEALSLVSHIVKWKREEHYIVLSSELRLSHSRTHEPMVEERVVSTSDAVDNFMRQIYFESYVRPFVATTRTLGSFTWFPHKTSVPEGEGLHR
MGPFSSFVEKVIHKGVERPMFKHDLMMGYAWIDFDIEPARFNQNQLIASGLVDPKFDSLEDFFDAVASLPPGSAKLSQTVRFRVKSQDASFKESFAIHLEYTGSMNQQAKYLVHDVTVMYSGAVSPCVLSDCWRLVLSGPTFKGKSAWYVDTEIINEFLIDTNQLG
HVTPVEIVVDMERLQFTEYDFVLVGPCTEPTPLVVHRGGLWECGKKLASFTPVIQDQDLEIFVREVGDTSSDLLIGALSDMMIDRLGLRMQWSGVDIVSTLRAAAPSCEGILSAVLEAVDNWVEFKGYALCYSKSRGKVMVQSSGGKLRLKGRTCEELTRKDECIEDI
E
00000000000000000000000000000000000000000000000000000000000000000000000000000000000000000000000000000000000000000000000000000000000000000000000000000000000
00000000000000000000000000000000000000000000000000000000000000000000000000000000000000000000000000000000000000000000000000000000000000000000000000000000000
00000000000000000000000000000000000000000000000000000000000000000000000000000000000000000000000000000000000000000000000000000000000000000000000000000000000
00000000000000000000000000000000000000000000000000000000000000000000000000000000000000000000000000000000000000000000000000000000000000000000000000000000000
00000000000000000000000000000000000000000000000000000000000000000000000000000000000000000000000000000000000000000000000000000000000000000000000000000000000
00000000000000000000000000000000000000000000000000000000000000000000000000000000000000000000000000000000000000000000000000000000000000000000000000000000000
00000000000000000000000000000000000000000000000000000000000000000000000000000000000000000000000000000000000000000000000000000000000000000000000000000000000
00000000000000000000000000000000000000000000000000000000000000000000000000000000000000000000000000000000000000000000000000000000000000000000000000000000000
00000000000000000000000000000000000000000000000000000000000000000000000000000000000000000000000000000000000000000000000000000000000000000000000000000000000
00000000000000000000000000000000000000000000000000000000000000000000000000000000000000000000000000000000000000000000000000000000000000000000000000000000000
00000000000000000000000000000000000000000000000000000000000000000000000000000000000000000000000000000000000000000000000000000000000000000000000000000000000
00000000000000000000000000000000000000000000000000000000000000000000000000000000000000000000000000000000000000000000000000000000000000000000000000000000000
00000000000000000000000000000000000000000000000000000000000000000000000000000000000000000000000000000000000000000000000000000000000000000000000000000000000
00000000000000000000000000000000000000000000000000000000000000000000000000000000000000000000000000000000000000000000000000000000000000000000000000000000000
00000000000000000000000000000000000000000000000000000000000000000000000000000000000000000000000000000000000000000000000000000000000000000000000000000000000
00000000000000000000000000000000000000000000000000000000000000000000000000000000000000000000000
00000000000000000000000000000000000000000000000000000000000000000000000000000000000000000000000000000000000000000000000000000000000000000000000000000000000
00000000000000000000000000000000000000000000000000000000000000000000000000000000000000000000000000000000000000000000000000000000000000000000000000000000000
00000000000000000000000000000000000000000000000000000000000000000000000000000000000000000000000000000000000000000000000000000000000000000000000000000000000
00000000000000000000000000000000000000000000000000000000000000000000000000000000000000000000000000000000000000000000000000000000000000000000000000000000000
00000000000000000000000000000000000000000000000000000000000000000000000000000000000000000000000000000000000000000000000000000000000000000000000000000000000
00000000000000000000000000000000000000000000000000000000000000000000000000000000000000000000000000000000000000000000000000000000000000000000000000000000000
00000000000000000000000000000000000000000000000000000000000000000000000000000000000000000000000000000000000000000000000000000000000000000000000000000000000
00000000000000000000000000000000000000000000000000000000000000000000000000000000000000000000000000000000000000000000000000000000000000000000000000000000000
00000000000000000000000000000000000000000000000000000000000000000000000000000000000000000000000000000000000000000000000000000000000000000000000000000000000
00000000000000000000000000000000000000000000000000000000000000000000000000000000000000000000000000000000000000000000000000000000000000000000000000000000000
00000000000000000000000000000000000000000000000000000000000000000000000000000000000000000000000000000000000000000000000000000000000000000000000000000000000
00000000000000000000000000000000000000000000000000000000000000000000000000000000000000000000000000000000000000000000000000000000000000000000000000000000000
00000000000000000000000000000000000000000000000000000000000000000000000000000000000000000000000000000000000000000000000000000000000000000000000000000000000
00000000000000000000000000000000000000000000000000000000000000000000000000000000000000000000000000000000000000000000000000000000000000000000000000000000000
00000000000000000000000000000000000000000000000000000000000000000000000000000000000000000000000000000000000000000000000000000000000000000000000000000000000
00000000000000000000000000000000000000000000000000000000000000000000000000000000000000000000000
00000000000000000000000000000000000000000000000000000000000000000000000000000000000000000000000000000000000000000000000000000000000000000000000000000000000
00000000000000000000000000000000000000000000000000000000000000000000000000000000000000000000000000000000000000000000000000000000000000000000000000000000000
00000000000000000000000000000000000000000000000000000000000000000000000000000000000000000000000000000000000000000000000000000000000000000000000000000000000
00000000000000000000000000000000000000000000000000000000000000000000000000000000000000000000000000000000000000000000000000000000000000000000000000000000000
00000000000000000000000000000000000000000000000000000000000000000000000000000000000000000000000000000000000000000000000000000000000000000000000000000000000
00000000000000000000000000000000000000000000000000000000000000000000000000000000000000000000000000000000000000000000000000000000000000000000000000000000000
00000000000000000000000000000000000000000000000000000000000000000000000000000000000000000000000000000000000000000000000000000000000000000000000000000000000
00000000000000000000000000000000000000000000000000000000000000000000000000000000000000000000000000000000000000000000000000000000000000000000000000000000000
00000000000000000000000000000000000000000000000000000000000000000000000000000000000000000000000000000000000000000000000000000000000000000000000000000000000
00000000000000000000000000000000000000000000000000000000000000000000000000000000000000000000000000000000000000000000000000000000000000000000000000000000000
00000000000000000000000000000000000000000000000000000000000000000000000000000000000000000000000000000000000000000000000000000000000000000000000000000000000
00000000000000000000000000000000000000000000000000000000000000000000000000000000000000000000000000000000000000000000000000000000000000000000000000000000000
00000000000000000000000000000000000000000000000000000000000000000000000000000000000000000000000000000000000000000000000000000000000000000000000000000000000
00000000000000000000000000000000000000000000000000000000000000000000000000000000000000000000000000000000000000000000000000000000000000000000000000000000000
00000000000000000000000000000000000000000000000000000000000000000000000000000000000000000000000000000000000000000000000000000000000000000000000000000000000
00000000000000000000000000000000000000000000000000000000000000000000000000000000000000000000000
>P96202
MTAATPDRRAIITEALHKIDDLTARLEIAEKSSSEPIAVIGMGCRFPGGVNNPEQFWDLLCAGRSGIVRVPAQRWDADAYYCDDHTVPGTICSTEGGFLTSWQPDEFDAEFFSISPREAAAMDPQQRLLIEVAWEALEDAGVPQHTIRGTQTSVFVGVTAYDYMLT
LAGRLRPVDLDAYIPTGNSANFAAGRLAYILGARGPAVVIDTACSSSLVAVHLACQSLRGRESDMALVGGTNLLLSPGPSIACSRWGMLSPEGRCKTFDASADGYVRGEGAAVVVLKRLDDAVRDGNRILAVVRGSAVNQDGASSGVTVPNGPAQQALLAKALTS
SKLTAADIDYVEAHGTGTPLGDPIELDSLSKVFSDRAGSDQLVIGSVKTNLGHLEAAAGVAGLMKAVLAVHNGYIPRHLNFHQLTPHASEAASRLRIAADGIDWPTTGRPRRAGVSSFGVSGTNAHVVIEQAPDPMAAAGTEPQRGPVPAVSTLVVFGKTAPRVAA
TASVLADWLDGPGAAVPLADVAHTLNHHRARQTRFGTVAAVDRRQAVIGLRALAAGQSAPGVVAPREGSIGGGTVFVYSGRGSQWAGMGRQLLADEPAFAAAIAELEPEFVAQGGFSLRDVIAGGKELVGIEQIQLGLIGMQLALTALWRSYGVTPDAVIGHS
MGEVAAAVVAGALTPAQGLRVTAVRSRLMAPLSGQGTMALLELDAEATEALIADYPEVSLGIYASPRQTVISGPPLLIDELIDKVRQQNGFATRVNIEVAPHNPAMDALQPAMRSELADLTPQPPTIPIISTTYADLGISLGSGPRFDAEHWATNMRNPVRFHQAIA
HAGADHHTFIEISAHPLLTHSISDTLRASYDVDNYLSIGTLQRDAHDTLEFHTNLNTTHTTHPPQTPHPPEPHPVLPTTPWQHTQHWITATSAAYHRPDTHPLLGVGVTDPTNGTRVWESELDPDLLWLADHVIDDLVVLPGAAYAEIALAAATDTFAVEQDQPW
MISELDLRQMLHVTPGTVLVTTLTGDEQRCQVEIRTRSGSSGWTTHATATVARAEPLAPLDHEGQRREVTTADLEDQLDPDDLYQRLRGAGQQHGPAFQGIVGLAVTQAGVARAQVRLPASARTGSREFMLHPVMMDIALQTLGATRTATDLAGGQDARQGP
SSNSALVVPVRFAGVHVYGDITRGVRAVGSLAAAGDRLVGEVVLTDANGQPLLVVDEVEMAVLGSGSGATELTNRLFMLEWEPAPLEKTAEATGALLLIGDPAAGDPLLPALQSSLRDRITDLELASAADEATLRAAISRTSWDGIVVVCPPRANDESMPDEAQLEL
ARTRTLLVASVVETVTRMGARKSPRLWIVTRGAAQFDAGESVTLAQTGLRGIARVLTFEHSELNTTLVDIEPDGTGSLAALAEELLAGSEADEVALRDGQRYVNRLVPAPTTTSGDLAAEARHQVVNLDSSGASRAAVRLQIDQPGRLDALNVHEVKRGRPQGDQV
EVRVVAAGLNFSDVLKAMGVYPGLDGAAPVIGGECVGYVTAIGDEVDGVEVGQRVIAFGPGPTGFGTHLGTIADLVVPIPDTLADNEAATFGVAYLTAWHSLCEVGRLSPGERVLIHSATGGVGMAAVSIAKMIGARIYTTAGSDAKREMLSRLGVEYVGDSRSVDFA

DEILELTDGYGVDVVLNSLAGEAIQRGVQILAPGGRFIELGKKDVYADASLGLAALAKSASFSVVDLDLNLKLQPARYRQLLQHILQHVADGKLEVLPVTAFSLHDAADAFRLMASGKHTGKIVISIPQHGSIEAIAAPPPLPLVSRDGGYLIVGGMGGLGFVVARWLA
EQGAGLIVLNGRSAPSDEVAAAIAELNASGSRIEVITGDITEPDTAERLVRAVEDAGFRLAGVVHSAMVLADEIVLNMTDSAARRVFAPKVTGSWRLHVATAARDVDWWLTFSSAAALLGTPGQGAYAAANSWVDGLVAHRRSAGLPAVGINWGPWADVGRA
QFFKDLGVEMINAEQGLAAMQAVLTADRGRTGVFSLDARQWFQSFPAVAGSSLFAKLHDSAARKSGQRRGGGAIRAQLDALDAAERPGHLASAIADEIRAVLRSGDPIDHHRPLETLGLDSLMGLELRNRLEASLGITLPVALVWAYPTISDLATALCERMDYATP
AAAQEISDTEPELSDEEMDLLADLVDASELEAATRGES
00000000000000000000000000000000000000000000000000000000000000000000000000000000000000000000000000000000000000000000000000000000000000000000000000000000000000000000000000000000000000000000000000000
00000000000000000000000000000000000000000000000000000000000000000000000000000000000000000000000000000000000000000000000000000000000000000000000000000000000000000000000000000000000000000000000000000
00000000000000000000000000000000000000000000000000000000000000000000000000000000000000000000000000000000000000000000000000000000000000000000000000000000000000000000000000000000000000000000000000000
00000000000000000000000000000000000000000000000000000000000000000000000000000000000000000000000000000000000000000000000000000000000000000000000000000000000000000000000000000000000000000000000000000
00000000000000000000000000000000000000000000000000000000000000000000000000000000000000000000000000000000000000000000000000000000000000000000000000000000000000000000000000000000000000000000000000000
00000000000000000000000000000000000000000000000000000000000000000000000000000000000000000000000000000000000000000000000000000000000000000000000000000000000000000000000000000000000000000000000000000
00000000000000000000000000000000000000000000000000000000000000000000000000000000000000000000000000000000000000000000000000000000000000000000000000000000000000000000000000000000000000000000000000000
00000000000000000000000000000000000000000000000000000000000000000000000000000000000000000000000000000000000000000000000000000000000000000000000000000000000000000000000000000000000000000000000000000
00000000000000000000000000000000000000000000000000000000000000000000000000000000000000000000000000000000000000000000000000000000000000000000000000000000000000000000000000000000000000000000000000000
00000000000000000000000000000000000000000000000000000000000000000000000000000000000000000000000000000000000000000000000000000000000000000000000000000000000000000000000000000000000000000000000000000
00000000000000000000000000000000000000000000000000000000000000000000000000000000000000000000000000000000000000000000000000000000000000000000000000000000000000000000000000000000000000000000000000000
00000000000000000000000000000000000000000000000000000000000000000000000000000000000000000000000000000000000000000000000000000000000000000000000000000000000000000000000000000000000000000000000000000
00000000000000000000000000000000000000000000000000000000000000000000000000000000000000000000000000000000000000000000000000000000000000000000000000000000000000000000000000000000000000000000000000000
00000000000000000000000000000000000000000000000000000000000000000000000000000000000000000000000000000000000000000000000000000000000000000000000000000000000000000000000000000000000000000000000000000
00000000000000000000000000000000000000000000000000000000000000000000000000000000000000000000000000000000000000000000000000000000000000000000000000000000000000000000000000000000000000000000000000000
00000000000000000000000000000000000000000000000000000000000000000000000000000000000000000000000000
00000000000000000000000000000000000000000000000000000000000000000000000000000000000000000000000000000000000000000000000000000000000000000000000000000000000000000000000000000000000000000000000000000
00000000000000000000000000000000000000000000000000000000000000000000000000000000000000000000000000000000000000000000000000000000000000000000000000000000000000000000000000000000000000000000000000000
00000000000000000000000000000000000000000000000000000000000000000000000000000000000000000000000000000000000000000000000000000000000000000000000000000000000000000000000000000000000000000000000000000
00000000000000000000000000000000000000000000000000000000000000000000000000000000000000000000000000000000000000000000000000000000000000000000000000000000000000000000000000000000000000000000000000000
00000000000000000000000000000000000000000000000000000000000000000000000000000000000000000000000000000000000000000000000000000000000000000000000000000000000000000000000000000000000000000000000000000
00000000000000000000000000000000000000000000000000000000000000000000000000000000000000000000000000000000000000000000000000000000000000000000000000000000000000000000000000000000000000000000000000000
00000000000000000000000000000000000000000000000000000000000000000000000000000000000000000000000000000000000000000000000000000000000000000000000000000000000000000000000000000000000000000000000000000
00000000000000000000000000000000000000000000000000000000000000000000000000000000000000000000000000000000000000000000000000000000000000000000000000000000000000000000000000000000000000000000000000000
00000000000000000000000000000000000000000000000000000000000000000000000000000000000000000000000000000000000000000000000000000000000000000000000000000000000000000000000000000000000000000000000000000
00000000000000000000000000000000000000000000000000000000000000000000000000000000000000000000000000000000000000000000000000000000000000000000000000000000000000000000000000000000000000000000000000000
00000000000000000000000000000000000000000000000000000000000000000000000000000000000000000000000000000000000000000000000000000000000000000000000000000000000000000000000000000000000000000000000000000
00000000000000000000000000000000000000000000000000000000000000000000000000000000000000000000000000000000000000000000000000000000000000000000000000000000000000000000000000000000000000000000000000000
00000000000000000000000000000000000000000000000000000000000000000000000000000000000000000000000000000000000000000000000000000000000000000000000000000000000000000000000000000000000000000000000000000
00000000000000000000000000000000000000000000000000000000000000000000000000000000000000000000000000000000000000000000000000000000000000000000000000000000000000000000000000000000000000000000000000000
00000000000000000000000000000000000000000000000000000000000000000000000000000000000000000000000000000000000000000000000000000000000000000000000000000000000000000000000000000000000000000000000000000
00000000000000000000000000000000000000000000000000000000000000000000000000000000000000000000000000
00000000000000000000000000000000000000000000000000000000000000000000000000000000000000000000000000000000000000000000000000000000000000000000000000000000000000000000000000000000000000000000000000000
00000000000000000000000000000000000000000000000000000000000000000000000000000000000000000000000000000000000000000000000000000000000000000000000000000000000000000000000000000000000000000000000000000
00000000000000000000000000000000000000000000000000000000000000000000000000000000000000000000000000000000000000000000000000000000000000000000000000000000000000000000000000000000000000000000000000000
00000000000000000000000000000000000000000000000000000000000000000000000000000000000000000000000000000000000000000000000000000000000000000000000000000000000000000000000000000000000000000000000000000
00000000000000000000000000000000000000000000000000000000000000000000000000000000000000000000000000000000000000000000000000000000000000000000000000000000000000000000000000000000000000000000000000000
00000000000000000000000000000000000000000000000000000000000000000000000000000000000000000000000000000000000000000000000000000000000000000000000000000000000000000000000000000000000000000000000000000
00000000000000000000000000000000000000000000000000000000000000000000000000000000000000000000000000000000000000000000000000000000000000000000000000000000000000000000000000000000000000000000000000000
00000000000000000000000000000000000000000000000000000000000000000000000000000000000000000000000000000000000000000000000000000000000000000000000000000000000000000000000000000000000000000000000000000
00000000000000000000000000000000000000000000000000000000000000000000000000000000000000000000000000000000000000000000000000000000000000000000000000000000000000000000000000000000000000000000000000000
00000000000000000000000000000000000000000000000000000000000000000000000000000000000000000000000000000000000000000000000000000000000000000000000000000000000000000000000000000000000000000000000000000
00000000000000000000000000000000000000000000000000000000000000000000000000000000000000000000000000000000000000000000000000000000000000000000000000000000000000000000000000000000000000000000000000000
00000000000000000000000000000000000000000000000000000000000000000000000000000000000000000000000000000000000000000000000000000000000000000000000000000000000000000000000000000000000000000000000000000
00000000000000000000000000000000000000000000000000000000000000000000000000000000000000000000000000000000000000000000000000000000000000000000000000000000000000000000000000000000000000000000000000000
00000000000000000000000000000000000000000000000000000000000000000000000000000000000000000000000000000000000000000000000000000000000000000000000000000000000000000000000000000000000000000000000000000
00000000000000000000000000000000000000000000000000000000000000000000000000000000000000000000000000000000000000000000000000000000000000000000000000000000000000000000000000000000000000000000000000000
00000000000000000000000000000000000000000000000000000000000000000000000000000000000000000000000000
>A0AEF4
MKKLLSAVTSAVMSTSLMTGAFASSVSAAGSYAAAQPNVSMGGVLDVAANKTAGEDVVFDFGKWTANAGETIDVDVFVDTQGKAVSAMDVRFQIDSPLKITAMEKEAPAFNTTAMVNLSNLGLNFKSLSDKGEPLTPINNEPVFMLAVEVPANTPSGDYKIGF
GDKCEVHKSNDGSKYSTAAINGVITVKGTNDTPSTTASTSKNTNTSNTTVSSQTPSTPTQTSGTPSAPSKDGEVMFDFQNYTAKAGDEVVVDVLVDTQGKPVSAMDVKFKVDSPLKISEIDKESLAFNTTAMTNMAILGANFKSLDDKGEPLVPKAGAAVFTLYVD
VPANTPDGTYYVGFNGKNEVHKSNDGSQYTVGYKNGAITVGNPTTGSGSQPTPTTQTSSKTPTNTTTTAPVANADVVFDFQNYTAKAGDEVTVDVLVDSKNKPISAMDVKFKVDSPLTIEEIDKESLAFNTTVMTNMAILGANFKSLDDKGEPLVPKDGAAVFTLY
VNVPANTPDGTYYVGFNGKNEVHKSNDGSQFTVASKNGAITVGTPNEEGSTTPKATTDNPTTTVTTNKPAESGEAVFDFGNYTAKPGDEVAVDVTVDTAGGKVCAMDVVFKIDSPLQIVDIDKESQAFKTTAMTNLAVLGENFKSLDDKGEPLVPTSDPVFTFYV
KVPENTEDGVYNVGFGNKHEVVKDNSNAQYKVSTKNGKITVANPQAATTTANQSSTTASATTKATSATSAEVTTTKNVTPATGSAEWVIPTVNAKPGEKVTMDVVVKNSAIEVAGAQFNIKQTAPIAYGSAASGDAYAAIVPNETEQYYAFGEGIGKGIKAADGA
KIITLTFNVPADCAKGTYPVKWSNAFITDTNGNKITDKITLTDGAIVVGDTPVVAEGTAEWVIPTVNAKPGEKVTMNVVVKDSAVEVAGAQFNIKQTAPITYGSSTSGDAYAAIVPNETEQYYAFGEGIGKGIKAADGATIITLTFNVPADCTKGTYPVKWSNAFITDT
NGNKITDKITLTDGAIVVGDTPVTEDGKAEWVIPTVNAEPGDKVTMNVIVKDSAAEVAGAQFNIKQTAPITYGSSTSGDAYAAIVPNETEQYYAFGEGIGKGVKAAEGATIITLTFNVPADCADGTYPVKWSNAFITDTNGKKITDNITLTDGAIIVGKVTTASTEANN
TTTSTTKVTEVDTDTTLQSTVSSTESTDATNTTTATTIVTSPRPTDIVLPEGGIAWQAGTTEAKPGDVVTIDIKIVDTEGVKLPVAGASFTVVNEGDIQATDVSKDENGYNSSVIYNPTTHEFAFAEGTGKNVASEDGKIVATITYKIADNCAPGEYKVDLGGLKVFDTTG
KDITSLVAPYDGLIKVVGDPVDSTTTTTTVTGSDTPDTTTTTTTTVTGSDTPDTTTTTTVTGSNTPDTTTTTTVTGSDTPDTTTTTTVTGSNTPDTTTTTTVTGSNTPDTTTTTTVTGSNTPDTTTTTTTVTGSNTPDPVTTSTTATGVQPGTSTTTSAKGSVKWVGETVE
ATPGETVKVKFVIEDDGSGLPVAGAQFTIKSPVGVGKVDDGFTNFYGASIVANPTEGQYAFANPVGAGIKSENGKTVIELEYTIPADCTPGEYAVDIDNLKVFDTNGNDITDLVTGKDGKIIVKTPDSTTTTTTTGTGTDETTTTTTVTGSDTPDTTTTTTVTGSNTPDT
TTTTTVTGSDTPDTTTTTTVTGSNTPDTTTTTTVTGSNTPDTTTTTTVTGSNTPDTTTTTTVTGSNTPDTTTTTTVTGSNTPDTTTTTTVTGSNTPDTTTTTTVTGSNTPDTTTSTSATTSDTDTGTTTTSTGPVNPGTEPHSTTPTTATNSGDNVSVYYTIETVAGYYFS
HDTGVRGNGEAGGFDKNQVVKITKYTKDKNGNIIAINDLDLANVNYNGYTPNKAYIDRFGDPAQNPTDQTLANFADNFAYDIPVYYGGDQLVDENGQPLTVKAYIGVKGDTNLDFIVDGRDATATLTYYARVSTDNYTEADTPISPAPFITGADDPLDDLAAFLSD
VDTNEWDKDNWKLAREDRILDGRDATNILTYYARASAGDGEYAGLDAQELWNTVVPNRFG
00000000000000000000000000000000000000000000000000000000000000000000000000000000000000000000000000000000000000000000000000000000000000000000000000000000000000000000000000000000000000000000000000000
00000000000000000000000000000000000000000000000000000000000000000000000000000000000000000000000000000000000000000000000000000000000000000000000000000000000000000000000000000000000000000000000000000
00000000000000000000000000000000000000000000000000000000000000000000000000000000000000000000000000000000000000000000000000000000000000000000000000000000000000000000000000000000000000000000000000000
00000000000000000000000000000000000000000000000000000000000000000000000000000000000000000000000000000000000000000000000000000000000000000000000000000000000000000000000000000000000000000000000000000
00000000000000000000000000000000000000000000000000000000000000000000000000000000000000000000000000000000000000000000000000000000000000000000000000000000000000000000000000000000000000000000000000000
00000000000000000000000000000000000000000000000000000000000000000000000000000000000000000000000000000000000000000000000000000000000000000000000000000000000000000000000000000000000000000000000000000
00000000000000000000000000000000000000000000000000000000000000000000000000000000000000000000000000000000000000000000000000000000000000000000000000000000000000000000000000000000000000000000000000000
00000000000000000000000000000000000000000000000000000000000000000000000000000000000000000000000000000000000000000000000000000000000000000000000000000000000000000000000000000000000000000000000000000
00000000000000000000000000000000000000000000000000000000000000000000000000000000000000000000000000000000000000000000000000000000000000000000000000000000000000000000000000000000000000000000000000000
00000000000000000000000000000000000000000000000000000000000000000000000000000000000000000000000000000000000000000000000000000000000000000000000000000000000000000000000000000000000000000000000000000
00000000000000000000000000000000000000000000000000000000000000000000000000000000000000000000000000000000000000000000000000000000000000000000000000000000000000000000000000000000000000000000000000000
000000000000000000000000000000000000000000000000000000000000000000000000000000000000000000000000000000000000000000000000000000000000000000000000000000000
00000000000000000000000000000000000000000000000000000000000000000000000000000000000000000000000000000000000000000000000000000000000000000000000000000000000000000000000000000000000000000000000000000
00000000000000000000000000000000000000000000000000000000000000000000000000000000000000000000000000000000000000000000000000000000000000000000000000000000000000000000000000000000000000000000000000000
00000000000000000000000000000000000000000000000000000000000000000000000000000000000000000000000000000000000000000000000000000000000000000000000000000000000000000000000000000000000000000000000000000
00000000000000000000000000000000000000000000000000000000000000000000000000000000000000000000000000000000000000000000000000000000000000000000000000000000000000000000000000000000000000000000000000000
00000000000000000000000000000000000000000000000000000000000000000000000000000000000000000000000000000000000000000000000000000000000000000000000000000000000000000000000000000000000000000000000000000
00000000000000000000000000000000000000000000000000000000000000000000000000000000000000000000000000000000000000000000000000000000000000000000000000000000000000000000000000000000000000000000000000000
00000000000000000000000000000000000000000000000000000000000000000000000000000000000000000000000000000000000000000000000000000000000000000000000000000000000000000000000000000000000000000000000000000
00000000000000000000000000000000000000000000000000000000000000000000000000000000000000000000000000000000000000000000000000000000000000000000000000000000000000000000000000000000000000000000000000000
00000000000000000000000000000000000000000000000000000000000000000000000000000000000000000000000000000000000000000000000000000000000000000000000000000000000000000000000000000000000000000000000000000
00000000000000000000000000000000000000000000000000000000000000000000000000000000000000000000000000000000000000000000000000000000000000000000000000000000000000000000000000000000000000000000000000000
00000000000000000000000000000000000000000000000000000000000000000000000000000000000000000000000000000000000000000000000000000000000000000000000000000000000000000000000000000000000000000000000000000
000000000000000000000000000000000000000000000000000000000000000000000000000000000000000000000000000000000000000000000000000000000000000000000000000000000
00000000000000000000000000000000000000000000000000000000000000000000000000000000000000000000000000000000000000000000000000000000000000000000000000000000000000000000000000000000000000000000000000000
00000000000000000000000000000000000000000000000000000000000000000000000000000000000000000000000000000000000000000000000000000000000000000000000000000000000000000000000000000000000000000000000000000
00000000000000000000000000000000000000000000000000000000000000000000000000000000000000000000000000000000000000000000000000000000000000000000000000000000000000000000000000000000000000000000000000000
00000000000000000000000000000000000000000000000000000000000000000000000000000000000000000000000000000000000000000000000000000000000000000000000000000000000000000000000000000000000000000000000000000
00000000000000000000000000000000000000000000000000000000000000000000000000000000000000000000000000000000000000000000000000000000000000000000000000000000000000000000000000000000000000000000000000000
00000000000000000000000000000000000000000000000000000000000000000000000000000000000000000000000000000000000000000000000000000000000000000000000000000000000000000000000000000000000000000000000000000
00000000000000000000000000000000000000000000000000000000000000000000000000000000000000000000000000000000000000000000000000000000000000000000000000000000000000000000000000000000000000000000000000000
00000000000000000000000000000000000000000000000000000000000000000000000000000000000000000000000000000000000000000000000000000000000000000000000000000000000000000000000000000000000000000000000000000
00000000000000000000000000000000000000000000000000000000000000000000000000000000000000000000000000000000000000000000000000000000000000000000000000000000000000000000000000000000000000000000000000000
00000000000000000000000000000000000000000000000000000000000000000000000000000000000000000000000000000000000000000000000000000000000000000000000000000000000000000000000000000000000000000000000000000
00000000000000000000000000000000000000000000000000000000000000000000000000000000000000000000000000000000000000000000000000000000000000000000000000000000000000000000000000000000000000000000000000000
000000000000000000000000000000000000000000000000000000000000000000000000000000000000000000000000000000000000000000000000000000000000000000000000000000000
>Q9VG78
MSSSVFTEAPSARPAAAEADSNLVVVYSKSGISFDERAIGNIMEEKSISTISVVRITSPTPSMVDQEEDERLEELANFMETATDSELDSDNGSQSGGDNASEMDEHDEENHNTGDDNNSDSTETEADAPRTRKRVGRKPKAIKKRKRRKPKERPQIVGLRVEQFYAD
NTADMVVKNALKLAGVSVYKRTAETDALMEVIRNDHNYTPFTSPEQLKNHKRDEKMAMEMQAQSRKIIMQAPGSVKLINAKKRVQAIPLSPVQVKVQRLPVKPHQVQQAAQLQPKVQLIRKPIHSPLPRQKPEIIANNSVNARLRPTRAPVKVIAPPVQEYIEDD
DDAQSSDSAEEENADKAYDSEEMSEESDEFQESDNDRDSDMDFDMRHSNRRNPNKRKRVRKVVRQTPSKPMKPLPPPPTTPQHFPELKKRKEVLEPKAPQIGQIIQMPATKSVPAVFALGRGLPSTSFLKIRPTHQSLPVKRPPLPTTPANSNVRRMPAVQLGRI
VNSPQEVKEIIINKNMASPKGVFTNLNTLLGEDNNAATKSSPVPLRHRAVMSPSTPRSSYNQQMSPIVVPVPAQAHTPSKGFMPIGVDTAQSHKLPAQIVIETHQSSSELAAENDKQLDLINSIVQDELLKSTLVEQPVVNADEDIPKLVKMLESTAADLDPPPVSLP
TNIFPAPEMQGVGNAAAADPNNIMDTANEDEITADFLQHVVGLIEEDKQFEAEVVKQVLASTEPGTLDAIVSMPTSIEPVDVPQAHTNLLPNASLTEPAQSMTSLPIACSTPSRSVAASTPPTSAKVVRGYGRVIYLPPIEAPTTRAKRRAQFPSAPGMAATSSSDAG
NLSFGESSLDASINQPLNTSSLSNDSQPGSGPKRPNPREPSMARRSTAPRRSKKLDASQNNDPDASESQEDDDDPNKLWCICRQPHNNRFMICCDLCEDWFHGTCVGVTKAMGTDMENKGIDWKCPKCVKRQEERSQPRITDMLVTRPTTQPEQRPSETKVLT
TTAEIVQVAAPSAPRRTLPVVLTVASSPMRIPMAKPAKKFPTGAISHQQQQQLNFIRLGPSPGKRISETLCVVCKRPASTSSVYCGEECIRKYAQSAIQAHAATKGPLPQNAGAQSLLNNSFDAKKNKKKDLFEDVLRQADTVSKVERINVFERKSGRVITGHMAPSA
HQFRKWLQENPSFEVLPSGTVQSADAEKRLLKGAPEAATSTSEPAVLGVAKKPPEGPAKLSHPQNTTVQASHQLGISSVRPLAKKDKEKTTPTVQAPTPNRIAAGKPEPVRIGIRRSLREQLLARIKEAQAAEENSGQPTTQWPTVLEVDQFVKNVELEMFNSFGRD
VGAKYKAKYRSLMFNIKDRKNRTLFEKICAKQVEPRQLVRMTPEQLASQELAKWREEENRHQLDMIKKSELDLLACAQNYVVKTHKGEEVIATKVDVTLPEENVSEASSMADTKQTSLVSEPSTSTLERSTSREKAGSSKEKRHKSHKHHHRKRSRSRSNSRGRSVDK
RHRRQHNEGGEQPGSGEREHRSREKQSRERDEIVPSPLPKKRDENDQSPVPKKTAEKKTEASAYNLVDQILESEKTVEQAANLGKPKPSPKPLPTLPSSLKAPEPMDNYSRYLHGLTTSSLWSGTLKMIDLADFEIVMYPVQGNCHQLGNLMPSQMDVIGRITRVN
VWEYIKKLKKSPTKEVVIVNIFPASPSETYKFDLFFEYLDSRQRLGVLGVDSDQIRDFYIFPLGSGDKLPPALQTAEPVPFYEEAQRPNTLLGIIVRCLSKRPADAVPSTPSVPTPVPALSSKVAKRSRSSISYPAQSSPKRKISTHSTSSKDDEFDIDAIIKAPIAKLQKTAPKVV
PQPSVPNDADEPYSPGGSDDELVPSAPQRPNDLERQVNEINKQIAAQQMEIAGLLKVEPTGSASSSNVLAAISIPANLSKILASIKDKTNLPSSADDGDEEYNPEDDITATSSFASKKPRSKGRLAHLSEAELLSMVPDNLAGVIPRSSRTRHQLAQSPLTPTPLPPPPPP
PGV
00000000000000000000000000000000000000000000000000000000000000000000000000000000000000000000000000000000000000000000000000000000000000000000000000000000000000000000000000000000000000000000000000000
00000000000000000000000000000000000000000000000000000000000000000000000000000000000000000000000000000000000000000000000000000000000000000000000000000000000000000000000000000000000000000000000000000
00000000000000000000000000000000000000000000000000000000000000000000000000000000000000000000000000000000000000000000000000000000000000000000000000000000000000000000000000000000000000000000000000000
00000000000000000000000000000000000000000000000000000000000000000000000000000000000000000000000000000000000000000000000000000000000000000000000000000000000000000000000000000000000000000000000000000
00000000000000000000000000000000000000000000000000000000000000000000000000000000000000000000000000000000000000000000000000000000000000000000000000000000000000000000000000000000000000000000000000000
00000000000000000000000000000000000000000000000000000000000000000000000000000000000000000000000000000000000000000000000000000000000000000000000000000000000000000000000000000000000000000000000000000
00000000000000000000000000000000000000000000000000000000000000000000000000000000000000000000000000000000000000000000000000000000000000000000000000000000000000000000000000000000000000000000000000000
00000000000000000000000000000000000000000000000000000000000000000000000000000000000000000000000000000000000000000000000000000000000000000000000000000000000000000000000000000000000000000000000000000
00000000000000000000000000000000000000000000000000000000000000000000000000000000000000000000000000000000000000000000000000000000000000000000000000000000000000000000000000000000000000000000000000000
00000000000000000000000000000000000000000000000000000000000000000000000000000000000000000000000000000000000000000000000000000000000000000000000000000000000000000000000000000000000000000000000000000
00000000000000000000000000000000000000000000000000000000000000000000000000000000000000000000000000000000000000000000000000000000000000000000000000000000000000000000000000000000000000000000000000000
0000000000000000000000000000000000000000000000000000000000000000000000000000000000000000000
00000000000000000000000000000000000000000000000000000000000000000000000000000000000000000000000000000000000000000000000000000000000000000000000000000000000000000000000000000000000000000000000000000
00000000000000000000000000000000000000000000000000000000000000000000000000000000000000000000000000000000000000000000000000000000000000000000000000000000000000000000000000000000000000000000000000000
00000000000000000000000000000000000000000000000000000000000000000000000000000000000000000000000000000000000000000000000000000000000000000000000000000000000000000000000000000000000000000000000000000
00000000000000000000000000000000000000000000000000000000000000000000000000000000000000000000000000000000000000000000000000000000000000000000000000000000000000000000000000000000000000000000000000000
00000000000000000000000000000000000000000000000000000000000000000000000000000000000000000000000000000000000000000000000000000000000000000000000000000000000000000000000000000000000000000000000000000
00000000000000000000000000000000000000000000000000000000000000000000000000000000000000000000000000000000000000000000000000000000000000000000000000000000000000000000000000000000000000000000000000000
00000000000000000000000000000000000000000000000000000000000000000000000000000000000000000000000000000000000000000000000000000000000000000000000000000000000000000000000000000000000000000000000000000
00000000000000000000000000000000000000000000000000000000000000000000000000000000000000000000000000000000000000000000000000000000000000000000000000000000000000000000000000000000000000000000000000000
00000000000000000000000000000000000000000000000000000000000000000000000000000000000000000000000000000000000000000000000000000000000000000000000000000000000000000000000000000000000000000000000000000

00000000000000000000000000000000000000000000000000000000000000000000000000000000000000000000000000000000000000000000000000000000000000000000000000000000000000000000000000
00000000000000000000000000000000000000000000000000000000000000000000000000000000000000000000000000000000000000000000000000000000000000000000000000000000000000000000000000
0000000000000000000000000000000000000000000000000000000000000000000000000000000
00000000000000000000000000000000000000000000000000000000000000000000000000000000000000000000000000000000000000000000000000000000000000000000000000000000000000000000000000
00000000000000000000000000000000000000000000000000000000000000000000000000000000000000000000000000000000000000000000000000000000000000000000000000000000000000000000000000
00000000000000000000000000000000000000000000000000000000000000000000000000000000000000000000000000000000000000000000000000000000000000000000000000000000000000000000000000
00000000000000000000000000000000000000000000000000000000000000000000000000000000000000000000000000000000000000000000000000000000000000000000000000000000000000000000000000
00000000000000000000000000000000000000000000000000000000000000000000000000000000000000000000000000000000000000000000000000000000000000000000000000000000000000000000000000
00000000000000000000000000000000000000000000000000000000000000000000000000000000000000000000000000000000000000000000000000000000000000000000000000000000000000000000000000
00000000000000000000000000000000000000000000000000000000000000000000000000000000000000000000000000000000000000000000000000000000000000000000000000000000000000000000000000
00000000000000000000000000000000000000000000000000000000000000000000000000000000000000000000000000000000000000000000000000000000000000000000000000000000000000000000000000
00000000000000000000000000000000000000000000000000000000000000000000000000000000000000000000000000000000000000000000000000000000000000000000000000000000000000000000000000
00000000000000000000000000000000000000000000000000000000000000000000000000000000000000000000000000000000000000000000000000000000000000000000000000000000000000000000000000
00000000000000000000000000000000000000000000000000000000000000000000000000000000000000000000000000000000000000000000000000000000000000000000000000000000000000000000000000
0000000000000000000000000000000000000000000000000000000000000000000000000000000
>A0A0H2YST8
MKRKMIIKKLSVLTLGTIFTSNILLPSTLIYAFPTEGINHNIEKEEKKEIKESNYSQIGGVENFTQNENDVLLDLSTGEKIRISFLKEDVLRIYMDPTGEFQEEPTPNSKDHITKIINKTEEEYENPTPIVEDGEIIKISTSAVQLRIEKSTGKMELFNKLNNKTVWKEAEPLKHRV
DGTIQTLESNEDEYFYGGGMQNGRFSHKGKKINIKNENNWVDGGVASPNPFYFSTNGYGVMRHTFKPGEYDFEVSASGKVTTKHEENRFDAYYFIDEKPTNIIDKFTELTGEPVLLPEYGFYLGHANCYSRDWINDETGQESQTQKPGFDRQESLMVDAKKVVDD
HVSNDTPLGWFLPNDGYGCGYGREESIDGNIANLKEFVDYARGFGIQTGLWTQSSLKPTGNQEAYLERDIDKEVGVAGTNGVKTDVAWVGAGYSFALNSVRQAAEGIINNSKDKARPFIVSLDGWAGTQRYASIWSGDQYGGEWEYIRFHIPTYIGAGLSGQPN
VGSDMDGIFGGSKLVQTRDFQWKAFTPVQIDMDGWGANAKYPYVFGEPYTSINRMYLKLKAEMMPYNYSIANEATNNGVPMIRAMMLEYPEEYTYGTDTQYQYMWGPNMLVAPIYQNTDGDSEGNDIRNNIYLPDEEQIWIDYFTGKQYRGGGVLNNFEA
PLWKLPIFVKNGAIIPMTSENNNPEERDDSHRIYEVYPSGDTSFEVYEDDGLTTDYKEGKSAKTMVTSSAPKTGKGTAVINVGLLEGDYNGIVLDRSTEFIVNVSEKPSNLGVTLGGNDVQLTEAQSLEEFEKGSNMYFYDETPNLNKYATEGSEFAKVEITSTPKLRVK
VDKTNVKENEVKLTIDGFNNTQDIDKNEVNESLGVPGNFRASEEDITPESIKLTWDEVEGATTYDVEIDGTIFKNIKNTEYLDTGLNYDTEYSYRVRSVNKDGHSNWSELIKVKTDLDPYRNVPKDMKTEWKWGQYSSDEPSKAVDGDDSSQFHSQDSAIDKPFIID
MQKAYTIEKLELLFRKNGNGSVKRAEIYSSLDGVTYEKVFSNAEGSDIAPWATDGEVKTINFNKPIKVRYFKIVTKESIGNFLAMREFRPYKVDGTNGQIVGDWNNSGSIEEGDLVFLENYAGLTTADSDWGYVSMADLNNNGLIDAYDISYVSSKLEGGVKPSEGLEL
QGDMMLVPSKSEIKSGETFTIDLVGTGLSDINAFSAEIPLDSTKYEYIKTEGTVSTSGMKNLSKARVHTDNTQHVYVNFTNIGDNVKVNGTDTIARITLKAKQNITWDMEISNALLVDSKLNSKSAIAKIIDLESELPSGRPNSSKVSKENITVSGDSSQLQSGMGLDKLI
DGTTSSDDSSRMDLKWIFTSDQQDKGTLPFEMTFEFNEPKTLENFTIYNRMNSNGTINIAAMKKVKAVGYLNGEEFDLGEKANITSATTVYELGGKEFDKIVITALDSHKDKNTLAINEIEFYEKSEVETTGISFAENTPESVYLNRITPIFAEVTPDNANNLNYRLASEN
PDILQILRVDREDKATYYLRGLNPGVAKLVATTAEGNHKVEKEIVVLDGIDKTLLTKAIEEAKSYESLSEIYTIESYEALLEAIKHAEDVLENSSSEKEIGDAIINLRSKISKLEERETVEEDKIDSSKLEAIYATSEADRDYKENAVDGDENTIWHSAYQAADKLPVSITIKLDKAY
DLNQIDYLPRQNSRNGHVTEYKIETSLDNENWTEVRTGNLEVNEAGNALANRGYNPIRFNTINAQYLRFTALKTLGDTNNKYASAAELVFYGKEGKVSAESITLEKTELKLNVNESEQLKAVLNPIESNDTITWTSSDESIAKVDENGVVTGIGKGEALITATIPNGKSA
TSKVIVEDSVSEEIIVSPVRDFKASQVNKKDVTVTWTTPESTTGLEGYILYRDGKKVAQLEADETSYMFNKLNRHTIYNFKIAAKYSNGKISEKSSITIRTAR
00000000000000000000000000000000000000000000000000000000000000000000000000000000000000000000000000000000000000000000000000000000000000000000000000000000000000000000000000
00000000000000000000000000000000000000000000000000000000000000000000000000000000000000000000000000000000000000000000000000000000000000000000000000000000000000000000000000
00000000000000000000000000000000000000000000000000000000000000000000000000000000000000000000000000000000000000000000000000000000000000000000000000000000000000000000000000
00000000000000000000000000000000000000000000000000000000000000000000000000000000000000000000000000000000000000000000000000000000000000000000000000000000000000000000000000
00000000000000000000000000000000000000000000000000000000000000000000000000000000000000000000000000000000000000000000000000000000000000000000000000000000000000000000000000
00000000000000000000000000000000000000000000000000000000000000000000000000000000000000000000000000000000000000000000000000000000000000000000000000000000000000000000000000
00000000000000000000000000000000000000000000000000000000000000000000000000000000000000000000000000000000000000000000000000000000000000000000000000000000000000000000000000
00000000000000000000000000000000000000000000000000000000000000000000000000000000000000000000000000000000000000000000000000000000000000000000000000000000000000000000000000
00000000000000000000000000000000000000000000000000000000000000000000000000000000000000000000000000000000000000000000000000000000000000000000000000000000000000000000000000
00000000000000000000000000000000000000000000000000000000000000000000000000000000000000000000000000000000000000000000000000000000000000000000000000000000000000000000000000
00000000000000000000000000000000000000000000000000000000000000000000000000000000000000000000000000000000000000000000000000000000000000000000000000000000000000000000000000
00000000000000000000000000000
00000000000000000000000000000000000000000000000000000000000000000000000000000000000000000000000000000000000000000000000000000000000000000000000000000000000000000000000000
00000000000000000000000000000000000000000000000000000000000000000000000000000000000000000000000000000000000000000000000000000000000000000000000000000000000000000000000000
00000000000000000000000000000000000000000000000000000000000000000000000000000000000000000000000000000000000000000000000000000000000000000000000000000000000000000000000000
00000000000000000000000000000000000000000000000000000000000000000000000000000000000000000000000000000000000000000000000000000000000000000000000000000000000000000000000000
00000000000000000000000000000000000000000000000000000000000000000000000000000000000000000000000000000000000000000000000000000000000000000000000000000000000000000000000000
00000000000000000000000000000000000000000000000000000000000000000000000000000000000000000000000000000000000000000000000000000000000000000000000000000000000000000000000000
00000000000000000000000000000000000000000000000000000000000000000000000000000000000000000000000000000000000000000000000000000000000000000000000000000000000000000000000000
00000000000000000000000000000000000000000000000000000000000000000000000000000000000000000000000000000000000000000000000000000000000000000000000000000000000000000000000000
00000000000000000000000000000000000000000000000000000000000000000000000000000000000000000000000000000000000000000000000000000000000000000000000000000000000000000000000000
00000000000000000000000000000000000000000000000000000000000000000000000000000000000000000000000000000000000000000000000000000000000000000000000000000000000000000000000000
00000000000000000000000000000000000000000000000000000000000000000000000000000000000000000000000000000000000000000000000000000000000000000000000000000000000000000000000000
00000000000000000000000000000
00000000000000000000000000000000000000000000000000000000000000000000000000000000000000000000000000000000000000000000000000000000000000000000000000000000000000000000000000
00000000000000000000000000000000000000000000000000000000000000000000000000000000000000000000000000000000000000000000000000000000000000000000000000000000000000000000000000
00000000000000000000000000000000000000000000000000000000000000000000000000000000000000000000000000000000000000000000000000000000000000000000000000000000000000000000000000
00000000000000000000000000000000000000000000000000000000000000000000000000000000000000000000000000000000000000000000000000000000000000000000000000000000000000000000000000
00000000000000000000000000000000000000000000000000000000000000000000000000000000000000000000000000000000000000000000000000000000000000000000000000000000000000000000000000
00000000000000000000000000000000000000000000000000000000000000000000000000000000000000000000000000000000000000000000000000000000000000000000000000000000000000000000000000
00000000000000000000000000000000000000000000000000000000000000000000000000000000000000000000000000000000000000000000000000000000000000000000000000000000000000000000000000
00000000000000000000000000000000000000000000000000000000000000000000000000000000000000000000000000000000000000000000000000000000000000000000000000000000000000000000000000
00000000000000000000000000000000000000000000000000000000000000000000000000000000000000000000000000000000000000000000000000000000000000000000000000000000000000000000000000
00000000000000000000000000000000000000000000000000000000000000000000000000000000000000000000000000000000000000000000000000000000000000000000000000000000000000000000000000
00000000000000000000000000000000000000000000000000000000000000000000000000000000000000000000000000000000000000000000000000000000000000000000000000000000000000000000000000
00000000000000000000000000000
>Q5J1Q6
MTFRVRDLFADDAGEATAQLAAWNDTARDFPTEVGVHELVRRQAAEHGARGAVTFEGATTTYAELDAAAAGLARRLAGAGVRPGDVVAIALPPSERRIAAVLGALRAGAAYLPLDPELPAPRLEFLLADAGAAVLITDTATREGPVAAHDGPTVLCDRLSPDGAP
TAEPTAEPAEFPGSALAAYVIYTSGSTGRPKGVAVDHRSLVNRLLWMRERYGVGQGDVVLHKTSFGFDVSVWEQLLPLISGARLVIAPQETRRDATALVRLVRAESVTVLHFVPSLLPAFLDVAEVEQARALRVAISSGEALPKPVAVDFARRLPHCALHNLYGPTEA
TIDVTHWPVDGDDERPFVPIGTPIDNTSVYVLDDHLEPLPVGAEGELCIGGQAVALGYLGRPALTAEKFAPDPFGAPGARMYRTGDRARFGPDGAIEYLGRRDGQVKLRGYRVELGETEAALLESDLVRQAAVVVRGEGGAKRLVGFVSGRPDAAGGAEGAEAHV
LARLSATLPAYQVPSQLVWLDALPLTPSGKVDRAALPETAAAHRSSAAPETPTERAIAALWAELLDGPEPGLDDDFFRTGGDSVSSMRLAARAQRQGVRLDVQHIFTHRTVRGMAAAADEASTRPVVAEPRGDDEEALLAGVDPEHLAQVRARPGVVDVYPLAP
MQEGMLFRGLFWPDSDAYFNQNVLELVGGTDEDVLREAWRRVADRYEILRTGFTWRDRAEPLQYVRDDLAPPWALLDWSGEDPDRRAELLERLLADDRAAGLSLADCPLYRLTLVRLGGGRHYLLWSHHHILLDGWCLSLIWGDVFRVYEELAHGREPRLEPV
RPYRDYVRWIRARAARSEESERFWREYLRDLTPSPFSTAPADREGEFDTVVHEFPEQLTRLVNEAARGYGVTTNAISQAAWSMLLALQTGARDVVHGLTIAGRPPELEGSEHMVGIFINTIPLRIRLDPDQTVTGLLETIHRDLATTAAHGHVPLAQIKSWASGERLS
GGRVFDNLVAFENYPEDNLPGDDLSIQIIDLHAQEKTEYPIGLIVLPLQKLAFHFNYDTTHFTRAQVDRFIEVFRLLTEQLCRRPSARLSELELLPAADRELLGSWNGATRGTTGDGQEPSLLDLFLRHRAATPDAPAVVENGVVLDYAGLDERAIALADRLAEAGVRAG
HLVGVCLRRSAAHPIALLAVHRSGAVPVLLDPEHPVARLREAAADAGVAAVVTTADQAGFEAWPVVHPDGGIVAPTEPAACRPGSADPARDACVVYTSGSTGGPKGVLAPQAGLVNRVLWSGRAFPTPEPPRVLATAGTAFDIALWELFFPLAHGGAVVTAPQ
DAVLDPDALAELIVAERVTVAHFLPSLLAAFLDSPKAAGCTGLAHVLSGGEAVTPAIVRAFTGSGLPARLHQAYGPAEASISVTHHTCAPEDGLRERVPIGRPIDGAGAHVVDEHLRELPVGCVGELAVTGVAVGAGYPNDPELTARRFPANPFGAQPLYLTGDLAR
WGADGELEFLGRRDRQVKVRGHRVEPAEVDRTAELHPGVRQAVTVFRDGALTTFAAPRAGALLDVADLRRLLRERLPDWMVGELVVLDALPVTGNGKVDQRALLDLAGAGTRSAVVGSRPPATELEKLVARCWEEVLGVTGIGAHDDFFEIGGHSLLVVRLLRV
LRDNLPEGVHCDVPHILKAPTVAGLAEQLLAPVEGSGSAYVHTLNPEATGGALVLVHPGEGLALPYHGLAPLLPDVRLHVLSDPRFGQSDNRFATLAEMATRYVEWVRTTEPEGPYRLGGWSFGGVVALEMASQMTAHGDEVSDLLLVDSHNLNAAPRTGDPRE
GVRQRLVELGVDPDSPEGVDVVEELLHNGALAAQYAPPAYRGRVSLLVTPTDGDRDAVRARGWDRALLPDLVVEPVPGAHERLFDEEHLSDTADAIRRALGGER
00000000000000000000000000000000000000000000000000000000000000000000000000000000000000000000000000000000000000000000000000000000000000000000000000000000000000000000000000
00000000000000000000000000000000000000000000000000000000000000000000000000000000000000000000000000000000000000000000000000000000000000000000000000000000000000000000000000
00000000000000000000000000000000000000000000000000000000000000000000000000000000000000000000000000000000000000000000000000000000000000000000000000000000000000000000000000
00000000000000000000000000000000000000000000000000000000000000000000000000000000000000000000000000000000000000000000000000000000000000000000000000000000000000000000000000
00000000000000000000000000000000000000000000000000000000000000000000000000000000000000000000000000000000000000000000000000000000000000000000000000000000000000000000000000
00000000000000000000000000000000000000000000000000000000000000000000000000000000000000000000000000000000000000000000000000000000000000000000000000000000000000000000000000
00000000000000000000000000000000000000000000000000000000000000000000000000000000000000000000000000000000000000000000000000000000000000000000000000000000000000000000000000
00000000000000000000000000000000000000000000000000000000000000000000000000000000000000000000000000000000000000000000000000000000000000000000000000000000000000000000000000
00000000000000000000000000000000000000000000000000000000000000000000000000000000000000000000000000000000000000000000000000000000000000000000000000000000000000000000000000
00000000000000000000000000000000000000000000000000000000000000000000000000000000000000000000000000000000000000000000000000000000000000000000000000000000000000000000000000
00000000000000000000000000000000000000000000000000000000000000000000000000000000000000000000000000000000000000000000000000000000000000000000000000000000000000000000000000
00000000000000000000000000000000000000000000000000000000000000000000000000000000000000000000000000000000000000000000000000000000000000000000000000000000000000000000000
00000000000000000000000000000000000000000000000000000000000000000000000000000000000000000000000000000000000000000000000000000000000000000000000000000000000000000000000000
00000000000000000000000000000000000000000000000000000000000000000000000000000000000000000000000000000000000000000000000000000000000000000000000000000000000000000000000000
00000000000000000000000000000000000000000000000000000000000000000000000000000000000000000000000000000000000000000000000000000000000000000000000000000000000000000000000000
00000000000000000000000000000000000000000000000000000000000000000000000000000000000000000000000000000000000000000000000000000000000000000000000000000000000000000000000000
00000000000000000000000000000000000000000000000000000000000000000000000000000000000000000000000000000000000000000000000000000000000000000000000000000000000000000000000000
00000000000000000000000000000000000000000000000000000000000000000000000000000000000000000000000000000000000000000000000000000000000000000000000000000000000000000000000000
00000000000000000000000000000000000000000000000000000000000000000000000000000000000000000000000000000000000000000000000000000000000000000000000000000000000000000000000000
00000000000000000000000000000000000000000000000000000000000000000000000000000000000000000000000000000000000000000000000000000000000000000000000000000000000000000000000000
00000000000000000000000000000000000000000000000000000000000000000000000000000000000000000000000000000000000000000000000000000000000000000000000000000000000000000000000000
00000000000000000000000000000000000000000000000000000000000000000000000000000000000000000000000000000000000000000000000000000000000000000000000000000000000000000000000000
00000000000000000000000000000000000000000000000000000000000000000000000000000000000000000000000000000000000000000000000000000000000000000000000000000000000000000000000000
00000000000000000000000000000000000000000000000000000000000000000000000000000000000000000000000000000000000000000000000000000000000000000000000000000000000000000000000
00000000000000000000000000000000000000000000000000000000000000000000000000000000000000000000000000000000000000000000000000000000000000000000000000000000000000000000000000
00000000000000000000000000000000000000000000000000000000000000000000000000000000000000000000000000000000000000000000000000000000000000000000000000000000000000000000000000
00000000000000000000000000000000000000000000000000000000000000000000000000000000000000000000000000000000000000000000000000000000000000000000000000000000000000000000000000
00000000000000000000000000000000000000000000000000000000000000000000000000000000000000000000000000000000000000000000000000000000000000000000000000000000000000000000000000
00000000000000000000000000000000000000000000000000000000000000000000000000000000000000000000000000000000000000000000000000000000000000000000000000000000000000000000000000
00000000000000000000000000000000000000000000000000000000000000000000000000000000000000000000000000000000000000000000000000000000000000000000000000000000000000000000000000
00000000000000000000000000000000000000000000000000000000000000000000000000000000000000000000000000000000000000000000000000000000000000000000000000000000000000000000000000
00000000000000000000000000000000000000000000000000000000000000000000000000000000000000000000000000000000000000000000000000000000000000000000000000000000000000000000000000
00000000000000000000000000000000000000000000000000000000000000000000000000000000000000000000000000000000000000000000000000000000000000000000000000000000000000000000000000
>Q84HI8
MVDDDVVIVGLACRYPAADNPTQLWENVLNRRQAFRPLPAGRLPLAEYGGDGPDRTTVRRAAVLDGWRFDRARFRIPGSTFRAVDLSHWLALETAADALVDAGLPDGTGTDRSRVGVVIGNSLTGEFSRAAGLRIRWPYVRRAVAAGLVDGGLATDEVAALLD
RVEERFKAPFPVPGDETLAGALANTIAGRICNQFDFHGTGYTVDGACASSLLAVITAAEAVAAGQLDVALAGGVDLSLDPLELVGFSRVGALAGDEMRVYDAHPTGFLPGEGCGVVVLARAGYARERGLRPYGRLLGWASSSDGSGGLTRPEESGQRLALRRAYD
RAGIEPGAVRLVEGHGTGTAVGDEAELRALLGVRGPAASPAVLGSIKANIGHTKAAAGVAGLIKAVLAVHREVLPPTTGCHRPHPLLAEGGALRVTDEARPWPDGERYASVSAMGFGGINTHVVLGGTAPSASRPLSAHVRRLSRPHPDHELVVCGAPDAAALTG
LLTRVRAAATALSRPELTDLAAGLAAAHRGDVPARFAAAVRDADGLVAALDRALGHLAEGGRRLLDAGRGLFLVVGGPLRVGLLFPGQAAPVHADRGALGHLLGDADAGTGSDPDSGVKPAEPVDTAVAQPAIIADSLAGIRWLDRLGARPVGALGHSLGELAAL
SWAGALDADDTLALARARGEAMSAATEAPSGMLSLRADLAAARELAAGTGAVVAVDNGERHVVVAGTRPELDRVAEAARHAGIEATPLAVSHAFHSPLMAPAAEALRRAAGRLPWRRPERPVASTVTGAWWADEDPVEVLVRQLTGPVRFREALGLLDADLL
VEVGPGRMLSALAEAAGRTAVSLDAGAASAAGMAAGTAALFAAGAVDDATPFFAGRFTRPFDRDREREFLVNPCEREVPAVALPAPRAATPDPRPRASAGPVAARPADGRGTAELVAARVADLAELPAAEVGPGTRLLADLHLNSIRVGQLAAEIAGELGRALP
ATPLSLADATVGEFAAAIEGLPTADAPAAELPAAGVGPWVRVFRHHLVPRPAGAAAPAGEPPAWEVVGPLAGHPHAEAVRTAFRVDAGAPAGRLLALPPGLADIPTADVVAALRAAAGDRRPLVVLHHGGVGAAVGRSLAVETGAPVLVVETPPGPAGVRLAA
VEASRPWSGYQEVVYGADGVRTVPVTRLLDGQPRTDRRIPLGPGEVCLVTGGAKGIGAACAAALGEATGARLVLFGRSPADDPEVVEAVRRTGAVYRSVDLADPVAVAAAVTAVRAEHGPVRGLLHAAGRNEPAPVTDLTAEELHRTLAPKAYGFEHVVAALDP
DELRFAVAFGSVIGRAGLPGEAAYATANEYLSRRCAELAATVPQVRWLSIEWSVWSGTGMGVRLGVLDGLLRRGVSPIPPDEGTRLLLELLATPALPPVVLVAGRLPVSDTLEWDECAEEHGRFLESRLVGTPCVELVSEARLSLGADPYLDDHRIDGAAVLPAVLGL
EAMAQAAAALGAKQVPTVIRDVALTRPVTVGDREPRPVRVAALTGADGTVRAAVRSAETGFAVDHLTGTYGGAVTSGEPVPPPSTGLLVPGGPLYGPAFFHGPRFRRVAGYHGLSAYRCVAEVHAEAGTWFGGFHDQRLQLGDPGVRDAFLHLLQACVPDRR
VLPVGVDRIDIHARPEGTLTVHARQVAEAGDEFRFDVTVIDADGSPVERWTGLRLRAVGPVENAVWPAELVGPLVTRRLRRWRPELRVDLAAAPAGRGDRERSRALAGWLLGGEVRADATGRLRPGVPATPLGWRGISVSHLDGHLLTAAADRPVGVDWEVV
TPTPPLPEAAARVAEALARLTGEDAATAGTRVWTALEALVKAGAAVDVPLVLRDETGPDGWVALHAGRHAVYGTVVPATPGPIAVSLAVAVDADGEG
00000000000000000000000000000000000000000000000000000000000000000000000000000000000000000000000000000000000000000000000000000000000000000000000000000000000000000000000000
00000000000000000000000000000000000000000000000000000000000000000000000000000000000000000000000000000000000000000000000000000000000000000000000000000000000000000000000000
00000000000000000000000000000000000000000000000000000000000000000000000000000000000000000000000000000000000000000000000000000000000000000000000000000000000000000000000000
00000000000000000000000000000000000000000000000000000000000000000000000000000000000000000000000000000000000000000000000000000000000000000000000000000000000000000000000000
00000000000000000000000000000000000000000000000000000000000000000000000000000000000000000000000000000000000000000000000000000000000000000000000000000000000000000000000000

00
00
00
00
00
00
000000000000000000000000000000000000000000000000000000000000000000000000000000000000000000000000000000000000000000000000000000000000000000000000000000000000000000000000000000000
00
00
00
00
00
00
00
00
00
00
00
00
000000000000000000000000000000000000000000000000000000000000000000000000000000000000000000000000000000000000000000000000000000000000000000000000000000000000000000000000000000000
00
00
00
00
00
00
00
00
00
00
00
00
000000000000000000000000000000000000000000000000000000000000000000000000000000000000000000000000000000000000000000000000000000000000000000000000000000000000000000000000000000000
>Q8IY92
MKLSVNEAQLGFYLGSLSHLSACPGIDPRSSEDQPESLKTGQMMDESDEDFKELCASFFQRVKKHGIKEVSGERKTQKAASNGTQIRSKLKRTKQTATKTKTLQGPAEKKPPSGSQAPRTKKQRVTKWQASEPAHSVNGEGGVLASAPDPPVLRETAQNTQTGNQ
QEPSPNLSREKTRENVPNSDSQPPPSCLTTAVPSPSKPRTAQLVLQRMQQFKRADPERLRHASEECSLEAAREENVPKDPQEEMMAGNVYGLGPPAPESDAAVALTLQQEFARVGASAHDDSLEEKGLFFCQICQKNLSAMNVTRREQHVNRCLDEAEKTLRPS
VPQIPECPICGKPFLTLKSRTSHLKQCAVKMEVGPQLLLQAVRLQTAQPEGSSSPPMFSFSDHSRGLKRRGPTSKKEPRKRRKVDEAPSEDLLVAMALSRSEMEPGAAVPALRLESAFSERIRPEAENKSRKKKPPVSPPLLLVQDSETTGRQIEDRVALLLSEEVELSST
PPLPASRILKEGWERAGQCPPPPERKQSFLWEGSALTGAWAMEDFYTARLVPPLVPQRPAQGLMQEPVPPLVPPEHSELSERRSPALHGTPTAGCGSRGPSPSASQREHQALQDLVDLAREGLSASPWPGSGGLAGSEGTAGLDVVPGGLPLTGFVVPSQDKHP
DRGGRTLLSLGLLVADFGAMVNNPHLSDVQFQTDSGEVLYAHKFVLYARCPLLIQYVNNEGFSAVEDGVLTQRVLLGDVSTEAARTFLHYLYTADTGLPPGLSSELSSLAHRFGVSELVHLCEQVPIATDSEGKPWEEKEAENCESRAENFQELLRSMWADEEEEAE
TLLKSKDHEEDQENVNEAEMEEIYEFAATQRKLLQEERAAGAGEDADWLEGGSPVSGQLLAGVQVQKQWDKVEEMEPLEPGRDEAATTWEKMGQCALPPPQGQHSGARGAEAPEQEAPEEALGHSSCSSPSRDCQAERKEGSLPHSDDAGDYEQLFSSTQG
EISEPSQITSEPEEQSGAVRERGLEVSHRLAPWQASPPHPCRFLLGPPQGGSPRGSHHTSGSSLSTPRSRGGTSQVGSPTLLSPAVPSKQKRDRSILTLSKEPGHQKGKERRSVLECRNKGVLMFPEKSPSIDLTQSNPDHSSSRSQKSSSKLNEEDEVILLLDSDEELELE
QTKMKSISSDPLEEKKALEISPRSCELFSIIDVDADQEPSQSPPRSEAVLQQEDEGALPENRGSLGRRGAPWLFCDRESSPSEASTTDTSWLVPATPLASRSRDCSSQTQISSLRSGLAVQAVTQHTPRASVGNREGNEVAQKFSVIRPQTPPPQTPSSCLTPVSPGTSD
GRRQGHRSPSRPHPGGHPHSSPLAPHPISGDRAHFSRRFLKHSPPGPSFLNQTPAGEVVEVGDSDDEQEVASHQANRSPPLDSDPPIPIDDCCWHMEPLSPIPIDHWNLERTGPLSTSSPSRRMNEAADSRDCRSPGLLDTTPIRGSCTTQRKLQEKSSGAGSLGN
SRPSFLNSALWDVWDGEEQRPPETPPPAQMPSAGGAQKPEGLETPKGANRKKNLPPKVPITPMPQYSIMETPVLKKELDRFGVRPLPKRQMVLKLKEIFQYTHQTLDSDSEDESQSSQPLLQAPHCQTLASQTYKPSRAGVHAQQEATTGPGAHRPKGPAKTKG
PRHQRKHHESITPPSRSPTKEAPPGLNDDAQIPASQESVATSVDGSDSSLSSQSSSSCEFGAAFESAGEEEGEGEVSASQAAVQAADTDEALRCYIRSKPALYQKVLLYQPFELRELQAELRQNGLRVSSRRLLDFLDTHCITFTTAATRREKLQGRRRQPRGKKKVERN
00
00
00
00
00
00
00
00
00
00
00
00
0000000000000000000000000000000000000000000000000000000000000000000000000000000000000000000000
00
00
00
00
00
00
00
00
00
00
00
00
0000000000000000000000000000000000000000000000000000000000000000000000000000000000000000000000
00
00
00
00
00
00
00
00
00
00
00
00
0000000000000000000000000000000000000000000000000000000000000000000000000000000000000000000000
>I6X8D2
MADVAESQENAPAERAELTVPEMRQWLRNWVGKAVGKAPDSIDESVPMVELGLSSRDAVAMAADIEDLTGVTLSVAVAFAHPTIESLATRIIEGEPETDLAGDDAEDWSRTGPAERVDIAIVGLSTRFPGEMNTPEQTWQALLEGRDGITDLPDGRWSEFLEEP
RLAARVAGARTRGGYLKDIKGFDSEFFAVAKTEADNIDPQQRMALELTWEALEHARIPASSLRGQAVGVYIGSSTNDYSFLAVSDPTVAHPYAITGTSSSIIANRVSYFYDFHGPSVTIDTACSSSLVAIHQGVQALRNGEADVVVAGGVNALITPMVTLGFDEIGAVL
APDGRIKSFSADADGYTRSEGGGMLVLKRVDDARRDGDAILAVIAGSAVNHDGRSNGLIAPNQDAQADVLRRAYKDAGIDPRTVDYIEAHGTGTILGDPIEAEALGRVVGRGRPADRPALLGAVKTNVGHLESAAGAASMAKVVLALQHDKLPPSINFAGPSPYID
FDAMRLKMITTPTDWPRYGGYALAGVSSFGFGGANAHVVVREVLPRDVVEKEPEPEPEPKAAAEPAEAPTLAGHALRFDEFGNIITDSAVAEEPEPELPGVTEEALRLKEAALEELAAQEVTAPLVPLAVSAFLTSRKKAAAAELADWMQSPEGQASSLESIGRSLSR
RNHGRSRAVVLAHDHDEAIKGLRAVAAGKQAPNVFSVDGPVTTGPVWVLAGFGAQHRKMGKSLYLRNEVFAAWIEKVDALVQDELGYSVLELILDDAQDYGIETTQVTIFAIQIALGELLRHHGAKPAAVIGQSLGEAASAYFAGGLSLRDATRAICSRSHLMGEG
EAMLFGEYIRLMALVEYSADEIREVFSDFPDLEVCVYAAPTQTVIGGPPEQVDAILARAEAEGKFARKFATKGASHTSQMDPLLGELTAELQGIKPTSPTCGIFSTVHEGRYIKPGGEPIHDVEYWKKGLRHSVYFTHGIRNAVDSGHTTFLELAPNPVALMQVALTTA
DAGLHDAQLIPTLARKQDEVSSMVSTMAQLYVYGHDLDIRTLFSRASGPQDYANIPPTRFKRKEHWLPAHFSGDGSTYMPGTHVALPDGRHVWEYAPRDGNVDLAALVRAAAAHVLPDAQLTAAEQRAVPGDGARLVTTMTRHPGGASVQVHARIDESFTLV
YDALVSRAGSESVLPTAVGAATAIAVADGAPVAPETPAEDADAETLSDSLTTRYMPSGMTRWSPDSGETIAERLGLIVGSAMGYEPEDLPWEVPLIELGLDSLMAVRIKNRVEYDFDLPPIQLTAVRDANLYNVEKLIEYAVEHRDEVQQLHEHQKTQTAEEIARAQ
AELLHGKVGKTEPVDSEAGVALPSPQNGEQPNPTGPALNVDVPPRDAAERVTFATWAIVTGKSPGGIFNELPRLDDEAAAKIAQRLSERAEGPITAEDVLTSSNIEALADKVRTYLEAGQIDGFVRTLRARPEAGGKVPVFVFHPAGGSTVVYEPLLGRLPADTPMY
GFERVEGSIEERAQQYVPKLIEMQGDGPYVLVGWSLGGVLAYACAIGLRRLGKDVRFVGLIDAVRAGEEIPQTKEEIRKRWDRYAAFAEKTFNVTIPAIPYEQLEELDDEGQVRFVLDAVSQSGVQIPAGIIEHQRTSYLDNRAIDTAQIQPYDGHVTLYMADRYHDD
AIMFEPRYAVRQPDGGWGEYVSDLEVVPIGGEHIQAIDEPIIAKVGEHMSRALGQIEADRTSEVGKQ
00
00
00
00
00
00
00
00
00
00
00
0000000000000000000000000000000000000000000000000000000000000000000000000000000000000000000000000000000000000000000000000000000000000000000000000000000000000000000000000000000000000000000000
00
00
00
00
00
00
00
00
00
00
00
0000000000000000000000000000000000000000000000000000000000000000000000000000000000000000000000000000000000000000000000000000000000000000000000000000000000000000000000000000000000000000000000
00
00
00
00
00
00
00
00
00
00
00
0000000000000000000000000000000000000000000000000000000000000000000000000000000000000000000000000000000000000000000000000000000000000000000000000000000000000000000000000000000000000000000000
>Q89444
MEAHQFIKAPGITTAIEQAALAAANSALANAVVVRPFLSHQQIEILINLMQPRQLVFRPEVFWNHPIQRVIHNELELYCRARSGRCLEIGAHPRSINDNPNVVHRCFLRPAGRDVQRWYTAPTRGPAANCRRSALRGLPAADRTYCFDGFSGCNFPAETGIALYSLH
DMSPSDVAEAMFRHGMTRLYAALHLPPEVLLPPGTYRTASYLLIHDGRRVVVTYEGDTSAGYNHDVSNLRSWIRTTKVTGDHPLVIERVRAIGCHFVLLLTAAPEPSPMPYVPYPRSTEVYVRSIFGPGGTPSLFPTSCSTKSTFHAVPAHIWDRLMLFGATLDDQAF
CCSRLMTYLRGISYKVTVGTLVANEGWNASEDALTAVITAAYLTICHQRYLRTQAISKGMRRLEREHAQKFITRLYSWLFEKSGRDYIPGRQLEFYAQCRRWLSAGFHLDPRVLVFDESAPCHCRTAIRKAVSKFCCFMKWLGQECTCFLQPAEGVVGDQGHDNEA
YEGSDVDPAESAISDISGSYVVPGTALQPLYQALDLPAEIVARAGRLTATVKVSQVDGRIDCETLLGNKTFRTSFVDGAVLETNGPERHNLSFDASQSTMAAGPFSLTYAASAAGLEVRYVAAGLDHRAVFAPGVSPRSAPGEVTAFCSALYRFNREAQRLSLTGNFW
FHPEGLLGPFAPFSPGHVWESANPFCGESTLYTRTWSEVDAVSSPAQPDLGFTSEPSIPSRAATLTPAAPLPPPAPDPSPTPSAPARGEPAPGATAGAPAITHQTARHRRLLFTYPDGSKVFAGSLFESTCTWLVNASNVDHRPGGELCHAFYQRYPASFDAASFVM
RDGAAAYTLTPRPIIHAVAPDYRLEHNPKRLEAAYRETCSRLGTAAYPLLGTGIYQVPIGPSFDAWERNHRPGDELYLPELAARWFEANRPTCPTLTITEDVARTANLAIELDSATDVGRACAGCRVTPGVVQYQFTAGVPGSGKSRSITQADVDVVVVPTRELRNA
WRRRGFAAFTPHTAARVTQGRRVVIDEAPSLPPHLLLLHMQRAATVHLLGDPNQIPAIDFEHAGLVPAIRPDLAPTSWWHVTHRCPADVCELIRGAYPMIQTTSRVLRSLFWGEPAVGQKLVFTQAAKAANPGSVTVHEAQGATYTETTIIATADARGLIQSSRAH
AIVALTRHTEKCVIIDAPGLLREVGISDAIVNNFFLAGGEIGHQRPSVIPRGNPDANVDTLAAFPPSCQISAFHQLAEELGHRPAPVAAVLPPCPELEQGLLYLPQELTTCDSVVTFELTDIVHCRMAAPSQRKAVLSTLVGRYGRRTKLYNASHSDVRDSLARFIPAIGP
VQVTTCELYELVEAMVEKGQDGSAVLELDLCNRDVSRITFFQKDCNKFTTGETIAHGKVGQGISAWSKTFCALFGPWFRAIEKAILALLPQGVFYGDAFDDTVFSAAVAAAKASMVFENDFSEFDSTQNNFSLGLECAIMEECGMPQWLIRLYHLIRSAWILQAPKE
SLRGFWKKHSGEPGTLLWNTVWNMAVITHCYDFRDLQVAAFKGDDSIVLCSEYRQSPGAAVLIAGCGLKLKVDFRPIGLYAGVVVAPGLGALPDVVRFAGRLTEKNWGPGPERAEQLRLAVSDFLRKLTNVAQMCVDVVSRVYGVSPGLVHNLIGMLQAVADG
KAHFTESVKPVLDLTNSILSRVE

000
000
000
000
000
000
000
000
000
000
000000000000000000000000000000000000000000000000000000000000000000000000000000000000000000000000000000000000000000000000000000
000
000
000
000
000
000
000
000
000
000
000000000000000000000000000000000000000000000000000000000000000000000000000000000000000000000000000000000000000000000000000000
000
000
000
000
000
000
000
000
000
000
000000000000000000000000000000000000000000000000000000000000000000000000000000000000000000000000000000000000000000000000000000
>A0A0H2YN38
MNKRKIAAIILATMITNLSATTIDVLAQELNTKNNSKVEVSHDDESHQARVSKFDLYNSDKLDAYNQEFQVSRSNIKSINNNGGKYNSSTIDKAIDGNLETHWETGKPNDANFTNEVVVTFNEITNIDRIVYSARRDSARGKGFAKEFEIYASLKDEGDDFNLVSSGEYT
ESTRDLVEIKFNPTDFKRLKFKFKKADQNWASAAEFMFYKEDKLNEKFNGLFTDSSMNKVSEEFNTLEKLNAFENELKDHPIYDLYKEGLNNARAILTETSENPTKATLGQITYNLNDDYNNQYRMPYKNIKAIKNNGRHYAAQNIEKAIDNDVNTYWETGTLNSSSF
NNEVEVEFNDLVTLDRIVYGSRQSDLKGFAEEVYIYASRTSKGDTYKLVATGAHEATKGLVEAKFEPTEFKRVKFKFKKSKQNSATLNELMFYKPDEVYSSIPKLFTDGTMSELSEEFNSLEKINAFKEKAKNHPLYNDFNETIELAESLISNPRKEDVLELEMRGDSISEAK
KRKVWNFQDWQITGLSARAGDKITVYVDVAEGDPTPTLLYKQSLTQHGGATSFQLKPGKNEITIPEINYESNGIPKDVIQGGDLFFTNYKSDSQKRAPKVRIEGASKYPVFILGKSDENEVMKELEAYVEKIKAEPKTTPNIFAVSSNKSLEFVQATYALDWYKKNNKT
PKYTAEQWDQYIADAMGFWGFDNSKDVNSDFNFRIMPMVKNLSGGAFMNAGNGVIGIRPGNQDAILAANKGWGVAHELGHNFDTGGRTIVEVTNNMMPLFFESKYKTKTRITDQNIWENNTYPKVGLDDYSNNELYNKADSTHLAQLAPLWQLYLYDNTF
YGKFERQFRERDFGNKNREDIYKSWVVAASDAMELDLTEFFARHGIRVDDKVKEDLAKYPKPDKKIYYLNDLAMNYKGDGFTENAKVSVSTSGSNGNIKLSFSVDDENKDNILGYEIRRDGKYVGFTSNDSFVDTKSNLDEDGVYVVTPYDRKLNTLNPIEVNALQP
TLSVNPVITLALGEEFNEEEYIVAKDIKGNSLSESVKVKSSNVNTSKVGEYEVLYSLEDSKGNEYTKTSKVNVVSRKEYMSDLTPKQSSNGWGTVRKDKSISGGVIGLTRDGDFVDYNKGLGLHSNAEYVYDLEGKDYDYFESYVGVDKAMSSRPASSVIFKVLVDGEEK
FNSGVMRSTTPQKYVKVDVKNAKELKLIVNDAGDGDSSDHASFGDAKLATLSSKPIIKGENLAYSMDEKVDLMKGITATDIEDGNITSKVQIKSSDFVEGKSGIFTVVYSVTDSDGLTSECSRTIAVTDKETQLSDLNWKSATIGSGSVRKDRAVSGNQIRLLNEDNSV
ETFAKGIGTHSYSEIVYNSEGYDIFDTWVGIDRHVADKKVSSVKFKVYVDGELKAETDVMRIDTPKKRLVVDVRNSKEIKLVVDVADNGNNWDHADWADAKFRNLAEYDASELNKAIEEAKKLDLNNYTEESSEALKNAISKGEEALLSKDKETINSALEELNKEMNS
LVKVDLNAVINIPDKYLLKSIQNQLNKTGDITLGDMYSLTTLTLSGVEDLTGLENAKNLETLNMDYNEVKDLRPLSKLKKLNTLNAQEQFIAAGELKPSNGKVIGDSKVYNREGKNVAKTIRVVDKNGNTILEQDAKDEFTINTKDLSSGLYGVHVLFEDEGFSGVMFYL
FNV
000
000
000
000
000
000
000
000
000
000
0000000000000000000000000000000000000000000000000000000000000000000000000000000000000000000000000000000000000000000000
000
000
000
000
000
000
000
000
000
000
0000000000000000000000000000000000000000000000000000000000000000000000000000000000000000000000000000000000000000000000
000
000
000
000
000
000
000
000
000
000
0000000000000000000000000000000000000000000000000000000000000000000000000000000000000000000000000000000000000000000000
>P29400
MKLRGVSLAAGLFLLALSLWGQPAEAAACYGCSPGSKCDCSGIKGEKGERGFPGLEGHPGLPGFPGPEGPPGPRGQKGDDGIPGPPGPKGIRGPPGLPGFPGTPGLPGMPGHDGAPGPQGIPGCNGTKGERGFPGSPGFPGLQGPPGPPGIPGMKGEPGSIIM
SSLPGPKGNPGYPGPPGIQGLPGPTGIPGPIGPPGPPGLMGPPGPPGLPGPKGNMGLNFQGPKGEKGEQGLQGPPGPPGQISEQKRPIDVEFQKGDQGLPGDRGPPGPPGIRGPPGPPGGEKGEKGEQGEPGKRGKPGKDGENGQPGIPGLPGDPGYPGEPG
RDGEKGQKGDTGPPGPPGLVIPRPGTGITIGEKGNIGLPGLPGEKGERGFPGIQGPPGLPGPPGAAVMGPPGPPGFPGERGQKGDEGPPGISIPGPPGLDGQPGAPGLPGPPGPAGPHIPPSDEICEPGPPGPPGSPGDKGLQGEQGVKGDKGDTCFNCIGTGIS
GPPGQPGLPGLPGPPGSLGFPGQKGEKGQAGATGPKGLPGIPGAPGAPGFPGSKGEPGDILTFPGMKGDKGELGSPGAPGLPGLPGTPGQDGLPGLPGPKGEPGGITFKGERGPPGNPGLPGLPGNIGPMGPPGFGPPGPVGEKGIQGVAGNPGQPGIPGPK
GDPGQTITQPGKPGLPGNPGRDGDVGLPGDPGLPGQPGLPGIPGSKGEPGIPGIGLPGPPGPKGFPGIPGPPGAPGTPGRIGLEGPPGPPGFPGPKGEPGFALPGPPGPPGLPGFKGALGPKGDRGFPGPPGPPGRTGLDGLPGPKGDVGPNGQPGPMGPPGL
PGIGVQGPPGPPGIPGPIGQPGLHGIPGEKGDPGPPGLDVPGPPGERGSPGIPGAPGPIGPPGSPGLPGKAGASGFPGTKGEMGMMGPPGPPGPLGIPGRSGVPGLKGDDGLQGQPGLPGPTGEKGSKGEPGLPGPPGPMDPNLLGSKGEKGEPGLPGIPGVS
GPKGYQGLPGDPGQPGLSGQPGLPGPPGPKGNPGLPGQPGLIGPPGLKGTIGDMGFPGPQGVEGPPGPSGVPGQPGSPGLPGQKGDKGDPGISSIGLPGLPGPKGEPGLPGYPGNPGIKGSVGDPGLPGLPGTPGAKGQPGLPGFPGTPGPPGPKGISGPPGN
PGLPGEPGPVGGGGHPGQPGPPGEKGKPGQDGIPGPAGQKGEPGQPGFGNPGPPGLPGLSGQKGDGGLPGIPGNPGLPGPKGEPGFHGFPGVQGPPGPPGSPGPALEGPKGNPGPQGPPGRPGLPGPEGPPGLPGNGGIKGEKGNPGQPGLPGLPGLKGD
QGPPGLQGNPGRPGLNGMKGDPGLPGVPGFPGMKGPSGVPGSAGPEGEPGLIGPPGPPGLPGPSGQSIIIKGDAGPPGIPGQPGLKGLPGPQGPQGLPGPTGPPGDPGRNGLPGFDGAGGRKGDPGLPGQPGTRGLDGPPGPDGLQGPPGPPGTSSVAHGF
LITRHSQTTDAPQCPQGTLQVYEGFSLLYVQGNKRAHGQDLGTAGSCLRRFSTMPFMFCNINNVCNFASRNDYSYWLSTPEPMPMSMQPLKGQSIQPFISRCAVCEAPAVVIAVHSQTIQIPHCPQGWDSLWIGYSFMMHTSAGAEGSGQALASPGSCLEEFRS
APFIECHGRGTCNYYANSYSFWLATVDVSDMFSKPQSETLKAGDLRTRISRCQVCMKRT
000
000
000
000
000
000
000
000
000
000
00000000000000000000000000000000000000000000000000000000000000000000000000000000000000000000000000000000000000000000
000
000
000
000
000
000
000
000
000
000
00000000000000000000000000000000000000000000000000000000000000000000000000000000000000000000000000000000000000000000
000
000
000
000
000
000
000
000
000
000
00000000000000000000000000000000000000000000000000000000000000000000000000000000000000000000000000000000000000000000
>Q8TCU6
MEAPSGSEPGGDGAGDCAHPDPRAPGAAAPSSGPGPCAAARESERQLRLRLCVLNEILGTERDYVGTLRFLQSAFLHRIRQNVADSVEKGLTEENVKVLFSNIEDILEVHKDFLAALEYCLHPEPQSQHELGNVFLKFKDKFCVYEEYCSNHEKALRLLVELNKIPTVR
AFLLSCMLLGGRKTTDIPLEGYLLSPIQRICKYPLLLKELAKRTPGKHPDHPAVQSALQAMKTVCSNINETKRQMEKLEALEQLQSHIEGWEGSNLTDICTQLLLQGTLLKISAGNIQERAFFLFDNLLVYCKRKSRVTGSKKSTKRTKSINGSLYIFRGRINTEVMEVENV
EDGTADYHSNGYTVTNGWKIHNTAKNKWFVCMAKTAEEKQKWLDAIIREREQRESLKLGMERDAYVMIAEKGEKLYHMMMNKKVNLIKDRRRKLSTVPKCFLGNEFVAWLLEIGEISKTEEGVNLGQALLENGIIHHVSDKHQFKNEQVMYRFRYDDGTYKAR
SELEDIMSKGVRLYCRLHSLYTPVIKDRDYHLKTYKSVLPGSKLVDWLLAQGDCQTREEAVALGVGLCNNGFMHHVLEKSEFRDESQYFRFHADEEMEGTSSKNKQLRNDFKLVENILAKRLLILPQEEDYGFDIEEKNKAVVVKSVQRGSLAEVAGLQVGRKIYSINE
DLVFLRPFSEVESILNQSFCSRRPLRLLVATKAKEIIKIPDQPDTLCFQIRGAAPPYVYAVGRGSEAMAAGLCAGQCILKVNGSNVMNDGAPEVLEHFQAFRSRREEALGLYQWIYHTHEDAQEARASQEASTEDPSGEQAQEEDQADSAFPLLSLGPRLSLCEDSPM
VTLTVDNVHLEHGVVYEYVSTAGVRCHVLEKIVEPRGCFGLTAKILEAFAANDSVFVENCRRLMALSSAIVTMPHFEFRNICDTKLESIGQRIACYQEFAAQLKSRVSPPFKQAPLEPHPLCGLDFCPTNCHINLMEVSYPKTTPSVGRSFSIRFGRKPSLIGLDPEQGHL
NPMSYTQHCITTMAAPSWKCLPAAEGDPQGQGLHDGSFGPASGTLGQEDRGLSFLLKQEDREIQDAYLQLFTKLDVALKEMKQYVTQINRLLSTITEPTSGGSCDASLAEEASSLPLVSEESEMDRSDHGGIKKVCFKVAEEDQEDSGHDTMSYRDSYSECNSNRD
SVLSYTSVRSNSSYLGSDEMGSGDELPCDMRIPSDKQDKLHGCLEHLFNQVDSINALLKGPVMSRAFEETKHFPMNHSLQEFKQKEECTIRGRSLIQISIQEDPWNLPNSIKTLVDNIQRYVEDGKNQLLLALLKCTDTELQLRRDAIFCQALVAAVCTFSKQLLAALGY
RYNNNGEYEESSRDASRKWLEQVAATGVLLHCQSLLSPATVKEERTMLEDIWVTLSELDNVTFSFKQLDENYVANTNVFYHIEGSRQALKVIFYLDSYHFSKLPSRLEGGASLRLHTALFTKVLENVEGLPSPGSQAAEDLQQDINAQSLEKVQQYYRKLRAFYLERSN
LPTDASTTAVKIDQLIRPINALDELCRLMKSFVHPKPGAAGSVGAGLIPISSELCYRLGACQMVMCGTGMQRSTLSVSLEQAAILARSHGLLPKCIMQATDIMRKQGPRVEILAKNLRVKDQMPQGAPRLYRLCQPPVDGDL
000
000
000

0000000000000000000000000000000000000000000000000000000000000000000000000000000000000000000000000000000000000000000000000000000000000000000000000000000000000000000000000000000000
0000000000000000000000000000000000000000000000000000000000000000000000000000000000000000000000000000000000000000000000000000000000000000000000000000000000000000000000000000000000
0000000000000000000000000000000000000000000000000000000000000000000000000000000000000000000000000000000000000000000000000000000000000000000000000000000000000000000000000000000000
0000000000000000000000000000000000000000000000000000000000000000000000000000000000000000000000000000000000000000000000000000000000000000000000000000000000000000000000000000000000
0000000000000000000000000000000000000000000000000000000000000000000000000000000000000000000000000000000000000000000000000000000000000000000000000000000000000000000000000000000000
0000000000000000000000000000000000000000000000000000000000000000000000000000000000000000000000000000000000000000000000000000000000000000000000000000000000000000000000000000000000
0000000000000000000000000000000000000000000000000000000000000000000000000000000000000000000000000000000000000000000000000000000000000000000000000000000000000000000000000000000000
00000000000000000000000000000000000000000000000000000000000000000000000000
0000000000000000000000000000000000000000000000000000000000000000000000000000000000000000000000000000000000000000000000000000000000000000000000000000000000000000000000000000000000
0000000000000000000000000000000000000000000000000000000000000000000000000000000000000000000000000000000000000000000000000000000000000000000000000000000000000000000000000000000000
0000000000000000000000000000000000000000000000000000000000000000000000000000000000000000000000000000000000000000000000000000000000000000000000000000000000000000000000000000000000
0000000000000000000000000000000000000000000000000000000000000000000000000000000000000000000000000000000000000000000000000000000000000000000000000000000000000000000000000000000000
0000000000000000000000000000000000000000000000000000000000000000000000000000000000000000000000000000000000000000000000000000000000000000000000000000000000000000000000000000000000
0000000000000000000000000000000000000000000000000000000000000000000000000000000000000000000000000000000000000000000000000000000000000000000000000000000000000000000000000000000000
0000000000000000000000000000000000000000000000000000000000000000000000000000000000000000000000000000000000000000000000000000000000000000000000000000000000000000000000000000000000
0000000000000000000000000000000000000000000000000000000000000000000000000000000000000000000000000000000000000000000000000000000000000000000000000000000000000000000000000000000000
0000000000000000000000000000000000000000000000000000000000000000000000000000000000000000000000000000000000000000000000000000000000000000000000000000000000000000000000000000000000
0000000000000000000000000000000000000000000000000000000000000000000000000000000000000000000000000000000000000000000000000000000000000000000000000000000000000000000000000000000000
0000000000000000000000000000000000000000000000000000000000000000000000000000000000000000000000000000000000000000000000000000000000000000000000000000000000000000000000000000000000
00000000000000000000000000000000000000000000000000000000000000000000000000
0000000000000000000000000000000000000000000000000000000000000000000000000000000000000000000000000000000000000000000000000000000000000000000000000000000000000000000000000000000000
0000000000000000000000000000000000000000000000000000000000000000000000000000000000000000000000000000000000000000000000000000000000000000000000000000000000000000000000000000000000
0000000000000000000000000000000000000000000000000000000000000000000000000000000000000000000000000000000000000000000000000000000000000000000000000000000000000000000000000000000000
0000000000000000000000000000000000000000000000000000000000000000000000000000000000000000000000000000000000000000000000000000000000000000000000000000000000000000000000000000000000
0000000000000000000000000000000000000000000000000000000000000000000000000000000000000000000000000000000000000000000000000000000000000000000000000000000000000000000000000000000000
0000000000000000000000000000000000000000000000000000000000000000000000000000000000000000000000000000000000000000000000000000000000000000000000000000000000000000000000000000000000
0000000000000000000000000000000000000000000000000000000000000000000000000000000000000000000000000000000000000000000000000000000000000000000000000000000000000000000000000000000000
0000000000000000000000000000000000000000000000000000000000000000000000000000000000000000000000000000000000000000000000000000000000000000000000000000000000000000000000000000000000
0000000000000000000000000000000000000000000000000000000000000000000000000000000000000000000000000000000000000000000000000000000000000000000000000000000000000000000000000000000000
0000000000000000000000000000000000000000000000000000000000000000000000000000000000000000000000000000000000000000000000000000000000000000000000000000000000000000000000000000000000
00000000000000000000000000000000000000000000000000000000000000000000000000
>Q70Z35
MSEDSRGDSRAESAKDLEKQLRLRVCVLSELQKTERDYVGTLEFLVSAFLHRMNQCAASKVDKNVTEETVKMLFSNIEDILAVHKEFLKVVEECLHPEPNAQQEVGTCFLHFKDKFRIYDEYCSNHEKAQKLLLELNKIRTIRTFLLNCMLLGGRKNTDVPLEGYLVTPI
QRICKYPLILKELLKRTPRKHSDYAAVMEALQAMKAVCSNINEAKRQMEKLEVLEEWQSHIEGWEGSNITDTCTEMLMCGVLLKISSGNIQERVFFLFDNLLVYCKRKHRRLKNSKASTDGHRYLFRGRINTEVMEVENVDDGTADFHSSGHIVVNGWKIHNTAKN
KWFVCMAKTPEEKHEWFEAILKERERRKGLKLGMEQDTWVMISEQGEKLYKMMCRQGNLIKDRKRKLTTFPKCFLGSEFVSWLLEIGEIHRPEEGVHLGQALLENGIIHHVTDKHQFKPEQMLYRFRYDDGTFYPRNEMQDVISKGVRLYCRLHSLFTPVIRDKDY
HLRTYKSVVMANKLIDWLIAQGDCRTREEAMIFGVGLCDNGFMHHVLEKSEFKDEPLLFRFFSDEEMEGSNMKHRLMKHDLKVVENVIAKSLLIKSNEGSYGFGLEDKNKVPIIKLVEKGSNAEMAGMEVGKKIFAINGDLVFMRPFNEVDCFLKSCLNSRKPLRVL
VSTKPRETVKIPDSADGLGFQIRGFGPSVVHAVGRGTVAAAAGLHPGQCIIKVNGINVSKETHASVIAHVTACRKYRRPTKQDSIQWVYNSIESAQEDLQKSHSKPPGDEAGDAFDCKVEEVIDKFNTMAIIDGKKEHVSLTVDNVHLEYGVVYEYDSTAGIKCNVVE
KMIEPKGFFSLTAKILEALAKSDEHFVQNCTSLNSLNEVIPTDLQSKFSALCSERIEHLCQRISSYKKFSRVLKNRAWPTFKQAKSKISPLHSSDFCPTNCHVNVMEVSYPKTSTSLGSAFGVQLDSRKHNSHDKENKSSEQGKLSPMVYIQHTITTMAAPSGLSLGQQD
GHGLRYLLKEEDLETQDIYQKLLGKLQTALKEVEMCVCQIDDLLSSITYSPKLERKTSEGIIPTDSDNEKGERNSKRVCFNVAGDEQEDSGHDTISNRDSYSDCNSNRNSIASFTSICSSQCSSYFHSDEMDSGDELPLSVRISHDKQDKIHSCLEHLFSQVDSITNLLKGQ
AVVRAFDQTKYLTPGRGLQEFQQEMEPKLSCPKRLRLHIKQDPWNLPSSVRTLAQNIRKFVEEVKCRLLLALLEYSDSETQLRRDMVFCQTLVATVCAFSEQLMAALNQMFDNSKENEMETWEASRRWLDQIANAGVLFHFQSLLSPNLTDEQAMLEDTLVALFD
LEKVSFYFKPSEEEPLVANVPLTYQAEGSRQALKVYFYIDSYHFEQLPQRLKNGGGFKIHPVLFAQALESMEGYYYRDNVSVEEFQAQINAASLEKVKQYNQKLRAFYLDKSNSPPNSTSKAAYVDKLMRPLNALDELYRLVASFIRSKRTAACANTACSASGVGLLSVS
SELCNRLGACHIIMCSSGVHRCTLSVTLEQAIILARSHGLPPRYIMQATDVMRKQGARVQNTAKNLGVRDRTPQSAPRLYKLCEPPPPAGEE
0000000000000000000000000000000000000000000000000000000000000000000000000000000000000000000000000000000000000000000000000000000000000000000000000000000000000000000000000000000000
0000000000000000000000000000000000000000000000000000000000000000000000000000000000000000000000000000000000000000000000000000000000000000000000000000000000000000000000000000000000
0000000000000000000000000000000000000000000000000000000000000000000000000000000000000000000000000000000000000000000000000000000000000000000000000000000000000000000000000000000000
0000000000000000000000000000000000000000000000000000000000000000000000000000000000000000000000000000000000000000000000000000000000000000000000000000000000000000000000000000000000
0000000000000000000000000000000000000000000000000000000000000000000000000000000000000000000000000000000000000000000000000000000000000000000000000000000000000000000000000000000000
0000000000000000000000000000000000000000000000000000000000000000000000000000000000000000000000000000000000000000000000000000000000000000000000000000000000000000000000000000000000
0000000000000000000000000000000000000000000000000000000000000000000000000000000000000000000000000000000000000000000000000000000000000000000000000000000000000000000000000000000000
0000000000000000000000000000000000000000000000000000000000000000000000000000000000000000000000000000000000000000000000000000000000000000000000000000000000000000000000000000000000
0000000000000000000000000000000000000000000000000000000000000000000000000000000000000000000000000000000000000000000000000000000000000000000000000000000000000000000000000000000000
0000000000000000000000000000000000000000000000000000000000000000000000000000000000000000000000000000000000000000000000000000000000000000000000000000000000000000000000000000000000
0000000000000000000000000000000000000000000000000000000000000000000000000000000000000000000000000000000000000000000000000000000000000000000000000000000000000000000000000000000000
0000000000000000000000
0000000000000000000000000000000000000000000000000000000000000000000000000000000000000000000000000000000000000000000000000000000000000000000000000000000000000000000000000000000000
0000000000000000000000000000000000000000000000000000000000000000000000000000000000000000000000000000000000000000000000000000000000000000000000000000000000000000000000000000000000
0000000000000000000000000000000000000000000000000000000000000000000000000000000000000000000000000000000000000000000000000000000000000000000000000000000000000000000000000000000000
0000000000000000000000000000000000000000000000000000000000000000000000000000000000000000000000000000000000000000000000000000000000000000000000000000000000000000000000000000000000
0000000000000000000000000000000000000000000000000000000000000000000000000000000000000000000000000000000000000000000000000000000000000000000000000000000000000000000000000000000000
0000000000000000000000000000000000000000000000000000000000000000000000000000000000000000000000000000000000000000000000000000000000000000000000000000000000000000000000000000000000
0000000000000000000000000000000000000000000000000000000000000000000000000000000000000000000000000000000000000000000000000000000000000000000000000000000000000000000000000000000000
0000000000000000000000000000000000000000000000000000000000000000000000000000000000000000000000000000000000000000000000000000000000000000000000000000000000000000000000000000000000
0000000000000000000000000000000000000000000000000000000000000000000000000000000000000000000000000000000000000000000000000000000000000000000000000000000000000000000000000000000000
0000000000000000000000000000000000000000000000000000000000000000000000000000000000000000000000000000000000000000000000000000000000000000000000000000000000000000000000000000000000
0000000000000000000000000000000000000000000000000000000000000000000000000000000000000000000000000000000000000000000000000000000000000000000000000000000000000000000000000000000000
0000000000000000000000
0000000000000000000000000000000000000000000000000000000000000000000000000000000000000000000000000000000000000000000000000000000000000000000000000000000000000000000000000000000000
0000000000000000000000000000000000000000000000000000000000000000000000000000000000000000000000000000000000000000000000000000000000000000000000000000000000000000000000000000000000
0000000000000000000000000000000000000000000000000000000000000000000000000000000000000000000000000000000000000000000000000000000000000000000000000000000000000000000000000000000000
0000000000000000000000000000000000000000000000000000000000000000000000000000000000000000000000000000000000000000000000000000000000000000000000000000000000000000000000000000000000
0000000000000000000000000000000000000000000000000000000000000000000000000000000000000000000000000000000000000000000000000000000000000000000000000000000000000000000000000000000000
0000000000000000000000000000000000000000000000000000000000000000000000000000000000000000000000000000000000000000000000000000000000000000000000000000000000000000000000000000000000
0000000000000000000000000000000000000000000000000000000000000000000000000000000000000000000000000000000000000000000000000000000000000000000000000000000000000000000000000000000000
0000000000000000000000000000000000000000000000000000000000000000000000000000000000000000000000000000000000000000000000000000000000000000000000000000000000000000000000000000000000
0000000000000000000000000000000000000000000000000000000000000000000000000000000000000000000000000000000000000000000000000000000000000000000000000000000000000000000000000000000000
0000000000000000000000000000000000000000000000000000000000000000000000000000000000000000000000000000000000000000000000000000000000000000000000000000000000000000000000000000000000
0000000000000000000000000000000000000000000000000000000000000000000000000000000000000000000000000000000000000000000000000000000000000000000000000000000000000000000000000000000000
0000000000000000000000
>P76578
MKKLRVAACMLMLALAGCDNNDNAPTAVKKDAPSEVTKAASSENASSAKLSVPERQKLAQQSAGKVLTLLDLSEVQLDGAATLVLTFSIPLDPDQDFSRVIHVVDKKSGKVDGAWELSDNLKELRLRHLEPKRDLIVTIGKEVKALNNATFSKDYEKTITTRDIQPSV
GFASRGSLLPGKVVEGLPVMALNVNNVDVNFFRVKPESLPAFISQWEYRNSLANWQSDKLLQMADLVYTGRFDLNPARNTREKLLLPLGDIKPLQQAGVYLAVMNQAGRYDYSNPATLFTLSDIGVSAHRYHNRLDIFTQSLENGAAQQGIEVSLLNEKGQTLTQ
ATSDAQGHVQLENDKNAALLLARKDGQTTLLDLKLPALDLAEFNIAGAPGYSKQFFMFGPRDLYRPGETVILNGLLRDADGKALPNQPIKLDVIKPDGQVLRSVVSQPENGLYHFTWPLDSNAATGMWHIRANTGDNQYRMWDFHVEDFMPERMALNLTGEK
TPLTPKDEVKFSVVGYYLYGAPANGNTLQGQLFLRPLREAVSALPGFEFGDIAAENLSRTLDEVQLTLDDKGRGEVSTESQWKETHSPLQVIFQGSLLESGGRPVTRRAEQAIWPADALPGIRPQFASKSVYDYRTDSTVKQPIVDEGSNAAFDIVYSDAQGVKKAVS
GLQVRLIRERRDYYWNWSEDEGWQSQFDQKDLIENEQTLDLKADETGKVSFPVEWGAYRLEVKAPNEAVSSVRFWAGYSWQDNSDGSGAVRPDRVTLKLDKASYRPGDTIKLHIAAPTAGKGYAMVESSEGPLWWQEIDVRAQGLDLTIPVDKTWNRHDLYL
STLVVRPGDKSRSATPKRAVGVLHLPLGDENRRLDLALETPAKMRPNQPLTVKIKASTKNGEKPKQVNVLVSAVDSGVLNITDYVTPDPWQAFFGQKRYGADIYDIYGQVIEGQGRLAALRFGGDGDELKRGGKPPVNHVNIVVQQALPVTLNEQGEGSVTLPIGD
FNGELRVMAQAWTADDFGSNESKVIVAAPVIAELNMPRFMASGDTSRLTLDITNLTDKPQKLNVALTASGLLELVSDSPAAVELAPGVRTTLFIPVRALPGYGDGEIQATISGLALPGETVADQHKQWKIGVRPAFPAQTVNYGTALQPGETWAIPADGLQNFSPV
TLEGQLLLSGKPPLNIARYIKELKAYPYGCLEQTASGLFPSLYTNAAQLQALGIKGDSDEKRRASVDIGISRLLQMQRDNGGFALWDKNGDEEYWLTAYVMDFLVRAGEQGYSVPTDAINRGNERLLRYLQDPGMMSIPYADNLKASKFAVQSYAALVLARQQKAP
LGALREIWEHRADAASGLPLLQLGVALKTMGDATRGEEAIALALKTPRNSDERIWLGDYGSSLRDNALMLSLLEENKLLPDEQYTLLNTLSQQAFGERWLSTQESNALFLAARTIQDLPGKWQAQTSFSAEQLTGEKAQNSNLNSDQLVTLQVSNSGDQPLWLRM
DASGYPQSAPLPANNVLQIERHILGTDGKSKSLDSLRSGDLVLVWLQVKASNSVPDALVVDLLPAGLELENQNLANGSASLEQSGGEVQNLLNQMQQASIKHIEFRDDRFVAAVAVDEYQPVTLVYLARAVTPGTYQVPQPMVESMYVPQWRATGAAEDLLIVR
P
0000000000000000000000000000000000000000000000000000000000000000000000000000000000000000000000000000000000000000000000000000000000000000000000000000000000000000000000000000000000
0000000000000000000000000000000000000000000000000000000000000000000000000000000000000000000000000000000000000000000000000000000000000000000000000000000000000000000000000000000000
0000000000000000000000000000000000000000000000000000000000000000000000000000000000000000000000000000000000000000000000000000000000000000000000000000000000000000000000000000000000
0000000000000000000000000000000000000000000000000000000000000000000000000000000000000000000000000000000000000000000000000000000000000000000000000000000000000000000000000000000000
0000000000000000000000000000000000000000000000000000000000000000000000000000000000000000000000000000000000000000000000000000000000000000000000000000000000000000000000000000000000
0000000000000000000000000000000000000000000000000000000000000000000000000000000000000000000000000000000000000000000000000000000000000000000000000000000000000000000000000000000000
0000000000000000000000000000000000000000000000000000000000000000000000000000000000000000000000000000000000000000000000000000000000000000000000000000000000000000000000000000000000
0000000000000000000000000000000000000000000000000000000000000000000000000000000000000000000000000000000000000000000000000000000000000000000000000000000000000000000000000000000000
0000000000000000000000000000000000000000000000000000000000000000000000000000000000000000000000000000000000000000000000000000000000000000000000000000000000000000000000000000000000
0000000000000000000000000000000000000000000000000000000000000000000000000000000000000000000000000000000000000000000000000000000000000000000000000000000000000000000000000000000000
00000000000000000000000000000000000000000000000000000000000000000000
0000000000000000000000000000000000000000000000000000000000000000000000000000000000000000000000000000000000000000000000000000000000000000000000000000000000000000000000000000000000
0000000000000000000000000000000000000000000000000000000000000000000000000000000000000000000000000000000000000000000000000000000000000000000000000000000000000000000000000000000000
0000000000000000000000000000000000000000000000000000000000000000000000000000000000000000000000000000000000000000000000000000000000000000000000000000000000000000000000000000000000
0000000000000000000000000000000000000000000000000000000000000000000000000000000000000000000000000000000000000000000000000000000000000000000000000000000000000000000000000000000000
0000000000000000000000000000000000000000000000000000000000000000000000000000000000000000000000000000000000000000000000000000000000000000000000000000000000000000000000000000000000
0000000000000000000000000000000000000000000000000000000000000000000000000000000000000000000000000000000000000000000000000000000000000000000000000000000000000000000000000000000000
0000000000000000000000000000000000000000000000000000000000000000000000000000000000000000000000000000000000000000000000000000000000000000000000000000000000000000000000000000000000
0000000000000000000000000000000000000000000000000000000000000000000000000000000000000000000000000000000000000000000000000000000000000000000000000000000000000000000000000000000000
0000000000000000000000000000000000000000000000000000000000000000000000000000000000000000000000000000000000000000000000000000000000000000000000000000000000000000000000000000000000
0000000000000000000000000000000000000000000000000000000000000000000000000000000000000000000000000000000000000000000000000000000000000000000000000000000000000000000000000000000000
00000000000000000000000000000000000000000000000000000000000000000000
0000000000000000000000000000000000000000000000000000000000000000000000000000000000000000000000000000000000000000000000000000000000000000000000000000000000000000000000000000000000
0000000000000000000000000000000000000000000000000000000000000000000000000000000000000000000000000000000000000000000000000000000000000000000000000000000000000000000000000000000000
0000000000000000000000000000000000000000000000000000000000000000000000000000000000000000000000000000000000000000000000000000000000000000000000000000000000000000000000000000000000
0000000000000000000000000000000000000000000000000000000000000000000000000000000000000000000000000000000000000000000000000000000000000000000000000000000000000000000000000000000000
0000000000000000000000000000000000000000000000000000000000000000000000000000000000000000000000000000000000000000000000000000000000000000000000000000000000000000000000000000000000
0000000000000000000000000000000000000000000000000000000000000000000000000000000000000000000000000000000000000000000000000000000000000000000000000000000000000000000000000000000000
0000000000000000000000000000000000000000000000000000000000000000000000000000000000000000000000000000000000000000000000000000000000000000000000000000000000000000000000000000000000
0000000000000000000000000000000000000000000000000000000000000000000000000000000000000000000000000000000000000000000000000000000000000000000000000000000000000000000000000000000000
0000000000000000000000000000000000000000000000000000000000000000000000000000000000000000000000000000000000000000000000000000000000000000000000000000000000000000000000000000000000
0000000000000000000000000000000000000000000000000000000000000000000000000000000000000000000000000000000000000000000000000000000000000000000000000000000000000000000000000000000000
00000000000000000000000000000000000000000000000000000000000000000000
>Q9GRZ3
MRSTTFDKNSGAPEEPRRPRGPRTPDGEPTEANERPCSSSSSLSNDSFVPAPQGPQNGHTSSSHDNDYSEYRPRRGPKTPPLPPPDEPVKQQQQQPIVAPYSYYPTYGSSTGYPYPYPTMMMPQQGIPGPSQHPATPSYYIQHPPPPSMSNGAPYYPHMNPSP
YYQPRPNYVTQPPAPIIPPPQPPVMTASAAAAAYRDRLPPPPPKPPMPDLRTKEPIGIRSWNGSGIPPPPILPPVACLSSTMSPGMLRSPVPRERFNSVESNGSFSLHPPPPVPPPAKKPLNMDVRELLNGAHQTTVESVKKDPPAPQPRPRPIPVPPTTSYNSSFCG
LLPPPPVPPDLRASAASAAASPPMVQDASFSSVLTPKTKAPEKIPSPPATVTSSTDSLEHMQRKRKKQQETEEQEKAAKKAKRHQEERERQKIEAEKRKKAILEEAAKVTRPPAEKKPPVVVEETIRAVPFVFKQEMPLPFVLAAAAAAAKEPETRPEPRPEPKQNGYH
VKQAEPLQAEPPQNGYRLAAAQALQHEPRQASEPLQNVQPEPSKQASELVQNGYAAPQEPRRATPPPEPLRNELPKHSDLLNVSKPSEPAEPPKPSDAPREPEVAAAEAVIPEPSPAVSVEEPPRREERAASMAGIPPPPPTSSRHSPVPRREDRAEFMARSTPPAP
GKDRKSLGGRMAMWRNKKNKTASMVGPPEPKELPAVVEVEAIVEPVVEDQPEPVVSEELKPTEPEPVEPEPMEVEPVVAKEVVEEVVEEVVETQEPWVPLSECSRSSSLELLAELPPPPRASEPPPPPPPPVATAPVPASMEEDEIIDIETIDEITSTSTKKPEKVEKIK
KAPVVTKAALKMVGRPKKTPGRRRKKDRGASPSPSPSPPPPPETTLTPIVLPRKKQRIEKKKLTPPPPQQAPVTSHTPPPVEQLMSRKKQIMMEHSSLDHIQFKLIEIELAAKRRKAEAAAKAAAAVEQKDEKAEEVENRETPPGPSTSMRSSSLHTPNTSEEDEVIFVE
PSTLEKPERRNGTTEERVMTADQRAMFDAKIEEARRSRMSTRDCSVVSTLGPVKSKASQRLHDIIEGKEELEDSMDDPTNNNGTLAGILYPMRSERAESVSSNHRSEGAGGSMSLKHHLARKNELKEEANVARRSEILKAVVKRQREIGVPTTLMSKSAIELVEEDEK
ERKNHKNNKTLSHPDYVRSKNEAEKAEFHGKGGTMRITNRNLKMLTRQFDLPKMSSRFRKFVRIRRHPNGMATIISCDYNQIKQHLGPNEMKHFERQFVRLGFAENNGVPLFAIGVMENAAEALHDQFEWLAKNSPNTQVKVGSLTNKQFIETMPMKKYYESA
METLDMGTFRFGPLMSLSMVGTKNEEAGGNFKEMLDALNAAPFLGPIMPWGDFSEVQGIKEDTSDDGPIFWVRPGEQMVPTDGKNRSTEPRHPLATRGNDRRETAFNDRTNAHADQVRESTEDDPTTTTTTTTTSSSSSSSKSKKSAKSDPTFVKSTAAVGVL
QGIRNPDANDDDEYYEDERKAVKEVIVFDAHDLHKVAHHLAMDLYEPPVSQCHRWVDDAILNTMRREGIRYAKLELHENDMYFLPRNVIHQFRTVSACSSVAWHVRLRHYYDVDQPASLSDPQFECDSDYSDDGDFDD
0000000000000000000000000000000000000000000000000000000000000000000000000000000000000000000000000000000000000000000000000000000000000000000000000000000000000000000000000000000000
0000000000000000000000000000000000000000000000000000000000000000000000000000000000000000000000000000000000000000000000000000000000000000000000000000000000000000000000000000000000
0000000000000000000000000000000000000000000000000000000000000000000000000000000000000000000000000000000000000000000000000000000000000000000000000000000000000000000000000000000000
0000000000000000000000000000000000000000000000000000000000000000000000000000000000000000000000000000000000000000000000000000000000000000000000000000000000000000000000000000000000
0000000000000000000000000000000000000000000000000000000000000000000000000000000000000000000000000000000000000000000000000000000000000000000000000000000000000000000000000000000000
0000000000000000000000000000000000000000000000000000000000000000000000000000000000000000000000000000000000000000000000000000000000000000000000000000000000000000000000000000000000
0000000000000000000000000000000000000000000000000000000000000000000000000000000000000000000000000000000000000000000000000000000000000000000000000000000000000000000000000000000000

0000000000000000000000000000000000000000000000000000000000000000000000000000000000000000000000000000000000000000000000000000000000000000000000000000000000000000000000000000000000000000000
0000000000000000000000000000000000000000000000000000000000000000000000000000000000000000000000000000000000000000000000000000000000000000000000000000000000000000000000000000000000000000000
0000000000000000000000000000000000000000000000000000000000000
0000000000000000000000000000000000000000000000000000000000000000000000000000000000000000000000000000000000000000000000000000000000000000000000000000000000000000000000000000000000000000000
0000000000000000000000000000000000000000000000000000000000000000000000000000000000000000000000000000000000000000000000000000000000000000000000000000000000000000000000000000000000000000000
0000000000000000000000000000000000000000000000000000000000000000000000000000000000000000000000000000000000000000000000000000000000000000000000000000000000000000000000000000000000000000000
0000000000000000000000000000000000000000000000000000000000000000000000000000000000000000000000000000000000000000000000000000000000000000000000000000000000000000000000000000000000000000000
0000000000000000000000000000000000000000000000000000000000000000000000000000000000000000000000000000000000000000000000000000000000000000000000000000000000000000000000000000000000000000000
0000000000000000000000000000000000000000000000000000000000000000000000000000000000000000000000000000000000000000000000000000000000000000000000000000000000000000000000000000000000000000000
0000000000000000000000000000000000000000000000000000000000000000000000000000000000000000000000000000000000000000000000000000000000000000000000000000000000000000000000000000000000000000000
0000000000000000000000000000000000000000000000000000000000000000000000000000000000000000000000000000000000000000000000000000000000000000000000000000000000000000000000000000000000000000000
0000000000000000000000000000000000000000000000000000000000000000000000000000000000000000000000000000000000000000000000000000000000000000000000000000000000000000000000000000000000000000000
0000000000000000000000000000000000000000000000000000000000000000000000000000000000000000000000000000000000000000000000000000000000000000000000000000000000000000000000000000000000000000000
0000000000000000000000000000000000000000000000000000000000000
0000000000000000000000000000000000000000000000000000000000000000000000000000000000000000000000000000000000000000000000000000000000000000000000000000000000000000000000000000000000000000000
0000000000000000000000000000000000000000000000000000000000000000000000000000000000000000000000000000000000000000000000000000000000000000000000000000000000000000000000000000000000000000000
0000000000000000000000000000000000000000000000000000000000000000000000000000000000000000000000000000000000000000000000000000000000000000000000000000000000000000000000000000000000000000000
0000000000000000000000000000000000000000000000000000000000000000000000000000000000000000000000000000000000000000000000000000000000000000000000000000000000000000000000000000000000000000000
0000000000000000000000000000000000000000000000000000000000000000000000000000000000000000000000000000000000000000000000000000000000000000000000000000000000000000000000000000000000000000000
0000000000000000000000000000000000000000000000000000000000000000000000000000000000000000000000000000000000000000000000000000000000000000000000000000000000000000000000000000000000000000000
0000000000000000000000000000000000000000000000000000000000000000000000000000000000000000000000000000000000000000000000000000000000000000000000000000000000000000000000000000000000000000000
0000000000000000000000000000000000000000000000000000000000000000000000000000000000000000000000000000000000000000000000000000000000000000000000000000000000000000000000000000000000000000000
0000000000000000000000000000000000000000000000000000000000000000000000000000000000000000000000000000000000000000000000000000000000000000000000000000000000000000000000000000000000000000000
0000000000000000000000000000000000000000000000000000000000000000000000000000000000000000000000000000000000000000000000000000000000000000000000000000000000000000000000000000000000000000000
0000000000000000000000000000000000000000000000000000000000000
>P38198
MSSFNNETNNNSNTNTHPDDSFPLYTVFKDESVPIEEKMALLTRFKGHVKKELVNESSIQAYFAALLFISGHYAYRSYPRLIFLSHSSLCYLIKRVAMQSPVQFNDTLVEQLLNHLIFELPNEKKFWLASIKAIEAIYLVNPSKIQAILANFLRRPSENQNGDYLNRIKSTLLTI
DELIQINEKNNSNHLQLLRFFMLSFTNLLNNNLNEHANDDNNNVIIELIFDIMYKYLKMDDENSQDLIDGFINDLEVEKFKQKFISLAKSQDQHGSQEDKSTLFDEEYEFQLLLAEAKLPQLSNNLSSKDPAMKKNYESLNQLQQDLENLLAPFQSVKETEQNWKLRQ
SNIIELDNIISGNIPKDNPEEFVTVIKEVQLIELISRATSSLRTTLSLTALLFLKRLIHILNDQLPLSILDQIFVIFKNLLSSTKKISSQTAFHCLITLIIDINHFHNKLFQLSFLLINEKTVTPRFCSAILLRSFLIKFNDSNLSLNNSNTTSPTSKLENNIIYIEEWLKKGISDSQTTVREAM
RLTFWYFYKCYPTNAKRLLSSSFSPQLKKATELAIPAHLNINYQVSRVSSTASASSATSRLYSHSSNNSSRKTSLLEQKRNYPSYAQPTQSSSTSLLNAPAVTAGGSVIASKLSNKLKTNLRSTSEYSSKENEKRARHHDSMNSVSNSNTKDNNNVTKRKVSAPPSSTAAT
KVSENYTNFDDFPSNQIDLTDELSNSYSNPLIKKYMDKNDVSMSSSPISLKGSNKLGEYETLYKKFNDASFPAQIKDALQYLQKELLLTSQQSSSAPKFEFPMIMKKLRQIMIKSPNDFKPFLSIEKFTNGVPLNYLIELYSINSFDYAEILKNRMNPEKPYELTNLIITIADLF
NFLNANNCPNDFKLYYMKYKTTFFNYNFKLLLEIFRNLNIKHDNTLRSGTNDLMPKISMILFQIYGKEFDYTCYFNLIFEIYKFDNNRFNKLLADFDIVSTKMKICHELEKKDANFKVEDIISRESSVSFTPIDNKKSEGDEESDDAVDENDVKKCMEMTMINPFKNLETD
KTLELKNNVGKRTSSTDSVVIHDDNDKDKKLSEMTKIVSVYQLDQPNPAKEEDDIDMENSQKSDLNLSEIFQNSGENTERKLKDDNEPTVKFSTDPPKIINEPEKLIGNGNENEKPDLETMSPIKINGDENMGQKQRITVKRERDVALTEQDINSKKMKLVNNKKSEK
MHLLIMDNFPRDSLTVYEISHLLMVDSNGNTLMDFDVYFNHMSKAINRIKSGSFTMKHINYLIEPLITCFQNQKMTDWLTNENGFDELLDVAIMLLKSTDDTPSIPSKISSKSIILVHCLLVWKKFLNTLSENADDDGVSVRMCFEEVWEQILLMLNKFSDYGNEIYKL
AQEFRDSLMLSHFFKKHSATRILSMLVTEIQPDTAGVKETFLIETLWKMLQSPTICQQFKKSNISEIIQTMSYFIMGTDNTSWNFTSAVVLARCLRVLQTTPDYTEQETERLFDCLPKNVFKMIMFIASNE
0000000000000000000000000000000000000000000000000000000000000000000000000000000000000000000000000000000000000000000000000000000000000000000000000000000000000000000000000000000000000000000
0000000000000000000000000000000000000000000000000000000000000000000000000000000000000000000000000000000000000000000000000000000000000000000000000000000000000000000000000000000000000000000
0000000000000000000000000000000000000000000000000000000000000000000000000000000000000000000000000000000000000000000000000000000000000000000000000000000000000000000000000000000000000000000
0000000000000000000000000000000000000000000000000000000000000000000000000000000000000000000000000000000000000000000000000000000000000000000000000000000000000000000000000000000000000000000
0000000000000000000000000000000000000000000000000000000000000000000000000000000000000000000000000000000000000000000000000000000000000000000000000000000000000000000000000000000000000000000
0000000000000000000000000000000000000000000000000000000000000000000000000000000000000000000000000000000000000000000000000000000000000000000000000000000000000000000000000000000000000000000
0000000000000000000000000000000000000000000000000000000000000000000000000000000000000000000000000000000000000000000000000000000000000000000000000000000000000000000000000000000000000000000
0000000000000000000000000000000000000000000000000000000000000000000000000000000000000000000000000000000000000000000000000000000000000000000000000000000000000000000000000000000000000000000
0000000000000000000000000000000000000000000000000000000000000000000000000000000000000000000000000000000000000000000000000000000000000000000000000000000000000000000000000000000000000000000
00000000000000000000000000000000000000000000000000000000000000000000000000000000000000000000000000000000000000000
0000000000000000000000000000000000000000000000000000000000000000000000000000000000000000000000000000000000000000000000000000000000000000000000000000000000000000000000000000000000000000000
0000000000000000000000000000000000000000000000000000000000000000000000000000000000000000000000000000000000000000000000000000000000000000000000000000000000000000000000000000000000000000000
0000000000000000000000000000000000000000000000000000000000000000000000000000000000000000000000000000000000000000000000000000000000000000000000000000000000000000000000000000000000000000000
0000000000000000000000000000000000000000000000000000000000000000000000000000000000000000000000000000000000000000000000000000000000000000000000000000000000000000000000000000000000000000000
0000000000000000000000000000000000000000000000000000000000000000000000000000000000000000000000000000000000000000000000000000000000000000000000000000000000000000000000000000000000000000000
0000000000000000000000000000000000000000000000000000000000000000000000000000000000000000000000000000000000000000000000000000000000000000000000000000000000000000000000000000000000000000000
0000000000000000000000000000000000000000000000000000000000000000000000000000000000000000000000000000000000000000000000000000000000000000000000000000000000000000000000000000000000000000000
0000000000000000000000000000000000000000000000000000000000000000000000000000000000000000000000000000000000000000000000000000000000000000000000000000000000000000000000000000000000000000000
0000000000000000000000000000000000000000000000000000000000000000000000000000000000000000000000000000000000000000000000000000000000000000000000000000000000000000000000000000000000000000000
00000000000000000000000000000000000000000000000000000000000000000000000000000000000000000000000000000000000000000
0000000000000000000000000000000000000000000000000000000000000000000000000000000000000000000000000000000000000000000000000000000000000000000000000000000000000000000000000000000000000000000
0000000000000000000000000000000000000000000000000000000000000000000000000000000000000000000000000000000000000000000000000000000000000000000000000000000000000000000000000000000000000000000
0000000000000000000000000000000000000000000000000000000000000000000000000000000000000000000000000000000000000000000000000000000000000000000000000000000000000000000000000000000000000000000
0000000000000000000000000000000000000000000000000000000000000000000000000000000000000000000000000000000000000000000000000000000000000000000000000000000000000000000000000000000000000000000
0000000000000000000000000000000000000000000000000000000000000000000000000000000000000000000000000000000000000000000000000000000000000000000000000000000000000000000000000000000000000000000
0000000000000000000000000000000000000000000000000000000000000000000000000000000000000000000000000000000000000000000000000000000000000000000000000000000000000000000000000000000000000000000
0000000000000000000000000000000000000000000000000000000000000000000000000000000000000000000000000000000000000000000000000000000000000000000000000000000000000000000000000000000000000000000
0000000000000000000000000000000000000000000000000000000000000000000000000000000000000000000000000000000000000000000000000000000000000000000000000000000000000000000000000000000000000000000
0000000000000000000000000000000000000000000000000000000000000000000000000000000000000000000000000000000000000000000000000000000000000000000000000000000000000000000000000000000000000000000
00000000000000000000000000000000000000000000000000000000000000000000000000000000000000000000000000000000000000000
>G0SB58
MMRLSQLPWGLAALLAAACLPSLQVAASPSINVALKAAFPSPPYLVELLETAASDNTTIYYSLLDRIAKGHFAEATTDKALYEKFLEVLRDDGHMDPEALSAFKLALSLRTATPRVEAHYQYYTATVEPSLSGTQEGCDQWFLIDGEQYCSPTLDTSHGKVKGEDQLRT
LPFDRKFGVGSRDVILYADITSKSFAPFHEVAMDLAKKGKASYRVRYRRSPSHSRESLSVNGYGVELVLKRTDYIVIDDRDTGAAAKPAEENDQKPLVGHETVLDDGEEIADIKPLEKSELAALGMKAASFVMQSEKPFEALLKLTQDFPKYSNSLGSQNVSAEFEAEH
RGNREVFLPEGSNVLWLNGLHLIDRQIQPFGLVDLLTRERKLIKSVLDLGLTGQQAVDLLGHAEVAHAKSGDDEPRRFDWRDDIEEGQVIIWLNNLEKDKRYKSFSPSIWVLIHHFGHGLPQIRRDVFNLVVPVDLTKADDVKIVVEGLLSFVKRLIPVRFGFVPLTPT
GQAIDQAKVVYYLLENYGLAAATAYLEKSYEEQSTGQPNERIFNEVIKDKSLRPDGVELSFKDIFISEKHEKQIHLSKHWVERLRAGGDVPTVFFDGFPIPREDNWLRVMNHRLMQDLQALQQAGYFGMLNESMWLPGFFLEKALSRRNTLIFPEDKNELTVLNVNK
IYIENHDLMSKVPVIEASKESTRDDWAALTVVADLDDIEGQELVYYALRFRKSNDGVRLDIVHNPKDTSRSPSVLAQRLKSREDKLLDFTRFLDLETALETGEFEPDVAYDASLANFLASSNMKAGDNFVILNGRVLGPITSADDFKKEDFEVFLQAERRTRILPVYKALE
DLGLDDKVSGPLSAAKLTSVTALSTISDLPQGIFDNAPTVRTTLFKQWNSTYTSFEVGDASTATIFFVAVINPASEIGQRWVAVLKVLSELEGVHLRVFLNPTVMIEELPVKRFYRYVLSSSPSFDESGKVKALSARFTGVPRETLLVVGMDVPPAWLVTSKVAVDDLDN
LRIKDIKAKRGTEHVEAIYELEHILIEGHSREIPGAHAPRGVQLVLETENNPHFADTIIMANLGYFQFKANPGVYNIRLKEGRSSEIFTLESVGAKGWGPIPGDDNTEVVLMDFQGTTLYPRLRRKPGMEEEDVLEPSTKSGEESGSGARNLVSRGIKFAEGLLGRGNKA
AEATKSVSKTEHAEINIFSVASGHLYERMLNIMMASVMHHTNHTVKFWFIEQFLSPSFKDFIPHMAAEYGFKYEMVTYKWPHWLRQQKEKQREIWGYKILFLDVLFPLSLDKVIFVDADQIVRTDMYDLVEHPLDGAPYGFAPMCDSRVEMEGYRFWKTGYWA
NYLKGKPYHISALYVVDLQRFRELAAGDRLRQQYHALSADPNSLANLDQDLPNHMQFTIPIATLPQEWLWCETWCSDETLKDARTIDLCNNPMTKEPKLDRARRQVPEWTKYDEEIAELARRVREEKPKKKEEEKVQKNPKSRRLDGDEEEVKTVREKDEL
0000000000000000000000000000000000000000000000000000000000000000000000000000000000000000000000000000000000000000000000000000000000000000000000000000000000000000000000000000000000000000000
0000000000000000000000000000000000000000000000000000000000000000000000000000000000000000000000000000000000000000000000000000000000000000000000000000000000000000000000000000000000000000000
0000000000000000000000000000000000000000000000000000000000000000000000000000000000000000000000000000000000000000000000000000000000000000000000000000000000000000000000000000000000000000000
0000000000000000000000000000000000000000000000000000000000000000000000000000000000000000000000000000000000000000000000000000000000000000000000000000000000000000000000000000000000000000000
0000000000000000000000000000000000000000000000000000000000000000000000000000000000000000000000000000000000000000000000000000000000000000000000000000000000000000000000000000000000000000000
0000000000000000000000000000000000000000000000000000000000000000000000000000000000000000000000000000000000000000000000000000000000000000000000000000000000000000000000000000000000000000000
0000000000000000000000000000000000000000000000000000000000000000000000000000000000000000000000000000000000000000000000000000000000000000000000000000000000000000000000000000000000000000000
0000000000000000000000000000000000000000000000000000000000000000000000000000000000000000000000000000000000000000000000000000000000000000000000000000000000000000000000000000000000000000000
0000000000000000000000000000000000000000000000000000000000000000000000000000000000000000000000000000000000000000000000000000000000000000000000000000000000000000000000000000000000000000000
000000000000000000000000000000000000000000000000000000000000000000000000000000000000000000000000000000000000
0000000000000000000000000000000000000000000000000000000000000000000000000000000000000000000000000000000000000000000000000000000000000000000000000000000000000000000000000000000000000000000
0000000000000000000000000000000000000000000000000000000000000000000000000000000000000000000000000000000000000000000000000000000000000000000000000000000000000000000000000000000000000000000
0000000000000000000000000000000000000000000000000000000000000000000000000000000000000000000000000000000000000000000000000000000000000000000000000000000000000000000000000000000000000000000
0000000000000000000000000000000000000000000000000000000000000000000000000000000000000000000000000000000000000000000000000000000000000000000000000000000000000000000000000000000000000000000
0000000000000000000000000000000000000000000000000000000000000000000000000000000000000000000000000000000000000000000000000000000000000000000000000000000000000000000000000000000000000000000
0000000000000000000000000000000000000000000000000000000000000000000000000000000000000000000000000000000000000000000000000000000000000000000000000000000000000000000000000000000000000000000
0000000000000000000000000000000000000000000000000000000000000000000000000000000000000000000000000000000000000000000000000000000000000000000000000000000000000000000000000000000000000000000
0000000000000000000000000000000000000000000000000000000000000000000000000000000000000000000000000000000000000000000000000000000000000000000000000000000000000000000000000000000000000000000
0000000000000000000000000000000000000000000000000000000000000000000000000000000000000000000000000000000000000000000000000000000000000000000000000000000000000000000000000000000000000000000
000000000000000000000000000000000000000000000000000000000000000000000000000000000000000000000000000000000000
0000000000000000000000000000000000000000000000000000000000000000000000000000000000000000000000000000000000000000000000000000000000000000000000000000000000000000000000000000000000000000000
0000000000000000000000000000000000000000000000000000000000000000000000000000000000000000000000000000000000000000000000000000000000000000000000000000000000000000000000000000000000000000000
0000000000000000000000000000000000000000000000000000000000000000000000000000000000000000000000000000000000000000000000000000000000000000000000000000000000000000000000000000000000000000000
0000000000000000000000000000000000000000000000000000000000000000000000000000000000000000000000000000000000000000000000000000000000000000000000000000000000000000000000000000000000000000000
0000000000000000000000000000000000000000000000000000000000000000000000000000000000000000000000000000000000000000000000000000000000000000000000000000000000000000000000000000000000000000000
0000000000000000000000000000000000000000000000000000000000000000000000000000000000000000000000000000000000000000000000000000000000000000000000000000000000000000000000000000000000000000000
0000000000000000000000000000000000000000000000000000000000000000000000000000000000000000000000000000000000000000000000000000000000000000000000000000000000000000000000000000000000000000000
0000000000000000000000000000000000000000000000000000000000000000000000000000000000000000000000000000000000000000000000000000000000000000000000000000000000000000000000000000000000000000000
0000000000000000000000000000000000000000000000000000000000000000000000000000000000000000000000000000000000000000000000000000000000000000000000000000000000000000000000000000000000000000000
000000000000000000000000000000000000000000000000000000000000000000000000000000000000000000000000000000000000
>A0A2D0TCJ6
GSADINIFSVASGHLYERMLNIMMVSVMKHTKHSVKFWFIEQFLSPSFKKFLPHLAKEYGFSYEMVTYKWPHWLRGQREKQREIWGYKILFLDVLFPLSLDKVIFVDADQIVRTDMYDLVQLDLEGAPYGFTPMCDSRKEMEGFRFWKQGYWKSHLRGRPYHISA
LYVVDLNRFRALAAGDRLRGQYHTLSADPNSLANLDQDLPNNMQAMIPIKSLPQEWLWCETWCADEDLKTARTIDLCNNPLTKEPKLDRARRQVPEWTEYDNEIAELAVRVRAASKQSRREEDEKKDV
0000000000000000000000000000000000000000000000000000000000000000000000000000000000000000000000000000000000000000000000000000000000000000000000000000000000000000000000000000000000000000000
000000000000000000000000000000000000000000000000000000000000000000000000000000000000000000000000000000000000000000000000000000
0000000000000000000000000000000000000000000000000000000000000000000000000000000000000000000000000000000000000000000000000000000000000000000000000000000000000000000000000000000000000000000
000000000000000000000000000000000000000000000000000000000000000000000000000000000000000000000000000000000000000000000000000000
0000000000000000000000000000000000000000000000000000000000000000000000000000000000000000000000000000000000000000000000000000000000000000000000000000000000000000000000000000000000000000000
000000000000000000000000000000000000000000000000000000000000000000000000000000000000000000000000000000000000000000000000000000
>Q8TEQ6
MGQEPRTLPPSPNWYCARCSDAVPGGLFGFAARTSVFLVRVGPGAGESPGTPPFRVIGELVGHTERVSGFTFSHHPGQYNLCATSSDDGTVKIWDVETKTVVTEHALHQHTISTLHWSPRVKDLIVSGDEKGVVFCYWFNRNDSQHLFIEPRTIFCLTCSPHHEDL
VAIGYKDGIVVIIDISKKGEVIHRLRGHDDEIHSIAWCPLPGEDCLSINQEETSEEAEITNGNAVAQAPVTKGCYLATGSKDQTIRIWSCSRGRGVMILKLPFLKRRGGGIDPTVKERLWLTLHWPSNQPTQLVSSCFGGELLQWDLTQSWRRKYTLFSASSEGQNHSR
IVFNLCPLQTEDDKQLLLSTSMDRDVKCWDIATLECSWTLPSLGGFAYSLAFSSVDIGSLAIGVGDGMIRVWNTLSIKNNYDVKNFWQGVKSKVTALCWHPTKEGCLAFGTDDGKVGLYDTYSNKPPQISSTYHKKTVYTLAWGPPVPPMSLGGEGDRPSLALYSC
GGEGIVLQHNPWKLSGEAFDINKLIRDTNSIKYKLPVHTEISWKADGKIMALGNEDGSIEIFQIPNLKLICTIQQHHKLVNTISWHHEHGSQPELSYLMASGSNNAVIYVHNLKTVIESSPESPVTITEPYRTLSGHTAKITSVAWSPHHDGRLVSASYDGTAQVWDALR
EEPLCNFRGHRGRLLCVAWSPLDPDCIYSGADDFCVHKWLTSMQDHSRPPQGKKSIELEKKRLSQPKAKPKKKKKPTLRTPVKLESIDGNEEESMKENSGPVENGVSDQEGEEQAREPELPCGLAPAVSREPVICTPVSSGFEKSKVTINNKVILLKKEPPKEKPETLIK
KRKARSLLPLSTSLDHRSKEELHQDCLVLATAKHSRELNEDVSADVEERFHLGLFTDRATLYRMIDIEGKGHLENGHPELFHQLMLWKGDLKGVLQTAAERGELTDNLVAMAPAAGYHVWLWAVEAFAKQLCFQDQYVKAASHLLSIHKVYEAVELLKSNHFYREA
IAIAKARLRPEDPVLKDLYLSWGTVLERDGHYAVAAKCYLGATCAYDAAKVLAKKGDAASLRTAAELAAIVGEDELSASLALRCAQELLLANNWVGAQEALQLHESLQGQRLVFCLLELLSRHLEEKQLSEGKSSSSYHTWNTGTEGPFVERVTAVWKSIFSLDTPEQY
QEAFQKLQNIKYPSATNNTPAKQLLLHICHDLTLAVLSQQMASWDEAVQALLRAVVRSYDSGSFTIMQEVYSAFLPDGCDHLRDKLGDHQSPATPAFKSLEAFFLYGRLYEFWWSLSRPCPNSSVWVRAGHRTLSVEPSQQLDTASTEETDPETSQPEPNRPSELD
LRLTEEGERMLSTFKELFSEKHASLQNSQRTVAEVQETLAEMIRQHQKSQLCKSTANGPDKNEPEVEAEQPLCSSQSQCKEEKNEPLSLPELTKRLTEANQRMAKFPESIKAWPFPDVLECCLVLLLIRSHFPGCLAQEMQQQAQELLQKYGNTKTYRRHCQTFCM
0000000000000000000000000000000000000000000000000000000000000000000000000000000000000000000000000000000000000000000000000000000000000000000000000000000000000000000000000000000000000000000
0000000000000000000000000000000000000000000000000000000000000000000000000000000000000000000000000000000000000000000000000000000000000000000000000000000000000000000000000000000000000000000
0000000000000000000000000000000000000000000000000000000000000000000000000000000000000000000000000000000000000000000000000000000000000000000000000000000000000000000000000000000000000000000
0000000000000000000000000000000000000000000000000000000000000000000000000000000000000000000000000000000000000000000000000000000000000000000000000000000000000000000000000000000000000000000
0000000000000000000000000000000000000000000000000000000000000000000000000000000000000000000000000000000000000000000000000000000000000000000000000000000000000000000000000000000000000000000
0000000000000000000000000000000000000000000000000000000000000000000000000000000000000000000000000000000000000000000000000000000000000000000000000000000000000000000000000000000000000000000
0000000000000000000000000000000000000000000000000000000000000000000000000000000000000000000000000000000000000000000000000000000000000000000000000000000000000000000000000000000000000000000
0000000000000000000000000000000000000000000000000000000000000000000000000000000000000000000000000000000000000000000000000000000000000000000000000000000000000000000000000000000000000000000
0000000000000000000000000000000000000000000000000000000000000000000000000000000000000000000000000000000000000000000000000000000000000000000000000000000000000000000000000000000000000000000
00000000000000000000000000000000000000000000000000000000000000000000000000000000000000000000000000000000000
0000000000000000000000000000000000000000000000000000000000000000000000000000000000000000000000000000000000000000000000000000000000000000000000000000000000000000000000000000000000000000000
0000000000000000000000000000000000000000000000000000000000000000000000000000000000000000000000000000000000000000000000000000000000000000000000000000000000000000000000000000000000000000000
0000000000000000000000000000000000000000000000000000000000000000000000000000000000000000000000000000000000000000000000000000000000000000000000000000000000000000000000000000000000000000000

00
00
00
00
00
000000000000000000000000000000000000000000000000000000000000000000000000000000000000000000000000000000000000000000000
00
00
00
00
00
00
00
00
000000000000000000000000000000000000000000000000000000000000000000000000000000000000000000000000000000000000000000000
>Q5ZTK4
MPKYVEGVELTQEGMHAIFARMGYGDITSGSIYNGVPTIDTGALNRQGFMPVLTGVGPHRDSGHWIMLIKGPGNQYYLFDPLGKTSGEGYQNILAAQLPMGSTLSVIPNGSGLNMGLCGYWVASAGLRAHQALNQHNPPTLLNVGQTITNEMRNELDHDGY
RKITGWLRAVADEFPEGDPQLDGKALRENTEKDLKIEIPTLVLPGKDTSPKEMSVKPTAPQDKSVPVWNGFSLYTDDTVKAAAQYAYDNYLGKPYTGSVESAPANFGGRMVYRQHHGLSHTLRTMAYAELIVEEARKAKLRGETLGKFKDGRTIADVTPQELKKIMI
AQAFFVAGRDDEASDAKNYQKYHEQSRDAFLKYVKDNESTLIPDVFKDQEDVNFYARVIEDKSHDWESTPAHVLINQGHMVDLVRVKQPPESFLQRYFSSMQRWIGSQATEAVFGIQRQFFHATYEVVAGFDSDNKEPHLVVSGLGRYVIGEDGQPIREAPKKG
QKEGDLKVFPQTYKLKENERLMRVDEFLKLPEIQNTFPGSGKHLQGGMPGMNEMDYWNRLNSLNRARCENDVDFCLKQLQTAHDKAKIEPIKQAFQSSKGKERRQPNVDEIAAARIIQQILANPDCIHDDHVLINGQKLEQQFFRDLLAKCEMAVVGSLLNDTDI
GNIDTLMRHEKDTEFHSTNPEAVPVKIGEYWINDQRINNSSGNITQKKHDLIFLMQNDAWYFSRVNAIAQNRDKGSTFKEVLITTLMTPLTSKALVDTSQAKPPTRLFRGLNLSEEFTKGLIDQANAMIANTTERLFTDHSPEAFKQIKLNDLSKMSGRTNASTTTEIK
LVKETWDSNVIFEMLDPDGLLHSKQVGRHGEGTESEFSVYLPEDVALVPVKVTLDGKTQKGENRYVFTFVAVKSPDFTPRHESGYAVEPFLRMQAAKLAEVKSSIEKAQRAPDLETIFNLQNEVEAVQYSHLSTGYKNFLKNTVGPVLENSLSGLMESDTDTLSKALA
AFPSDTQWSAFNFEEARQAKRQMDAIKQMVGNKVVLDALTQCQDALEKQNIAGALDALKKIPSEKEMGTIRRELREQIQSARQELESLQRAVVTPVVTDEKKVRERYDALIENTSKKITELETGKLPNLDAVKKGISNLSNLKQEVTVLRNEKIRMHVGTDKVDFSD
VEKLEQQIQVIDTKLADAYLLEVTKQISALDNTKPKNQTELKTKIAAFLDRTTDIEMLRNERIKKHGSSKDPLDLSDLDKLSGSLQRINQSLVSDLITTIRVSINQMEAKTFHEQEKEIQQNFELLAKLEKTLDKSKTSEKLREDIPKLNDLLVAKQKAYPQMVQMQLKSEV
FVTQLREVCQANHDDLDKTRNARLRELDRLDREAGITRMVGNLIWGLTNKVGLTTDERLDIRTKQQSLARFKNELFNDKIDTDQLISNLARKRPSELQEGLGISTDNAMELHLLLTELAGKTTSPDELEERMKAIDDISTKIGREPEHLKFVMVEEDESNKKTIGF
00
00
00
00
00
00
00
00
0000000000000000000000000000000000000000000000000000000000000000000000000000000000000000000000000000000000
00
00
00
00
00
00
00
00
0000000000000000000000000000000000000000000000000000000000000000000000000000000000000000000000000000000000
00
00
00
00
00
00
00
00
0000000000000000000000000000000000000000000000000000000000000000000000000000000000000000000000000000000000
